# Machine learning-integrated network toxicology uncovers glioma targets of DEHP

**DOI:** 10.3389/ftox.2026.1771011

**Published:** 2026-05-08

**Authors:** Ren Li, Chaomin Ren, Lu He, Shule Wang, Geng Guo

**Affiliations:** 1 Department of Environmental Health, School of Public Health, Shanxi Medical University, Taiyuan, China; 2 Center for Cerebrovascular Diseases Research, Shanxi Medical University, Taiyuan, Shanxi, China; 3 Cerebrovascular Disease Center, First Hospital of Shanxi Medical University, Taiyuan, Shanxi, China; 4 Department of Public Health Laboratory Sciences, School of Public Health, Shanxi Medical University, Taiyuan, China; 5 Hacken Merdian Health, JFK University Medical Center, Neuroscience Institute, Edison, NJ, United States; 6 Department of Emergency, First Hospital of Shanxi Medical University, Taiyuan, Shanxi, China

**Keywords:** Di(2-ethylhexyl) phthalate, glioma, machine learning, molecular docking, network toxicology, SHAP

## Abstract

**Background:**

Di (2-ethylhexyl) phthalate (DEHP), a ubiquitous environmental plasticizer, is increasingly linked to neurotoxicity and carcinogenesis. However, its role in glioma pathogenesis remains poorly understood. This study integrates network toxicology and machine learning to identify molecular targets of DEHP in glioma.

**Methods:**

Potential DEHP targets were identified through four databases (CHEMBL, CTD, SwissTargetPrediction, PharmMapper). Glioma-related genes were screened using differential expression analysis and weighted gene co-expression network analysis (WGCNA) on GEO and TCGA datasets. Overlapping genes were subjected to functional enrichment, followed by 127 machine learning models to prioritize core genes. SHAP analysis interpreted model contributions, while COX regression assessed prognostic value. Molecular docking and dynamics simulations evaluated binding stability between DEHP and target proteins. *In vitro* validation was performed in U87 cells via RT-qPCR and Western blotting.

**Results:**

A total of 77 overlapping genes were identified, enriched in neuroactive ligand-receptor interactions, GABAergic synapses, and ion channel activity. Machine learning prioritized 12 key genes (e.g., RELA, ABCA1, HIF1A), forming a parsimonious 12-gene diagnostic model with strong external discrimination (pooled validation AUC = 0.994). A high DEHP-related risk score was associated with poorer survival in the TCGA cohort and showed similar prognostic stratification across the external CGGA_325, CGGA_693, and GSE16011 cohorts. Molecular simulations confirmed stable binding between DEHP and core proteins. Experimental validation demonstrated dose- and time-dependent upregulation of RELA, ABCA1, and HIF1A in DEHP-exposed U87 cells.

**Conclusion:**

This integrative approach provides a systems-level framework to prioritize DEHP-associated target genes and molecular signatures in glioma, extending beyond the previously reported PER3-related observation and offering candidate biomarkers for early detection and prognosis under environmental exposure.

## 1 Introduction

Glioma is among the most common primary intracranial tumors and remains a major cause of morbidity and mortality in neuro-oncology. Recent population-based studies continue to show a substantial disease burden and poor outcomes for high-grade glioma, particularly glioblastoma, underscoring the need to better understand its molecular pathogenesis (Ostrom et al., 2023; Bauchet et al., 2025; Królikowska et al., 2025). The high disability rate, high recurrence rate, and side effects during treatment of this tumor significantly affect the physical and psychological health of patients and impose a heavy burden on their families and society, constituting a major challenge in the field of global public health (Pöhlmann et al., 2024; van der Meer et al., 2023; Li et al., 2022a). However, at present, the pathogenesis of glioma has not been fully elucidated. It has been well accepted that a complex interaction of genetic susceptibility and environmental exposures might induce glioma (Lapointe et al., 2018). In terms of genetic factors, hereditary cancer syndromes such as neurofibromatosis type 1 (NF1), neurofibromatosis type 2 (NF2), and Li-Fraumeni syndrome are considered to increase the risk of glioma (Pan et al., 2021; Coy et al., 2020; Kite et al., 2025). In addition, family history and genetic mutations are also strongly associated with the development of glioma (Choi et al., 2023; Yanchus et al., 2022). In terms of environmental factors, high-dose ionizing radiation is considered to be the most definitive causative factor of glioma (Hauptmann et al., 2023). However, other genetic and environmental factors that might induce glioma should be further explored. Moreover, the therapeutic effect is still limited in the case of WHO grade IV glioma, which has a higher degree of malignancy (Weller et al., 2024; Obrador et al., 2024). Therefore, to elucidate the molecular mechanisms of glioma and to explore new therapeutic targets and strategies have become important directions in the field of glioma research.

Di (2-ethylhexyl) phthalate (DEHP), a commonly used plasticizer, has been widely used in food packaging and medical devices, which has been identified as one of the plastic additives posing a significant threat to human health (Symeonides et al., 2024). DEHP is widely distributed in water, atmosphere, and soil. Its volatility and mobility have led to a significant increase in human health risks through respiration, dermal contact, and food ingestion (Zhang et al., 2022b; Zhou et al., 2025). Recent studies have suggested that DEHP may be associated with the development and progression of glioma (Sims et al., 2014). Epidemiological studies have shown significant correlations between DEHP exposure and neurobehavioral and neurodevelopmental problems in children, particularly with regard to attention deficits, learning disabilities, social impairments, and autistic traits (Liu et al., 2023; Bai et al., 2025). Other evidence also suggests that DEHP may be a potential risk factor for a number of cancers, including breast, prostate, and thyroid cancers, in a dose-dependent manner (Zhou et al., 2025; National Toxicology Program, 2021). Experimental animal studies have also shown that DEHP exposure has significant effects on neurobehavioral, emotional, learning, and memory neurological functions, and that early exposure in particular may lead to long-term behavioral and cognitive abnormalities (Li et al., 2022b; Capela and Mhaouty-Kodja, 2021; Sun et al., 2021). Meanwhile, chronic DEHP exposure significantly downregulates the core clock gene PER3, affects neurological function, and has been reported to be associated with glioblastoma-related changes in a zebrafish model (Men et al., 2024). These emerging findings suggest a potential association between DEHP exposure and glioma-related alterations, but direct causal relationships remain to be established. However, the detailed DEHP-gene interactions in the occurrence and development of glioma remain largely unknown and should be further explored.

The rise of network toxicology has brought a revolutionary perspective to the research field, which has provided insights into the mechanisms of toxicant-gene interactions by integrating multi-omics datasets, constructing dynamic network models, and performing computational structure-activity analyses. In studies on DEHP, transcriptomic analysis reveals that DEHP exposure alters functional gene clusters relevant to stress response and inflammatory response in the mouse brain, particularly genes related to endoplasmic reticulum protein processing, such as Hspa5, Hspa1a, and Hspa1b. These changes trigger neuronal apoptosis and neuroinflammation (Yang et al., 2023). Lipidomics studies have revealed that low-dose DEHP exposure causes significant changes in long-chain acylcarnitine levels in humans and mice after birth (Vacy et al., 2025). Molecular docking studies show that ferulic acid inhibits the neurotoxicity of DEHP by docking with cysteine-3 and nitric oxide synthase, thereby improving abnormal brain function and structure (Khalifa et al., 2024). However, all these datasets are relatively isolated and unconnected. It is promising to construct an exposure-response-effect network at the systemic level. To this end, we employed network toxicology to identify potential biological targets through which DEHP may exert effects on glioma. Subsequently, we integrat machine learning, molecular docking, and molecular dynamics simulations to pinpoint specific targets, which are experimentally validated. Given the increasing application of machine learning in toxicology, careful attention should also be paid to issues such as model overfitting, external validation, and interpretability when prioritizing toxicity-associated targets (Ajisafe et al., 2025; Seal et al., 2025). This study aimed to characterize DEHP-associated molecular signatures in glioma through an integrated network toxicology and machine learning framework, and to provide a hypothesis-generating basis for future mechanistic and experimental studies under environmental exposure.

## 2 Methods

### 2.1 Acquire the chemical structure and potential targets of DEHP

The 2D structure of DEHP in PubChem has the following SMILES sequence: CCCCC(CC)COC(=O)C1 = CC = CC = C1C(=O)OCC(CC)CCCC. To comprehensively identify potential biological targets of DEHP, we predicted and summarized DEHP-related targets using four publicly available resources (CHEMBL, CTD, SwissTargetPrediction, and PharmMapper), and then performed a union operation on the results. This target-collection strategy is consistent with published network toxicology studies, in which compound-related targets are first integrated from multiple public resources and then further prioritized through intersection with disease-associated genes or transcriptomic features. Therefore, the union set in the present study was used only as an initial candidate pool, and potential false positives were subsequently reduced through downstream overlap with glioma-associated DEGs and WGCNA-derived genes from the module showing the strongest absolute association with glioma status.

### 2.2 Data sources for glioma

**FIGURE 1:**
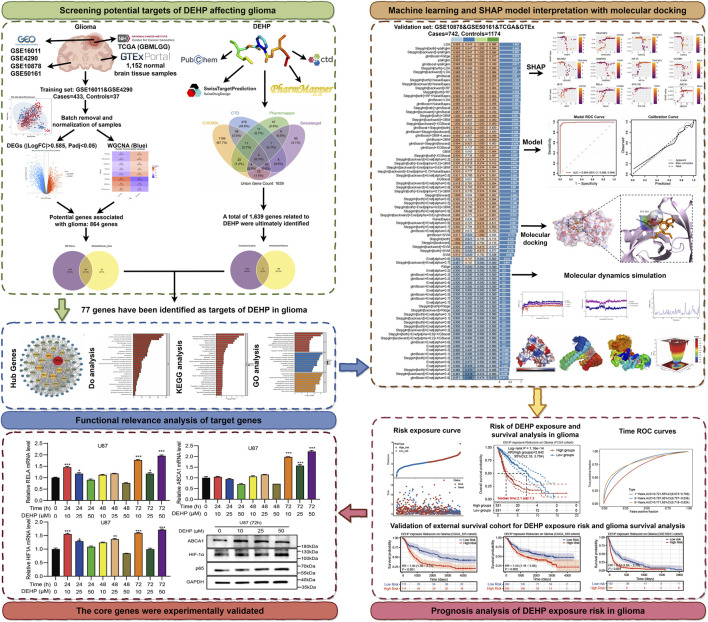
The workflow of this study. First, we identified DEHP target genes that were associated with glioma. Then, we determined the potential target genes of DEHP in glioma. Subsequently, we performed functional enrichment analysis on the target genes to identify the potential biological pathways through which DEHP induced glioma. Finally, we used machine learning to screen the key core genes and to identify the optimal core genes influenced by DEHP in glioma, providing a basis for further analysis. COX prognosis analysis was used to determine the association between DEHP exposure and survival outcomes in glioma patients, with further validation through external population data. The binding stability of core genes with DEHP was validated using molecular docking and molecular dynamics simulation. Ultimately, the U87 glioma cell line will be used for DEHP exposure to observe the expression profile of core genes.

The glioma datasets included four glioma datasets from the Gene Expression Omnibus (GEO) database and glioma data from the Cancer Genome Atlas (TCGA) program. To compensate for the absence of normal tissue data in TCGA, we supplemented the dataset with normal human brain tissue expression data from the Genotype-Tissue Expression (GTEx) database. GSE16011 and GSE4290 were used as the discovery cohorts, comprising 31 normal tissue samples and 433 glioma tissue samples in total. GSE10878, GSE50161, and TCGA glioma data matched with normal brain tissue from the GTEx project were used as independent validation cohorts. These validation datasets comprised a total of 1,174 normal tissue samples and 742 glioma tissue samples and were not used for feature screening or model training. The workflow for screening glioma key genes was shown in Supplementary Figure S1; Figure 1. Supplementary Figure S1 illustrated the complete analytical workflow of this study, while Figure 1 expanded upon this framework by incorporating specific analytical details and schematic representations of partial results. The first one was the target screening module, which identified potential targets through which DEHP might act on glioma. The second was the biological pathway analysis module for these targets. The third was the machine learning module for screening core genes. The fourth was the module for evaluating DEHP exposure risk and glioma prognosis based on core genes. Finally, the module involved selecting core genes for *in vitro* experimental validation. The website links to access of the dataset were provided in Supplementary Table S1.

### 2.3 Screening for glioma genes and identification of functional genes using weights gene co-expression network analysis (WGCNA)

Data processing was performed using the “limma” R package (version 3.54.2), which facilitated handling genes with the same sequence but different expression levels. Therefore, we carried out batch removal and normalization on different datasets using “SVA” R package (version 3.46.0). The minimum gene expression fold change threshold was set to 1.5-fold (|Log Fold Change| ≥ 0.585), and the P-values were adjusted to be less than 0.05 using the BH method (*P.adj* < 0.05). In order to further identify the functional groups involved in glioma functions, we performed a WGCNA analysis on the discovery cohort.

For the corrected expression matrix, genes were retained with a variance greater than 0.5 and high variability. Outliers were removed using the Euclidean distance-based average linkage hierarchical clustering method (hclust parameters: static cut-off height = 20,000; minimum cluster size = 10). In the network construction stage, the optimal soft threshold (power value) was determined using the scale-free topological fit index (R^2^ ≥ 0.9) and the dynamic tree cutting algorithm (deepSplit = 2, minClusterSize = 50) was used to partition the gene modules. Similar modules were then merged with a correlation coefficient >0.75 (merging threshold = 0.25). In the module-trait association analysis, principal component analysis was used to extract the module feature values (MEs). Pearson correlation tests were employed to assess the association between MEs and the binary clinical trait (control group = 1; treatment group = 0) (significance threshold: *P* < 0.05). Additionally, gene module membership (MM, |MM| > 0.8) and gene significance (|GS| > 0.2 and *P* < 0.05) were used to screen for key genes. The module showing the strongest absolute correlation between module eigengene and glioma status was defined as the most disease-relevant module and retained for downstream analysis.

### 2.4 Identification of the targets of DEHP in glioma disease and their functional enrichment analysis

To identify DEHP targets in glioma, we overlapped glioma-associated differentially expressed genes, genes from the WGCNA module showing the strongest absolute correlation with glioma status, and DEHP-related potential targets. Once the core targets had been obtained, we used the “DOSE” package (version 3.24.2) to perform disease enrichment analysis. Simultaneously, we used the “clusterProfiler” package (version 4.4.4) to perform Gene Ontology (GO) and Kyoto Encyclopedia of Genes and Genomes (KEGG) analyses to gain an initial understanding of the potential biological pathways and effects of DEHP on glioma.

### 2.5 Screening of disease-causing genes using 127 machine learning algorithms

We established a comprehensive diagnostic model for the toxic effect of DEHP on glioma using 127 machine learning algorithms, which was used to identify DEHP-related glioma biomarkers. Specifically, multiple machine learning algorithms were employed to develop diagnostic models, including Elastic Net, Lasso, Ridge Regression, Support Vector Machines (SVM), Linear Discriminant Analysis (LDA), Gradient Boosting Machines (GBM), Random Forests (RF) and XGBoost. First, the discovery-cohort data were standardized and random noise was added to enhance robustness. Next, feature selection was performed within the discovery cohorts to identify key variables. A prediction model was then developed and evaluated using 10-fold cross-validation in the discovery cohorts. To further evaluate whether model performance might be influenced by overfitting or class imbalance, we additionally compared the top-performing workflows using retained feature number, precision, recall, F1-score, Matthews correlation coefficient (MCC), and precision-recall analysis in the external validation cohorts. For threshold-dependent metrics, the optimal cutoff was determined in the discovery cohort and then fixed for external validation. The expanded benchmarking results are provided in Supplementary Table S9; Supplementary Figures S8, S9. Heatmaps were used to visualize the importance of key genes and models. All analyses were performed in the R 4.2.0 environment using core R packages such as glmnet, caret, randomForest, SRC and xgboost.

### 2.6 Model interpretation and diagnostic performance analysis of core genes

This study combined a machine learning approach with the SHapley Additive exPlanations (SHAP) interpretability analysis framework to explain the selection of machine learning models. SHAP values were used to quantify the direction and magnitude of each feature gene’s contribution to the model prediction for a given sample. In this study, positive SHAP values indicated that a feature pushed the prediction toward the glioma class, whereas negative SHAP values indicated that it pushed the prediction away from the glioma class. The SHAP values of key genes and their relationship with expression levels were visualized using honeycomb plots, bar charts, and dependency plots. Clinical decision curves (DCA curves) were used to compare the diagnostic utility of the combined model and individual genes. ROC curves were used to evaluate model discrimination. Calibration was assessed using the calibration curve to examine the agreement between predicted and observed outcomes. Prognostic score plots were used to assign specific scores to glioma patients identified by the model.

### 2.7 COX model analysis of the association between the DEHP-related risk score and glioma survival

A prognostic model based on the 12-gene DEHP-related molecular signature was established using multivariable Cox regression analysis. To evaluate the relationship between this DEHP-related risk score and glioma prognosis, the model was further assessed in independent external survival cohorts, including CGGA_325, CGGA_693, and GSE16011. We further analyzed the roles of 12 characteristic genes in gliomas, conducted clinical feature correlation analysis and prognosis survival analysis to increase their potential as biomarkers of DEHP-associated molecular effects in gliomas.

### 2.8 Molecular docking analysis and molecular dynamics simulation

Molecular docking was utilized to identify the binding sites of DEHP and its potential targets in glioma. A sampling molecular docking simulation was used to validate the interaction between DEHP and the core genes identified through screening. The DEHP structure was downloaded from PubChem (https://pubchem.ncbi.nlm.nih.gov/) and converted to 3D PDB format using Discovery Studio 2019. The core genes were retrieved from UniProt (https://www.uniprot.org/) (Consortium, 2024). The PDB IDs with high resolution were selected and were downloaded from the Protein Data Bank website (https://www.rcsb.org/) (Burley et al., 2024). Preprocessing was performed using MOE to remove water and ligands from proteins and to repair proteins using SPDBV. The PDB files of DEHP and the key genes were processed using AutoDock 4.2 and MGLTools 1.5.6. Hydrogen atoms were added and the charges were preserved in the processed PDB files, which were then saved in PDBQT format. The Lamarckian genetic algorithm was applied to the processed DEHP and core genes for 50 docking simulations. After the calculation, a series of energy clusters were generated. The docking model with the lowest binding energy was selected based on the principle of energy optimization. The model was analyzed and visualized using Pymol. We used the Protein-Ligand Interaction Profiler (Schake et al., 2025) to analyze the types of non-covalent interactions. Molecular dynamics (MD) simulations were performed using the Gromacs2022 program. Small molecules were modeled using the GAFF force field, while proteins were modeled using the AMBER14SB force field and the TIP3P water model. Protein and small molecule ligand files were merged to construct the simulated complex system. Simulations were conducted under isothermal and isobaric conditions with periodic boundary conditions. During the MD simulations, all hydrogen bonds were constrained using the LINCS algorithm, with an integration time step of 2 fs. Electrostatic interactions were calculated using the Particle-Mesh Ewald (PME) method with a cutoff value of 1.2 nm. Non-bonded interactions were truncated at 10 Å, with updates every 10 steps. The simulation temperature was controlled at 298 K using the V-rescale temperature coupling method; and the pressure was maintained at 1 bar using the Berendsen method. At 298 K, 100 ps of NVT and NPT equilibration simulations were performed, followed by 100 ns of MD simulations of the complex system, with conformations saved every 10 ps. After the simulations, the simulation trajectories were analyzed using VMD and Pymol; and the MMPBSA binding free energy analysis between the protein and small-molecule ligands was performed using the g_mmpbsa program.

### 2.9 Cell culture and DEHP preparation

Human glioma U87 cells were obtained from the ATCC. U87 cells were cultured in DMEM medium with 10% FBS and 1% penicillin/streptomycin. Cells were incubated at 37 °C with 5% CO_2_. When U87 cells reached 80% confluence, they were treated with 10, 25, or 50 μM DEHP (MCE, Catalog No. HY-B1945) for 24, 48, or 72 h, respectively. Cells treated with 0 μM DEHP served as the untreated control group for all *in vitro* analyses. The selected DEHP concentrations (10–50 μM) and exposure durations (24–72 h) were intended to capture short-term, dose- and time-dependent cellular responses *in vitro* for mechanistic exploration, rather than to directly model chronic human internal exposure levels.

### 2.10 Reverse transcription-quantitative PCR (RT-qPCR)

Total RNAs were extracted from U87 cells using TRIzol reagent (Invitrogen, USA). Subsequently, reverse transcription and quantitative real-time PCR (qPCR) was performed. cDNA synthesis was conducted according to the manufacturer’s instructions, and qPCR was carried out using a SuperReal PreMix Color (SYBR Green) kit (Tiangen, China). The primer sequences were in Table 1. The levels of mRNAs were expressed as 2^−ΔΔCT^, where Ct was cycle threshold, ΔCt = testing gene (Ct) - average β-actin (Ct), and ΔΔCt = sample group Δ(Ct) - average control group Δ(Ct). All experiments were replicated thrice. Data from RT-qPCR and Western blot quantification are presented as mean ± SD from three independent experiments. For RT-qPCR, differences among DEHP concentrations were analyzed at each time point using one-way ANOVA followed by Dunnett’s multiple-comparisons test against the 0 μM control group.

**TABLE 1:** qPCR experimental details and primer sequences.

Gene symbol	Primer direction	Sequence (5’→3’)	Function
RELA	Forward	AGTGTGTGAAGAAGCGGGACCT	Target gene
	Reverse	GCACTGTCACCTGGAAGCAGAG	
ABCA1	Forward	ACCCACCCTATGAACAACATGA	Target gene
	Reverse	GAGTCGGGTAACGGAAACAGG	
HIF1A	Forward	AGTTCCGCAAGCCCTGAAAGC	Target gene
	Reverse	TCAGTGGTGGCAGTGGTAGTGG	
β-actin	Forward	CATGTACGTTGCTATCCAGGC	Endogenous control
	Reverse	CTCCTTAATGTCACGCACGAT	

### 2.11 Western blotting (WB)

Total proteins were extracted from U87 cells using radioimmunoprecipitation assay lysis buffer and were quantified with a bicinchoninic acid assay kit. Equal amounts of proteins were then separated by 10% sodium dodecyl sulfate-polyacrylamide gel electrophoresis and transferred onto 0.45-μm polyvinylidene difluoride membranes. Following blocking with 5% bovine serum albumin, the membranes were incubated with primary antibodies overnight at 4 °C. The primary antibodies were ABCA1 polyclonal antibody (cat#: 26564-1-AP; proteintech, dilution rate, 1:1000), p65 polyclonal antibody (cat#: 10745-1-AP; proteintech, dilution rate, 1:1000), HIF1A polyclonal antibody (cat#: 20960-1-AP; proteintech, dilution rate, 1:1000), and GAPDH monoclonal antibody (cat#: 60004-1-Ig; proteintech, dilution rate, 1:1000). Subsequently, the membrane was probed with a horseradish peroxidase (HRP)-conjugated secondary antibody. The immunoreactive bands were then visualized using an ECL detection kit and captured with a Servicebio imaging system (SCG-W3000). The intensity of each band was quantified by ImageJ. The value of each band density in experimental and control groups was normalized to its corresponding loading control. For Western blot quantification at 72 h, differences among groups were analyzed using one-way ANOVA followed by Dunnett’s multiple-comparisons test. A two-sided *P* < 0.05 was considered statistically significant.

## 3 Result

### 3.1 Identification of DEHP-related and glioma-related target genes

**FIGURE 2:**
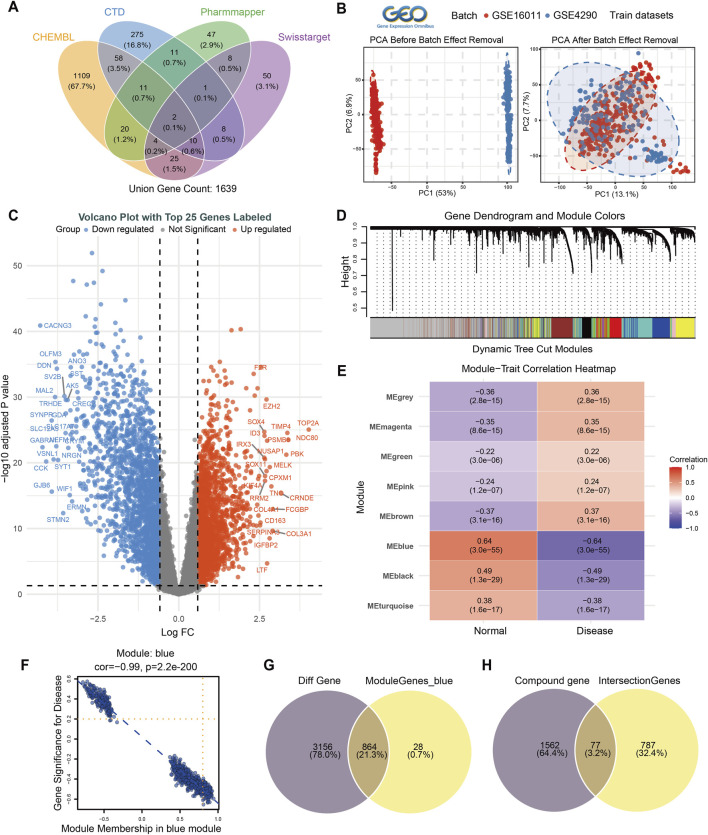
Identification of potential biological targets of DEHP in glioma. **(A)** Four public databases were employed to predict the potential targets of DEHP. The amalgamation of results from these databases yielded a total of 1,639 genes that were associated with DEHP. **(B)** GSE16011 and GSE4290 were utilized as training datasets and underwent batch removal and correction. The left side of the figure displayed the data prior to the implementation of batch removal. whereas the right side illustrated the data subsequent to the execution of batch removal. Batch removal diminished the heterogeneity of the samples. **(C)** The volcano plot (x-axis: LogFC; y-axis: −log10 adjusted P value) illustrated the significantly differentially expressed genes and highlighted the top 25 genes with the largest expression differences. **(D,E)** WGCNA analysis was performed on the training set. As illustrated in Figure D, the modules generated by clustering were displayed, while Figure E demonstrated the correlation between the modules and the disease, along with the corresponding P-values. The blue modules demonstrated a marked correlation with the disease, indicating that the genes contained within these modules were of significant importance. **(F)** Module correlation analysis demonstrated a robust correlation between blue modules and diseases, suggesting a significant association. **(G)** The intersection of the differential genes with the blue module genes yielded 864 genes associated with glioma. **(H)** The genes associated with glioma and DEHP were intersected to identify 77 genes that might be potential targets of DEHP in glioma.

To identify target genes that were associated with DEHP, four databases were consulted: CHEMBL, CTD, Swiss Target Prediction, and Pharmmapper. Among them, CHEMBL identified 1,239 target genes associated with DEHP (Supplementary Table S2). The CTD database revealed 376 relevant genes (Supplementary Table S3), the Pharmmapper database (The Pharmmapper database requires the import of DEHP SDF format files) obtained 104 relevant genes (Supplementary Table S4), and the Swiss Target Prediction database identified 108 relevant genes (Supplementary Table S5). A total of 1,639 DEHP-related target genes were finally identified (Figure 2A).

Subsequently, the module genes identified by differential gene expression and WGCNA analysis were utilized to collectively ascertain the target genes that were associated with glioma. The glioma datasets GSE16011 and GSE4290 were selected from the GEO database to serve as the training set. In order to facilitate reasonable comparison and data processing, batch removal and normalization were performed on the samples (Figure 2B). After performing differential gene analysis between the normal group and the disease group (|LogFC| ≥ 0.585 & *P.adj* < 0.05), a total of 4,020 significantly differentially expressed genes were identified. The volcano plot highlights the top 25 genes with the highest or lowest expression levels (Figure 2C). Next, WGCNA identified eight gene modules (Figure 2D). Module–trait correlation analysis showed that the blue module had the strongest absolute association with glioma status among all identified modules and was therefore selected for downstream analysis (Figure 2E). Correlation analysis further demonstrated a significant negative association between genes in the blue module and disease status (cor = −0.99, P = 2.2e−200) (Figure 2F). This pattern suggests relative suppression of the blue-module program in glioma compared with normal brain tissue, supporting the interpretation that its downregulation may represent a disease-associated hallmark rather than implying that every gene in the module is individually protective. Intersecting the 892 genes in the blue module with the differentially expressed genes yielded 864 glioma-associated genes (Figure 2G; Supplementary Table S6). Further overlap of these glioma-associated genes with the DEHP-related target set identified 77 potential DEHP targets in glioma (Figure 2H).

### 3.2 Construction of core gene networks and functional and pathway analysis

**FIGURE 3:**
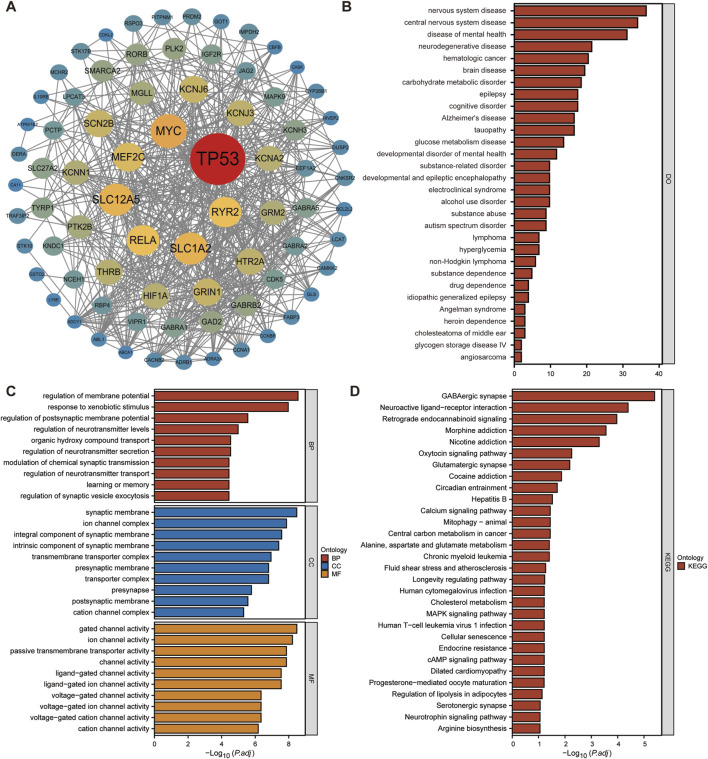
Functional enrichment analysis of potential target genes. **(A)** The molecular network diagram indicated that TP53, MYC, RELA, and other genes played pivotal roles among these 77 genes. **(B)** A disease enrichment analysis was performed using 77 genes and the top 30 results were presented. The aforementioned genes have been found to be primarily associated with developmental and epileptic brain disorders, substance abuse, and drug dependence. **(C)** The bar chart provided a visual representation of the biological functions, cellular components, and molecular functions of these genes, as analyzed by GO analysis. The primary function has involved the regulation of membrane potential, neurotransmitter regulation, and neurotransmitter synapses, gating, and ion channel activity. **(D)** KEGG analysis suggested that these genes might be associated with GABAergic synapses and interactions between neuroactive ligands and receptors.

A PPI network was constructed for 77 genes, and then Cytocsape was used to identify core genes, revealing that TP53, MYC, RELA, and other genes played pivotal roles (Figure 3A). Disease enrichment analysis had shown that these 77 genes were mainly associated with neurological disorders, central nervous system disorders, mental health disorders, neurodegenerative diseases, and other conditions (Figure 3B) (all results were presented in Supplementary Table S7). GO analysis showed that these genes were mainly related to membrane potential regulation, response to heterogenous stimuli, regulation of synaptic vesicle exocytosis, synaptic membrane, ion channel complexes, components of synaptic membranes, ligand-gated ion channel activity, and voltage-gated channel activity (Figure 3C) (Supplementary Table S8). KEGG analysis suggested that these genes were mainly related to GABAergic synapses, interactions between neuroactive ligands and receptors, retrograde endocannabinoid signaling, morphine addiction, nicotine addiction, and oxytocin signaling pathways (Figure 3D) (Supplementary Table S8).

### 3.3 Machine learning identified key genes and provided SHAP model explanations

**FIGURE 4:**
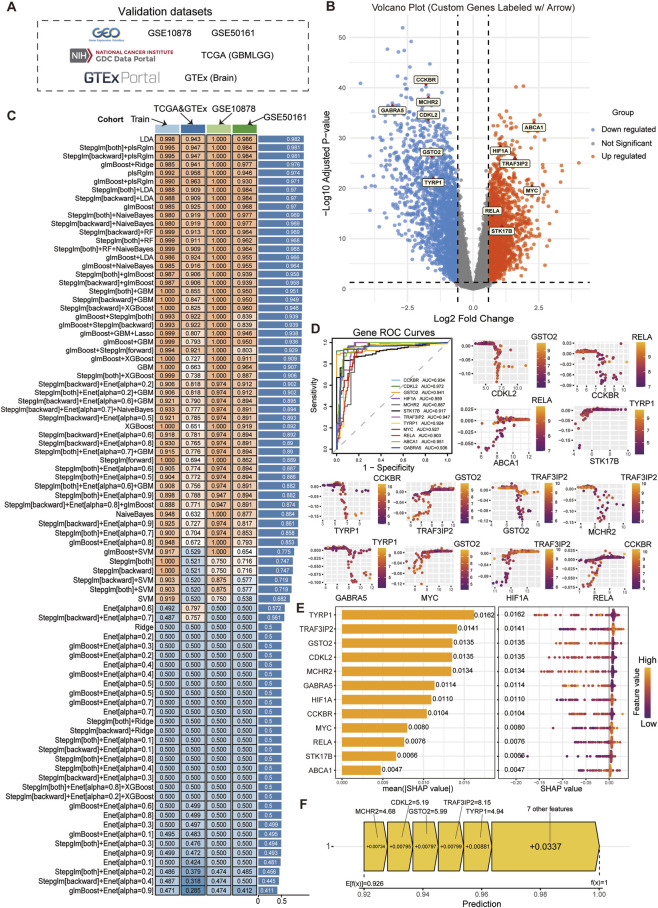
Machine learning and SHAP model explanation. **(A)** The data for validation encompassed GSE10878 and GSE50161 from the GEO database, in conjunction with TCGA glioma data that were matched with the GTEx normal human brain tissue database. **(B,C)** A total of 127 candidate machine-learning workflows were benchmarked using the 77 intersecting genes. Although LDA achieved the highest ROC in the initial comparison, it retained all 77 candidate genes and was therefore not selected for downstream analysis. glmBoost + Ridge was chosen as the final model because it maintained strong external diagnostic performance while reducing the feature set to a compact 12-gene signature. Expanded benchmarking of the top-performing models is provided in Supplementary Table S9; Supplementary Figure S8. **(D)** The AUC curves of the 12 characteristic genes in the diagnosis of glioma all exceeded 0.85. Furthermore, the SHAP dependence plot provided a visual representation of the interaction effects between different feature genes, with the X-axis denoting gene expression levels and the Y-axis representing SHAP values. **(E)** Bar charts and honeycomb charts were more intuitive in showing the distribution and direction of the contribution of feature genes to the model. The most significant contributing gene was TYRP1, with a SHAP value of 0.0162, suggesting that its high expression promoted the model prediction of glioblastoma. **(F)** The model predicted that an increase in MCHR2 increased the probability of a glioma diagnosis by 0.00734 in a given sample.

Among the 77 genes that might be affected by DEHP in glioma, we used 127 machine learning algorithms to establish diagnostic models. We obtained the GSE datasets GSE10878 and GSE50161 and matched TCGA data from normal brain tissue as three independent validation datasets for model construction (Figure 4A). All candidate machine-learning workflows were benchmarked in the discovery cohort and further evaluated in independent validation cohorts. The initial benchmarking showed that LDA achieved the highest ROC in the discovery-stage comparison, whereas glmBoost + Enet [alpha = 0.9] showed the poorest discrimination (Figure 4C; Supplementary Table S9). However, model selection in the present study was not based on ROC alone. Because LDA retained all 77 candidate genes, it was not carried forward for downstream analysis due to limited feature parsimony and reduced tractability for SHAP interpretation, clinical correlation, survival modeling, and experimental validation. We therefore selected glmBoost + Ridge for downstream analysis because it preserved strong external diagnostic performance while reducing the model to a compact 12-gene signature. Expanded benchmarking of the top-performing models, including retained feature number, precision, recall, F1-score, MCC, and precision-recall analysis, is provided in Supplementary Table S9; Supplementary Figure S8. Twelve model genes were identified, six of which exhibited high levels of expression in the disease group, namely, ABCA1, HIF1A, TRAF3IP2, MYC, RELA, and STK17B; whereas the other six demonstrated low levels of expression in the disease group, namely, CCKBR, MCHR2, GABRA5, CDKL2, TYRP1, and GSTO2 (Figure 4B). Then, we used the SHAP framework to interpret how each feature gene contributed to glioma classification in the selected model. In this context, the SHAP value reflects the contribution of a gene to model prediction rather than a direct biological effect size. Positive SHAP values indicate that the feature drives the prediction toward the glioma class, whereas negative SHAP values indicate that it shifts the prediction away from the glioma class. The SHAP dependence plot showed the interaction effects between genes and the direction in which each feature gene influenced model prediction (Figure 4D). Bar charts and honeycomb charts were used to illustrate both the magnitude and direction of feature contributions. Genes such as RELA, HIF1A, and ABCA1 were biologically noteworthy because their SHAP contributions were consistent with glioma-relevant processes including inflammatory signaling, hypoxia/metabolic adaptation, and lipid transport/drug resistance, respectively. Therefore, SHAP was used here to prioritize biologically plausible DEHP-associated feature genes for downstream interpretation and validation (Figures 4E–F).

### 3.4 The clinical application value of the model

**FIGURE 5:**
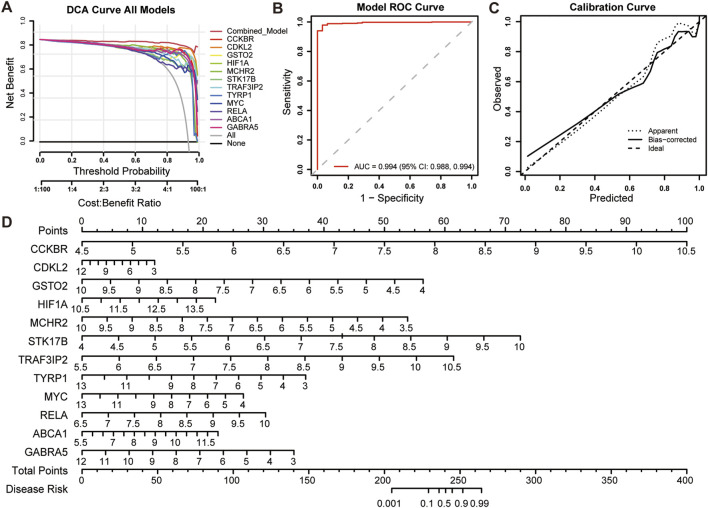
Model evaluation. **(A)** The DCA decision curve evaluated machine learning models and single-gene diagnostic models. The combined model outperformed the 12 individual genes in terms of diagnostic performance. **(B)** ROC analysis in the independent validation datasets showed that the diagnostic model achieved an AUC of 0.994. **(C)** The calibration curve was used to assess the agreement between predicted and observed outcomes. **(D)** The Prognosis line chart displayed the specific scores that had been predicted by the model for the disease. Additional precision-recall analyses for the top-performing workflows and cohort-specific PR curves for glmBoost + Ridge are shown in Supplementary Figures S8, S9.

The clinical translation of models requires careful consideration. To this end, we evaluated clinical models, analyzed the clinical relevance of genes, and conducted prognostic survival analysis. The clinical translation of prediction models requires careful evaluation. To this end, we assessed the model using DCA, ROC analysis, and calibration analysis. The DCA curves showed that the net benefit of the 12-gene model was consistently higher than that of any single-gene model, suggesting potential clinical utility (Figure 5A). In the independent validation datasets, the model achieved an AUC of 0.994, indicating strong diagnostic discrimination (Figure 5B). Consistent with the ROC-based findings, precision-recall analysis of the top-performing workflows and cohort-wise PR curves for glmBoost + Ridge supported robust positive-class discrimination in external validation (Supplementary Figures S8, S9). The calibration curve (Figure 5C) suggested acceptable agreement between predicted and observed outcomes. Based on these results, a nomogram was constructed to provide an intuitive estimate of individual diagnostic scores for glioma prediction (Figure 5D).

### 3.5 COX prognostic analysis showed that a high DEHP-related risk score was associated with poorer glioma outcomes

**FIGURE 6:**
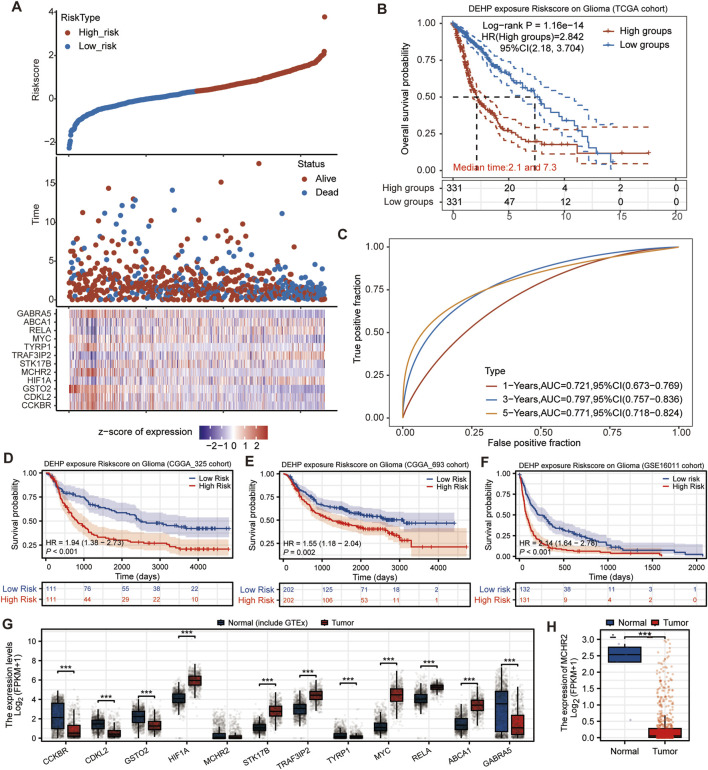
COX prognostic analysis further clarified the association between the DEHP-related risk score and glioma prognosis. COX prognostic analysis further clarified the association between the DEHP-related risk score and glioma prognosis. With increasing DEHP-related risk score, more deaths were observed in the high-risk group. Three independent external survival cohorts (CGGA_325, CGGA_693, and GSE16011) were used to further evaluate survival stratification by the DEHP-related risk score. **(A)** Sample risk calculation: the upper part showed the sample risk score. the middle part represented the survival status. And the lower part showed the expression heat map of model genes. With the increase in DEHP exposure risk, the number of glioblastoma deaths was increased. **(B)** KM survival curve: Comparisons between different groups were performed using the Log-rank test. HR (high-risk group) represented the hazard ratio of the high-risk group relative to the low-risk group. If HR > 1, the model was considered a risk model. if HR < 1, the model was considered a protective model. **(C)** In addition, the ROC curves and AUC values of the risk model at different time points showed that the higher was the AUC value, the stronger was the predictive ability of the model. **(D–F)** Three external survival cohorts further demonstrated the survival status of high-risk glioma patients stratified by the DEHP-related risk score. Patients in the high-risk group had poorer outcomes. **(G,H)** Analysis of mRNA expression levels of model genes in TCGA and GTEx combined data.

A Cox regression analysis was used to establish a DEHP-related risk score model for glioblastoma patients based on 12 genes. Risk curve analysis of the risk model revealed that the number of patients who died was increased with increasing DEHP exposure risk (Figure 6A). The KM curve also revealed that glioblastoma patients with high exposure risk to DEHP in the TCGA survival cohort had lower survival capacity (divided into high-risk and low-risk groups based on the median risk value). Among them, the median survival time in the high-risk group was 2.1 years, and the median survival time in the low-risk group was 7.3 years (Figure 6B). The ROC curve evaluated the predictive potential of the model at 1, 3, and 5 years, and found that the ROC values were all above 0.7, indicating high accuracy (Figure 6C). In addition, to increase the extrapolation of this model, we combined external glioma survival cohorts to assess the impact of DEHP-associated molecular effects on glioma survival. CGGA_325 (including 222 newly diagnosed glioma patients) (Figure 6D), CGGA_693 (including 404 newly diagnosed glioma patients) (Figure 6E), and GSE16011 (including 263 glioma patients, most of whom were high-grade glioblastoma patients) (Figure 6F) were three external population survival cohorts. The results were consistent that patients in the high-risk group for DEHP exposure had reduced survival capacity. Additionally, we analyzed the mRNA levels of model genes in normal tissues (including normal brain tissues from GTEx) and glioma tissues. Except for MCHR2, all other genes showed significant expression differences (*P* < 0.001) (Figure 6G). Compared with adjacent normal tissues, the expression of MCHR2 is significantly reduced in tumor tissues (Figure 6H).

### 3.6 Characteristic genes and clinical features of glioma and independent gene survival analysis

**FIGURE 7:**
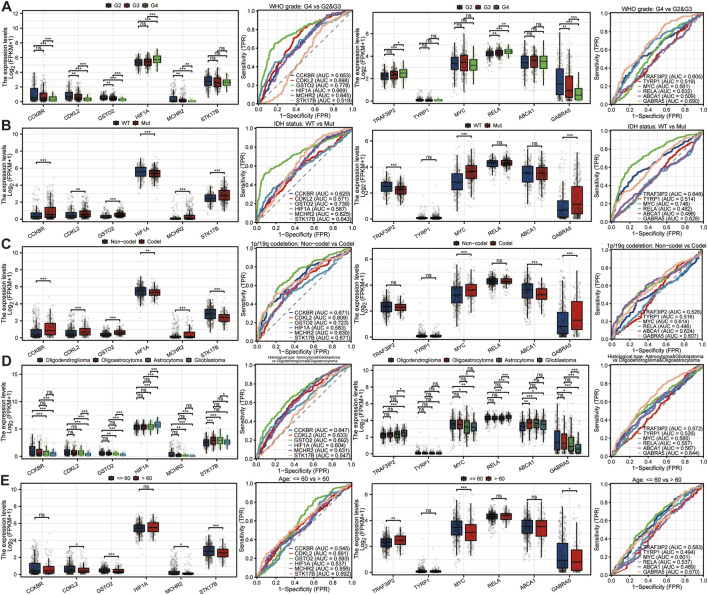
Analysis of the clinical relevance of characteristic genes. The clinical relevance of model genes should be evaluated based on the clinical characteristics of gliomas, including WHO grade **(A)**, IDH mutations **(B)**, 1p/19q co-deficiency **(C)**, histological subtypes **(D)**, and age **(E)**. The expression of model genes in these characteristics must be analyzed, as well as their potential to distinguish clinical features. These analyses also support the interpretation that the clinical relevance of the selected markers may differ across low-grade and high-grade glioma contexts.

To determine the value of characteristic genes as biomarkers for DEHP’s impact on glioma, we conducted a clinical feature analysis of the characteristic genes. We analyzed the relationship between 12 characteristic genes and glioma clinical features and evaluated the ability of different genes to identify their difference. In the WHO grading system for glioma, we found that STK17B, TYRP1, MYC, and ABCA1 did not show any differences in expression across different grades of glioma (Figure 7A). Among them, GSTO2 had a high ability to identify high-grade glioma (AUC = 0.778). Similarly, in IDH mutations, TYRP1, RELA, and ABCA1 showed no significant expression differences between the mutation group and the wild-type group. MYC had the highest ability to identify this clinical feature, with an AUC value of 0.746 (Figure 7B). In the clinical phenotype of 1p/19q co-deletion, TRAF3IP2 and RELA showed no significant differences between the coding and non-coding groups, and the gene GSTO2 had the strongest ability to identify this clinical feature (AUC = 0.723) (Figure 7C). Among the different pathological types of glioma, there were four main types: oligodendroglioma, oligoastrocytoma, astrocytoma, and glioblastoma. The first two were of lower grade, while the latter two were of higher grade and more malignant. All genes showed expression differences across different pathological types, and GSTO2 still exhibited the highest discriminatory ability (AUC = 0.692) (Figure 7D). In the final clinical characteristics, glioma patients were divided into two groups: those ≤60 years old and those >60 years old. It was found that only GSTO2, STK17B, and MYC showed significant expression differences in different groups, and all characteristic genes showed general diagnostic ability (Figure 7E). In summary, the 12 model feature genes had certain application value to identify key clinical features of glioma. These findings suggest that, although the 12-gene model was developed as a glioma-versus-normal classifier, several constituent genes may show stronger biological relevance in higher-grade and more aggressive glioma subtypes.

Finally, we integrated three external survival cohorts and the TCGA cohort (total four survival cohorts) to analyze the predictive ability of model features for survival in glioblastoma patients. After screening, we included 222 glioblastoma samples from CGGA_325, 404 samples from CGGA_693, and 263 samples from GSE16011 (all glioblastomas were primary tumors). The TCGA cohort included 698 glioma samples. Comprehensive analysis revealed that nearly all model feature genes exhibited significant prognostic differences between high- and low-expression groups (Supplementary Figures S2, S3). However, in the survival cohort primarily based on GSE16011 (mainly glioblastoma type), only ABCA1, GSTO2, MYC, and RELA showed significant survival differences. Therefore, these genes exerted an influence on glioma under the sole exposure of DEHP.

### 3.7 Molecular docking and kinetic simulations evaluate the binding stability of DEHP with target genes

**FIGURE 8:**
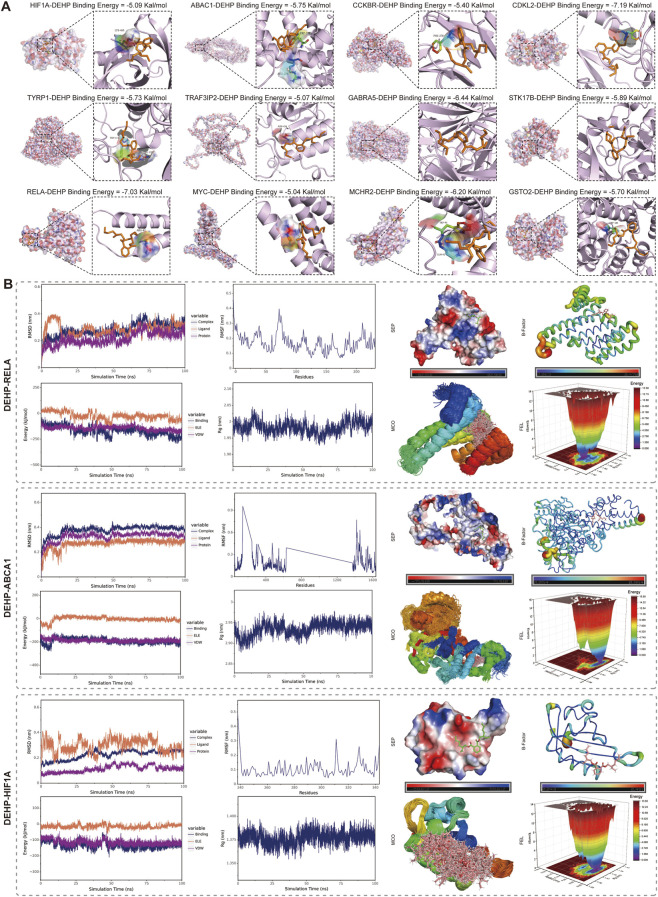
Molecular docking and molecular dynamics simulation analysis. **(A)** The molecular docking of DEHP to each of 12 major proteins with all binding energy < −1.2 kcal/mol. **(B)** We selected three proteins that were most associated with the clinical characteristics of glioma for MD simulations: RELA, ABCA1, and HIF1A. For each protein, we calculated the RMSD of the protein before or after binding to DEHP, the RMSF of the protein in the complex, the Rg of the complex, and the landscape. The binding of the three proteins to DEHP was relatively stable, with van der Waals forces as a major role. Note: RMSD: Root Mean Square Deviation. RMSF: Root mean square fluctuation. SEP: Surface Electrostatic Potential. Rg: Radius of Gyration. MCO: Molecular Conformation Overlaps. FEL: Free Energy Landscape. These docking and MD analyses support relative binding stability and candidate target compatibility, but should not be interpreted as definitive proof of direct intracellular target engagement.

The selected core genes were subjected to molecular docking with DEHP. The binding conformation with the lowest energy among the 50 simulations was selected. DEHP was represented in orange, and the colored regions indicated the amino acid residues that formed hydrogen bonds with DEHP. Yellow bonds indicated hydrogen bonds formed during docking. The formation of stable binding conformations between DEHP and the core residues supported potential molecular-level compatibility between DEHP and the selected core residues (Figure 8A). The binding energies, binding amino acid residues, and types of non-covalent interactions between the core residues and DEHP were presented in Supplementary Table S10. Furthermore, all binding energies < −1.2 kcal/mol and the formation of hydrogen bonds suggested that the binding conformations between DEHP and the core residues were relatively stable.

We selected three representative proteins, RELA, ABCA1, and HIF1A, for MD simulations based on their biological relevance to inflammation, hypoxia/metabolic adaptation, and lipid transport/drug resistance (Figure 8B). The figure mainly showed the root mean square fluctuation (RMSF), root mean square deviation (RMSD), radius of gyration (Rg), the distance between the binding sites of the protein and small molecules, the buried solvent accessible surface area (Buried SASA), and the free energy landscape (FEL) in molecular simulations, all of which were used to illustrate the binding stability between the ligand and the protein. The remaining parameters were shown in the supplementary figures and Supplementary Material. All indicators suggested that DEHP bound stably to RELA, ABCA1, or HIF1A. The binding energy analysis showed that van der Waals forces played a major role, while hydrophobic and electrostatic forces played minor and complementary roles, respectively. The final binding energy between RELA and DEHP was −139.161 ± 4.246 kJ/mol, and the binding energy between ABCA1 and DEHP was −200.408 ± 0.821 kJ/mol, and the binding energy between HIF1A and DEHP was −114.177 ± 0.552 kJ/mol. These results indicated that the binding energies and affinities between small molecules and proteins were relatively high. Specific combination parameters and details were provided in the (Supplementary Figures S4, S6).

### 3.8 Expression of core target genes in DEHP exposure

**FIGURE 9:**
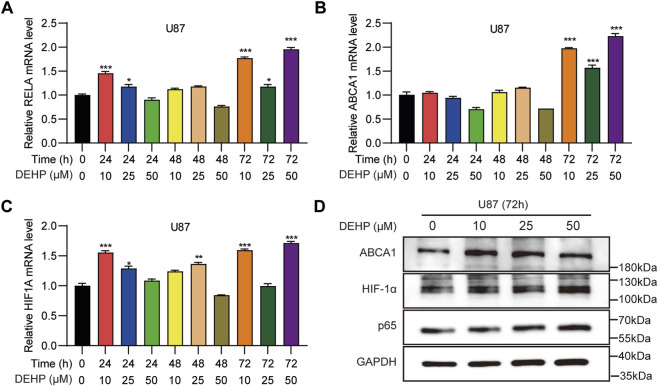
Initial proof-of-concept validation of core gene expression in U87 cells. **(A–C)** Relative mRNA levels of RELA, ABCA1, and HIF1A in U87 cells treated with 0, 10, 25, or 50 μM DEHP for 24, 48, or 72 h. **(D)** Quantified protein expression of RELA/p65, ABCA1, and HIF1A in U87 cells after 72 h DEHP exposure. Data are presented as mean ± SD from three independent experiments. Statistical significance was assessed by one-way ANOVA followed by Dunnett’s multiple-comparisons test versus the 0 μM control group. *P < 0.05, **P < 0.01, ***P < 0.001.

To provide initial proof-of-concept validation of the computationally prioritized targets, we conducted cell assays in the glioma cell line U87. Cells were exposed to 0, 10, 25, or 50 μM DEHP for 24, 48, or 72 h. Among the 12 predicted major genes, we selected RELA, HIF1A, and ABCA1 for experimental validation based on their biological relevance and potential translational significance. RELA was a critical subunit in the NF-κB pathway, regulating multiple pro-inflammatory cytokines (e.g., IL-6 and TNF-α), and it was involvement in the development of brain tumors (Wang et al., 2019). Although DEHP exposure had been reported to activate the NF-κB pathway and promote inflammation, the direct research in glioma had not been conducted (Li et al., 2024b). HIF1A was highly expressed in gliomas and was involved in glioma progression, chemoresistance, and metabolic reprogramming (Ding et al., 2021; Liao et al., 2022). However, no studies had been reported on the association between DEHP and HIF1A. ABCA1 activated temozolomide resistance in glioblastoma (Wang et al., 2021); however, no studies had been conducted on the association between DEHP and ABCA1. Compared with the untreated control group, RELA mRNA expression showed statistically significant increases at selected DEHP concentrations and time points, as indicated in Figure 9A. ABCA1 mRNA expression showed statistically significant elevation mainly at later exposure durations (Figure 9B). HIF1A mRNA expression also showed statistically significant increases at higher concentrations and prolonged exposure times (Figure 9C). Western blot analysis at the 72-h time point further demonstrated significant upregulation of RELA, ABCA1, and HIF1A in DEHP-exposed U87 cells compared with the untreated control group (Figure 9D). Therefore, these experiments support the responsiveness of RELA, ABCA1, and HIF1A to DEHP exposure in U87 cells, although broader validation in additional glioma models is still needed.

## 4 Discussion

Glioma are the most common primary intracranial tumors, originating from glial cells, accounting for approximately 30% of all primary brain tumors, with 80% being malignant. Due to their high disability rate and recurrence rate, glioma severely impact patients' physical and mental health, placing a significant burden on families and healthcare systems, making their clinical management highly challenging. Although there are advances in treatment modalities such as chemotherapy and radiotherapy, the prognosis for high-grade glioma remains poor, highlighting the urgent need for new treatment strategies and a deeper understanding of the underlying molecular mechanisms of glioma development. Current research indicates that the development of glioma is influenced by the combined effects of genetic susceptibility and environmental factors, necessitating further investigation into potential risk factors. DEHP has been identified as a factor influencing glioblastoma with a high carcinogenic potential (Men et al., 2024). This study aims to elucidate the molecular mechanisms underlying glioma development that is associated with DEHP exposure through the application of network toxicology and machine learning. Through extensive database mining, we integrated multi-omics data and identified 1,639 DEHP-related target genes. We then performed differential gene expression analysis and WGCNA to identify 77 genes. Based on 127 machine learning algorithms to construct robust diagnostic models, we ultimately identify 12 key feature genes with significant clinical relevance. Additionally, we conduct functional enrichment analysis to explore biological pathways potentially linking DEHP exposure to glioma-related molecular changes, providing a theoretical framework for future diagnostic and therapeutic strategies. Subsequently, molecular docking and kinetic simulation validated the stability of the binding between DEHP and the identified proteins. The MD simulations were performed in a standard explicit-water system to evaluate relative protein-ligand binding stability. This setup does not fully recapitulate the complex glioma microenvironment, which includes membrane-associated, metabolic, and hypoxic contexts. Finally, the 12 proteins are further screened and validated by experimental assays, ultimately giving three key proteins, RELA, ABCA1, and HIF1A. This study provides a new methodology to identify key genes associated with DEHP-related molecular signatures in glioma. This study not only deepens our understanding of the interactions between environmental toxins and glioma pathogenesis but also highlights the potential biomarkers and therapeutic targets in clinical practice. However, because the *in vitro* validation was performed in a single glioma cell line (U87), the generalizability of these findings remains limited. Future studies should validate these responses in additional glioma models, such as U251 and other established glioma cell systems, to better account for glioma heterogeneity.

In the present study, SHAP analysis was used as an interpretability tool to prioritize genes according to their contribution to glioma classification within the model, rather than to infer direct biological causality. The biological relevance of the SHAP-prioritized genes was supported by known glioma-related pathways: for example, RELA is linked to NF-κB-mediated inflammatory signaling, HIF1A is associated with hypoxia and metabolic adaptation, and ABCA1 is implicated in lipid transport and therapy resistance. These pathways are biologically compatible with the inflammatory, metabolic, and stress-response alterations that may underlie DEHP-associated molecular changes in glioma.

A similar interpretive principle applies to the WGCNA results. The strong negative correlation of the blue module with glioma status does not necessarily indicate that every individual gene within this module functions as a strictly protective factor. Instead, combined with the enrichment results, this pattern suggests that the blue module captures normal neuronal and synaptic programs that are relatively suppressed in glioma. In particular, the genes in this module were enriched in synaptic membrane components, ion channel activity, GABAergic synapse, and neuroactive ligand-receptor interaction pathways, all of which are more consistent with normal neural functional states than with canonical oncogenic activation. Therefore, the negative correlation is more appropriately interpreted as reflecting downregulation of normal neuronal/synaptic characteristics in the disease state and may represent a hallmark of glioma-associated transcriptional reprogramming.

Phthalates, as indispensable additives in modern industrial production, are widely used in plastic products to enhance their flexibility and durability. Among these, DEHP is the most commonly used phthalate plasticizer. Due to its high production volume and widespread application, it has become a common environmental pollutant. Since DEHP does not form covalent bonds with the plastic matrix, it is easily released from plastic products into the environment, leading to widespread human exposure. Particularly in the medical field, patients undergoing treatments such as hemodialysis face significantly high risks of exposure to high doses of DEHP (Shih et al., 2024). Studies have examined the effects of DEHP on cancer and found that it can promote tumor development and regulate the immune microenvironment (Wang et al., 2024b). This increases tumor resistance to immunity and produces drug resistance (Wang et al., 2024a). First, DEHP has been reported as a recognized liver carcinogen. Feige et al. conduct a lifetime low-dose DEHP exposure study (up to 300 mg/kg/d) on Sprague-Dawley rats and find a significant increase in the incidence of liver and testicular tumors, with testicular tumors appearing earlier than hepatocellular tumors, suggesting that the testes may be a new target organ for DEHP(Voss et al., 2005). Lee et al. also find that DEHP suppresses the proinflammatory function of M1-type macrophages by regulating macrophage polarization, promotes the immunosuppressive function of M2-type macrophages, and leads to a decrease in tumor immune surveillance and promoting melanoma tumor growth (Lee et al., 2018). In addition, DEHP can promote the expression of immune checkpoint protein PD-L1 in hepatocellular carcinoma (HCC) cells by activating the JAK2/STAT3 signaling pathway, thereby enhancing tumor immune escape (Xu et al., 2021). Jin et al. find in a colon cancer model induced by 1,2-dimethylhydrazine (DMH) that DEHP exposure significantly increases the number of abnormal crypt foci (ACF) and tumor incidence, accompanied by the upregulation of tumor-related proteins such as β-catenin, COX-2, and VEGF, suggesting that DEHP may exacerbate the development of colon tumors (Chen et al., 2017). Collectively, these studies showed that exposure to DEHP is associated with various tumors.

Most importantly, current evidence suggests a potential association between DEHP exposure and glioma-related phenotypes. A zebrafish study reported that DEHP exposure was associated with glioblastoma-related changes accompanied by circadian dysregulation of PER3 (Men et al., 2024). Although current research is still limited and mostly focused on single genes or mechanisms, the 12 characteristic genes identified in this study provide new perspectives and directions for further exploration of the effects of DEHP on glioma. In glioma research, the expression of the CDKL2 gene is significantly lower in glioma tissue than in normal tissue (Yi et al., 2020). Low levels of CDKL2 are closely associated with the type of astrocyte and higher clinical WHO grade. Further analysis revealed that patients with low CDKL2 expression had significantly shorter overall survival than those with high expression, suggesting that CDKL2 could serve as a potential biomarker for assessing the prognosis of glioma patients, consistent with our analysis results. Additionally, Bu et al. utilize the GEO database to investigate the expression levels and diagnostic value of GSTO2, finding that its expression was significantly reduced in glioblastoma samples and exhibited high diagnostic accuracy (Bu et al., 2025). HIF1A expression is also closely associated with the malignant degree of glioma. A study involving immunohistochemical detection of tissue samples from 120 glioma patients found that HIF1A expression was significantly higher in high-grade glioma tissues than in low-grade glioma tissues, and patients with high HIF1A expression had lower overall survival rates, indicating that HIF1A may play a key role in the malignant progression of glioma (Ding et al., 2021).

Taken together with our clinical-feature analysis, these observations suggest that the biological contribution of the 12-gene signature may not be uniform across glioma subtypes. The present model was developed primarily as a glioma-versus-normal classifier rather than as a grade-specific classifier; however, several constituent genes appear to be more strongly linked to higher-grade disease biology. This point should be considered when interpreting the potential translational value of the signature across low-grade and high-grade glioma contexts.

The STK17B gene is significantly more highly expressed in glioma tissue and glioma cell lines (such as LN229 and U251 cell lines) than in normal brain tissue and normal human astrocyte cell lines (Li et al., 2024a). Its overexpression promotes glioma cell proliferation and epithelial-mesenchymal transition processes, thereby driving the malignant progression of glioma. Conversely, knockdown of STK17B significantly inhibits these processes, and high STK17B expression is closely associated with poor prognosis in glioma patients, further suggesting that STK17B may be a potential therapeutic target for glioma treatment. TRAF3IP2 expression in glioma cells is closely associated with tumor cell proliferation and invasive capacity (Bayat et al., 2018). Studies have found that inhibiting TRAF3IP2 expression with atorvastatin significantly reduces the proliferation and invasive capacity of U87 glioma cells and promotes apoptosis, indicating that TRAF3IP2 plays an important role in glioma progression. In studies on glioblastoma recurrence, pathogenic mutations in TYRP1 gene were identified in recurrent tumors, suggesting that TYRP1 may play a crucial role in tumor recurrence (Chanez et al., 2022). This provides new insights for the early diagnosis and treatment of glioblastoma. Additionally, MYC can directly bind to the promoter and super-enhancer regions of TMEM44-AS1, activates its transcription and forms a positive feedback loop, and further promotes glioma progression (Bian et al., 2021). Meanwhile, RELA promotes the transcription of the STC1 gene by binding to the P5 region of the STC1 promoter, thereby driving the malignant progression of glioma (Wang et al., 2023). In the GL261 glioma mouse model, flow cytometry analysis reveals that the mRNA expression levels of ABCA1 in tumor-associated macrophages significantly increase with tumor progression (Wang et al., 2022). Immunohistochemical analysis of brain tissue further confirms that ABCA1 expression is significantly higher in tumor tissue than in normal brain tissue. However, previous studies indicate that its expression is unrelated to prognosis. Our analysis may address this limitation, as we find that its high expression is consistent with the reduced survival in primary glioma. Additionally, antagonists of GABRA5 significantly inhibit the proliferation and invasion of glioblastoma organoid cultures, suggesting that its reduction may be associated with better prognosis in glioma (Shard et al., 2025). However, interestingly, this is inconsistent with our analysis, potentially indicating that the protective effect of GABRA5 is reduced, thereby promoting progression and lowering patient survival rates. Although no literature has reported an association between CCKBR or MCHR2 and the occurrence and development of gliomas, research in this field remains worthy of in-depth exploration.

Our analysis also found that all characteristic genes are unrelated to DEHP, except for MYC. Some studies have pointed out that MYC is involved in the carcinogenic process of DEHP. For example, DEHP promotes thyroid development through the c-MYC signaling pathway regulated by bisphenol A (Zhang et al., 2022a). However, there is no evidence that DEHP participates in glioma by altering MYC. Our analysis results provide researchers with new targets and insights into the potential role of DEHP-associated molecular alterations in glioma. Among the 12 predicted biotargets, our cellular experiments confirmed that the three selected genes, HIF1A, ABCA1, and RELA, were highly expressed in DEHP-exposed U87 cells, further validating the reliability of this prediction model. Further mechanistic studies are required to explore the potential molecular mechanisms and biological pathways through which DEHP influences glioma development. In addition, a dedicated vehicle-only control was not included in the present study, which may limit the exclusion of potential carrier-related effects and should be addressed in future validation experiments. Furthermore, the study results emphasize the necessity of further exploring the molecular mechanisms potentially linking DEHP exposure to glioma-related molecular changes, which may lay the foundation for developing novel therapeutic strategies for gliomas. Importantly, the prognostic model in this study was derived from transcriptomic features and does not represent directly measured DEHP exposure in human subjects. Therefore, the DEHP-related risk score should be interpreted as a molecular signature associated with DEHP-related target genes rather than as a direct surrogate for individual exposure burden. Although the model was evaluated in TCGA and three external survival cohorts (CGGA_325, CGGA_693, and GSE16011), further validation in additional prospective cohorts with matched biomonitoring data will be needed.

## 5 Conclusion

In summary, our comprehensive and in-depth analysis integrating network toxicology, machine learning, molecular docking, and molecular dynamics simulations identified potential associations between DEHP-related molecular signatures and glioma pathogenesis. By integrating multi-source data, we identified 12 DEHP-associated target genes that may be involved in glioma-related processes, and further validated three key genes, namely RELA, ABCA1, and HIF1A. The application of machine learning algorithms has further deepened our understanding of these genes. Combined with clinical and prognostic analyses, this study reveals the potential of these core target genes as diagnostic and prognostic biomarkers under DEHP exposure. Notably, their diagnostic performance highlights clinical application potential, suggesting that these key genes might serve as important tools for the early detection and management of glioma. Overall, this study adds new evidence to the knowledge regarding the association between environmental factors and cancer, underscoring the significance of exposure factors in public health intervention measures.

## Data Availability

The original contributions presented in this study are publicly available. The data can be accessed from the Gene Expression Omnibus (GEO) under accession numbers GSE16011, GSE4290, GSE10878, and GSE50161, as well as from The Cancer Genome Atlas (TCGA) and the Chinese Glioma Genome Atlas (CGGA; datasets CGGA_325 and CGGA_693). Details and access links are provided in the article/Supplementary Material.

## Supplementary material

SUPPLEMENTARY FIGURE S1 A simplified workflow diagram illustrates the process of screening targets through which DEHP affects glioma, utilizing machine learning to identify core targets, and ultimately integrating experimental validation into the analytical workflow.

SUPPLEMENTARY FIGURE S2 Prognostic analysis of the 12 core hub genes identified by machine learning in glioma survival cohorts. In the TCGA survival cohort, except for TYRP1, all genes exhibited significant differences in overall survival between high and low expression groups. In the CGGA_325 survival cohort, apart from MYC, TRAF3IP2, and TYRP1, the remaining genes showed survival differences between high and low expression groups.

SUPPLEMENTARY FIGURE S3 Prognostic analysis of the 12 core hub genes identified by machine learning in glioma survival cohorts. In the CGGA_693 survival cohort, except for RELA, STK17B, and TRAF3IP2, significant survival differences in overall survival were observed between the high and low expression groups of the remaining genes. In the GSE16011 survival cohort, which primarily consists of glioblastomas, only ABAC1, GSTO2, HIF1A, MYC, RELA, and TYRP1 exhibited survival differences between the high and low expression groups.

SUPPLEMENTARY FIGURE S4 Molecular Dynamics Simulation of RELA and DEHP. **(A)** RMSD of the complex, RELA, and the small-molecule ligand DEHP. **(B)** Rg of the complex. **(C)** RMSF of RELA within the complex. **(D)** Distance between the binding sites of RELA and DEHP (Dock site-ligand). **(E)** Buried solvent-accessible surface area (Buried SASA) between RELA and DEHP. **(F)** Superposition of simulated conformations. **(G)** Binding energies (VDW and ELE) between RELA and DEHP. **(H)** Contribution of amino acids to the binding energy. **(I)** Number of hydrogen bonds (Hbond number). **(J)** Interaction between RELA and DEHP.

SUPPLEMENTARY FIGURE S5 Molecular Dynamics Simulation of RELA and DEHP. **(A)** RMSD of the complex, ABAC1, and the small-molecule ligand DEHP. **(B)** Rg of the complex. **(C)** RMSF of ABAC1 within the complex. **(D)** Distance between the binding sites of ABAC1 and DEHP (Dock site-ligand). **(E)** Buried solvent-accessible surface area (Buried SASA) between ABAC1 and DEHP. **(F)** Superposition of simulated conformations. **(G)** Binding energies (VDW and ELE) between ABAC1 and DEHP. **(H)** Contribution of amino acids to the binding energy. **(I)** Number of hydrogen bonds (Hbond number). **(J)** Interaction between ABAC1 and DEHP.

SUPPLEMENTARY FIGURE S6 Molecular Dynamics Simulation of HIF1A and DEHP. **(A)** RMSD of the complex, HIF1A, and the small-molecule ligand DEHP. **(B)** Rg of the complex. **(C)** RMSF of HIF1A within the complex. **(D)** Distance between the binding sites of HIF1A and DEHP (Dock site-ligand). **(E)** Buried solvent-accessible surface area (Buried SASA) between HIF1A and DEHP. **(F)** Superposition of simulated conformations. **(G)** Binding energies (VDW and ELE) between HIF1A and DEHP. **(H)** Contribution of amino acids to the binding energy. **(I)** Number of hydrogen bonds (Hbond number). **(J)** Interaction between HIF1A and DEHP.

SUPPLEMENTARY FIGURE S7 Original image from the WB experiment.

SUPPLEMENTARY FIGURE S8 Precision–recall curves of the top-performing machine-learning workflows in pooled external validation cohorts. Precision–recall curves were generated using pooled external validation cohorts (TCGA>Ex + GSE10878 + GSE50161). Average precision (AP, PR-AUC) is shown in the legend for each workflow. This analysis complements ROC/AUC benchmarking by providing a class-imbalance-sensitive evaluation of positive-class discrimination.

SUPPLEMENTARY FIGURE S9 Cohort-specific precision–recall curves of the selected glmBoost + Ridge model in external validation datasets. Precision–recall curves are shown separately for each independent validation cohort to illustrate the robustness of positive-class discrimination for the selected 12-gene glmBoost + Ridge model beyond ROC/AUC alone. Average precision (AP, PR-AUC) is shown in the legend.
