# Supplementary figures and images for: Machine learning-integrated network toxicology uncovers glioma targets of DEHP
Source: Front Toxicol. 2026 May 8;8:1771011. doi: 10.3389/ftox.2026.1771011 (PMC13193683; doi:10.3389/ftox.2026.1771011)

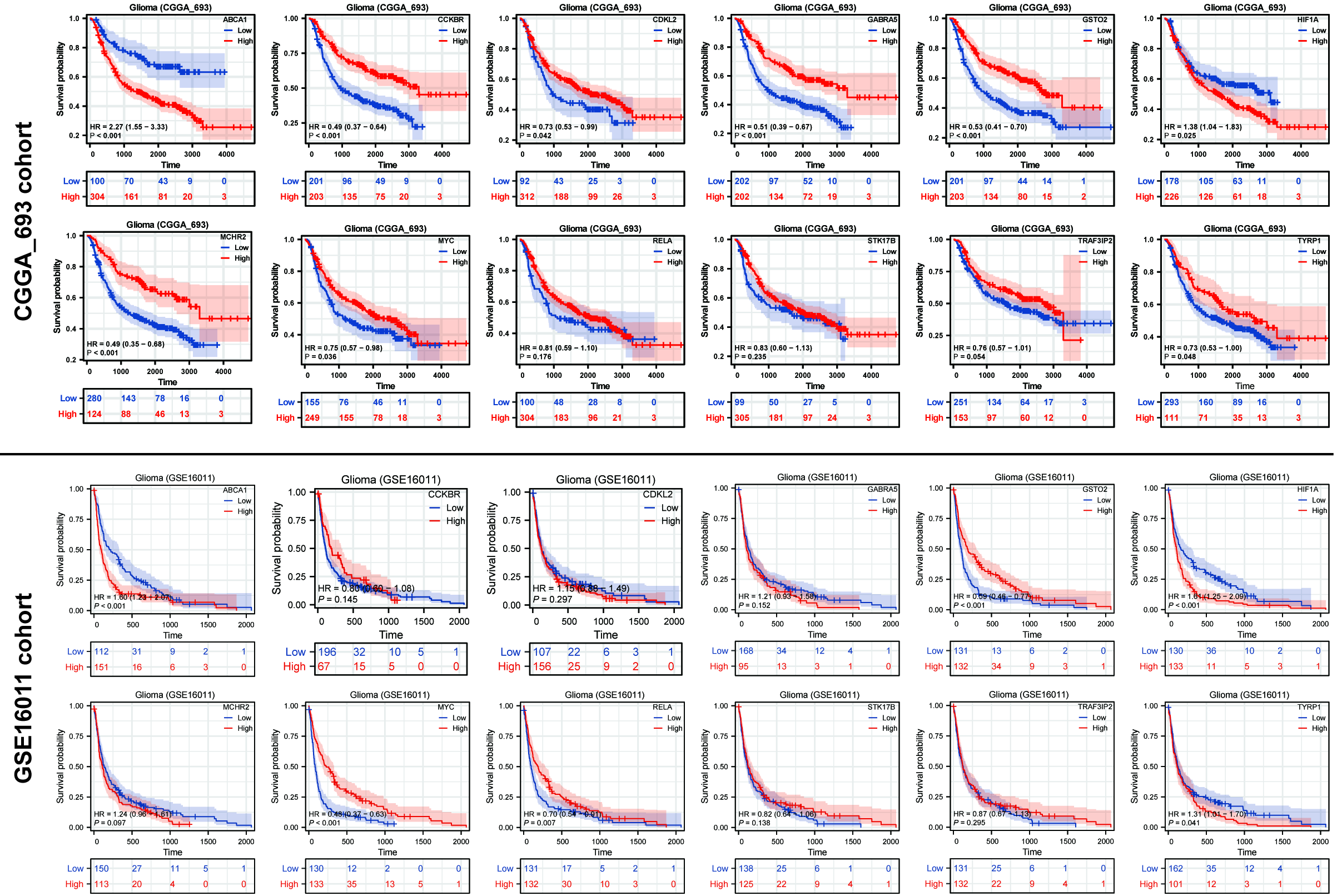

Supplement: Supplementary file 1 [file Image3.TIF]

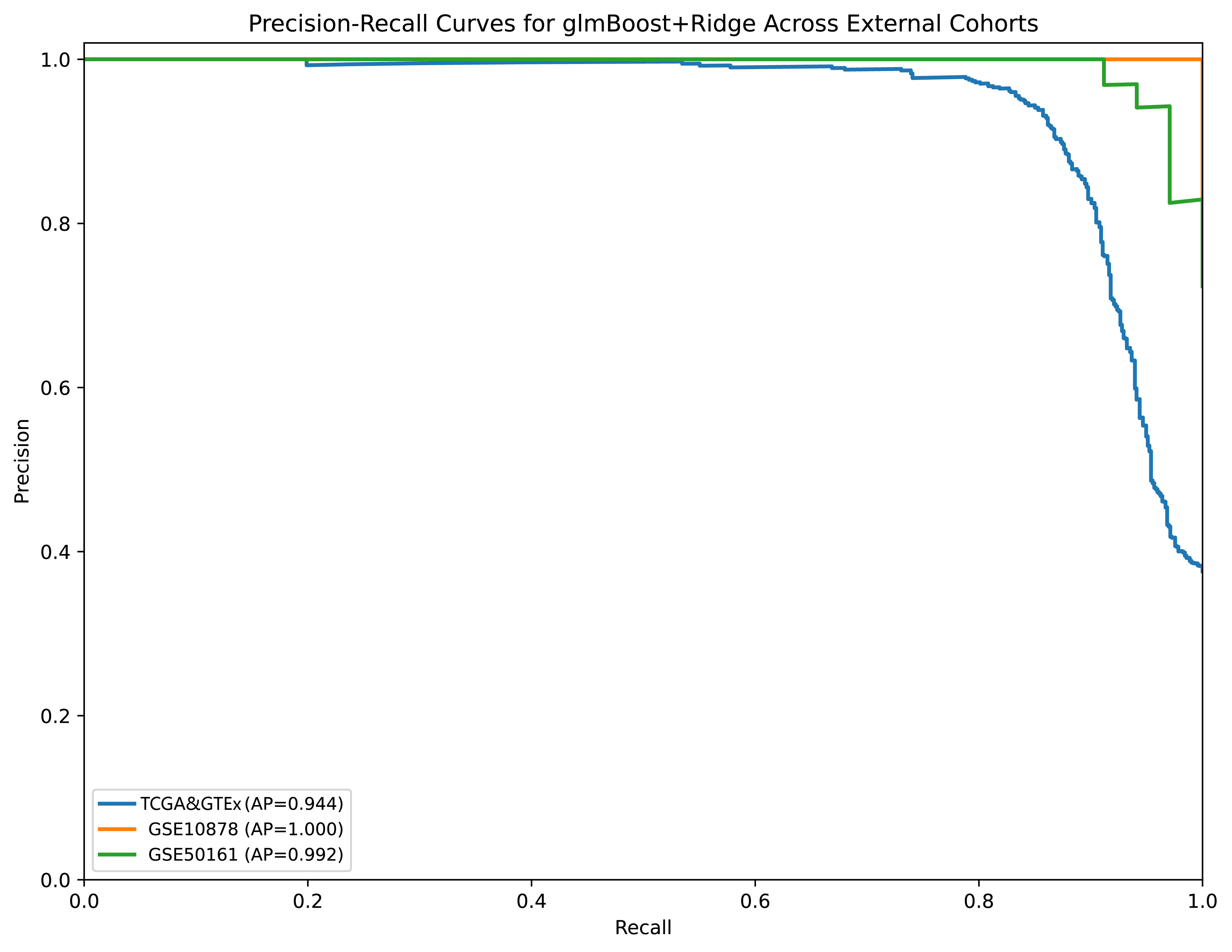

Supplement: Supplementary file 2 [file Image9.TIF]

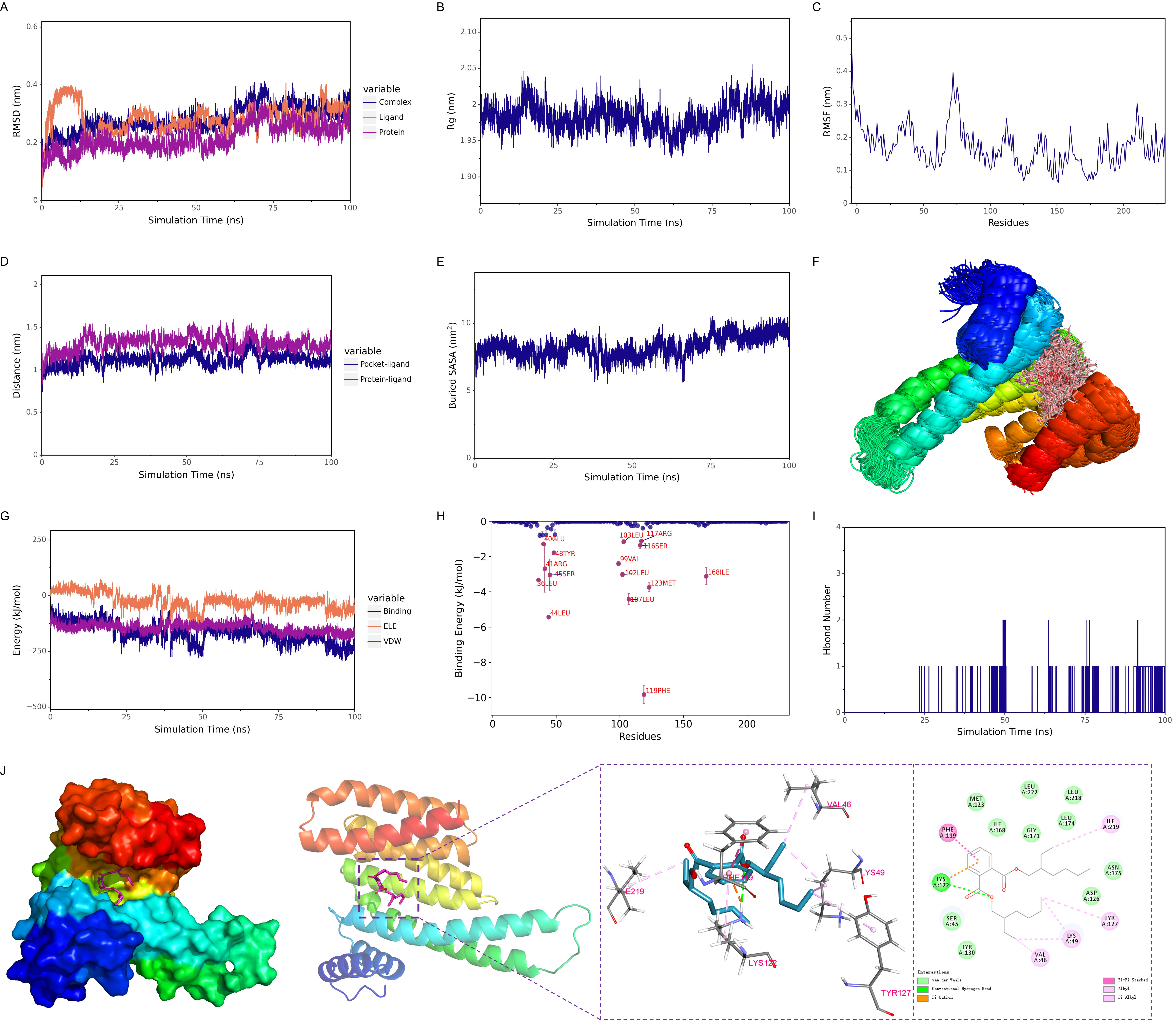

Supplement: Supplementary file 3 [file Image4.JPEG]

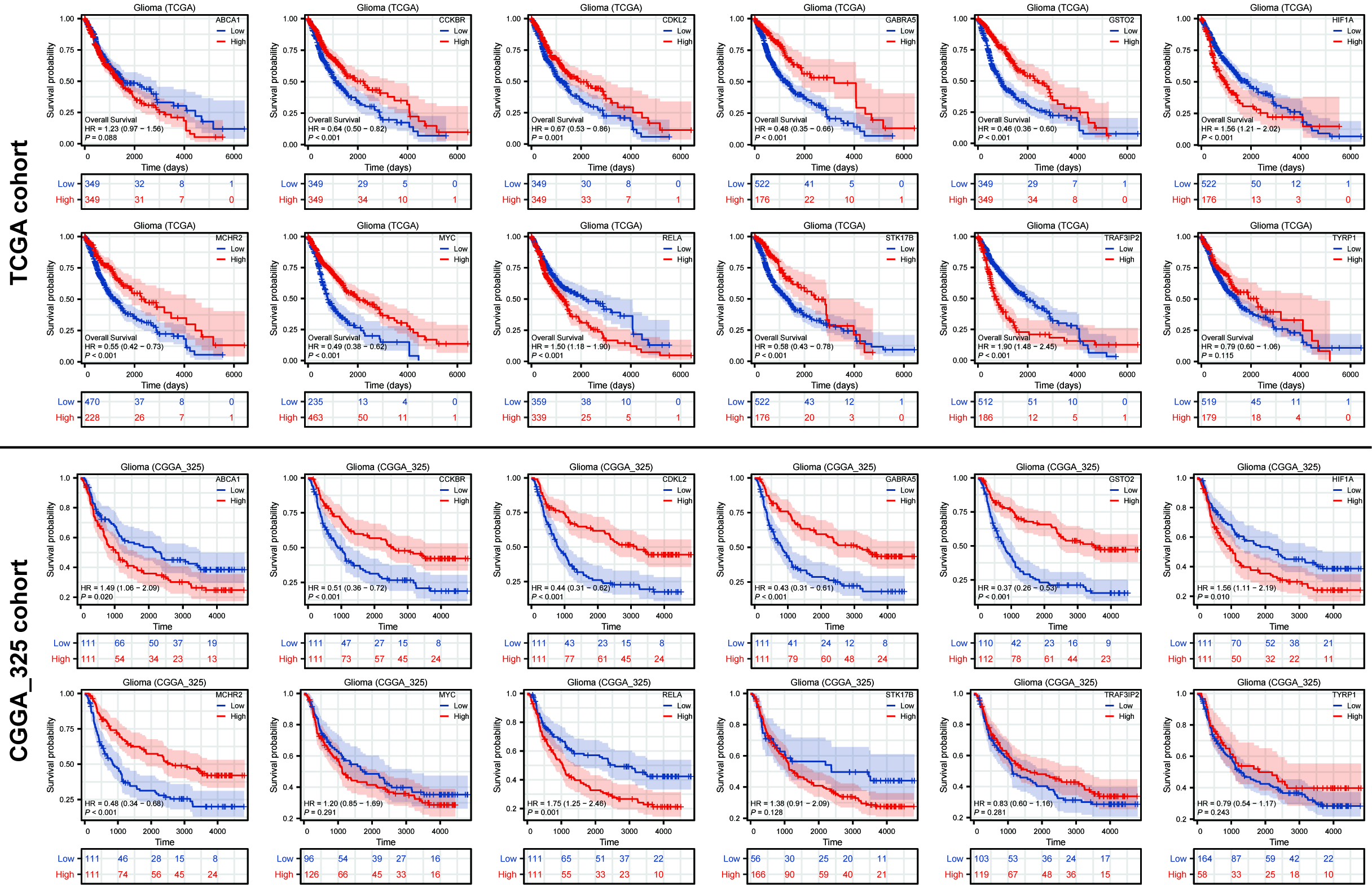

Supplement: Supplementary file 4 [file Image2.TIF]

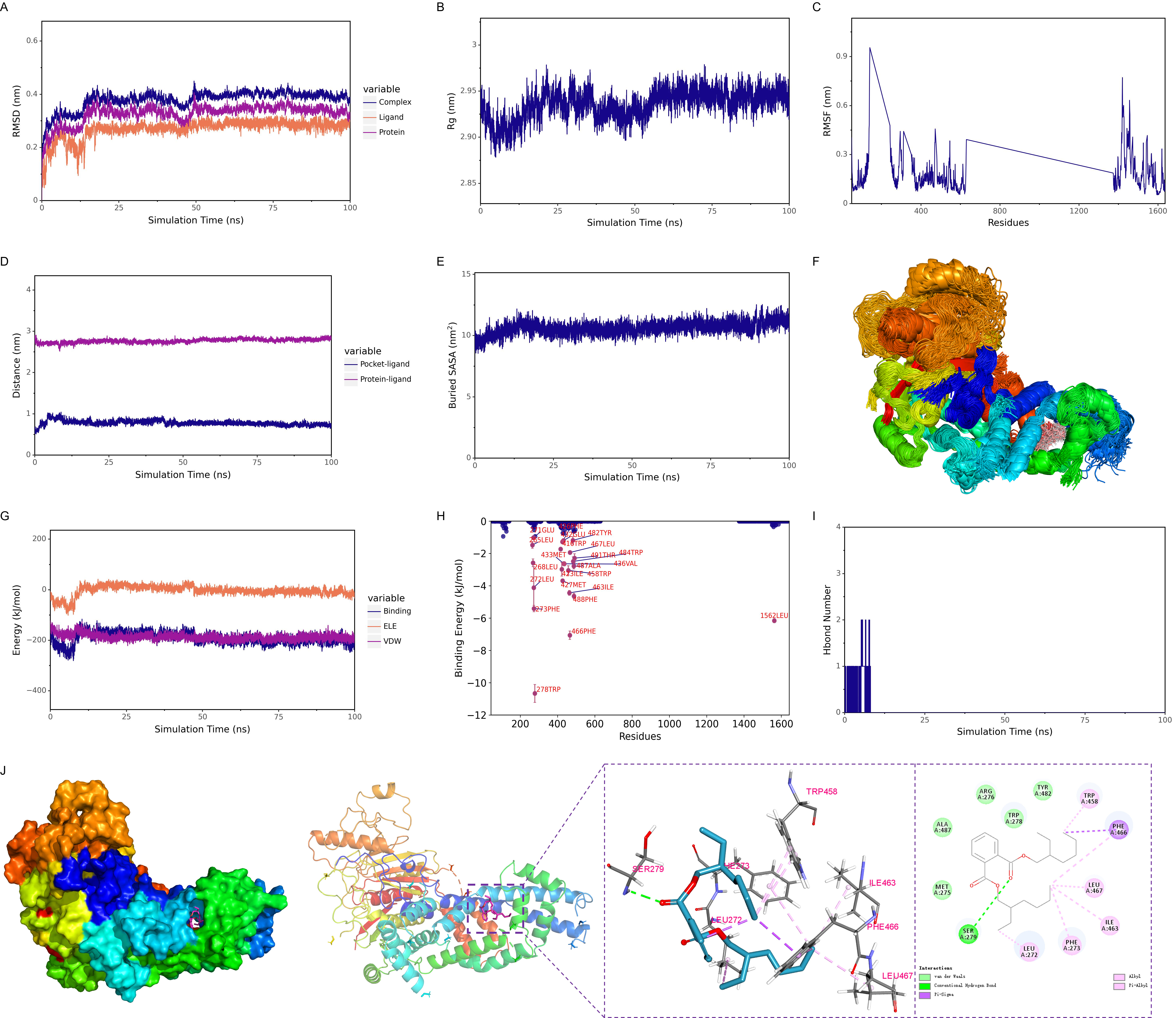

Supplement: Supplementary file 5 [file Image5.JPEG]

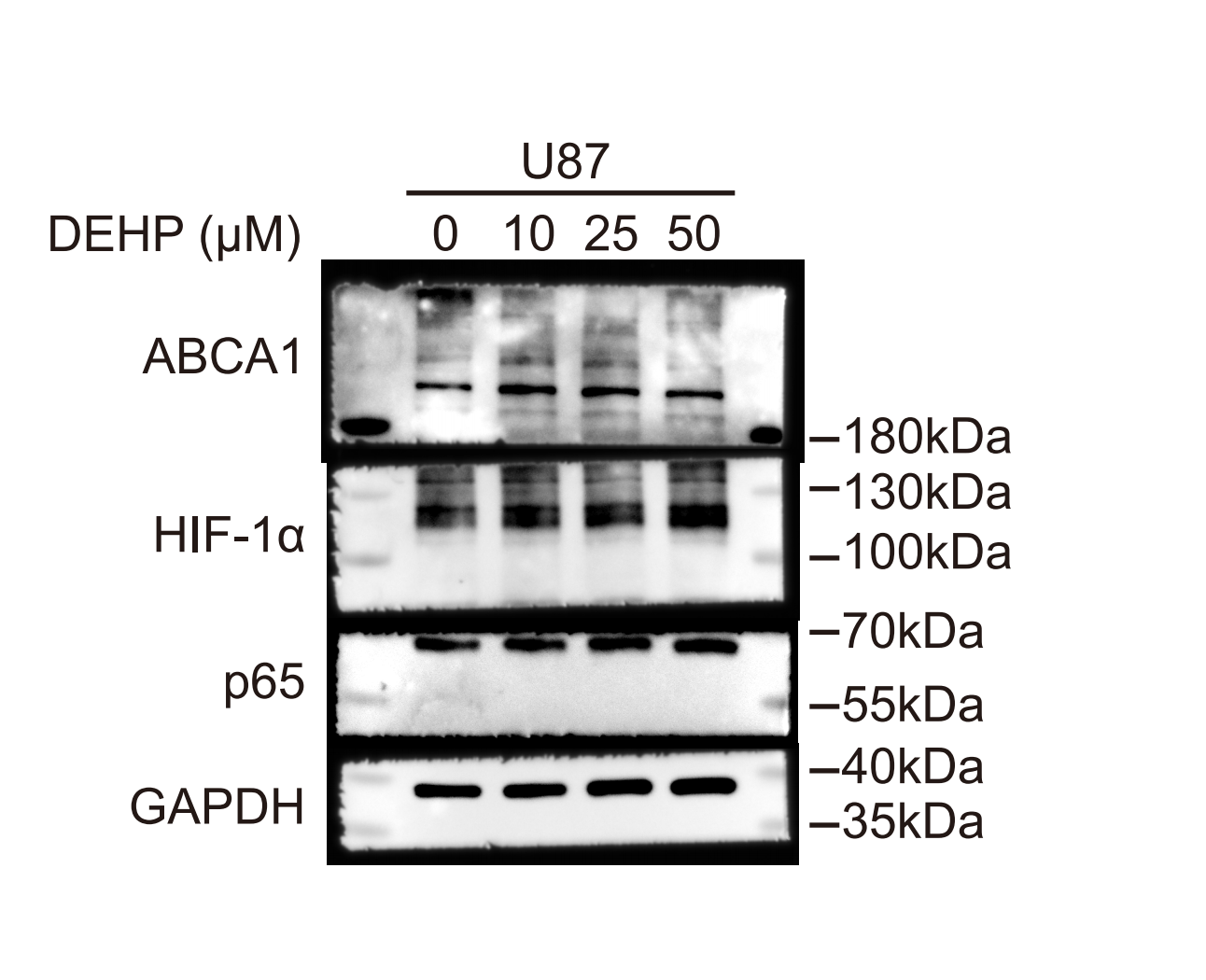

Supplement: Supplementary file 6 [file Image7.TIF]

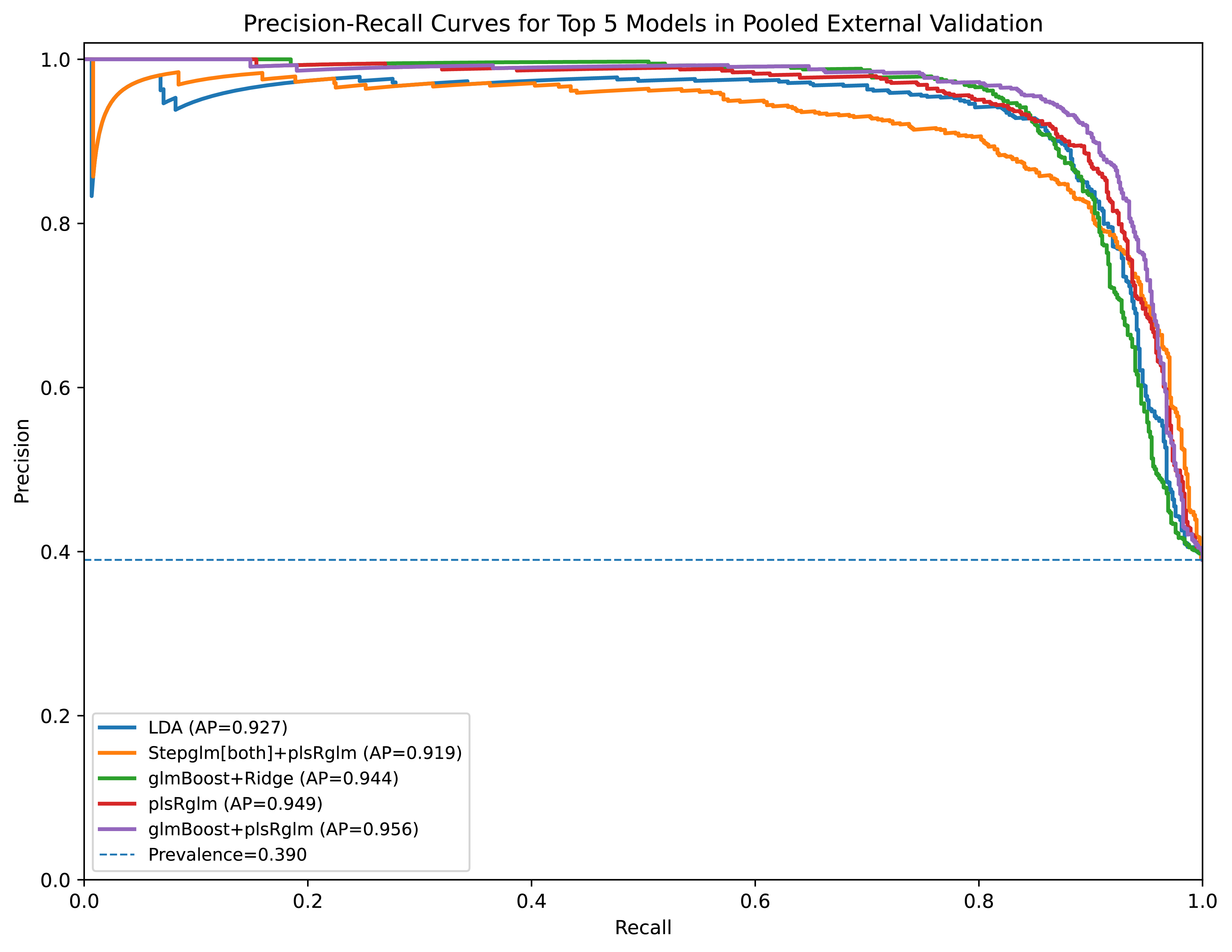

Supplement: Supplementary file 8 [file Image8.TIF]

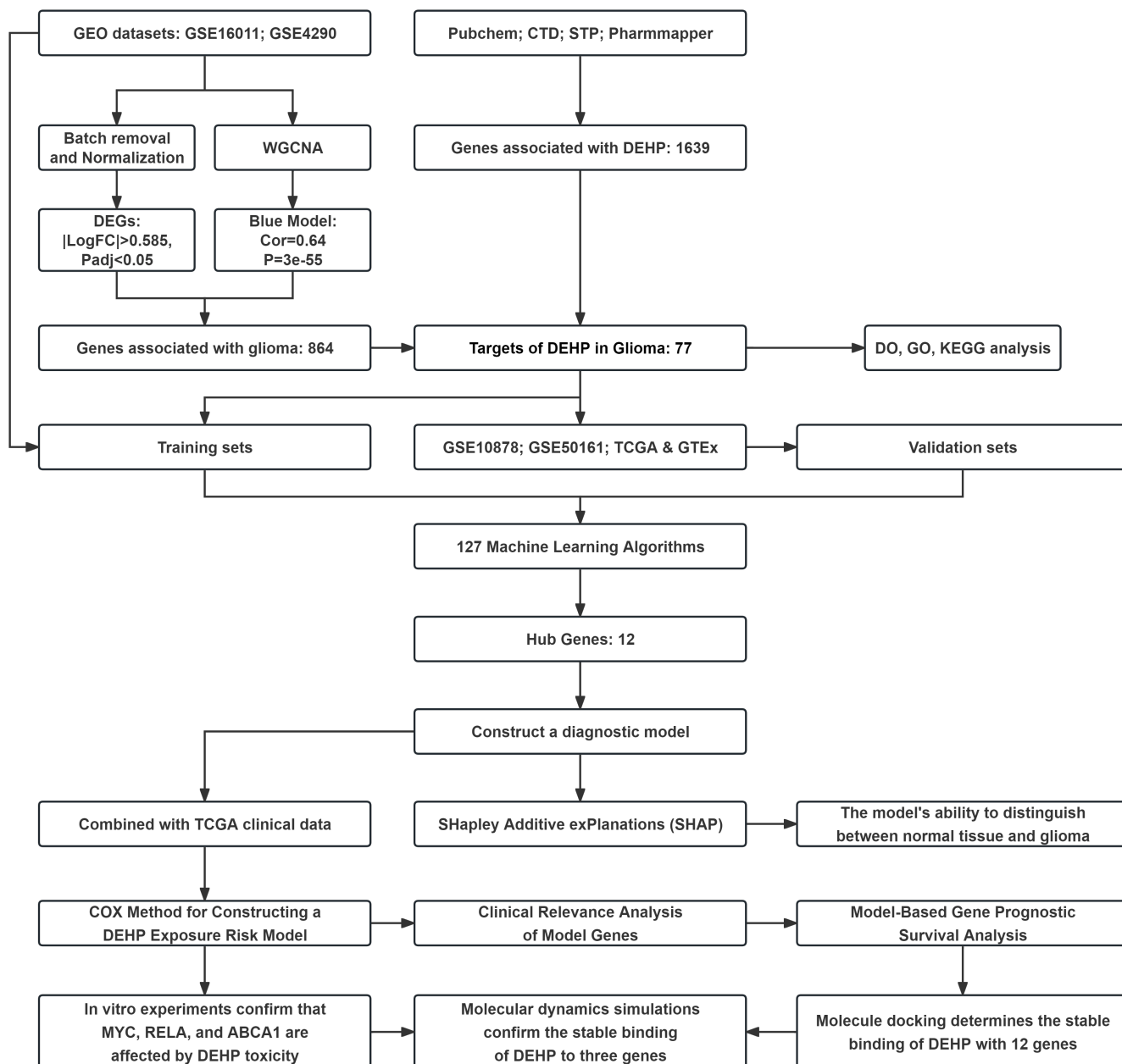

Supplement: Supplementary file 10 [file Image1.pdf]

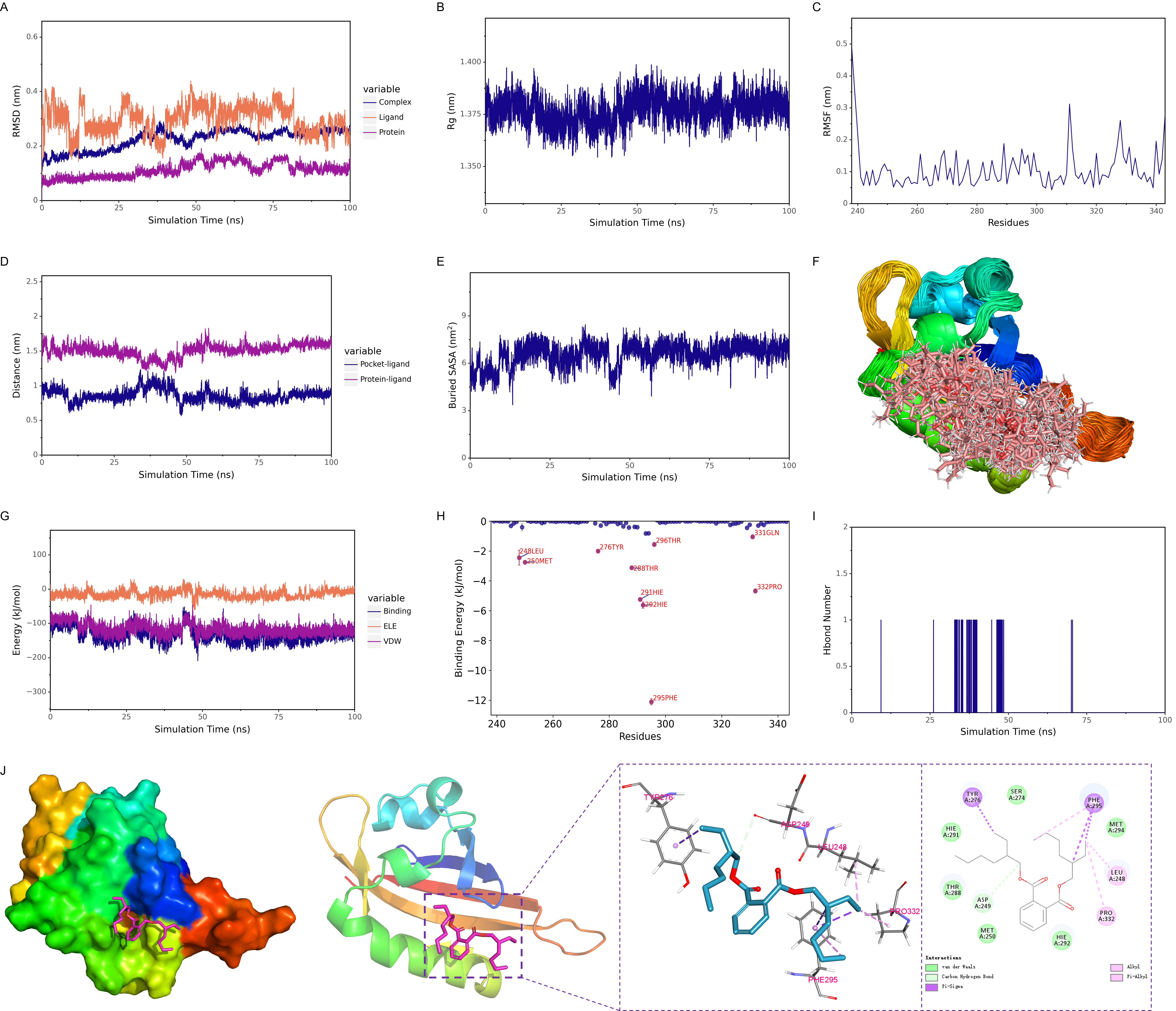

Supplement: Supplementary file 11 [file Image6.JPEG]
