# Supplementary material for: Machine learning-integrated network toxicology uncovers glioma targets of DEHP
Source: Front Toxicol. 2026 May 8;8:1771011. doi: 10.3389/ftox.2026.1771011 (PMC13193683; doi:10.3389/ftox.2026.1771011)
Supplement: Supplementary file 7 [file DataSheet2.docx]

**Table S6. WGCNA ModuleGenes_blue**

| **Blue** | **BLACK** | **Brown** | **Green** | **Grey** | **Magenta** | **Pink** | **Turquoise** |
| --- | --- | --- | --- | --- | --- | --- | --- |
| AACS | AATK | ABLIM1 | A2M | A1BG | APOL6 | ACE | AASS |
| ABCA1 | ABCA2 | ABLIM3 | AADAT | AAAS | BATF2 | ACSS1 | ABCA5 |
| ABCB4 | ABCA8 | ABTB1 | ABAT | AASDH | BST2 | ACTA2 | ABCC1 |
| ABCC4 | ACTN2 | ACKR1 | ABCA13 | ABCA11P | BTN3A2 | ACTG2 | ABCC5 |
| ABHD8 | ADAP1 | ACSM5 | ABCA3 | ABCA12 | BTN3A3 | ADAM12 | ABCC8 |
| ABL1 | ADARB2 | ACY3 | ABCC3 | ABCA6 | CXCL10 | AFAP1L1 | ABHD3 |
| ABR | ADIRF | ADCY2 | ABHD14B | ABCB1 | CXCL11 | ANGPT2 | ABHD6 |
| ACOT4 | AGPAT4 | ADCY8 | ABHD15 | ABCC6P1 | CXCL9 | ANO1 | ABI1 |
| ACOT7 | AGPAT4-IT1 | ADD3 | ABRACL | ABCD2 | DDX58 | ANPEP | ACACA |
| ACTL6A | AGTPBP1 | ADIPOR2 | ACAA2 | ABCG1 | DDX60 | AOC3 | ACADSB |
| ACTR3B | ALDH1A1 | AGO2 | ACAT2 | ABCG2 | DDX60L | ARHGAP29 | ACBD5 |
| ACTR3C | ALDH8A1 | AHCYL1 | ACCS | ABHD12 | DHX58 | ASPN | ACKR3 |
| ADAM11 | ANKRD18A | AHCYL2 | ACP5 | ABHD17C | EPSTI1 | BGN | ACO2 |
| ADAM23 | ANKS1B | AIG1 | ACPP | ABHD2 | GBP5 | C7 | ACOT9 |
| ADAMTS6 | ANLN | AKNAD1 | ACSL1 | ABI3BP | HCP5 | CALD1 | ACOX2 |
| ADAMTS8 | ANO4 | AKR1C1 | ACSL5 | ACADL | HERC5 | CD248 | ACP6 |
| ADCY1 | APLP1 | AKR1C3 | ACTN1 | ACAN | HERC6 | CD34 | ACSL6 |
| ADCYAP1 | APOD | AKTIP | ACVR2B | ACER3 | HLA-A | CD36 | ACSS3 |
| ADRA1B | ARHGAP22 | ALDH1L1 | ADAM28 | ACHE | HLA-B | CD93 | ACTL6B |
| ADRA2A | ARRB1 | ALDH2 | ADAMDEC1 | ACOT12 | HLA-C | CDC42EP5 | ACTN4 |
| ADRB1 | ARRDC2 | ALG6 | ADAMTS1 | ACSBG1 | HLA-E | CDH5 | ACVR1C |
| AGPAT5 | ASPA | ALYREF | ADAMTS19 | ACSF2 | HLA-F | CLEC11A | ACVR2A |
| AIFM3 | ATP10B | AMOT | ADAP2 | ACSL3 | HLA-G | CLEC14A | ADAM9 |
| AK5 | BCAS1 | AMT | ADM | ACTC1 | IFI27 | CNN2 | ADAMTS9 |
| AKAP5 | BEST1 | ANKLE1 | ADORA3 | ACTL9 | IFI35 | COL12A1 | ADD2 |
| ALDH18A1 | BIN1 | ANKS1A | ADRB2 | ACTRT3 | IFI44 | COL13A1 | ADHFE1 |
| ALKBH6 | BOK | AP4M1 | ADSSL1 | ACVRL1 | IFI44L | COL15A1 | AEBP1 |
| ANK2 | CAPN3 | APCDD1 | AGA | ACY1 | IFI6 | COL18A1 | AEN |
| ANKRD10 | CARNS1 | APOBEC3B | AHR | ACYP1 | IFIH1 | COL1A1 | AFAP1L2 |
| ANKRD2 | CCP110 | APOL4 | AIF1 | ADA | IFIT1 | COL1A2 | AFF3 |
| ANKRD29 | CD22 | AQP4-AS1 | AKAP12 | ADAD2 | IFIT3 | COL3A1 | AGAP1 |
| ANKRD34A | CDC42EP2 | ARHGAP11A | AKAP7 | ADAL | INSM2 | COL4A1 | AGAP2 |
| ANKRD9 | CDH19 | ARHGAP26 | AKNA | ADAM10 | IRF1 | COL4A2 | AGTRAP |
| ANO3 | CDK18 | ARHGAP36 | ALG14 | ADAM2 | IRF7 | COL5A1 | AJAP1 |
| ANTXR1 | CDK19 | ARHGAP5 | ALOX5 | ADAM32 | IRF9 | COL5A2 | AJUBA |
| ANXA11 | CDKN1C | ARHGEF37 | ALOX5AP | ADAM3A | ISG15 | COL6A1 | AK2 |
| ANXA3 | CERCAM | ARL2 | ALPK1 | ADAM6 | LAP3 | COL6A2 | AKAP11 |
| APOO | CHN2 | ASF1B | ALPK2 | ADAM8 | LGALS3BP | COL6A3 | AKAP13 |
| APRT | CLCA4 | ASPM | AMIGO2 | ADAMTS15 | MX1 | CRIP1 | AKAP6 |
| ARF3 | CLDN11 | ASTN2 | AMPD3 | ADAMTS18 | MX2 | CTHRC1 | AKT3 |
| ARHGAP19 | CLDND1 | ATAD2 | ANGPT1 | ADAMTS3 | NMI | CTNND2 | ALCAM |
| ARHGAP20 | CLMN | ATP1A2 | ANGPTL4 | ADAMTS5 | OAS1 | CTSK | ALDH16A1 |
| ARHGAP44 | CNDP1 | ATP1B2 | ANKRD22 | ADAMTSL2 | OAS2 | CYP1B1 | ALDH5A1 |
| ARHGDIG | CNP | ATP6V0A2 | ANXA2P3 | ADCK5 | OAS3 | CYYR1 | ALDH6A1 |
| ARHGEF40 | CNTN2 | AURKA | ANXA4 | ADCY10P1 | PARP12 | DCN | ALDOC |
| ARHGEF6 | CNTNAP4 | AURKB | AOAH | ADCY4 | PARP14 | DUSP5 | ALOX12B |
| ARL6IP6 | COBL | BBS2 | AP1S2 | ADCYAP1R1 | PARP9 | DUSP6 | ALPK3 |
| ART3 | COL9A2 | BCO2 | AP2B1 | ADGB | PLSCR1 | ECM1 | AMER2 |
| ASB2 | CPOX | BCOR | APBB1IP | ADH1B | PSMB8 | EGFL6 | AMER3 |
| ASCC2 | CSRP1 | BIRC5 | APBB2 | ADH1C | PSMB9 | EHD2 | AMN1 |
| ASIC2 | CYB5R2 | BLM | APC2 | ADI1 | RSAD2 | EHD4 | AMOTL2 |
| ASPHD2 | CYP2J2 | BMPR1B | APLN | ADM5 | RTP4 | EMCN | AMPH |
| ATF1 | DHRS9 | BORA | APLNR | ADORA1 | SAMD9 | ENG | ANG |
| ATOH7 | DIP2B | BRAT1 | APOBEC3C | ADORA2B | SAMD9L | ENPEP | ANGPTL2 |
| ATP13A2 | DLG1 | BRCA1 | APOBEC3G | ADRA1D | SP110 | EOGT | ANK1 |
| ATP1B1 | DNAH17 | BRCA2 | APOBR | AFAP1 | STAT1 | EPS8 | ANK3 |
| ATP2B1 | DNAJB2 | BRD2 | APOC1 | AFG3L1P | TAP1 | ESAM | ANKH |
| ATP2B3 | DNAJC6 | BRIP1 | APOC2 | AFP | TRIM22 | ESYT2 | ANKRD13B |
| ATP6V1A | DOCK10 | BTG2 | APOE | AGBL2 | TRIM5 | FAT3 | ANKRD26 |
| ATP6V1B2 | DOCK5 | BTG3 | APOL1 | AGL | XAF1 | FBLIM1 | ANKRD28 |
| ATP6V1E1 | EDIL3 | BUB1 | APOL3 | AGO3 |  | FBLN1 | ANKRD46 |
| ATP7A | ELOVL7 | BUB1B | AQP1 | AGR3 |  | FBN1 | ANKRD55 |
| ATP8A2 | ENPP2 | BYSL | AQP9 | AGRP |  | FCN3 | ANKRD6 |
| ATPAF1 | ENPP6 | BZW2 | ARHGAP18 | AGT |  | FKBP11 | ANO6 |
| AVPI1 | EPB41L3 | C1QTNF2 | ARHGAP25 | AHNAK |  | FMN2 | ANTXR2 |
| BAIAP2 | EPCAM | CAB39L | ARHGAP30 | AHNAK2 |  | FN1 | ANXA1 |
| BAIAP3 | ERBB3 | CADM3 | ARHGAP4 | AIF1L |  | FNDC1 | ANXA2 |
| BARD1 | ERMN | CARHSP1 | ARHGAP6 | AIM2 |  | FOXS1 | ANXA2P1 |
| BAZ1A | ERMP1 | CASP3 | ARHGAP9 | AK1 |  | FRZB | ANXA2R |
| BBS7 | EVI2A | CBX5 | ARHGDIB | AKAP14 |  | FSTL1 | ANXA5 |
| BCL11A | FA2H | CBX7 | ARID5A | AKAP4 |  | GGT5 | AP1G2 |
| BCL2L2 | FAAH | CCDC15 | ARL11 | AKIRIN1 |  | GJA4 | AP2S1 |
| BDH1 | FAM107B | CCDC159 | ARL4C | AKR1B10 |  | GNG11 | AP3B2 |
| BEGAIN | FAM124A | CCDC167 | ARPC1B | AKR1C4 |  | GPM6B | AP3M2 |
| BEND6 | FBXO2 | CCDC77 | ARPC5 | AKT1 |  | GPX8 | APBA1 |
| BEX5 | FCHO1 | CCL4 | ARRDC3 | ALDH1A2 |  | HMCN1 | APBA2 |
| BHLHB9 | FEZ1 | CCNA2 | ASF1A | ALDH1A3 |  | HSPG2 | APBB1 |
| BHLHE22 | FGF1 | CCNB1 | ASGR1 | ALDH3A1 |  | IGFBP4 | APC |
| BRSK1 | FGFR2 | CCNB2 | ATAT1 | ALDH4A1 |  | ISLR | APH1A |
| BTBD10 | FMNL2 | CCND2 | ATF3 | ALDH7A1 |  | ITGA4 | AQP11 |
| BTBD3 | FNBP1 | CCNE1 | ATP6V0E2 | ALDOA |  | ITGA5 | AR |
| BTBD6 | FOLH1 | CCNE2 | ATP8B4 | ALG12 |  | ITGBL1 | ARAP3 |
| BTG1 | FOLH1B | CCNJL | AXL | ALK |  | ITPR3 | ARHGAP12 |
| CA11 | FRMD4B | CCNY | B4GALNT4 | ALKBH8 |  | JAG1 | ARHGAP31 |
| CA4 | FUT8 | CCPG1 | BAG3 | ALLC |  | JAKMIP2 | ARHGAP32 |
| CABLES1 | GAL3ST1 | CCT2 | BATF | ALOX12 |  | KCNE3 | ARHGAP42 |
| CABP1 | GALNT6 | CCT6A | BATF3 | ALOX15 |  | KDELR3 | ARHGEF17 |
| CABYR | GJB1 | CDC20 | BCL2A1 | ALX3 |  | KDR | ARHGEF7 |
| CACNA1B | GJC2 | CDC25A | BCR | ALX4 |  | KIF1B | ARHGEF9 |
| CACNB2 | GLDN | CDC25B | BDH2 | AMD1 |  | LAMA4 | ARL13B |
| CACNB3 | GNG7 | CDC25C | BDKRB2 | AMDHD1 |  | LAMB1 | ARL3 |
| CACNB4 | GPIHBP1 | CDC45 | BHLHE40 | AMELY |  | LAMC1 | ARMCX5 |
| CACNG3 | GPR37 | CDC6 | BHLHE41 | AMER1 |  | LAMC3 | ARNT2 |
| CADPS2 | GPR62 | CDC7 | BIN2 | AMFR |  | LBH | ARNTL2 |
| CALB1 | GPRC5B | CDCA2 | BIRC3 | ANGPT4 |  | LMNA | ARPP19 |
| CALB2 | GREM1 | CDCA3 | BLNK | ANGPTL1 |  | LOXL2 | ARPP21 |
| CALM1 | GRM3 | CDCA4 | BLVRB | ANGPTL6 |  | LRP1B | ARSD |
| CALY | HAPLN2 | CDCA5 | BMP2K | ANGPTL7 |  | LRRC32 | ARSJ |
| CAMK1 | HDAC11 | CDCA7 | BNC2 | ANKDD1A |  | LUM | ASAH2B |
| CAMK1D | HHATL | CDCA7L | BRD3 | ANKFN1 |  | LXN | ASAP3 |
| CAMK1G | HHIP | CDCA8 | BRI3 | ANKHD1 |  | MCAM | ASB13 |
| CAMK2A | HOXD1 | CDK1 | BST1 | ANKMY2 |  | MECOM | ASCC1 |
| CAMK2B | HSPA2 | CDK2 | BTBD17 | ANKRD1 |  | METRNL | ASCL1 |
| CAMK2N1 | IP6K3 | CDK4 | BTK | ANKRD18B |  | MFAP2 | ASH1L |
| CAMKK1 | IPO13 | CDK6 | C1QA | ANKRD23 |  | MGP | ASIC1 |
| CAMKK2 | JAKMIP3 | CDKN2C | C1QB | ANKRD30A |  | MMP9 | ASIC4 |
| CAMKV | KANK4 | CDKN3 | C1QC | ANKRD35 |  | MMRN1 | ASL |
| CAMTA2 | KBTBD11 | CDT1 | C1R | ANKRD37 |  | MXRA5 | ASPDH |
| CAP2 | KCNJ2 | CENPA | C1RL | ANKRD45 |  | MYH9 | ASPHD1 |
| CARTPT | KCNMB4 | CENPE | C1S | ANKRD65 |  | MYL9 | ASRGL1 |
| CASK | KIAA0930 | CENPF | C2 | ANKRD7 |  | MYO1B | ASTE1 |
| CBFB | KIAA1324L | CENPH | C2CD2 | ANKS3 |  | NDUFA4L2 | ASTN1 |
| CBLB | KIF13B | CENPI | C3 | ANKS4B |  | NID1 | ASXL3 |
| CBLN4 | KLHL2 | CENPJ | C3AR1 | ANKZF1 |  | NID2 | ATAD1 |
| CCDC110 | KLHL32 | CENPK | C5AR1 | ANO10 |  | NOTCH3 | ATF5 |
| CCDC177 | KLK6 | CENPL | CA3 | ANO8 |  | NOX4 | ATL1 |
| CCDC184 | LACC1 | CENPM | CA9 | ANP32A |  | NR2F2 | ATL3 |
| CCDC28A | LANCL1 | CENPN | CACNG4 | ANP32D |  | NRP1 | ATOH8 |
| CCDC3 | LARP6 | CENPQ | CAPG | ANXA13 |  | OLFML1 | ATP10D |
| CCDC50 | LDB3 | CENPU | CAPZA2 | ANXA6 |  | OLFML2A | ATP1A1 |
| CCDC68 | LDLRAP1 | CENPW | CARD16 | AOX1 |  | PCDH12 | ATP2B2 |
| CCDC92 | LGI3 | CEP135 | CARD6 | AP1S1 |  | PCOLCE | ATP6V0A1 |
| CCK | LHPP | CEP152 | CASKIN2 | APCDD1L |  | PDGFD | ATP6V1G2 |
| CCKBR | LPAR1 | CEP192 | CASP1 | APOA4 |  | PDGFRB | ATP8A1 |
| CCNA1 | LRP2 | CEP55 | CASP4 | APOF |  | PDLIM1 | ATP9A |
| CCNDBP1 | LRRC63 | CEP76 | CASP5 | APOLD1 |  | PDLIM7 | ATRNL1 |
| CDC42 | MAG | CHAC2 | CASQ1 | APP |  | PEA15 | AVEN |
| CDH12 | MAL | CHAF1A | CAV1 | APPL2 |  | PECAM1 | B3GALT2 |
| CDH8 | MAN2A2 | CHAF1B | CAV2 | AQP4 |  | PELO | B3GAT1 |
| CDK14 | MAP6D1 | CHEK1 | CBLN1 | AQP8 |  | PHGDH | B3GNT5 |
| CDK5 | MAP7 | CHEK2 | CBR1 | ARC |  | PHLDA2 | B3GNT9 |
| CDKL2 | MAP7D1 | CHRNA5 | CBS | ARF1 |  | PHLDB2 | B4GALT4 |
| CDKN2D | MAPRE2 | CHST15 | CCDC120 | ARG1 |  | PLVAP | B4GALT5 |
| CDS1 | MBP | CIPC | CCDC69 | ARG2 |  | PLXDC1 | B4GALT6 |
| CELSR2 | MEGF10 | CKAP2 | CCL2 | ARHGAP21 |  | PLXNB1 | B9D2 |
| CEP170B | MID1IP1 | CKAP2L | CCL20 | ARHGAP24 |  | PODXL | BACE2 |
| CEP95 | MITF | CKS2 | CCL5 | ARHGEF10 |  | POSTN | BASP1 |
| CES4A | MOBP | CLIP4 | CCL8 | ARHGEF10L |  | PROCR | BAX |
| CHAC1 | MOG | CNTN3 | CCNB1IP1 | ARHGEF19 |  | PXDNL | BCAN |
| CHCHD10 | MTURN | COLCA2 | CCNYL1 | ARHGEF25 |  | RBPMS | BCAT1 |
| CHD5 | MTUS1 | CPE | CCR1 | ARHGEF26 |  | RCN3 | BCAT2 |
| CHN1 | MYLK | CPXM1 | CCR5 | ARHGEF3 |  | RGS3 | BCL11B |
| CHRM1 | MYO1D | CREB5 | CCRL2 | ARHGEF4 |  | ROBO4 | BCL2L12 |
| CHRM3 | MYOT | CREBL2 | CD14 | ARIH2OS |  | SEC24D | BCL7A |
| CHST1 | MYRF | CREBRF | CD163 | ARL14 |  | SERPINH1 | BCL7C |
| CHST11 | NACAD | CRY1 | CD180 | ARL9 |  | SHC1 | BEST3 |
| CHST14 | NFASC | CSDC2 | CD1D | ARMC12 |  | SLC38A11 | BEX1 |
| CIDEA | NINJ2 | CSGALNACT1 | CD2 | ARMC2 |  | SNAI2 | BEX2 |
| CKLF | NIPA1 | CTDSPL2 | CD2AP | ARMC3 |  | SPRY1 | BEX4 |
| CLCN4 | NIPAL3 | CTNNAL1 | CD300A | ARMCX2 |  | SPRY4 | BICD1 |
| CLDN10 | NIPAL4 | CTPS1 | CD300LF | ARNTL |  | STC1 | BID |
| CLEC2D | NKAIN2 | CTPS2 | CD33 | ARPC4 |  | SUSD2 | BLCAP |
| CLEC4G | NPC1 | CTSF | CD37 | ARRB2 |  | SVIL | BMP2 |
| CLSTN1 | NRIP2 | CYLD | CD3D | ARRDC4 |  | TAGLN | BPGM |
| CLTB | NT5DC1 | CYP27B1 | CD4 | ARSF |  | TES | BRINP1 |
| CMIP | NXPE3 | DAAM2 | CD44 | ARSG |  | TFPI2 | BRINP2 |
| CMTM3 | OPALIN | DAPK1 | CD48 | ARSK |  | THBS1 | BRMS1L |
| CMTR2 | OR7A5 | DBF4 | CD52 | ARX |  | TM4SF1 | BRSK2 |
| CNKSR2 | OSBPL1A | DCAF4L2 | CD53 | AS3MT |  | TM4SF18 | BSN |
| CNTLN | PADI2 | DCPS | CD59 | ASAP1-IT1 |  | TPM2 | BTAF1 |
| CNTN4 | PAQR6 | DCTPP1 | CD69 | ASAP2 |  | TPM4 | BTBD2 |
| CNTN5 | PCBP4 | DDIAS | CD72 | ASB11 |  | TRPC6 | BTNL9 |
| CNTNAP1 | PCSK6 | DDX11 | CD74 | ASB15 |  | UACA | BTRC |
| COL4A3BP | PDE1C | DDX39A | CD84 | ASB5 |  | USP46 | C1QL1 |
| COMMD2 | PDIA2 | DEPDC1 | CD86 | ASCL2 |  | VEGFA | C1QTNF1 |
| CORO1C | PEX5L | DEPDC1B | CDK8 | ASCL3 |  | VGLL3 | C1QTNF4 |
| CORO2A | PIEZO2 | DEPTOR | CDKN1A | ASIC5 |  | VWF | C1QTNF6 |
| COX7A1 | PIP4K2A | DESI2 | CDKN1B | ASIP |  |  | C2CD2L |
| CPNE2 | PKP4 | DEXI | CEBPA | ASNS |  |  | C2CD5 |
| CPNE3 | PLA2G4C | DHRS13 | CEBPB | ASPSCR1 |  |  | CA10 |
| CPNE4 | PLCL1 | DHX9 | CEBPD | ASS1 |  |  | CA12 |
| CPNE6 | PLD1 | DLGAP5 | CEP72 | ASZ1 |  |  | CA2 |
| CPNE9 | PLEKHB1 | DLST | CETN2 | ATAD3C |  |  | CACNA1E |
| CREG2 | PLEKHG3 | DNA2 | CFB | ATF2 |  |  | CACNA2D1 |
| CRELD1 | PLEKHH1 | DNAAF2 | CFD | ATF7IP |  |  | CACNA2D2 |
| CRH | PLLP | DNAJB4 | CFH | ATOH1 |  |  | CACNA2D3 |
| CRHBP | PLP1 | DNMT1 | CFI | ATP13A4 |  |  | CACNG2 |
| CRISPLD1 | PLXNB3 | DNMT3B | CFLAR | ATP4A |  |  | CADM2 |
| CRYM | POPDC3 | DOCK1 | CH25H | ATP6AP1L |  |  | CADPS |
| CSPG4 | PPP1R14A | DONSON | CHCHD7 | ATP6V0A4 |  |  | CALCOCO1 |
| CTXN1 | PPP1R16B | DRAXIN | CHI3L2 | ATP6V0E2-AS1 |  |  | CALHM2 |
| CTXN3 | PRKCQ | DSCC1 | CHML | ATP6V1B1 |  |  | CALN1 |
| CX3CL1 | PRKCQ-AS1 | DSEL | CHPT1 | ATP6V1E2 |  |  | CALU |
| CYP20A1 | PRR18 | DSN1 | CHST6 | ATRX |  |  | CAMK2D |
| CYP26A1 | PRRG1 | DTL | CIB1 | ATXN8OS |  |  | CAMK2G |
| CYP26B1 | PTGDS | DTNA | CKB | AUTS2 |  |  | CAMK2N2 |
| CYP46A1 | PTK7 | DTYMK | CLCF1 | AVIL |  |  | CAMK4 |
| CYP4X1 | PTPRD | DYNLRB2 | CLDN23 | AVP |  |  | CAMSAP2 |
| DBI | PTPRK | E2F7 | CLEC12A | AVPR1B |  |  | CAMSAP3 |
| DCAF13 | PXK | E2F8 | CLEC2B | AXIN1 |  |  | CAMTA1 |
| DCAF6 | QDPR | EBF2 | CLEC4A | AZGP1 |  |  | CAND2 |
| DCLK1 | RAB40B | EBF3 | CLEC7A | AZGP1P1 |  |  | CAPN5 |
| DDAH2 | RAP1GDS1 | ECT2 | CLECL1 | B3GALNT1 |  |  | CAPS2 |
| DDN | RAPGEF3 | EDN3 | CLGN | B3GNT2 |  |  | CARD8 |
| DDX55 | RAPGEF5 | EFHD1 | CLIC2 | B3GNT8 |  |  | CASD1 |
| DERA | RASGEF1C | EIF4EBP1 | CLU | B4GALNT1 |  |  | CASKIN1 |
| DGKZ | RASGRP3 | EMC9 | CMAHP | B4GALNT2 |  |  | CASP6 |
| DHCR24 | RBP7 | EN1 | CMTM6 | B9D1 |  |  | CASP7 |
| DHRS11 | RGL1 | ENKUR | CMTM7 | BAALC |  |  | CASP8 |
| DIRAS1 | RHBDL2 | ENPP4 | COL14A1 | BAAT |  |  | CAST |
| DIRAS2 | RNASE1 | ENPP5 | COL8A2 | BACH2 |  |  | CBFA2T3 |
| DISP1 | RNF125 | EPHB3 | COLEC12 | BAG2 |  |  | CBLN2 |
| DKK3 | RNF220 | EPHX1 | COPZ2 | BAMBI |  |  | CBR4 |
| DLEU1 | ROGDI | ERCC6L | CORO1A | BANF1 |  |  | CBX6 |
| DLG2 | S100A1 | ESPL1 | COTL1 | BARHL1 |  |  | CBX8 |
| DLG5 | S100B | ETNPPL | CP | BARX1 |  |  | CCDC102A |
| DLGAP2 | S1PR5 | ETS2 | CPD | BAZ2B |  |  | CCDC102B |
| DMTN | SEC14L5 | EXO1 | CPED1 | BBOX1 |  |  | CCDC146 |
| DNAJA4 | SEMA4D | EXOSC9 | CPM | BBS10 |  |  | CCDC6 |
| DNAJB14 | SEPTIN4 | EXPH5 | CPPED1 | BBS12 |  |  | CCDC8 |
| DNAL4 | SH3GL3 | EZH2 | CPQ | BBX |  |  | CCDC80 |
| DNM1 | SH3PXD2A | F5 | CPT1C | BCAR3 |  |  | CCDC85A |
| DOC2A | SHISA2 | F8 | CPVL | BCHE |  |  | CCDC85B |
| DOCK11 | SIRT2 | FAM102A | CRB2 | BCKDHB |  |  | CCNG2 |
| DOCK9 | SLAIN1 | FAM107A | CREG1 | BCL6 |  |  | CCSAP |
| DPP3 | SLC12A2 | FAM111B | CRMP1 | BDNF |  |  | CCSER2 |
| DPYSL3 | SLC22A15 | FAM126A | CSF1R | BEND2 |  |  | CD109 |
| DRD1 | SLC24A2 | FAM13A | CSF3R | BEND5 |  |  | CD151 |
| DUS3L | SLC26A9 | FAM189A2 | CSPG5 | BEST4 |  |  | CD164 |
| DUSP2 | SLC31A2 | FAM200A | CSTA | BFSP1 |  |  | CD200 |
| DYNLT1 | SLC45A3 | FAM83D | CTSB | BFSP2 |  |  | CD276 |
| DYSF | SLC48A1 | FANCE | CTSC | BHLHE23 |  |  | CD320 |
| E2F5 | SLC5A11 | FANCG | CTSD | BHMT |  |  | CD58 |
| EDNRA | SLCO1A2 | FANCI | CTSH | BHMT2 |  |  | CD63 |
| EEF1A2 | SLCO3A1 | FBL | CTSL | BLOC1S1 |  |  | CD9 |
| EEF1D | SNX30 | FBXO15 | CTSS | BMF |  |  | CDC42BPB |
| EFHD2 | SORT1 | FBXO31 | CTSZ | BMP3 |  |  | CDH10 |
| EFNA3 | SOX10 | FBXO43 | CTTNBP2NL | BMP4 |  |  | CDH11 |
| EFR3A | SPOCK3 | FBXO5 | CX3CR1 | BMP5 |  |  | CDH13 |
| EGFL7 | ST18 | FBXW4 | CXCL12 | BMP6 |  |  | CDH18 |
| EGR3 | ST6GALNAC3 | FEN1 | CXCL16 | BMP7 |  |  | CDH2 |
| EGR4 | SUN2 | FGF2 | CXCL2 | BMPER |  |  | CDH6 |
| EID2B | SYNDIG1 | FGFR1OP | CXCL3 | BMPR1A |  |  | CDH9 |
| EIF4E3 | SYNJ2 | FIGNL1 | CXCL5 | BNIP3 |  |  | CDHR1 |
| EIF5A2 | TBC1D12 | FMNL1 | CXCL6 | BOC |  |  | CDIP1 |
| ELN | TCFL5 | FMO2 | CXCL8 | BPIFB2 |  |  | CDK5R1 |
| EMX1 | TESK2 | FOXM1 | CXCR2 | BPIFB4 |  |  | CDK5R2 |
| EMX2 | TF | FOXO4 | CXCR4 | BRDT |  |  | CDKL1 |
| EMX2OS | TLE4 | FUS | CXXC4 | BRINP3 |  |  | CDR1 |
| ENC1 | TLL2 | GAB2 | CYB561A3 | BSG |  |  | CEBPG |
| ENKD1 | TMC6 | GABRG1 | CYB5R1 | BTBD11 |  |  | CELF2 |
| ENO2 | TMCC2 | GAS1 | CYBA | BTBD16 |  |  | CELF3 |
| ENTPD3 | TMCC3 | GAS2L1 | CYBB | BTBD8 |  |  | CELF4 |
| ENTPD6 | TMEM125 | GAS2L3 | CYBRD1 | BTC |  |  | CELF5 |
| EPB41L1 | TMEM144 | GCLC | CYFIP1 | BTLA |  |  | CELF6 |
| EPHA4 | TMEM151A | GDF10 | CYP27A1 | BTN1A1 |  |  | CELSR1 |
| EPHA5 | TMEM178A | GDPD5 | CYSTM1 | BVES |  |  | CELSR3 |
| EPHB6 | TMEM63A | GGH | CYTIP | C1D |  |  | CEND1 |
| EPHX4 | TMTC4 | GHR | DAB2 | C1GALT1 |  |  | CEP112 |
| EPN3 | TP53INP2 | GINS2 | DCBLD2 | C1QTNF3 |  |  | CEP68 |
| EPS15 | TPPP | GINS3 | DCLRE1A | C2CD4B |  |  | CGREF1 |
| ERC2 | TPRN | GINS4 | DCX | C4BPA |  |  | CHD3 |
| ERICH3 | TRIM59 | GJC1 | DCXR | C5 |  |  | CHD7 |
| ETAA1 | TSPAN15 | GLUD1 | DDIT4L | C5AR2 |  |  | CHGA |
| ETS1 | TSPAN8 | GLUD2 | DDR2 | C6 |  |  | CHGB |
| EXOSC8 | TTLL7 | GLUL | DEF6 | C8B |  |  | CHI3L1 |
| EXTL1 | TTYH2 | GPC2 | DENND2D | CA14 |  |  | CHIC2 |
| F2R | TUBB4A | GPR146 | DENND3 | CA5A |  |  | CHPF |
| FABP3 | TULP4 | GPR161 | DEPDC5 | CA8 |  |  | CHPF2 |
| FABP6 | TYRO3 | GRAMD1C | DHRS3 | CAAP1 |  |  | CHRNA9 |
| FAM102B | UGT8 | GRB14 | DICER1-AS1 | CABP2 |  |  | CHST2 |
| FAM131A | ULK2 | GRHL3 | DKK1 | CABP7 |  |  | CHSY1 |
| FAM174A | YPEL2 | GRINA | DLGAP1-AS1 | CACHD1 |  |  | CISD1 |
| FAM49A | ZEB2 | GTF2H4 | DNAH9 | CACNA1S |  |  | CISH |
| FAM50B | ZNF536 | GTF2I | DNAJB1 | CACNG1 |  |  | CKAP4 |
| FAM71E1 |  | GTSE1 | DNAJC3 | CADM1 |  |  | CLASP2 |
| FBN2 |  | GYG1 | DNASE1L1 | CADM4 |  |  | CLCN6 |
| FBXL2 |  | H2AFX | DOCK2 | CAGE1 |  |  | CLDN12 |
| FBXO16 |  | HAND2-AS1 | DOCK8 | CALCB |  |  | CLEC5A |
| FBXW7 |  | HAUS1 | DOK4 | CALCRL |  |  | CLIC1 |
| FDXR |  | HAUS6 | DOK5 | CALML5 |  |  | CLIC4 |
| FEZF2 |  | HAUS8 | DPEP2 | CALML6 |  |  | CLIP3 |
| FIGN |  | HELLS | DPH3 | CALR3 |  |  | CLN5 |
| FKBP1B |  | HEPH | DPP6 | CAMP |  |  | CLSTN2 |
| FKBP7 |  | HIST1H1E | DPYD | CAPN11 |  |  | CLVS1 |
| FKBP8 |  | HIST1H2AE | DRAM2 | CAPN12 |  |  | CMTM4 |
| FNDC9 |  | HIST1H2AM | DSE | CAPN14 |  |  | CMTM5 |
| FOCAD |  | HIST1H3B | DTX1 | CAPN6 |  |  | CMYA5 |
| FOXP1 |  | HIST1H3C | DTX4 | CAPRIN2 |  |  | CNGA3 |
| FSTL5 |  | HJURP | DUSP1 | CAPS |  |  | CNIH2 |
| FXYD1 |  | HMGB2 | DUSP23 | CAPSL |  |  | CNIH4 |
| GABARAPL1 |  | HMGB3P1 | DZIP1 | CAPZA3 |  |  | CNN3 |
| GABPA |  | HMGN1 | EBI3 | CARM1 |  |  | CNNM1 |
| GABRA1 |  | HMMR | ECM2 | CASC1 |  |  | CNRIP1 |
| GABRA2 |  | HNRNPAB | ECRP | CASP9 |  |  | CNST |
| GABRA4 |  | HOGA1 | ECSIT | CASQ2 |  |  | CNTN1 |
| GABRA5 |  | HOXA1 | EFEMP1 | CATSPER1 |  |  | CNTN6 |
| GABRB1 |  | HOXA10 | EFHC2 | CATSPERD |  |  | CNTNAP2 |
| GABRB2 |  | HOXA11 | EFNA1 | CAV3 |  |  | CNTNAP5 |
| GABRD |  | HOXA2 | EGF | CBLN3 |  |  | COL22A1 |
| GAD2 |  | HOXA3 | EGR1 | CBR3 |  |  | COL26A1 |
| GALK1 |  | HOXA4 | EGR2 | CCAR2 |  |  | COL4A6 |
| GALNT16 |  | HOXA5 | ELF1 | CCDC105 |  |  | COLGALT1 |
| GALNT18 |  | HOXA7 | ELL2 | CCDC112 |  |  | COQ2 |
| GALNTL5 |  | HOXC10 | EMP2 | CCDC113 |  |  | CORO6 |
| GAS7 |  | HOXC11 | ENDOG | CCDC114 |  |  | CORO7 |
| GDA |  | HOXC13 | ENTPD1 | CCDC136 |  |  | COX20 |
| GFRA2 |  | HOXC4 | ERMAP | CCDC140 |  |  | CPEB3 |
| GJB6 |  | HOXC6 | ESYT1 | CCDC142 |  |  | CPLX1 |
| GLS |  | HOXC9 | EVA1C | CCDC151 |  |  | CPLX2 |
| GLS2 |  | HOXD10 | EVI2B | CCDC153 |  |  | CPNE5 |
| GLT1D1 |  | HOXD11 | F13A1 | CCDC160 |  |  | CPT2 |
| GMNN |  | HOXD13 | F2RL2 | CCDC170 |  |  | CRB1 |
| GNA12 |  | HOXD4 | F3 | CCDC172 |  |  | CREB3L2 |
| GNG13 |  | HOXD9 | FAH | CCDC173 |  |  | CRNDE |
| GNG3 |  | HPR | FAM104B | CCDC178 |  |  | CROT |
| GORAB |  | HPSE2 | FAM114A1 | CCDC181 |  |  | CRTAC1 |
| GOT1 |  | HSD11B2 | FAM155B | CCDC185 |  |  | CRY2 |
| GPC5 |  | HSD17B14 | FAM181A | CCDC24 |  |  | CRYL1 |
| GPD1L |  | HSDL2 | FAM20A | CCDC26 |  |  | CRYZ |
| GPR22 |  | HSPA1L | FAP | CCDC30 |  |  | CSMD1 |
| GPR26 |  | HSPB8 | FBLN5 | CCDC54 |  |  | CSMD3 |
| GPR83 |  | HTRA1 | FBP1 | CCDC58 |  |  | CSRNP3 |
| GPR88 |  | IER5 | FBXO32 | CCDC60 |  |  | CSRP2 |
| GPRASP1 |  | IGIP | FCER1G | CCDC63 |  |  | CSTF2T |
| GRASP |  | IGSF1 | FCGBP | CCDC65 |  |  | CTBS |
| GREM2 |  | ILF2 | FCGR2B | CCDC70 |  |  | CTNNA2 |
| GRIN1 |  | INCENP | FCGR3B | CCDC71L |  |  | CTNNA3 |
| GRIN2A |  | INSM1 | FCGRT | CCDC78 |  |  | CTSA |
| GRM1 |  | INTS7 | FERMT3 | CCDC81 |  |  | CTSV |
| GRM2 |  | IRAK1BP1 | FES | CCDC83 |  |  | CTTNBP2 |
| GSKIP |  | ISL2 | FGD1 | CCDC84 |  |  | CUTC |
| GSTM5 |  | ITGB3BP | FGF20 | CCDC88C |  |  | CUX2 |
| GSTO2 |  | IZUMO4 | FGL2 | CCDC89 |  |  | CYB561 |
| HAPLN4 |  | JADE2 | FGR | CCDC91 |  |  | CYFIP2 |
| HAS1 |  | KBTBD2 | FHOD1 | CCDC96 |  |  | CYP2E1 |
| HBQ1 |  | KCNN3 | FILIP1L | CCER1 |  |  | CYTH1 |
| HCN1 |  | KCTD21 | FLI1 | CCL11 |  |  | CYTL1 |
| HENMT1 |  | KDM1A | FLNC | CCL13 |  |  | DAB1 |
| HEY1 |  | KIF11 | FOLR2 | CCL16 |  |  | DACH2 |
| HIF1A |  | KIF14 | FOS | CCL17 |  |  | DACT3 |
| HIVEP2 |  | KIF15 | FOSL1 | CCL18 |  |  | DAGLA |
| HK1 |  | KIF18A | FOSL2 | CCL19 |  |  | DAP |
| HMGCLL1 |  | KIF20A | FOXRED2 | CCL21 |  |  | DBP |
| HNRNPF |  | KIF20B | FPR1 | CCL25 |  |  | DCHS1 |
| HOMER1 |  | KIF23 | FPR2 | CCL26 |  |  | DCTD |
| HOOK1 |  | KIF2C | FPR3 | CCL7 |  |  | DCTN1 |
| HPCA |  | KIF4A | FRMD3 | CCM2L |  |  | DDB2 |
| HPCAL1 |  | KIF6 | FRMPD1 | CCND1 |  |  | DDOST |
| HPCAL4 |  | KIFC1 | FSIP1 | CCND3 |  |  | DDX25 |
| HPRT1 |  | KLF10 | FUCA1 | CCNJ |  |  | DEAF1 |
| HS3ST2 |  | KLF2 | FXYD5 | CCNL2 |  |  | DEDD2 |
| HS3ST4 |  | KLHDC8A | FZD6 | CCNO |  |  | DENND2A |
| HS6ST3 |  | KLHL5 | G0S2 | CCR10 |  |  | DENND5A |
| HSD11B1 |  | KNTC1 | G6PD | CCR3 |  |  | DERL2 |
| HSD17B6 |  | KPNA2 | GAA | CCR6 |  |  | DGAT2 |
| HSPA12A |  | LAPTM4B | GAB3 | CCR8 |  |  | DGCR5 |
| HSPB3 |  | LCNL1 | GADD45B | CCT6B |  |  | DGKB |
| HSPH1 |  | LDHD | GAL3ST4 | CCT8L2 |  |  | DGKI |
| HTR2A |  | LDLRAD4 | GALM | CD163L1 |  |  | DHTKD1 |
| HTR2C |  | LHFPL1 | GALNT15 | CD177 |  |  | DIP2C |
| HTR3B |  | LIG1 | GALNT7 | CD1B |  |  | DIRAS3 |
| HTR5A |  | LIMCH1 | GAPT | CD1C |  |  | DISP2 |
| HYLS1 |  | LIN9 | GAS6 | CD247 |  |  | DLC1 |
| ICAM5 |  | LIX1 | GATAD2B | CD38 |  |  | DLG4 |
| ID3 |  | LMNB1 | GBE1 | CD3E |  |  | DLGAP1 |
| IDH1 |  | LMNB2 | GBGT1 | CD55 |  |  | DLGAP3 |
| IDH3A |  | LMO1 | GBP1 | CD70 |  |  | DLK2 |
| IDI2-AS1 |  | LOC441179 | GBP2 | CD82 |  |  | DLL1 |
| IDS |  | LOC730101 | GBP3 | CD83 |  |  | DLL3 |
| IGF2R |  | LPCAT1 | GCA | CD8A |  |  | DMRTA2 |
| IGSF8 |  | LRR1 | GCH1 | CDA |  |  | DNAAF3 |
| IL10RB |  | LRRC23 | GCLM | CDC14B |  |  | DNAJB6 |
| IL12RB2 |  | LSM5 | GEM | CDC37L1 |  |  | DNAJC12 |
| IMPDH2 |  | LSM7 | GFAP | CDC42BPA |  |  | DNAJC18 |
| IMPG1 |  | LSR | GFPT2 | CDC42EP1 |  |  | DNAJC2 |
| INPP5J |  | LYPD6 | GIMAP1 | CDC42EP3 |  |  | DNAJC22 |
| IPCEF1 |  | LYPLA1 | GIMAP2 | CDC42EP4 |  |  | DNALI1 |
| IQSEC1 |  | MAD1L1 | GIMAP4 | CDH1 |  |  | DNER |
| ITGAV |  | MAD2L1 | GIMAP6 | CDH16 |  |  | DNM3 |
| ITPKA |  | MAD2L2 | GIMAP7 | CDH17 |  |  | DOCK3 |
| ITPR1 |  | MAOA | GIMAP8 | CDH20 |  |  | DOCK7 |
| JAG2 |  | MAP3K1 | GJB2 | CDH3 |  |  | DOK6 |
| JAKMIP1 |  | MAP3K5 | GLA | CDH4 |  |  | DPF1 |
| JAM2 |  | MAPK3 | GLCCI1 | CDH7 |  |  | DPP10 |
| JPH1 |  | MASTL | GLIPR1 | CDK9 |  |  | DPP4 |
| KALRN |  | MCM10 | GLRX | CDKAL1 |  |  | DPP7 |
| KCNA1 |  | MCM2 | GM2A | CDKN2A |  |  | DPY19L1 |
| KCNA2 |  | MCM3 | GMFG | CDKN2B |  |  | DPYSL4 |
| KCNA3 |  | MCM4 | GNA15 | CDNF |  |  | DRAM1 |
| KCNAB2 |  | MCM5 | GNAI2 | CDO1 |  |  | DRP2 |
| KCNG3 |  | MCM6 | GNG4 | CDON |  |  | DSCAM |
| KCNH3 |  | MCM7 | GNGT2 | CDR2L |  |  | DSCAML1 |
| KCNIP4 |  | MCM8 | GNLY | CEACAM19 |  |  | DSTYK |
| KCNJ3 |  | MDH1B | GPM6A | CEACAM8 |  |  | DUSP26 |
| KCNJ4 |  | MEGF9 | GPNMB | CECR2 |  |  | DUSP9 |
| KCNJ6 |  | MELK | GPR132 | CEL |  |  | DYNC1I1 |
| KCNK1 |  | MEST | GPR160 | CELA2B |  |  | DYNLT3 |
| KCNK12 |  | METTL1 | GPR183 | CEMIP |  |  | DYRK3 |
| KCNMA1 |  | METTL7A | GPR34 | CENPB |  |  | DZIP3 |
| KCNN1 |  | MEX3A | GPR65 | CENPT |  |  | ECHDC2 |
| KCNQ3 |  | MEX3D | GPR82 | CENPV |  |  | ECI2 |
| KCNQ5 |  | MGAT4C | GPR84 | CEP131 |  |  | EDEM2 |
| KCNS1 |  | MGME1 | GPRC5A | CEP19 |  |  | EFEMP2 |
| KCNV1 |  | MGMT | GPX1 | CEP350 |  |  | EFHC1 |
| KCTD1 |  | MIS18A | GRIA3 | CEP41 |  |  | EFNA4 |
| KCTD11 |  | MIS18BP1 | GRIK2 | CEP44 |  |  | EFNB2 |
| KCTD16 |  | MKI67 | GRN | CEP83 |  |  | EFNB3 |
| KCTD3 |  | MKX | GSDMD | CERS4 |  |  | EFS |
| KDM3A |  | MMP28 | GSE1 | CES1P1 |  |  | EHD3 |
| KIAA0319 |  | MND1 | GSN | CETP |  |  | EIF3L |
| KIAA0513 |  | MNS1 | GXYLT2 | CFHR2 |  |  | EIF4E2 |
| KIAA1211L |  | MRAS | GYPC | CFL1 |  |  | ELAVL2 |
| KIAA1324 |  | MRO | GZMA | CFP |  |  | ELAVL3 |
| KIF17 |  | MROH7 | GZMH | CHAD |  |  | ELAVL4 |
| KIF5A |  | MRPL12 | GZMK | CHAT |  |  | ELF4 |
| KIFC2 |  | MRPS17 | H2AFJ | CHD9 |  |  | ELFN2 |
| KLC1 |  | MRTO4 | H2AFY2 | CHIT1 |  |  | ELK3 |
| KLC2 |  | MRVI1 | HAMP | CHKA |  |  | ELMO1 |
| KLHDC9 |  | MSANTD3 | HAVCR2 | CHL1 |  |  | ELMOD2 |
| KLHL1 |  | MSH2 | HCK | CHODL |  |  | ELOVL2 |
| KLHL26 |  | MTBP | HCLS1 | CHRDL2 |  |  | ELOVL4 |
| KLK7 |  | MTFR2 | HCST | CHRM2 |  |  | EMILIN1 |
| KNDC1 |  | MXD3 | HDHD3 | CHRM4 |  |  | EMILIN2 |
| KRT222 |  | MXI1 | HEBP1 | CHRNA1 |  |  | EMP1 |
| L3HYPDH |  | MYB | HEG1 | CHRNB1 |  |  | EMP3 |
| LAMP5 |  | MYBL1 | HES5 | CHRNB4 |  |  | EN2 |
| LBR |  | MYBL2 | HGF | CHST3 |  |  | ENHO |
| LCAT |  | MYBPC1 | HHEX | CHST7 |  |  | ENO1 |
| LDB2 |  | MYLIP | HILPDA | CHST8 |  |  | ENOX1 |
| LGI1 |  | MYO5B | HIST1H1C | CHST9 |  |  | EPB41L4A-AS1 |
| LGI4 |  | MYOM1 | HIST1H2BD | CHSY3 |  |  | EPHA2 |
| LHX2 |  | N4BP2L1 | HIST1H2BK | CHURC1 |  |  | EPHB1 |
| LHX6 |  | NASP | HK3 | CIART |  |  | EPRS |
| LIMA1 |  | NCAPD2 | HLA-DMA | CITED1 |  |  | ERAP2 |
| LIN7B |  | NCAPD3 | HLA-DMB | CITED2 |  |  | ERBB2 |
| LINGO2 |  | NCAPG | HLA-DPA1 | CITED4 |  |  | ERBB4 |
| LMO4 |  | NCAPG2 | HLA-DPB1 | CKMT2 |  |  | ERF |
| LMO7 |  | NCAPH | HLA-DQA1 | CLCA1 |  |  | ERRFI1 |
| LNX1 |  | NCBP1 | HLA-DQB1 | CLCA3P |  |  | ESRRG |
| LOC153684 |  | NCLN | HLA-DRA | CLCN1 |  |  | EVC |
| LOC730098 |  | NDC1 | HLA-DRB6 | CLDN16 |  |  | EVC2 |
| LPCAT3 |  | NDC80 | HLX | CLDN2 |  |  | EVL |
| LPCAT4 |  | NEBL | HMGB3 | CLDN5 |  |  | EXOC6 |
| LPGAT1 |  | NEIL3 | HMGCR | CLDN7 |  |  | EXOC6B |
| LRFN2 |  | NEK2 | HMGCS1 | CLDN8 |  |  | EYA1 |
| LRFN5 |  | NETO2 | HMOX1 | CLEC10A |  |  | EYA2 |
| LRP11 |  | NHLH1 | HNMT | CLEC1A |  |  | EYA4 |
| LRP5 |  | NKX3-2 | HP | CLEC3A |  |  | F11R |
| LRRC3B |  | NLGN4X | HPGDS | CLHC1 |  |  | F2RL1 |
| LRRC7 |  | NLRP11 | HPSE | CLIC3 |  |  | FABP7 |
| LRRC73 |  | NMU | HS3ST1 | CLIC6 |  |  | FAIM |
| LRTM2 |  | NOC4L | HSD3B7 | CLIP2 |  |  | FAIM2 |
| LSM2 |  | NOM1 | HSF2BP | CLK1 |  |  | FAM110B |
| LSM8 |  | NONO | HSP90AB1 | CLMP |  |  | FAM111A |
| LTBP3 |  | NR2F1 | HSPA5 | CLPTM1 |  |  | FAM126B |
| LUZP1 |  | NR3C2 | HSPA6 | CLRN3 |  |  | FAM131B |
| LY6E |  | NREP | HSPBAP1 | CLTCL1 |  |  | FAM133A |
| LY6H |  | NRM | HTATIP2 | CLUL1 |  |  | FAM13C |
| LYSMD4 |  | NT5C2 | HTR2B | CLVS2 |  |  | FAM149A |
| MAK |  | NT5C3A | HTRA4 | CLYBL |  |  | FAM155A |
| MAL2 |  | NT5DC2 | HUNK | CMA1 |  |  | FAM160B1 |
| MALT1 |  | NT5DC3 | HVCN1 | CMTM1 |  |  | FAM171A1 |
| MAML2 |  | NTRK2 | IBSP | CMTM8 |  |  | FAM171B |
| MAP1LC3A |  | NTSR2 | ICAM1 | CNFN |  |  | FAM201A |
| MAP2K1 |  | NUDT1 | IER2 | CNIH3 |  |  | FAM210A |
| MAP3K9 |  | NUF2 | IER3 | CNKSR3 |  |  | FAM214A |
| MAP7D2 |  | NUP107 | IER5L | CNN1 |  |  | FAM216A |
| MAP9 |  | NUP155 | IFI16 | CNNM4 |  |  | FAM219A |
| MAPK7 |  | NUP205 | IFITM1 | CNR1 |  |  | FAM221A |
| MAPK9 |  | NUP35 | IFITM2 | CNTD1 |  |  | FAM222A |
| MAPRE3 |  | NUP62 | IFITM3 | CNTD2 |  |  | FAR2 |
| MAST3 |  | NUSAP1 | IFNGR1 | CNTF |  |  | FAS |
| MATK |  | NVL | IGDCC3 | COCH |  |  | FAXC |
| MBNL2 |  | ODC1 | IGFBP7 | COL10A1 |  |  | FBLL1 |
| MBOAT7 |  | OIP5 | IGFBP7-AS1 | COL11A1 |  |  | FBLN7 |
| MCHR2 |  | ORC1 | IGFLR1 | COL16A1 |  |  | FBRSL1 |
| MDH1 |  | ORC6 | IGHM | COL19A1 |  |  | FBXL15 |
| ME1 |  | OSBPL11 | IGKC | COL20A1 |  |  | FBXL16 |
| MEF2C |  | OTP | IGLON5 | COL21A1 |  |  | FBXL7 |
| MEG3 |  | OTUD1 | IGSF6 | COL23A1 |  |  | FBXO17 |
| MEPE |  | PAFAH1B3 | IL10 | COL24A1 |  |  | FBXO27 |
| MGAT5B |  | PAICS | IL10RA | COL28A1 |  |  | FCHSD2 |
| MGLL |  | PAQR8 | IL13RA1 | COL2A1 |  |  | FDFT1 |
| MICAL2 |  | PARP8 | IL13RA2 | COL4A3 |  |  | FEM1C |
| MIDN |  | PARPBP | IL15 | COL4A4 |  |  | FERMT1 |
| MIF4GD |  | PAX6 | IL15RA | COL4A5 |  |  | FERMT2 |
| MMD |  | PAXIP1 | IL18 | COL5A3 |  |  | FGF12 |
| MORC4 |  | PBK | IL18BP | COL9A1 |  |  | FGF13 |
| MPPED1 |  | PC | IL1B | COL9A3 |  |  | FGF14 |
| MPZL1 |  | PCCB | IL1R1 | COLEC11 |  |  | FGF9 |
| MRAP2 |  | PCDHB13 | IL1R2 | COLGALT2 |  |  | FGFRL1 |
| MRPL41 |  | PCDHB14 | IL21R | COLQ |  |  | FHL3 |
| MTHFD2 |  | PCDHB3 | IL2RA | COMP |  |  | FHOD3 |
| MTUS2 |  | PCDHB8 | IL4I1 | COPE |  |  | FIBIN |
| MVB12A |  | PCGF5 | IL4R | COPG1 |  |  | FJX1 |
| MYC |  | PCNA | IL6 | CORO2B |  |  | FKBP10 |
| MYH7B |  | PDE4DIP | IL6R | COX8C |  |  | FKBP14 |
| MYO5A |  | PDK4 | IL7R | CPA1 |  |  | FKBP5 |
| MYPOP |  | PEBP1 | IMPA2 | CPA3 |  |  | FLNA |
| MYRIP |  | PEBP4 | IMPACT | CPA5 |  |  | FLRT1 |
| N4BP2 |  | PER3 | INPP5D | CPA6 |  |  | FLT3LG |
| NAGLU |  | PFAS | IQCG | CPAMD8 |  |  | FMOD |
| NAGPA |  | PGBD1 | IRF8 | CPB1 |  |  | FNBP1L |
| NAPA |  | PHF19 | ITGA3 | CPB2 |  |  | FNDC3B |
| NAPB |  | PHF5A | ITGAM | CPEB2 |  |  | FNDC5 |
| NAPEPLD |  | PHYHD1 | ITGAX | CPEB4 |  |  | FOXD1 |
| NAPG |  | PIF1 | ITGB2 | CPNE1 |  |  | FOXO1 |
| NAT8L |  | PIGW | ITGB4 | CPNE8 |  |  | FPGT |
| NAV3 |  | PIK3IP1 | ITGB5 | CPS1 |  |  | FRA10AC1 |
| NBL1 |  | PIK3R3 | ITK | CPSF1 |  |  | FRAT1 |
| NCALD |  | PINK1 | ITPKB | CPXM2 |  |  | FREM3 |
| NCDN |  | PITX2 | ITPKC | CRABP1 |  |  | FRMD4A |
| NCEH1 |  | PKD2L1 | JADE1 | CRABP2 |  |  | FRMPD3 |
| NCOA7 |  | PKN3 | JUNB | CREB3L1 |  |  | FRRS1L |
| NCS1 |  | PLCXD3 | KAT2A | CREB3L3 |  |  | FRY |
| NDEL1 |  | PLEKHA5 | KAT6B | CREB3L4 |  |  | FSD1 |
| NDFIP2 |  | PLIN1 | KCND2 | CRIM1 |  |  | FSD1L |
| NDST3 |  | PLK1 | KCNJ11 | CRIP2 |  |  | FUCA2 |
| NECAB1 |  | PLK4 | KCNJ8 | CRIPAK |  |  | FUT9 |
| NEDD4 |  | PMCH | KCNK7 | CRISP2 |  |  | FXYD6 |
| NEDD4L |  | POC1A | KCNMB1 | CRISP3 |  |  | FXYD7 |
| NEFH |  | POLA1 | KCNN4 | CRISPLD2 |  |  | FZD1 |
| NEFM |  | POLD1 | KCNQ1 | CRLF1 |  |  | FZD2 |
| NELL1 |  | POLE | KCNQ2 | CRNN |  |  | FZD5 |
| NELL2 |  | POLE2 | KCTD12 | CRTAM |  |  | FZD7 |
| NEUROD2 |  | POLQ | KDM3B | CRYAA |  |  | GABBR1 |
| NEUROD6 |  | POU3F2 | KIAA1549 | CRYAB |  |  | GABBR2 |
| NGB |  | PPAT | KIAA1586 | CRYBA1 |  |  | GABRA3 |
| NGEF |  | PPIH | KIF16B | CRYBA4 |  |  | GABRB3 |
| NIPAL2 |  | PPM1D | KIF26A | CRYBB2 |  |  | GABRG2 |
| NIPSNAP3B |  | PPM1K | KLF15 | CRYBG3 |  |  | GAD1 |
| NLK |  | PPP1R1B | KLHL23 | CRYGA |  |  | GADD45A |
| NME4 |  | PPP1R3C | KLHL28 | CRYGC |  |  | GADD45G |
| NNAT |  | PPP2R3A | KLRB1 | CRYGD |  |  | GALNT10 |
| NOS1AP |  | PPP2R5A | KMO | CRYGS |  |  | GALNT13 |
| NPM2 |  | PPP5C | KMT2A | CSAD |  |  | GALNT14 |
| NPTN |  | PRC1 | KYNU | CSMD2 |  |  | GALNT2 |
| NPTX1 |  | PRDM13 | LACTB2 | CSN1S1 |  |  | GALNT9 |
| NPY |  | PRELP | LAIR1 | CSN1S2AP |  |  | GARNL3 |
| NPY1R |  | PRICKLE2 | LAMA2 | CSNK1E |  |  | GATAD1 |
| NPY5R |  | PRIM1 | LAMP2 | CSPG4P5 |  |  | GBF1 |
| NRGN |  | PRKAG2 | LAMP3 | CSRNP1 |  |  | GDAP1 |
| NRIP3 |  | PRMT1 | LAPTM5 | CST1 |  |  | GDAP1L1 |
| NRN1 |  | PRMT6 | LAT2 | CST3 |  |  | GDF15 |
| NRSN2 |  | PROX1 | LCK | CST5 |  |  | GDPD1 |
| NRXN3 |  | PRUNE2 | LCP1 | CST6 |  |  | GDPD2 |
| NSF |  | PSMC3IP | LCP2 | CST7 |  |  | GFOD1 |
| NUAK1 |  | PSMD8 | LEPROT | CST8 |  |  | GFRA1 |
| NUDT14 |  | PSPH | LGALS9 | CTCFL |  |  | GHITM |
| NUP160 |  | PTMA | LGMN | CTDP1 |  |  | GKAP1 |
| NWD2 |  | PTN | LHFPL2 | CTDSP2 |  |  | GLB1L |
| NXT1 |  | PTPRM | LIF | CTDSPL |  |  | GLI3 |
| NXT2 |  | PTTG1 | LILRA2 | CTF1 |  |  | GLIPR2 |
| OCA2 |  | PTTG2 | LILRB1 | CTH |  |  | GLRB |
| OGDHL |  | PTTG3P | LILRB2 | CTSG |  |  | GNAI1 |
| OLFM3 |  | PURA | LILRB4 | CTSO |  |  | GNAI3 |
| OPN3 |  | PUS7 | LIMK2 | CUBN |  |  | GNAL |
| OPRK1 |  | PXMP2 | LINC00574 | CUEDC1 |  |  | GNAO1 |
| OPTN |  | PYCR1 | LINC01089 | CWH43 |  |  | GNAQ |
| OXCT1 |  | PYGB | LIPA | CXADR |  |  | GNAZ |
| OXR1 |  | RAB4B | LMO2 | CXCL1 |  |  | GNB2 |
| PABPC3 |  | RABGAP1L | LOX | CXCL13 |  |  | GNB4 |
| PACSIN1 |  | RACGAP1 | LPAR5 | CXCL14 |  |  | GNB5 |
| PAG1 |  | RAD18 | LPAR6 | CXXC5 |  |  | GNG12 |
| PAK1 |  | RAD51 | LPCAT2 | CYCS |  |  | GNG2 |
| PAK6 |  | RAD51AP1 | LPXN | CYMP |  |  | GNG5 |
| PANX2 |  | RAD54B | LRIG3 | CYP11A1 |  |  | GNL2 |
| PARM1 |  | RAD54L | LRMP | CYP1A1 |  |  | GNPTAB |
| PAXBP1 |  | RANBP3L | LRP4 | CYP27C1 |  |  | GNS |
| PCDH8 |  | RASL10A | LRRC25 | CYP2A13 |  |  | GOLGA7B |
| PCDHAC2 |  | RASL12 | LRRC49 | CYP2B7P |  |  | GPC1 |
| PCP4 |  | RASSF4 | LRRC75B | CYP2C8 |  |  | GPC4 |
| PCP4L1 |  | RAVER2 | LRRFIP1 | CYP2R1 |  |  | GPHN |
| PCSK1 |  | RBBP4 | LRRN1 | CYP2U1 |  |  | GPI |
| PCTP |  | RBBP8 | LRRN4CL | CYP2W1 |  |  | GPR137C |
| PDE1A |  | RBM3 | LSAMP | CYP39A1 |  |  | GPR155 |
| PDE1B |  | RBM34 | LSP1 | CYP3A5 |  |  | GPR158 |
| PDP1 |  | RCAN2 | LST1 | CYP4F11 |  |  | GPR162 |
| PDYN |  | RCC1 | LTBP1 | CYP4F12 |  |  | GPR17 |
| PEG3 |  | RCC2 | LTBP2 | CYP4F2 |  |  | GPR19 |
| PFKP |  | RDM1 | LTBR | CYP4F22 |  |  | GPR27 |
| PGLS |  | RECQL4 | LTC4S | CYP4F3 |  |  | GPR85 |
| PGM2L1 |  | RFC2 | LY75 | CYP4F8 |  |  | GPRIN1 |
| PGM3 |  | RFC3 | LY86 | CYP4V2 |  |  | GPX7 |
| PHACTR1 |  | RFC4 | LY96 | CYP4Z2P |  |  | GRB10 |
| PHLDA1 |  | RFC5 | LYL1 | CYP7A1 |  |  | GREB1L |
| PHYHIP |  | RFWD3 | LYN | DAAM1 |  |  | GRIA2 |
| PI4KA |  | RGS9 | LYVE1 | DACH1 |  |  | GRIA4 |
| PIN1 |  | RIN2 | LYZ | DACT1 |  |  | GRID2 |
| PIP5K1B |  | RMI1 | M6PR | DACT2 |  |  | GRIK4 |
| PITHD1 |  | RMI2 | MAFB | DAND5 |  |  | GRIN2C |
| PITPNM1 |  | RNASEH2A | MAFF | DAO |  |  | GRIN3A |
| PITPNM3 |  | RNF141 | MAGEL2 | DAPK3 |  |  | GRIP1 |
| PKN1 |  | RP9 | MAMLD1 | DAPL1 |  |  | GRM5 |
| PLD3 |  | RPA3 | MAN1A1 | DAW1 |  |  | GSTA4 |
| PLEKHA1 |  | RPL36 | MAN1C1 | DAZL |  |  | GSTK1 |
| PLEKHB2 |  | RPP40 | MAN2A1 | DBNDD1 |  |  | GUSB |
| PLEKHH3 |  | RPS6KA2 | MAN2B1 | DCAF12L1 |  |  | GYS1 |
| PLGRKT |  | RRM1 | MAOB | DCAF16 |  |  | HABP4 |
| PLK2 |  | RRM2 | MAP3K8 | DCAF4L1 |  |  | HACE1 |
| PNCK |  | RRM2B | MAPK12 | DCBLD1 |  |  | HAS2 |
| PNKD |  | RTKN2 | MARCKSL1 | DCC |  |  | HAT1 |
| PNOC |  | RUVBL1 | MARCO | DCDC2 |  |  | HCFC1R1 |
| POFUT1 |  | RYR1 | MARK1 | DCDC2B |  |  | HCN3 |
| POLI |  | S1PR1 | MARVELD1 | DCLK2 |  |  | HDAC1 |
| POLR1E |  | SALL3 | MBIP | DCLK3 |  |  | HDAC4 |
| POLR3D |  | SASH1 | MCM3AP-AS1 | DCLRE1C |  |  | HDAC5 |
| POPDC2 |  | SASS6 | MERTK | DCSTAMP |  |  | HDDC2 |
| POU6F2 |  | SCD | MEX3B | DCT |  |  | HEBP2 |
| PPEF1 |  | SCRN1 | MFNG | DCTN2 |  |  | HECW1 |
| PPFIA4 |  | SDS | MFSD1 | DCUN1D1 |  |  | HECW2 |
| PPM1H |  | SELL | MGST1 | DDA1 |  |  | HERC1 |
| PPP1R14C |  | SERPINI2 | MGST2 | DDAH1 |  |  | HES1 |
| PPP1R18 |  | SESN1 | MICALL2 | DDC |  |  | HES6 |
| PPP2R2C |  | SFRP2 | MICB | DDIT3 |  |  | HESX1 |
| PPP3CA |  | SFTPD | MILR1 | DDIT4 |  |  | HEXB |
| PPP3R1 |  | SHCBP1 | MKNK1 | DDO |  |  | HINT3 |
| PPP4R4 |  | SHE | MLKL | DDX17 |  |  | HIST1H2BE |
| PRCP |  | SHMT2 | MLLT3 | DDX3Y |  |  | HIST1H2BH |
| PRDM2 |  | SIAE | MMP19 | DDX43 |  |  | HK2 |
| PREPL |  | SIDT1 | MMP24 | DDX53 |  |  | HLF |
| PRKAR1B |  | SIRPA | MMP7 | DEFB119 |  |  | HMBOX1 |
| PRKCB |  | SKA1 | MNDA | DEFB123 |  |  | HMG20B |
| PRKCG |  | SKA2 | MOB1A | DEFB124 |  |  | HMGN5 |
| PRKD1 |  | SKA3 | MORC2-AS1 | DEFB125 |  |  | HNF4G |
| PRKD3 |  | SLC10A4 | MPEG1 | DEFB129 |  |  | HOMER3 |
| PRMT3 |  | SLC14A1 | MPPED2 | DEFB132 |  |  | HOXB2 |
| PRMT8 |  | SLC15A2 | MR1 | DEGS2 |  |  | HOXB3 |
| PRNP |  | SLC18B1 | MREG | DENND4C |  |  | HOXB7 |
| PRPF3 |  | SLC1A3 | MS4A14 | DEPDC7 |  |  | HRH1 |
| PRRG3 |  | SLC1A4 | MS4A4A | DFFB |  |  | HRK |
| PRRT3 |  | SLC20A2 | MS4A6A | DGAT1 |  |  | HS2ST1 |
| PRRX1 |  | SLC24A4 | MS4A7 | DGKG |  |  | HS3ST3A1 |
| PRSS3 |  | SLC25A18 | MSMO1 | DHCR7 |  |  | HS3ST3B1 |
| PRSS3P2 |  | SLC25A48 | MSR1 | DHRS2 |  |  | HSF2 |
| PSD3 |  | SLC2A12 | MT1E | DHX36 |  |  | HSP90B1 |
| PTBP1 |  | SLC39A11 | MT1F | DIAPH2 |  |  | HSPB11 |
| PTH2R |  | SLC39A6 | MT1G | DICER1 |  |  | HSPB6 |
| PTK2B |  | SLC46A3 | MT1HL1 | DIO2 |  |  | HUS1 |
| PTPN3 |  | SLC7A11 | MT1M | DIO3 |  |  | ICAM3 |
| PTPN5 |  | SLC7A2 | MT1X | DKFZP586I1420 |  |  | ICK |
| PTPRR |  | SMAP2 | MT2A | DKK2 |  |  | IDI1 |
| PTTG1IP |  | SMC2 | MT3 | DKK4 |  |  | IFIT5 |
| PUS3 |  | SMC4 | MTMR11 | DLEC1 |  |  | IFNGR2 |
| PVALB |  | SNRNP40 | MUC1 | DLK1 |  |  | IFRD1 |
| PXDC1 |  | SNRNP70 | MVP | DLL4 |  |  | IGDCC4 |
| PYDC1 |  | SNRPB | MXRA7 | DLX1 |  |  | IGF2BP2 |
| PYGM |  | SNRPD3 | MXRA8 | DLX2 |  |  | IGF2BP3 |
| R3HDM1 |  | SNRPF | MYCN | DLX3 |  |  | IGFBP2 |
| RAB15 |  | SORBS1 | MYD88 | DLX5 |  |  | IGFBP3 |
| RAB31 |  | SORL1 | MYH10 | DLX6 |  |  | IGFBP5 |
| RAB3B |  | SOX11 | MYO10 | DMBX1 |  |  | IGSF21 |
| RAB6B |  | SPAG5 | MYO16 | DMD |  |  | IKBIP |
| RAP1GAP2 |  | SPARCL1 | MYO1F | DMKN |  |  | IKZF5 |
| RASAL1 |  | SPC24 | MYOF | DMPK |  |  | IL17D |
| RASD2 |  | SPC25 | NABP1 | DMRT1 |  |  | IL1RAP |
| RASGEF1A |  | SPDL1 | NAGA | DMRT2 |  |  | IL1RAPL1 |
| RASGRF1 |  | SPIN4 | NAPSB | DNAAF1 |  |  | IL34 |
| RASL11B |  | SPOCK2 | NAT1 | DNAH11 |  |  | IL7 |
| RAVER1 |  | SPSB4 | NCAM1 | DNAH7 |  |  | INA |
| RBAK |  | SPX | NCAN | DNAJB5 |  |  | INHBB |
| RBCK1 |  | SREBF1 | NCF2 | DNAJB7 |  |  | INPP1 |
| RBFOX1 |  | SRGAP2 | NCF4 | DNAJC15 |  |  | INPP5A |
| RBM11 |  | SSPN | NCKAP1L | DNAL1 |  |  | INPP5F |
| RBM24 |  | SSR2 | NDST4 | DNASE1L2 |  |  | IPO4 |
| RBM28 |  | ST6GALNAC1 | NET1 | DNASE1L3 |  |  | IQGAP1 |
| RBM7 |  | ST8SIA2 | NEU1 | DNASE2B |  |  | IQGAP2 |
| RBP4 |  | STIL | NEXN | DNLZ |  |  | IQSEC3 |
| RCN1 |  | STIP1 | NFIL3 | DNM1P46 |  |  | IQUB |
| RDX |  | STOX1 | NFKBIZ | DOCK4 |  |  | IRAK1 |
| RELA |  | STT3A | NINJ1 | DOK2 |  |  | IRX5 |
| RELN |  | SUMF2 | NIPSNAP3A | DPEP1 |  |  | ISG20 |
| RERG |  | SYNPO2 | NKAIN1 | DPEP3 |  |  | ISYNA1 |
| RFPL1S |  | TACC3 | NKD1 | DPF3 |  |  | ITGA2 |
| RFPL2 |  | TAF15 | NKG7 | DPPA2 |  |  | ITGA7 |
| RGS20 |  | TAF1A | NLGN3 | DPPA4 |  |  | ITGB8 |
| RGS4 |  | TAS2R10 | NLRP3 | DPPA5 |  |  | ITPK1 |
| RHBDD2 |  | TBC1D10A | NOD2 | DPT |  |  | ITPRIP |
| RIMBP2 |  | TBC1D31 | NOVA1 | DPY19L2 |  |  | JADE3 |
| RIMS3 |  | TBC1D4 | NPC2 | DQX1 |  |  | JMJD1C |
| RNF128 |  | TCEAL3 | NPL | DRC1 |  |  | JPH3 |
| RNF175 |  | TCF3 | NPPA | DRG2 |  |  | JUN |
| ROMO1 |  | TENM1 | NQO1 | DSC1 |  |  | KANK2 |
| RORB |  | TFAP2A | NRROS | DSCR8 |  |  | KATNAL2 |
| RP2 |  | TGFBR3 | NSUN5 | DSG1 |  |  | KAZN |
| RPL12 |  | TICRR | NT5M | DSG2 |  |  | KBTBD6 |
| RPL22L1 |  | TIMELESS | NTAN1 | DSP |  |  | KBTBD7 |
| RPLP2 |  | TIPIN | NUDT22 | DTX3 |  |  | KCNA4 |
| RPRML |  | TJP2 | NUPR1 | DUOX2 |  |  | KCNAB1 |
| RRAS2 |  | TK1 | NXPH1 | DUSP10 |  |  | KCNB1 |
| RSPO2 |  | TMA16 | OGFRL1 | DUSP4 |  |  | KCNC1 |
| RSPO3 |  | TMEM106C | OLFML2B | DYDC1 |  |  | KCNC2 |
| RSPRY1 |  | TMEM192 | OLFML3 | DYDC2 |  |  | KCNE4 |
| RTBDN |  | TMEM47 | OLR1 | DYRK1B |  |  | KCNH8 |
| RTN4R |  | TMPO | ORMDL2 | DYRK2 |  |  | KCNIP2 |
| RTN4RL1 |  | TNF | OSBPL9 | DYRK4 |  |  | KCNIP3 |
| RUSC1 |  | TNPO3 | OSCAR | EAF2 |  |  | KCNJ10 |
| RXFP1 |  | TNS3 | OSM | EBF1 |  |  | KCNJ9 |
| RYBP |  | TONSL | OSTF1 | EBF4 |  |  | KCNK10 |
| RYR2 |  | TOP2A | P2RX4 | EBLN2 |  |  | KCNK3 |
| SAMD12 |  | TPX2 | P2RY12 | EBPL |  |  | KCNK4 |
| SATB2-AS1 |  | TRAF4 | P2RY13 | ECEL1 |  |  | KCNMB2 |
| SCARF2 |  | TRAIP | P2RY6 | ECHDC3 |  |  | KCNN2 |
| SCG5 |  | TRIB2 | PALD1 | EDA2R |  |  | KCTD13 |
| SCN1B |  | TRIL | PALMD | EDN1 |  |  | KCTD4 |
| SCN2B |  | TRIM24 | PAM | EDN2 |  |  | KDELR1 |
| SCN4B |  | TRIM28 | PAMR1 | EDNRB |  |  | KDELR2 |
| SCN8A |  | TRIP13 | PANK1 | EEF1A1 |  |  | KHDRBS2 |
| SDR16C5 |  | TRMT13 | PARVG | EEPD1 |  |  | KHDRBS3 |
| SEMA4A |  | TRRAP | PATZ1 | EFCAB1 |  |  | KHNYN |
| SERPINI1 |  | TSC22D3 | PBX1 | EFCAB12 |  |  | KIAA0040 |
| SERTAD4 |  | TSFM | PCDH19 | EFCAB2 |  |  | KIAA0232 |
| SERTM1 |  | TSPAN18 | PCED1B | EFCAB7 |  |  | KIAA0408 |
| SGPP2 |  | TSPAN31 | PCK2 | EFCC1 |  |  | KIAA0754 |
| SH2D5 |  | TST | PDGFRL | EFHB |  |  | KIAA1755 |
| SH3BP5 |  | TTK | PDK1 | EFNA2 |  |  | KIAA2026 |
| SH3GLB2 |  | TTYH3 | PDK3 | EGFLAM |  |  | KIF1A |
| SHISA8 |  | TUBB | PDZD8 | EGFR |  |  | KIF21B |
| SIGIRR |  | TUBG1 | PEPD | EGLN2 |  |  | KIF2A |
| SLC12A5 |  | TYMS | PERP | EGLN3 |  |  | KIF3A |
| SLC13A5 |  | UBALD2 | PFKFB3 | EHHADH |  |  | KIF3C |
| SLC17A6 |  | UBE2C | PGAP1 | EID3 |  |  | KIF9 |
| SLC17A7 |  | UBE2T | PGK1 | EIF1AY |  |  | KIFAP3 |
| SLC1A2 |  | USH1C | PHF11 | EIF3E |  |  | KIN |
| SLC25A12 |  | USP1 | PHF21B | EIF4A2 |  |  | KIT |
| SLC25A13 |  | VASH2 | PHLPP1 | EIF5A |  |  | KIZ |
| SLC25A22 |  | VPS13D | PI3 | ELANE |  |  | KLF13 |
| SLC25A4 |  | VPS37B | PIFO | ELOVL3 |  |  | KLF6 |
| SLC26A4 |  | VRK1 | PIGB | ELOVL6 |  |  | KLHDC1 |
| SLC26A8 |  | WBP2 | PIK3AP1 | EMID1 |  |  | KLHDC3 |
| SLC27A2 |  | WDHD1 | PIK3R1 | EMILIN3 |  |  | KLHL3 |
| SLC2A6 |  | WDR34 | PIM1 | EML1 |  |  | KLHL4 |
| SLC30A3 |  | WDR54 | PKM | ENAH |  |  | KLRC3 |
| SLC32A1 |  | WDR76 | PLA2G2A | ENDOD1 |  |  | KLRC4 |
| SLC35F3 |  | WEE1 | PLA2G4A | ENDOU |  |  | KPNA5 |
| SLC39A10 |  | WFS1 | PLAC8 | ENOSF1 |  |  | KRAS |
| SLC39A12 |  | XXYLT1 | PLAGL1 | ENPP1 |  |  | L1CAM |
| SLC45A1 |  | YBX1 | PLAU | ENPP7 |  |  | LAMA5 |
| SLC6A12 |  | YEATS4 | PLAUR | ENTHD1 |  |  | LAMB2 |
| SLC6A15 |  | ZBED4 | PLBD1 | ENTPD2 |  |  | LATS2 |
| SLC6A17 |  | ZBED5-AS1 | PLCG2 | EOMES |  |  | LCOR |
| SLC7A10 |  | ZBED8 | PLD4 | EPAS1 |  |  | LDHA |
| SLC8A2 |  | ZBTB16 | PLEK | EPB41L2 |  |  | LDOC1 |
| SLIT2 |  | ZBTB8A | PLEKHA7 | EPDR1 |  |  | LEF1 |
| SLITRK4 |  | ZDHHC15 | PLIN2 | EPHA3 |  |  | LETMD1 |
| SMAD1 |  | ZFP69B | PLK3 | EPHA7 |  |  | LFNG |
| SMARCA2 |  | ZFYVE21 | PLS3 | EPHX2 |  |  | LGALS1 |
| SMPX |  | ZNF124 | PLTP | EPHX3 |  |  | LGALS8 |
| SNCA |  | ZNF146 | PLXDC2 | EPS8L2 |  |  | LGR5 |
| SNCG |  | ZNF180 | PLXND1 | EPS8L3 |  |  | LIMS1 |
| SNPH |  | ZNF222 | PMEPA1 | EPYC |  |  | LINC00467 |
| SNTA1 |  | ZNF229 | PMP22 | ERAP1 |  |  | LINC00632 |
| SORBS2 |  | ZNF232 | POLD4 | ERC1 |  |  | LINC00643 |
| SORCS1 |  | ZNF235 | POMGNT2 | ERG |  |  | LINC00839 |
| SORCS2 |  | ZNF253 | POU2F1 | ERI3 |  |  | LINGO1 |
| SOSTDC1 |  | ZNF260 | PPARG | ERP27 |  |  | LIPG |
| SOWAHA |  | ZNF300 | PPCS | ERVMER61-1 |  |  | LITAF |
| SOX2 |  | ZNF302 | PPM1M | ERVV-1 |  |  | LMAN1 |
| SOX4 |  | ZNF311 | PPP1R15A | ESRP1 |  |  | LMCD1 |
| SPARC |  | ZNF320 | PRAM1 | ESX1 |  |  | LMO3 |
| SPATA2 |  | ZNF324 | PRDX1 | ETNK2 |  |  | LMTK3 |
| SPIN1 |  | ZNF385A | PRDX6 | ETV1 |  |  | LNP1 |
| SPINT2 |  | ZNF420 | PRELID2 | ETV2 |  |  | LOC389906 |
| SPRYD3 |  | ZNF426 | PREX1 | ETV4 |  |  | LOXL1 |
| SPRYD7 |  | ZNF43 | PRKCD | ETV5 |  |  | LOXL3 |
| SPTSSA |  | ZNF439 | PROS1 | EVA1A |  |  | LPIN1 |
| SRBD1 |  | ZNF443 | PRR3 | EXD1 |  |  | LPP |
| SRGAP1 |  | ZNF512B | PRSS23 | EXO5 |  |  | LRCH2 |
| SSBP3 |  | ZNF549 | PSMB10 | EXOSC10 |  |  | LRP1 |
| SST |  | ZNF558 | PSTPIP2 | EZR |  |  | LRP10 |
| SSX2IP |  | ZNF566 | PTAFR | F10 |  |  | LRP8 |
| ST6GALNAC5 |  | ZNF567 | PTER | F12 |  |  | LRRC1 |
| ST6GALNAC6 |  | ZNF581 | PTGER4 | F13B |  |  | LRRC17 |
| STAC2 |  | ZNF607 | PTGR1 | FABP2 |  |  | LRRC20 |
| STAT4 |  | ZNF649 | PTGS1 | FABP4 |  |  | LRRC4 |
| STEAP2 |  | ZNF670 | PTGS2 | FADS2 |  |  | LRRC42 |
| STK10 |  | ZNF677 | PTPN22 | FADS3 |  |  | LRRC4C |
| STK17B |  | ZNF680 | PTPN6 | FAM106A |  |  | LRRC56 |
| STK33 |  | ZNF700 | PTPRC | FAM110A |  |  | LRRC8A |
| STK36 |  | ZNF713 | PYCARD | FAM110D |  |  | LRRTM1 |
| STK38 |  | ZNF77 | QPRT | FAM118A |  |  | LRRTM2 |
| STK39 |  | ZNF789 | RAB20 | FAM124B |  |  | LRRTM4 |
| STMN1 |  | ZNF823 | RAB27A | FAM162A |  |  | LTF |
| STON1 |  | ZNF85 | RAB29 | FAM162B |  |  | LUZP2 |
| STPG1 |  | ZNF883 | RAB33B | FAM163A |  |  | LYPD1 |
| STX1A |  | ZNF92 | RAB38 | FAM167A |  |  | LYRM7 |
| STXBP5 |  | ZNF93 | RAB42 | FAM167B |  |  | LYSMD2 |
| STXBP5L |  | ZSCAN16 | RAC2 | FAM169B |  |  | LZIC |
| STXBP6 |  | ZWILCH | RALGAPA1 | FAM170B |  |  | LZTS3 |
| STYK1 |  | ZWINT | RAP1A | FAM178B |  |  | MACROD2 |
| SUCLA2 |  |  | RARRES1 | FAM181B |  |  | MADD |
| SULT4A1 |  |  | RARRES2 | FAM184A |  |  | MAGEE1 |
| SV2B |  |  | RASSF5 | FAM184B |  |  | MAGEH1 |
| SVIP |  |  | RBM47 | FAM187B |  |  | MAGT1 |
| SYN2 |  |  | RBM4B | FAM215A |  |  | MANF |
| SYNPR |  |  | RCSD1 | FAM216B |  |  | MAP1A |
| SYP |  |  | REEP6 | FAM218A |  |  | MAP1LC3C |
| SYT1 |  |  | REL | FAM221B |  |  | MAP2 |
| SYT12 |  |  | RENBP | FAM24A |  |  | MAP2K4 |
| SYT13 |  |  | RERGL | FAM3B |  |  | MAP3K14 |
| SYT5 |  |  | RETSAT | FAM43A |  |  | MAP3K6 |
| SYT7 |  |  | REXO2 | FAM43B |  |  | MAP3K7CL |
| TAC1 |  |  | RFTN1 | FAM47A |  |  | MAP4K5 |
| TAC3 |  |  | RFX7 | FAM47B |  |  | MAP6 |
| TANC1 |  |  | RGMB | FAM47C |  |  | MAPK10 |
| TBR1 |  |  | RGR | FAM71D |  |  | MAPK8IP2 |
| TCEAL7 |  |  | RGS1 | FAM71F2 |  |  | MAPK8IP3 |
| TCERG1L |  |  | RGS10 | FAM81B |  |  | MAPT |
| TCF12 |  |  | RGS16 | FAM83F |  |  | MAST1 |
| TCF7L1 |  |  | RGS18 | FANK1 |  |  | MB21D2 |
| TEAD1 |  |  | RGS19 | FARP1 |  |  | MBLAC2 |
| TENM2 |  |  | RGS2 | FAT1 |  |  | MBOAT1 |
| TESC |  |  | RHBDF2 | FAT2 |  |  | MCC |
| TESPA1 |  |  | RHBDL3 | FBLN2 |  |  | MCF2 |
| TEX29 |  |  | RHOG | FBN3 |  |  | MCF2L2 |
| TEX9 |  |  | RHOH | FBXL13 |  |  | MCL1 |
| THEMIS |  |  | RHOQ | FBXL8 |  |  | MCOLN2 |
| THOC6 |  |  | RIC3 | FBXO30 |  |  | MDFIC |
| THRB |  |  | RILPL2 | FBXO6 |  |  | MDGA2 |
| THY1 |  |  | RIN3 | FCER1A |  |  | MDK |
| TIAM2 |  |  | RNASE2 | FCN1 |  |  | MDM1 |
| TIGD2 |  |  | RNASE3 | FCRLA |  |  | ME3 |
| TIGD7 |  |  | RNASE6 | FCRLB |  |  | MEGF11 |
| TIMP4 |  |  | RNASEH2B | FDCSP |  |  | MEOX2 |
| TLE3 |  |  | RNASET2 | FETUB |  |  | METTL7B |
| TLN2 |  |  | RND3 | FEV |  |  | MFAP3 |
| TMEM123 |  |  | RNF130 | FFAR2 |  |  | MFAP4 |
| TMEM130 |  |  | RNF135 | FGF10 |  |  | MFSD10 |
| TMEM132D |  |  | RNF149 | FGF11 |  |  | MFSD6 |
| TMEM14A |  |  | RNF19A | FGF14-AS2 |  |  | MGARP |
| TMEM155 |  |  | RPS6KA1 | FGF17 |  |  | MGAT4B |
| TMEM160 |  |  | RTCA | FGF19 |  |  | MICAL3 |
| TMEM255A |  |  | RUNX3 | FGF21 |  |  | MICALL1 |
| TMEM45A |  |  | S100A10 | FGF23 |  |  | MICU1 |
| TMEM99 |  |  | S100A11 | FGF3 |  |  | MICU3 |
| TMTC1 |  |  | S100A12 | FGFBP2 |  |  | MID1 |
| TMX1 |  |  | S100A3 | FGFBP3 |  |  | MIPOL1 |
| TNIP2 |  |  | S100A4 | FGFR3 |  |  | MIR7-3HG |
| TNNT2 |  |  | S100A6 | FGG |  |  | MLLT11 |
| TOLLIP |  |  | S100A8 | FGGY |  |  | MMD2 |
| TP53 |  |  | S100A9 | FHDC1 |  |  | MMP14 |
| TP53I3 |  |  | S1PR3 | FHIT |  |  | MMP17 |
| TP53INP1 |  |  | SAMSN1 | FHL1 |  |  | MMP2 |
| TPD52L1 |  |  | SAPCD2 | FHL2 |  |  | MN1 |
| TPRG1L |  |  | SASH3 | FHL5 |  |  | MOAP1 |
| TRAF3IP2 |  |  | SAT1 | FIBCD1 |  |  | MOB3B |
| TRHDE |  |  | SBK1 | FICD |  |  | MORN4 |
| TRIM4 |  |  | SCARA3 | FIGLA |  |  | MOV10 |
| TRMT5 |  |  | SCIN | FIP1L1 |  |  | MOXD1 |
| TRNP1 |  |  | SCO2 | FKBP6 |  |  | MPP2 |
| TSPAN13 |  |  | SCPEP1 | FLG2 |  |  | MRPS15 |
| TSPAN5 |  |  | SDC2 | FLJ13224 |  |  | MSN |
| TSPAN6 |  |  | SDC4 | FLJ25758 |  |  | MSRB1 |
| TSPYL5 |  |  | SEC31B | FLJ34503 |  |  | MSRB2 |
| TUBA4A |  |  | SECTM1 | FLNB |  |  | MSX1 |
| TUBB2A |  |  | SEL1L3 | FLRT3 |  |  | MTCP1 |
| TYRP1 |  |  | SELPLG | FLT3 |  |  | MTFP1 |
| UAP1L1 |  |  | SERPINA1 | FLYWCH2 |  |  | MTMR7 |
| UCHL1 |  |  | SERPINA3 | FMO1 |  |  | MTMR9 |
| UGDH |  |  | SERPINA5 | FMO3 |  |  | MTPAP |
| UGP2 |  |  | SERPINB1 | FMO6P |  |  | MTSS1 |
| ULK1 |  |  | SERPINE1 | FMO9P |  |  | MYADM |
| UNC13C |  |  | SERPINF1 | FMR1NB |  |  | MYCBP2 |
| USP3 |  |  | SERPING1 | FN3K |  |  | MYH7 |
| VAMP1 |  |  | SERTAD1 | FNDC4 |  |  | MYO1E |
| VAT1 |  |  | SERTAD2 | FNDC7 |  |  | MYOM2 |
| VCAN |  |  | SEZ6 | FOLR1 |  |  | MYT1 |
| VIP |  |  | SGMS2 | FOLR3 |  |  | MYT1L |
| VIPR1 |  |  | SH2B3 | FOSB |  |  | MZT1 |
| VSNL1 |  |  | SH3BGRL3 | FOXA1 |  |  | NALCN |
| VSTM2L |  |  | SH3TC1 | FOXA3 |  |  | NAP1L2 |
| WDR47 |  |  | SH3YL1 | FOXB1 |  |  | NAP1L3 |
| WDR5B |  |  | SHC2 | FOXC1 |  |  | NAP1L5 |
| WDR7 |  |  | SHPRH | FOXD2 |  |  | NAV1 |
| WDR90 |  |  | SIGLEC1 | FOXE1 |  |  | NBEA |
| WFDC1 |  |  | SIGLEC10 | FOXF1 |  |  | NDN |
| WIF1 |  |  | SIGLEC11 | FOXF2 |  |  | NDRG2 |
| WNT10B |  |  | SIGLEC9 | FOXG1 |  |  | NDRG3 |
| XK |  |  | SIL1 | FOXJ1 |  |  | NDRG4 |
| XRN2 |  |  | SIPA1 | FOXL2 |  |  | NDUFB8 |
| YJEFN3 |  |  | SIPA1L2 | FOXQ1 |  |  | NDUFS1 |
| YWHAH |  |  | SIRPB2 | FRAS1 |  |  | NECAB2 |
| ZBTB20 |  |  | SKAP2 | FREM1 |  |  | NECAP1 |
| ZBTB25 |  |  | SLA | FRMD5 |  |  | NECAP2 |
| ZCCHC12 |  |  | SLAMF8 | FRMD6 |  |  | NEDD1 |
| ZDHHC8P1 |  |  | SLC11A1 | FRMD6-AS1 |  |  | NEDD9 |
| ZIC1 |  |  | SLC12A7 | FRMD7 |  |  | NEGR1 |
| ZMAT4 |  |  | SLC15A3 | FRS2 |  |  | NEK6 |
| ZNF112 |  |  | SLC15A4 | FSCB |  |  | NES |
| ZNF204P |  |  | SLC16A10 | FSCN1 |  |  | NETO1 |
| ZNF227 |  |  | SLC17A8 | FSCN2 |  |  | NEU4 |
| ZNF266 |  |  | SLC22A18 | FSHB |  |  | NEUROD1 |
| ZNF365 |  |  | SLC22A4 | FSIP2 |  |  | NFE2L3 |
| ZNF385B |  |  | SLC24A3 | FST |  |  | NHSL1 |
| ZNF385D |  |  | SLC25A19 | FSTL3 |  |  | NKAIN4 |
| ZNF501 |  |  | SLC25A20 | FTCDNL1 |  |  | NKIRAS1 |
| ZNF516 |  |  | SLC25A37 | FTH1 |  |  | NLGN1 |
| ZNF559 |  |  | SLC25A41 | FTHL17 |  |  | NLN |
| ZNF606 |  |  | SLC2A3 | FUT5 |  |  | NMB |
| ZNF608 |  |  | SLC2A5 | FXYD4 |  |  | NMNAT1 |
| ZNF667 |  |  | SLC37A2 | FYN |  |  | NMNAT2 |
|  |  |  | SLC38A6 | FZD10 |  |  | NMRAL1 |
|  |  |  | SLC39A14 | FZD3 |  |  | NMT2 |
|  |  |  | SLC39A4 | FZD9 |  |  | NNMT |
|  |  |  | SLC39A8 | GAB1 |  |  | NOG |
|  |  |  | SLC40A1 | GAB4 |  |  | NOL4 |
|  |  |  | SLC44A3 | GABRA6 |  |  | NOTCH2 |
|  |  |  | SLC47A2 | GAGE1 |  |  | NPDC1 |
|  |  |  | SLC7A7 | GAL |  |  | NPM1 |
|  |  |  | SLC9A9 | GAL3ST3 |  |  | NPNT |
|  |  |  | SLCO2B1 | GALC |  |  | NPTXR |
|  |  |  | SLFN11 | GALNT12 |  |  | NR1D2 |
|  |  |  | SLFN12 | GALNT3 |  |  | NR2E1 |
|  |  |  | SLFN5 | GALNT5 |  |  | NRAS |
|  |  |  | SLIT1 | GALR1 |  |  | NRP2 |
|  |  |  | SLITRK2 | GALR2 |  |  | NRSN1 |
|  |  |  | SLN | GALR3 |  |  | NRXN1 |
|  |  |  | SLPI | GAP43 |  |  | NRXN2 |
|  |  |  | SMAGP | GAS2 |  |  | NSG1 |
|  |  |  | SMCO4 | GAS2L2 |  |  | NSUN6 |
|  |  |  | SMIM10 | GAS5 |  |  | NSUN7 |
|  |  |  | SMPDL3A | GAS8 |  |  | NTM |
|  |  |  | SNX9 | GATA6 |  |  | NTN4 |
|  |  |  | SOAT1 | GATM |  |  | NTNG2 |
|  |  |  | SOCS3 | GBX2 |  |  | NTRK3 |
|  |  |  | SOCS6 | GC |  |  | NUCB1 |
|  |  |  | SOD2 | GCG |  |  | NUDT10 |
|  |  |  | SOX1 | GCHFR |  |  | NUDT11 |
|  |  |  | SOX15 | GCK |  |  | NUDT3 |
|  |  |  | SP100 | GCKR |  |  | NUMA1 |
|  |  |  | SP140L | GCNT2 |  |  | NUP37 |
|  |  |  | SPAG1 | GCNT3 |  |  | OAT |
|  |  |  | SPAG4 | GCSAML |  |  | OCIAD2 |
|  |  |  | SPAST | GDF11 |  |  | OLA1 |
|  |  |  | SPATS2 | GDF2 |  |  | OLFM1 |
|  |  |  | SPATS2L | GDF5 |  |  | OLIG2 |
|  |  |  | SPCS3 | GGT1 |  |  | OPCML |
|  |  |  | SPHK1 | GGT6 |  |  | OPLAH |
|  |  |  | SPI1 | GINM1 |  |  | OR2L13 |
|  |  |  | SPIRE1 | GJA1 |  |  | OSBPL10 |
|  |  |  | SPOCD1 | GJA3 |  |  | OSBPL3 |
|  |  |  | SPP1 | GJA8 |  |  | OSMR |
|  |  |  | SPTLC2 | GK2 |  |  | OSTC |
|  |  |  | SQLE | GKN1 |  |  | OTOGL |
|  |  |  | SREBF2 | GLB1L2 |  |  | OTUD7A |
|  |  |  | SRGN | GLCE |  |  | OXTR |
|  |  |  | SRPX2 | GLDC |  |  | P2RX5 |
|  |  |  | SS18L1 | GLG1 |  |  | P2RX7 |
|  |  |  | ST14 | GLI1 |  |  | P2RY1 |
|  |  |  | ST6GAL1 | GLIS1 |  |  | P4HA1 |
|  |  |  | ST6GAL2 | GLRA2 |  |  | P4HB |
|  |  |  | ST8SIA4 | GLRX2 |  |  | PAAF1 |
|  |  |  | STAB1 | GLT8D2 |  |  | PABPC1L |
|  |  |  | STAT5A | GLTPD2 |  |  | PABPC4L |
|  |  |  | STAT6 | GLYATL1 |  |  | PABPC5 |
|  |  |  | STBD1 | GMDS |  |  | PAIP1 |
|  |  |  | STEAP1 | GMPR |  |  | PAK3 |
|  |  |  | STK40 | GNA14 |  |  | PALLD |
|  |  |  | STOM | GNAS-AS1 |  |  | PALM |
|  |  |  | STXBP2 | GNAT2 |  |  | PARVB |
|  |  |  | STXBP3 | GNE |  |  | PBLD |
|  |  |  | SULF1 | GNG8 |  |  | PBX3 |
|  |  |  | SUMF1 | GNGT1 |  |  | PCBP3 |
|  |  |  | SUSD3 | GNMT |  |  | PCCA |
|  |  |  | SWAP70 | GNRH2 |  |  | PCDH15 |
|  |  |  | SYK | GOLGA6L2 |  |  | PCDH18 |
|  |  |  | SYNC | GOLGA8A |  |  | PCDH7 |
|  |  |  | SYNGR2 | GOLIM4 |  |  | PCDHA9 |
|  |  |  | SYNM | GOLM1 |  |  | PCGF2 |
|  |  |  | SYT11 | GOLT1A |  |  | PCOLCE2 |
|  |  |  | SYT17 | GP9 |  |  | PCSK1N |
|  |  |  | SYTL3 | GPA33 |  |  | PCSK2 |
|  |  |  | TACC2 | GPAM |  |  | PCSK5 |
|  |  |  | TAF5 | GPBAR1 |  |  | PDCD4 |
|  |  |  | TAGAP | GPC3 |  |  | PDE2A |
|  |  |  | TAPBPL | GPC6 |  |  | PDE4A |
|  |  |  | TARBP1 | GPD1 |  |  | PDE8B |
|  |  |  | TBC1D19 | GPER1 |  |  | PDGFA |
|  |  |  | TBC1D8B | GPR1 |  |  | PDGFC |
|  |  |  | TBXAS1 | GPR101 |  |  | PDIA4 |
|  |  |  | TC2N | GPR137B |  |  | PDIA6 |
|  |  |  | TCIRG1 | GPR143 |  |  | PDLIM3 |
|  |  |  | TCN2 | GPR171 |  |  | PDLIM4 |
|  |  |  | TEAD4 | GPR18 |  |  | PDPN |
|  |  |  | TEX26 | GPR20 |  |  | PDSS1 |
|  |  |  | TFEC | GPR25 |  |  | PDZD4 |
|  |  |  | TGFB1 | GPR3 |  |  | PDZRN4 |
|  |  |  | TGFBI | GPR32 |  |  | PELI2 |
|  |  |  | TGFBR1 | GPR37L1 |  |  | PELI3 |
|  |  |  | TGFBR2 | GPR45 |  |  | PFKFB4 |
|  |  |  | THAP10 | GPR87 |  |  | PFKM |
|  |  |  | THBD | GPRC5C |  |  | PFN1 |
|  |  |  | THEMIS2 | GPRC6A |  |  | PFN2 |
|  |  |  | THNSL1 | GPSM2 |  |  | PGBD5 |
|  |  |  | TIFA | GPT2 |  |  | PGGT1B |
|  |  |  | TIGD3 | GPX3 |  |  | PGM2 |
|  |  |  | TIPARP | GRAP |  |  | PHACTR3 |
|  |  |  | TLN1 | GRHL1 |  |  | PHKA1 |
|  |  |  | TLR1 | GRIA1 |  |  | PHLPP2 |
|  |  |  | TLR10 | GRIK1 |  |  | PHTF1 |
|  |  |  | TLR2 | GRIN3B |  |  | PHYHIPL |
|  |  |  | TLR3 | GRP |  |  | PIANP |
|  |  |  | TLR4 | GRTP1 |  |  | PID1 |
|  |  |  | TLR5 | GSC2 |  |  | PIEZO1 |
|  |  |  | TLR7 | GSDMB |  |  | PIGZ |
|  |  |  | TLR8 | GSG1L |  |  | PIH1D2 |
|  |  |  | TLX1 | GSK3B |  |  | PIK3CB |
|  |  |  | TM6SF1 | GSTM3 |  |  | PINLYP |
|  |  |  | TM7SF2 | GSTM4 |  |  | PIPOX |
|  |  |  | TMBIM4 | GSTT1 |  |  | PITX1 |
|  |  |  | TMEM106A | GSTZ1 |  |  | PKD2 |
|  |  |  | TMEM107 | GSX1 |  |  | PKDCC |
|  |  |  | TMEM119 | GTF2F2 |  |  | PKIA |
|  |  |  | TMEM140 | GTPBP10 |  |  | PKIB |
|  |  |  | TMEM169 | GTSF1 |  |  | PKN2 |
|  |  |  | TMEM173 | GUCA2A |  |  | PKNOX2 |
|  |  |  | TMEM176A | GUCA2B |  |  | PLA2G5 |
|  |  |  | TMEM176B | GUCY1A2 |  |  | PLAT |
|  |  |  | TMEM181 | GUCY2C |  |  | PLCB1 |
|  |  |  | TMEM185B | GUCY2D |  |  | PLCB4 |
|  |  |  | TMEM220 | GULP1 |  |  | PLCE1 |
|  |  |  | TMEM52B | GYG2 |  |  | PLCH2 |
|  |  |  | TMEM97 | GYS2 |  |  | PLCL2 |
|  |  |  | TMSB10 | GZMB |  |  | PLCXD2 |
|  |  |  | TNFAIP2 | GZMM |  |  | PLEK2 |
|  |  |  | TNFAIP3 | H1F0 |  |  | PLEKHA4 |
|  |  |  | TNFAIP8 | H1FOO |  |  | PLEKHA6 |
|  |  |  | TNFAIP8L2 | H1FX |  |  | PLEKHA8P1 |
|  |  |  | TNFRSF14 | HAAO |  |  | PLEKHF2 |
|  |  |  | TNFRSF1A | HAND2 |  |  | PLEKHG1 |
|  |  |  | TNFRSF1B | HAO1 |  |  | PLEKHG2 |
|  |  |  | TNFSF10 | HAPLN1 |  |  | PLIN3 |
|  |  |  | TNFSF13B | HBB |  |  | PLOD1 |
|  |  |  | TNFSF4 | HBD |  |  | PLOD2 |
|  |  |  | TNIP1 | HBEGF |  |  | PLOD3 |
|  |  |  | TNK2 | HBM |  |  | PLP2 |
|  |  |  | TNKS | HBZ |  |  | PLXNB2 |
|  |  |  | TNRC6B | HCAR1 |  |  | PMM2 |
|  |  |  | TNRC6C | HCG26 |  |  | PNKP |
|  |  |  | TOX3 | HCG27 |  |  | PNMA1 |
|  |  |  | TPP1 | HCG4 |  |  | PNMA2 |
|  |  |  | TPPP3 | HCRTR1 |  |  | PNMA3 |
|  |  |  | TPRG1 | HDDC3 |  |  | PNMA6A |
|  |  |  | TPST1 | HEATR3 |  |  | PNP |
|  |  |  | TPST2 | HEATR4 |  |  | PODXL2 |
|  |  |  | TRAC | HECTD2 |  |  | POLR2F |
|  |  |  | TRAF3IP2-AS1 | HEPACAM2 |  |  | POLR2L |
|  |  |  | TREM1 | HES4 |  |  | POLR3A |
|  |  |  | TREM2 | HEXA-AS1 |  |  | POR |
|  |  |  | TREML1 | HEY2 |  |  | POU6F1 |
|  |  |  | TRIB1 | HGFAC |  |  | PPA1 |
|  |  |  | TRIM38 | HHAT |  |  | PPCDC |
|  |  |  | TRIM6 | HHIP-AS1 |  |  | PPDPF |
|  |  |  | TRIP4 | HHLA3 |  |  | PPFIA2 |
|  |  |  | TRO | HIBADH |  |  | PPFIA3 |
|  |  |  | TRPM4 | HIF3A |  |  | PPIB |
|  |  |  | TRPM8 | HIGD1B |  |  | PPIC |
|  |  |  | TRPV6 | HILS1 |  |  | PPIF |
|  |  |  | TSC2 | HIP1 |  |  | PPIL6 |
|  |  |  | TSHZ1 | HIRA |  |  | PPM1E |
|  |  |  | TSPO | HIST1H1A |  |  | PPP1R13B |
|  |  |  | TTC12 | HIST1H1B |  |  | PPP1R1A |
|  |  |  | TTC38 | HIST1H2BA |  |  | PPP1R3E |
|  |  |  | TUBA3FP | HIST1H2BL |  |  | PPP1R9A |
|  |  |  | TUBE1 | HIST1H2BM |  |  | PPP2R2B |
|  |  |  | TWIST1 | HIST1H2BO |  |  | PPP2R2D |
|  |  |  | TXLNB | HIST1H3A |  |  | PPP3CB |
|  |  |  | TXN | HIST1H3G |  |  | PPP4C |
|  |  |  | TYMP | HIST1H3I |  |  | PRDX4 |
|  |  |  | TYROBP | HIST1H4E |  |  | PRICKLE1 |
|  |  |  | UAP1 | HIST1H4H |  |  | PRKACB |
|  |  |  | UCP2 | HIST1H4L |  |  | PRKAR2B |
|  |  |  | UGCG | HIST2H2BE |  |  | PRKCE |
|  |  |  | UNC93B1 | HIST3H3 |  |  | PRKCZ |
|  |  |  | VAMP3 | HKDC1 |  |  | PRKD2 |
|  |  |  | VAMP5 | HMGA1 |  |  | PRPF31 |
|  |  |  | VAMP8 | HMGB4 |  |  | PRPS2 |
|  |  |  | VASN | HOMER2 |  |  | PRRT2 |
|  |  |  | VASP | HOPX |  |  | PSD |
|  |  |  | VAV1 | HORMAD1 |  |  | PSD2 |
|  |  |  | VCAM1 | HOXA13 |  |  | PSENEN |
|  |  |  | VCL | HOXA6 |  |  | PSRC1 |
|  |  |  | VMO1 | HOXB4 |  |  | PSTK |
|  |  |  | VMP1 | HOXB6 |  |  | PTBP2 |
|  |  |  | VNN1 | HOXC5 |  |  | PTGFRN |
|  |  |  | VNN2 | HOXC8 |  |  | PTPN12 |
|  |  |  | VPS51 | HPD |  |  | PTPN13 |
|  |  |  | VRK2 | HPDL |  |  | PTPN14 |
|  |  |  | VSIG4 | HRC |  |  | PTPN4 |
|  |  |  | WARS | HRCT1 |  |  | PTPRB |
|  |  |  | WDR63 | HS3ST5 |  |  | PTPRN |
|  |  |  | WDR86 | HS3ST6 |  |  | PTPRN2 |
|  |  |  | WIPF1 | HS6ST2 |  |  | PTPRT |
|  |  |  | WNT7B | HSBP1L1 |  |  | PTX3 |
|  |  |  | XKR6 | HSD17B2 |  |  | PUSL1 |
|  |  |  | XPO7 | HSD17B3 |  |  | PYGL |
|  |  |  | YAP1 | HSF5 |  |  | R3HDM2 |
|  |  |  | ZCCHC18 | HSPA13 |  |  | RAB11FIP2 |
|  |  |  | ZFP2 | HSPA4L |  |  | RAB11FIP4 |
|  |  |  | ZFP36 | HSPB2 |  |  | RAB18 |
|  |  |  | ZFP36L1 | HSPB9 |  |  | RAB26 |
|  |  |  | ZNF239 | HTATSF1P2 |  |  | RAB27B |
|  |  |  | ZNF287 | HTN3 |  |  | RAB32 |
|  |  |  | ZNF436 | HTR1A |  |  | RAB33A |
|  |  |  | ZNF708 | HTR1B |  |  | RAB36 |
|  |  |  | ZNF711 | HTR1D |  |  | RAB39B |
|  |  |  | ZNF74 | HYAL1 |  |  | RAB3A |
|  |  |  | ZNRF2 | HYAL3 |  |  | RAB3C |
|  |  |  | ZSWIM5 | HYDIN |  |  | RAB3IP |
|  |  |  |  | HYMAI |  |  | RAI14 |
|  |  |  |  | ICAM2 |  |  | RAI2 |
|  |  |  |  | ICAM4 |  |  | RALGPS1 |
|  |  |  |  | ID1 |  |  | RALYL |
|  |  |  |  | ID2 |  |  | RANBP17 |
|  |  |  |  | ID4 |  |  | RANBP6 |
|  |  |  |  | IDI2 |  |  | RAP1GAP |
|  |  |  |  | IDNK |  |  | RAP2A |
|  |  |  |  | IDO1 |  |  | RAPGEF2 |
|  |  |  |  | IFIT2 |  |  | RAPGEF4 |
|  |  |  |  | IFNA1 |  |  | RAPGEFL1 |
|  |  |  |  | IFNA2 |  |  | RASSF1 |
|  |  |  |  | IFNA21 |  |  | RBFOX2 |
|  |  |  |  | IFNA8 |  |  | RBMS1 |
|  |  |  |  | IFNG |  |  | RBP1 |
|  |  |  |  | IFNK |  |  | RBPJ |
|  |  |  |  | IFNW1 |  |  | RCAN1 |
|  |  |  |  | IFT74 |  |  | RCBTB1 |
|  |  |  |  | IFT80 |  |  | RCOR2 |
|  |  |  |  | IFT88 |  |  | RDH10 |
|  |  |  |  | IGF1 |  |  | RDH5 |
|  |  |  |  | IGFBP1 |  |  | RECQL |
|  |  |  |  | IGFBP6 |  |  | REEP1 |
|  |  |  |  | IGFN1 |  |  | REEP2 |
|  |  |  |  | IGHV1-69 |  |  | REPS2 |
|  |  |  |  | IGHV3-54 |  |  | RFTN2 |
|  |  |  |  | IGLL1 |  |  | RFX2 |
|  |  |  |  | IGLL3P |  |  | RFX4 |
|  |  |  |  | IGSF11 |  |  | RFXANK |
|  |  |  |  | IGSF5 |  |  | RGCC |
|  |  |  |  | IGSF9B |  |  | RGS11 |
|  |  |  |  | IL12A |  |  | RGS7 |
|  |  |  |  | IL17B |  |  | RGS7BP |
|  |  |  |  | IL17F |  |  | RHBDL1 |
|  |  |  |  | IL17RB |  |  | RHOBTB2 |
|  |  |  |  | IL18R1 |  |  | RHOC |
|  |  |  |  | IL18RAP |  |  | RHOJ |
|  |  |  |  | IL1RL2 |  |  | RHPN2 |
|  |  |  |  | IL2 |  |  | RICTOR |
|  |  |  |  | IL21 |  |  | RIIAD1 |
|  |  |  |  | IL22RA2 |  |  | RIMS1 |
|  |  |  |  | IL23A |  |  | RIMS2 |
|  |  |  |  | IL24 |  |  | RIPPLY2 |
|  |  |  |  | IL26 |  |  | RIT1 |
|  |  |  |  | IL27 |  |  | RIT2 |
|  |  |  |  | IL32 |  |  | RMDN1 |
|  |  |  |  | IL33 |  |  | RNASE4 |
|  |  |  |  | IL5 |  |  | RND1 |
|  |  |  |  | IL6ST |  |  | RNF112 |
|  |  |  |  | IL9 |  |  | RNF144A |
|  |  |  |  | IMMP1L |  |  | RNF146 |
|  |  |  |  | IMMP2L |  |  | RNF150 |
|  |  |  |  | IMPDH1 |  |  | RNF165 |
|  |  |  |  | INE1 |  |  | RNF41 |
|  |  |  |  | INGX |  |  | RNFT1 |
|  |  |  |  | INHBA |  |  | ROR1 |
|  |  |  |  | INHBE |  |  | RPE65 |
|  |  |  |  | INMT |  |  | RPGR |
|  |  |  |  | INO80B |  |  | RPN1 |
|  |  |  |  | INPP4B |  |  | RPN2 |
|  |  |  |  | INSIG1 |  |  | RPRM |
|  |  |  |  | INSIG2 |  |  | RPS27L |
|  |  |  |  | INTS2 |  |  | RPS6KA5 |
|  |  |  |  | INTU |  |  | RPS6KL1 |
|  |  |  |  | INVS |  |  | RRAS |
|  |  |  |  | IPO11 |  |  | RRBP1 |
|  |  |  |  | IQCA1 |  |  | RSF1 |
|  |  |  |  | IQCC |  |  | RTKN |
|  |  |  |  | IQCF2 |  |  | RTN1 |
|  |  |  |  | IQCF3 |  |  | RTN2 |
|  |  |  |  | IQCF5 |  |  | RTN3 |
|  |  |  |  | IRF2BP2 |  |  | RTN4RL2 |
|  |  |  |  | IRGC |  |  | RUFY3 |
|  |  |  |  | IRGM |  |  | RUNDC3A |
|  |  |  |  | IRS1 |  |  | RUNDC3B |
|  |  |  |  | IRS2 |  |  | RUNX1T1 |
|  |  |  |  | IRX1 |  |  | RUSC2 |
|  |  |  |  | IRX2 |  |  | S100A13 |
|  |  |  |  | IRX3 |  |  | S100A16 |
|  |  |  |  | IRX4 |  |  | S100A2 |
|  |  |  |  | ISL1 |  |  | SALL1 |
|  |  |  |  | ISM1 |  |  | SALL2 |
|  |  |  |  | ISOC1 |  |  | SAP18 |
|  |  |  |  | ITFG2 |  |  | SAP30 |
|  |  |  |  | ITGA10 |  |  | SATB1 |
|  |  |  |  | ITGA6 |  |  | SBF1 |
|  |  |  |  | ITGA8 |  |  | SBNO2 |
|  |  |  |  | ITGB1BP2 |  |  | SC5D |
|  |  |  |  | ITGB7 |  |  | SCAI |
|  |  |  |  | ITIH2 |  |  | SCAMP1 |
|  |  |  |  | ITIH3 |  |  | SCAMP5 |
|  |  |  |  | ITIH5 |  |  | SCAPER |
|  |  |  |  | ITM2A |  |  | SCD5 |
|  |  |  |  | ITM2C |  |  | SCG3 |
|  |  |  |  | ITPR2 |  |  | SCN1A |
|  |  |  |  | IVD |  |  | SCN2A |
|  |  |  |  | IYD |  |  | SCN3A |
|  |  |  |  | IZUMO1 |  |  | SCN3B |
|  |  |  |  | IZUMO2 |  |  | SCRT1 |
|  |  |  |  | JAK1 |  |  | SDC1 |
|  |  |  |  | JAM3 |  |  | SDF2L1 |
|  |  |  |  | JSRP1 |  |  | SDF4 |
|  |  |  |  | JUP |  |  | SDSL |
|  |  |  |  | KAAG1 |  |  | SEC61A1 |
|  |  |  |  | KANK1 |  |  | SEC61A2 |
|  |  |  |  | KANK3 |  |  | SEC61G |
|  |  |  |  | KANSL1L |  |  | SECISBP2L |
|  |  |  |  | KAT2B |  |  | SEMA3G |
|  |  |  |  | KBTBD12 |  |  | SEPHS1 |
|  |  |  |  | KCNA5 |  |  | SEPTIN10 |
|  |  |  |  | KCNA6 |  |  | SEPTIN3 |
|  |  |  |  | KCND1 |  |  | SEPTIN9 |
|  |  |  |  | KCNF1 |  |  | SERINC1 |
|  |  |  |  | KCNG1 |  |  | SERP2 |
|  |  |  |  | KCNH1 |  |  | SERPINB6 |
|  |  |  |  | KCNH2 |  |  | SERTAD3 |
|  |  |  |  | KCNH4 |  |  | SESN2 |
|  |  |  |  | KCNIP1 |  |  | SESTD1 |
|  |  |  |  | KCNJ13 |  |  | SETD9 |
|  |  |  |  | KCNJ16 |  |  | SEZ6L |
|  |  |  |  | KCNJ2-AS1 |  |  | SEZ6L2 |
|  |  |  |  | KCNK13 |  |  | SFR1 |
|  |  |  |  | KCNK2 |  |  | SFRP4 |
|  |  |  |  | KCNK9 |  |  | SGIP1 |
|  |  |  |  | KCNQ1DN |  |  | SGMS1 |
|  |  |  |  | KCNRG |  |  | SGSM1 |
|  |  |  |  | KCNS3 |  |  | SH2D3C |
|  |  |  |  | KCNT2 |  |  | SH2D4A |
|  |  |  |  | KCNV2 |  |  | SH3BGRL2 |
|  |  |  |  | KDM5A |  |  | SH3GL2 |
|  |  |  |  | KDM5B |  |  | SH3GLB1 |
|  |  |  |  | KDM5D |  |  | SH3PXD2B |
|  |  |  |  | KEL |  |  | SHANK2 |
|  |  |  |  | KERA |  |  | SHANK3 |
|  |  |  |  | KHDC3L |  |  | SHC3 |
|  |  |  |  | KIAA0895 |  |  | SHD |
|  |  |  |  | KIAA1522 |  |  | SHISA4 |
|  |  |  |  | KIAA1614 |  |  | SHISA5 |
|  |  |  |  | KIF12 |  |  | SHKBP1 |
|  |  |  |  | KIF21A |  |  | SHOX2 |
|  |  |  |  | KIF26B |  |  | SHROOM3 |
|  |  |  |  | KIF2B |  |  | SIK3 |
|  |  |  |  | KIF7 |  |  | SIPA1L1 |
|  |  |  |  | KIR2DL3 |  |  | SIRT1 |
|  |  |  |  | KIR3DL1 |  |  | SIX5 |
|  |  |  |  | KISS1R |  |  | SLC10A3 |
|  |  |  |  | KITLG |  |  | SLC16A1 |
|  |  |  |  | KL |  |  | SLC16A14 |
|  |  |  |  | KLF16 |  |  | SLC16A4 |
|  |  |  |  | KLF4 |  |  | SLC16A7 |
|  |  |  |  | KLF5 |  |  | SLC16A9 |
|  |  |  |  | KLF8 |  |  | SLC1A1 |
|  |  |  |  | KLF9 |  |  | SLC1A5 |
|  |  |  |  | KLHDC10 |  |  | SLC1A6 |
|  |  |  |  | KLHL11 |  |  | SLC20A1 |
|  |  |  |  | KLHL13 |  |  | SLC22A17 |
|  |  |  |  | KLHL21 |  |  | SLC25A24 |
|  |  |  |  | KLHL25 |  |  | SLC25A27 |
|  |  |  |  | KLHL29 |  |  | SLC25A43 |
|  |  |  |  | KLHL8 |  |  | SLC26A2 |
|  |  |  |  | KLHL9 |  |  | SLC27A3 |
|  |  |  |  | KLK11 |  |  | SLC2A10 |
|  |  |  |  | KLKB1 |  |  | SLC2A13 |
|  |  |  |  | KLRF1 |  |  | SLC30A10 |
|  |  |  |  | KMT2E |  |  | SLC35D2 |
|  |  |  |  | KNCN |  |  | SLC35F5 |
|  |  |  |  | KPNA3 |  |  | SLC35G2 |
|  |  |  |  | KRBA2 |  |  | SLC38A1 |
|  |  |  |  | KREMEN2 |  |  | SLC39A1 |
|  |  |  |  | KRT13 |  |  | SLC3A2 |
|  |  |  |  | KRT14 |  |  | SLC43A3 |
|  |  |  |  | KRT16 |  |  | SLC44A1 |
|  |  |  |  | KRT18 |  |  | SLC4A2 |
|  |  |  |  | KRT19 |  |  | SLC4A7 |
|  |  |  |  | KRT20 |  |  | SLC52A2 |
|  |  |  |  | KRT23 |  |  | SLC6A1 |
|  |  |  |  | KRT27 |  |  | SLC8A3 |
|  |  |  |  | KRT3 |  |  | SLC9A6 |
|  |  |  |  | KRT32 |  |  | SLITRK1 |
|  |  |  |  | KRT35 |  |  | SLITRK5 |
|  |  |  |  | KRT5 |  |  | SMAD7 |
|  |  |  |  | KRT72 |  |  | SMAD9 |
|  |  |  |  | KRT75 |  |  | SMC5 |
|  |  |  |  | KRT81 |  |  | SMIM3 |
|  |  |  |  | KRT85 |  |  | SMO |
|  |  |  |  | KRTAP1-3 |  |  | SMOC1 |
|  |  |  |  | KRTAP11-1 |  |  | SMPD3 |
|  |  |  |  | KRTAP3-3 |  |  | SNAP25 |
|  |  |  |  | KRTAP4-1 |  |  | SNAP91 |
|  |  |  |  | KRTAP4-11 |  |  | SNAPC1 |
|  |  |  |  | KRTAP4-4 |  |  | SNAPC3 |
|  |  |  |  | KRTAP9-4 |  |  | SNCB |
|  |  |  |  | KRTCAP3 |  |  | SNN |
|  |  |  |  | KSR2 |  |  | SNTB1 |
|  |  |  |  | LAG3 |  |  | SNX22 |
|  |  |  |  | LALBA |  |  | SNX32 |
|  |  |  |  | LAMA1 |  |  | SNX7 |
|  |  |  |  | LAMB3 |  |  | SNX8 |
|  |  |  |  | LANCL2 |  |  | SOBP |
|  |  |  |  | LAYN |  |  | SOCS2 |
|  |  |  |  | LCE2B |  |  | SORCS3 |
|  |  |  |  | LCN1 |  |  | SOWAHC |
|  |  |  |  | LCN15 |  |  | SOX6 |
|  |  |  |  | LCN2 |  |  | SOX8 |
|  |  |  |  | LDHAL6A |  |  | SOX9 |
|  |  |  |  | LDHC |  |  | SPATA17 |
|  |  |  |  | LDLR |  |  | SPATA6 |
|  |  |  |  | LEAP2 |  |  | SPHKAP |
|  |  |  |  | LEMD1 |  |  | SPOCK1 |
|  |  |  |  | LENG8 |  |  | SPPL2A |
|  |  |  |  | LENG9 |  |  | SPRED1 |
|  |  |  |  | LEP |  |  | SPRY2 |
|  |  |  |  | LEPR |  |  | SPTAN1 |
|  |  |  |  | LGALS13 |  |  | SPTBN1 |
|  |  |  |  | LGALS2 |  |  | SPTBN2 |
|  |  |  |  | LGALSL |  |  | SRGAP3 |
|  |  |  |  | LGR4 |  |  | SRPX |
|  |  |  |  | LGR6 |  |  | SRRM3 |
|  |  |  |  | LGSN |  |  | SRRM4 |
|  |  |  |  | LHCGR |  |  | SSR3 |
|  |  |  |  | LHFPL5 |  |  | SSTR1 |
|  |  |  |  | LHX1 |  |  | SSTR2 |
|  |  |  |  | LHX8 |  |  | ST3GAL5 |
|  |  |  |  | LHX9 |  |  | ST5 |
|  |  |  |  | LIAS |  |  | ST8SIA3 |
|  |  |  |  | LIFR |  |  | STAC |
|  |  |  |  | LIG4 |  |  | STAM |
|  |  |  |  | LILRA4 |  |  | STAMBPL1 |
|  |  |  |  | LILRB5 |  |  | STAR |
|  |  |  |  | LIMD2 |  |  | STARD10 |
|  |  |  |  | LIMS2 |  |  | STAU2 |
|  |  |  |  | LIN28A |  |  | STEAP3 |
|  |  |  |  | LIN28B |  |  | STK17A |
|  |  |  |  | LIN54 |  |  | STK3 |
|  |  |  |  | LIN7A |  |  | STK32C |
|  |  |  |  | LINC00052 |  |  | STMN2 |
|  |  |  |  | LINC00242 |  |  | STMN3 |
|  |  |  |  | LINC00290 |  |  | STMN4 |
|  |  |  |  | LINC00305 |  |  | STOX2 |
|  |  |  |  | LINC00312 |  |  | STRBP |
|  |  |  |  | LINC00314 |  |  | STRIP2 |
|  |  |  |  | LINC00319 |  |  | STXBP1 |
|  |  |  |  | LINC00460 |  |  | SUCLG2 |
|  |  |  |  | LINC00471 |  |  | SUPT3H |
|  |  |  |  | LINC00472 |  |  | SUSD4 |
|  |  |  |  | LINC00479 |  |  | SUSD5 |
|  |  |  |  | LINC00482 |  |  | SV2A |
|  |  |  |  | LINC00487 |  |  | SVOP |
|  |  |  |  | LINC00488 |  |  | SYBU |
|  |  |  |  | LINC00545 |  |  | SYCE1 |
|  |  |  |  | LINC00597 |  |  | SYN1 |
|  |  |  |  | LINC00619 |  |  | SYN3 |
|  |  |  |  | LINC00652 |  |  | SYNE1 |
|  |  |  |  | LINC00656 |  |  | SYNGR1 |
|  |  |  |  | LINC00668 |  |  | SYNGR3 |
|  |  |  |  | LINC00687 |  |  | SYNJ1 |
|  |  |  |  | LINC00691 |  |  | SYPL1 |
|  |  |  |  | LINC00955 |  |  | SYT14 |
|  |  |  |  | LINC01098 |  |  | SYT4 |
|  |  |  |  | LINC01114 |  |  | SYT9 |
|  |  |  |  | LINC01116 |  |  | TAF12 |
|  |  |  |  | LINC01139 |  |  | TAF3 |
|  |  |  |  | LINC01242 |  |  | TAGLN2 |
|  |  |  |  | LINC01341 |  |  | TAGLN3 |
|  |  |  |  | LIPF |  |  | TAOK3 |
|  |  |  |  | LIPT1 |  |  | TAPT1 |
|  |  |  |  | LMBR1 |  |  | TAPT1-AS1 |
|  |  |  |  | LMF1 |  |  | TARSL2 |
|  |  |  |  | LMO7DN |  |  | TBC1D24 |
|  |  |  |  | LMOD1 |  |  | TBC1D30 |
|  |  |  |  | LMX1A |  |  | TBC1D9 |
|  |  |  |  | LMX1B |  |  | TBPL1 |
|  |  |  |  | LOC285095 |  |  | TBX15 |
|  |  |  |  | LOC285191 |  |  | TBX2 |
|  |  |  |  | LOC441052 |  |  | TCEA3 |
|  |  |  |  | LOC441601 |  |  | TCEAL2 |
|  |  |  |  | LOC613266 |  |  | TCTN1 |
|  |  |  |  | LOC644090 |  |  | TDRD6 |
|  |  |  |  | LOC645188 |  |  | TDRD9 |
|  |  |  |  | LOC645261 |  |  | TEAD2 |
|  |  |  |  | LOC645485 |  |  | TEAD3 |
|  |  |  |  | LOC646903 |  |  | TEF |
|  |  |  |  | LOC653160 |  |  | TEK |
|  |  |  |  | LOC728485 |  |  | TEP1 |
|  |  |  |  | LOC728613 |  |  | TERF2IP |
|  |  |  |  | LOC729652 |  |  | TFPI |
|  |  |  |  | LOC729970 |  |  | TFRC |
|  |  |  |  | LOC730202 |  |  | TGFB1I1 |
|  |  |  |  | LPA |  |  | TGFB2 |
|  |  |  |  | LPAR4 |  |  | TGIF1 |
|  |  |  |  | LPIN3 |  |  | TGIF2 |
|  |  |  |  | LPL |  |  | THBS3 |
|  |  |  |  | LPO |  |  | THRA |
|  |  |  |  | LRG1 |  |  | TIMP1 |
|  |  |  |  | LRIF1 |  |  | TM9SF1 |
|  |  |  |  | LRIG1 |  |  | TMCC1 |
|  |  |  |  | LRP12 |  |  | TMCO4 |
|  |  |  |  | LRP2BP |  |  | TMED2 |
|  |  |  |  | LRP5L |  |  | TMED4 |
|  |  |  |  | LRP6 |  |  | TMED9 |
|  |  |  |  | LRRC18 |  |  | TMEFF2 |
|  |  |  |  | LRRC19 |  |  | TMEM100 |
|  |  |  |  | LRRC2 |  |  | TMEM108 |
|  |  |  |  | LRRC31 |  |  | TMEM132B |
|  |  |  |  | LRRC34 |  |  | TMEM132C |
|  |  |  |  | LRRC36 |  |  | TMEM145 |
|  |  |  |  | LRRC37A3 |  |  | TMEM147 |
|  |  |  |  | LRRC37A5P |  |  | TMEM151B |
|  |  |  |  | LRRC39 |  |  | TMEM154 |
|  |  |  |  | LRRC45 |  |  | TMEM158 |
|  |  |  |  | LRRC4B |  |  | TMEM168 |
|  |  |  |  | LRRC6 |  |  | TMEM196 |
|  |  |  |  | LRRC61 |  |  | TMEM209 |
|  |  |  |  | LRRC75A |  |  | TMEM246 |
|  |  |  |  | LRRK1 |  |  | TMEM248 |
|  |  |  |  | LRRK2 |  |  | TMEM51 |
|  |  |  |  | LRRN2 |  |  | TMEM54 |
|  |  |  |  | LRRN3 |  |  | TMEM59L |
|  |  |  |  | LTB4R2 |  |  | TMEM63C |
|  |  |  |  | LTBP4 |  |  | TMEM64 |
|  |  |  |  | LURAP1L |  |  | TMEM67 |
|  |  |  |  | LUZP4 |  |  | TMEM71 |
|  |  |  |  | LY6G5C |  |  | TMEM74B |
|  |  |  |  | LY6G6E |  |  | TMEM8A |
|  |  |  |  | LYG1 |  |  | TMOD2 |
|  |  |  |  | LYG2 |  |  | TMUB1 |
|  |  |  |  | LYPD5 |  |  | TNC |
|  |  |  |  | LYPD6B |  |  | TNFAIP6 |
|  |  |  |  | LYZL6 |  |  | TNFRSF10B |
|  |  |  |  | LZTFL1 |  |  | TNFRSF11B |
|  |  |  |  | LZTS1 |  |  | TNFRSF12A |
|  |  |  |  | MAB21L3 |  |  | TNFRSF19 |
|  |  |  |  | MACROD1 |  |  | TNKS2 |
|  |  |  |  | MAEL |  |  | TNNT1 |
|  |  |  |  | MAFG |  |  | TNPO1 |
|  |  |  |  | MAGEA1 |  |  | TNR |
|  |  |  |  | MAGEA11 |  |  | TOM1L1 |
|  |  |  |  | MAGEA12 |  |  | TOX |
|  |  |  |  | MAGEA4 |  |  | TP53I13 |
|  |  |  |  | MAGEB1 |  |  | TP53RK |
|  |  |  |  | MAGEB18 |  |  | TP73-AS1 |
|  |  |  |  | MAGEB3 |  |  | TPD52 |
|  |  |  |  | MAGEB6 |  |  | TPI1 |
|  |  |  |  | MAGEC1 |  |  | TPTE2P6 |
|  |  |  |  | MAGEC2 |  |  | TRAF5 |
|  |  |  |  | MAGEC3 |  |  | TRAM1 |
|  |  |  |  | MAGEE2 |  |  | TRAM1L1 |
|  |  |  |  | MAGI2 |  |  | TRAM2 |
|  |  |  |  | MALAT1 |  |  | TRAPPC6B |
|  |  |  |  | MALL |  |  | TRIB3 |
|  |  |  |  | MALRD1 |  |  | TRIM13 |
|  |  |  |  | MAMDC2 |  |  | TRIM2 |
|  |  |  |  | MAMDC4 |  |  | TRIM23 |
|  |  |  |  | MANEAL |  |  | TRIM47 |
|  |  |  |  | MANSC1 |  |  | TRIM56 |
|  |  |  |  | MAP1B |  |  | TRIM67 |
|  |  |  |  | MAP3K19 |  |  | TRIM8 |
|  |  |  |  | MAPK4 |  |  | TRIO |
|  |  |  |  | MAPK8IP1 |  |  | TRIP10 |
|  |  |  |  | MARCKS |  |  | TRIP6 |
|  |  |  |  | MARK2 |  |  | TRPC1 |
|  |  |  |  | MARVELD3 |  |  | TSKU |
|  |  |  |  | MASP1 |  |  | TSPAN4 |
|  |  |  |  | MAT2A |  |  | TSPAN7 |
|  |  |  |  | MATN3 |  |  | TSPYL1 |
|  |  |  |  | MAZ |  |  | TSPYL2 |
|  |  |  |  | MBD6 |  |  | TSPYL4 |
|  |  |  |  | MBL2 |  |  | TTC23 |
|  |  |  |  | MC3R |  |  | TTC26 |
|  |  |  |  | MC5R |  |  | TTC30B |
|  |  |  |  | MCEMP1 |  |  | TTC33 |
|  |  |  |  | MCHR1 |  |  | TTC7B |
|  |  |  |  | MCOLN1 |  |  | TTC9 |
|  |  |  |  | MCOLN3 |  |  | TTC9B |
|  |  |  |  | MDFI |  |  | TTF2 |
|  |  |  |  | MDM2 |  |  | TUB |
|  |  |  |  | MDM4 |  |  | TUBB6 |
|  |  |  |  | MED13L |  |  | TUBG2 |
|  |  |  |  | MED24 |  |  | TULP3 |
|  |  |  |  | MED29 |  |  | TUSC3 |
|  |  |  |  | MEDAG |  |  | TWSG1 |
|  |  |  |  | MEFV |  |  | TXLNA |
|  |  |  |  | MEGF6 |  |  | TXNDC16 |
|  |  |  |  | MEGF8 |  |  | UBALD1 |
|  |  |  |  | MEIG1 |  |  | UBAP1L |
|  |  |  |  | MEIS1 |  |  | UBE2E2 |
|  |  |  |  | MEIS2 |  |  | UBE2QL1 |
|  |  |  |  | MEIS3 |  |  | UBL3 |
|  |  |  |  | MEOX1 |  |  | UBR3 |
|  |  |  |  | MET |  |  | UNC119 |
|  |  |  |  | METRN |  |  | UNC13A |
|  |  |  |  | METTL25 |  |  | UNC5A |
|  |  |  |  | MFAP5 |  |  | UNC79 |
|  |  |  |  | MFGE8 |  |  | UPF2 |
|  |  |  |  | MFSD2A |  |  | UPP1 |
|  |  |  |  | MFSD3 |  |  | UROS |
|  |  |  |  | MGA |  |  | USP11 |
|  |  |  |  | MGAM |  |  | USP27X |
|  |  |  |  | MIA |  |  | USP51 |
|  |  |  |  | MICAL1 |  |  | UST |
|  |  |  |  | MICALCL |  |  | UTP15 |
|  |  |  |  | MIF |  |  | VAMP2 |
|  |  |  |  | MINPP1 |  |  | VANGL1 |
|  |  |  |  | MIOX |  |  | VAT1L |
|  |  |  |  | MIR205HG |  |  | VAV3 |
|  |  |  |  | MIR646HG |  |  | VAX2 |
|  |  |  |  | MIR663AHG |  |  | VGLL4 |
|  |  |  |  | MIRLET7BHG |  |  | VIM |
|  |  |  |  | MISP |  |  | VIPR2 |
|  |  |  |  | MKLN1 |  |  | VKORC1 |
|  |  |  |  | MKRN3 |  |  | VPS13A |
|  |  |  |  | MLC1 |  |  | VPS36 |
|  |  |  |  | MLF1 |  |  | VSTM2A |
|  |  |  |  | MLH3 |  |  | WAC |
|  |  |  |  | MLIP |  |  | WASF1 |
|  |  |  |  | MLNR |  |  | WASF2 |
|  |  |  |  | MME |  |  | WASF3 |
|  |  |  |  | MMP1 |  |  | WDFY3-AS2 |
|  |  |  |  | MMP10 |  |  | WDR1 |
|  |  |  |  | MMP12 |  |  | WDR17 |
|  |  |  |  | MMP13 |  |  | WDR37 |
|  |  |  |  | MMP15 |  |  | WDR41 |
|  |  |  |  | MMP16 |  |  | WDR78 |
|  |  |  |  | MMP20 |  |  | WIPI1 |
|  |  |  |  | MMP21 |  |  | WLS |
|  |  |  |  | MMP3 |  |  | WNT5A |
|  |  |  |  | MNX1 |  |  | WSCD2 |
|  |  |  |  | MOGAT3 |  |  | WWTR1 |
|  |  |  |  | MOK |  |  | XKR4 |
|  |  |  |  | MORC1 |  |  | XPR1 |
|  |  |  |  | MORN3 |  |  | YBX3 |
|  |  |  |  | MORN5 |  |  | YES1 |
|  |  |  |  | MPDU1 |  |  | YKT6 |
|  |  |  |  | MPDZ |  |  | YPEL1 |
|  |  |  |  | MPND |  |  | YPEL3 |
|  |  |  |  | MPP6 |  |  | YPEL4 |
|  |  |  |  | MRGPRX2 |  |  | ZBTB18 |
|  |  |  |  | MRGPRX3 |  |  | ZBTB44 |
|  |  |  |  | MROH2A |  |  | ZC2HC1A |
|  |  |  |  | MROH9 |  |  | ZC3H12B |
|  |  |  |  | MRPL38 |  |  | ZC3HAV1 |
|  |  |  |  | MRPL50 |  |  | ZC3HAV1L |
|  |  |  |  | MRPS33 |  |  | ZC4H2 |
|  |  |  |  | MRPS6 |  |  | ZCCHC24 |
|  |  |  |  | MS4A5 |  |  | ZDHHC1 |
|  |  |  |  | MS4A8 |  |  | ZDHHC11 |
|  |  |  |  | MSANTD2 |  |  | ZDHHC12 |
|  |  |  |  | MSI1 |  |  | ZDHHC21 |
|  |  |  |  | MSI2 |  |  | ZDHHC22 |
|  |  |  |  | MSLN |  |  | ZFAND1 |
|  |  |  |  | MSMB |  |  | ZFP36L2 |
|  |  |  |  | MSRB3 |  |  | ZFPM2 |
|  |  |  |  | MSS51 |  |  | ZFR2 |
|  |  |  |  | MST1 |  |  | ZFYVE16 |
|  |  |  |  | MST1R |  |  | ZHX2 |
|  |  |  |  | MSTN |  |  | ZMIZ1 |
|  |  |  |  | MSX2 |  |  | ZMYM1 |
|  |  |  |  | MT4 |  |  | ZMYND11 |
|  |  |  |  | MTA1 |  |  | ZNF134 |
|  |  |  |  | MTA2 |  |  | ZNF155 |
|  |  |  |  | MTHFD1L |  |  | ZNF211 |
|  |  |  |  | MTHFD2L |  |  | ZNF217 |
|  |  |  |  | MTNR1A |  |  | ZNF226 |
|  |  |  |  | MTNR1B |  |  | ZNF248 |
|  |  |  |  | MTPN |  |  | ZNF25 |
|  |  |  |  | MUC15 |  |  | ZNF275 |
|  |  |  |  | MUC20 |  |  | ZNF280B |
|  |  |  |  | MUCL1 |  |  | ZNF292 |
|  |  |  |  | MVD |  |  | ZNF347 |
|  |  |  |  | MYADML2 |  |  | ZNF383 |
|  |  |  |  | MYBPC2 |  |  | ZNF395 |
|  |  |  |  | MYBPH |  |  | ZNF416 |
|  |  |  |  | MYEF2 |  |  | ZNF423 |
|  |  |  |  | MYF5 |  |  | ZNF45 |
|  |  |  |  | MYH1 |  |  | ZNF468 |
|  |  |  |  | MYH11 |  |  | ZNF488 |
|  |  |  |  | MYH13 |  |  | ZNF521 |
|  |  |  |  | MYH16 |  |  | ZNF540 |
|  |  |  |  | MYH3 |  |  | ZNF593 |
|  |  |  |  | MYH4 |  |  | ZNF600 |
|  |  |  |  | MYH6 |  |  | ZNF662 |
|  |  |  |  | MYL1 |  |  | ZNF706 |
|  |  |  |  | MYL2 |  |  | ZNF765 |
|  |  |  |  | MYL3 |  |  | ZNF776 |
|  |  |  |  | MYO5C |  |  | ZNF804A |
|  |  |  |  | MYO6 |  |  | ZNF821 |
|  |  |  |  | MYOZ2 |  |  | ZNF91 |
|  |  |  |  | MYPN |  |  | ZRANB1 |
|  |  |  |  | MYRFL |  |  | ZSCAN31 |
|  |  |  |  | N4BP2L2 |  |  | ZSWIM6 |
|  |  |  |  | NAA40 |  |  | ZYX |
|  |  |  |  | NAALAD2 |  |  |  |
|  |  |  |  | NAALADL2 |  |  |  |
|  |  |  |  | NAB2 |  |  |  |
|  |  |  |  | NADK2 |  |  |  |
|  |  |  |  | NAGS |  |  |  |
|  |  |  |  | NANOS1 |  |  |  |
|  |  |  |  | NANOS3 |  |  |  |
|  |  |  |  | NAPRT |  |  |  |
|  |  |  |  | NAT14 |  |  |  |
|  |  |  |  | NAT2 |  |  |  |
|  |  |  |  | NAV2 |  |  |  |
|  |  |  |  | NBPF4 |  |  |  |
|  |  |  |  | NBR2 |  |  |  |
|  |  |  |  | NCOA3 |  |  |  |
|  |  |  |  | NDNF |  |  |  |
|  |  |  |  | NDP |  |  |  |
|  |  |  |  | NDRG1 |  |  |  |
|  |  |  |  | NDUFA3 |  |  |  |
|  |  |  |  | NEB |  |  |  |
|  |  |  |  | NEK1 |  |  |  |
|  |  |  |  | NEK10 |  |  |  |
|  |  |  |  | NEK11 |  |  |  |
|  |  |  |  | NEURL2 |  |  |  |
|  |  |  |  | NEURL3 |  |  |  |
|  |  |  |  | NEUROG1 |  |  |  |
|  |  |  |  | NEUROG2 |  |  |  |
|  |  |  |  | NEUROG3 |  |  |  |
|  |  |  |  | NEXN-AS1 |  |  |  |
|  |  |  |  | NFAT5 |  |  |  |
|  |  |  |  | NFATC2 |  |  |  |
|  |  |  |  | NFE2 |  |  |  |
|  |  |  |  | NFIA |  |  |  |
|  |  |  |  | NFIB |  |  |  |
|  |  |  |  | NFIC |  |  |  |
|  |  |  |  | NFIX |  |  |  |
|  |  |  |  | NFKBIA |  |  |  |
|  |  |  |  | NFKBIL1 |  |  |  |
|  |  |  |  | NGF |  |  |  |
|  |  |  |  | NGFR |  |  |  |
|  |  |  |  | NHLH2 |  |  |  |
|  |  |  |  | NHS |  |  |  |
|  |  |  |  | NIM1K |  |  |  |
|  |  |  |  | NINL |  |  |  |
|  |  |  |  | NKAPL |  |  |  |
|  |  |  |  | NKAPP1 |  |  |  |
|  |  |  |  | NKD2 |  |  |  |
|  |  |  |  | NKTR |  |  |  |
|  |  |  |  | NKX2-1 |  |  |  |
|  |  |  |  | NKX2-2 |  |  |  |
|  |  |  |  | NKX2-3 |  |  |  |
|  |  |  |  | NKX2-5 |  |  |  |
|  |  |  |  | NKX2-8 |  |  |  |
|  |  |  |  | NLGN4Y |  |  |  |
|  |  |  |  | NLRP13 |  |  |  |
|  |  |  |  | NLRP2 |  |  |  |
|  |  |  |  | NLRP4 |  |  |  |
|  |  |  |  | NLRP6 |  |  |  |
|  |  |  |  | NMBR |  |  |  |
|  |  |  |  | NME5 |  |  |  |
|  |  |  |  | NME7 |  |  |  |
|  |  |  |  | NMRK1 |  |  |  |
|  |  |  |  | NMRK2 |  |  |  |
|  |  |  |  | NOL3 |  |  |  |
|  |  |  |  | NOS2 |  |  |  |
|  |  |  |  | NOS3 |  |  |  |
|  |  |  |  | NOSTRIN |  |  |  |
|  |  |  |  | NOTCH1 |  |  |  |
|  |  |  |  | NOTUM |  |  |  |
|  |  |  |  | NOXO1 |  |  |  |
|  |  |  |  | NPAS1 |  |  |  |
|  |  |  |  | NPAS2 |  |  |  |
|  |  |  |  | NPAS3 |  |  |  |
|  |  |  |  | NPAS4 |  |  |  |
|  |  |  |  | NPB |  |  |  |
|  |  |  |  | NPBWR2 |  |  |  |
|  |  |  |  | NPFFR2 |  |  |  |
|  |  |  |  | NPHP3 |  |  |  |
|  |  |  |  | NPIPB15 |  |  |  |
|  |  |  |  | NPPB |  |  |  |
|  |  |  |  | NPPC |  |  |  |
|  |  |  |  | NPR1 |  |  |  |
|  |  |  |  | NPTX2 |  |  |  |
|  |  |  |  | NPVF |  |  |  |
|  |  |  |  | NPW |  |  |  |
|  |  |  |  | NPY2R |  |  |  |
|  |  |  |  | NQO2 |  |  |  |
|  |  |  |  | NR0B1 |  |  |  |
|  |  |  |  | NR1H3 |  |  |  |
|  |  |  |  | NR4A1 |  |  |  |
|  |  |  |  | NR4A2 |  |  |  |
|  |  |  |  | NR4A3 |  |  |  |
|  |  |  |  | NR5A1 |  |  |  |
|  |  |  |  | NRBF2 |  |  |  |
|  |  |  |  | NRCAM |  |  |  |
|  |  |  |  | NRIP1 |  |  |  |
|  |  |  |  | NRTN |  |  |  |
|  |  |  |  | NSMF |  |  |  |
|  |  |  |  | NSUN5P1 |  |  |  |
|  |  |  |  | NT5E |  |  |  |
|  |  |  |  | NTF3 |  |  |  |
|  |  |  |  | NTN3 |  |  |  |
|  |  |  |  | NTNG1 |  |  |  |
|  |  |  |  | NTS |  |  |  |
|  |  |  |  | NTSR1 |  |  |  |
|  |  |  |  | NUCB2 |  |  |  |
|  |  |  |  | NUCKS1 |  |  |  |
|  |  |  |  | NUDT12 |  |  |  |
|  |  |  |  | NUDT13 |  |  |  |
|  |  |  |  | NUDT2 |  |  |  |
|  |  |  |  | NUDT4 |  |  |  |
|  |  |  |  | NUDT9P1 |  |  |  |
|  |  |  |  | NUP210L |  |  |  |
|  |  |  |  | NUP62CL |  |  |  |
|  |  |  |  | NXN |  |  |  |
|  |  |  |  | NYNRIN |  |  |  |
|  |  |  |  | OAF |  |  |  |
|  |  |  |  | OAZ3 |  |  |  |
|  |  |  |  | OBP2B |  |  |  |
|  |  |  |  | ODF2L |  |  |  |
|  |  |  |  | ODF3L1 |  |  |  |
|  |  |  |  | ODF3L2 |  |  |  |
|  |  |  |  | OGFRP1 |  |  |  |
|  |  |  |  | OGN |  |  |  |
|  |  |  |  | OLFM2 |  |  |  |
|  |  |  |  | OLFM4 |  |  |  |
|  |  |  |  | OMA1 |  |  |  |
|  |  |  |  | OMD |  |  |  |
|  |  |  |  | ONECUT1 |  |  |  |
|  |  |  |  | OOSP2 |  |  |  |
|  |  |  |  | OPRD1 |  |  |  |
|  |  |  |  | OR10H1 |  |  |  |
|  |  |  |  | OR12D3 |  |  |  |
|  |  |  |  | OR2A4 |  |  |  |
|  |  |  |  | OR2B3 |  |  |  |
|  |  |  |  | OR2C3 |  |  |  |
|  |  |  |  | OR2F1 |  |  |  |
|  |  |  |  | OR2F2 |  |  |  |
|  |  |  |  | OR2J3 |  |  |  |
|  |  |  |  | OR4D2 |  |  |  |
|  |  |  |  | OR5K1 |  |  |  |
|  |  |  |  | OR5P3 |  |  |  |
|  |  |  |  | OR7A10 |  |  |  |
|  |  |  |  | OR7D2 |  |  |  |
|  |  |  |  | OR8B2 |  |  |  |
|  |  |  |  | OR8G1 |  |  |  |
|  |  |  |  | ORM1 |  |  |  |
|  |  |  |  | OS9 |  |  |  |
|  |  |  |  | OSBPL6 |  |  |  |
|  |  |  |  | OSBPL8 |  |  |  |
|  |  |  |  | OSCP1 |  |  |  |
|  |  |  |  | OSGEPL1 |  |  |  |
|  |  |  |  | OSGIN1 |  |  |  |
|  |  |  |  | OSGIN2 |  |  |  |
|  |  |  |  | OSR1 |  |  |  |
|  |  |  |  | OSR2 |  |  |  |
|  |  |  |  | OSTM1 |  |  |  |
|  |  |  |  | OTOS |  |  |  |
|  |  |  |  | OXGR1 |  |  |  |
|  |  |  |  | OXT |  |  |  |
|  |  |  |  | P2RX1 |  |  |  |
|  |  |  |  | P2RX3 |  |  |  |
|  |  |  |  | P2RY14 |  |  |  |
|  |  |  |  | PACRG |  |  |  |
|  |  |  |  | PACSIN3 |  |  |  |
|  |  |  |  | PAGE1 |  |  |  |
|  |  |  |  | PALM3 |  |  |  |
|  |  |  |  | PAN2 |  |  |  |
|  |  |  |  | PANX1 |  |  |  |
|  |  |  |  | PAPLN |  |  |  |
|  |  |  |  | PAPPA |  |  |  |
|  |  |  |  | PAPSS2 |  |  |  |
|  |  |  |  | PAQR3 |  |  |  |
|  |  |  |  | PAQR4 |  |  |  |
|  |  |  |  | PARD3 |  |  |  |
|  |  |  |  | PARS2 |  |  |  |
|  |  |  |  | PARVA |  |  |  |
|  |  |  |  | PATL2 |  |  |  |
|  |  |  |  | PAWR |  |  |  |
|  |  |  |  | PAX3 |  |  |  |
|  |  |  |  | PBX4 |  |  |  |
|  |  |  |  | PBXIP1 |  |  |  |
|  |  |  |  | PCBD1 |  |  |  |
|  |  |  |  | PCDH10 |  |  |  |
|  |  |  |  | PCDH17 |  |  |  |
|  |  |  |  | PCDH9 |  |  |  |
|  |  |  |  | PCDHA10 |  |  |  |
|  |  |  |  | PCDHA2 |  |  |  |
|  |  |  |  | PCDHA5 |  |  |  |
|  |  |  |  | PCDHB11 |  |  |  |
|  |  |  |  | PCDHB12 |  |  |  |
|  |  |  |  | PCDHB15 |  |  |  |
|  |  |  |  | PCDHB16 |  |  |  |
|  |  |  |  | PCDHB2 |  |  |  |
|  |  |  |  | PCDHB4 |  |  |  |
|  |  |  |  | PCDHB5 |  |  |  |
|  |  |  |  | PCDHB6 |  |  |  |
|  |  |  |  | PCDHB7 |  |  |  |
|  |  |  |  | PCDHB9 |  |  |  |
|  |  |  |  | PCDHGA10 |  |  |  |
|  |  |  |  | PCDHGA9 |  |  |  |
|  |  |  |  | PCDHGB6 |  |  |  |
|  |  |  |  | PCDHGB7 |  |  |  |
|  |  |  |  | PCDHGC3 |  |  |  |
|  |  |  |  | PCDHGC4 |  |  |  |
|  |  |  |  | PCK1 |  |  |  |
|  |  |  |  | PCMTD2 |  |  |  |
|  |  |  |  | PCP2 |  |  |  |
|  |  |  |  | PCYOX1 |  |  |  |
|  |  |  |  | PDCD1 |  |  |  |
|  |  |  |  | PDCL2 |  |  |  |
|  |  |  |  | PDE4B |  |  |  |
|  |  |  |  | PDE6A |  |  |  |
|  |  |  |  | PDE6H |  |  |  |
|  |  |  |  | PDE7B |  |  |  |
|  |  |  |  | PDE8A |  |  |  |
|  |  |  |  | PDE9A |  |  |  |
|  |  |  |  | PDGFRA |  |  |  |
|  |  |  |  | PDLIM5 |  |  |  |
|  |  |  |  | PDP2 |  |  |  |
|  |  |  |  | PDPK1 |  |  |  |
|  |  |  |  | PDZD2 |  |  |  |
|  |  |  |  | PDZD9 |  |  |  |
|  |  |  |  | PDZK1 |  |  |  |
|  |  |  |  | PDZK1IP1 |  |  |  |
|  |  |  |  | PDZRN3 |  |  |  |
|  |  |  |  | PEAR1 |  |  |  |
|  |  |  |  | PEG10 |  |  |  |
|  |  |  |  | PELI1 |  |  |  |
|  |  |  |  | PENK |  |  |  |
|  |  |  |  | PEX6 |  |  |  |
|  |  |  |  | PF4 |  |  |  |
|  |  |  |  | PF4V1 |  |  |  |
|  |  |  |  | PFKFB2 |  |  |  |
|  |  |  |  | PGAM2 |  |  |  |
|  |  |  |  | PGF |  |  |  |
|  |  |  |  | PGLYRP2 |  |  |  |
|  |  |  |  | PGM5 |  |  |  |
|  |  |  |  | PHACTR2 |  |  |  |
|  |  |  |  | PHF2P1 |  |  |  |
|  |  |  |  | PHKG1 |  |  |  |
|  |  |  |  | PHLDA3 |  |  |  |
|  |  |  |  | PHOSPHO1 |  |  |  |
|  |  |  |  | PHOSPHO2 |  |  |  |
|  |  |  |  | PHOX2A |  |  |  |
|  |  |  |  | PHOX2B |  |  |  |
|  |  |  |  | PHYH |  |  |  |
|  |  |  |  | PI15 |  |  |  |
|  |  |  |  | PI16 |  |  |  |
|  |  |  |  | PIBF1 |  |  |  |
|  |  |  |  | PIDD1 |  |  |  |
|  |  |  |  | PIGK |  |  |  |
|  |  |  |  | PIK3C2A |  |  |  |
|  |  |  |  | PIK3C2B |  |  |  |
|  |  |  |  | PIK3C2G |  |  |  |
|  |  |  |  | PIK3CA |  |  |  |
|  |  |  |  | PIM3 |  |  |  |
|  |  |  |  | PIP |  |  |  |
|  |  |  |  | PIP4K2C |  |  |  |
|  |  |  |  | PIR |  |  |  |
|  |  |  |  | PITPNC1 |  |  |  |
|  |  |  |  | PIWIL4 |  |  |  |
|  |  |  |  | PKP2 |  |  |  |
|  |  |  |  | PLA1A |  |  |  |
|  |  |  |  | PLA2G7 |  |  |  |
|  |  |  |  | PLAA |  |  |  |
|  |  |  |  | PLAC1 |  |  |  |
|  |  |  |  | PLAC9 |  |  |  |
|  |  |  |  | PLAG1 |  |  |  |
|  |  |  |  | PLCD1 |  |  |  |
|  |  |  |  | PLCD3 |  |  |  |
|  |  |  |  | PLCD4 |  |  |  |
|  |  |  |  | PLCZ1 |  |  |  |
|  |  |  |  | PLD5 |  |  |  |
|  |  |  |  | PLEC |  |  |  |
|  |  |  |  | PLEKHF1 |  |  |  |
|  |  |  |  | PLEKHG4 |  |  |  |
|  |  |  |  | PLEKHH2 |  |  |  |
|  |  |  |  | PLEKHN1 |  |  |  |
|  |  |  |  | PLEKHO1 |  |  |  |
|  |  |  |  | PLEKHS1 |  |  |  |
|  |  |  |  | PLIN4 |  |  |  |
|  |  |  |  | PLN |  |  |  |
|  |  |  |  | PLS1 |  |  |  |
|  |  |  |  | PLSCR4 |  |  |  |
|  |  |  |  | PLXNA2 |  |  |  |
|  |  |  |  | PLXNC1 |  |  |  |
|  |  |  |  | PM20D2 |  |  |  |
|  |  |  |  | PMAIP1 |  |  |  |
|  |  |  |  | PMEL |  |  |  |
|  |  |  |  | PMFBP1 |  |  |  |
|  |  |  |  | PMP2 |  |  |  |
|  |  |  |  | PNISR |  |  |  |
|  |  |  |  | PNLIP |  |  |  |
|  |  |  |  | PNLIPRP2 |  |  |  |
|  |  |  |  | PNLIPRP3 |  |  |  |
|  |  |  |  | PNMT |  |  |  |
|  |  |  |  | PNPLA1 |  |  |  |
|  |  |  |  | PNPLA3 |  |  |  |
|  |  |  |  | PNPLA4 |  |  |  |
|  |  |  |  | PNPLA6 |  |  |  |
|  |  |  |  | PNRC1 |  |  |  |
|  |  |  |  | PODN |  |  |  |
|  |  |  |  | POLE4 |  |  |  |
|  |  |  |  | POLM |  |  |  |
|  |  |  |  | POLR1D |  |  |  |
|  |  |  |  | POMC |  |  |  |
|  |  |  |  | PON2 |  |  |  |
|  |  |  |  | PON3 |  |  |  |
|  |  |  |  | POU1F1 |  |  |  |
|  |  |  |  | POU2F3 |  |  |  |
|  |  |  |  | POU5F1P3 |  |  |  |
|  |  |  |  | POU5F1P4 |  |  |  |
|  |  |  |  | PPARGC1A |  |  |  |
|  |  |  |  | PPBP |  |  |  |
|  |  |  |  | PPFIBP1 |  |  |  |
|  |  |  |  | PPFIBP2 |  |  |  |
|  |  |  |  | PPIEL |  |  |  |
|  |  |  |  | PPL |  |  |  |
|  |  |  |  | PPM1J |  |  |  |
|  |  |  |  | PPP1R13L |  |  |  |
|  |  |  |  | PPP1R15B |  |  |  |
|  |  |  |  | PPP1R16A |  |  |  |
|  |  |  |  | PPP1R17 |  |  |  |
|  |  |  |  | PPP1R1C |  |  |  |
|  |  |  |  | PPP1R32 |  |  |  |
|  |  |  |  | PPP1R36 |  |  |  |
|  |  |  |  | PPP1R3D |  |  |  |
|  |  |  |  | PPP2R1A |  |  |  |
|  |  |  |  | PPP4R2 |  |  |  |
|  |  |  |  | PRAME |  |  |  |
|  |  |  |  | PRAMEF12 |  |  |  |
|  |  |  |  | PRAP1 |  |  |  |
|  |  |  |  | PRB3 |  |  |  |
|  |  |  |  | PRDM12 |  |  |  |
|  |  |  |  | PRDM14 |  |  |  |
|  |  |  |  | PRDM16 |  |  |  |
|  |  |  |  | PRDM6 |  |  |  |
|  |  |  |  | PRDM7 |  |  |  |
|  |  |  |  | PRG2 |  |  |  |
|  |  |  |  | PRG4 |  |  |  |
|  |  |  |  | PRICKLE3 |  |  |  |
|  |  |  |  | PRIMA1 |  |  |  |
|  |  |  |  | PRKACG |  |  |  |
|  |  |  |  | PRKG2 |  |  |  |
|  |  |  |  | PRKX |  |  |  |
|  |  |  |  | PRKY |  |  |  |
|  |  |  |  | PRM1 |  |  |  |
|  |  |  |  | PRM3 |  |  |  |
|  |  |  |  | PRMT7 |  |  |  |
|  |  |  |  | PROK1 |  |  |  |
|  |  |  |  | PROK2 |  |  |  |
|  |  |  |  | PROM1 |  |  |  |
|  |  |  |  | PROSER2 |  |  |  |
|  |  |  |  | PRPF40B |  |  |  |
|  |  |  |  | PRPH |  |  |  |
|  |  |  |  | PRPS1 |  |  |  |
|  |  |  |  | PRR15 |  |  |  |
|  |  |  |  | PRR16 |  |  |  |
|  |  |  |  | PRR19 |  |  |  |
|  |  |  |  | PRR35 |  |  |  |
|  |  |  |  | PRR5L |  |  |  |
|  |  |  |  | PRR7 |  |  |  |
|  |  |  |  | PRRG2 |  |  |  |
|  |  |  |  | PRRX2 |  |  |  |
|  |  |  |  | PRSS12 |  |  |  |
|  |  |  |  | PRSS16 |  |  |  |
|  |  |  |  | PRSS21 |  |  |  |
|  |  |  |  | PRSS33 |  |  |  |
|  |  |  |  | PRSS35 |  |  |  |
|  |  |  |  | PRSS37 |  |  |  |
|  |  |  |  | PRSS50 |  |  |  |
|  |  |  |  | PRSS54 |  |  |  |
|  |  |  |  | PRSS8 |  |  |  |
|  |  |  |  | PRTFDC1 |  |  |  |
|  |  |  |  | PRTN3 |  |  |  |
|  |  |  |  | PRX |  |  |  |
|  |  |  |  | PSAT1 |  |  |  |
|  |  |  |  | PSCA |  |  |  |
|  |  |  |  | PSG11 |  |  |  |
|  |  |  |  | PSG2 |  |  |  |
|  |  |  |  | PSG3 |  |  |  |
|  |  |  |  | PSIP1 |  |  |  |
|  |  |  |  | PSMD5 |  |  |  |
|  |  |  |  | PSORS1C1 |  |  |  |
|  |  |  |  | PSPN |  |  |  |
|  |  |  |  | PSTPIP1 |  |  |  |
|  |  |  |  | PTCD2 |  |  |  |
|  |  |  |  | PTCH1 |  |  |  |
|  |  |  |  | PTCH2 |  |  |  |
|  |  |  |  | PTCHD1 |  |  |  |
|  |  |  |  | PTGER2 |  |  |  |
|  |  |  |  | PTGES |  |  |  |
|  |  |  |  | PTGFR |  |  |  |
|  |  |  |  | PTGR2 |  |  |  |
|  |  |  |  | PTH |  |  |  |
|  |  |  |  | PTHLH |  |  |  |
|  |  |  |  | PTK6 |  |  |  |
|  |  |  |  | PTMS |  |  |  |
|  |  |  |  | PTOV1 |  |  |  |
|  |  |  |  | PTP4A3 |  |  |  |
|  |  |  |  | PTPDC1 |  |  |  |
|  |  |  |  | PTPRCAP |  |  |  |
|  |  |  |  | PTPRE |  |  |  |
|  |  |  |  | PTPRF |  |  |  |
|  |  |  |  | PTPRG |  |  |  |
|  |  |  |  | PTPRH |  |  |  |
|  |  |  |  | PTPRO |  |  |  |
|  |  |  |  | PTPRZ1 |  |  |  |
|  |  |  |  | PURB |  |  |  |
|  |  |  |  | PXYLP1 |  |  |  |
|  |  |  |  | PYROXD1 |  |  |  |
|  |  |  |  | PYROXD2 |  |  |  |
|  |  |  |  | PYY |  |  |  |
|  |  |  |  | PYY2 |  |  |  |
|  |  |  |  | QPCT |  |  |  |
|  |  |  |  | QPCTL |  |  |  |
|  |  |  |  | QRFPR |  |  |  |
|  |  |  |  | QRICH2 |  |  |  |
|  |  |  |  | QSER1 |  |  |  |
|  |  |  |  | QTRT1 |  |  |  |
|  |  |  |  | R3HDML |  |  |  |
|  |  |  |  | RAB12 |  |  |  |
|  |  |  |  | RAB17 |  |  |  |
|  |  |  |  | RAB23 |  |  |  |
|  |  |  |  | RAB3GAP2 |  |  |  |
|  |  |  |  | RAB40A |  |  |  |
|  |  |  |  | RAB6A |  |  |  |
|  |  |  |  | RAB9BP1 |  |  |  |
|  |  |  |  | RAC3 |  |  |  |
|  |  |  |  | RADIL |  |  |  |
|  |  |  |  | RAET1E |  |  |  |
|  |  |  |  | RAG2 |  |  |  |
|  |  |  |  | RALGDS |  |  |  |
|  |  |  |  | RALGPS2 |  |  |  |
|  |  |  |  | RAMP1 |  |  |  |
|  |  |  |  | RAMP2 |  |  |  |
|  |  |  |  | RAMP3 |  |  |  |
|  |  |  |  | RAPSN |  |  |  |
|  |  |  |  | RASD1 |  |  |  |
|  |  |  |  | RASEF |  |  |  |
|  |  |  |  | RASGRP1 |  |  |  |
|  |  |  |  | RASSF2 |  |  |  |
|  |  |  |  | RASSF9 |  |  |  |
|  |  |  |  | RAX2 |  |  |  |
|  |  |  |  | RBAKDN |  |  |  |
|  |  |  |  | RBBP5 |  |  |  |
|  |  |  |  | RBBP8NL |  |  |  |
|  |  |  |  | RBM12B |  |  |  |
|  |  |  |  | RBM20 |  |  |  |
|  |  |  |  | RBM6 |  |  |  |
|  |  |  |  | RBMS3 |  |  |  |
|  |  |  |  | RBMXL1 |  |  |  |
|  |  |  |  | RBMY3AP |  |  |  |
|  |  |  |  | RBP3 |  |  |  |
|  |  |  |  | RBPJL |  |  |  |
|  |  |  |  | RBPMS2 |  |  |  |
|  |  |  |  | RCAN3 |  |  |  |
|  |  |  |  | RCBTB2 |  |  |  |
|  |  |  |  | RCVRN |  |  |  |
|  |  |  |  | RDH12 |  |  |  |
|  |  |  |  | RDH16 |  |  |  |
|  |  |  |  | REC8 |  |  |  |
|  |  |  |  | REEP3 |  |  |  |
|  |  |  |  | REG1A |  |  |  |
|  |  |  |  | REG1B |  |  |  |
|  |  |  |  | RELT |  |  |  |
|  |  |  |  | REM1 |  |  |  |
|  |  |  |  | REM2 |  |  |  |
|  |  |  |  | RETN |  |  |  |
|  |  |  |  | RETNLB |  |  |  |
|  |  |  |  | REV3L |  |  |  |
|  |  |  |  | REXO1 |  |  |  |
|  |  |  |  | RFPL1 |  |  |  |
|  |  |  |  | RFPL3S |  |  |  |
|  |  |  |  | RFX6 |  |  |  |
|  |  |  |  | RGL4 |  |  |  |
|  |  |  |  | RGMA |  |  |  |
|  |  |  |  | RGN |  |  |  |
|  |  |  |  | RGS17 |  |  |  |
|  |  |  |  | RGS22 |  |  |  |
|  |  |  |  | RGS5 |  |  |  |
|  |  |  |  | RGS6 |  |  |  |
|  |  |  |  | RGS9BP |  |  |  |
|  |  |  |  | RHBDF1 |  |  |  |
|  |  |  |  | RHCG |  |  |  |
|  |  |  |  | RHOB |  |  |  |
|  |  |  |  | RHOBTB1 |  |  |  |
|  |  |  |  | RHOBTB3 |  |  |  |
|  |  |  |  | RHOU |  |  |  |
|  |  |  |  | RHOV |  |  |  |
|  |  |  |  | RIBC2 |  |  |  |
|  |  |  |  | RIMKLB |  |  |  |
|  |  |  |  | RIPK2 |  |  |  |
|  |  |  |  | RIPPLY1 |  |  |  |
|  |  |  |  | RLBP1 |  |  |  |
|  |  |  |  | RND2 |  |  |  |
|  |  |  |  | RNF151 |  |  |  |
|  |  |  |  | RNF182 |  |  |  |
|  |  |  |  | RNF212 |  |  |  |
|  |  |  |  | RNF215 |  |  |  |
|  |  |  |  | RNF25 |  |  |  |
|  |  |  |  | RNF43 |  |  |  |
|  |  |  |  | RNF5 |  |  |  |
|  |  |  |  | RNPC3 |  |  |  |
|  |  |  |  | ROBO1 |  |  |  |
|  |  |  |  | ROBO2 |  |  |  |
|  |  |  |  | ROBO3 |  |  |  |
|  |  |  |  | ROM1 |  |  |  |
|  |  |  |  | ROPN1B |  |  |  |
|  |  |  |  | ROPN1L |  |  |  |
|  |  |  |  | ROR2 |  |  |  |
|  |  |  |  | RORA |  |  |  |
|  |  |  |  | RPA4 |  |  |  |
|  |  |  |  | RPGRIP1L |  |  |  |
|  |  |  |  | RPL10L |  |  |  |
|  |  |  |  | RPL39L |  |  |  |
|  |  |  |  | RPL3L |  |  |  |
|  |  |  |  | RPP25 |  |  |  |
|  |  |  |  | RPPH1 |  |  |  |
|  |  |  |  | RPS11 |  |  |  |
|  |  |  |  | RPS23 |  |  |  |
|  |  |  |  | RPS4Y1 |  |  |  |
|  |  |  |  | RRAD |  |  |  |
|  |  |  |  | RRAGB |  |  |  |
|  |  |  |  | RRAGD |  |  |  |
|  |  |  |  | RRP9 |  |  |  |
|  |  |  |  | RSPH1 |  |  |  |
|  |  |  |  | RSPH4A |  |  |  |
|  |  |  |  | RSPH6A |  |  |  |
|  |  |  |  | RSPH9 |  |  |  |
|  |  |  |  | RSPO4 |  |  |  |
|  |  |  |  | RXRG |  |  |  |
|  |  |  |  | RYR3 |  |  |  |
|  |  |  |  | S100A7 |  |  |  |
|  |  |  |  | S100P |  |  |  |
|  |  |  |  | S100Z |  |  |  |
|  |  |  |  | S1PR4 |  |  |  |
|  |  |  |  | SACS |  |  |  |
|  |  |  |  | SALL4 |  |  |  |
|  |  |  |  | SAMD1 |  |  |  |
|  |  |  |  | SAMD13 |  |  |  |
|  |  |  |  | SAMD5 |  |  |  |
|  |  |  |  | SAMHD1 |  |  |  |
|  |  |  |  | SATB2 |  |  |  |
|  |  |  |  | SBSN |  |  |  |
|  |  |  |  | SBSPON |  |  |  |
|  |  |  |  | SCAF1 |  |  |  |
|  |  |  |  | SCAF11 |  |  |  |
|  |  |  |  | SCARA5 |  |  |  |
|  |  |  |  | SCARB1 |  |  |  |
|  |  |  |  | SCARNA17 |  |  |  |
|  |  |  |  | SCARNA2 |  |  |  |
|  |  |  |  | SCFD2 |  |  |  |
|  |  |  |  | SCG2 |  |  |  |
|  |  |  |  | SCGB1A1 |  |  |  |
|  |  |  |  | SCGB1D1 |  |  |  |
|  |  |  |  | SCGB1D2 |  |  |  |
|  |  |  |  | SCGB2A1 |  |  |  |
|  |  |  |  | SCGB2B2 |  |  |  |
|  |  |  |  | SCGB3A1 |  |  |  |
|  |  |  |  | SCGN |  |  |  |
|  |  |  |  | SCML1 |  |  |  |
|  |  |  |  | SCN4A |  |  |  |
|  |  |  |  | SCN7A |  |  |  |
|  |  |  |  | SCN9A |  |  |  |
|  |  |  |  | SCNN1B |  |  |  |
|  |  |  |  | SCNN1D |  |  |  |
|  |  |  |  | SCNN1G |  |  |  |
|  |  |  |  | SCRG1 |  |  |  |
|  |  |  |  | SCUBE1 |  |  |  |
|  |  |  |  | SCUBE2 |  |  |  |
|  |  |  |  | SDC3 |  |  |  |
|  |  |  |  | SDR9C7 |  |  |  |
|  |  |  |  | SEC11C |  |  |  |
|  |  |  |  | SEC14L1 |  |  |  |
|  |  |  |  | SEC14L1P1 |  |  |  |
|  |  |  |  | SEC14L2 |  |  |  |
|  |  |  |  | SECISBP2 |  |  |  |
|  |  |  |  | SEL1L2 |  |  |  |
|  |  |  |  | SELE |  |  |  |
|  |  |  |  | SELENBP1 |  |  |  |
|  |  |  |  | SEMA3A |  |  |  |
|  |  |  |  | SEMA3B |  |  |  |
|  |  |  |  | SEMA3C |  |  |  |
|  |  |  |  | SEMA3D |  |  |  |
|  |  |  |  | SEMA3E |  |  |  |
|  |  |  |  | SEMA4B |  |  |  |
|  |  |  |  | SEMA5A |  |  |  |
|  |  |  |  | SEMA5B |  |  |  |
|  |  |  |  | SEMA6A |  |  |  |
|  |  |  |  | SEMA6D |  |  |  |
|  |  |  |  | SEMG1 |  |  |  |
|  |  |  |  | SEMG2 |  |  |  |
|  |  |  |  | SENP7 |  |  |  |
|  |  |  |  | SEPTIN1 |  |  |  |
|  |  |  |  | SEPTIN11 |  |  |  |
|  |  |  |  | SEPTIN6 |  |  |  |
|  |  |  |  | SERAC1 |  |  |  |
|  |  |  |  | SERHL2 |  |  |  |
|  |  |  |  | SERINC2 |  |  |  |
|  |  |  |  | SERPINA10 |  |  |  |
|  |  |  |  | SERPINA6 |  |  |  |
|  |  |  |  | SERPINB12 |  |  |  |
|  |  |  |  | SERPINB2 |  |  |  |
|  |  |  |  | SERPINB4 |  |  |  |
|  |  |  |  | SERPIND1 |  |  |  |
|  |  |  |  | SERPINE2 |  |  |  |
|  |  |  |  | SERTAD4-AS1 |  |  |  |
|  |  |  |  | SESN3 |  |  |  |
|  |  |  |  | SETBP1 |  |  |  |
|  |  |  |  | SETD6 |  |  |  |
|  |  |  |  | SF3A2 |  |  |  |
|  |  |  |  | SF3B4 |  |  |  |
|  |  |  |  | SFRP1 |  |  |  |
|  |  |  |  | SFRP5 |  |  |  |
|  |  |  |  | SFTA2 |  |  |  |
|  |  |  |  | SFTA3 |  |  |  |
|  |  |  |  | SFXN3 |  |  |  |
|  |  |  |  | SGCE |  |  |  |
|  |  |  |  | SGCG |  |  |  |
|  |  |  |  | SGCZ |  |  |  |
|  |  |  |  | SGK1 |  |  |  |
|  |  |  |  | SH2B2 |  |  |  |
|  |  |  |  | SH3BGR |  |  |  |
|  |  |  |  | SH3BP1 |  |  |  |
|  |  |  |  | SH3BP4 |  |  |  |
|  |  |  |  | SH3D19 |  |  |  |
|  |  |  |  | SH3KBP1 |  |  |  |
|  |  |  |  | SH3RF1 |  |  |  |
|  |  |  |  | SHANK2-AS3 |  |  |  |
|  |  |  |  | SHC4 |  |  |  |
|  |  |  |  | SHCBP1L |  |  |  |
|  |  |  |  | SHISA3 |  |  |  |
|  |  |  |  | SHPK |  |  |  |
|  |  |  |  | SHROOM2 |  |  |  |
|  |  |  |  | SHROOM4 |  |  |  |
|  |  |  |  | SI |  |  |  |
|  |  |  |  | SIGLEC16 |  |  |  |
|  |  |  |  | SIGLEC17P |  |  |  |
|  |  |  |  | SIK1 |  |  |  |
|  |  |  |  | SIM2 |  |  |  |
|  |  |  |  | SIMC1 |  |  |  |
|  |  |  |  | SIT1 |  |  |  |
|  |  |  |  | SIX1 |  |  |  |
|  |  |  |  | SIX3 |  |  |  |
|  |  |  |  | SIX4 |  |  |  |
|  |  |  |  | SIX6 |  |  |  |
|  |  |  |  | SKIDA1 |  |  |  |
|  |  |  |  | SLC12A8 |  |  |  |
|  |  |  |  | SLC13A4 |  |  |  |
|  |  |  |  | SLC16A6 |  |  |  |
|  |  |  |  | SLC17A2 |  |  |  |
|  |  |  |  | SLC18A1 |  |  |  |
|  |  |  |  | SLC18A3 |  |  |  |
|  |  |  |  | SLC19A2 |  |  |  |
|  |  |  |  | SLC22A1 |  |  |  |
|  |  |  |  | SLC22A12 |  |  |  |
|  |  |  |  | SLC22A3 |  |  |  |
|  |  |  |  | SLC22A5 |  |  |  |
|  |  |  |  | SLC22A6 |  |  |  |
|  |  |  |  | SLC23A1 |  |  |  |
|  |  |  |  | SLC25A1 |  |  |  |
|  |  |  |  | SLC25A10 |  |  |  |
|  |  |  |  | SLC25A15 |  |  |  |
|  |  |  |  | SLC25A21 |  |  |  |
|  |  |  |  | SLC25A33 |  |  |  |
|  |  |  |  | SLC25A36 |  |  |  |
|  |  |  |  | SLC25A45 |  |  |  |
|  |  |  |  | SLC25A47 |  |  |  |
|  |  |  |  | SLC26A10 |  |  |  |
|  |  |  |  | SLC27A1 |  |  |  |
|  |  |  |  | SLC27A5 |  |  |  |
|  |  |  |  | SLC27A6 |  |  |  |
|  |  |  |  | SLC29A1 |  |  |  |
|  |  |  |  | SLC29A4 |  |  |  |
|  |  |  |  | SLC2A1 |  |  |  |
|  |  |  |  | SLC35D3 |  |  |  |
|  |  |  |  | SLC35E3 |  |  |  |
|  |  |  |  | SLC35F1 |  |  |  |
|  |  |  |  | SLC35F2 |  |  |  |
|  |  |  |  | SLC35F4 |  |  |  |
|  |  |  |  | SLC35G3 |  |  |  |
|  |  |  |  | SLC36A4 |  |  |  |
|  |  |  |  | SLC37A1 |  |  |  |
|  |  |  |  | SLC38A3 |  |  |  |
|  |  |  |  | SLC38A4 |  |  |  |
|  |  |  |  | SLC38A5 |  |  |  |
|  |  |  |  | SLC44A2 |  |  |  |
|  |  |  |  | SLC44A5 |  |  |  |
|  |  |  |  | SLC46A2 |  |  |  |
|  |  |  |  | SLC47A1 |  |  |  |
|  |  |  |  | SLC4A3 |  |  |  |
|  |  |  |  | SLC4A4 |  |  |  |
|  |  |  |  | SLC4A8 |  |  |  |
|  |  |  |  | SLC5A10 |  |  |  |
|  |  |  |  | SLC5A4 |  |  |  |
|  |  |  |  | SLC6A11 |  |  |  |
|  |  |  |  | SLC6A18 |  |  |  |
|  |  |  |  | SLC6A20 |  |  |  |
|  |  |  |  | SLC6A3 |  |  |  |
|  |  |  |  | SLC6A6 |  |  |  |
|  |  |  |  | SLC6A8 |  |  |  |
|  |  |  |  | SLC6A9 |  |  |  |
|  |  |  |  | SLC7A13 |  |  |  |
|  |  |  |  | SLC7A3 |  |  |  |
|  |  |  |  | SLC7A5 |  |  |  |
|  |  |  |  | SLC9A2 |  |  |  |
|  |  |  |  | SLC9A3R1 |  |  |  |
|  |  |  |  | SLC9A3R2 |  |  |  |
|  |  |  |  | SLC9A7 |  |  |  |
|  |  |  |  | SLCO1B1 |  |  |  |
|  |  |  |  | SLCO1B3 |  |  |  |
|  |  |  |  | SLCO1C1 |  |  |  |
|  |  |  |  | SLCO2A1 |  |  |  |
|  |  |  |  | SLCO4A1 |  |  |  |
|  |  |  |  | SLCO5A1 |  |  |  |
|  |  |  |  | SLFN13 |  |  |  |
|  |  |  |  | SLITRK3 |  |  |  |
|  |  |  |  | SLITRK6 |  |  |  |
|  |  |  |  | SLX4IP |  |  |  |
|  |  |  |  | SMARCB1 |  |  |  |
|  |  |  |  | SMARCD3 |  |  |  |
|  |  |  |  | SMC1B |  |  |  |
|  |  |  |  | SMC3 |  |  |  |
|  |  |  |  | SMIM1 |  |  |  |
|  |  |  |  | SMIM20 |  |  |  |
|  |  |  |  | SMIM21 |  |  |  |
|  |  |  |  | SMIM22 |  |  |  |
|  |  |  |  | SMIM5 |  |  |  |
|  |  |  |  | SMOC2 |  |  |  |
|  |  |  |  | SMOX |  |  |  |
|  |  |  |  | SMPD2 |  |  |  |
|  |  |  |  | SMR3A |  |  |  |
|  |  |  |  | SMR3B |  |  |  |
|  |  |  |  | SMTN |  |  |  |
|  |  |  |  | SMTNL1 |  |  |  |
|  |  |  |  | SMYD2 |  |  |  |
|  |  |  |  | SMYD3 |  |  |  |
|  |  |  |  | SMYD5 |  |  |  |
|  |  |  |  | SNAI1 |  |  |  |
|  |  |  |  | SNCAIP |  |  |  |
|  |  |  |  | SNED1 |  |  |  |
|  |  |  |  | SNORA72 |  |  |  |
|  |  |  |  | SNRPE |  |  |  |
|  |  |  |  | SNTG1 |  |  |  |
|  |  |  |  | SNTN |  |  |  |
|  |  |  |  | SNX1 |  |  |  |
|  |  |  |  | SNX10 |  |  |  |
|  |  |  |  | SNX31 |  |  |  |
|  |  |  |  | SOD3 |  |  |  |
|  |  |  |  | SOGA1 |  |  |  |
|  |  |  |  | SOHLH2 |  |  |  |
|  |  |  |  | SORBS3 |  |  |  |
|  |  |  |  | SOST |  |  |  |
|  |  |  |  | SOX13 |  |  |  |
|  |  |  |  | SOX14 |  |  |  |
|  |  |  |  | SOX18 |  |  |  |
|  |  |  |  | SOX2-OT |  |  |  |
|  |  |  |  | SOX21 |  |  |  |
|  |  |  |  | SOX3 |  |  |  |
|  |  |  |  | SOX30 |  |  |  |
|  |  |  |  | SOX9-AS1 |  |  |  |
|  |  |  |  | SP5 |  |  |  |
|  |  |  |  | SP8 |  |  |  |
|  |  |  |  | SPA17 |  |  |  |
|  |  |  |  | SPACA7 |  |  |  |
|  |  |  |  | SPAG17 |  |  |  |
|  |  |  |  | SPAG8 |  |  |  |
|  |  |  |  | SPATA12 |  |  |  |
|  |  |  |  | SPATA18 |  |  |  |
|  |  |  |  | SPATA20 |  |  |  |
|  |  |  |  | SPATA22 |  |  |  |
|  |  |  |  | SPATA3 |  |  |  |
|  |  |  |  | SPATA7 |  |  |  |
|  |  |  |  | SPATA9 |  |  |  |
|  |  |  |  | SPATC1 |  |  |  |
|  |  |  |  | SPATC1L |  |  |  |
|  |  |  |  | SPESP1 |  |  |  |
|  |  |  |  | SPG7 |  |  |  |
|  |  |  |  | SPIC |  |  |  |
|  |  |  |  | SPINK1 |  |  |  |
|  |  |  |  | SPINK2 |  |  |  |
|  |  |  |  | SPINK5 |  |  |  |
|  |  |  |  | SPINK7 |  |  |  |
|  |  |  |  | SPN |  |  |  |
|  |  |  |  | SPNS3 |  |  |  |
|  |  |  |  | SPO11 |  |  |  |
|  |  |  |  | SPON1 |  |  |  |
|  |  |  |  | SPP2 |  |  |  |
|  |  |  |  | SPPL2C |  |  |  |
|  |  |  |  | SPRED2 |  |  |  |
|  |  |  |  | SPSB1 |  |  |  |
|  |  |  |  | SPTSSB |  |  |  |
|  |  |  |  | SRD5A1 |  |  |  |
|  |  |  |  | SRD5A3 |  |  |  |
|  |  |  |  | SRGAP2C |  |  |  |
|  |  |  |  | SRI |  |  |  |
|  |  |  |  | SRPK3 |  |  |  |
|  |  |  |  | SRRM2 |  |  |  |
|  |  |  |  | SRSF6 |  |  |  |
|  |  |  |  | SRXN1 |  |  |  |
|  |  |  |  | SRY |  |  |  |
|  |  |  |  | SSBP2 |  |  |  |
|  |  |  |  | SSPO |  |  |  |
|  |  |  |  | SSTR4 |  |  |  |
|  |  |  |  | SSTR5 |  |  |  |
|  |  |  |  | ST3GAL6 |  |  |  |
|  |  |  |  | ST6GALNAC2 |  |  |  |
|  |  |  |  | ST6GALNAC4 |  |  |  |
|  |  |  |  | ST7-AS1 |  |  |  |
|  |  |  |  | ST7-OT4 |  |  |  |
|  |  |  |  | ST8SIA1 |  |  |  |
|  |  |  |  | ST8SIA5 |  |  |  |
|  |  |  |  | STAC3 |  |  |  |
|  |  |  |  | STAG2 |  |  |  |
|  |  |  |  | STAG3 |  |  |  |
|  |  |  |  | STAG3L4 |  |  |  |
|  |  |  |  | STARD13 |  |  |  |
|  |  |  |  | STARD5 |  |  |  |
|  |  |  |  | STARD6 |  |  |  |
|  |  |  |  | STC2 |  |  |  |
|  |  |  |  | STEAP1B |  |  |  |
|  |  |  |  | STEAP4 |  |  |  |
|  |  |  |  | STIM2 |  |  |  |
|  |  |  |  | STK31 |  |  |  |
|  |  |  |  | STK32A |  |  |  |
|  |  |  |  | STK32B |  |  |  |
|  |  |  |  | STK38L |  |  |  |
|  |  |  |  | STOML3 |  |  |  |
|  |  |  |  | STRADB |  |  |  |
|  |  |  |  | STRN4 |  |  |  |
|  |  |  |  | STX19 |  |  |  |
|  |  |  |  | SUCNR1 |  |  |  |
|  |  |  |  | SUGCT |  |  |  |
|  |  |  |  | SULF2 |  |  |  |
|  |  |  |  | SULT1A1 |  |  |  |
|  |  |  |  | SULT1C4 |  |  |  |
|  |  |  |  | SUN3 |  |  |  |
|  |  |  |  | SUPT16H |  |  |  |
|  |  |  |  | SURF2 |  |  |  |
|  |  |  |  | SUSD1 |  |  |  |
|  |  |  |  | SVOPL |  |  |  |
|  |  |  |  | SYCE3 |  |  |  |
|  |  |  |  | SYCP2 |  |  |  |
|  |  |  |  | SYCP2L |  |  |  |
|  |  |  |  | SYNDIG1L |  |  |  |
|  |  |  |  | SYT6 |  |  |  |
|  |  |  |  | SYTL2 |  |  |  |
|  |  |  |  | SYTL4 |  |  |  |
|  |  |  |  | SYTL5 |  |  |  |
|  |  |  |  | TAAR1 |  |  |  |
|  |  |  |  | TAAR8 |  |  |  |
|  |  |  |  | TACR2 |  |  |  |
|  |  |  |  | TACR3 |  |  |  |
|  |  |  |  | TAF13 |  |  |  |
|  |  |  |  | TAF1C |  |  |  |
|  |  |  |  | TAF1D |  |  |  |
|  |  |  |  | TAF1L |  |  |  |
|  |  |  |  | TAF9B |  |  |  |
|  |  |  |  | TAOK1 |  |  |  |
|  |  |  |  | TAS2R13 |  |  |  |
|  |  |  |  | TAS2R38 |  |  |  |
|  |  |  |  | TAS2R40 |  |  |  |
|  |  |  |  | TAS2R41 |  |  |  |
|  |  |  |  | TAS2R5 |  |  |  |
|  |  |  |  | TBC1D10C |  |  |  |
|  |  |  |  | TBC1D2 |  |  |  |
|  |  |  |  | TBC1D8 |  |  |  |
|  |  |  |  | TBL1XR1 |  |  |  |
|  |  |  |  | TBL1Y |  |  |  |
|  |  |  |  | TBX19 |  |  |  |
|  |  |  |  | TBX20 |  |  |  |
|  |  |  |  | TBX22 |  |  |  |
|  |  |  |  | TBX5 |  |  |  |
|  |  |  |  | TCAM1P |  |  |  |
|  |  |  |  | TCAP |  |  |  |
|  |  |  |  | TCF15 |  |  |  |
|  |  |  |  | TCF4 |  |  |  |
|  |  |  |  | TCF7L2 |  |  |  |
|  |  |  |  | TCP10 |  |  |  |
|  |  |  |  | TCTEX1D1 |  |  |  |
|  |  |  |  | TCTEX1D2 |  |  |  |
|  |  |  |  | TDO2 |  |  |  |
|  |  |  |  | TDRD1 |  |  |  |
|  |  |  |  | TECRL |  |  |  |
|  |  |  |  | TEDDM1 |  |  |  |
|  |  |  |  | TEKT1 |  |  |  |
|  |  |  |  | TEKT2 |  |  |  |
|  |  |  |  | TEKT3 |  |  |  |
|  |  |  |  | TENM3 |  |  |  |
|  |  |  |  | TEPP |  |  |  |
|  |  |  |  | TET2 |  |  |  |
|  |  |  |  | TEX12 |  |  |  |
|  |  |  |  | TEX13A |  |  |  |
|  |  |  |  | TEX14 |  |  |  |
|  |  |  |  | TEX30 |  |  |  |
|  |  |  |  | TEX33 |  |  |  |
|  |  |  |  | TEX35 |  |  |  |
|  |  |  |  | TEX38 |  |  |  |
|  |  |  |  | TFAP2B |  |  |  |
|  |  |  |  | TFAP2C |  |  |  |
|  |  |  |  | TFAP2D |  |  |  |
|  |  |  |  | TFAP4 |  |  |  |
|  |  |  |  | TFCP2L1 |  |  |  |
|  |  |  |  | TFDP2 |  |  |  |
|  |  |  |  | TFPT |  |  |  |
|  |  |  |  | TGFA |  |  |  |
|  |  |  |  | TGFB3 |  |  |  |
|  |  |  |  | TGFBRAP1 |  |  |  |
|  |  |  |  | TGM5 |  |  |  |
|  |  |  |  | TH |  |  |  |
|  |  |  |  | THAP2 |  |  |  |
|  |  |  |  | THBS2 |  |  |  |
|  |  |  |  | THBS4 |  |  |  |
|  |  |  |  | THEG |  |  |  |
|  |  |  |  | THEM4 |  |  |  |
|  |  |  |  | THEM5 |  |  |  |
|  |  |  |  | THEM6 |  |  |  |
|  |  |  |  | THNSL2 |  |  |  |
|  |  |  |  | THSD7A |  |  |  |
|  |  |  |  | THSD7B |  |  |  |
|  |  |  |  | TIAM1 |  |  |  |
|  |  |  |  | TIE1 |  |  |  |
|  |  |  |  | TIGD1 |  |  |  |
|  |  |  |  | TIGIT |  |  |  |
|  |  |  |  | TIMD4 |  |  |  |
|  |  |  |  | TIMP3 |  |  |  |
|  |  |  |  | TINAGL1 |  |  |  |
|  |  |  |  | TKT |  |  |  |
|  |  |  |  | TKTL1 |  |  |  |
|  |  |  |  | TLCD1 |  |  |  |
|  |  |  |  | TLE1 |  |  |  |
|  |  |  |  | TLE2 |  |  |  |
|  |  |  |  | TLX2 |  |  |  |
|  |  |  |  | TLX3 |  |  |  |
|  |  |  |  | TM4SF20 |  |  |  |
|  |  |  |  | TMC1 |  |  |  |
|  |  |  |  | TMCO2 |  |  |  |
|  |  |  |  | TMCO3 |  |  |  |
|  |  |  |  | TMCO6 |  |  |  |
|  |  |  |  | TMED6 |  |  |  |
|  |  |  |  | TMEM102 |  |  |  |
|  |  |  |  | TMEM106B |  |  |  |
|  |  |  |  | TMEM116 |  |  |  |
|  |  |  |  | TMEM117 |  |  |  |
|  |  |  |  | TMEM132E |  |  |  |
|  |  |  |  | TMEM141 |  |  |  |
|  |  |  |  | TMEM163 |  |  |  |
|  |  |  |  | TMEM165 |  |  |  |
|  |  |  |  | TMEM17 |  |  |  |
|  |  |  |  | TMEM170A |  |  |  |
|  |  |  |  | TMEM190 |  |  |  |
|  |  |  |  | TMEM200A |  |  |  |
|  |  |  |  | TMEM200B |  |  |  |
|  |  |  |  | TMEM200C |  |  |  |
|  |  |  |  | TMEM201 |  |  |  |
|  |  |  |  | TMEM207 |  |  |  |
|  |  |  |  | TMEM216 |  |  |  |
|  |  |  |  | TMEM217 |  |  |  |
|  |  |  |  | TMEM229B |  |  |  |
|  |  |  |  | TMEM243 |  |  |  |
|  |  |  |  | TMEM255B |  |  |  |
|  |  |  |  | TMEM259 |  |  |  |
|  |  |  |  | TMEM30B |  |  |  |
|  |  |  |  | TMEM38B |  |  |  |
|  |  |  |  | TMEM52 |  |  |  |
|  |  |  |  | TMEM61 |  |  |  |
|  |  |  |  | TMEM74 |  |  |  |
|  |  |  |  | TMEM75 |  |  |  |
|  |  |  |  | TMEM79 |  |  |  |
|  |  |  |  | TMEM88 |  |  |  |
|  |  |  |  | TMEM95 |  |  |  |
|  |  |  |  | TMEM98 |  |  |  |
|  |  |  |  | TMIE |  |  |  |
|  |  |  |  | TMIGD2 |  |  |  |
|  |  |  |  | TMOD1 |  |  |  |
|  |  |  |  | TMPRSS5 |  |  |  |
|  |  |  |  | TMSB4Y |  |  |  |
|  |  |  |  | TMTC2 |  |  |  |
|  |  |  |  | TMX4 |  |  |  |
|  |  |  |  | TNFRSF10C |  |  |  |
|  |  |  |  | TNFRSF10D |  |  |  |
|  |  |  |  | TNFRSF17 |  |  |  |
|  |  |  |  | TNFRSF21 |  |  |  |
|  |  |  |  | TNFRSF25 |  |  |  |
|  |  |  |  | TNFRSF4 |  |  |  |
|  |  |  |  | TNIK |  |  |  |
|  |  |  |  | TNIP3 |  |  |  |
|  |  |  |  | TNNC2 |  |  |  |
|  |  |  |  | TNNI3K |  |  |  |
|  |  |  |  | TNP1 |  |  |  |
|  |  |  |  | TOB2 |  |  |  |
|  |  |  |  | TOMM40L |  |  |  |
|  |  |  |  | TOP1MT |  |  |  |
|  |  |  |  | TOP3B |  |  |  |
|  |  |  |  | TOPORS |  |  |  |
|  |  |  |  | TOR2A |  |  |  |
|  |  |  |  | TOX2 |  |  |  |
|  |  |  |  | TP53BP2 |  |  |  |
|  |  |  |  | TPBG |  |  |  |
|  |  |  |  | TPGS1 |  |  |  |
|  |  |  |  | TPH2 |  |  |  |
|  |  |  |  | TPM1 |  |  |  |
|  |  |  |  | TPO |  |  |  |
|  |  |  |  | TPP2 |  |  |  |
|  |  |  |  | TPSAB1 |  |  |  |
|  |  |  |  | TPTE |  |  |  |
|  |  |  |  | TRAF2 |  |  |  |
|  |  |  |  | TRAK2 |  |  |  |
|  |  |  |  | TRANK1 |  |  |  |
|  |  |  |  | TRAPPC1 |  |  |  |
|  |  |  |  | TRAT1 |  |  |  |
|  |  |  |  | TRAV12-2 |  |  |  |
|  |  |  |  | TRBC1 |  |  |  |
|  |  |  |  | TRDV3 |  |  |  |
|  |  |  |  | TREH |  |  |  |
|  |  |  |  | TRH |  |  |  |
|  |  |  |  | TRHR |  |  |  |
|  |  |  |  | TRIM17 |  |  |  |
|  |  |  |  | TRIM36 |  |  |  |
|  |  |  |  | TRIM40 |  |  |  |
|  |  |  |  | TRIM45 |  |  |  |
|  |  |  |  | TRIM52 |  |  |  |
|  |  |  |  | TRIM54 |  |  |  |
|  |  |  |  | TRIM63 |  |  |  |
|  |  |  |  | TRIM69 |  |  |  |
|  |  |  |  | TRIM9 |  |  |  |
|  |  |  |  | TRIML2 |  |  |  |
|  |  |  |  | TRMT2A |  |  |  |
|  |  |  |  | TRPC2 |  |  |  |
|  |  |  |  | TRPC4 |  |  |  |
|  |  |  |  | TRPC5 |  |  |  |
|  |  |  |  | TRPM3 |  |  |  |
|  |  |  |  | TRPV4 |  |  |  |
|  |  |  |  | TSACC |  |  |  |
|  |  |  |  | TSC22D4 |  |  |  |
|  |  |  |  | TSGA10IP |  |  |  |
|  |  |  |  | TSHR |  |  |  |
|  |  |  |  | TSHZ3 |  |  |  |
|  |  |  |  | TSLP |  |  |  |
|  |  |  |  | TSPAN1 |  |  |  |
|  |  |  |  | TSPAN10 |  |  |  |
|  |  |  |  | TSPAN12 |  |  |  |
|  |  |  |  | TSPAN33 |  |  |  |
|  |  |  |  | TSPO2 |  |  |  |
|  |  |  |  | TSPYL6 |  |  |  |
|  |  |  |  | TSTD2 |  |  |  |
|  |  |  |  | TTC14 |  |  |  |
|  |  |  |  | TTC25 |  |  |  |
|  |  |  |  | TTC29 |  |  |  |
|  |  |  |  | TTC30A |  |  |  |
|  |  |  |  | TTC32 |  |  |  |
|  |  |  |  | TTLL2 |  |  |  |
|  |  |  |  | TTLL6 |  |  |  |
|  |  |  |  | TTR |  |  |  |
|  |  |  |  | TTTY10 |  |  |  |
|  |  |  |  | TTTY13 |  |  |  |
|  |  |  |  | TTTY15 |  |  |  |
|  |  |  |  | TTYH1 |  |  |  |
|  |  |  |  | TUBA8 |  |  |  |
|  |  |  |  | TUBB2B |  |  |  |
|  |  |  |  | TUBB3 |  |  |  |
|  |  |  |  | TUBBP5 |  |  |  |
|  |  |  |  | TUBGCP6 |  |  |  |
|  |  |  |  | TUFT1 |  |  |  |
|  |  |  |  | TULP1 |  |  |  |
|  |  |  |  | TUSC1 |  |  |  |
|  |  |  |  | TXK |  |  |  |
|  |  |  |  | TXLNGY |  |  |  |
|  |  |  |  | TXNDC2 |  |  |  |
|  |  |  |  | TXNIP |  |  |  |
|  |  |  |  | TXNRD2 |  |  |  |
|  |  |  |  | TYW3 |  |  |  |
|  |  |  |  | U2AF1L4 |  |  |  |
|  |  |  |  | UBAP2 |  |  |  |
|  |  |  |  | UBASH3B |  |  |  |
|  |  |  |  | UBE2M |  |  |  |
|  |  |  |  | UBN2 |  |  |  |
|  |  |  |  | UBQLNL |  |  |  |
|  |  |  |  | UBXN10 |  |  |  |
|  |  |  |  | UBXN11 |  |  |  |
|  |  |  |  | UCHL3 |  |  |  |
|  |  |  |  | UCMA |  |  |  |
|  |  |  |  | UCN |  |  |  |
|  |  |  |  | UCN2 |  |  |  |
|  |  |  |  | UCN3 |  |  |  |
|  |  |  |  | UFSP1 |  |  |  |
|  |  |  |  | UGT2A3 |  |  |  |
|  |  |  |  | UGT2B17 |  |  |  |
|  |  |  |  | UGT2B28 |  |  |  |
|  |  |  |  | UGT2B4 |  |  |  |
|  |  |  |  | UHMK1 |  |  |  |
|  |  |  |  | UHRF2 |  |  |  |
|  |  |  |  | ULBP2 |  |  |  |
|  |  |  |  | UMOD |  |  |  |
|  |  |  |  | UMODL1 |  |  |  |
|  |  |  |  | UNC13B |  |  |  |
|  |  |  |  | UNC45B |  |  |  |
|  |  |  |  | UNC5B |  |  |  |
|  |  |  |  | UPF3A |  |  |  |
|  |  |  |  | UQCC2 |  |  |  |
|  |  |  |  | USF1 |  |  |  |
|  |  |  |  | USH2A |  |  |  |
|  |  |  |  | USP26 |  |  |  |
|  |  |  |  | USP40 |  |  |  |
|  |  |  |  | USP9Y |  |  |  |
|  |  |  |  | UTP23 |  |  |  |
|  |  |  |  | UTRN |  |  |  |
|  |  |  |  | UTY |  |  |  |
|  |  |  |  | VANGL2 |  |  |  |
|  |  |  |  | VARS2 |  |  |  |
|  |  |  |  | VASH1 |  |  |  |
|  |  |  |  | VCPKMT |  |  |  |
|  |  |  |  | VEGFC |  |  |  |
|  |  |  |  | VENTX |  |  |  |
|  |  |  |  | VEPH1 |  |  |  |
|  |  |  |  | VGF |  |  |  |
|  |  |  |  | VILL |  |  |  |
|  |  |  |  | VIT |  |  |  |
|  |  |  |  | VLDLR |  |  |  |
|  |  |  |  | VPS37D |  |  |  |
|  |  |  |  | VSIG10L |  |  |  |
|  |  |  |  | VSTM1 |  |  |  |
|  |  |  |  | VTCN1 |  |  |  |
|  |  |  |  | VWA1 |  |  |  |
|  |  |  |  | VWA3A |  |  |  |
|  |  |  |  | VWA5A |  |  |  |
|  |  |  |  | VWCE |  |  |  |
|  |  |  |  | VWDE |  |  |  |
|  |  |  |  | WBP2NL |  |  |  |
|  |  |  |  | WDR19 |  |  |  |
|  |  |  |  | WDR24 |  |  |  |
|  |  |  |  | WDR25 |  |  |  |
|  |  |  |  | WDR46 |  |  |  |
|  |  |  |  | WDR49 |  |  |  |
|  |  |  |  | WDR60 |  |  |  |
|  |  |  |  | WEE2-AS1 |  |  |  |
|  |  |  |  | WFDC10A |  |  |  |
|  |  |  |  | WFDC11 |  |  |  |
|  |  |  |  | WFDC13 |  |  |  |
|  |  |  |  | WFDC21P |  |  |  |
|  |  |  |  | WFDC3 |  |  |  |
|  |  |  |  | WFDC5 |  |  |  |
|  |  |  |  | WFIKKN1 |  |  |  |
|  |  |  |  | WHAMM |  |  |  |
|  |  |  |  | WIPF3 |  |  |  |
|  |  |  |  | WNK1 |  |  |  |
|  |  |  |  | WNK3 |  |  |  |
|  |  |  |  | WNK4 |  |  |  |
|  |  |  |  | WNT1 |  |  |  |
|  |  |  |  | WNT16 |  |  |  |
|  |  |  |  | WNT2 |  |  |  |
|  |  |  |  | WNT7A |  |  |  |
|  |  |  |  | WSB1 |  |  |  |
|  |  |  |  | WT1 |  |  |  |
|  |  |  |  | WT1-AS |  |  |  |
|  |  |  |  | WTIP |  |  |  |
|  |  |  |  | WWC1 |  |  |  |
|  |  |  |  | WWC2 |  |  |  |
|  |  |  |  | XIRP1 |  |  |  |
|  |  |  |  | XIST |  |  |  |
|  |  |  |  | YAF2 |  |  |  |
|  |  |  |  | YBEY |  |  |  |
|  |  |  |  | YBX2 |  |  |  |
|  |  |  |  | YDJC |  |  |  |
|  |  |  |  | ZAP70 |  |  |  |
|  |  |  |  | ZBBX |  |  |  |
|  |  |  |  | ZBED2 |  |  |  |
|  |  |  |  | ZBED3 |  |  |  |
|  |  |  |  | ZBED6CL |  |  |  |
|  |  |  |  | ZBED9 |  |  |  |
|  |  |  |  | ZBTB12 |  |  |  |
|  |  |  |  | ZBTB26 |  |  |  |
|  |  |  |  | ZBTB43 |  |  |  |
|  |  |  |  | ZBTB5 |  |  |  |
|  |  |  |  | ZBTB6 |  |  |  |
|  |  |  |  | ZBTB7B |  |  |  |
|  |  |  |  | ZBTB7C |  |  |  |
|  |  |  |  | ZC2HC1B |  |  |  |
|  |  |  |  | ZC2HC1C |  |  |  |
|  |  |  |  | ZC3H12C |  |  |  |
|  |  |  |  | ZCCHC13 |  |  |  |
|  |  |  |  | ZCCHC2 |  |  |  |
|  |  |  |  | ZCWPW1 |  |  |  |
|  |  |  |  | ZCWPW2 |  |  |  |
|  |  |  |  | ZDBF2 |  |  |  |
|  |  |  |  | ZDHHC13 |  |  |  |
|  |  |  |  | ZDHHC14 |  |  |  |
|  |  |  |  | ZDHHC2 |  |  |  |
|  |  |  |  | ZDHHC23 |  |  |  |
|  |  |  |  | ZDHHC9 |  |  |  |
|  |  |  |  | ZFAND2A |  |  |  |
|  |  |  |  | ZFAND4 |  |  |  |
|  |  |  |  | ZFHX4 |  |  |  |
|  |  |  |  | ZFP14 |  |  |  |
|  |  |  |  | ZFP3 |  |  |  |
|  |  |  |  | ZFP37 |  |  |  |
|  |  |  |  | ZFP41 |  |  |  |
|  |  |  |  | ZFP69 |  |  |  |
|  |  |  |  | ZFP82 |  |  |  |
|  |  |  |  | ZFY |  |  |  |
|  |  |  |  | ZG16B |  |  |  |
|  |  |  |  | ZIC2 |  |  |  |
|  |  |  |  | ZIC3 |  |  |  |
|  |  |  |  | ZIC5 |  |  |  |
|  |  |  |  | ZIM3 |  |  |  |
|  |  |  |  | ZKSCAN7 |  |  |  |
|  |  |  |  | ZMAT1 |  |  |  |
|  |  |  |  | ZMAT3 |  |  |  |
|  |  |  |  | ZMAT5 |  |  |  |
|  |  |  |  | ZMYND10 |  |  |  |
|  |  |  |  | ZMYND12 |  |  |  |
|  |  |  |  | ZMYND15 |  |  |  |
|  |  |  |  | ZNF114 |  |  |  |
|  |  |  |  | ZNF12 |  |  |  |
|  |  |  |  | ZNF135 |  |  |  |
|  |  |  |  | ZNF136 |  |  |  |
|  |  |  |  | ZNF148 |  |  |  |
|  |  |  |  | ZNF157 |  |  |  |
|  |  |  |  | ZNF160 |  |  |  |
|  |  |  |  | ZNF165 |  |  |  |
|  |  |  |  | ZNF184 |  |  |  |
|  |  |  |  | ZNF195 |  |  |  |
|  |  |  |  | ZNF205 |  |  |  |
|  |  |  |  | ZNF208 |  |  |  |
|  |  |  |  | ZNF22 |  |  |  |
|  |  |  |  | ZNF221 |  |  |  |
|  |  |  |  | ZNF23 |  |  |  |
|  |  |  |  | ZNF233 |  |  |  |
|  |  |  |  | ZNF257 |  |  |  |
|  |  |  |  | ZNF259P1 |  |  |  |
|  |  |  |  | ZNF264 |  |  |  |
|  |  |  |  | ZNF274 |  |  |  |
|  |  |  |  | ZNF295-AS1 |  |  |  |
|  |  |  |  | ZNF296 |  |  |  |
|  |  |  |  | ZNF300P1 |  |  |  |
|  |  |  |  | ZNF329 |  |  |  |
|  |  |  |  | ZNF331 |  |  |  |
|  |  |  |  | ZNF334 |  |  |  |
|  |  |  |  | ZNF397 |  |  |  |
|  |  |  |  | ZNF404 |  |  |  |
|  |  |  |  | ZNF415 |  |  |  |
|  |  |  |  | ZNF417 |  |  |  |
|  |  |  |  | ZNF442 |  |  |  |
|  |  |  |  | ZNF462 |  |  |  |
|  |  |  |  | ZNF469 |  |  |  |
|  |  |  |  | ZNF485 |  |  |  |
|  |  |  |  | ZNF497 |  |  |  |
|  |  |  |  | ZNF514 |  |  |  |
|  |  |  |  | ZNF518A |  |  |  |
|  |  |  |  | ZNF518B |  |  |  |
|  |  |  |  | ZNF526 |  |  |  |
|  |  |  |  | ZNF529 |  |  |  |
|  |  |  |  | ZNF542P |  |  |  |
|  |  |  |  | ZNF560 |  |  |  |
|  |  |  |  | ZNF571 |  |  |  |
|  |  |  |  | ZNF572 |  |  |  |
|  |  |  |  | ZNF573 |  |  |  |
|  |  |  |  | ZNF574 |  |  |  |
|  |  |  |  | ZNF577 |  |  |  |
|  |  |  |  | ZNF580 |  |  |  |
|  |  |  |  | ZNF584 |  |  |  |
|  |  |  |  | ZNF594 |  |  |  |
|  |  |  |  | ZNF596 |  |  |  |
|  |  |  |  | ZNF610 |  |  |  |
|  |  |  |  | ZNF618 |  |  |  |
|  |  |  |  | ZNF620 |  |  |  |
|  |  |  |  | ZNF630 |  |  |  |
|  |  |  |  | ZNF646 |  |  |  |
|  |  |  |  | ZNF652 |  |  |  |
|  |  |  |  | ZNF654 |  |  |  |
|  |  |  |  | ZNF660 |  |  |  |
|  |  |  |  | ZNF665 |  |  |  |
|  |  |  |  | ZNF674 |  |  |  |
|  |  |  |  | ZNF687 |  |  |  |
|  |  |  |  | ZNF749 |  |  |  |
|  |  |  |  | ZNF750 |  |  |  |
|  |  |  |  | ZNF76 |  |  |  |
|  |  |  |  | ZNF784 |  |  |  |
|  |  |  |  | ZNF785 |  |  |  |
|  |  |  |  | ZNF787 |  |  |  |
|  |  |  |  | ZNF792 |  |  |  |
|  |  |  |  | ZNF80 |  |  |  |
|  |  |  |  | ZNF804B |  |  |  |
|  |  |  |  | ZNF827 |  |  |  |
|  |  |  |  | ZNF83 |  |  |  |
|  |  |  |  | ZNF84 |  |  |  |
|  |  |  |  | ZNF862 |  |  |  |
|  |  |  |  | ZNF876P |  |  |  |
|  |  |  |  | ZNRF3 |  |  |  |
|  |  |  |  | ZP1 |  |  |  |
|  |  |  |  | ZP2 |  |  |  |
|  |  |  |  | ZPBP |  |  |  |
|  |  |  |  | ZSCAN18 |  |  |  |

**Table S7. DO analysis.**

| **ID** | **Description** | **GeneRatio** | **BgRatio** | **pvalue** | **qvalue** | **Count** | **GeneID** |
| --- | --- | --- | --- | --- | --- | --- | --- |
| DOID:0112202 | developmental and epileptic encephalopathy | 0.1299 (10/77) | 0.0110 (111/10086) | 2.178685768244034e-09 | 1.7124470138398108e-06 | 10 | GABRA1/KCNA2/GLS/GRIN1/GABRA2/EEF1A2/GABRB2/SLC1A2/GABRA5/SLC12A5 |
| DOID:150 | disease of mental health | 0.4156 (32/77) | 0.1775 (1790/10086) | 9.562826698972738e-09 | 3.758190892696286e-06 | 32 | KCNJ6/CAMKK2/CNKSR2/CCKBR/EEF1A2/GSTO2/ADCY1/SLC1A2/GABRA5/RYR2/MEF2C/HTR2A/GABRB2/ADRB1/GABRA1/FABP3/CASK/GLS/ATP6V1B2/GRIN1/HIVEP2/GABRA2/GAD2/ADRA2A/RELA/ABCA1/CDK5/TP53/GRM2/THRB/VIPR1/SMARCA2 |
| DOID:1826 | epilepsy | 0.2338 (18/77) | 0.0630 (635/10086) | 7.509285202307759e-08 | 1.4870008336433079e-05 | 18 | GABRA1/GAD2/KCNA2/SCN2B/GLS/BCL2L2/RELA/RORB/GRIN1/GABRA2/PTK2B/CDK5/EEF1A2/GABRB2/SLC1A2/GABRA5/MEF2C/SLC12A5 |
| DOID:331 | central nervous system disease | 0.4545 (35/77) | 0.2270 (2290/10086) | 7.567434267904875e-08 | 1.4870008336433079e-05 | 35 | MAPK9/STK17B/CCKBR/EEF1A2/GSTO2/SLC1A2/GABRA5/MEF2C/SLC12A5/BCL2L2/HTR2A/ABL1/PTK2B/IGF2R/GABRB2/KCNA2/ADRB1/GABRA1/FABP3/GLS/GRIN1/HIF1A/HIVEP2/LY6E/GABRA2/GAD2/SCN2B/RELA/ABCA1/RORB/CDK5/TP53/GOT1/MYC/GRM2 |
| DOID:0050701 | electroclinical syndrome | 0.1299 (10/77) | 0.0177 (179/10086) | 2.1534084606383547e-07 | 3.3851581001234934e-05 | 10 | GABRA1/KCNA2/GLS/GRIN1/GABRA2/EEF1A2/GABRB2/SLC1A2/GABRA5/SLC12A5 |
| DOID:303 | substance-related disorder | 0.1558 (12/77) | 0.0314 (317/10086) | 8.100348049010884e-07 | 0.00010214002780654524 | 12 | GAD2/KCNJ6/HTR2A/GRIN1/GRM2/VIPR1/GSTO2/ADCY1/SLC1A2/RYR2/GABRA2/SMARCA2 |
| DOID:10652 | Alzheimer's disease | 0.2208 (17/77) | 0.0666 (672/10086) | 9.443114756686596e-07 | 0.00010214002780654524 | 17 | FABP3/MAPK9/HIVEP2/BCL2L2/ABCA1/HTR2A/ABL1/GRIN1/IGF2R/CDK5/TP53/GRM2/EEF1A2/HIF1A/GSTO2/SLC1A2/ADRB1 |
| DOID:680 | tauopathy | 0.2208 (17/77) | 0.0675 (681/10086) | 1.1364745988747318e-06 | 0.00010214002780654524 | 17 | FABP3/MAPK9/HIVEP2/BCL2L2/ABCA1/HTR2A/ABL1/GRIN1/IGF2R/CDK5/TP53/GRM2/EEF1A2/HIF1A/GSTO2/SLC1A2/ADRB1 |
| DOID:1574 | alcohol use disorder | 0.1299 (10/77) | 0.0213 (215/10086) | 1.1695423031283806e-06 | 0.00010214002780654524 | 10 | GAD2/KCNJ6/HTR2A/GRIN1/VIPR1/GSTO2/ADCY1/RYR2/GABRA2/SMARCA2 |
| DOID:863 | nervous system disease | 0.5195 (40/77) | 0.3224 (3252/10086) | 1.8963662174960813e-06 | 0.000149054384695192 | 40 | MAPK9/PITPNM1/STK17B/CCKBR/EEF1A2/TYRP1/GSTO2/ADCY1/GABRA5/SLC1A2/MEF2C/SLC12A5/BCL2L2/HTR2A/ABL1/PTK2B/IGF2R/GABRB2/ADRB1/KCNA2/GABRA1/FABP3/GLS/RBP4/GRIN1/LCAT/HIF1A/HIVEP2/LY6E/GABRA2/GAD2/SCN2B/RELA/ABCA1/RORB/CDK5/TP53/GOT1/MYC/GRM2 |
| DOID:302 | substance abuse | 0.1299 (10/77) | 0.0229 (231/10086) | 2.2423376525843468e-06 | 0.00016022521772102695 | 10 | GAD2/KCNJ6/HTR2A/GRIN1/VIPR1/GSTO2/ADCY1/RYR2/GABRA2/SMARCA2 |
| DOID:936 | brain disease | 0.2597 (20/77) | 0.1037 (1046/10086) | 6.658142271185594e-06 | 0.0004361083187626564 | 20 | EEF1A2/SLC1A2/GABRA5/MEF2C/SLC12A5/BCL2L2/HTR2A/PTK2B/GABRB2/KCNA2/GABRA1/GLS/GRIN1/HIF1A/GABRA2/GAD2/SCN2B/RELA/RORB/CDK5 |
| DOID:9974 | drug dependence | 0.0519 (4/77) | 0.0023 (23/10086) | 1.348651138033181e-05 | 0.000815415226533908 | 4 | HTR2A/GRM2/GABRA2/KCNJ6 |
| DOID:1932 | Angelman syndrome | 0.0390 (3/77) | 0.0011 (11/10086) | 4.253902454971352e-05 | 0.002237427175605182 | 3 | GABRA1/GABRA5/GABRB2 |
| DOID:1289 | neurodegenerative disease | 0.2857 (22/77) | 0.1381 (1393/10086) | 4.269899190086225e-05 | 0.002237427175605182 | 22 | MAPK9/CCKBR/EEF1A2/GSTO2/SLC1A2/BCL2L2/HTR2A/ABL1/IGF2R/ADRB1/GABRA1/FABP3/GRIN1/HIF1A/HIVEP2/LY6E/GAD2/ABCA1/CDK5/TP53/GOT1/GRM2 |
| DOID:1561 | cognitive disorder | 0.2338 (18/77) | 0.1019 (1028/10086) | 7.19834987040062e-05 | 0.003536189373834304 | 18 | GAD2/FABP3/ADRA2A/HIVEP2/CAMKK2/GLS/ATP6V1B2/HTR2A/ABCA1/GRIN1/CDK5/CCKBR/TP53/GRM2/GABRB2/SLC1A2/GABRA5/SMARCA2 |
| DOID:0060060 | non-Hodgkin lymphoma | 0.0779 (6/77) | 0.0110 (111/10086) | 8.241466511158086e-05 | 0.0036734108327110596 | 6 | BCL2L2/ATP6V1B2/IGF2R/TP53/MYC/HIF1A |
| DOID:0060058 | lymphoma | 0.0909 (7/77) | 0.0161 (162/10086) | 8.51369732916255e-05 | 0.0036734108327110596 | 7 | BCL2L2/ATP6V1B2/RELA/IGF2R/TP53/MYC/HIF1A |
| DOID:2978 | carbohydrate metabolic disorder | 0.2468 (19/77) | 0.1135 (1145/10086) | 8.879746287723936e-05 | 0.0036734108327110596 | 19 | KCNJ6/MAPK9/MEF2C/HTR2A/IGF2R/ADRB1/FABP3/RBP4/LCAT/HIF1A/GAD2/ADRA2A/RELA/ABCA1/KCNJ3/TP53/GOT1/MYC/IMPDH2 |
| DOID:9976 | heroin dependence | 0.0390 (3/77) | 0.0016 (16/10086) | 0.0001410315052530998 | 0.005542538156446822 | 3 | HTR2A/GRM2/KCNJ6 |
| DOID:2531 | hematologic cancer | 0.2727 (21/77) | 0.1395 (1407/10086) | 0.00015638466977267889 | 0.005825029263090579 | 21 | CCNA1/MAPK9/PRDM2/CCKBR/DUSP2/EEF1A2/GSTO2/MEF2C/BCL2L2/ABL1/PTK2B/CBFB/IGF2R/PLK2/ATP6V1B2/HIF1A/RELA/CDK5/TP53/MYC/THRB |
| DOID:1827 | idiopathic generalized epilepsy | 0.0519 (4/77) | 0.0043 (43/10086) | 0.00017036507936349056 | 0.005825029263090579 | 4 | GABRA1/RORB/SLC12A5/GAD2 |
| DOID:10964 | cholesteatoma of middle ear | 0.0390 (3/77) | 0.0017 (17/10086) | 0.00017045251024310853 | 0.005825029263090579 | 3 | RELA/TP53/HIF1A |
| DOID:4195 | hyperglycemia | 0.0909 (7/77) | 0.0181 (183/10086) | 0.0001821717284456218 | 0.0059661241065941135 | 7 | RELA/RBP4/HTR2A/GOT1/MYC/HIF1A/ADRB1 |
| DOID:0060041 | autism spectrum disorder | 0.1169 (9/77) | 0.0315 (318/10086) | 0.0002090705326859093 | 0.006573177547644989 | 9 | GABRA1/CASK/HTR2A/GRIN1/GABRA2/TP53/GABRB2/GABRA5/MEF2C |
| DOID:0060037 | developmental disorder of mental health | 0.1818 (14/77) | 0.0726 (732/10086) | 0.00022177815147197112 | 0.0067045241175757424 | 14 | GABRA1/ADRA2A/CASK/CNKSR2/HTR2A/GRIN1/GABRA2/TP53/THRB/EEF1A2/GABRB2/HIVEP2/GABRA5/MEF2C |
| DOID:9973 | substance dependence | 0.0649 (5/77) | 0.0085 (86/10086) | 0.00023809197787817582 | 0.0068007398484824354 | 5 | KCNJ6/HTR2A/GRM2/SLC1A2/GABRA2 |
| DOID:2750 | glycogen storage disease IV | 0.0260 (2/77) | 0.0004 (4/10086) | 0.00025091788245036975 | 0.0068007398484824354 | 2 | KCNJ6/KCNJ3 |
| DOID:0001816 | angiosarcoma | 0.0260 (2/77) | 0.0004 (4/10086) | 0.00025091788245036975 | 0.0068007398484824354 | 2 | TP53/MYC |
| DOID:0060040 | pervasive developmental disorder | 0.1169 (9/77) | 0.0327 (330/10086) | 0.0002754534892985601 | 0.007087841868069942 | 9 | GABRA1/CASK/HTR2A/GRIN1/GABRA2/TP53/GABRB2/GABRA5/MEF2C |
| DOID:13413 | hepatic encephalopathy | 0.0390 (3/77) | 0.0020 (20/10086) | 0.00028177432384258365 | 0.007087841868069942 | 3 | GABRA1/GLS/HIF1A |
| DOID:4194 | glucose metabolism disease | 0.2208 (17/77) | 0.1034 (1043/10086) | 0.00028856353661353457 | 0.007087841868069942 | 17 | GAD2/FABP3/ADRA2A/MAPK9/RELA/HTR2A/RBP4/ABCA1/IGF2R/TP53/GOT1/MYC/LCAT/HIF1A/IMPDH2/ADRB1/MEF2C |
| DOID:869 | cholesteatoma | 0.0390 (3/77) | 0.0021 (21/10086) | 0.00032720245380530357 | 0.0074492194283151915 | 3 | RELA/TP53/HIF1A |
| DOID:2559 | opiate dependence | 0.0390 (3/77) | 0.0021 (21/10086) | 0.00032720245380530357 | 0.0074492194283151915 | 3 | HTR2A/GRM2/KCNJ6 |
| DOID:8791 | breast carcinoma in situ | 0.0519 (4/77) | 0.0051 (51/10086) | 0.0003317082442633991 | 0.0074492194283151915 | 4 | RELA/TP53/IGF2R/HIF1A |
| DOID:7 | disease of anatomical entity | 0.7143 (55/77) | 0.6375 (6430/10086) | 0.0003869308856248312 | 0.008447991002808814 | 55 | KCNJ6/MAPK9/PITPNM1/STK17B/CCKBR/TRAF3IP2/DUSP2/EEF1A2/NCEH1/TYRP1/GSTO2/ADCY1/CACNB2/MEF2C/GABRA5/SLC1A2/RYR2/SLC12A5/PCTP/BCL2L2/JAG2/HTR2A/ABL1/PTK2B/IGF2R/GABRB2/ADRB1/KCNA2/KCNN1/GABRA1/FABP3/GLS/ATP6V1B2/RBP4/GRIN1/IL10RB/LCAT/HIF1A/HIVEP2/LY6E/GABRA2/GAD2/SCN2B/ADRA2A/CYP26B1/RELA/ABCA1/RORB/CDK5/TP53/GOT1/MYC/GRM2/THRB/VIPR1 |
| DOID:9970 | obesity | 0.1558 (12/77) | 0.0591 (596/10086) | 0.00042218441473211967 | 0.008968566215660705 | 12 | GAD2/FABP3/ADRA2A/RELA/RBP4/HTR2A/ABCA1/GOT1/MYC/HIF1A/ADRB1/GABRA2 |
| DOID:654 | overnutrition | 0.1558 (12/77) | 0.0594 (599/10086) | 0.00044181486925711964 | 0.009138591769370949 | 12 | GAD2/FABP3/ADRA2A/RELA/RBP4/HTR2A/ABCA1/GOT1/MYC/HIF1A/ADRB1/GABRA2 |
| DOID:12849 | autistic disorder | 0.0909 (7/77) | 0.0215 (217/10086) | 0.0005137503344503252 | 0.010354045201998861 | 7 | GABRA1/HTR2A/GABRA2/TP53/GABRB2/GABRA5/MEF2C |
| DOID:3324 | mood disorder | 0.1039 (8/77) | 0.0292 (295/10086) | 0.000640833035970787 | 0.012592369156825966 | 8 | FABP3/ADRA2A/HTR2A/GRIN1/CDK5/GRM2/GABRB2/GABRA5 |
| DOID:707 | B-cell lymphoma | 0.0519 (4/77) | 0.0062 (63/10086) | 0.0007455335957737142 | 0.014292424543369252 | 4 | ATP6V1B2/MYC/TP53/BCL2L2 |
| DOID:374 | nutrition disease | 0.1558 (12/77) | 0.0633 (638/10086) | 0.0007764357321694678 | 0.01453044013060004 | 12 | GAD2/FABP3/ADRA2A/RELA/RBP4/HTR2A/ABCA1/GOT1/MYC/HIF1A/ADRB1/GABRA2 |
| DOID:0060071 | pre-malignant neoplasm | 0.0779 (6/77) | 0.0168 (169/10086) | 0.000801248609977635 | 0.014646079242847 | 6 | MAPK9/RELA/IGF2R/TP53/THRB/HIF1A |
| DOID:2747 | glycogen storage disease | 0.0390 (3/77) | 0.0029 (29/10086) | 0.000865960581217443 | 0.015125444818598005 | 3 | KCNJ6/KCNJ3/HIF1A |
| DOID:0050728 | glycogen metabolism disorder | 0.0390 (3/77) | 0.0029 (29/10086) | 0.000865960581217443 | 0.015125444818598005 | 3 | KCNJ6/KCNJ3/HIF1A |
| DOID:771 | retinal cell cancer | 0.0649 (5/77) | 0.0119 (120/10086) | 0.0010984344668176185 | 0.01836956363656698 | 5 | CCNA1/CDK5/TP53/MYC/HIF1A |
| DOID:768 | retinoblastoma | 0.0649 (5/77) | 0.0119 (120/10086) | 0.0010984344668176185 | 0.01836956363656698 | 5 | CCNA1/CDK5/TP53/MYC/HIF1A |
| DOID:7400 | Nijmegen breakage syndrome | 0.0260 (2/77) | 0.0008 (8/10086) | 0.0011512891479850953 | 0.01859403158483687 | 2 | TP53/MYC |
| DOID:0060307 | autosomal dominant intellectual developmental disorder | 0.0519 (4/77) | 0.0070 (71/10086) | 0.0011694343182599505 | 0.01859403158483687 | 4 | HIVEP2/MEF2C/EEF1A2/GRIN1 |
| DOID:4645 | retinal cancer | 0.0649 (5/77) | 0.0121 (122/10086) | 0.0011828264366944574 | 0.01859403158483687 | 5 | CCNA1/CDK5/TP53/MYC/HIF1A |
| DOID:3312 | bipolar disorder | 0.0779 (6/77) | 0.0182 (184/10086) | 0.0012457366913538276 | 0.01919900077262958 | 6 | FABP3/HTR2A/GRIN1/GRM2/GABRB2/GABRA5 |
| DOID:8725 | vascular dementia | 0.0390 (3/77) | 0.0034 (34/10086) | 0.0013854484853029851 | 0.02094158672015666 | 3 | TP53/GRIN1/CDK5 |
| DOID:1094 | attention deficit hyperactivity disorder | 0.0519 (4/77) | 0.0074 (75/10086) | 0.0014345672869515488 | 0.0210645584772587 | 4 | HTR2A/THRB/MEF2C/ADRA2A |
| DOID:3910 | lung adenocarcinoma | 0.0909 (7/77) | 0.0257 (259/10086) | 0.001452230648713847 | 0.0210645584772587 | 7 | FABP3/RELA/IGF2R/TP53/MYC/EEF1A2/HIF1A |
| DOID:5773 | oral submucous fibrosis | 0.0260 (2/77) | 0.0009 (9/10086) | 0.0014739830995537258 | 0.0210645584772587 | 2 | TP53/HIF1A |
| DOID:0060038 | specific developmental disorder | 0.1299 (10/77) | 0.0502 (506/10086) | 0.0015461666137074948 | 0.021307440010338387 | 10 | ADRA2A/CASK/CNKSR2/HTR2A/GRIN1/TP53/THRB/EEF1A2/HIVEP2/MEF2C |
| DOID:178 | vascular disease | 0.2727 (21/77) | 0.1651 (1665/10086) | 0.001562693965615979 | 0.021307440010338387 | 21 | MAPK9/NCEH1/CACNB2/MEF2C/PCTP/BCL2L2/HTR2A/PTK2B/ADRB1/FABP3/RBP4/GRIN1/LCAT/HIF1A/ADRA2A/RELA/ABCA1/CDK5/TP53/GOT1/MYC |
| DOID:1287 | cardiovascular system disease | 0.3247 (25/77) | 0.2136 (2154/10086) | 0.0015723047335873108 | 0.021307440010338387 | 25 | MAPK9/EEF1A2/NCEH1/CACNB2/MEF2C/RYR2/PCTP/BCL2L2/JAG2/HTR2A/PTK2B/ADRB1/FABP3/RBP4/GRIN1/LCAT/HIF1A/SCN2B/ADRA2A/RELA/ABCA1/CDK5/TP53/GOT1/MYC |
| DOID:0060158 | acquired metabolic disease | 0.1558 (12/77) | 0.0706 (712/10086) | 0.002002690889935696 | 0.026679915923550117 | 12 | GAD2/FABP3/ADRA2A/RELA/RBP4/HTR2A/ABCA1/GOT1/MYC/HIF1A/ADRB1/GABRA2 |
| DOID:8719 | in situ carcinoma | 0.0649 (5/77) | 0.0139 (140/10086) | 0.0021741556781721743 | 0.02830827121180982 | 5 | RELA/IGF2R/TP53/THRB/HIF1A |
| DOID:175 | vascular cancer | 0.0260 (2/77) | 0.0011 (11/10086) | 0.0022329679581834716 | 0.02830827121180982 | 2 | TP53/MYC |
| DOID:591 | phobic disorder | 0.0260 (2/77) | 0.0011 (11/10086) | 0.0022329679581834716 | 0.02830827121180982 | 2 | HTR2A/CAMKK2 |
| DOID:5419 | schizophrenia | 0.1558 (12/77) | 0.0717 (723/10086) | 0.002277959085035975 | 0.028420251441877407 | 12 | GAD2/FABP3/ADRA2A/HIVEP2/ATP6V1B2/HTR2A/GRIN1/TP53/GRM2/GABRB2/SLC1A2/SMARCA2 |
| DOID:5520 | head and neck squamous cell carcinoma | 0.0779 (6/77) | 0.0206 (208/10086) | 0.002323236015953967 | 0.028532242320934657 | 6 | MAPK9/RELA/TP53/MYC/HIF1A/SMARCA2 |
| DOID:2468 | psychotic disorder | 0.1558 (12/77) | 0.0724 (730/10086) | 0.0024688430667525905 | 0.02985401000719286 | 12 | GAD2/FABP3/ADRA2A/HIVEP2/ATP6V1B2/HTR2A/GRIN1/TP53/GRM2/GABRB2/SLC1A2/SMARCA2 |
| DOID:225 | syndrome | 0.3506 (27/77) | 0.2461 (2482/10086) | 0.0025082776396602637 | 0.02987130643595405 | 27 | CCNA1/KCNJ6/CCKBR/TYRP1/CACNB2/GABRA5/RYR2/HTR2A/ABL1/IGF2R/GABRB2/ADRB1/GABRA1/FABP3/CASK/ATP6V1B2/RBP4/LCAT/HIF1A/GAD2/ADRA2A/RELA/ABCA1/TP53/GOT1/MYC/THRB |
| DOID:176 | cardiovascular cancer | 0.0260 (2/77) | 0.0012 (12/10086) | 0.002668270867456078 | 0.030842072085595255 | 2 | TP53/MYC |
| DOID:13948 | bladder neck obstruction | 0.0260 (2/77) | 0.0012 (12/10086) | 0.002668270867456078 | 0.030842072085595255 | 2 | HTR2A/HIF1A |
| DOID:326 | ischemia | 0.1169 (9/77) | 0.0460 (464/10086) | 0.0030610503368466682 | 0.03486935601103596 | 9 | FABP3/MAPK9/BCL2L2/CDK5/PTK2B/TP53/GOT1/HIF1A/ADRB1 |
| DOID:13042 | persistent fetal circulation syndrome | 0.0260 (2/77) | 0.0013 (13/10086) | 0.003140129887632457 | 0.03524777953311657 | 2 | ABCA1/HIF1A |
| DOID:0081062 | obsolete diabetes | 0.1818 (14/77) | 0.0952 (960/10086) | 0.003183959728818418 | 0.03524777953311657 | 14 | GAD2/FABP3/ADRA2A/MAPK9/ABCA1/HTR2A/RBP4/IGF2R/TP53/LCAT/HIF1A/IMPDH2/ADRB1/MEF2C |
| DOID:0060116 | sensory system cancer | 0.0649 (5/77) | 0.0153 (154/10086) | 0.003286410200477792 | 0.03538518380240472 | 5 | CCNA1/CDK5/TP53/MYC/HIF1A |
| DOID:2174 | ocular cancer | 0.0649 (5/77) | 0.0153 (154/10086) | 0.003286410200477792 | 0.03538518380240472 | 5 | CCNA1/CDK5/TP53/MYC/HIF1A |
| DOID:866 | vein disease | 0.0390 (3/77) | 0.0047 (47/10086) | 0.003533745888182934 | 0.037033656908157145 | 3 | TP53/PCTP/HIF1A |
| DOID:0060074 | ductal carcinoma in situ | 0.0390 (3/77) | 0.0047 (47/10086) | 0.003533745888182934 | 0.037033656908157145 | 3 | RELA/TP53/HIF1A |
| DOID:3963 | thyroid gland carcinoma | 0.0779 (6/77) | 0.0229 (231/10086) | 0.003907116785811488 | 0.03969386509634204 | 6 | CDK5/CCKBR/TP53/MYC/THRB/HIF1A |
| DOID:1240 | leukemia | 0.2078 (16/77) | 0.1193 (1203/10086) | 0.0039343176459575155 | 0.03969386509634204 | 16 | CCNA1/MAPK9/PRDM2/RELA/ABL1/CDK5/PTK2B/CBFB/CCKBR/TP53/MYC/THRB/DUSP2/HIF1A/GSTO2/MEF2C |
| DOID:11981 | morbid obesity | 0.0390 (3/77) | 0.0049 (49/10086) | 0.00397801922107634 | 0.03969386509634204 | 3 | RBP4/ADRB1/HIF1A |
| DOID:3347 | osteosarcoma | 0.0779 (6/77) | 0.0230 (232/10086) | 0.003990552475916407 | 0.03969386509634204 | 6 | PRDM2/RELA/IGF2R/TP53/HIF1A/IMPDH2 |
| DOID:1387 | hypolipoproteinemia | 0.0260 (2/77) | 0.0015 (15/10086) | 0.004191591352412709 | 0.03969386509634204 | 2 | ABCA1/LCAT |
| DOID:0080630 | B-lymphoblastic leukemia/lymphoma | 0.0260 (2/77) | 0.0015 (15/10086) | 0.004191591352412709 | 0.03969386509634204 | 2 | TP53/ABL1 |
| DOID:0080638 | B-cell acute lymphoblastic leukemia | 0.0260 (2/77) | 0.0015 (15/10086) | 0.004191591352412709 | 0.03969386509634204 | 2 | TP53/ABL1 |
| DOID:3948 | adrenocortical carcinoma | 0.0260 (2/77) | 0.0015 (15/10086) | 0.004191591352412709 | 0.03969386509634204 | 2 | TP53/SMARCA2 |
| DOID:10273 | heart conduction disease | 0.0519 (4/77) | 0.0103 (104/10086) | 0.004711636465470228 | 0.04082037336052803 | 4 | CACNB2/SCN2B/RYR2/HIF1A |
| DOID:9408 | acute myocardial infarction | 0.0519 (4/77) | 0.0104 (105/10086) | 0.004874471794767893 | 0.04082037336052803 | 4 | TP53/ADRB1/FABP3/HIF1A |
| DOID:8584 | Burkitt lymphoma | 0.0260 (2/77) | 0.0017 (17/10086) | 0.005383548125670687 | 0.04082037336052803 | 2 | MYC/BCL2L2 |
| DOID:1781 | thyroid gland cancer | 0.0779 (6/77) | 0.0245 (247/10086) | 0.005407420432410198 | 0.04082037336052803 | 6 | CDK5/CCKBR/TP53/MYC/THRB/HIF1A |
| DOID:9742 | pelvic varices | 0.0260 (2/77) | 0.0018 (18/10086) | 0.0060310431801988505 | 0.04082037336052803 | 2 | TP53/HIF1A |
| DOID:660 | adrenal cortex cancer | 0.0260 (2/77) | 0.0018 (18/10086) | 0.0060310431801988505 | 0.04082037336052803 | 2 | TP53/SMARCA2 |
| DOID:12337 | varicocele | 0.0260 (2/77) | 0.0018 (18/10086) | 0.0060310431801988505 | 0.04082037336052803 | 2 | TP53/HIF1A |
| DOID:2154 | nephroblastoma | 0.0519 (4/77) | 0.0112 (113/10086) | 0.006315307932836035 | 0.04082037336052803 | 4 | TP53/MYC/IGF2R/HIF1A |
| DOID:2030 | anxiety disorder | 0.0519 (4/77) | 0.0112 (113/10086) | 0.006315307932836035 | 0.04082037336052803 | 4 | HTR2A/ADRA2A/CAMKK2/CCKBR |
| DOID:0080242 | syndromic X-linked mental retardation Hough type | 0.0130 (1/77) | 0.0001 (1/10086) | 0.006543723973825104 | 0.04082037336052803 | 1 | CNKSR2 |
| DOID:0080957 | primary hypoalphalipoproteinemia 1 | 0.0130 (1/77) | 0.0001 (1/10086) | 0.006543723973825104 | 0.04082037336052803 | 1 | ABCA1 |
| DOID:0080460 | developmental and epileptic encephalopathy 34 | 0.0130 (1/77) | 0.0001 (1/10086) | 0.006543723973825104 | 0.04082037336052803 | 1 | SLC12A5 |
| DOID:0060675 | catecholaminergic polymorphic ventricular tachycardia 1 | 0.0130 (1/77) | 0.0001 (1/10086) | 0.006543723973825104 | 0.04082037336052803 | 1 | RYR2 |
| DOID:0080720 | autosomal dominant congenital deafness with onychodystrophy | 0.0130 (1/77) | 0.0001 (1/10086) | 0.006543723973825104 | 0.04082037336052803 | 1 | ATP6V1B2 |
| DOID:7615 | sarcomatosis | 0.0130 (1/77) | 0.0001 (1/10086) | 0.006543723973825104 | 0.04082037336052803 | 1 | TP53 |
| DOID:139 | squamous cell papilloma | 0.0130 (1/77) | 0.0001 (1/10086) | 0.006543723973825104 | 0.04082037336052803 | 1 | TP53 |
| DOID:1391 | Norum disease | 0.0130 (1/77) | 0.0001 (1/10086) | 0.006543723973825104 | 0.04082037336052803 | 1 | LCAT |
| DOID:4916 | pituitary carcinoma | 0.0130 (1/77) | 0.0001 (1/10086) | 0.006543723973825104 | 0.04082037336052803 | 1 | THRB |
| DOID:7614 | meninges sarcoma | 0.0130 (1/77) | 0.0001 (1/10086) | 0.006543723973825104 | 0.04082037336052803 | 1 | TP53 |
| DOID:0112231 | lissencephaly 7 with cerebellar hypoplasia | 0.0130 (1/77) | 0.0001 (1/10086) | 0.006543723973825104 | 0.04082037336052803 | 1 | CDK5 |
| DOID:0112214 | developmental and epileptic encephalopathy 78 | 0.0130 (1/77) | 0.0001 (1/10086) | 0.006543723973825104 | 0.04082037336052803 | 1 | GABRA2 |
| DOID:0111316 | idiopathic generalized epilepsy 15 | 0.0130 (1/77) | 0.0001 (1/10086) | 0.006543723973825104 | 0.04082037336052803 | 1 | RORB |
| DOID:0111287 | psoriasis 13 | 0.0130 (1/77) | 0.0001 (1/10086) | 0.006543723973825104 | 0.04082037336052803 | 1 | TRAF3IP2 |
| DOID:0111315 | idiopathic generalized epilepsy 14 | 0.0130 (1/77) | 0.0001 (1/10086) | 0.006543723973825104 | 0.04082037336052803 | 1 | SLC12A5 |
| DOID:0111314 | idiopathic generalized epilepsy 13 | 0.0130 (1/77) | 0.0001 (1/10086) | 0.006543723973825104 | 0.04082037336052803 | 1 | GABRA1 |
| DOID:0111503 | Li-Fraumeni syndrome 1 | 0.0130 (1/77) | 0.0001 (1/10086) | 0.006543723973825104 | 0.04082037336052803 | 1 | TP53 |
| DOID:0110221 | Brugada syndrome 4 | 0.0130 (1/77) | 0.0001 (1/10086) | 0.006543723973825104 | 0.04082037336052803 | 1 | CACNB2 |
| DOID:0110909 | inflammatory bowel disease 25 | 0.0130 (1/77) | 0.0001 (1/10086) | 0.006543723973825104 | 0.04082037336052803 | 1 | IL10RB |
| DOID:0112215 | developmental and epileptic encephalopathy 79 | 0.0130 (1/77) | 0.0001 (1/10086) | 0.006543723973825104 | 0.04082037336052803 | 1 | GABRA5 |
| DOID:0080471 | developmental and epileptic encephalopathy 92 | 0.0130 (1/77) | 0.0001 (1/10086) | 0.006543723973825104 | 0.04082037336052803 | 1 | GABRB2 |
| DOID:0080442 | developmental and epileptic encephalopathy 41 | 0.0130 (1/77) | 0.0001 (1/10086) | 0.006543723973825104 | 0.04082037336052803 | 1 | SLC1A2 |
| DOID:0110501 | autosomal recessive nonsyndromic deafness 44 | 0.0130 (1/77) | 0.0001 (1/10086) | 0.006543723973825104 | 0.04082037336052803 | 1 | ADCY1 |
| DOID:0070038 | autosomal dominant intellectual developmental disorder 8 | 0.0130 (1/77) | 0.0001 (1/10086) | 0.006543723973825104 | 0.04082037336052803 | 1 | GRIN1 |
| DOID:0070073 | autosomal dominant intellectual developmental disorder 43 | 0.0130 (1/77) | 0.0001 (1/10086) | 0.006543723973825104 | 0.04082037336052803 | 1 | HIVEP2 |
| DOID:0070097 | oculocutaneous albinism type III | 0.0130 (1/77) | 0.0001 (1/10086) | 0.006543723973825104 | 0.04082037336052803 | 1 | TYRP1 |
| DOID:0070050 | neurodevelopmental disorder with hypotonia, stereotypic hand movements, and impaired language | 0.0130 (1/77) | 0.0001 (1/10086) | 0.006543723973825104 | 0.04082037336052803 | 1 | MEF2C |
| DOID:0070068 | autosomal dominant intellectual developmental disorder 38 | 0.0130 (1/77) | 0.0001 (1/10086) | 0.006543723973825104 | 0.04082037336052803 | 1 | EEF1A2 |
| DOID:0080463 | developmental and epileptic encephalopathy 33 | 0.0130 (1/77) | 0.0001 (1/10086) | 0.006543723973825104 | 0.04082037336052803 | 1 | EEF1A2 |
| DOID:0111374 | selective pituitary thyroid hormone resistance | 0.0130 (1/77) | 0.0001 (1/10086) | 0.006543723973825104 | 0.04082037336052803 | 1 | THRB |
| DOID:0080416 | developmental and epileptic encephalopathy 32 | 0.0130 (1/77) | 0.0001 (1/10086) | 0.006543723973825104 | 0.04082037336052803 | 1 | KCNA2 |
| DOID:0080431 | developmental and epileptic encephalopathy 19 | 0.0130 (1/77) | 0.0001 (1/10086) | 0.006543723973825104 | 0.04082037336052803 | 1 | GABRA1 |
| DOID:0112207 | developmental and epileptic encephalopathy 71 | 0.0130 (1/77) | 0.0001 (1/10086) | 0.006543723973825104 | 0.04082037336052803 | 1 | GLS |
| DOID:0110071 | obsolete arrhythmogenic right ventricular dysplasia 2 | 0.0130 (1/77) | 0.0001 (1/10086) | 0.006543723973825104 | 0.04082037336052803 | 1 | RYR2 |
| DOID:10283 | prostate cancer | 0.1299 (10/77) | 0.0618 (623/10086) | 0.006879152832849176 | 0.042574914382830335 | 10 | CCNA1/MAPK9/RELA/IGF2R/PTK2B/TP53/MYC/THRB/HIF1A/SMARCA2 |
| DOID:0080008 | ischemic bone disease | 0.0390 (3/77) | 0.0060 (61/10086) | 0.007350152216360273 | 0.04422302400136162 | 3 | TP53/HTR2A/HIF1A |
| DOID:9351 | diabetes mellitus | 0.1688 (13/77) | 0.0936 (944/10086) | 0.007361084804658325 | 0.04422302400136162 | 13 | GAD2/FABP3/ADRA2A/MAPK9/ABCA1/RBP4/IGF2R/TP53/LCAT/HIF1A/IMPDH2/ADRB1/MEF2C |
| DOID:4247 | coronary restenosis | 0.0260 (2/77) | 0.0020 (20/10086) | 0.007426767389541646 | 0.04422302400136162 | 2 | TP53/MYC |
| DOID:0050902 | medulloblastoma | 0.0260 (2/77) | 0.0020 (20/10086) | 0.007426767389541646 | 0.04422302400136162 | 2 | TP53/MYC |
| DOID:8632 | Kaposi's sarcoma | 0.0260 (2/77) | 0.0020 (20/10086) | 0.007426767389541646 | 0.04422302400136162 | 2 | MYC/IGF2R |
| DOID:3856 | male reproductive organ cancer | 0.1299 (10/77) | 0.0631 (636/10086) | 0.007919262657038628 | 0.04678357569491705 | 10 | CCNA1/MAPK9/RELA/IGF2R/PTK2B/TP53/MYC/THRB/HIF1A/SMARCA2 |
| DOID:5082 | liver cirrhosis | 0.0909 (7/77) | 0.0350 (353/10086) | 0.008014583813852389 | 0.04678357569491705 | 7 | GLS/ABCA1/RBP4/TP53/GOT1/MYC/VIPR1 |
| DOID:643 | progressive multifocal leukoencephalopathy | 0.0390 (3/77) | 0.0062 (63/10086) | 0.00803534697050102 | 0.04678357569491705 | 3 | TP53/MYC/HIF1A |
| DOID:1542 | head and neck carcinoma | 0.0779 (6/77) | 0.0267 (269/10086) | 0.00811189068555799 | 0.04688195646212192 | 6 | MAPK9/RELA/TP53/MYC/HIF1A/SMARCA2 |
| DOID:4007 | bladder carcinoma | 0.0390 (3/77) | 0.0063 (64/10086) | 0.008391608918245565 | 0.04814455919518988 | 3 | TP53/MYC/IGF2R |
| DOID:1824 | status epilepticus | 0.0519 (4/77) | 0.0122 (123/10086) | 0.008482773945484254 | 0.04831492986341032 | 4 | RELA/MEF2C/PTK2B/GRIN1 |
| DOID:77 | gastrointestinal system disease | 0.2078 (16/77) | 0.1296 (1307/10086) | 0.008784331642100698 | 0.04921453361515655 | 16 | KCNN1/HIVEP2/GLS/RELA/HTR2A/RBP4/ABL1/ABCA1/CCKBR/IL10RB/TP53/GOT1/MYC/VIPR1/HIF1A/ADRB1 |
| DOID:0080639 | bone sarcoma | 0.0779 (6/77) | 0.0272 (274/10086) | 0.00884243211686255 | 0.04921453361515655 | 6 | PRDM2/RELA/IGF2R/TP53/HIF1A/IMPDH2 |
| DOID:0050745 | diffuse large B-cell lymphoma | 0.0260 (2/77) | 0.0022 (22/10086) | 0.008953789194614994 | 0.04921453361515655 | 2 | TP53/BCL2L2 |
| DOID:3953 | adrenal gland cancer | 0.0260 (2/77) | 0.0022 (22/10086) | 0.008953789194614994 | 0.04921453361515655 | 2 | TP53/SMARCA2 |
| DOID:4706 | infratentorial cancer | 0.0260 (2/77) | 0.0022 (22/10086) | 0.008953789194614994 | 0.04921453361515655 | 2 | TP53/MYC |
| DOID:11934 | head and neck cancer | 0.0779 (6/77) | 0.0274 (276/10086) | 0.00914751743573985 | 0.04993019933674668 | 6 | MAPK9/RELA/TP53/MYC/HIF1A/SMARCA2 |

**Table S8. GO and KEGG analysis.**

| **ONTOLOGY** | **ID** | **Description** | **GeneRatio** | **BgRatio** | **pvalue** | **p.adjust** | **qvalue** | **geneID** | **Count** |
| --- | --- | --- | --- | --- | --- | --- | --- | --- | --- |
| BP | GO:0042391 | regulation of membrane potential | 17/77 | 425/18800 | 1.08413E-12 | 2.81765E-09 | 1.97198E-09 | ABL1/ADRB1/CACNB2/GABRA1/GABRA2/GABRB2/GRIN1/KCNA2/KCNH3/KCNJ3/PTK2B/RYR2/SCN2B/MYC/MEF2C/GABRA5/CDK5 | 17 |
| BP | GO:0009410 | response to xenobiotic stimulus | 16/77 | 411/18800 | 8.23905E-12 | 1.07066E-08 | 7.49319E-09 | ABL1/ADCY1/CYP26B1/GAD2/GRIN1/GRM2/GSTO2/HTR2A/NCEH1/PTK2B/SLC12A5/SLC1A2/TP53/TRAF3IP2/MYC/MEF2C | 16 |
| BP | GO:0060078 | regulation of postsynaptic membrane potential | 9/77 | 131/18800 | 3.17348E-09 | 2.74929E-06 | 1.92413E-06 | ADRB1/GABRA1/GABRA2/GABRB2/GRIN1/PTK2B/MEF2C/GABRA5/CDK5 | 9 |
| BP | GO:0001505 | regulation of neurotransmitter levels | 10/77 | 213/18800 | 1.5976E-08 | 1.03804E-05 | 7.2649E-06 | ADCY1/ADRA2A/CACNB2/CASK/GABRA2/GAD2/HTR2A/SLC1A2/MEF2C/CDK5 | 10 |
| BP | GO:0046928 | regulation of neurotransmitter secretion | 7/77 | 86/18800 | 6.0694E-08 | 2.9147E-05 | 2.03989E-05 | ADCY1/ADRA2A/CACNB2/CASK/HTR2A/MEF2C/CDK5 | 7 |
| BP | GO:0015850 | organic hydroxy compound transport | 10/77 | 248/18800 | 6.72882E-08 | 2.9147E-05 | 2.03989E-05 | ADRA2A/GRM2/HTR2A/KCNA2/LCAT/LY6E/MYC/RBP4/LPCAT3/ABCA1 | 10 |
| BP | GO:2000300 | regulation of synaptic vesicle exocytosis | 6/77 | 56/18800 | 1.06837E-07 | 3.65574E-05 | 2.55852E-05 | ADCY1/ADRA2A/CACNB2/CASK/HTR2A/CDK5 | 6 |
| BP | GO:0007611 | learning or memory | 10/77 | 264/18800 | 1.20695E-07 | 3.65574E-05 | 2.55852E-05 | ABL1/ADCY1/GRIN1/HIF1A/HTR2A/PLK2/SLC12A5/MEF2C/GABRA5/CDK5 | 10 |
| BP | GO:0051588 | regulation of neurotransmitter transport | 7/77 | 99/18800 | 1.61176E-07 | 3.65574E-05 | 2.55852E-05 | ADCY1/ADRA2A/CACNB2/CASK/HTR2A/MEF2C/CDK5 | 7 |
| BP | GO:0050804 | modulation of chemical synaptic transmission | 12/77 | 429/18800 | 1.64496E-07 | 3.65574E-05 | 2.55852E-05 | ABL1/ADCY1/ADRA2A/CACNB2/CASK/GRIN1/GRM2/HTR2A/PLK2/PTK2B/MEF2C/CDK5 | 12 |
| BP | GO:0099177 | regulation of trans-synaptic signaling | 12/77 | 430/18800 | 1.68672E-07 | 3.65574E-05 | 2.55852E-05 | ABL1/ADCY1/ADRA2A/CACNB2/CASK/GRIN1/GRM2/HTR2A/PLK2/PTK2B/MEF2C/CDK5 | 12 |
| BP | GO:0006836 | neurotransmitter transport | 9/77 | 207/18800 | 1.68791E-07 | 3.65574E-05 | 2.55852E-05 | ADCY1/ADRA2A/CACNB2/CASK/GABRA2/HTR2A/SLC1A2/MEF2C/CDK5 | 9 |
| BP | GO:0023061 | signal release | 12/77 | 451/18800 | 2.81301E-07 | 5.62386E-05 | 3.93594E-05 | ADCY1/ADRA2A/CACNB2/CASK/GRM2/HIF1A/HTR2A/KCNA2/RBP4/ABCA1/MEF2C/CDK5 | 12 |
| BP | GO:0032526 | response to retinoic acid | 7/77 | 109/18800 | 3.12243E-07 | 5.79656E-05 | 4.05681E-05 | CYP26B1/IGF2R/PTK2B/RORB/MYC/RBP4/ABCA1 | 7 |
| BP | GO:0050890 | cognition | 10/77 | 306/18800 | 4.71755E-07 | 8.11418E-05 | 5.67882E-05 | ABL1/ADCY1/GRIN1/HIF1A/HTR2A/PLK2/SLC12A5/MEF2C/GABRA5/CDK5 | 10 |
| BP | GO:0034765 | regulation of ion transmembrane transport | 12/77 | 476/18800 | 4.99526E-07 | 8.11418E-05 | 5.67882E-05 | ABL1/ADRA2A/CACNB2/KCNA2/KCNH3/KCNJ3/KCNJ6/PTK2B/RYR2/SCN2B/MEF2C/CDK5 | 12 |
| BP | GO:0051402 | neuron apoptotic process | 9/77 | 241/18800 | 6.08696E-07 | 9.30589E-05 | 6.51286E-05 | ABL1/GABRB2/HIF1A/PTK2B/THRB/TP53/MEF2C/GABRA5/CDK5 | 9 |
| BP | GO:0006813 | potassium ion transport | 9/77 | 243/18800 | 6.52241E-07 | 9.41764E-05 | 6.59107E-05 | ADRA2A/HTR2A/KCNA2/KCNH3/KCNJ3/KCNJ6/KCNN1/PTK2B/SLC12A5 | 9 |
| BP | GO:1903522 | regulation of blood circulation | 9/77 | 260/18800 | 1.14514E-06 | 0.000156642 | 0.000109628 | ABL1/ADRA2A/ADRB1/CACNB2/HTR2A/KCNJ3/RYR2/SCN2B/THRB | 9 |
| BP | GO:0042220 | response to cocaine | 5/77 | 47/18800 | 1.35489E-06 | 0.000176068 | 0.000123224 | GRM2/HTR2A/PTK2B/SLC1A2/CDK5 | 5 |
| BP | GO:0043523 | regulation of neuron apoptotic process | 8/77 | 207/18800 | 2.07058E-06 | 0.000243047 | 0.0001701 | ABL1/GABRB2/HIF1A/PTK2B/TP53/MEF2C/GABRA5/CDK5 | 8 |
| BP | GO:0007269 | neurotransmitter secretion | 7/77 | 145/18800 | 2.15086E-06 | 0.000243047 | 0.0001701 | ADCY1/ADRA2A/CACNB2/CASK/HTR2A/MEF2C/CDK5 | 7 |
| BP | GO:0099643 | signal release from synapse | 7/77 | 145/18800 | 2.15086E-06 | 0.000243047 | 0.0001701 | ADCY1/ADRA2A/CACNB2/CASK/HTR2A/MEF2C/CDK5 | 7 |
| BP | GO:0043279 | response to alkaloid | 6/77 | 97/18800 | 2.84732E-06 | 0.000308341 | 0.000215797 | GRM2/HTR2A/PTK2B/RYR2/SLC1A2/CDK5 | 6 |
| BP | GO:0050432 | catecholamine secretion | 5/77 | 57/18800 | 3.58223E-06 | 0.000372409 | 0.000260636 | ADRA2A/GRM2/HTR2A/KCNA2/LY6E | 5 |
| BP | GO:0007214 | gamma-aminobutyric acid signaling pathway | 4/77 | 27/18800 | 4.2494E-06 | 0.000424776 | 0.000297286 | GABRA1/GABRA2/GABRB2/GABRA5 | 4 |
| BP | GO:0016079 | synaptic vesicle exocytosis | 6/77 | 107/18800 | 5.04158E-06 | 0.000485298 | 0.000339643 | ADCY1/ADRA2A/CACNB2/CASK/HTR2A/CDK5 | 6 |
| BP | GO:0010959 | regulation of metal ion transport | 10/77 | 403/18800 | 5.59172E-06 | 0.00051156 | 0.000358022 | ABL1/ADRA2A/CACNB2/CASK/GRIN1/HTR2A/PTK2B/RYR2/SCN2B/CDK5 | 10 |
| BP | GO:0071466 | cellular response to xenobiotic stimulus | 7/77 | 168/18800 | 5.70805E-06 | 0.00051156 | 0.000358022 | CYP26B1/GRIN1/GSTO2/NCEH1/TP53/MYC/MEF2C | 7 |
| BP | GO:1902476 | chloride transmembrane transport | 5/77 | 66/18800 | 7.42958E-06 | 0.000630853 | 0.000441512 | GABRA1/GABRA2/GABRB2/SLC12A5/GABRA5 | 5 |
| BP | GO:0001782 | B cell homeostasis | 4/77 | 31/18800 | 7.5246E-06 | 0.000630853 | 0.000441512 | ABL1/HIF1A/TRAF3IP2/MEF2C | 4 |
| BP | GO:0071300 | cellular response to retinoic acid | 5/77 | 67/18800 | 8.00319E-06 | 0.000650009 | 0.000454918 | CYP26B1/PTK2B/RORB/MYC/ABCA1 | 5 |
| BP | GO:0042752 | regulation of circadian rhythm | 6/77 | 117/18800 | 8.44714E-06 | 0.00065623 | 0.000459272 | ADCY1/ADRB1/KCNA2/MAPK9/RORB/TP53 | 6 |
| BP | GO:0051924 | regulation of calcium ion transport | 8/77 | 251/18800 | 8.58477E-06 | 0.00065623 | 0.000459272 | ABL1/ADRA2A/CACNB2/CASK/GRIN1/PTK2B/RYR2/CDK5 | 8 |
| BP | GO:0051928 | positive regulation of calcium ion transport | 6/77 | 119/18800 | 9.31178E-06 | 0.000691466 | 0.000483932 | ABL1/CACNB2/CASK/GRIN1/RYR2/CDK5 | 6 |
| BP | GO:0051966 | regulation of synaptic transmission, glutamatergic | 5/77 | 70/18800 | 9.93418E-06 | 0.000717192 | 0.000501937 | GRM2/HTR2A/PTK2B/MEF2C/CDK5 | 5 |
| BP | GO:0051937 | catecholamine transport | 5/77 | 72/18800 | 1.14113E-05 | 0.000801568 | 0.000560989 | ADRA2A/GRM2/HTR2A/KCNA2/LY6E | 5 |
| BP | GO:0003206 | cardiac chamber morphogenesis | 6/77 | 126/18800 | 1.29204E-05 | 0.00088369 | 0.000618463 | HIF1A/LY6E/RYR2/TP53/RBP4/MEF2C | 6 |
| BP | GO:0070997 | neuron death | 9/77 | 353/18800 | 1.374E-05 | 0.000915648 | 0.000640829 | ABL1/GABRB2/HIF1A/PTK2B/THRB/TP53/MEF2C/GABRA5/CDK5 | 9 |
| BP | GO:0042596 | fear response | 4/77 | 37/18800 | 1.55027E-05 | 0.001007289 | 0.000704966 | ADRA2A/ADRB1/MEF2C/GABRA5 | 4 |
| BP | GO:0048167 | regulation of synaptic plasticity | 7/77 | 198/18800 | 1.66699E-05 | 0.001056711 | 0.000739554 | ABL1/ADCY1/GRIN1/PLK2/PTK2B/MEF2C/CDK5 | 7 |
| BP | GO:0008306 | associative learning | 5/77 | 80/18800 | 1.91132E-05 | 0.001182744 | 0.00082776 | ABL1/GRIN1/HIF1A/GABRA5/CDK5 | 5 |
| BP | GO:0007623 | circadian rhythm | 7/77 | 205/18800 | 2.08571E-05 | 0.001260643 | 0.000882279 | ADCY1/ADRB1/IMPDH2/KCNA2/MAPK9/RORB/TP53 | 7 |
| BP | GO:0015844 | monoamine transport | 5/77 | 83/18800 | 2.28645E-05 | 0.001350563 | 0.000945211 | ADRA2A/GRM2/HTR2A/KCNA2/LY6E | 5 |
| BP | GO:1903305 | regulation of regulated secretory pathway | 6/77 | 140/18800 | 2.35224E-05 | 0.001358549 | 0.0009508 | ADCY1/ADRA2A/CACNB2/CASK/HTR2A/CDK5 | 6 |
| BP | GO:0007612 | learning | 6/77 | 145/18800 | 2.8678E-05 | 0.001597282 | 0.00111788 | ABL1/GRIN1/HIF1A/SLC12A5/GABRA5/CDK5 | 6 |
| BP | GO:0048511 | rhythmic process | 8/77 | 297/18800 | 2.8885E-05 | 0.001597282 | 0.00111788 | ADCY1/ADRB1/IMPDH2/KCNA2/MAPK9/RORB/TP53/CDK5 | 8 |
| BP | GO:0098661 | inorganic anion transmembrane transport | 5/77 | 90/18800 | 3.38469E-05 | 0.001809558 | 0.001266445 | GABRA1/GABRA2/GABRB2/SLC12A5/GABRA5 | 5 |
| BP | GO:1902895 | positive regulation of miRNA transcription | 4/77 | 45/18800 | 3.41163E-05 | 0.001809558 | 0.001266445 | HIF1A/TP53/MYC/RELA | 4 |
| BP | GO:1904862 | inhibitory synapse assembly | 3/77 | 16/18800 | 3.56017E-05 | 0.001850575 | 0.001295151 | GABRA1/GABRA2/GABRB2 | 3 |
| BP | GO:0009416 | response to light stimulus | 8/77 | 310/18800 | 3.91552E-05 | 0.001995382 | 0.001396497 | GRIN1/HIF1A/PITPNM1/SLC1A2/TP53/MYC/RELA/CDK5 | 8 |
| BP | GO:0035249 | synaptic transmission, glutamatergic | 5/77 | 94/18800 | 4.17385E-05 | 0.002043344 | 0.001430064 | GRM2/HTR2A/PTK2B/MEF2C/CDK5 | 5 |
| BP | GO:1901214 | regulation of neuron death | 8/77 | 313/18800 | 4.19151E-05 | 0.002043344 | 0.001430064 | ABL1/GABRB2/HIF1A/PTK2B/TP53/MEF2C/GABRA5/CDK5 | 8 |
| BP | GO:0070588 | calcium ion transmembrane transport | 8/77 | 314/18800 | 4.28705E-05 | 0.002043344 | 0.001430064 | ABL1/ADRA2A/CACNB2/GRIN1/HTR2A/PTK2B/RYR2/CDK5 | 8 |
| BP | GO:1903169 | regulation of calcium ion transmembrane transport | 6/77 | 156/18800 | 4.32412E-05 | 0.002043344 | 0.001430064 | ABL1/ADRA2A/CACNB2/PTK2B/RYR2/CDK5 | 6 |
| BP | GO:0007632 | visual behavior | 4/77 | 48/18800 | 4.41417E-05 | 0.00204865 | 0.001433777 | GRIN1/HIF1A/SLC1A2/CDK5 | 4 |
| BP | GO:0050806 | positive regulation of synaptic transmission | 6/77 | 161/18800 | 5.15724E-05 | 0.002351522 | 0.001645746 | ABL1/ADCY1/CACNB2/PLK2/PTK2B/CDK5 | 6 |
| BP | GO:0007204 | positive regulation of cytosolic calcium ion concentration | 8/77 | 325/18800 | 5.46375E-05 | 0.002448327 | 0.001713496 | ABL1/CACNB2/CCKBR/GRIN1/HTR2A/PTK2B/RYR2/CDK5 | 8 |
| BP | GO:0051932 | synaptic transmission, GABAergic | 4/77 | 51/18800 | 5.61667E-05 | 0.002474193 | 0.001731599 | GABRA1/GABRA2/GABRB2/GABRA5 | 4 |
| BP | GO:0006816 | calcium ion transport | 9/77 | 424/18800 | 5.7653E-05 | 0.002497337 | 0.001747797 | ABL1/ADRA2A/CACNB2/CASK/GRIN1/HTR2A/PTK2B/RYR2/CDK5 | 9 |
| BP | GO:0086014 | atrial cardiac muscle cell action potential | 3/77 | 19/18800 | 6.10607E-05 | 0.002518998 | 0.001762957 | CACNB2/KCNJ3/RYR2 | 3 |
| BP | GO:0086026 | atrial cardiac muscle cell to AV node cell signaling | 3/77 | 19/18800 | 6.10607E-05 | 0.002518998 | 0.001762957 | CACNB2/KCNJ3/RYR2 | 3 |
| BP | GO:0086066 | atrial cardiac muscle cell to AV node cell communication | 3/77 | 19/18800 | 6.10607E-05 | 0.002518998 | 0.001762957 | CACNB2/KCNJ3/RYR2 | 3 |
| BP | GO:0007188 | adenylate cyclase-modulating G protein-coupled receptor signaling pathway | 7/77 | 244/18800 | 6.32082E-05 | 0.002527689 | 0.00176904 | ADCY1/ADRA2A/ADRB1/GRM2/PTH2R/VIPR1/ABCA1 | 7 |
| BP | GO:0003205 | cardiac chamber development | 6/77 | 167/18800 | 6.32165E-05 | 0.002527689 | 0.00176904 | HIF1A/LY6E/RYR2/TP53/RBP4/MEF2C | 6 |
| BP | GO:0098656 | anion transmembrane transport | 7/77 | 247/18800 | 6.82562E-05 | 0.002659319 | 0.001861162 | GABRA1/GABRA2/GABRB2/SLC12A5/SLC1A2/MYC/GABRA5 | 7 |
| BP | GO:0010506 | regulation of autophagy | 8/77 | 336/18800 | 6.89849E-05 | 0.002659319 | 0.001861162 | ABL1/ATP6V1B2/CAMKK2/EEF1A2/HIF1A/PLK2/TP53/CDK5 | 8 |
| BP | GO:0086002 | cardiac muscle cell action potential involved in contraction | 4/77 | 54/18800 | 7.04219E-05 | 0.002659319 | 0.001861162 | CACNB2/KCNJ3/RYR2/SCN2B | 4 |
| BP | GO:0002027 | regulation of heart rate | 5/77 | 105/18800 | 7.08978E-05 | 0.002659319 | 0.001861162 | ADRB1/CACNB2/KCNJ3/RYR2/SCN2B | 5 |
| BP | GO:0045821 | positive regulation of glycolytic process | 3/77 | 20/18800 | 7.16246E-05 | 0.002659319 | 0.001861162 | HIF1A/HTR2A/MYC | 3 |
| BP | GO:0043470 | regulation of carbohydrate catabolic process | 4/77 | 55/18800 | 7.57102E-05 | 0.002771422 | 0.001939619 | HIF1A/HTR2A/TP53/MYC | 4 |
| BP | GO:0050433 | regulation of catecholamine secretion | 4/77 | 56/18800 | 8.12819E-05 | 0.002934052 | 0.002053438 | ADRA2A/GRM2/HTR2A/KCNA2 | 4 |
| BP | GO:0006821 | chloride transport | 5/77 | 111/18800 | 9.23234E-05 | 0.003286965 | 0.00230043 | GABRA1/GABRA2/GABRB2/SLC12A5/GABRA5 | 5 |
| BP | GO:1904062 | regulation of cation transmembrane transport | 8/77 | 352/18800 | 9.53499E-05 | 0.003333202 | 0.002332789 | ABL1/ADRA2A/CACNB2/PTK2B/RYR2/SCN2B/MEF2C/CDK5 | 8 |
| BP | GO:0044346 | fibroblast apoptotic process | 3/77 | 22/18800 | 9.61871E-05 | 0.003333202 | 0.002332789 | STK17B/TP53/MYC | 3 |
| BP | GO:1902893 | regulation of miRNA transcription | 4/77 | 59/18800 | 9.97887E-05 | 0.003411335 | 0.002387471 | HIF1A/TP53/MYC/RELA | 4 |
| BP | GO:0032412 | regulation of ion transmembrane transporter activity | 7/77 | 263/18800 | 0.000101067 | 0.003411335 | 0.002387471 | ADRA2A/CACNB2/PTK2B/RYR2/SCN2B/MEF2C/CDK5 | 7 |
| BP | GO:0051480 | regulation of cytosolic calcium ion concentration | 8/77 | 356/18800 | 0.000103107 | 0.003435593 | 0.002404449 | ABL1/CACNB2/CCKBR/GRIN1/HTR2A/PTK2B/RYR2/CDK5 | 8 |
| BP | GO:0099504 | synaptic vesicle cycle | 6/77 | 183/18800 | 0.000104781 | 0.003447169 | 0.00241255 | ADCY1/ADRA2A/CACNB2/CASK/HTR2A/CDK5 | 6 |
| BP | GO:0061614 | miRNA transcription | 4/77 | 60/18800 | 0.000106586 | 0.003462707 | 0.002423425 | HIF1A/TP53/MYC/RELA | 4 |
| BP | GO:1901522 | positive regulation of transcription from RNA polymerase II promoter involved in cellular response to chemical stimulus | 3/77 | 24/18800 | 0.000125674 | 0.003989657 | 0.002792218 | HIF1A/TP53/RELA | 3 |
| BP | GO:0022898 | regulation of transmembrane transporter activity | 7/77 | 273/18800 | 0.000127411 | 0.003989657 | 0.002792218 | ADRA2A/CACNB2/PTK2B/RYR2/SCN2B/MEF2C/CDK5 | 7 |
| BP | GO:0043270 | positive regulation of ion transport | 7/77 | 273/18800 | 0.000127411 | 0.003989657 | 0.002792218 | ABL1/ADRA2A/CACNB2/CASK/GRIN1/RYR2/CDK5 | 7 |
| BP | GO:0002260 | lymphocyte homeostasis | 4/77 | 65/18800 | 0.000145712 | 0.004508411 | 0.003155276 | ABL1/HIF1A/TRAF3IP2/MEF2C | 4 |
| BP | GO:0099003 | vesicle-mediated transport in synapse | 6/77 | 197/18800 | 0.000156794 | 0.004794202 | 0.003355291 | ADCY1/ADRA2A/CACNB2/CASK/HTR2A/CDK5 | 6 |
| BP | GO:0034377 | plasma lipoprotein particle assembly | 3/77 | 26/18800 | 0.00016049 | 0.004850169 | 0.00339446 | LCAT/LPCAT3/ABCA1 | 3 |
| BP | GO:0009065 | glutamine family amino acid catabolic process | 3/77 | 27/18800 | 0.000180021 | 0.005377854 | 0.003763768 | GAD2/GLS/GOT1 | 3 |
| BP | GO:0000302 | response to reactive oxygen species | 6/77 | 203/18800 | 0.000184542 | 0.005450285 | 0.00381446 | ABL1/CAMKK2/HIF1A/MAPK9/PTK2B/RELA | 6 |
| BP | GO:0017157 | regulation of exocytosis | 6/77 | 204/18800 | 0.000189522 | 0.005534465 | 0.003873375 | ADCY1/ADRA2A/CACNB2/CASK/HTR2A/CDK5 | 6 |
| BP | GO:0006869 | lipid transport | 8/77 | 391/18800 | 0.000196104 | 0.00566305 | 0.003963367 | LCAT/PCTP/PITPNM1/SLC27A2/RBP4/FABP3/LPCAT3/ABCA1 | 8 |
| BP | GO:0030431 | sleep | 3/77 | 28/18800 | 0.00020103 | 0.005741502 | 0.004018272 | ADRB1/HTR2A/KCNA2 | 3 |
| BP | GO:1904427 | positive regulation of calcium ion transmembrane transport | 4/77 | 71/18800 | 0.000205269 | 0.005798836 | 0.004058399 | ABL1/CACNB2/RYR2/CDK5 | 4 |
| BP | GO:1901215 | negative regulation of neuron death | 6/77 | 209/18800 | 0.000216037 | 0.005990191 | 0.004192321 | GABRB2/HIF1A/PTK2B/MEF2C/GABRA5/CDK5 | 6 |
| BP | GO:0003208 | cardiac ventricle morphogenesis | 4/77 | 72/18800 | 0.000216672 | 0.005990191 | 0.004192321 | HIF1A/LY6E/RYR2/MEF2C | 4 |
| BP | GO:0043467 | regulation of generation of precursor metabolites and energy | 5/77 | 134/18800 | 0.000223541 | 0.005990191 | 0.004192321 | HIF1A/HTR2A/IL10RB/TP53/MYC | 5 |
| BP | GO:0006656 | phosphatidylcholine biosynthetic process | 3/77 | 29/18800 | 0.000223566 | 0.005990191 | 0.004192321 | LCAT/FABP3/LPCAT3 | 3 |
| BP | GO:0065005 | protein-lipid complex assembly | 3/77 | 29/18800 | 0.000223566 | 0.005990191 | 0.004192321 | LCAT/LPCAT3/ABCA1 | 3 |
| BP | GO:0008016 | regulation of heart contraction | 6/77 | 211/18800 | 0.000227428 | 0.00599935 | 0.004198731 | ADRB1/CACNB2/KCNJ3/RYR2/SCN2B/THRB | 6 |
| BP | GO:0033555 | multicellular organismal response to stress | 4/77 | 73/18800 | 0.000228525 | 0.00599935 | 0.004198731 | ADRA2A/ADRB1/MEF2C/GABRA5 | 4 |
| BP | GO:0032409 | regulation of transporter activity | 7/77 | 305/18800 | 0.000251473 | 0.006526323 | 0.00456754 | ADRA2A/CACNB2/PTK2B/RYR2/SCN2B/MEF2C/CDK5 | 7 |
| BP | GO:0086003 | cardiac muscle cell contraction | 4/77 | 75/18800 | 0.00025362 | 0.006526323 | 0.00456754 | CACNB2/KCNJ3/RYR2/SCN2B | 4 |
| BP | GO:0071805 | potassium ion transmembrane transport | 6/77 | 219/18800 | 0.000277816 | 0.007078856 | 0.004954239 | KCNA2/KCNH3/KCNJ3/KCNJ6/KCNN1/SLC12A5 | 6 |
| BP | GO:0086001 | cardiac muscle cell action potential | 4/77 | 77/18800 | 0.000280638 | 0.00708135 | 0.004955984 | CACNB2/KCNJ3/RYR2/SCN2B | 4 |
| BP | GO:0001508 | action potential | 5/77 | 143/18800 | 0.000302125 | 0.007550212 | 0.005284124 | CACNB2/KCNA2/KCNJ3/RYR2/SCN2B | 5 |
| BP | GO:0050808 | synapse organization | 8/77 | 419/18800 | 0.000312696 | 0.007686955 | 0.005379826 | ABL1/CACNB2/CNKSR2/GABRA1/GABRA2/GABRB2/MEF2C/CDK5 | 8 |
| BP | GO:0046777 | protein autophosphorylation | 6/77 | 224/18800 | 0.000313512 | 0.007686955 | 0.005379826 | ABL1/CAMKK2/PTK2B/STK10/STK17B/CDK5 | 6 |
| BP | GO:0043524 | negative regulation of neuron apoptotic process | 5/77 | 145/18800 | 0.000322119 | 0.007824181 | 0.005475865 | GABRB2/HIF1A/PTK2B/MEF2C/GABRA5 | 5 |
| BP | GO:0006536 | glutamate metabolic process | 3/77 | 34/18800 | 0.000360775 | 0.008524131 | 0.005965735 | GAD2/GLS/GOT1 | 3 |
| BP | GO:0034405 | response to fluid shear stress | 3/77 | 34/18800 | 0.000360775 | 0.008524131 | 0.005965735 | PTK2B/ABCA1/MEF2C | 3 |
| BP | GO:0086019 | cell-cell signaling involved in cardiac conduction | 3/77 | 34/18800 | 0.000360775 | 0.008524131 | 0.005965735 | CACNB2/KCNJ3/RYR2 | 3 |
| BP | GO:0060041 | retina development in camera-type eye | 5/77 | 149/18800 | 0.000365102 | 0.008548656 | 0.0059829 | HIF1A/IMPDH2/RORB/THRB/RBP4 | 5 |
| BP | GO:1903131 | mononuclear cell differentiation | 8/77 | 433/18800 | 0.000389388 | 0.009006129 | 0.006303068 | ABL1/CBFB/CYP26B1/PTK2B/TP53/TRAF3IP2/MYC/JAG2 | 8 |
| BP | GO:0097006 | regulation of plasma lipoprotein particle levels | 4/77 | 84/18800 | 0.000391571 | 0.009006129 | 0.006303068 | LCAT/NCEH1/LPCAT3/ABCA1 | 4 |
| BP | GO:0045055 | regulated exocytosis | 6/77 | 234/18800 | 0.000395662 | 0.0090204 | 0.006313056 | ADCY1/ADRA2A/CACNB2/CASK/HTR2A/CDK5 | 6 |
| BP | GO:0016052 | carbohydrate catabolic process | 5/77 | 152/18800 | 0.000400086 | 0.009041947 | 0.006328136 | DERA/HIF1A/HTR2A/TP53/MYC | 5 |
| BP | GO:0062197 | cellular response to chemical stress | 7/77 | 332/18800 | 0.000419446 | 0.009335059 | 0.006533275 | ABL1/CAMKK2/HIF1A/MAPK9/TP53/MYC/RELA | 7 |
| BP | GO:0009314 | response to radiation | 8/77 | 438/18800 | 0.000420239 | 0.009335059 | 0.006533275 | GRIN1/HIF1A/PITPNM1/SLC1A2/TP53/MYC/RELA/CDK5 | 8 |
| BP | GO:0097553 | calcium ion transmembrane import into cytosol | 5/77 | 154/18800 | 0.000424778 | 0.009346453 | 0.006541249 | ABL1/GRIN1/HTR2A/PTK2B/RYR2 | 5 |
| BP | GO:1903146 | regulation of autophagy of mitochondrion | 3/77 | 36/18800 | 0.000427945 | 0.009346453 | 0.006541249 | CAMKK2/HIF1A/TP53 | 3 |
| BP | GO:0098657 | import into cell | 6/77 | 238/18800 | 0.00043288 | 0.009375463 | 0.006561552 | KCNJ3/KCNJ6/SLC12A5/SLC1A2/SLC27A2/FABP3 | 6 |
| BP | GO:0014046 | dopamine secretion | 3/77 | 37/18800 | 0.000464336 | 0.009654484 | 0.006756829 | GRM2/HTR2A/KCNA2 | 3 |
| BP | GO:0014059 | regulation of dopamine secretion | 3/77 | 37/18800 | 0.000464336 | 0.009654484 | 0.006756829 | GRM2/HTR2A/KCNA2 | 3 |
| BP | GO:0030279 | negative regulation of ossification | 3/77 | 37/18800 | 0.000464336 | 0.009654484 | 0.006756829 | HIF1A/PTK2B/MEF2C | 3 |
| BP | GO:1901021 | positive regulation of calcium ion transmembrane transporter activity | 3/77 | 37/18800 | 0.000464336 | 0.009654484 | 0.006756829 | CACNB2/RYR2/CDK5 | 3 |
| BP | GO:1903580 | positive regulation of ATP metabolic process | 3/77 | 37/18800 | 0.000464336 | 0.009654484 | 0.006756829 | HIF1A/HTR2A/MYC | 3 |
| BP | GO:0010876 | lipid localization | 8/77 | 446/18800 | 0.000473726 | 0.00977154 | 0.006838752 | LCAT/PCTP/PITPNM1/SLC27A2/RBP4/FABP3/LPCAT3/ABCA1 | 8 |
| BP | GO:0001776 | leukocyte homeostasis | 4/77 | 89/18800 | 0.000487907 | 0.009906801 | 0.006933417 | ABL1/HIF1A/TRAF3IP2/MEF2C | 4 |
| BP | GO:1903578 | regulation of ATP metabolic process | 4/77 | 89/18800 | 0.000487907 | 0.009906801 | 0.006933417 | HIF1A/HTR2A/TP53/MYC | 4 |
| BP | GO:0031331 | positive regulation of cellular catabolic process | 8/77 | 449/18800 | 0.000495158 | 0.009976092 | 0.006981911 | ADRA2A/CAMKK2/HIF1A/HTR2A/MAPK9/PLK2/PTK2B/MYC | 8 |
| BP | GO:0062012 | regulation of small molecule metabolic process | 7/77 | 342/18800 | 0.000500705 | 0.010010045 | 0.007005673 | HIF1A/HTR2A/PTK2B/TP53/MYC/FABP3/LPCAT3 | 7 |
| BP | GO:0045926 | negative regulation of growth | 6/77 | 245/18800 | 0.000504546 | 0.010010045 | 0.007005673 | ADRB1/HIF1A/SMARCA2/TP53/RBP4/CDK5 | 6 |
| BP | GO:0060047 | heart contraction | 6/77 | 247/18800 | 0.000526631 | 0.010294916 | 0.007205044 | ADRB1/CACNB2/KCNJ3/RYR2/SCN2B/THRB | 6 |
| BP | GO:0090398 | cellular senescence | 4/77 | 91/18800 | 0.000530788 | 0.010294916 | 0.007205044 | ABL1/MAPK9/PLK2/TP53 | 4 |
| BP | GO:1901019 | regulation of calcium ion transmembrane transporter activity | 4/77 | 91/18800 | 0.000530788 | 0.010294916 | 0.007205044 | ADRA2A/CACNB2/RYR2/CDK5 | 4 |
| BP | GO:0055081 | anion homeostasis | 3/77 | 39/18800 | 0.000542945 | 0.010375844 | 0.007261683 | GLS/GRM2/SLC12A5 | 3 |
| BP | GO:0086091 | regulation of heart rate by cardiac conduction | 3/77 | 39/18800 | 0.000542945 | 0.010375844 | 0.007261683 | CACNB2/KCNJ3/SCN2B | 3 |
| BP | GO:0006874 | cellular calcium ion homeostasis | 8/77 | 456/18800 | 0.000548244 | 0.01040063 | 0.00727903 | ABL1/CACNB2/CCKBR/GRIN1/HTR2A/PTK2B/RYR2/CDK5 | 8 |
| BP | GO:0051952 | regulation of amine transport | 4/77 | 92/18800 | 0.000553204 | 0.010418683 | 0.007291665 | ADRA2A/GRM2/HTR2A/KCNA2 | 4 |
| BP | GO:0035637 | multicellular organismal signaling | 5/77 | 164/18800 | 0.000565919 | 0.010581473 | 0.007405595 | CACNB2/KCNA2/KCNJ3/RYR2/SCN2B | 5 |
| BP | GO:0010038 | response to metal ion | 7/77 | 351/18800 | 0.000584161 | 0.010787595 | 0.007549853 | ADCY1/HIF1A/LCAT/MAPK9/PTK2B/RYR2/MEF2C | 7 |
| BP | GO:0021587 | cerebellum morphogenesis | 3/77 | 40/18800 | 0.000585245 | 0.010787595 | 0.007549853 | ABL1/KNDC1/CDK5 | 3 |
| BP | GO:0003007 | heart morphogenesis | 6/77 | 254/18800 | 0.000609919 | 0.01116324 | 0.007812754 | HIF1A/LY6E/RYR2/TP53/RBP4/MEF2C | 6 |
| BP | GO:0060079 | excitatory postsynaptic potential | 4/77 | 95/18800 | 0.000624489 | 0.01134997 | 0.007943439 | GRIN1/PTK2B/MEF2C/CDK5 | 4 |
| BP | GO:0003015 | heart process | 6/77 | 257/18800 | 0.000648599 | 0.011649725 | 0.008153227 | ADRB1/CACNB2/KCNJ3/RYR2/SCN2B/THRB | 6 |
| BP | GO:0055074 | calcium ion homeostasis | 8/77 | 468/18800 | 0.000649946 | 0.011649725 | 0.008153227 | ABL1/CACNB2/CCKBR/GRIN1/HTR2A/PTK2B/RYR2/CDK5 | 8 |
| BP | GO:0008542 | visual learning | 3/77 | 42/18800 | 0.000676035 | 0.011952485 | 0.008365118 | GRIN1/HIF1A/CDK5 | 3 |
| BP | GO:2000249 | regulation of actin cytoskeleton reorganization | 3/77 | 42/18800 | 0.000676035 | 0.011952485 | 0.008365118 | ABL1/PTK2B/CDK5 | 3 |
| BP | GO:0060402 | calcium ion transport into cytosol | 5/77 | 171/18800 | 0.000683883 | 0.012009543 | 0.008405051 | ABL1/GRIN1/HTR2A/PTK2B/RYR2 | 5 |
| BP | GO:0021549 | cerebellum development | 4/77 | 98/18800 | 0.00070206 | 0.012035314 | 0.008423087 | ABL1/TP53/KNDC1/CDK5 | 4 |
| BP | GO:0060291 | long-term synaptic potentiation | 4/77 | 98/18800 | 0.00070206 | 0.012035314 | 0.008423087 | ABL1/ADCY1/PLK2/PTK2B | 4 |
| BP | GO:0061337 | cardiac conduction | 4/77 | 98/18800 | 0.00070206 | 0.012035314 | 0.008423087 | CACNB2/KCNJ3/RYR2/SCN2B | 4 |
| BP | GO:0010469 | regulation of signaling receptor activity | 5/77 | 173/18800 | 0.000720739 | 0.012035314 | 0.008423087 | ADRA2A/HIF1A/LY6E/PTK2B/MEF2C | 5 |
| BP | GO:0021575 | hindbrain morphogenesis | 3/77 | 43/18800 | 0.000724605 | 0.012035314 | 0.008423087 | ABL1/KNDC1/CDK5 | 3 |
| BP | GO:0048512 | circadian behavior | 3/77 | 43/18800 | 0.000724605 | 0.012035314 | 0.008423087 | ADRB1/KCNA2/TP53 | 3 |
| BP | GO:0019233 | sensory perception of pain | 4/77 | 99/18800 | 0.000729367 | 0.012035314 | 0.008423087 | HTR2A/KCNA2/MGLL/CDK5 | 4 |
| BP | GO:0071073 | positive regulation of phospholipid biosynthetic process | 2/77 | 10/18800 | 0.000729424 | 0.012035314 | 0.008423087 | HTR2A/FABP3 | 2 |
| BP | GO:1901387 | positive regulation of voltage-gated calcium channel activity | 2/77 | 10/18800 | 0.000729424 | 0.012035314 | 0.008423087 | CACNB2/CDK5 | 2 |
| BP | GO:0030217 | T cell differentiation | 6/77 | 263/18800 | 0.000731658 | 0.012035314 | 0.008423087 | ABL1/CBFB/CYP26B1/TP53/TRAF3IP2/JAG2 | 6 |
| BP | GO:0048771 | tissue remodeling | 5/77 | 174/18800 | 0.000739714 | 0.012091304 | 0.008462272 | HIF1A/PTK2B/RSPO3/TP53/MEF2C | 5 |
| BP | GO:0015837 | amine transport | 4/77 | 100/18800 | 0.000757417 | 0.012253418 | 0.00857573 | ADRA2A/GRM2/HTR2A/KCNA2 | 4 |
| BP | GO:0043534 | blood vessel endothelial cell migration | 5/77 | 175/18800 | 0.000759061 | 0.012253418 | 0.00857573 | ABL1/HIF1A/PLK2/PTK2B/MEF2C | 5 |
| BP | GO:0045981 | positive regulation of nucleotide metabolic process | 3/77 | 44/18800 | 0.000775344 | 0.012362688 | 0.008652204 | HIF1A/HTR2A/MYC | 3 |
| BP | GO:1900544 | positive regulation of purine nucleotide metabolic process | 3/77 | 44/18800 | 0.000775344 | 0.012362688 | 0.008652204 | HIF1A/HTR2A/MYC | 3 |
| BP | GO:0099565 | chemical synaptic transmission, postsynaptic | 4/77 | 101/18800 | 0.000786222 | 0.012459697 | 0.008720097 | GRIN1/PTK2B/MEF2C/CDK5 | 4 |
| BP | GO:1903706 | regulation of hemopoiesis | 7/77 | 371/18800 | 0.000809438 | 0.012627912 | 0.008837825 | ABL1/CBFB/CYP26B1/HIF1A/PTK2B/MYC/MEF2C | 7 |
| BP | GO:0006110 | regulation of glycolytic process | 3/77 | 45/18800 | 0.000828288 | 0.012627912 | 0.008837825 | HIF1A/HTR2A/MYC | 3 |
| BP | GO:0007622 | rhythmic behavior | 3/77 | 45/18800 | 0.000828288 | 0.012627912 | 0.008837825 | ADRB1/KCNA2/TP53 | 3 |
| BP | GO:0071827 | plasma lipoprotein particle organization | 3/77 | 45/18800 | 0.000828288 | 0.012627912 | 0.008837825 | LCAT/LPCAT3/ABCA1 | 3 |
| BP | GO:0009135 | purine nucleoside diphosphate metabolic process | 4/77 | 103/18800 | 0.00084614 | 0.012627912 | 0.008837825 | CASK/HIF1A/HTR2A/MYC | 4 |
| BP | GO:0009179 | purine ribonucleoside diphosphate metabolic process | 4/77 | 103/18800 | 0.00084614 | 0.012627912 | 0.008837825 | CASK/HIF1A/HTR2A/MYC | 4 |
| BP | GO:0010522 | regulation of calcium ion transport into cytosol | 4/77 | 103/18800 | 0.00084614 | 0.012627912 | 0.008837825 | ABL1/GRIN1/PTK2B/RYR2 | 4 |
| BP | GO:0070252 | actin-mediated cell contraction | 4/77 | 103/18800 | 0.00084614 | 0.012627912 | 0.008837825 | CACNB2/KCNJ3/RYR2/SCN2B | 4 |
| BP | GO:0007416 | synapse assembly | 5/77 | 180/18800 | 0.00086153 | 0.012627912 | 0.008837825 | GABRA1/GABRA2/GABRB2/MEF2C/CDK5 | 5 |
| BP | GO:0044282 | small molecule catabolic process | 7/77 | 376/18800 | 0.000875296 | 0.012627912 | 0.008837825 | CYP26B1/DERA/GAD2/GLS/SLC27A2/TP53/GOT1 | 7 |
| BP | GO:0022600 | digestive system process | 4/77 | 104/18800 | 0.000877276 | 0.012627912 | 0.008837825 | ADRA2A/CCKBR/RBP4/LPCAT3 | 4 |
| BP | GO:0002327 | immature B cell differentiation | 2/77 | 11/18800 | 0.000889153 | 0.012627912 | 0.008837825 | ABL1/TRAF3IP2 | 2 |
| BP | GO:0042670 | retinal cone cell differentiation | 2/77 | 11/18800 | 0.000889153 | 0.012627912 | 0.008837825 | RORB/THRB | 2 |
| BP | GO:0046549 | retinal cone cell development | 2/77 | 11/18800 | 0.000889153 | 0.012627912 | 0.008837825 | RORB/THRB | 2 |
| BP | GO:0048241 | epinephrine transport | 2/77 | 11/18800 | 0.000889153 | 0.012627912 | 0.008837825 | ADRA2A/LY6E | 2 |
| BP | GO:0070243 | regulation of thymocyte apoptotic process | 2/77 | 11/18800 | 0.000889153 | 0.012627912 | 0.008837825 | HIF1A/TP53 | 2 |
| BP | GO:0086016 | AV node cell action potential | 2/77 | 11/18800 | 0.000889153 | 0.012627912 | 0.008837825 | CACNB2/RYR2 | 2 |
| BP | GO:0086027 | AV node cell to bundle of His cell signaling | 2/77 | 11/18800 | 0.000889153 | 0.012627912 | 0.008837825 | CACNB2/RYR2 | 2 |
| BP | GO:0099171 | presynaptic modulation of chemical synaptic transmission | 2/77 | 11/18800 | 0.000889153 | 0.012627912 | 0.008837825 | CACNB2/CDK5 | 2 |
| BP | GO:0015698 | inorganic anion transport | 5/77 | 182/18800 | 0.00090528 | 0.012787078 | 0.00894922 | GABRA1/GABRA2/GABRB2/SLC12A5/GABRA5 | 5 |
| BP | GO:0072503 | cellular divalent inorganic cation homeostasis | 8/77 | 494/18800 | 0.000923003 | 0.012963781 | 0.009072888 | ABL1/CACNB2/CCKBR/GRIN1/HTR2A/PTK2B/RYR2/CDK5 | 8 |
| BP | GO:0006109 | regulation of carbohydrate metabolic process | 5/77 | 183/18800 | 0.000927766 | 0.012963781 | 0.009072888 | HIF1A/HTR2A/PTK2B/TP53/MYC | 5 |
| BP | GO:0030098 | lymphocyte differentiation | 7/77 | 382/18800 | 0.000959847 | 0.013340329 | 0.00933642 | ABL1/CBFB/CYP26B1/PTK2B/TP53/TRAF3IP2/JAG2 | 7 |
| BP | GO:0022037 | metencephalon development | 4/77 | 107/18800 | 0.000975528 | 0.013414794 | 0.009388536 | ABL1/TP53/KNDC1/CDK5 | 4 |
| BP | GO:0033209 | tumor necrosis factor-mediated signaling pathway | 4/77 | 107/18800 | 0.000975528 | 0.013414794 | 0.009388536 | PTK2B/TP53/TRAF3IP2/RELA | 4 |
| BP | GO:0071825 | protein-lipid complex subunit organization | 3/77 | 48/18800 | 0.001000734 | 0.013688985 | 0.009580432 | LCAT/LPCAT3/ABCA1 | 3 |
| BP | GO:0006805 | xenobiotic metabolic process | 4/77 | 108/18800 | 0.00100993 | 0.013742449 | 0.00961785 | CYP26B1/GRIN1/GSTO2/NCEH1 | 4 |
| BP | GO:0071887 | leukocyte apoptotic process | 4/77 | 109/18800 | 0.001045177 | 0.014147994 | 0.009901677 | HIF1A/TP53/TRAF3IP2/MEF2C | 4 |
| BP | GO:2000772 | regulation of cellular senescence | 3/77 | 49/18800 | 0.001062874 | 0.014256356 | 0.009977515 | ABL1/PLK2/TP53 | 3 |
| BP | GO:0086067 | AV node cell to bundle of His cell communication | 2/77 | 12/18800 | 0.001064153 | 0.014256356 | 0.009977515 | CACNB2/RYR2 | 2 |
| BP | GO:0034599 | cellular response to oxidative stress | 6/77 | 284/18800 | 0.001088899 | 0.014472808 | 0.010129002 | ABL1/CAMKK2/HIF1A/MAPK9/TP53/RELA | 6 |
| BP | GO:0050731 | positive regulation of peptidyl-tyrosine phosphorylation | 5/77 | 190/18800 | 0.001097015 | 0.014472808 | 0.010129002 | ABL1/ADRA2A/HTR2A/PTK2B/TP53 | 5 |
| BP | GO:0060401 | cytosolic calcium ion transport | 5/77 | 190/18800 | 0.001097015 | 0.014472808 | 0.010129002 | ABL1/GRIN1/HTR2A/PTK2B/RYR2 | 5 |
| BP | GO:0043457 | regulation of cellular respiration | 3/77 | 50/18800 | 0.001127403 | 0.014724222 | 0.010304958 | HIF1A/IL10RB/MYC | 3 |
| BP | GO:1990573 | potassium ion import across plasma membrane | 3/77 | 50/18800 | 0.001127403 | 0.014724222 | 0.010304958 | KCNJ3/KCNJ6/SLC12A5 | 3 |
| BP | GO:0009185 | ribonucleoside diphosphate metabolic process | 4/77 | 112/18800 | 0.001156099 | 0.01502351 | 0.010514419 | CASK/HIF1A/HTR2A/MYC | 4 |
| BP | GO:0015872 | dopamine transport | 3/77 | 51/18800 | 0.001194355 | 0.015443428 | 0.010808305 | GRM2/HTR2A/KCNA2 | 3 |
| BP | GO:0001768 | establishment of T cell polarity | 2/77 | 13/18800 | 0.0012543 | 0.015944299 | 0.011158846 | CYP26B1/TRAF3IP2 | 2 |
| BP | GO:0061418 | regulation of transcription from RNA polymerase II promoter in response to hypoxia | 2/77 | 13/18800 | 0.0012543 | 0.015944299 | 0.011158846 | HIF1A/TP53 | 2 |
| BP | GO:1903599 | positive regulation of autophagy of mitochondrion | 2/77 | 13/18800 | 0.0012543 | 0.015944299 | 0.011158846 | CAMKK2/HIF1A | 2 |
| BP | GO:0007215 | glutamate receptor signaling pathway | 3/77 | 52/18800 | 0.001263765 | 0.015944299 | 0.011158846 | GRIN1/GRM2/PTK2B | 3 |
| BP | GO:0043525 | positive regulation of neuron apoptotic process | 3/77 | 52/18800 | 0.001263765 | 0.015944299 | 0.011158846 | ABL1/TP53/CDK5 | 3 |
| BP | GO:0043620 | regulation of DNA-templated transcription in response to stress | 3/77 | 53/18800 | 0.001335667 | 0.016770036 | 0.01173675 | HIF1A/TP53/RELA | 3 |
| BP | GO:0001767 | establishment of lymphocyte polarity | 2/77 | 14/18800 | 0.00145947 | 0.017808276 | 0.012463377 | CYP26B1/TRAF3IP2 | 2 |
| BP | GO:0043650 | dicarboxylic acid biosynthetic process | 2/77 | 14/18800 | 0.00145947 | 0.017808276 | 0.012463377 | GLS/GOT1 | 2 |
| BP | GO:0048148 | behavioral response to cocaine | 2/77 | 14/18800 | 0.00145947 | 0.017808276 | 0.012463377 | HTR2A/CDK5 | 2 |
| BP | GO:0071236 | cellular response to antibiotic | 2/77 | 14/18800 | 0.00145947 | 0.017808276 | 0.012463377 | TP53/MEF2C | 2 |
| BP | GO:0071871 | response to epinephrine | 2/77 | 14/18800 | 0.00145947 | 0.017808276 | 0.012463377 | ABL1/RYR2 | 2 |
| BP | GO:1901857 | positive regulation of cellular respiration | 2/77 | 14/18800 | 0.00145947 | 0.017808276 | 0.012463377 | IL10RB/MYC | 2 |
| BP | GO:0051209 | release of sequestered calcium ion into cytosol | 4/77 | 121/18800 | 0.001538129 | 0.018680359 | 0.013073717 | ABL1/HTR2A/PTK2B/RYR2 | 4 |
| BP | GO:0048593 | camera-type eye morphogenesis | 4/77 | 122/18800 | 0.001585381 | 0.019075951 | 0.013350578 | HIF1A/RORB/THRB/RBP4 | 4 |
| BP | GO:0051283 | negative regulation of sequestering of calcium ion | 4/77 | 122/18800 | 0.001585381 | 0.019075951 | 0.013350578 | ABL1/HTR2A/PTK2B/RYR2 | 4 |
| BP | GO:0060042 | retina morphogenesis in camera-type eye | 3/77 | 57/18800 | 0.001648844 | 0.019268121 | 0.01348507 | RORB/THRB/RBP4 | 3 |
| BP | GO:0034329 | cell junction assembly | 7/77 | 420/18800 | 0.001656104 | 0.019268121 | 0.01348507 | ABL1/GABRA1/GABRA2/GABRB2/PTK2B/MEF2C/CDK5 | 7 |
| BP | GO:0006098 | pentose-phosphate shunt | 2/77 | 15/18800 | 0.001679541 | 0.019268121 | 0.01348507 | DERA/TP53 | 2 |
| BP | GO:0032488 | Cdc42 protein signal transduction | 2/77 | 15/18800 | 0.001679541 | 0.019268121 | 0.01348507 | ABL1/ABCA1 | 2 |
| BP | GO:0034380 | high-density lipoprotein particle assembly | 2/77 | 15/18800 | 0.001679541 | 0.019268121 | 0.01348507 | LCAT/ABCA1 | 2 |
| BP | GO:0045187 | regulation of circadian sleep/wake cycle, sleep | 2/77 | 15/18800 | 0.001679541 | 0.019268121 | 0.01348507 | ADRB1/KCNA2 | 2 |
| BP | GO:0055091 | phospholipid homeostasis | 2/77 | 15/18800 | 0.001679541 | 0.019268121 | 0.01348507 | FABP3/ABCA1 | 2 |
| BP | GO:0099509 | regulation of presynaptic cytosolic calcium ion concentration | 2/77 | 15/18800 | 0.001679541 | 0.019268121 | 0.01348507 | CACNB2/CDK5 | 2 |
| BP | GO:1903727 | positive regulation of phospholipid metabolic process | 2/77 | 15/18800 | 0.001679541 | 0.019268121 | 0.01348507 | HTR2A/FABP3 | 2 |
| BP | GO:2000052 | positive regulation of non-canonical Wnt signaling pathway | 2/77 | 15/18800 | 0.001679541 | 0.019268121 | 0.01348507 | ABL1/RSPO3 | 2 |
| BP | GO:0051282 | regulation of sequestering of calcium ion | 4/77 | 124/18800 | 0.001682902 | 0.019268121 | 0.01348507 | ABL1/HTR2A/PTK2B/RYR2 | 4 |
| BP | GO:0086065 | cell communication involved in cardiac conduction | 3/77 | 58/18800 | 0.001733691 | 0.019762562 | 0.013831112 | CACNB2/KCNJ3/RYR2 | 3 |
| BP | GO:0003231 | cardiac ventricle development | 4/77 | 126/18800 | 0.00178452 | 0.020253137 | 0.014174448 | HIF1A/LY6E/RYR2/MEF2C | 4 |
| BP | GO:0034764 | positive regulation of transmembrane transport | 5/77 | 213/18800 | 0.001816548 | 0.020490719 | 0.014340723 | ABL1/CACNB2/RYR2/SLC1A2/CDK5 | 5 |
| BP | GO:0051353 | positive regulation of oxidoreductase activity | 3/77 | 59/18800 | 0.001821222 | 0.020490719 | 0.014340723 | ABL1/HIF1A/PTK2B | 3 |
| BP | GO:0046474 | glycerophospholipid biosynthetic process | 5/77 | 214/18800 | 0.001854136 | 0.020771113 | 0.014536961 | HTR2A/LCAT/PITPNM1/FABP3/LPCAT3 | 5 |
| BP | GO:0051208 | sequestering of calcium ion | 4/77 | 128/18800 | 0.001890324 | 0.020851866 | 0.014593477 | ABL1/HTR2A/PTK2B/RYR2 | 4 |
| BP | GO:0060113 | inner ear receptor cell differentiation | 3/77 | 60/18800 | 0.001911466 | 0.020851866 | 0.014593477 | GABRB2/JAG2/GABRA5 | 3 |
| BP | GO:0006089 | lactate metabolic process | 2/77 | 16/18800 | 0.001914389 | 0.020851866 | 0.014593477 | HIF1A/TP53 | 2 |
| BP | GO:0034349 | glial cell apoptotic process | 2/77 | 16/18800 | 0.001914389 | 0.020851866 | 0.014593477 | TP53/CDK5 | 2 |
| BP | GO:0070242 | thymocyte apoptotic process | 2/77 | 16/18800 | 0.001914389 | 0.020851866 | 0.014593477 | HIF1A/TP53 | 2 |
| BP | GO:2000095 | regulation of Wnt signaling pathway, planar cell polarity pathway | 2/77 | 16/18800 | 0.001914389 | 0.020851866 | 0.014593477 | ABL1/RSPO3 | 2 |
| BP | GO:0048732 | gland development | 7/77 | 431/18800 | 0.001917505 | 0.020851866 | 0.014593477 | ABL1/CCKBR/HIF1A/IGF2R/LY6E/MYC/RELA | 7 |
| BP | GO:0001101 | response to acid chemical | 4/77 | 129/18800 | 0.001944822 | 0.021060805 | 0.014739706 | BCL2L2/GRIN1/SLC1A2/MYC | 4 |
| BP | GO:0006979 | response to oxidative stress | 7/77 | 433/18800 | 0.001968297 | 0.021226574 | 0.014855722 | ABL1/CAMKK2/HIF1A/MAPK9/PTK2B/TP53/RELA | 7 |
| BP | GO:0009132 | nucleoside diphosphate metabolic process | 4/77 | 130/18800 | 0.0020004 | 0.02148364 | 0.015035633 | CASK/HIF1A/HTR2A/MYC | 4 |
| BP | GO:0051651 | maintenance of location in cell | 5/77 | 219/18800 | 0.002050688 | 0.021911157 | 0.015334837 | ABL1/HTR2A/PTK2B/RYR2/CDK5 | 5 |
| BP | GO:0010595 | positive regulation of endothelial cell migration | 4/77 | 131/18800 | 0.002057069 | 0.021911157 | 0.015334837 | ABL1/HIF1A/PLK2/PTK2B | 4 |
| BP | GO:0001956 | positive regulation of neurotransmitter secretion | 2/77 | 17/18800 | 0.002163894 | 0.022769069 | 0.015935259 | CACNB2/CDK5 | 2 |
| BP | GO:0044539 | long-chain fatty acid import into cell | 2/77 | 17/18800 | 0.002163894 | 0.022769069 | 0.015935259 | SLC27A2/FABP3 | 2 |
| BP | GO:0050802 | circadian sleep/wake cycle, sleep | 2/77 | 17/18800 | 0.002163894 | 0.022769069 | 0.015935259 | ADRB1/KCNA2 | 2 |
| BP | GO:0030048 | actin filament-based movement | 4/77 | 133/18800 | 0.002173721 | 0.022780249 | 0.015943083 | CACNB2/KCNJ3/RYR2/SCN2B | 4 |
| BP | GO:0019229 | regulation of vasoconstriction | 3/77 | 64/18800 | 0.002300164 | 0.024008541 | 0.016802721 | ABL1/ADRA2A/HTR2A | 3 |
| BP | GO:0030534 | adult behavior | 4/77 | 136/18800 | 0.002357151 | 0.024363709 | 0.017051291 | GRM2/HTR2A/SLC1A2/CDK5 | 4 |
| BP | GO:0051235 | maintenance of location | 6/77 | 331/18800 | 0.002364475 | 0.024363709 | 0.017051291 | ABL1/HTR2A/PTK2B/RYR2/ABCA1/CDK5 | 6 |
| BP | GO:0042490 | mechanoreceptor differentiation | 3/77 | 65/18800 | 0.002404412 | 0.024363709 | 0.017051291 | GABRB2/JAG2/GABRA5 | 3 |
| BP | GO:0007586 | digestion | 4/77 | 137/18800 | 0.002420592 | 0.024363709 | 0.017051291 | ADRA2A/CCKBR/RBP4/LPCAT3 | 4 |
| BP | GO:0006740 | NADPH regeneration | 2/77 | 18/18800 | 0.002427934 | 0.024363709 | 0.017051291 | DERA/TP53 | 2 |
| BP | GO:0006977 | DNA damage response, signal transduction by p53 class mediator resulting in cell cycle arrest | 2/77 | 18/18800 | 0.002427934 | 0.024363709 | 0.017051291 | PLK2/TP53 | 2 |
| BP | GO:0008340 | determination of adult lifespan | 2/77 | 18/18800 | 0.002427934 | 0.024363709 | 0.017051291 | PRDM2/TP53 | 2 |
| BP | GO:0043649 | dicarboxylic acid catabolic process | 2/77 | 18/18800 | 0.002427934 | 0.024363709 | 0.017051291 | GAD2/GOT1 | 2 |
| BP | GO:0071071 | regulation of phospholipid biosynthetic process | 2/77 | 18/18800 | 0.002427934 | 0.024363709 | 0.017051291 | HTR2A/FABP3 | 2 |
| BP | GO:0140354 | lipid import into cell | 2/77 | 18/18800 | 0.002427934 | 0.024363709 | 0.017051291 | SLC27A2/FABP3 | 2 |
| BP | GO:0060048 | cardiac muscle contraction | 4/77 | 138/18800 | 0.002485199 | 0.024842429 | 0.017386329 | CACNB2/KCNJ3/RYR2/SCN2B | 4 |
| BP | GO:0010594 | regulation of endothelial cell migration | 5/77 | 230/18800 | 0.002536226 | 0.025255371 | 0.017675333 | ABL1/HIF1A/PLK2/PTK2B/MEF2C | 5 |
| BP | GO:0016358 | dendrite development | 5/77 | 231/18800 | 0.002584164 | 0.025634514 | 0.017940682 | ABL1/SLC12A5/KNDC1/MEF2C/CDK5 | 5 |
| BP | GO:0019216 | regulation of lipid metabolic process | 6/77 | 339/18800 | 0.00266244 | 0.026245922 | 0.018368584 | ADRA2A/EEF1A2/HTR2A/PTK2B/FABP3/LPCAT3 | 6 |
| BP | GO:0035296 | regulation of tube diameter | 4/77 | 141/18800 | 0.002686127 | 0.026245922 | 0.018368584 | ABL1/ADRA2A/ADRB1/HTR2A | 4 |
| BP | GO:0097746 | blood vessel diameter maintenance | 4/77 | 141/18800 | 0.002686127 | 0.026245922 | 0.018368584 | ABL1/ADRA2A/ADRB1/HTR2A | 4 |
| BP | GO:0042749 | regulation of circadian sleep/wake cycle | 2/77 | 19/18800 | 0.00270639 | 0.026245922 | 0.018368584 | ADRB1/KCNA2 | 2 |
| BP | GO:2000251 | positive regulation of actin cytoskeleton reorganization | 2/77 | 19/18800 | 0.00270639 | 0.026245922 | 0.018368584 | ABL1/CDK5 | 2 |
| BP | GO:2000269 | regulation of fibroblast apoptotic process | 2/77 | 19/18800 | 0.00270639 | 0.026245922 | 0.018368584 | STK17B/TP53 | 2 |
| BP | GO:0030183 | B cell differentiation | 4/77 | 142/18800 | 0.002755508 | 0.026426439 | 0.018494922 | ABL1/PTK2B/TP53/TRAF3IP2 | 4 |
| BP | GO:0032147 | activation of protein kinase activity | 4/77 | 142/18800 | 0.002755508 | 0.026426439 | 0.018494922 | ABL1/ADRA2A/CAMKK2/PTK2B | 4 |
| BP | GO:0035150 | regulation of tube size | 4/77 | 142/18800 | 0.002755508 | 0.026426439 | 0.018494922 | ABL1/ADRA2A/ADRB1/HTR2A | 4 |
| BP | GO:0035264 | multicellular organism growth | 4/77 | 143/18800 | 0.002826109 | 0.027003886 | 0.018899056 | ADRB1/SLC12A5/SLC1A2/TP53 | 4 |
| BP | GO:0043550 | regulation of lipid kinase activity | 3/77 | 69/18800 | 0.002850515 | 0.02703828 | 0.018923127 | CCKBR/EEF1A2/PTK2B | 3 |
| BP | GO:0050795 | regulation of behavior | 3/77 | 69/18800 | 0.002850515 | 0.02703828 | 0.018923127 | ADRB1/KCNA2/MEF2C | 3 |
| BP | GO:0051592 | response to calcium ion | 4/77 | 144/18800 | 0.002897941 | 0.027388172 | 0.019168005 | ADCY1/PTK2B/RYR2/MEF2C | 4 |
| BP | GO:0046395 | carboxylic acid catabolic process | 5/77 | 238/18800 | 0.002938378 | 0.027669725 | 0.019365053 | CYP26B1/GAD2/GLS/SLC27A2/GOT1 | 5 |
| BP | GO:0003143 | embryonic heart tube morphogenesis | 3/77 | 70/18800 | 0.00296945 | 0.02783846 | 0.019483145 | HIF1A/RYR2/MEF2C | 3 |
| BP | GO:0022410 | circadian sleep/wake cycle process | 2/77 | 20/18800 | 0.002999141 | 0.02783846 | 0.019483145 | ADRB1/KCNA2 | 2 |
| BP | GO:0043691 | reverse cholesterol transport | 2/77 | 20/18800 | 0.002999141 | 0.02783846 | 0.019483145 | LCAT/ABCA1 | 2 |
| BP | GO:2001014 | regulation of skeletal muscle cell differentiation | 2/77 | 20/18800 | 0.002999141 | 0.02783846 | 0.019483145 | CYP26B1/MEF2C | 2 |
| BP | GO:0006936 | muscle contraction | 6/77 | 349/18800 | 0.003073636 | 0.028390622 | 0.019869584 | ADRA2A/CACNB2/HTR2A/KCNJ3/RYR2/SCN2B | 6 |
| BP | GO:0050805 | negative regulation of synaptic transmission | 3/77 | 71/18800 | 0.003091399 | 0.028390622 | 0.019869584 | HTR2A/PLK2/PTK2B | 3 |
| BP | GO:2000379 | positive regulation of reactive oxygen species metabolic process | 3/77 | 71/18800 | 0.003091399 | 0.028390622 | 0.019869584 | GRIN1/PTK2B/TP53 | 3 |
| BP | GO:0034614 | cellular response to reactive oxygen species | 4/77 | 147/18800 | 0.003120925 | 0.028460649 | 0.019918592 | ABL1/CAMKK2/MAPK9/RELA | 4 |
| BP | GO:2000377 | regulation of reactive oxygen species metabolic process | 4/77 | 147/18800 | 0.003120925 | 0.028460649 | 0.019918592 | GRIN1/HIF1A/PTK2B/TP53 | 4 |
| BP | GO:0016054 | organic acid catabolic process | 5/77 | 242/18800 | 0.003155897 | 0.02867894 | 0.020071367 | CYP26B1/GAD2/GLS/SLC27A2/GOT1 | 5 |
| BP | GO:0010821 | regulation of mitochondrion organization | 4/77 | 148/18800 | 0.003197785 | 0.02885779 | 0.020196537 | BCL2L2/CAMKK2/HIF1A/TP53 | 4 |
| BP | GO:1904064 | positive regulation of cation transmembrane transport | 4/77 | 148/18800 | 0.003197785 | 0.02885779 | 0.020196537 | ABL1/CACNB2/RYR2/CDK5 | 4 |
| BP | GO:1901617 | organic hydroxy compound biosynthetic process | 5/77 | 243/18800 | 0.003212047 | 0.02888619 | 0.020216414 | PTK2B/SLC27A2/TYRP1/LPCAT3/GOT1 | 5 |
| BP | GO:0051259 | protein complex oligomerization | 5/77 | 244/18800 | 0.003268914 | 0.029028639 | 0.020316109 | GLS/GRIN1/KCNA2/SLC1A2/TP53 | 5 |
| BP | GO:0014850 | response to muscle activity | 2/77 | 21/18800 | 0.00330607 | 0.029028639 | 0.020316109 | HIF1A/RYR2 | 2 |
| BP | GO:0030502 | negative regulation of bone mineralization | 2/77 | 21/18800 | 0.00330607 | 0.029028639 | 0.020316109 | HIF1A/PTK2B | 2 |
| BP | GO:0038083 | peptidyl-tyrosine autophosphorylation | 2/77 | 21/18800 | 0.00330607 | 0.029028639 | 0.020316109 | ABL1/PTK2B | 2 |
| BP | GO:0071243 | cellular response to arsenic-containing substance | 2/77 | 21/18800 | 0.00330607 | 0.029028639 | 0.020316109 | GSTO2/MYC | 2 |
| BP | GO:0071498 | cellular response to fluid shear stress | 2/77 | 21/18800 | 0.00330607 | 0.029028639 | 0.020316109 | PTK2B/MEF2C | 2 |
| BP | GO:2000773 | negative regulation of cellular senescence | 2/77 | 21/18800 | 0.00330607 | 0.029028639 | 0.020316109 | ABL1/PLK2 | 2 |
| BP | GO:0033674 | positive regulation of kinase activity | 7/77 | 476/18800 | 0.003336343 | 0.029165861 | 0.020412146 | ABL1/ADRA2A/CAMKK2/EEF1A2/HTR2A/PTK2B/CDK5 | 7 |
| BP | GO:0030902 | hindbrain development | 4/77 | 150/18800 | 0.003355365 | 0.029165861 | 0.020412146 | ABL1/TP53/KNDC1/CDK5 | 4 |
| BP | GO:0043535 | regulation of blood vessel endothelial cell migration | 4/77 | 150/18800 | 0.003355365 | 0.029165861 | 0.020412146 | ABL1/HIF1A/PLK2/MEF2C | 4 |
| BP | GO:1903320 | regulation of protein modification by small protein conjugation or removal | 5/77 | 246/18800 | 0.003384825 | 0.029323865 | 0.020522727 | ABL1/HIF1A/MAPK9/RELA/CDK5 | 5 |
| BP | GO:0006887 | exocytosis | 6/77 | 357/18800 | 0.003435465 | 0.029663699 | 0.020760565 | ADCY1/ADRA2A/CACNB2/CASK/HTR2A/CDK5 | 6 |
| BP | GO:0046470 | phosphatidylcholine metabolic process | 3/77 | 74/18800 | 0.003475577 | 0.029910673 | 0.020933413 | LCAT/FABP3/LPCAT3 | 3 |
| BP | GO:0045834 | positive regulation of lipid metabolic process | 4/77 | 153/18800 | 0.003601539 | 0.029932825 | 0.020948917 | EEF1A2/HTR2A/PTK2B/FABP3 | 4 |
| BP | GO:0010822 | positive regulation of mitochondrion organization | 3/77 | 75/18800 | 0.003609825 | 0.029932825 | 0.020948917 | CAMKK2/HIF1A/TP53 | 3 |
| BP | GO:0043536 | positive regulation of blood vessel endothelial cell migration | 3/77 | 75/18800 | 0.003609825 | 0.029932825 | 0.020948917 | ABL1/HIF1A/PLK2 | 3 |
| BP | GO:0070227 | lymphocyte apoptotic process | 3/77 | 75/18800 | 0.003609825 | 0.029932825 | 0.020948917 | HIF1A/TP53/TRAF3IP2 | 3 |
| BP | GO:0010226 | response to lithium ion | 2/77 | 22/18800 | 0.003627059 | 0.029932825 | 0.020948917 | ADCY1/PTK2B | 2 |
| BP | GO:0035235 | ionotropic glutamate receptor signaling pathway | 2/77 | 22/18800 | 0.003627059 | 0.029932825 | 0.020948917 | GRIN1/PTK2B | 2 |
| BP | GO:0051000 | positive regulation of nitric-oxide synthase activity | 2/77 | 22/18800 | 0.003627059 | 0.029932825 | 0.020948917 | HIF1A/PTK2B | 2 |
| BP | GO:0051590 | positive regulation of neurotransmitter transport | 2/77 | 22/18800 | 0.003627059 | 0.029932825 | 0.020948917 | CACNB2/CDK5 | 2 |
| BP | GO:0086012 | membrane depolarization during cardiac muscle cell action potential | 2/77 | 22/18800 | 0.003627059 | 0.029932825 | 0.020948917 | CACNB2/SCN2B | 2 |
| BP | GO:1990806 | ligand-gated ion channel signaling pathway | 2/77 | 22/18800 | 0.003627059 | 0.029932825 | 0.020948917 | GRIN1/PTK2B | 2 |
| BP | GO:2000310 | regulation of NMDA receptor activity | 2/77 | 22/18800 | 0.003627059 | 0.029932825 | 0.020948917 | PTK2B/MEF2C | 2 |
| BP | GO:0006066 | alcohol metabolic process | 6/77 | 361/18800 | 0.003627872 | 0.029932825 | 0.020948917 | LCAT/PTK2B/RBP4/LPCAT3/GOT1/ABCA1 | 6 |
| BP | GO:1901653 | cellular response to peptide | 6/77 | 361/18800 | 0.003627872 | 0.029932825 | 0.020948917 | BCL2L2/TP53/MYC/RELA/GOT1/CDK5 | 6 |
| BP | GO:0016125 | sterol metabolic process | 4/77 | 154/18800 | 0.003686252 | 0.030222616 | 0.021151731 | CYP26B1/LCAT/LPCAT3/ABCA1 | 4 |
| BP | GO:0048592 | eye morphogenesis | 4/77 | 154/18800 | 0.003686252 | 0.030222616 | 0.021151731 | HIF1A/RORB/THRB/RBP4 | 4 |
| BP | GO:0007189 | adenylate cyclase-activating G protein-coupled receptor signaling pathway | 4/77 | 155/18800 | 0.003772311 | 0.030618071 | 0.021428496 | ADCY1/ADRA2A/ADRB1/ABCA1 | 4 |
| BP | GO:0009117 | nucleotide metabolic process | 7/77 | 487/18800 | 0.003781609 | 0.030618071 | 0.021428496 | ADCY1/CASK/DERA/HIF1A/HTR2A/IMPDH2/MYC | 7 |
| BP | GO:0010638 | positive regulation of organelle organization | 7/77 | 487/18800 | 0.003781609 | 0.030618071 | 0.021428496 | ABL1/CAMKK2/HIF1A/MAPK9/PTK2B/TP53/CDK5 | 7 |
| BP | GO:0090066 | regulation of anatomical structure size | 7/77 | 487/18800 | 0.003781609 | 0.030618071 | 0.021428496 | ABL1/ADRA2A/ADRB1/HTR2A/PTK2B/SLC12A5/CDK5 | 7 |
| BP | GO:0045017 | glycerolipid biosynthetic process | 5/77 | 253/18800 | 0.003813904 | 0.030783656 | 0.021544382 | HTR2A/LCAT/PITPNM1/FABP3/LPCAT3 | 5 |
| BP | GO:0009150 | purine ribonucleotide metabolic process | 6/77 | 366/18800 | 0.003879547 | 0.031185933 | 0.021825922 | ADCY1/CASK/HIF1A/HTR2A/IMPDH2/MYC | 6 |
| BP | GO:0045913 | positive regulation of carbohydrate metabolic process | 3/77 | 77/18800 | 0.003887742 | 0.031185933 | 0.021825922 | HIF1A/HTR2A/MYC | 3 |
| BP | GO:0042745 | circadian sleep/wake cycle | 2/77 | 23/18800 | 0.003961989 | 0.031683723 | 0.022174307 | ADRB1/KCNA2 | 2 |
| BP | GO:0071216 | cellular response to biotic stimulus | 5/77 | 256/18800 | 0.004009247 | 0.031904153 | 0.022328578 | ABL1/TP53/RELA/ABCA1/MEF2C | 5 |
| BP | GO:0009064 | glutamine family amino acid metabolic process | 3/77 | 78/18800 | 0.004031456 | 0.031904153 | 0.022328578 | GAD2/GLS/GOT1 | 3 |
| BP | GO:0045862 | positive regulation of proteolysis | 6/77 | 369/18800 | 0.004036655 | 0.031904153 | 0.022328578 | ADRA2A/GRIN1/MAPK9/PTK2B/MYC/LPCAT3 | 6 |
| BP | GO:0016241 | regulation of macroautophagy | 4/77 | 158/18800 | 0.004038656 | 0.031904153 | 0.022328578 | ATP6V1B2/HIF1A/TP53/CDK5 | 4 |
| BP | GO:0006091 | generation of precursor metabolites and energy | 7/77 | 494/18800 | 0.004087696 | 0.032115003 | 0.022476145 | DERA/HIF1A/HTR2A/IL10RB/TP53/TYRP1/MYC | 7 |
| BP | GO:2001233 | regulation of apoptotic signaling pathway | 6/77 | 370/18800 | 0.00409006 | 0.032115003 | 0.022476145 | BCL2L2/HIF1A/MAPK9/TP53/MYC/RELA | 6 |
| BP | GO:0006753 | nucleoside phosphate metabolic process | 7/77 | 495/18800 | 0.004132918 | 0.032320183 | 0.022619743 | ADCY1/CASK/DERA/HIF1A/HTR2A/IMPDH2/MYC | 7 |
| BP | GO:0008654 | phospholipid biosynthetic process | 5/77 | 258/18800 | 0.004143398 | 0.032320183 | 0.022619743 | HTR2A/LCAT/PITPNM1/FABP3/LPCAT3 | 5 |
| BP | GO:0006970 | response to osmotic stress | 3/77 | 79/18800 | 0.004178369 | 0.032320183 | 0.022619743 | PTK2B/SLC12A5/TP53 | 3 |
| BP | GO:0030301 | cholesterol transport | 3/77 | 79/18800 | 0.004178369 | 0.032320183 | 0.022619743 | LCAT/LPCAT3/ABCA1 | 3 |
| BP | GO:0048145 | regulation of fibroblast proliferation | 3/77 | 79/18800 | 0.004178369 | 0.032320183 | 0.022619743 | ABL1/TP53/MYC | 3 |
| BP | GO:0018108 | peptidyl-tyrosine phosphorylation | 6/77 | 373/18800 | 0.004253426 | 0.032803125 | 0.022957737 | ABL1/ADRA2A/CAMKK2/HTR2A/PTK2B/TP53 | 6 |
| BP | GO:0035994 | response to muscle stretch | 2/77 | 24/18800 | 0.004310745 | 0.032951843 | 0.023061819 | RYR2/RELA | 2 |
| BP | GO:0036003 | positive regulation of transcription from RNA polymerase II promoter in response to stress | 2/77 | 24/18800 | 0.004310745 | 0.032951843 | 0.023061819 | HIF1A/TP53 | 2 |
| BP | GO:0051156 | glucose 6-phosphate metabolic process | 2/77 | 24/18800 | 0.004310745 | 0.032951843 | 0.023061819 | DERA/TP53 | 2 |
| BP | GO:0051279 | regulation of release of sequestered calcium ion into cytosol | 3/77 | 80/18800 | 0.004328502 | 0.032990548 | 0.023088908 | ABL1/PTK2B/RYR2 | 3 |
| BP | GO:0050730 | regulation of peptidyl-tyrosine phosphorylation | 5/77 | 261/18800 | 0.004350609 | 0.033062085 | 0.023138974 | ABL1/ADRA2A/HTR2A/PTK2B/TP53 | 5 |
| BP | GO:0018212 | peptidyl-tyrosine modification | 6/77 | 376/18800 | 0.004421582 | 0.033503475 | 0.023447887 | ABL1/ADRA2A/CAMKK2/HTR2A/PTK2B/TP53 | 6 |
| BP | GO:0006096 | glycolytic process | 3/77 | 81/18800 | 0.004481876 | 0.033553996 | 0.023483244 | HIF1A/HTR2A/MYC | 3 |
| BP | GO:0021675 | nerve development | 3/77 | 81/18800 | 0.004481876 | 0.033553996 | 0.023483244 | GABRB2/KCNA2/GABRA5 | 3 |
| BP | GO:0030500 | regulation of bone mineralization | 3/77 | 81/18800 | 0.004481876 | 0.033553996 | 0.023483244 | HIF1A/PTK2B/MEF2C | 3 |
| BP | GO:0048144 | fibroblast proliferation | 3/77 | 81/18800 | 0.004481876 | 0.033553996 | 0.023483244 | ABL1/TP53/MYC | 3 |
| BP | GO:0003018 | vascular process in circulatory system | 5/77 | 263/18800 | 0.004492801 | 0.033553996 | 0.023483244 | ABL1/ADRA2A/ADRB1/HTR2A/SLC1A2 | 5 |
| BP | GO:0034767 | positive regulation of ion transmembrane transport | 4/77 | 163/18800 | 0.004510397 | 0.033588885 | 0.023507662 | ABL1/CACNB2/RYR2/CDK5 | 4 |
| BP | GO:0006757 | ATP generation from ADP | 3/77 | 82/18800 | 0.004638511 | 0.034309815 | 0.024012216 | HIF1A/HTR2A/MYC | 3 |
| BP | GO:0042310 | vasoconstriction | 3/77 | 82/18800 | 0.004638511 | 0.034309815 | 0.024012216 | ABL1/ADRA2A/HTR2A | 3 |
| BP | GO:0021697 | cerebellar cortex formation | 2/77 | 25/18800 | 0.004673211 | 0.034309815 | 0.024012216 | KNDC1/CDK5 | 2 |
| BP | GO:0060219 | camera-type eye photoreceptor cell differentiation | 2/77 | 25/18800 | 0.004673211 | 0.034309815 | 0.024012216 | RORB/THRB | 2 |
| BP | GO:2000463 | positive regulation of excitatory postsynaptic potential | 2/77 | 25/18800 | 0.004673211 | 0.034309815 | 0.024012216 | GRIN1/PTK2B | 2 |
| BP | GO:0090596 | sensory organ morphogenesis | 5/77 | 266/18800 | 0.00471227 | 0.034499123 | 0.024144705 | CYP26B1/HIF1A/RORB/THRB/RBP4 | 5 |
| BP | GO:0009259 | ribonucleotide metabolic process | 6/77 | 384/18800 | 0.00489406 | 0.035729389 | 0.025005724 | ADCY1/CASK/HIF1A/HTR2A/IMPDH2/MYC | 6 |
| BP | GO:0035050 | embryonic heart tube development | 3/77 | 84/18800 | 0.004961644 | 0.035953574 | 0.025162624 | HIF1A/RYR2/MEF2C | 3 |
| BP | GO:0051899 | membrane depolarization | 3/77 | 84/18800 | 0.004961644 | 0.035953574 | 0.025162624 | ABL1/CACNB2/SCN2B | 3 |
| BP | GO:0002026 | regulation of the force of heart contraction | 2/77 | 26/18800 | 0.005049271 | 0.035953574 | 0.025162624 | ADRB1/RYR2 | 2 |
| BP | GO:0030318 | melanocyte differentiation | 2/77 | 26/18800 | 0.005049271 | 0.035953574 | 0.025162624 | TYRP1/MEF2C | 2 |
| BP | GO:0046037 | GMP metabolic process | 2/77 | 26/18800 | 0.005049271 | 0.035953574 | 0.025162624 | CASK/IMPDH2 | 2 |
| BP | GO:0050951 | sensory perception of temperature stimulus | 2/77 | 26/18800 | 0.005049271 | 0.035953574 | 0.025162624 | ADRA2A/HTR2A | 2 |
| BP | GO:0060384 | innervation | 2/77 | 26/18800 | 0.005049271 | 0.035953574 | 0.025162624 | GABRB2/GABRA5 | 2 |
| BP | GO:1904385 | cellular response to angiotensin | 2/77 | 26/18800 | 0.005049271 | 0.035953574 | 0.025162624 | MYC/RELA | 2 |
| BP | GO:2000050 | regulation of non-canonical Wnt signaling pathway | 2/77 | 26/18800 | 0.005049271 | 0.035953574 | 0.025162624 | ABL1/RSPO3 | 2 |
| BP | GO:0001701 | in utero embryonic development | 6/77 | 387/18800 | 0.005080502 | 0.036077117 | 0.025249087 | HIF1A/LY6E/RSPO3/TP53/MYC/JAG2 | 6 |
| BP | GO:2000106 | regulation of leukocyte apoptotic process | 3/77 | 85/18800 | 0.005128181 | 0.036316463 | 0.025416597 | HIF1A/TP53/MEF2C | 3 |
| BP | GO:0046034 | ATP metabolic process | 5/77 | 273/18800 | 0.005253923 | 0.037105834 | 0.025969049 | ATP6V1B2/HIF1A/HTR2A/TP53/MYC | 5 |
| BP | GO:1900542 | regulation of purine nucleotide metabolic process | 3/77 | 86/18800 | 0.005298056 | 0.037316119 | 0.02611622 | HIF1A/HTR2A/MYC | 3 |
| BP | GO:2001242 | regulation of intrinsic apoptotic signaling pathway | 4/77 | 171/18800 | 0.005340262 | 0.037511732 | 0.026253123 | BCL2L2/HIF1A/TP53/MYC | 4 |
| BP | GO:0010460 | positive regulation of heart rate | 2/77 | 27/18800 | 0.005438811 | 0.0378967 | 0.026522548 | ADRB1/RYR2 | 2 |
| BP | GO:0071880 | adenylate cyclase-activating adrenergic receptor signaling pathway | 2/77 | 27/18800 | 0.005438811 | 0.0378967 | 0.026522548 | ADRA2A/ADRB1 | 2 |
| BP | GO:2000108 | positive regulation of leukocyte apoptotic process | 2/77 | 27/18800 | 0.005438811 | 0.0378967 | 0.026522548 | TP53/MEF2C | 2 |
| BP | GO:1901216 | positive regulation of neuron death | 3/77 | 87/18800 | 0.005471288 | 0.038021066 | 0.026609587 | ABL1/TP53/CDK5 | 3 |
| BP | GO:0006163 | purine nucleotide metabolic process | 6/77 | 394/18800 | 0.005535836 | 0.038264994 | 0.026780304 | ADCY1/CASK/HIF1A/HTR2A/IMPDH2/MYC | 6 |
| BP | GO:0019693 | ribose phosphate metabolic process | 6/77 | 394/18800 | 0.005535836 | 0.038264994 | 0.026780304 | ADCY1/CASK/HIF1A/HTR2A/IMPDH2/MYC | 6 |
| BP | GO:0055088 | lipid homeostasis | 4/77 | 173/18800 | 0.005562645 | 0.038317537 | 0.026817077 | LCAT/FABP3/GOT1/ABCA1 | 4 |
| BP | GO:0042176 | regulation of protein catabolic process | 6/77 | 395/18800 | 0.00560325 | 0.038317537 | 0.026817077 | ADRA2A/EEF1A2/MAPK9/PLK2/PTK2B/RELA | 6 |
| BP | GO:0000422 | autophagy of mitochondrion | 3/77 | 88/18800 | 0.005647896 | 0.038317537 | 0.026817077 | CAMKK2/HIF1A/TP53 | 3 |
| BP | GO:0006140 | regulation of nucleotide metabolic process | 3/77 | 88/18800 | 0.005647896 | 0.038317537 | 0.026817077 | HIF1A/HTR2A/MYC | 3 |
| BP | GO:0051262 | protein tetramerization | 3/77 | 88/18800 | 0.005647896 | 0.038317537 | 0.026817077 | GLS/GRIN1/TP53 | 3 |
| BP | GO:0061726 | mitochondrion disassembly | 3/77 | 88/18800 | 0.005647896 | 0.038317537 | 0.026817077 | CAMKK2/HIF1A/TP53 | 3 |
| BP | GO:0045860 | positive regulation of protein kinase activity | 6/77 | 396/18800 | 0.005671264 | 0.038317537 | 0.026817077 | ABL1/ADRA2A/CAMKK2/HTR2A/PTK2B/CDK5 | 6 |
| BP | GO:0046486 | glycerolipid metabolic process | 6/77 | 396/18800 | 0.005671264 | 0.038317537 | 0.026817077 | HTR2A/LCAT/PITPNM1/FABP3/LPCAT3/MGLL | 6 |
| BP | GO:0010634 | positive regulation of epithelial cell migration | 4/77 | 174/18800 | 0.005676126 | 0.038317537 | 0.026817077 | ABL1/HIF1A/PLK2/PTK2B | 4 |
| BP | GO:0043542 | endothelial cell migration | 5/77 | 279/18800 | 0.005752202 | 0.038730501 | 0.027106096 | ABL1/HIF1A/PLK2/PTK2B/MEF2C | 5 |
| BP | GO:0046031 | ADP metabolic process | 3/77 | 90/18800 | 0.006011304 | 0.040054078 | 0.02803242 | HIF1A/HTR2A/MYC | 3 |
| BP | GO:0048872 | homeostasis of number of cells | 5/77 | 282/18800 | 0.006013478 | 0.040054078 | 0.02803242 | ABL1/HIF1A/TRAF3IP2/LPCAT3/MEF2C | 5 |
| BP | GO:0043254 | regulation of protein-containing complex assembly | 6/77 | 401/18800 | 0.00602046 | 0.040054078 | 0.02803242 | ABL1/LCAT/MAPK9/PTK2B/TP53/ABCA1 | 6 |
| BP | GO:0001659 | temperature homeostasis | 4/77 | 177/18800 | 0.006025835 | 0.040054078 | 0.02803242 | ADRB1/HTR2A/PCTP/PTH2R | 4 |
| BP | GO:0050954 | sensory perception of mechanical stimulus | 4/77 | 177/18800 | 0.006025835 | 0.040054078 | 0.02803242 | GABRB2/HTR2A/THRB/GABRA5 | 4 |
| BP | GO:0006941 | striated muscle contraction | 4/77 | 178/18800 | 0.006145523 | 0.040745447 | 0.028516285 | CACNB2/KCNJ3/RYR2/SCN2B | 4 |
| BP | GO:0060996 | dendritic spine development | 3/77 | 91/18800 | 0.00619814 | 0.040885701 | 0.028614444 | SLC12A5/MEF2C/CDK5 | 3 |
| BP | GO:0070509 | calcium ion import | 3/77 | 91/18800 | 0.00619814 | 0.040885701 | 0.028614444 | CACNB2/CASK/CDK5 | 3 |
| BP | GO:0031571 | mitotic G1 DNA damage checkpoint signaling | 2/77 | 29/18800 | 0.006257876 | 0.041175242 | 0.028817083 | PLK2/TP53 | 2 |
| BP | GO:0060537 | muscle tissue development | 6/77 | 405/18800 | 0.006310951 | 0.041419602 | 0.028988101 | CYP26B1/LY6E/RYR2/RBP4/MEF2C/CDK5 | 6 |
| BP | GO:0001666 | response to hypoxia | 5/77 | 286/18800 | 0.006374737 | 0.041725269 | 0.029202027 | HIF1A/PTK2B/RYR2/TP53/MYC | 5 |
| BP | GO:2001257 | regulation of cation channel activity | 4/77 | 180/18800 | 0.006389633 | 0.041725269 | 0.029202027 | CACNB2/PTK2B/MEF2C/CDK5 | 4 |
| BP | GO:0044819 | mitotic G1/S transition checkpoint signaling | 2/77 | 30/18800 | 0.006687176 | 0.043019731 | 0.030107975 | PLK2/TP53 | 2 |
| BP | GO:0048147 | negative regulation of fibroblast proliferation | 2/77 | 30/18800 | 0.006687176 | 0.043019731 | 0.030107975 | TP53/MYC | 2 |
| BP | GO:0060218 | hematopoietic stem cell differentiation | 2/77 | 30/18800 | 0.006687176 | 0.043019731 | 0.030107975 | ABL1/TP53 | 2 |
| BP | GO:0060292 | long-term synaptic depression | 2/77 | 30/18800 | 0.006687176 | 0.043019731 | 0.030107975 | PLK2/PTK2B | 2 |
| BP | GO:0070168 | negative regulation of biomineral tissue development | 2/77 | 30/18800 | 0.006687176 | 0.043019731 | 0.030107975 | HIF1A/PTK2B | 2 |
| BP | GO:1990776 | response to angiotensin | 2/77 | 30/18800 | 0.006687176 | 0.043019731 | 0.030107975 | MYC/RELA | 2 |
| BP | GO:0010632 | regulation of epithelial cell migration | 5/77 | 290/18800 | 0.006751022 | 0.043291353 | 0.030298073 | ABL1/HIF1A/PLK2/PTK2B/MEF2C | 5 |
| BP | GO:0015918 | sterol transport | 3/77 | 94/18800 | 0.006779369 | 0.043291353 | 0.030298073 | LCAT/LPCAT3/ABCA1 | 3 |
| BP | GO:1901606 | alpha-amino acid catabolic process | 3/77 | 94/18800 | 0.006779369 | 0.043291353 | 0.030298073 | GAD2/GLS/GOT1 | 3 |
| BP | GO:0043648 | dicarboxylic acid metabolic process | 3/77 | 95/18800 | 0.006980072 | 0.044463742 | 0.031118587 | GAD2/GLS/GOT1 | 3 |
| BP | GO:0110150 | negative regulation of biomineralization | 2/77 | 31/18800 | 0.007129505 | 0.045293066 | 0.031699001 | HIF1A/PTK2B | 2 |
| BP | GO:0002040 | sprouting angiogenesis | 4/77 | 186/18800 | 0.00716046 | 0.045293066 | 0.031699001 | ABL1/PLK2/PTK2B/RSPO3 | 4 |
| BP | GO:0072521 | purine-containing compound metabolic process | 6/77 | 416/18800 | 0.007162543 | 0.045293066 | 0.031699001 | ADCY1/CASK/HIF1A/HTR2A/IMPDH2/MYC | 6 |
| BP | GO:1903321 | negative regulation of protein modification by small protein conjugation or removal | 3/77 | 96/18800 | 0.00718428 | 0.045320249 | 0.031718025 | ABL1/RELA/CDK5 | 3 |
| BP | GO:0097193 | intrinsic apoptotic signaling pathway | 5/77 | 295/18800 | 0.007242992 | 0.045579994 | 0.031899811 | ABL1/BCL2L2/HIF1A/TP53/MYC | 5 |
| BP | GO:0043123 | positive regulation of I-kappaB kinase/NF-kappaB signaling | 4/77 | 188/18800 | 0.007430465 | 0.046534409 | 0.032567772 | ABL1/PLK2/TRAF3IP2/RELA | 4 |
| BP | GO:0098739 | import across plasma membrane | 4/77 | 188/18800 | 0.007430465 | 0.046534409 | 0.032567772 | KCNJ3/KCNJ6/SLC12A5/SLC1A2 | 4 |
| BP | GO:0010743 | regulation of macrophage derived foam cell differentiation | 2/77 | 32/18800 | 0.007584752 | 0.047159738 | 0.033005418 | MAPK9/ABCA1 | 2 |
| BP | GO:0032770 | positive regulation of monooxygenase activity | 2/77 | 32/18800 | 0.007584752 | 0.047159738 | 0.033005418 | HIF1A/PTK2B | 2 |
| BP | GO:1902253 | regulation of intrinsic apoptotic signaling pathway by p53 class mediator | 2/77 | 32/18800 | 0.007584752 | 0.047159738 | 0.033005418 | TP53/MYC | 2 |
| BP | GO:0042632 | cholesterol homeostasis | 3/77 | 98/18800 | 0.007603269 | 0.04716204 | 0.033007029 | LCAT/FABP3/ABCA1 | 3 |
| BP | GO:0036293 | response to decreased oxygen levels | 5/77 | 299/18800 | 0.007654242 | 0.047365176 | 0.033149196 | HIF1A/PTK2B/RYR2/TP53/MYC | 5 |
| BP | GO:0006165 | nucleoside diphosphate phosphorylation | 3/77 | 99/18800 | 0.007818078 | 0.048149726 | 0.033698276 | HIF1A/HTR2A/MYC | 3 |
| BP | GO:0055092 | sterol homeostasis | 3/77 | 99/18800 | 0.007818078 | 0.048149726 | 0.033698276 | LCAT/FABP3/ABCA1 | 3 |
| BP | GO:0046939 | nucleotide phosphorylation | 3/77 | 100/18800 | 0.008036449 | 0.048786115 | 0.034143661 | HIF1A/HTR2A/MYC | 3 |
| BP | GO:0001662 | behavioral fear response | 2/77 | 33/18800 | 0.008052806 | 0.048786115 | 0.034143661 | MEF2C/GABRA5 | 2 |
| BP | GO:0045907 | positive regulation of vasoconstriction | 2/77 | 33/18800 | 0.008052806 | 0.048786115 | 0.034143661 | ABL1/HTR2A | 2 |
| BP | GO:0046685 | response to arsenic-containing substance | 2/77 | 33/18800 | 0.008052806 | 0.048786115 | 0.034143661 | GSTO2/MYC | 2 |
| BP | GO:0070050 | neuron cellular homeostasis | 2/77 | 33/18800 | 0.008052806 | 0.048786115 | 0.034143661 | CACNB2/CDK5 | 2 |
| BP | GO:0071875 | adrenergic receptor signaling pathway | 2/77 | 33/18800 | 0.008052806 | 0.048786115 | 0.034143661 | ADRA2A/ADRB1 | 2 |
| BP | GO:1903715 | regulation of aerobic respiration | 2/77 | 33/18800 | 0.008052806 | 0.048786115 | 0.034143661 | HIF1A/MYC | 2 |
| BP | GO:0019932 | second-messenger-mediated signaling | 5/77 | 303/18800 | 0.008081536 | 0.048846309 | 0.034185789 | ADCY1/CAMKK2/GRIN1/PTK2B/RYR2 | 5 |
| BP | GO:0008630 | intrinsic apoptotic signaling pathway in response to DNA damage | 3/77 | 101/18800 | 0.008258395 | 0.049684184 | 0.034772188 | ABL1/BCL2L2/TP53 | 3 |
| BP | GO:0070167 | regulation of biomineral tissue development | 3/77 | 101/18800 | 0.008258395 | 0.049684184 | 0.034772188 | HIF1A/PTK2B/MEF2C | 3 |
| BP | GO:0031334 | positive regulation of protein-containing complex assembly | 4/77 | 194/18800 | 0.008280617 | 0.049702824 | 0.034785233 | MAPK9/PTK2B/TP53/ABCA1 | 4 |
| BP | GO:0015914 | phospholipid transport | 3/77 | 102/18800 | 0.008483928 | 0.050520997 | 0.035357843 | PCTP/PITPNM1/ABCA1 | 3 |
| BP | GO:0002209 | behavioral defense response | 2/77 | 34/18800 | 0.008533558 | 0.050520997 | 0.035357843 | MEF2C/GABRA5 | 2 |
| BP | GO:0021696 | cerebellar cortex morphogenesis | 2/77 | 34/18800 | 0.008533558 | 0.050520997 | 0.035357843 | KNDC1/CDK5 | 2 |
| BP | GO:0048536 | spleen development | 2/77 | 34/18800 | 0.008533558 | 0.050520997 | 0.035357843 | ABL1/TRAF3IP2 | 2 |
| BP | GO:0070232 | regulation of T cell apoptotic process | 2/77 | 34/18800 | 0.008533558 | 0.050520997 | 0.035357843 | HIF1A/TP53 | 2 |
| BP | GO:1902692 | regulation of neuroblast proliferation | 2/77 | 34/18800 | 0.008533558 | 0.050520997 | 0.035357843 | HIF1A/TP53 | 2 |
| BP | GO:0110149 | regulation of biomineralization | 3/77 | 103/18800 | 0.008713062 | 0.051466471 | 0.036019547 | HIF1A/PTK2B/MEF2C | 3 |
| BP | GO:0006650 | glycerophospholipid metabolic process | 5/77 | 309/18800 | 0.008753235 | 0.051586523 | 0.036103567 | HTR2A/LCAT/PITPNM1/FABP3/LPCAT3 | 5 |
| BP | GO:0007200 | phospholipase C-activating G protein-coupled receptor signaling pathway | 3/77 | 104/18800 | 0.008945808 | 0.052602159 | 0.036814374 | ADRA2A/CCKBR/HTR2A | 3 |
| BP | GO:0042462 | eye photoreceptor cell development | 2/77 | 35/18800 | 0.009026899 | 0.052721146 | 0.036897649 | RORB/THRB | 2 |
| BP | GO:0086005 | ventricular cardiac muscle cell action potential | 2/77 | 35/18800 | 0.009026899 | 0.052721146 | 0.036897649 | KCNJ3/RYR2 | 2 |
| BP | GO:0090050 | positive regulation of cell migration involved in sprouting angiogenesis | 2/77 | 35/18800 | 0.009026899 | 0.052721146 | 0.036897649 | ABL1/PLK2 | 2 |
| BP | GO:0072001 | renal system development | 5/77 | 312/18800 | 0.0091032 | 0.053047574 | 0.037126104 | CYP26B1/TRAF3IP2/MYC/RBP4/MEF2C | 5 |
| BP | GO:0046425 | regulation of receptor signaling pathway via JAK-STAT | 3/77 | 105/18800 | 0.009182178 | 0.053150292 | 0.037197993 | IL10RB/PTK2B/CDK5 | 3 |
| BP | GO:0071868 | cellular response to monoamine stimulus | 3/77 | 105/18800 | 0.009182178 | 0.053150292 | 0.037197993 | ABL1/HTR2A/RYR2 | 3 |
| BP | GO:0071870 | cellular response to catecholamine stimulus | 3/77 | 105/18800 | 0.009182178 | 0.053150292 | 0.037197993 | ABL1/HTR2A/RYR2 | 3 |
| BP | GO:0009612 | response to mechanical stimulus | 4/77 | 201/18800 | 0.009350397 | 0.054003736 | 0.037795288 | HTR2A/PTK2B/RYR2/RELA | 4 |
| BP | GO:0006090 | pyruvate metabolic process | 3/77 | 106/18800 | 0.009422185 | 0.054057964 | 0.03783324 | HIF1A/HTR2A/MYC | 3 |
| BP | GO:0032414 | positive regulation of ion transmembrane transporter activity | 3/77 | 106/18800 | 0.009422185 | 0.054057964 | 0.03783324 | CACNB2/RYR2/CDK5 | 3 |
| BP | GO:0051341 | regulation of oxidoreductase activity | 3/77 | 106/18800 | 0.009422185 | 0.054057964 | 0.03783324 | ABL1/HIF1A/PTK2B | 3 |
| BP | GO:0019722 | calcium-mediated signaling | 4/77 | 202/18800 | 0.009510221 | 0.054213431 | 0.037942046 | CAMKK2/GRIN1/PTK2B/RYR2 | 4 |
| BP | GO:0050931 | pigment cell differentiation | 2/77 | 36/18800 | 0.00953272 | 0.054213431 | 0.037942046 | TYRP1/MEF2C | 2 |
| BP | GO:0071312 | cellular response to alkaloid | 2/77 | 36/18800 | 0.00953272 | 0.054213431 | 0.037942046 | RYR2/SLC1A2 | 2 |
| BP | GO:0086010 | membrane depolarization during action potential | 2/77 | 36/18800 | 0.00953272 | 0.054213431 | 0.037942046 | CACNB2/SCN2B | 2 |
| BP | GO:0015849 | organic acid transport | 5/77 | 318/18800 | 0.009831994 | 0.055793347 | 0.039047773 | GRM2/SLC1A2/SLC27A2/MYC/FABP3 | 5 |
| BP | GO:0007173 | epidermal growth factor receptor signaling pathway | 3/77 | 108/18800 | 0.00991315 | 0.055887801 | 0.039113878 | ABL1/ADRA2A/PTK2B | 3 |
| BP | GO:0071867 | response to monoamine | 3/77 | 108/18800 | 0.00991315 | 0.055887801 | 0.039113878 | ABL1/HTR2A/RYR2 | 3 |
| BP | GO:0071869 | response to catecholamine | 3/77 | 108/18800 | 0.00991315 | 0.055887801 | 0.039113878 | ABL1/HTR2A/RYR2 | 3 |
| BP | GO:0060249 | anatomical structure homeostasis | 5/77 | 319/18800 | 0.009957255 | 0.056014946 | 0.039202862 | CACNB2/HIF1A/PTK2B/RBP4/CDK5 | 5 |
| BP | GO:0006739 | NADP metabolic process | 2/77 | 37/18800 | 0.010050913 | 0.056177038 | 0.039316304 | DERA/TP53 | 2 |
| BP | GO:0090218 | positive regulation of lipid kinase activity | 2/77 | 37/18800 | 0.010050913 | 0.056177038 | 0.039316304 | EEF1A2/PTK2B | 2 |
| BP | GO:1903725 | regulation of phospholipid metabolic process | 2/77 | 37/18800 | 0.010050913 | 0.056177038 | 0.039316304 | HTR2A/FABP3 | 2 |
| BP | GO:0003012 | muscle system process | 6/77 | 449/18800 | 0.010217274 | 0.056862303 | 0.039795897 | ADRA2A/CACNB2/HTR2A/KCNJ3/RYR2/SCN2B | 6 |
| BP | GO:0048568 | embryonic organ development | 6/77 | 449/18800 | 0.010217274 | 0.056862303 | 0.039795897 | HIF1A/RSPO3/RYR2/TP53/RBP4/MEF2C | 6 |
| BP | GO:0043266 | regulation of potassium ion transport | 3/77 | 110/18800 | 0.01041879 | 0.057859902 | 0.040494081 | ADRA2A/HTR2A/PTK2B | 3 |
| BP | GO:0008202 | steroid metabolic process | 5/77 | 323/18800 | 0.010469306 | 0.058016476 | 0.040603662 | CYP26B1/LCAT/SLC27A2/LPCAT3/ABCA1 | 5 |
| BP | GO:0045637 | regulation of myeloid cell differentiation | 4/77 | 208/18800 | 0.010506653 | 0.058099555 | 0.040661806 | HIF1A/PTK2B/MYC/MEF2C | 4 |
| BP | GO:0010742 | macrophage derived foam cell differentiation | 2/77 | 38/18800 | 0.010581372 | 0.058121613 | 0.040677243 | MAPK9/ABCA1 | 2 |
| BP | GO:0098926 | postsynaptic signal transduction | 2/77 | 38/18800 | 0.010581372 | 0.058121613 | 0.040677243 | LY6E/RELA | 2 |
| BP | GO:1901385 | regulation of voltage-gated calcium channel activity | 2/77 | 38/18800 | 0.010581372 | 0.058121613 | 0.040677243 | CACNB2/CDK5 | 2 |
| BP | GO:0070482 | response to oxygen levels | 5/77 | 324/18800 | 0.010600094 | 0.058121613 | 0.040677243 | HIF1A/PTK2B/RYR2/TP53/MYC | 5 |
| BP | GO:0031532 | actin cytoskeleton reorganization | 3/77 | 111/18800 | 0.010677138 | 0.058175852 | 0.040715203 | ABL1/PTK2B/CDK5 | 3 |
| BP | GO:0048640 | negative regulation of developmental growth | 3/77 | 111/18800 | 0.010677138 | 0.058175852 | 0.040715203 | ADRB1/RBP4/CDK5 | 3 |
| BP | GO:0071347 | cellular response to interleukin-1 | 3/77 | 111/18800 | 0.010677138 | 0.058175852 | 0.040715203 | HIF1A/MYC/RELA | 3 |
| BP | GO:0009063 | cellular amino acid catabolic process | 3/77 | 112/18800 | 0.010939185 | 0.059478957 | 0.0416272 | GAD2/GLS/GOT1 | 3 |
| BP | GO:0016042 | lipid catabolic process | 5/77 | 327/18800 | 0.010999187 | 0.059544249 | 0.041672895 | ADRA2A/CYP26B1/NCEH1/SLC27A2/MGLL | 5 |
| BP | GO:0048863 | stem cell differentiation | 4/77 | 211/18800 | 0.011029295 | 0.059544249 | 0.041672895 | ABL1/HIF1A/TP53/MEF2C | 4 |
| BP | GO:0003016 | respiratory system process | 2/77 | 39/18800 | 0.011123989 | 0.059544249 | 0.041672895 | GLS/JAG2 | 2 |
| BP | GO:0043267 | negative regulation of potassium ion transport | 2/77 | 39/18800 | 0.011123989 | 0.059544249 | 0.041672895 | HTR2A/PTK2B | 2 |
| BP | GO:0045823 | positive regulation of heart contraction | 2/77 | 39/18800 | 0.011123989 | 0.059544249 | 0.041672895 | ADRB1/RYR2 | 2 |
| BP | GO:0090077 | foam cell differentiation | 2/77 | 39/18800 | 0.011123989 | 0.059544249 | 0.041672895 | MAPK9/ABCA1 | 2 |
| BP | GO:0098815 | modulation of excitatory postsynaptic potential | 2/77 | 39/18800 | 0.011123989 | 0.059544249 | 0.041672895 | GRIN1/PTK2B | 2 |
| BP | GO:0060562 | epithelial tube morphogenesis | 5/77 | 328/18800 | 0.011134477 | 0.059544249 | 0.041672895 | ABL1/HIF1A/RYR2/MYC/MEF2C | 5 |
| BP | GO:0048638 | regulation of developmental growth | 5/77 | 329/18800 | 0.011270902 | 0.060150052 | 0.042096875 | ABL1/ADRB1/RBP4/MEF2C/CDK5 | 5 |
| BP | GO:0043010 | camera-type eye development | 5/77 | 330/18800 | 0.011408469 | 0.060759448 | 0.04252337 | HIF1A/IMPDH2/RORB/THRB/RBP4 | 5 |
| BP | GO:0043200 | response to amino acid | 3/77 | 114/18800 | 0.011474412 | 0.060985677 | 0.042681699 | BCL2L2/GRIN1/SLC1A2 | 3 |
| BP | GO:0017158 | regulation of calcium ion-dependent exocytosis | 2/77 | 40/18800 | 0.01167866 | 0.061818406 | 0.043264497 | ADRA2A/CDK5 | 2 |
| BP | GO:1903524 | positive regulation of blood circulation | 2/77 | 40/18800 | 0.01167866 | 0.061818406 | 0.043264497 | ADRB1/RYR2 | 2 |
| BP | GO:0030258 | lipid modification | 4/77 | 216/18800 | 0.011937211 | 0.063058561 | 0.044132437 | LCAT/SLC27A2/FABP3/LPCAT3 | 4 |
| BP | GO:0007517 | muscle organ development | 5/77 | 334/18800 | 0.011970229 | 0.063104715 | 0.044164739 | CYP26B1/LY6E/RYR2/MEF2C/CDK5 | 5 |
| BP | GO:0043406 | positive regulation of MAP kinase activity | 3/77 | 116/18800 | 0.012024541 | 0.063134915 | 0.044185875 | ADRA2A/HTR2A/PTK2B | 3 |
| BP | GO:1904892 | regulation of receptor signaling pathway via STAT | 3/77 | 116/18800 | 0.012024541 | 0.063134915 | 0.044185875 | IL10RB/PTK2B/CDK5 | 3 |
| BP | GO:0071222 | cellular response to lipopolysaccharide | 4/77 | 217/18800 | 0.012124383 | 0.063530788 | 0.044462932 | ABL1/RELA/ABCA1/MEF2C | 4 |
| BP | GO:0009299 | mRNA transcription | 2/77 | 41/18800 | 0.012245279 | 0.063834842 | 0.044675728 | THRB/TP53 | 2 |
| BP | GO:0050892 | intestinal absorption | 2/77 | 41/18800 | 0.012245279 | 0.063834842 | 0.044675728 | ADRA2A/LPCAT3 | 2 |
| BP | GO:0042113 | B cell activation | 5/77 | 336/18800 | 0.012258065 | 0.063834842 | 0.044675728 | ABL1/PTK2B/TP53/TRAF3IP2/MEF2C | 5 |
| BP | GO:0007006 | mitochondrial membrane organization | 3/77 | 117/18800 | 0.012305216 | 0.063834842 | 0.044675728 | BCL2L2/TP53/MYC | 3 |
| BP | GO:0032411 | positive regulation of transporter activity | 3/77 | 117/18800 | 0.012305216 | 0.063834842 | 0.044675728 | CACNB2/RYR2/CDK5 | 3 |
| BP | GO:0030278 | regulation of ossification | 3/77 | 118/18800 | 0.01258964 | 0.06479302 | 0.045346323 | HIF1A/PTK2B/MEF2C | 3 |
| BP | GO:0098659 | inorganic cation import across plasma membrane | 3/77 | 118/18800 | 0.01258964 | 0.06479302 | 0.045346323 | KCNJ3/KCNJ6/SLC12A5 | 3 |
| BP | GO:0099587 | inorganic ion import across plasma membrane | 3/77 | 118/18800 | 0.01258964 | 0.06479302 | 0.045346323 | KCNJ3/KCNJ6/SLC12A5 | 3 |
| BP | GO:1904019 | epithelial cell apoptotic process | 3/77 | 118/18800 | 0.01258964 | 0.06479302 | 0.045346323 | ABL1/RYR2/JAG2 | 3 |
| BP | GO:0009167 | purine ribonucleoside monophosphate metabolic process | 2/77 | 42/18800 | 0.012823741 | 0.065608076 | 0.045916752 | CASK/IMPDH2 | 2 |
| BP | GO:0050999 | regulation of nitric-oxide synthase activity | 2/77 | 42/18800 | 0.012823741 | 0.065608076 | 0.045916752 | HIF1A/PTK2B | 2 |
| BP | GO:1900274 | regulation of phospholipase C activity | 2/77 | 42/18800 | 0.012823741 | 0.065608076 | 0.045916752 | ABL1/HTR2A | 2 |
| BP | GO:0045471 | response to ethanol | 3/77 | 119/18800 | 0.012877823 | 0.065626395 | 0.045929572 | GRIN1/PTK2B/MYC | 3 |
| BP | GO:2000278 | regulation of DNA biosynthetic process | 3/77 | 119/18800 | 0.012877823 | 0.065626395 | 0.045929572 | PTK2B/TP53/MYC | 3 |
| BP | GO:0001709 | cell fate determination | 2/77 | 43/18800 | 0.013413943 | 0.068087177 | 0.047651786 | CYP26B1/MEF2C | 2 |
| BP | GO:0030282 | bone mineralization | 3/77 | 121/18800 | 0.01346549 | 0.068087177 | 0.047651786 | HIF1A/PTK2B/MEF2C | 3 |
| BP | GO:0038127 | ERBB signaling pathway | 3/77 | 121/18800 | 0.01346549 | 0.068087177 | 0.047651786 | ABL1/ADRA2A/PTK2B | 3 |
| BP | GO:1903008 | organelle disassembly | 3/77 | 121/18800 | 0.01346549 | 0.068087177 | 0.047651786 | CAMKK2/HIF1A/TP53 | 3 |
| BP | GO:0000082 | G1/S transition of mitotic cell cycle | 4/77 | 225/18800 | 0.013689916 | 0.068953664 | 0.048258209 | PLK2/TP53/MYC/CDK5 | 4 |
| BP | GO:0002790 | peptide secretion | 4/77 | 225/18800 | 0.013689916 | 0.068953664 | 0.048258209 | ADRA2A/HIF1A/RBP4/ABCA1 | 4 |
| BP | GO:0007613 | memory | 3/77 | 122/18800 | 0.013764989 | 0.06919769 | 0.048428994 | ADCY1/HTR2A/PLK2 | 3 |
| BP | GO:0009743 | response to carbohydrate | 4/77 | 226/18800 | 0.013894222 | 0.069712514 | 0.048789301 | ADRA2A/HIF1A/PTK2B/MYC | 4 |
| BP | GO:0007265 | Ras protein signal transduction | 5/77 | 347/18800 | 0.013925762 | 0.069736139 | 0.048805836 | ABL1/ADRA2A/PLK2/TP53/ABCA1 | 5 |
| BP | GO:0009126 | purine nucleoside monophosphate metabolic process | 2/77 | 44/18800 | 0.014015781 | 0.069783552 | 0.048839018 | CASK/IMPDH2 | 2 |
| BP | GO:0014009 | glial cell proliferation | 2/77 | 44/18800 | 0.014015781 | 0.069783552 | 0.048839018 | PTK2B/MYC | 2 |
| BP | GO:0060119 | inner ear receptor cell development | 2/77 | 44/18800 | 0.014015781 | 0.069783552 | 0.048839018 | GABRB2/GABRA5 | 2 |
| BP | GO:0001952 | regulation of cell-matrix adhesion | 3/77 | 123/18800 | 0.014068272 | 0.069910975 | 0.048928197 | ABL1/CASK/PTK2B | 3 |
| BP | GO:0071219 | cellular response to molecule of bacterial origin | 4/77 | 229/18800 | 0.014518772 | 0.071516958 | 0.050052167 | ABL1/RELA/ABCA1/MEF2C | 4 |
| BP | GO:0071356 | cellular response to tumor necrosis factor | 4/77 | 229/18800 | 0.014518772 | 0.071516958 | 0.050052167 | PTK2B/TP53/TRAF3IP2/RELA | 4 |
| BP | GO:0001754 | eye photoreceptor cell differentiation | 2/77 | 45/18800 | 0.014629152 | 0.071516958 | 0.050052167 | RORB/THRB | 2 |
| BP | GO:0006984 | ER-nucleus signaling pathway | 2/77 | 45/18800 | 0.014629152 | 0.071516958 | 0.050052167 | TP53/LPCAT3 | 2 |
| BP | GO:0046427 | positive regulation of receptor signaling pathway via JAK-STAT | 2/77 | 45/18800 | 0.014629152 | 0.071516958 | 0.050052167 | IL10RB/PTK2B | 2 |
| BP | GO:1900271 | regulation of long-term synaptic potentiation | 2/77 | 45/18800 | 0.014629152 | 0.071516958 | 0.050052167 | ABL1/ADCY1 | 2 |
| BP | GO:1904646 | cellular response to amyloid-beta | 2/77 | 45/18800 | 0.014629152 | 0.071516958 | 0.050052167 | BCL2L2/CDK5 | 2 |
| BP | GO:2000273 | positive regulation of signaling receptor activity | 2/77 | 45/18800 | 0.014629152 | 0.071516958 | 0.050052167 | ADRA2A/HIF1A | 2 |
| BP | GO:0019221 | cytokine-mediated signaling pathway | 6/77 | 486/18800 | 0.0146391 | 0.071516958 | 0.050052167 | HIF1A/IL10RB/PTK2B/TP53/TRAF3IP2/RELA | 6 |
| BP | GO:0001655 | urogenital system development | 5/77 | 352/18800 | 0.014732115 | 0.071836338 | 0.05027569 | CYP26B1/TRAF3IP2/MYC/RBP4/MEF2C | 5 |
| BP | GO:0072593 | reactive oxygen species metabolic process | 4/77 | 231/18800 | 0.014944891 | 0.072737402 | 0.050906312 | GRIN1/HIF1A/PTK2B/TP53 | 4 |
| BP | GO:0001974 | blood vessel remodeling | 2/77 | 46/18800 | 0.015253956 | 0.073826874 | 0.051668795 | RSPO3/MEF2C | 2 |
| BP | GO:0046677 | response to antibiotic | 2/77 | 46/18800 | 0.015253956 | 0.073826874 | 0.051668795 | TP53/MEF2C | 2 |
| BP | GO:0090199 | regulation of release of cytochrome c from mitochondria | 2/77 | 46/18800 | 0.015253956 | 0.073826874 | 0.051668795 | BCL2L2/TP53 | 2 |
| BP | GO:1901652 | response to peptide | 6/77 | 491/18800 | 0.015325054 | 0.074033113 | 0.051813134 | BCL2L2/TP53/MYC/RELA/GOT1/CDK5 | 6 |
| BP | GO:0045732 | positive regulation of protein catabolic process | 4/77 | 233/18800 | 0.015378865 | 0.074155234 | 0.051898603 | ADRA2A/MAPK9/PLK2/PTK2B | 4 |
| BP | GO:0048738 | cardiac muscle tissue development | 4/77 | 234/18800 | 0.015598812 | 0.07495162 | 0.052455965 | LY6E/RYR2/RBP4/MEF2C | 4 |
| BP | GO:0042542 | response to hydrogen peroxide | 3/77 | 128/18800 | 0.015641677 | 0.07495162 | 0.052455965 | ABL1/PTK2B/RELA | 3 |
| BP | GO:0010631 | epithelial cell migration | 5/77 | 358/18800 | 0.015740554 | 0.07495162 | 0.052455965 | ABL1/HIF1A/PLK2/PTK2B/MEF2C | 5 |
| BP | GO:0007160 | cell-matrix adhesion | 4/77 | 235/18800 | 0.015820739 | 0.07495162 | 0.052455965 | ABL1/CASK/PTK2B/CDK5 | 4 |
| BP | GO:0097305 | response to alcohol | 4/77 | 235/18800 | 0.015820739 | 0.07495162 | 0.052455965 | ADCY1/GRIN1/PTK2B/MYC | 4 |
| BP | GO:0032365 | intracellular lipid transport | 2/77 | 47/18800 | 0.01589009 | 0.07495162 | 0.052455965 | FABP3/ABCA1 | 2 |
| BP | GO:0043618 | regulation of transcription from RNA polymerase II promoter in response to stress | 2/77 | 47/18800 | 0.01589009 | 0.07495162 | 0.052455965 | HIF1A/TP53 | 2 |
| BP | GO:0046717 | acid secretion | 2/77 | 47/18800 | 0.01589009 | 0.07495162 | 0.052455965 | CCKBR/MYC | 2 |
| BP | GO:0048066 | developmental pigmentation | 2/77 | 47/18800 | 0.01589009 | 0.07495162 | 0.052455965 | TYRP1/MEF2C | 2 |
| BP | GO:0051972 | regulation of telomerase activity | 2/77 | 47/18800 | 0.01589009 | 0.07495162 | 0.052455965 | TP53/MYC | 2 |
| BP | GO:0055010 | ventricular cardiac muscle tissue morphogenesis | 2/77 | 47/18800 | 0.01589009 | 0.07495162 | 0.052455965 | LY6E/RYR2 | 2 |
| BP | GO:2000378 | negative regulation of reactive oxygen species metabolic process | 2/77 | 47/18800 | 0.01589009 | 0.07495162 | 0.052455965 | HIF1A/TP53 | 2 |
| BP | GO:0090132 | epithelium migration | 5/77 | 361/18800 | 0.016261711 | 0.076565557 | 0.053585502 | ABL1/HIF1A/PLK2/PTK2B/MEF2C | 5 |
| BP | GO:0042311 | vasodilation | 2/77 | 48/18800 | 0.016537453 | 0.077582744 | 0.054297394 | ADRA2A/ADRB1 | 2 |
| BP | GO:0048146 | positive regulation of fibroblast proliferation | 2/77 | 48/18800 | 0.016537453 | 0.077582744 | 0.054297394 | ABL1/MYC | 2 |
| BP | GO:0090130 | tissue migration | 5/77 | 366/18800 | 0.017155749 | 0.079523599 | 0.05565573 | ABL1/HIF1A/PLK2/PTK2B/MEF2C | 5 |
| BP | GO:0035094 | response to nicotine | 2/77 | 49/18800 | 0.017195946 | 0.079523599 | 0.05565573 | GRM2/RELA | 2 |
| BP | GO:0042461 | photoreceptor cell development | 2/77 | 49/18800 | 0.017195946 | 0.079523599 | 0.05565573 | RORB/THRB | 2 |
| BP | GO:0050873 | brown fat cell differentiation | 2/77 | 49/18800 | 0.017195946 | 0.079523599 | 0.05565573 | ADRB1/FABP3 | 2 |
| BP | GO:0060043 | regulation of cardiac muscle cell proliferation | 2/77 | 49/18800 | 0.017195946 | 0.079523599 | 0.05565573 | RBP4/MEF2C | 2 |
| BP | GO:0090102 | cochlea development | 2/77 | 49/18800 | 0.017195946 | 0.079523599 | 0.05565573 | GABRB2/GABRA5 | 2 |
| BP | GO:0120009 | intermembrane lipid transfer | 2/77 | 49/18800 | 0.017195946 | 0.079523599 | 0.05565573 | PITPNM1/ABCA1 | 2 |
| BP | GO:1904894 | positive regulation of receptor signaling pathway via STAT | 2/77 | 49/18800 | 0.017195946 | 0.079523599 | 0.05565573 | IL10RB/PTK2B | 2 |
| BP | GO:0001889 | liver development | 3/77 | 134/18800 | 0.017655795 | 0.08136066 | 0.056941423 | IGF2R/MYC/RELA | 3 |
| BP | GO:2001235 | positive regulation of apoptotic signaling pathway | 3/77 | 134/18800 | 0.017655795 | 0.08136066 | 0.056941423 | MAPK9/TP53/MYC | 3 |
| BP | GO:0021695 | cerebellar cortex development | 2/77 | 50/18800 | 0.017865468 | 0.081747098 | 0.057211878 | KNDC1/CDK5 | 2 |
| BP | GO:0061178 | regulation of insulin secretion involved in cellular response to glucose stimulus | 2/77 | 50/18800 | 0.017865468 | 0.081747098 | 0.057211878 | ADRA2A/HIF1A | 2 |
| BP | GO:0070231 | T cell apoptotic process | 2/77 | 50/18800 | 0.017865468 | 0.081747098 | 0.057211878 | HIF1A/TP53 | 2 |
| BP | GO:1903307 | positive regulation of regulated secretory pathway | 2/77 | 50/18800 | 0.017865468 | 0.081747098 | 0.057211878 | CACNB2/CDK5 | 2 |
| BP | GO:0071900 | regulation of protein serine/threonine kinase activity | 5/77 | 372/18800 | 0.018271132 | 0.083456363 | 0.058408131 | ABL1/ADRA2A/HTR2A/PTK2B/CCNA1 | 5 |
| BP | GO:0010508 | positive regulation of autophagy | 3/77 | 136/18800 | 0.018357864 | 0.08370542 | 0.058582437 | CAMKK2/HIF1A/PLK2 | 3 |
| BP | GO:0007140 | male meiotic nuclear division | 2/77 | 51/18800 | 0.018545922 | 0.084267222 | 0.058975622 | CYP26B1/CCNA1 | 2 |
| BP | GO:0010823 | negative regulation of mitochondrion organization | 2/77 | 51/18800 | 0.018545922 | 0.084267222 | 0.058975622 | BCL2L2/TP53 | 2 |
| BP | GO:0061008 | hepaticobiliary system development | 3/77 | 137/18800 | 0.018714669 | 0.084885558 | 0.059408373 | IGF2R/MYC/RELA | 3 |
| BP | GO:0014706 | striated muscle tissue development | 4/77 | 248/18800 | 0.018888379 | 0.08552421 | 0.059855343 | LY6E/RYR2/RBP4/MEF2C | 4 |
| BP | GO:0007266 | Rho protein signal transduction | 3/77 | 138/18800 | 0.019075324 | 0.086057338 | 0.060228461 | ABL1/ADRA2A/ABCA1 | 3 |
| BP | GO:0031644 | regulation of nervous system process | 3/77 | 138/18800 | 0.019075324 | 0.086057338 | 0.060228461 | GRIN1/PTK2B/MGLL | 3 |
| BP | GO:0015833 | peptide transport | 4/77 | 249/18800 | 0.019138569 | 0.086057338 | 0.060228461 | ADRA2A/HIF1A/RBP4/ABCA1 | 4 |
| BP | GO:0034612 | response to tumor necrosis factor | 4/77 | 249/18800 | 0.019138569 | 0.086057338 | 0.060228461 | PTK2B/TP53/TRAF3IP2/RELA | 4 |
| BP | GO:0038093 | Fc receptor signaling pathway | 2/77 | 52/18800 | 0.019237208 | 0.086202593 | 0.060330119 | ABL1/MAPK9 | 2 |
| BP | GO:1902930 | regulation of alcohol biosynthetic process | 2/77 | 52/18800 | 0.019237208 | 0.086202593 | 0.060330119 | PTK2B/LPCAT3 | 2 |
| BP | GO:0008203 | cholesterol metabolic process | 3/77 | 139/18800 | 0.019439831 | 0.086960622 | 0.060860637 | LCAT/LPCAT3/ABCA1 | 3 |
| BP | GO:0001654 | eye development | 5/77 | 379/18800 | 0.019632015 | 0.087669427 | 0.061356704 | HIF1A/IMPDH2/RORB/THRB/RBP4 | 5 |
| BP | GO:0002931 | response to ischemia | 2/77 | 53/18800 | 0.019939229 | 0.088736399 | 0.06210344 | BCL2L2/TP53 | 2 |
| BP | GO:0060071 | Wnt signaling pathway, planar cell polarity pathway | 2/77 | 53/18800 | 0.019939229 | 0.088736399 | 0.06210344 | ABL1/RSPO3 | 2 |
| BP | GO:0070555 | response to interleukin-1 | 3/77 | 141/18800 | 0.020180412 | 0.089656225 | 0.062747193 | HIF1A/MYC/RELA | 3 |
| BP | GO:0043122 | regulation of I-kappaB kinase/NF-kappaB signaling | 4/77 | 254/18800 | 0.020420428 | 0.09049524 | 0.06333439 | ABL1/PLK2/TRAF3IP2/RELA | 4 |
| BP | GO:0150063 | visual system development | 5/77 | 383/18800 | 0.020438902 | 0.09049524 | 0.06333439 | HIF1A/IMPDH2/RORB/THRB/RBP4 | 5 |
| BP | GO:0006968 | cellular defense response | 2/77 | 54/18800 | 0.020651888 | 0.090956252 | 0.063657035 | PTK2B/RELA | 2 |
| BP | GO:0010524 | positive regulation of calcium ion transport into cytosol | 2/77 | 54/18800 | 0.020651888 | 0.090956252 | 0.063657035 | ABL1/GRIN1 | 2 |
| BP | GO:0051155 | positive regulation of striated muscle cell differentiation | 2/77 | 54/18800 | 0.020651888 | 0.090956252 | 0.063657035 | CYP26B1/MEF2C | 2 |
| BP | GO:0044843 | cell cycle G1/S phase transition | 4/77 | 255/18800 | 0.020683011 | 0.090956252 | 0.063657035 | PLK2/TP53/MYC/CDK5 | 4 |
| BP | GO:0030010 | establishment of cell polarity | 3/77 | 143/18800 | 0.020936426 | 0.091605676 | 0.064111544 | CYP26B1/PTK2B/TRAF3IP2 | 3 |
| BP | GO:0046165 | alcohol biosynthetic process | 3/77 | 143/18800 | 0.020936426 | 0.091605676 | 0.064111544 | PTK2B/LPCAT3/GOT1 | 3 |
| BP | GO:0071456 | cellular response to hypoxia | 3/77 | 143/18800 | 0.020936426 | 0.091605676 | 0.064111544 | HIF1A/TP53/MYC | 3 |
| BP | GO:0001954 | positive regulation of cell-matrix adhesion | 2/77 | 55/18800 | 0.021375089 | 0.093211168 | 0.065235171 | ABL1/PTK2B | 2 |
| BP | GO:1904645 | response to amyloid-beta | 2/77 | 55/18800 | 0.021375089 | 0.093211168 | 0.065235171 | BCL2L2/CDK5 | 2 |
| BP | GO:0006644 | phospholipid metabolic process | 5/77 | 388/18800 | 0.021477803 | 0.093502196 | 0.065438851 | HTR2A/LCAT/PITPNM1/FABP3/LPCAT3 | 5 |
| BP | GO:0048880 | sensory system development | 5/77 | 389/18800 | 0.021689654 | 0.094031314 | 0.065809162 | HIF1A/IMPDH2/RORB/THRB/RBP4 | 5 |
| BP | GO:0006865 | amino acid transport | 3/77 | 145/18800 | 0.021707883 | 0.094031314 | 0.065809162 | GRM2/SLC1A2/MYC | 3 |
| BP | GO:0015748 | organophosphate ester transport | 3/77 | 145/18800 | 0.021707883 | 0.094031314 | 0.065809162 | PCTP/PITPNM1/ABCA1 | 3 |
| BP | GO:0009411 | response to UV | 3/77 | 146/18800 | 0.022099405 | 0.094544824 | 0.066168549 | TP53/MYC/RELA | 3 |
| BP | GO:0062013 | positive regulation of small molecule metabolic process | 3/77 | 146/18800 | 0.022099405 | 0.094544824 | 0.066168549 | HIF1A/HTR2A/MYC | 3 |
| BP | GO:0106106 | cold-induced thermogenesis | 3/77 | 146/18800 | 0.022099405 | 0.094544824 | 0.066168549 | ADRB1/PCTP/PTH2R | 3 |
| BP | GO:0120161 | regulation of cold-induced thermogenesis | 3/77 | 146/18800 | 0.022099405 | 0.094544824 | 0.066168549 | ADRB1/PCTP/PTH2R | 3 |
| BP | GO:0001836 | release of cytochrome c from mitochondria | 2/77 | 56/18800 | 0.022108735 | 0.094544824 | 0.066168549 | BCL2L2/TP53 | 2 |
| BP | GO:0003229 | ventricular cardiac muscle tissue development | 2/77 | 56/18800 | 0.022108735 | 0.094544824 | 0.066168549 | LY6E/RYR2 | 2 |
| BP | GO:0010332 | response to gamma radiation | 2/77 | 56/18800 | 0.022108735 | 0.094544824 | 0.066168549 | TP53/MYC | 2 |
| BP | GO:0030099 | myeloid cell differentiation | 5/77 | 391/18800 | 0.02211745 | 0.094544824 | 0.066168549 | CBFB/HIF1A/PTK2B/MYC/MEF2C | 5 |
| BP | GO:0005996 | monosaccharide metabolic process | 4/77 | 261/18800 | 0.022302327 | 0.095178569 | 0.066612085 | GSTO2/TP53/MYC/RBP4 | 4 |
| BP | GO:0050796 | regulation of insulin secretion | 3/77 | 147/18800 | 0.02249479 | 0.095685695 | 0.066967004 | ADRA2A/HIF1A/RBP4 | 3 |
| BP | GO:0071322 | cellular response to carbohydrate stimulus | 3/77 | 147/18800 | 0.02249479 | 0.095685695 | 0.066967004 | ADRA2A/HIF1A/MYC | 3 |
| BP | GO:0032768 | regulation of monooxygenase activity | 2/77 | 57/18800 | 0.022852731 | 0.096576012 | 0.067590105 | HIF1A/PTK2B | 2 |
| BP | GO:0043551 | regulation of phosphatidylinositol 3-kinase activity | 2/77 | 57/18800 | 0.022852731 | 0.096576012 | 0.067590105 | CCKBR/PTK2B | 2 |
| BP | GO:0070228 | regulation of lymphocyte apoptotic process | 2/77 | 57/18800 | 0.022852731 | 0.096576012 | 0.067590105 | HIF1A/TP53 | 2 |
| BP | GO:0090175 | regulation of establishment of planar polarity | 2/77 | 57/18800 | 0.022852731 | 0.096576012 | 0.067590105 | ABL1/RSPO3 | 2 |
| BP | GO:0030522 | intracellular receptor signaling pathway | 4/77 | 264/18800 | 0.023140327 | 0.097632646 | 0.068329605 | CYP26B1/RORB/THRB/RELA | 4 |
| BP | GO:1902652 | secondary alcohol metabolic process | 3/77 | 149/18800 | 0.02329715 | 0.098134998 | 0.068681184 | LCAT/LPCAT3/ABCA1 | 3 |
| BP | GO:0045761 | regulation of adenylate cyclase activity | 2/77 | 58/18800 | 0.023606982 | 0.098958946 | 0.069257836 | ADRB1/GRM2 | 2 |
| BP | GO:0048010 | vascular endothelial growth factor receptor signaling pathway | 2/77 | 58/18800 | 0.023606982 | 0.098958946 | 0.069257836 | HIF1A/PTK2B | 2 |
| BP | GO:0060038 | cardiac muscle cell proliferation | 2/77 | 58/18800 | 0.023606982 | 0.098958946 | 0.069257836 | RBP4/MEF2C | 2 |
| BP | GO:0016331 | morphogenesis of embryonic epithelium | 3/77 | 150/18800 | 0.023704126 | 0.099069354 | 0.069335106 | ABL1/HIF1A/JAG2 | 3 |
| BP | GO:0045165 | cell fate commitment | 4/77 | 266/18800 | 0.023709557 | 0.099069354 | 0.069335106 | CYP26B1/TP53/JAG2/MEF2C | 4 |
| CC | GO:0097060 | synaptic membrane | 15/77 | 373/19594 | 1.42732E-11 | 3.33994E-09 | 2.23864E-09 | ADCY1/ADRA2A/CASK/CNKSR2/GABRA1/GABRA2/GABRB2/GAD2/GRIN1/GRM2/HTR2A/KCNA2/KCNJ3/SLC1A2/GABRA5 | 15 |
| CC | GO:0034702 | ion channel complex | 13/77 | 294/19594 | 1.16785E-10 | 1.36639E-08 | 9.15841E-09 | CACNB2/GABRA1/GABRA2/GABRB2/GRIN1/KCNA2/KCNJ3/KCNJ6/KCNN1/PTK2B/RYR2/SCN2B/GABRA5 | 13 |
| CC | GO:0099699 | integral component of synaptic membrane | 10/77 | 149/19594 | 3.38444E-10 | 2.63986E-08 | 1.76941E-08 | ADCY1/ADRA2A/GABRA2/GABRB2/GRM2/HTR2A/KCNA2/KCNJ3/SLC1A2/GABRA5 | 10 |
| CC | GO:0099240 | intrinsic component of synaptic membrane | 10/77 | 160/19594 | 6.81248E-10 | 3.9853E-08 | 2.67121E-08 | ADCY1/ADRA2A/GABRA2/GABRB2/GRM2/HTR2A/KCNA2/KCNJ3/SLC1A2/GABRA5 | 10 |
| CC | GO:1902495 | transmembrane transporter complex | 13/77 | 377/19594 | 2.43595E-09 | 1.14002E-07 | 7.64118E-08 | CACNB2/GABRA1/GABRA2/GABRB2/GRIN1/KCNA2/KCNJ3/KCNJ6/KCNN1/PTK2B/RYR2/SCN2B/GABRA5 | 13 |
| CC | GO:1990351 | transporter complex | 13/77 | 399/19594 | 4.81462E-09 | 1.61308E-07 | 1.08119E-07 | CACNB2/GABRA1/GABRA2/GABRB2/GRIN1/KCNA2/KCNJ3/KCNJ6/KCNN1/PTK2B/RYR2/SCN2B/GABRA5 | 13 |
| CC | GO:0042734 | presynaptic membrane | 9/77 | 143/19594 | 4.82546E-09 | 1.61308E-07 | 1.08119E-07 | ADRA2A/CASK/GAD2/GRM2/HTR2A/KCNA2/KCNJ3/SLC1A2/GABRA5 | 9 |
| CC | GO:0098793 | presynapse | 13/77 | 492/19594 | 5.72028E-08 | 1.67318E-06 | 1.12148E-06 | ADRA2A/CACNB2/CASK/GABRA2/GAD2/GRIN1/GRM2/HTR2A/KCNA2/KCNJ3/SLC1A2/GABRA5/CDK5 | 13 |
| CC | GO:0045211 | postsynaptic membrane | 10/77 | 271/19594 | 1.05367E-07 | 2.73954E-06 | 1.83622E-06 | ADCY1/ADRA2A/CNKSR2/GABRA1/GABRA2/GABRB2/GRIN1/GRM2/HTR2A/GABRA5 | 10 |
| CC | GO:0034703 | cation channel complex | 9/77 | 221/19594 | 2.08297E-07 | 4.87415E-06 | 3.26697E-06 | CACNB2/GRIN1/KCNA2/KCNJ3/KCNJ6/KCNN1/PTK2B/RYR2/SCN2B | 9 |
| CC | GO:0099056 | integral component of presynaptic membrane | 6/77 | 67/19594 | 2.49216E-07 | 5.30151E-06 | 3.55342E-06 | ADRA2A/HTR2A/KCNA2/KCNJ3/SLC1A2/GABRA5 | 6 |
| CC | GO:0043025 | neuronal cell body | 12/77 | 482/19594 | 3.69292E-07 | 7.20119E-06 | 4.82671E-06 | ABL1/ADRA2A/CNKSR2/GABRA2/HTR2A/KCNA2/KCNN1/PTK2B/SLC12A5/KNDC1/GABRA5/CDK5 | 12 |
| CC | GO:0099055 | integral component of postsynaptic membrane | 7/77 | 118/19594 | 4.07544E-07 | 7.3358E-06 | 4.91693E-06 | ADCY1/ADRA2A/GABRA2/GABRB2/GRM2/HTR2A/GABRA5 | 7 |
| CC | GO:0098889 | intrinsic component of presynaptic membrane | 6/77 | 74/19594 | 4.52666E-07 | 7.56599E-06 | 5.07122E-06 | ADRA2A/HTR2A/KCNA2/KCNJ3/SLC1A2/GABRA5 | 6 |
| CC | GO:0098936 | intrinsic component of postsynaptic membrane | 7/77 | 123/19594 | 5.40506E-07 | 8.43189E-06 | 5.6516E-06 | ADCY1/ADRA2A/GABRA2/GABRB2/GRM2/HTR2A/GABRA5 | 7 |
| CC | GO:1902711 | GABA-A receptor complex | 4/77 | 19/19594 | 8.16928E-07 | 1.19476E-05 | 8.00804E-06 | GABRA1/GABRA2/GABRB2/GABRA5 | 4 |
| CC | GO:1902710 | GABA receptor complex | 4/77 | 21/19594 | 1.25393E-06 | 1.726E-05 | 1.15688E-05 | GABRA1/GABRA2/GABRB2/GABRA5 | 4 |
| CC | GO:0032589 | neuron projection membrane | 5/77 | 58/19594 | 3.19938E-06 | 4.15919E-05 | 2.78776E-05 | GABRA1/GABRA2/SLC12A5/SLC1A2/GABRA5 | 5 |
| CC | GO:0031252 | cell leading edge | 10/77 | 416/19594 | 5.15494E-06 | 6.34871E-05 | 4.25532E-05 | ABL1/ATP6V1B2/GABRA1/GABRA2/KCNA2/PTK2B/SLC12A5/SLC1A2/GABRA5/CDK5 | 10 |
| CC | GO:0099634 | postsynaptic specialization membrane | 6/77 | 116/19594 | 6.36385E-06 | 7.44571E-05 | 4.9906E-05 | ADCY1/ADRA2A/CNKSR2/GABRA2/GABRB2/GABRA5 | 6 |
| CC | GO:0099572 | postsynaptic specialization | 9/77 | 341/19594 | 7.51169E-06 | 8.37017E-05 | 5.61023E-05 | ADCY1/ADRA2A/CNKSR2/GABRA2/GABRB2/GRIN1/PTK2B/GABRA5/CDK5 | 9 |
| CC | GO:0098982 | GABA-ergic synapse | 5/77 | 70/19594 | 8.14609E-06 | 8.66448E-05 | 5.8075E-05 | ADRA2A/GABRA1/GABRA2/GABRB2/GABRA5 | 5 |
| CC | GO:0099060 | integral component of postsynaptic specialization membrane | 5/77 | 77/19594 | 1.30166E-05 | 0.00013243 | 8.87632E-05 | ADCY1/ADRA2A/GABRA2/GABRB2/GABRA5 | 5 |
| CC | GO:0098948 | intrinsic component of postsynaptic specialization membrane | 5/77 | 80/19594 | 1.56932E-05 | 0.000153009 | 0.000102556 | ADCY1/ADRA2A/GABRA2/GABRB2/GABRA5 | 5 |
| CC | GO:0032590 | dendrite membrane | 4/77 | 41/19594 | 1.99901E-05 | 0.000187108 | 0.000125412 | GABRA1/GABRA2/SLC12A5/GABRA5 | 4 |
| CC | GO:0098978 | glutamatergic synapse | 8/77 | 319/19594 | 3.58717E-05 | 0.000322845 | 0.000216392 | ADCY1/ADRA2A/CNKSR2/HTR2A/PTK2B/SLC1A2/RELA/CDK5 | 8 |
| CC | GO:0034707 | chloride channel complex | 4/77 | 52/19594 | 5.17191E-05 | 0.000448233 | 0.000300435 | GABRA1/GABRA2/GABRB2/GABRA5 | 4 |
| CC | GO:0031256 | leading edge membrane | 6/77 | 175/19594 | 6.534E-05 | 0.000546056 | 0.000366003 | GABRA1/GABRA2/KCNA2/SLC12A5/SLC1A2/GABRA5 | 6 |
| CC | GO:0098685 | Schaffer collateral - CA1 synapse | 4/77 | 72/19594 | 0.000185201 | 0.001494384 | 0.001001634 | ADCY1/ADRB1/CASK/CDK5 | 4 |
| CC | GO:0008076 | voltage-gated potassium channel complex | 4/77 | 79/19594 | 0.000264918 | 0.00206636 | 0.00138501 | KCNA2/KCNJ3/KCNJ6/KCNN1 | 4 |
| CC | GO:0031253 | cell projection membrane | 7/77 | 339/19594 | 0.000371585 | 0.002804869 | 0.001880007 | CASK/GABRA1/GABRA2/KCNA2/SLC12A5/SLC1A2/GABRA5 | 7 |
| CC | GO:0034705 | potassium channel complex | 4/77 | 89/19594 | 0.000417925 | 0.003056079 | 0.002048384 | KCNA2/KCNJ3/KCNJ6/KCNN1 | 4 |
| CC | GO:0150034 | distal axon | 6/77 | 270/19594 | 0.000677331 | 0.00480289 | 0.003219211 | ABL1/ADRA2A/GRIN1/KCNA2/PTK2B/CDK5 | 6 |
| CC | GO:0014069 | postsynaptic density | 6/77 | 318/19594 | 0.001572255 | 0.010820811 | 0.007252815 | ADCY1/ADRA2A/CNKSR2/GRIN1/PTK2B/CDK5 | 6 |
| CC | GO:0044306 | neuron projection terminus | 4/77 | 129/19594 | 0.001674146 | 0.011058934 | 0.007412421 | ADRA2A/GRIN1/KCNA2/SLC1A2 | 4 |
| CC | GO:0032279 | asymmetric synapse | 6/77 | 323/19594 | 0.001701375 | 0.011058934 | 0.007412421 | ADCY1/ADRA2A/CNKSR2/GRIN1/PTK2B/CDK5 | 6 |
| CC | GO:0098984 | neuron to neuron synapse | 6/77 | 347/19594 | 0.00243696 | 0.014976295 | 0.010038092 | ADCY1/ADRA2A/CNKSR2/GRIN1/PTK2B/CDK5 | 6 |
| CC | GO:0060077 | inhibitory synapse | 2/77 | 19/19594 | 0.002496049 | 0.014976295 | 0.010038092 | GABRA2/GAD2 | 2 |
| CC | GO:0097449 | astrocyte projection | 2/77 | 19/19594 | 0.002496049 | 0.014976295 | 0.010038092 | GRM2/SLC1A2 | 2 |
| CC | GO:0043204 | perikaryon | 4/77 | 153/19594 | 0.00310945 | 0.018190283 | 0.012192318 | KCNA2/SLC12A5/KNDC1/CDK5 | 4 |
| CC | GO:0098839 | postsynaptic density membrane | 3/77 | 89/19594 | 0.005200378 | 0.029680206 | 0.019893616 | ADCY1/ADRA2A/CNKSR2 | 3 |
| CC | GO:0032809 | neuronal cell body membrane | 2/77 | 28/19594 | 0.005392877 | 0.030046032 | 0.020138816 | KCNA2/GABRA5 | 2 |
| CC | GO:0044298 | cell body membrane | 2/77 | 31/19594 | 0.006583821 | 0.035828235 | 0.024014427 | KCNA2/GABRA5 | 2 |
| CC | GO:0097386 | glial cell projection | 2/77 | 33/19594 | 0.007438033 | 0.039556814 | 0.026513564 | GRM2/SLC1A2 | 2 |
| CC | GO:0045121 | membrane raft | 5/77 | 326/19594 | 0.00920355 | 0.047402046 | 0.031771951 | ADCY1/HTR2A/PTK2B/SLC1A2/ABCA1 | 5 |
| CC | GO:0098857 | membrane microdomain | 5/77 | 327/19594 | 0.009318351 | 0.047402046 | 0.031771951 | ADCY1/HTR2A/PTK2B/SLC1A2/ABCA1 | 5 |
| CC | GO:0030665 | clathrin-coated vesicle membrane | 3/77 | 111/19594 | 0.00955187 | 0.047556118 | 0.03187522 | GAD2/IGF2R/TYRP1 | 3 |
| CC | GO:0043679 | axon terminus | 3/77 | 113/19594 | 0.010026366 | 0.048878535 | 0.032761591 | ADRA2A/GRIN1/KCNA2 | 3 |
| CC | GO:0008328 | ionotropic glutamate receptor complex | 2/77 | 40/19594 | 0.01079508 | 0.051552017 | 0.034553534 | GRIN1/PTK2B | 2 |
| CC | GO:0005667 | transcription regulator complex | 6/77 | 483/19594 | 0.011816003 | 0.055298895 | 0.037064936 | CBFB/HIF1A/THRB/TP53/MYC/RELA | 6 |
| CC | GO:0090575 | RNA polymerase II transcription regulator complex | 4/77 | 230/19594 | 0.012837756 | 0.058902646 | 0.039480406 | CBFB/HIF1A/THRB/MYC | 4 |
| CC | GO:0098878 | neurotransmitter receptor complex | 2/77 | 45/19594 | 0.013529482 | 0.060882671 | 0.040807548 | GRIN1/PTK2B | 2 |
| CC | GO:0042383 | sarcolemma | 3/77 | 131/19594 | 0.01491305 | 0.065842522 | 0.044131965 | CACNB2/KCNJ3/RYR2 | 3 |
| CC | GO:0000307 | cyclin-dependent protein kinase holoenzyme complex | 2/77 | 49/19594 | 0.015910026 | 0.068943446 | 0.046210407 | CCNA1/CDK5 | 2 |
| CC | GO:0030315 | T-tubule | 2/77 | 51/19594 | 0.017162655 | 0.070457217 | 0.047225035 | CACNB2/KCNJ3 | 2 |
| CC | GO:0060076 | excitatory synapse | 2/77 | 51/19594 | 0.017162655 | 0.070457217 | 0.047225035 | GRIN1/KCNJ3 | 2 |
| CC | GO:0099061 | integral component of postsynaptic density membrane | 2/77 | 51/19594 | 0.017162655 | 0.070457217 | 0.047225035 | ADCY1/ADRA2A | 2 |
| CC | GO:0099146 | intrinsic component of postsynaptic density membrane | 2/77 | 54/19594 | 0.01911756 | 0.077129466 | 0.051697213 | ADCY1/ADRA2A | 2 |
| CC | GO:0044304 | main axon | 2/77 | 61/19594 | 0.024021413 | 0.095271366 | 0.063857101 | KCNA2/SLC1A2 | 2 |
| CC | GO:0030426 | growth cone | 3/77 | 161/19594 | 0.025585 | 0.099126657 | 0.066441169 | ABL1/PTK2B/CDK5 | 3 |
| CC | GO:0005778 | peroxisomal membrane | 2/77 | 64/19594 | 0.026264328 | 0.099126657 | 0.066441169 | IMPDH2/SLC27A2 | 2 |
| CC | GO:0031903 | microbody membrane | 2/77 | 64/19594 | 0.026264328 | 0.099126657 | 0.066441169 | IMPDH2/SLC27A2 | 2 |
| MF | GO:0022836 | gated channel activity | 15/77 | 340/18410 | 9.13732E-12 | 3.30771E-09 | 2.26028E-09 | CACNB2/GABRA1/GABRA2/GABRB2/GRIN1/KCNA2/KCNH3/KCNJ3/KCNJ6/KCNN1/PTK2B/RYR2/SCN2B/GABRA5/CDK5 | 15 |
| MF | GO:0005216 | ion channel activity | 16/77 | 442/18410 | 3.3258E-11 | 6.0197E-09 | 4.11349E-09 | CACNB2/GABRA1/GABRA2/GABRB2/GRIN1/KCNA2/KCNH3/KCNJ3/KCNJ6/KCNN1/PTK2B/RYR2/SCN2B/SLC12A5/GABRA5/CDK5 | 16 |
| MF | GO:0015267 | channel activity | 16/77 | 489/18410 | 1.48154E-10 | 1.38165E-08 | 9.44132E-09 | CACNB2/GABRA1/GABRA2/GABRB2/GRIN1/KCNA2/KCNH3/KCNJ3/KCNJ6/KCNN1/PTK2B/RYR2/SCN2B/SLC12A5/GABRA5/CDK5 | 16 |
| MF | GO:0022803 | passive transmembrane transporter activity | 16/77 | 490/18410 | 1.52668E-10 | 1.38165E-08 | 9.44132E-09 | CACNB2/GABRA1/GABRA2/GABRB2/GRIN1/KCNA2/KCNH3/KCNJ3/KCNJ6/KCNN1/PTK2B/RYR2/SCN2B/SLC12A5/GABRA5/CDK5 | 16 |
| MF | GO:0015276 | ligand-gated ion channel activity | 10/77 | 145/18410 | 4.69787E-10 | 2.83438E-08 | 1.93684E-08 | GABRA1/GABRA2/GABRB2/GRIN1/KCNJ3/KCNJ6/KCNN1/PTK2B/RYR2/GABRA5 | 10 |
| MF | GO:0022834 | ligand-gated channel activity | 10/77 | 145/18410 | 4.69787E-10 | 2.83438E-08 | 1.93684E-08 | GABRA1/GABRA2/GABRB2/GRIN1/KCNJ3/KCNJ6/KCNN1/PTK2B/RYR2/GABRA5 | 10 |
| MF | GO:0022843 | voltage-gated cation channel activity | 9/77 | 144/18410 | 8.7505E-09 | 4.4953E-07 | 3.07181E-07 | CACNB2/GRIN1/KCNA2/KCNH3/KCNJ3/KCNJ6/KCNN1/PTK2B/CDK5 | 9 |
| MF | GO:0005244 | voltage-gated ion channel activity | 10/77 | 201/18410 | 1.11762E-08 | 4.4953E-07 | 3.07181E-07 | CACNB2/GRIN1/KCNA2/KCNH3/KCNJ3/KCNJ6/KCNN1/PTK2B/SCN2B/CDK5 | 10 |
| MF | GO:0022832 | voltage-gated channel activity | 10/77 | 201/18410 | 1.11762E-08 | 4.4953E-07 | 3.07181E-07 | CACNB2/GRIN1/KCNA2/KCNH3/KCNJ3/KCNJ6/KCNN1/PTK2B/SCN2B/CDK5 | 10 |
| MF | GO:0005261 | cation channel activity | 12/77 | 345/18410 | 1.91075E-08 | 6.63324E-07 | 4.53275E-07 | CACNB2/GRIN1/KCNA2/KCNH3/KCNJ3/KCNJ6/KCNN1/PTK2B/RYR2/SCN2B/SLC12A5/CDK5 | 12 |
| MF | GO:0030594 | neurotransmitter receptor activity | 8/77 | 111/18410 | 2.01563E-08 | 6.63324E-07 | 4.53275E-07 | ADRB1/GABRA1/GABRA2/GABRB2/GRIN1/HTR2A/PTK2B/GABRA5 | 8 |
| MF | GO:0022824 | transmitter-gated ion channel activity | 6/77 | 60/18410 | 1.83729E-07 | 4.83863E-06 | 3.30642E-06 | GABRA1/GABRA2/GABRB2/GRIN1/PTK2B/GABRA5 | 6 |
| MF | GO:0022835 | transmitter-gated channel activity | 6/77 | 60/18410 | 1.83729E-07 | 4.83863E-06 | 3.30642E-06 | GABRA1/GABRA2/GABRB2/GRIN1/PTK2B/GABRA5 | 6 |
| MF | GO:0022851 | GABA-gated chloride ion channel activity | 4/77 | 13/18410 | 1.96523E-07 | 4.83863E-06 | 3.30642E-06 | GABRA1/GABRA2/GABRB2/GABRA5 | 4 |
| MF | GO:0046873 | metal ion transmembrane transporter activity | 12/77 | 428/18410 | 2.00496E-07 | 4.83863E-06 | 3.30642E-06 | CACNB2/GRIN1/KCNA2/KCNH3/KCNJ3/KCNJ6/KCNN1/RYR2/SCN2B/SLC12A5/SLC1A2/CDK5 | 12 |
| MF | GO:0005237 | inhibitory extracellular ligand-gated ion channel activity | 4/77 | 15/18410 | 3.72805E-07 | 8.43471E-06 | 5.76376E-06 | GABRA1/GABRA2/GABRB2/GABRA5 | 4 |
| MF | GO:0005230 | extracellular ligand-gated ion channel activity | 6/77 | 73/18410 | 5.98433E-07 | 1.27431E-05 | 8.70785E-06 | GABRA1/GABRA2/GABRB2/GRIN1/PTK2B/GABRA5 | 6 |
| MF | GO:0099095 | ligand-gated anion channel activity | 4/77 | 18/18410 | 8.27816E-07 | 1.66483E-05 | 1.13764E-05 | GABRA1/GABRA2/GABRB2/GABRA5 | 4 |
| MF | GO:0004890 | GABA-A receptor activity | 4/77 | 19/18410 | 1.04524E-06 | 1.99147E-05 | 1.36084E-05 | GABRA1/GABRA2/GABRB2/GABRA5 | 4 |
| MF | GO:0099529 | neurotransmitter receptor activity involved in regulation of postsynaptic membrane potential | 5/77 | 49/18410 | 1.85302E-06 | 3.35396E-05 | 2.29189E-05 | ADRB1/GABRA1/GABRA2/GABRB2/GABRA5 | 5 |
| MF | GO:0016917 | GABA receptor activity | 4/77 | 22/18410 | 1.95395E-06 | 3.36824E-05 | 2.30165E-05 | GABRA1/GABRA2/GABRB2/GABRA5 | 4 |
| MF | GO:0098960 | postsynaptic neurotransmitter receptor activity | 5/77 | 64/18410 | 7.05584E-06 | 0.000116101 | 7.9336E-05 | ADRB1/GABRA1/GABRA2/GABRB2/GABRA5 | 5 |
| MF | GO:0099094 | ligand-gated cation channel activity | 6/77 | 115/18410 | 8.60939E-06 | 0.000135504 | 9.25953E-05 | GRIN1/KCNJ3/KCNJ6/KCNN1/PTK2B/RYR2 | 6 |
| MF | GO:0008503 | benzodiazepine receptor activity | 3/77 | 11/18410 | 1.13311E-05 | 0.000170911 | 0.00011679 | GABRA1/GABRA2/GABRA5 | 3 |
| MF | GO:0004712 | protein serine/threonine/tyrosine kinase activity | 10/77 | 446/18410 | 1.6196E-05 | 0.000234517 | 0.000160255 | ABL1/CAMKK2/CASK/CDKL2/MAPK9/PLK2/PTK2B/STK10/STK17B/CDK5 | 10 |
| MF | GO:0043177 | organic acid binding | 6/77 | 141/18410 | 2.75152E-05 | 0.000383096 | 0.000261784 | CYP26B1/GAD2/GRIN1/IGF2R/RYR2/FABP3 | 6 |
| MF | GO:0005249 | voltage-gated potassium channel activity | 5/77 | 91/18410 | 3.94167E-05 | 0.000522269 | 0.000356886 | KCNA2/KCNH3/KCNJ3/KCNJ6/KCNN1 | 5 |
| MF | GO:1904315 | transmitter-gated ion channel activity involved in regulation of postsynaptic membrane potential | 4/77 | 46/18410 | 4.03965E-05 | 0.000522269 | 0.000356886 | GABRA1/GABRA2/GABRB2/GABRA5 | 4 |
| MF | GO:0015079 | potassium ion transmembrane transporter activity | 6/77 | 154/18410 | 4.51595E-05 | 0.000553388 | 0.000378151 | KCNA2/KCNH3/KCNJ3/KCNJ6/KCNN1/SLC12A5 | 6 |
| MF | GO:0008525 | phosphatidylcholine transporter activity | 3/77 | 17/18410 | 4.58609E-05 | 0.000553388 | 0.000378151 | PCTP/PITPNM1/ABCA1 | 3 |
| MF | GO:0015108 | chloride transmembrane transporter activity | 5/77 | 99/18410 | 5.90661E-05 | 0.00068974 | 0.000471326 | GABRA1/GABRA2/GABRB2/SLC12A5/GABRA5 | 5 |
| MF | GO:0004674 | protein serine/threonine kinase activity | 9/77 | 430/18410 | 7.53293E-05 | 0.000852163 | 0.000582315 | CAMKK2/CASK/CDKL2/MAPK9/PLK2/PTK2B/STK10/STK17B/CDK5 | 9 |
| MF | GO:0106310 | protein serine kinase activity | 8/77 | 360/18410 | 0.000128542 | 0.001410064 | 0.00096355 | CAMKK2/CASK/CDKL2/MAPK9/PLK2/STK10/STK17B/CDK5 | 8 |
| MF | GO:0005267 | potassium channel activity | 5/77 | 121/18410 | 0.000152848 | 0.001627385 | 0.001112055 | KCNA2/KCNH3/KCNJ3/KCNJ6/KCNN1 | 5 |
| MF | GO:0005516 | calmodulin binding | 6/77 | 200/18410 | 0.000190491 | 0.001872798 | 0.001279754 | ADCY1/CAMKK2/CASK/GRIN1/KCNN1/RYR2 | 6 |
| MF | GO:0005242 | inward rectifier potassium channel activity | 3/77 | 27/18410 | 0.000191419 | 0.001872798 | 0.001279754 | KCNJ3/KCNJ6/KCNN1 | 3 |
| MF | GO:0008066 | glutamate receptor activity | 3/77 | 27/18410 | 0.000191419 | 0.001872798 | 0.001279754 | GRIN1/GRM2/PTK2B | 3 |
| MF | GO:0005254 | chloride channel activity | 4/77 | 74/18410 | 0.000260704 | 0.002483549 | 0.001697104 | GABRA1/GABRA2/GABRB2/GABRA5 | 4 |
| MF | GO:0031210 | phosphatidylcholine binding | 3/77 | 31/18410 | 0.000290645 | 0.002697786 | 0.0018435 | PCTP/PITPNM1/ABCA1 | 3 |
| MF | GO:0015103 | inorganic anion transmembrane transporter activity | 5/77 | 151/18410 | 0.000426835 | 0.003862859 | 0.002639639 | GABRA1/GABRA2/GABRB2/SLC12A5/GABRA5 | 5 |
| MF | GO:0005253 | anion channel activity | 4/77 | 87/18410 | 0.000484063 | 0.00417216 | 0.002850996 | GABRA1/GABRA2/GABRB2/GABRA5 | 4 |
| MF | GO:0042562 | hormone binding | 4/77 | 87/18410 | 0.000484063 | 0.00417216 | 0.002850996 | CCKBR/PTH2R/THRB/VIPR1 | 4 |
| MF | GO:0005501 | retinoid binding | 3/77 | 38/18410 | 0.000534117 | 0.004345885 | 0.002969709 | CYP26B1/IGF2R/RBP4 | 3 |
| MF | GO:0019840 | isoprenoid binding | 3/77 | 38/18410 | 0.000534117 | 0.004345885 | 0.002969709 | CYP26B1/IGF2R/RBP4 | 3 |
| MF | GO:0005319 | lipid transporter activity | 5/77 | 159/18410 | 0.000540234 | 0.004345885 | 0.002969709 | PCTP/PITPNM1/SLC27A2/RBP4/ABCA1 | 5 |
| MF | GO:0050997 | quaternary ammonium group binding | 3/77 | 39/18410 | 0.000576892 | 0.004539886 | 0.003102278 | PCTP/PITPNM1/ABCA1 | 3 |
| MF | GO:0001223 | transcription coactivator binding | 3/77 | 40/18410 | 0.000621797 | 0.00478916 | 0.003272616 | HIF1A/THRB/RELA | 3 |
| MF | GO:0001046 | core promoter sequence-specific DNA binding | 3/77 | 42/18410 | 0.000718169 | 0.005416191 | 0.00370109 | TP53/MYC/RELA | 3 |
| MF | GO:0016595 | glutamate binding | 2/77 | 10/18410 | 0.000760314 | 0.005617012 | 0.003838319 | GAD2/GRIN1 | 2 |
| MF | GO:0031406 | carboxylic acid binding | 5/77 | 173/18410 | 0.000791399 | 0.005729731 | 0.003915344 | CYP26B1/GAD2/GRIN1/IGF2R/FABP3 | 5 |
| MF | GO:0001221 | transcription coregulator binding | 4/77 | 108/18410 | 0.001090827 | 0.007720992 | 0.005276048 | HIF1A/THRB/MYC/RELA | 4 |
| MF | GO:0042301 | phosphate ion binding | 2/77 | 12/18410 | 0.001109093 | 0.007720992 | 0.005276048 | NCEH1/RELA | 2 |
| MF | GO:0016830 | carbon-carbon lyase activity | 3/77 | 50/18410 | 0.001197075 | 0.008176251 | 0.005587145 | DERA/GAD2/GOT1 | 3 |
| MF | GO:0008227 | G protein-coupled amine receptor activity | 3/77 | 51/18410 | 0.001268087 | 0.008500879 | 0.005808975 | ADRA2A/ADRB1/HTR2A | 3 |
| MF | GO:0017046 | peptide hormone binding | 3/77 | 52/18410 | 0.001341699 | 0.008726555 | 0.005963188 | CCKBR/PTH2R/VIPR1 | 3 |
| MF | GO:0016829 | lyase activity | 5/77 | 195/18410 | 0.001349964 | 0.008726555 | 0.005963188 | ADCY1/CA11/DERA/GAD2/GOT1 | 5 |
| MF | GO:0005262 | calcium channel activity | 4/77 | 119/18410 | 0.001561349 | 0.009915937 | 0.006775938 | CACNB2/GRIN1/RYR2/CDK5 | 4 |
| MF | GO:0030548 | acetylcholine receptor regulator activity | 2/77 | 15/18410 | 0.001750174 | 0.010738356 | 0.007337929 | LY6E/CDK5 | 2 |
| MF | GO:0099602 | neurotransmitter receptor regulator activity | 2/77 | 15/18410 | 0.001750174 | 0.010738356 | 0.007337929 | LY6E/CDK5 | 2 |
| MF | GO:0042826 | histone deacetylase binding | 4/77 | 126/18410 | 0.001925224 | 0.011615519 | 0.007937328 | HIF1A/TP53/RELA/MEF2C | 4 |
| MF | GO:0008509 | anion transmembrane transporter activity | 6/77 | 315/18410 | 0.002048424 | 0.012156222 | 0.008306811 | GABRA1/GABRA2/GABRB2/SLC12A5/SLC1A2/GABRA5 | 6 |
| MF | GO:0005548 | phospholipid transporter activity | 3/77 | 62/18410 | 0.002228355 | 0.013010716 | 0.008890719 | PCTP/PITPNM1/ABCA1 | 3 |
| MF | GO:0004713 | protein tyrosine kinase activity | 4/77 | 135/18410 | 0.002474375 | 0.013874562 | 0.009481018 | ABL1/CAMKK2/IGF2R/PTK2B | 4 |
| MF | GO:0015085 | calcium ion transmembrane transporter activity | 4/77 | 135/18410 | 0.002474375 | 0.013874562 | 0.009481018 | CACNB2/GRIN1/RYR2/CDK5 | 4 |
| MF | GO:0031690 | adrenergic receptor binding | 2/77 | 18/18410 | 0.002529616 | 0.013874562 | 0.009481018 | ADRA2A/ADRB1 | 2 |
| MF | GO:0034185 | apolipoprotein binding | 2/77 | 18/18410 | 0.002529616 | 0.013874562 | 0.009481018 | LCAT/ABCA1 | 2 |
| MF | GO:0046982 | protein heterodimerization activity | 6/77 | 332/18410 | 0.002662222 | 0.014182561 | 0.009691485 | ADRA2A/ADRB1/BCL2L2/HIF1A/TP53/MEF2C | 6 |
| MF | GO:0002039 | p53 binding | 3/77 | 66/18410 | 0.002664127 | 0.014182561 | 0.009691485 | HIF1A/TP53/CDK5 | 3 |
| MF | GO:0004970 | ionotropic glutamate receptor activity | 2/77 | 19/18410 | 0.002819576 | 0.014792557 | 0.010108319 | GRIN1/PTK2B | 2 |
| MF | GO:0001972 | retinoic acid binding | 2/77 | 20/18410 | 0.003124396 | 0.016157592 | 0.011041099 | CYP26B1/IGF2R | 2 |
| MF | GO:0001228 | DNA-binding transcription activator activity, RNA polymerase II-specific | 7/77 | 462/18410 | 0.003175279 | 0.016189451 | 0.01106287 | HIF1A/PRDM2/RORB/TP53/MYC/RELA/MEF2C | 7 |
| MF | GO:0001216 | DNA-binding transcription activator activity | 7/77 | 466/18410 | 0.003329729 | 0.01674114 | 0.01143986 | HIF1A/PRDM2/RORB/TP53/MYC/RELA/MEF2C | 7 |
| MF | GO:0008528 | G protein-coupled peptide receptor activity | 4/77 | 148/18410 | 0.003445042 | 0.017083635 | 0.0116739 | CCKBR/MCHR2/PTH2R/VIPR1 | 4 |
| MF | GO:0001653 | peptide receptor activity | 4/77 | 154/18410 | 0.003969751 | 0.019419593 | 0.013270149 | CCKBR/MCHR2/PTH2R/VIPR1 | 4 |
| MF | GO:0033293 | monocarboxylic acid binding | 3/77 | 81/18410 | 0.004749794 | 0.022925672 | 0.015665987 | CYP26B1/IGF2R/FABP3 | 3 |
| MF | GO:0051019 | mitogen-activated protein kinase binding | 2/77 | 26/18410 | 0.005258385 | 0.024721239 | 0.016892966 | ABL1/DUSP2 | 2 |
| MF | GO:0099604 | ligand-gated calcium channel activity | 2/77 | 26/18410 | 0.005258385 | 0.024721239 | 0.016892966 | GRIN1/RYR2 | 2 |
| MF | GO:0004683 | calmodulin-dependent protein kinase activity | 2/77 | 27/18410 | 0.005663742 | 0.026285573 | 0.017961936 | CAMKK2/PTK2B | 2 |
| MF | GO:0046875 | ephrin receptor binding | 2/77 | 28/18410 | 0.006082973 | 0.027873876 | 0.019047284 | ABL1/CDK5 | 2 |
| MF | GO:0004693 | cyclin-dependent protein serine/threonine kinase activity | 2/77 | 29/18410 | 0.006515957 | 0.029120695 | 0.019899283 | CDKL2/CDK5 | 2 |
| MF | GO:0097472 | cyclin-dependent protein kinase activity | 2/77 | 29/18410 | 0.006515957 | 0.029120695 | 0.019899283 | CDKL2/CDK5 | 2 |
| MF | GO:0033218 | amide binding | 6/77 | 402/18410 | 0.006725412 | 0.029690235 | 0.020288471 | CCKBR/GRIN1/PTH2R/RYR2/VIPR1/RELA | 6 |
| MF | GO:0016831 | carboxy-lyase activity | 2/77 | 34/18410 | 0.008883024 | 0.038742826 | 0.026474453 | GAD2/GOT1 | 2 |
| MF | GO:0097718 | disordered domain specific binding | 2/77 | 35/18410 | 0.009396047 | 0.040492488 | 0.027670063 | BCL2L2/TP53 | 2 |
| MF | GO:0000217 | DNA secondary structure binding | 2/77 | 36/18410 | 0.009922004 | 0.042256062 | 0.02887518 | ABL1/MEF2C | 2 |
| MF | GO:0042277 | peptide binding | 5/77 | 322/18410 | 0.011242542 | 0.047323259 | 0.03233779 | CCKBR/GRIN1/PTH2R/VIPR1/RELA | 5 |
| MF | GO:0051879 | Hsp90 protein binding | 2/77 | 43/18410 | 0.013956326 | 0.058071148 | 0.039682232 | HIF1A/CDK5 | 2 |
| MF | GO:0004715 | non-membrane spanning protein tyrosine kinase activity | 2/77 | 45/18410 | 0.015218995 | 0.06190198 | 0.042299986 | ABL1/PTK2B | 2 |
| MF | GO:0005245 | voltage-gated calcium channel activity | 2/77 | 45/18410 | 0.015218995 | 0.06190198 | 0.042299986 | CACNB2/CDK5 | 2 |
| MF | GO:0016298 | lipase activity | 3/77 | 130/18410 | 0.017220799 | 0.06926588 | 0.04733202 | LCAT/NCEH1/MGLL | 3 |
| MF | GO:0070888 | E-box binding | 2/77 | 49/18410 | 0.017885347 | 0.070374954 | 0.048089893 | HIF1A/MYC | 2 |
| MF | GO:0120013 | lipid transfer activity | 2/77 | 49/18410 | 0.017885347 | 0.070374954 | 0.048089893 | PITPNM1/ABCA1 | 2 |
| MF | GO:0004879 | nuclear receptor activity | 2/77 | 52/18410 | 0.020005154 | 0.076230166 | 0.052090983 | RORB/THRB | 2 |
| MF | GO:0098531 | ligand-activated transcription factor activity | 2/77 | 52/18410 | 0.020005154 | 0.076230166 | 0.052090983 | RORB/THRB | 2 |
| MF | GO:0140296 | general transcription initiation factor binding | 2/77 | 52/18410 | 0.020005154 | 0.076230166 | 0.052090983 | TP53/RELA | 2 |
| MF | GO:0008374 | O-acyltransferase activity | 2/77 | 53/18410 | 0.020734064 | 0.078184701 | 0.053426591 | LCAT/LPCAT3 | 2 |
| MF | GO:0030170 | pyridoxal phosphate binding | 2/77 | 55/18410 | 0.022224729 | 0.082941772 | 0.056677279 | GAD2/GOT1 | 2 |
| MF | GO:0016597 | amino acid binding | 2/77 | 56/18410 | 0.02298628 | 0.084050842 | 0.057435149 | GAD2/GRIN1 | 2 |
| MF | GO:0070279 | vitamin B6 binding | 2/77 | 56/18410 | 0.02298628 | 0.084050842 | 0.057435149 | GAD2/GOT1 | 2 |
| MF | GO:0019842 | vitamin binding | 3/77 | 148/18410 | 0.024164693 | 0.087476188 | 0.059775819 | GAD2/RBP4/GOT1 | 3 |
| MF | GO:0052689 | carboxylic ester hydrolase activity | 3/77 | 149/18410 | 0.024588714 | 0.088129846 | 0.060222489 | LCAT/NCEH1/MGLL | 3 |
| KEGG | hsa04727 | GABAergic synapse | 9/62 | 89/8164 | 1.8228E-08 | 3.99194E-06 | 2.99323E-06 | ADCY1/GABRA1/GABRA2/GABRB2/GAD2/GLS/KCNJ6/SLC12A5/GABRA5 | 9 |
| KEGG | hsa04080 | Neuroactive ligand-receptor interaction | 14/62 | 362/8164 | 3.69071E-07 | 4.04132E-05 | 3.03027E-05 | ADRA2A/ADRB1/CCKBR/GABRA1/GABRA2/GABRB2/GRIN1/GRM2/HTR2A/MCHR2/PTH2R/THRB/VIPR1/GABRA5 | 14 |
| KEGG | hsa04723 | Retrograde endocannabinoid signaling | 9/62 | 148/8164 | 1.48354E-06 | 0.000108299 | 8.12045E-05 | ADCY1/GABRA1/GABRA2/GABRB2/KCNJ3/KCNJ6/MAPK9/MGLL/GABRA5 | 9 |
| KEGG | hsa05032 | Morphine addiction | 7/62 | 91/8164 | 5.06066E-06 | 0.000277071 | 0.000207753 | ADCY1/GABRA1/GABRA2/GABRB2/KCNJ3/KCNJ6/GABRA5 | 7 |
| KEGG | hsa05033 | Nicotine addiction | 5/62 | 40/8164 | 1.15013E-05 | 0.000503758 | 0.000377728 | GABRA1/GABRA2/GABRB2/GRIN1/GABRA5 | 5 |
| KEGG | hsa04921 | Oxytocin signaling pathway | 7/62 | 154/8164 | 0.000152813 | 0.005577672 | 0.004182249 | ADCY1/CACNB2/CAMKK2/KCNJ3/KCNJ6/RYR2/MEF2C | 7 |
| KEGG | hsa04724 | Glutamatergic synapse | 6/62 | 114/8164 | 0.000211335 | 0.006611763 | 0.004957631 | ADCY1/GLS/GRIN1/GRM2/KCNJ3/SLC1A2 | 6 |
| KEGG | hsa05030 | Cocaine addiction | 4/62 | 49/8164 | 0.000494786 | 0.013544773 | 0.010156138 | GRIN1/GRM2/RELA/CDK5 | 4 |
| KEGG | hsa04713 | Circadian entrainment | 5/62 | 97/8164 | 0.000808775 | 0.019680199 | 0.014756602 | ADCY1/GRIN1/KCNJ3/KCNJ6/RYR2 | 5 |
| KEGG | hsa05161 | Hepatitis B | 6/62 | 162/8164 | 0.00136615 | 0.029918689 | 0.022433624 | MAPK9/PTK2B/TP53/MYC/RELA/CCNA1 | 6 |
| KEGG | hsa05230 | Central carbon metabolism in cancer | 4/62 | 70/8164 | 0.001901386 | 0.03632215 | 0.027235066 | GLS/HIF1A/TP53/MYC | 4 |
| KEGG | hsa04137 | Mitophagy - animal | 4/62 | 72/8164 | 0.002109472 | 0.03632215 | 0.027235066 | HIF1A/MAPK9/TP53/RELA | 4 |
| KEGG | hsa04020 | Calcium signaling pathway | 7/62 | 240/8164 | 0.002156109 | 0.03632215 | 0.027235066 | ADCY1/ADRB1/CCKBR/GRIN1/HTR2A/PTK2B/RYR2 | 7 |
| KEGG | hsa05220 | Chronic myeloid leukemia | 4/62 | 76/8164 | 0.002571951 | 0.039371092 | 0.029521223 | ABL1/TP53/MYC/RELA | 4 |
| KEGG | hsa00250 | Alanine, aspartate and glutamate metabolism | 3/62 | 37/8164 | 0.00269665 | 0.039371092 | 0.029521223 | GAD2/GLS/GOT1 | 3 |
| KEGG | hsa05418 | Fluid shear stress and atherosclerosis | 5/62 | 139/8164 | 0.003957093 | 0.054162711 | 0.040612271 | GSTO2/MAPK9/TP53/RELA/MEF2C | 5 |
| KEGG | hsa04211 | Longevity regulating pathway | 4/62 | 89/8164 | 0.004548951 | 0.058601186 | 0.043940327 | ADCY1/CAMKK2/TP53/RELA | 4 |
| KEGG | hsa05414 | Dilated cardiomyopathy | 4/62 | 96/8164 | 0.005950129 | 0.060967513 | 0.045714645 | ADCY1/ADRB1/CACNB2/RYR2 | 4 |
| KEGG | hsa04024 | cAMP signaling pathway | 6/62 | 221/8164 | 0.006389782 | 0.060967513 | 0.045714645 | ADCY1/ADRB1/GRIN1/MAPK9/RYR2/RELA | 6 |
| KEGG | hsa01522 | Endocrine resistance | 4/62 | 98/8164 | 0.006397813 | 0.060967513 | 0.045714645 | ADCY1/MAPK9/TP53/JAG2 | 4 |
| KEGG | hsa04218 | Cellular senescence | 5/62 | 156/8164 | 0.006438728 | 0.060967513 | 0.045714645 | TP53/TRAF3IP2/MYC/RELA/CCNA1 | 5 |
| KEGG | hsa05166 | Human T-cell leukemia virus 1 infection | 6/62 | 222/8164 | 0.006528953 | 0.060967513 | 0.045714645 | ADCY1/MAPK9/TP53/MYC/RELA/CCNA1 | 6 |
| KEGG | hsa04010 | MAPK signaling pathway | 7/62 | 294/8164 | 0.006593494 | 0.060967513 | 0.045714645 | CACNB2/DUSP2/MAPK9/TP53/MYC/RELA/MEF2C | 7 |
| KEGG | hsa04979 | Cholesterol metabolism | 3/62 | 51/8164 | 0.006703543 | 0.060967513 | 0.045714645 | LCAT/NCEH1/ABCA1 | 3 |
| KEGG | hsa05163 | Human cytomegalovirus infection | 6/62 | 225/8164 | 0.006959762 | 0.060967513 | 0.045714645 | ADCY1/IL10RB/PTK2B/TP53/MYC/RELA | 6 |
| KEGG | hsa04914 | Progesterone-mediated oocyte maturation | 4/62 | 102/8164 | 0.00735931 | 0.061988037 | 0.046479855 | ADCY1/MAPK9/STK10/CCNA1 | 4 |
| KEGG | hsa04923 | Regulation of lipolysis in adipocytes | 3/62 | 57/8164 | 0.00912069 | 0.073978932 | 0.055470864 | ADCY1/ADRB1/MGLL | 3 |
| KEGG | hsa04726 | Serotonergic synapse | 4/62 | 115/8164 | 0.011130468 | 0.087056158 | 0.065276427 | GABRB2/HTR2A/KCNJ3/KCNJ6 | 4 |
| KEGG | hsa00220 | Arginine biosynthesis | 2/62 | 22/8164 | 0.011888405 | 0.088376181 | 0.066266207 | GLS/GOT1 | 2 |
| KEGG | hsa04722 | Neurotrophin signaling pathway | 4/62 | 119/8164 | 0.01250141 | 0.088376181 | 0.066266207 | ABL1/MAPK9/TP53/RELA | 4 |
| KEGG | hsa04929 | GnRH secretion | 3/62 | 64/8164 | 0.01250987 | 0.088376181 | 0.066266207 | KCNJ3/KCNJ6/KCNN1 | 3 |
| KEGG | hsa04919 | Thyroid hormone signaling pathway | 4/62 | 121/8164 | 0.013225754 | 0.090513756 | 0.067869003 | HIF1A/THRB/TP53/MYC | 4 |
| KEGG | hsa05221 | Acute myeloid leukemia | 3/62 | 67/8164 | 0.01415514 | 0.091737433 | 0.06878654 | MYC/RELA/CCNA1 | 3 |
| KEGG | hsa04110 | Cell cycle | 4/62 | 126/8164 | 0.015152597 | 0.091737433 | 0.06878654 | ABL1/TP53/MYC/CCNA1 | 4 |
| KEGG | hsa05202 | Transcriptional misregulation in cancer | 5/62 | 193/8164 | 0.01527401 | 0.091737433 | 0.06878654 | TP53/MYC/RELA/CCNA1/MEF2C | 5 |
| KEGG | hsa04920 | Adipocytokine signaling pathway | 3/62 | 69/8164 | 0.015317203 | 0.091737433 | 0.06878654 | CAMKK2/MAPK9/RELA | 3 |
| KEGG | hsa05167 | Kaposi sarcoma-associated herpesvirus infection | 5/62 | 194/8164 | 0.01558835 | 0.091737433 | 0.06878654 | HIF1A/MAPK9/TP53/MYC/RELA | 5 |
| KEGG | hsa05120 | Epithelial cell signaling in Helicobacter pylori infection | 3/62 | 70/8164 | 0.015917911 | 0.091737433 | 0.06878654 | ATP6V1B2/MAPK9/RELA | 3 |

**Table S9. Expanded benchmarking of top-performing machine-learning workflows.**

| **Workflow** | **Retained genes**  **(n)** | **AUC**  **(Train)** | **AUC**  **(GSE00014)** | **AUC**  **(GSE10878)** | **AUC**  **(GSE50161)** | **Mean AUC** | **PR-AUC / AP**  **(pooled external)** | **F1-score**  **(pooled external)** | **MCC**  **(pooled external)** | **Precision**  **(pooled external)** | **Recall**  **(pooled external)** |
| --- | --- | --- | --- | --- | --- | --- | --- | --- | --- | --- | --- |
| LDA | 77.000 | 0.998 | 0.943 | 1.000 | 0.986 | 0.982 | 0.927 | 0.592 | 0.213 | 0.423 | 0.984 |
| Stepglm[both]+plsRglm | 12.000 | 0.995 | 0.947 | 1.000 | 0.984 | 0.981 | 0.919 | 0.588 | 0.206 | 0.417 | 0.997 |
| glmBoost+Ridge | 12.000 | 0.985 | 0.941 | 1.000 | 0.977 | 0.976 | 0.944 | 0.617 | 0.288 | 0.452 | 0.969 |
| plsRglm | 77.000 | 0.992 | 0.958 | 1.000 | 0.946 | 0.974 | 0.949 | 0.604 | 0.258 | 0.436 | 0.987 |
| glmBoost+plsRglm | 12.000 | 0.990 | 0.963 | 1.000 | 0.930 | 0.971 | 0.956 | 0.584 | 0.186 | 0.414 | 0.991 |
| Stepglm[both]+LDA | 12.000 | 0.988 | 0.909 | 1.000 | 0.984 | 0.970 | 0.911 | 0.599 | 0.230 | 0.434 | 0.964 |
| glmBoost | 12.000 | 0.985 | 0.925 | 1.000 | 0.968 | 0.970 | 0.912 | 0.666 | 0.410 | 0.510 | 0.963 |
| Stepglm[both]+NaiveBayes | 12.000 | 0.980 | 0.919 | 1.000 | 0.977 | 0.969 | 0.857 | 0.698 | 0.474 | 0.552 | 0.949 |
| Stepglm[backward]+RF | 4.000 | 0.999 | 0.913 | 1.000 | 0.964 | 0.969 | 0.883 | 0.580 | 0.169 | 0.410 | 0.992 |
| Stepglm[both]+RF | 4.000 | 0.999 | 0.911 | 1.000 | 0.962 | 0.968 | 0.870 | 0.579 | 0.162 | 0.409 | 0.992 |

**Table S10. Molecular docking.**

| **Ligand** | **Protein** | **PDB ID** | **Amino saci residue count** | **Molecular weight/Da** | **Binding energy /Kcal/moL** | **Bound amino acid** | **Types of Non-Covalent Interactions** |
| --- | --- | --- | --- | --- | --- | --- | --- |
| DEHP | ABCA1 | 7TC0 | 2,270 | 258,210 | -5.75 | TRP-278、ARG-276 | Hydrophobic Interaction、Hydrogen Bond |
|  | CCKBR | 7XOW | 447 | 166,360 | -5.40 | PHE-278 | Hydrophobic Interaction、Hydrogen Bond |
|  | CDKL2 | 4AAA | 331 | 39,190 | -7.19 | PHE-145 | Hydrophobic Interaction、Hydrogen Bond |
|  | GABRA5 | 8BHG | 350 | 210,360 | -6.44 | None | Hydrophobic Interaction、Salt Bridge |
|  | GSTO2 | 3Q18 | 239 | 55,810 | -5.70 | TRP-181 | Hydrophobic Interaction、Hydrogen Bond、π-Cation Interaction |
|  | HIF1A | 4H6J | 113 | 26,690 | -5.09 | LYS-465 | Hydrophobic Interaction、Hydrogen Bond |
|  | MCHR2 | 8WST | 246 | 128,820 | -6.20 | PHE-234、LEU-192 | Hydrophobic Interaction、Hydrogen Bond、Salt Bridge |
|  | MYC | 6G6K | 94 | 42,890 | -5.04 | ARG-214 | Hydrophobic Interaction、Salt Bridge |
|  | RELA | 6NV2 | 236 | 28,500 | -7.03 | ARG-82 | Hydrogen Bond |
|  | STK17B | 6ZJF | 327 | 37,790 | -5.89 | None | Hydrophobic Interaction |
|  | TRAF3IP2 | AF-O43734-F1 | 574 | 64,666 | -5.07 | ASN-434 | Hydrophobic Interaction、Hydrogen Bond、Salt Bridge |
|  | TYRP1 | 5M8S | 446 | 212,930 | -5.73 | TRP-117、ARG-125 | Hydrophobic Interaction、Salt Bridge |
