# Supplementary material for: Machine learning-integrated network toxicology uncovers glioma targets of DEHP
Source: Front Toxicol. 2026 May 8;8:1771011. doi: 10.3389/ftox.2026.1771011 (PMC13193683; doi:10.3389/ftox.2026.1771011)
Supplement: Supplementary file 9 [file DataSheet1.docx]

| Project | Databse | Data links |
| --- | --- | --- |
| Structure of DEHP | Pubchem | https://pubchem.ncbi.nlm.nih.gov/#query=%22DIETHYL%20PHTHALATE%22 |
| Biological targets of DEHP | CHEMBL | https://www.ebi.ac.uk/chembl/ |
|  | CTD | https://ctdbase.org/ |
|  | Swiss Target Prediction | http://www.swisstargetprediction.ch/ |
|  | Pharmmapper | https://www.lilab-ecust.cn/pharmmapper/index.html |
| Glioma | GSE16011 | https://ftp.ncbi.nlm.nih.gov/geo/series/GSE16nnn/GSE16011/matrix/ |
|  | GSE4290 | https://ftp.ncbi.nlm.nih.gov/geo/series/GSE4nnn/GSE4290/matrix/ |
|  | GSE10878 | https://ftp.ncbi.nlm.nih.gov/geo/series/GSE10nnn/GSE10878/matrix/ |
|  | GSE50161 | https://ftp.ncbi.nlm.nih.gov/geo/series/GSE50nnn/GSE50161/matrix/ |
|  | TCGA | https://www.cancer.gov/ccg/research/genome-sequencing/tcga |
|  | GTEx | https://www.gtexportal.org |

**Table S1. DEHP and glioma-associated proteins, as well as structural data for DEHP**

**Table S2. DEHP targets from CHEMBL databse.**

| **ChEMBL ID** | **Name** | **UniProt Accessions** | **Type** | **Organism** | **Compounds** | **Activities** | **Tax ID** | **Species Group Flag** |
| --- | --- | --- | --- | --- | --- | --- | --- | --- |
| CHEMBL3580484 | Interleukin-12 subunit beta | P29460 | SINGLE PROTEIN | Homo sapiens |  |  | 9606 | FALSE |
| CHEMBL3542438 | Uridine-cytidine kinase | Q9BZX2|Q9HA47 | PROTEIN FAMILY | Homo sapiens |  |  | 9606 | FALSE |
| CHEMBL3542433 | Phenol sulfotransferase | P50225|P50226|P0DMM9 | PROTEIN FAMILY | Homo sapiens |  |  | 9606 | FALSE |
| CHEMBL2363072 | Retinoid X receptor gamma/retinoic acid receptor alpha | P48443|P10276 | PROTEIN COMPLEX | Homo sapiens |  |  | 9606 | FALSE |
| CHEMBL5215 | Solute carrier family 2, facilitated glucose transporter member 3 | P11169 | SINGLE PROTEIN | Homo sapiens | 460 | 465 | 9606 | FALSE |
| CHEMBL3885612 | Nuclear receptor subfamily 2 group C member 2/TGF-beta-activated kinase 1 and MAP3K7-binding protein 1 | Q15750|P49116 | CHIMERIC PROTEIN | Homo sapiens | 49 | 49 | 9606 | FALSE |
| CHEMBL1075105 | Protein tyrosine phosphatase type IVA 2 | Q12974 | SINGLE PROTEIN | Homo sapiens | 32 | 37 | 9606 | FALSE |
| CHEMBL4630758 | 5-hydroxytryptamine receptor 2A/Metabotropic glutamate receptor 2 | P28223|Q14416 | PROTEIN COMPLEX | Homo sapiens | 17 | 187 | 9606 | FALSE |
| CHEMBL4106152 | CDK2/Cyclin O | P24941|P22674 | PROTEIN COMPLEX | Homo sapiens | 21 | 25 | 9606 | FALSE |
| CHEMBL3430879 | Retinoic acid receptor RXR-alpha/oxysterols receptor LXR-alpha | P19793|Q13133 | PROTEIN COMPLEX | Homo sapiens | 91 | 132 | 9606 | FALSE |
| CHEMBL5641 | V-type proton ATPase subunit B, brain isoform | P21281 | SINGLE PROTEIN | Homo sapiens | 2 | 3 | 9606 | FALSE |
| CHEMBL4523985 | UDP-glucuronosyltransferases (UGTs) | P22310|P16662|O75795|P36537|P54855|Q9BY64|P06133|O60656|Q9HAW8|P0DTE4|P22309|P19224|Q9HAW7|Q9HAW9|P35503|P35504|Q6UWM9|O75310|P0DTE5 | PROTEIN FAMILY | Homo sapiens | 30 | 68 | 9606 | FALSE |
| CHEMBL5169269 | BCL2A1/BAK1 | Q16611|Q16548 | PROTEIN-PROTEIN INTERACTION | Homo sapiens | 4 | 4 | 9606 | FALSE |
| CHEMBL4523398 | Prolyl 3-hydroxylase OGFOD1 | Q8N543 | SINGLE PROTEIN | Homo sapiens | 5 | 5 | 9606 | FALSE |
| CHEMBL3309108 | Taste receptor type 2 member 41 | P59536 | SINGLE PROTEIN | Homo sapiens | 10 | 57 | 9606 | FALSE |
| CHEMBL613881 | Huh-5-2 |  | CELL-LINE | Homo sapiens | 377 | 386 | 9606 | FALSE |
| CHEMBL3213 | Calcium-independent phospholipase A2 | O60733 | SINGLE PROTEIN | Homo sapiens | 183 | 359 | 9606 | FALSE |
| CHEMBL6174 | Cytochrome c oxidase subunit 2 | P00403 | SINGLE PROTEIN | Homo sapiens | 171 | 250 | 9606 | FALSE |
| CHEMBL4106171 | Superoxide dismutase 1/2 | P00441|P04179 | PROTEIN FAMILY | Homo sapiens | 13 | 13 | 9606 | FALSE |
| CHEMBL2457 | Arachidonate 15-lipoxygenase, type II | O15296 | SINGLE PROTEIN | Homo sapiens | 7035 | 7244 | 9606 | FALSE |
| CHEMBL3200 | Sialidase 2 | Q9Y3R4 | SINGLE PROTEIN | Homo sapiens | 233 | 382 | 9606 | FALSE |
| CHEMBL5501 | Histone acetyltransferase GCN5 | Q92830 | SINGLE PROTEIN | Homo sapiens | 13966 | 14293 | 9606 | FALSE |
| CHEMBL2535 | Glucose transporter | P11166 | SINGLE PROTEIN | Homo sapiens | 14043 | 14777 | 9606 | FALSE |
| CHEMBL4295812 | Peroxiredoxin-4 | Q13162 | SINGLE PROTEIN | Homo sapiens | 1 | 2 | 9606 | FALSE |
| CHEMBL4523417 | Tribbles homolog 2 | Q92519 | SINGLE PROTEIN | Homo sapiens | 16 | 18 | 9606 | FALSE |
| CHEMBL5794 | Serine/threonine-protein kinase Sgk2 | Q9HBY8 | SINGLE PROTEIN | Homo sapiens | 1506 | 2096 | 9606 | FALSE |
| CHEMBL5058622 | ES-2 |  | CELL-LINE | Homo sapiens | 134 | 147 | 9606 | FALSE |
| CHEMBL3774298 | Histone acetyltransferase KAT6A | Q92794 | SINGLE PROTEIN | Homo sapiens | 269 | 323 | 9606 | FALSE |
| CHEMBL2109230 | Neuronal acetylcholine receptor; alpha2/beta4 | P30926|Q15822 | PROTEIN COMPLEX | Homo sapiens | 33 | 47 | 9606 | FALSE |
| CHEMBL2111371 | PPAR delta/gamma | P37231|Q03181 | SELECTIVITY GROUP | Homo sapiens | 113 | 120 | 9606 | FALSE |
| CHEMBL5169147 | ADP-ribosyl cyclase/cyclic ADP-ribose hydrolase 2 | Q10588 | SINGLE PROTEIN | Homo sapiens | 1 | 1 | 9606 | FALSE |
| CHEMBL5169085 | CRBN/CDK13 | Q14004|Q96SW2 | PROTEIN-PROTEIN INTERACTION | Homo sapiens | 27 | 50 | 9606 | FALSE |
| CHEMBL2176774 | Bromodomain-containing protein 1 | O95696 | SINGLE PROTEIN | Homo sapiens | 167 | 263 | 9606 | FALSE |
| CHEMBL1255132 | Protein Wnt-2 | P09544 | SINGLE PROTEIN | Homo sapiens | 13 | 15 | 9606 | FALSE |
| CHEMBL3575 | RNase L | Q05823 | SINGLE PROTEIN | Homo sapiens | 152 | 245 | 9606 | FALSE |
| CHEMBL209 | Trypsin I | P07477 | SINGLE PROTEIN | Homo sapiens | 2042 | 2308 | 9606 | FALSE |
| CHEMBL3784 | Histone acetyltransferase p300 | Q09472 | SINGLE PROTEIN | Homo sapiens | 909 | 1411 | 9606 | FALSE |
| CHEMBL4295814 | Eukaryotic translation initiation factor 3 subunit I | Q13347 | SINGLE PROTEIN | Homo sapiens | 1 | 1 | 9606 | FALSE |
| CHEMBL5482997 | VEGFR2-VEGA | P15692|P35968 | PROTEIN COMPLEX | Homo sapiens | 2 | 2 | 9606 | FALSE |
| CHEMBL2251 | PI4-kinase type II | Q9BTU6 | SINGLE PROTEIN | Homo sapiens | 62 | 65 | 9606 | FALSE |
| CHEMBL3430907 | Aurora kinase B/Inner centromere protein | Q96GD4|Q9NQS7 | PROTEIN COMPLEX | Homo sapiens | 214 | 233 | 9606 | FALSE |
| CHEMBL2097167 | Adenylate cyclase | Q8NFM4|O60503|Q08828|P40145|O60266|O95622|O43306|Q08462|P51828 | PROTEIN FAMILY | Homo sapiens | 49 | 52 | 9606 | FALSE |
| CHEMBL4851 | Serine/threonine-protein kinase 38-like | Q9Y2H1 | SINGLE PROTEIN | Homo sapiens | 434 | 581 | 9606 | FALSE |
| CHEMBL5037 | Caspase-10 | Q92851 | SINGLE PROTEIN | Homo sapiens | 22 | 24 | 9606 | FALSE |
| CHEMBL4105836 | Parathyroid hormone 2 receptor | P49190 | SINGLE PROTEIN | Homo sapiens | 2 | 3 | 9606 | FALSE |
| CHEMBL5282 | Cytochrome P450 2A6 | P11509 | SINGLE PROTEIN | Homo sapiens | 1356 | 2864 | 9606 | FALSE |
| CHEMBL4630741 | VHL/Histone deacetylase 3 | O15379|P40337 | PROTEIN-PROTEIN INTERACTION | Homo sapiens | 33 | 81 | 9606 | FALSE |
| CHEMBL3885520 | Axin-2/Catenin beta-1 | P35222|Q9Y2T1 | PROTEIN-PROTEIN INTERACTION | Homo sapiens | 1 | 1 | 9606 | FALSE |
| CHEMBL4105963 | Probable bifunctional methylenetetrahydrofolate dehydrogenase/cyclohydrolase 2 | Q9H903 | SINGLE PROTEIN | Homo sapiens | 1 | 1 | 9606 | FALSE |
| CHEMBL4105986 | Transcription factor Dp-2 | Q14188 | SINGLE PROTEIN | Homo sapiens | 1 | 2 | 9606 | FALSE |
| CHEMBL3885639 | Small ubiquitin-related modifier 2/3 (SUMO 2/3) | P61956|P55854 | PROTEIN FAMILY | Homo sapiens | 3 | 3 | 9606 | FALSE |
| CHEMBL2111413 | GABA A receptor alpha-2/beta-2/gamma-2 | P47870|P18507|P47869 | PROTEIN COMPLEX | Homo sapiens | 135 | 194 | 9606 | FALSE |
| CHEMBL3351212 | LanC-like protein 2 | Q9NS86 | SINGLE PROTEIN | Homo sapiens | 13 | 13 | 9606 | FALSE |
| CHEMBL4742270 | Cereblon/Laminin subunit beta-1 | P07942|Q96SW2 | PROTEIN-PROTEIN INTERACTION | Homo sapiens | 1 | 1 | 9606 | FALSE |
| CHEMBL3125 | Ribosomal protein S6 kinase alpha 4 | O75676 | SINGLE PROTEIN | Homo sapiens | 1168 | 2183 | 9606 | FALSE |
| CHEMBL2163167 | S-methylmethionine--homocysteine S-methyltransferase BHMT2 | Q9H2M3 | SINGLE PROTEIN | Homo sapiens | 9 | 11 | 9606 | FALSE |
| CHEMBL4523619 | MAPK3/MAPK7 | P27361|Q13164 | PROTEIN FAMILY | Homo sapiens | 1 | 1 | 9606 | FALSE |
| CHEMBL5291688 | VHL/FGFR1 | P11362|P40337 | PROTEIN-PROTEIN INTERACTION | Homo sapiens | 6 | 21 | 9606 | FALSE |
| CHEMBL5330 | Mitogen-activated protein kinase kinase kinase kinase 2 | Q12851 | SINGLE PROTEIN | Homo sapiens | 2001 | 2743 | 9606 | FALSE |
| CHEMBL4296460 | LNCaP C4-2 |  | CELL-LINE | Homo sapiens | 127 | 192 | 9606 | FALSE |
| CHEMBL4523423 | Mitochondrial amidoxime reducing component 2 | Q969Z3 | SINGLE PROTEIN | Homo sapiens | 21 | 38 | 9606 | FALSE |
| CHEMBL3542432 | Flavin-containing monooxygenase | P31513|Q99518|P31512|P49326|Q01740 | PROTEIN FAMILY | Homo sapiens | 1 | 1 | 9606 | FALSE |
| CHEMBL2189114 | Histone-lysine N-methyltransferase MLL2 | O14686 | SINGLE PROTEIN | Homo sapiens | 16 | 17 | 9606 | FALSE |
| CHEMBL1795177 | Histone-lysine N-methyltransferase SUV39H2 | Q9H5I1 | SINGLE PROTEIN | Homo sapiens | 524 | 537 | 9606 | FALSE |
| CHEMBL3885552 | Cyclin-dependent kinase 2/G1/S-specific cyclin-D1 | P24385|P24941 | PROTEIN COMPLEX | Homo sapiens | 9 | 9 | 9606 | FALSE |
| CHEMBL4403 | Ryanodine receptor 2 | Q92736 | SINGLE PROTEIN | Homo sapiens | 68 | 84 | 9606 | FALSE |
| CHEMBL5465344 | RNA-binding protein Musashi homolog 2 | Q96DH6 | SINGLE PROTEIN | Homo sapiens | 1 | 2 | 9606 | FALSE |
| CHEMBL4295963 | ADP-ribose glycohydrolase ARH3 | Q9NX46 | SINGLE PROTEIN | Homo sapiens | 3 | 3 | 9606 | FALSE |
| CHEMBL2096974 | Adrenergic receptor beta; ADRB1 & ADRB2 | P08588|P07550 | SELECTIVITY GROUP | Homo sapiens | 189 | 240 | 9606 | FALSE |
| CHEMBL4804182 | Neuronal acetylcholine receptor | P17787|P30926|P32297|P43681|Q15822|Q9UGM1|Q15825|P30532|P36544|Q9GZZ6|Q05901 | PROTEIN COMPLEX GROUP | Homo sapiens | 3 | 5 | 9606 | FALSE |
| CHEMBL612409 | Anti-estrogen binding site (AEBS) | Q15125|Q9UBM7 | PROTEIN COMPLEX | Homo sapiens | 68 | 124 | 9606 | FALSE |
| CHEMBL2990 | Alcohol dehydrogenase class II | P08319 | SINGLE PROTEIN | Homo sapiens | 23 | 23 | 9606 | FALSE |
| CHEMBL3351200 | Kinesin-like protein KIFC1 | Q9BW19 | SINGLE PROTEIN | Homo sapiens | 84 | 110 | 9606 | FALSE |
| CHEMBL3108645 | Histone-lysine N-methyltransferase NSD2 | O96028 | SINGLE PROTEIN | Homo sapiens | 813 | 932 | 9606 | FALSE |
| CHEMBL2163175 | Gamma-butyrobetaine dioxygenase | O75936 | SINGLE PROTEIN | Homo sapiens | 75 | 162 | 9606 | FALSE |
| CHEMBL4523622 | NTRK1/NTRK2 | P04629|Q16620 | PROTEIN FAMILY | Homo sapiens | 5 | 5 | 9606 | FALSE |
| CHEMBL2146343 | Dual specificity protein phosphatase 4 | Q13115 | SINGLE PROTEIN | Homo sapiens | 7 | 7 | 9606 | FALSE |
| CHEMBL3885516 | Apoptosis regulator Bcl-2/Bcl-2 homologous antagonist/killer | P10415|Q16611 | PROTEIN-PROTEIN INTERACTION | Homo sapiens | 28 | 28 | 9606 | FALSE |
| CHEMBL3137283 | Bcl-xL/BAK | Q07817|Q16611 | PROTEIN-PROTEIN INTERACTION | Homo sapiens | 68 | 77 | 9606 | FALSE |
| CHEMBL4270 | Matrix metalloproteinase 10 | P09238 | SINGLE PROTEIN | Homo sapiens | 309 | 370 | 9606 | FALSE |
| CHEMBL2963 | Matrix metalloproteinase 15 | P51511 | SINGLE PROTEIN | Homo sapiens | 44 | 50 | 9606 | FALSE |
| CHEMBL1741207 | Sentrin-specific protease 8 | Q96LD8 | SINGLE PROTEIN | Homo sapiens | 1263 | 1687 | 9606 | FALSE |
| CHEMBL6122 | Group XIIA secretory phospholipase A2 | Q9BZM1 | SINGLE PROTEIN | Homo sapiens | 16 | 16 | 9606 | FALSE |
| CHEMBL5367 | DNA polymerase lambda | Q9UGP5 | SINGLE PROTEIN | Homo sapiens | 104 | 184 | 9606 | FALSE |
| CHEMBL4523663 | Serine/threonine-protein kinase 3/4 | Q13043|Q13188 | PROTEIN FAMILY | Homo sapiens | 1 | 3 | 9606 | FALSE |
| CHEMBL3883288 | Bcl-xL/Bcl-2-binding component 3 | Q07817|Q9BXH1 | PROTEIN-PROTEIN INTERACTION | Homo sapiens | 27 | 27 | 9606 | FALSE |
| CHEMBL3038517 | CDK2/CDK4 | P11802|P24941 | PROTEIN FAMILY | Homo sapiens | 6 | 6 | 9606 | FALSE |
| CHEMBL1743185 | Microsomal glutathione S-transferase 2 | Q99735 | SINGLE PROTEIN | Homo sapiens | 3 | 3 | 9606 | FALSE |
| CHEMBL2933 | Potassium-transporting ATPase alpha chain 2 | P54707 | SINGLE PROTEIN | Homo sapiens | 82 | 83 | 9606 | FALSE |
| CHEMBL5291941 | BRD2/BRD9 | P25440|Q9H8M2 | SELECTIVITY GROUP | Homo sapiens | 4 | 12 | 9606 | FALSE |
| CHEMBL4523631 | CDK9/Cyclin-T2 | P50750|O60583 | PROTEIN COMPLEX | Homo sapiens | 6 | 7 | 9606 | FALSE |
| CHEMBL4105740 | Choline/ethanolaminephosphotransferase 1 | Q9Y6K0 | SINGLE PROTEIN | Homo sapiens | 49 | 65 | 9606 | FALSE |
| CHEMBL4523984 | MMP-1/MMP-2 | P08253|P03956 | SELECTIVITY GROUP | Homo sapiens | 87 | 87 | 9606 | FALSE |
| CHEMBL2111375 | Vascular endothelial growth factor receptor 2 and tyrosine-protein kinase TIE-2 (KDR and TIE2) | P35968|Q02763 | SELECTIVITY GROUP | Homo sapiens | 14 | 14 | 9606 | FALSE |
| CHEMBL5465251 | MDM2-HDAC3 | Q00987|O15379 | PROTEIN-PROTEIN INTERACTION | Homo sapiens | 4 | 20 | 9606 | FALSE |
| CHEMBL5291977 | GTPase KRas/RAF1 | P04049|P01116 | PROTEIN-PROTEIN INTERACTION | Homo sapiens | 4 | 4 | 9606 | FALSE |
| CHEMBL2169728 | Pancreatic lipase-related protein 2 | P54317 | SINGLE PROTEIN | Homo sapiens | 4 | 17 | 9606 | FALSE |
| CHEMBL4106183 | Glutaminase 1/2 | O94925|Q9UI32 | PROTEIN FAMILY | Homo sapiens | 3 | 5 | 9606 | FALSE |
| CHEMBL3832642 | T1R2/T1R3 | Q8TE23|Q7RTX0 | PROTEIN COMPLEX | Homo sapiens | 179 | 282 | 9606 | FALSE |
| CHEMBL5416 | PITSLRE serine/threonine-protein kinase CDC2L2 | Q9UQ88 | SINGLE PROTEIN | Homo sapiens | 278 | 379 | 9606 | FALSE |
| CHEMBL4523303 | Transcription factor NF-E2 45 kDa subunit | Q16621 | SINGLE PROTEIN | Homo sapiens | 2 | 2 | 9606 | FALSE |
| CHEMBL4888454 | CDK2/Cyclin C | P24941|P24863 | PROTEIN COMPLEX | Homo sapiens | 1 | 1 | 9606 | FALSE |
| CHEMBL5812 | Dynamin-2 | P50570 | SINGLE PROTEIN | Homo sapiens | 36 | 62 | 9606 | FALSE |
| CHEMBL3038454 | AMPK alpha1/alpha2 | P54646|Q13131 | PROTEIN COMPLEX | Homo sapiens | 14 | 14 | 9606 | FALSE |
| CHEMBL4014 | Tyrosine-protein kinase ABL2 | P42684 | SINGLE PROTEIN | Homo sapiens | 1141 | 1901 | 9606 | FALSE |
| CHEMBL3736 | Angiotensin-converting enzyme 2 | Q9BYF1 | SINGLE PROTEIN | Homo sapiens | 199 | 289 | 9606 | FALSE |
| CHEMBL3885504 | 5'-AMP-activated protein kinase catalytic subunit alpha-2/beta-1/gamma-2 | P54646|Q9UGJ0|Q9Y478 | PROTEIN COMPLEX | Homo sapiens | 5 | 7 | 9606 | FALSE |
| CHEMBL2111426 | ETS-related transcription factor Elf-3/Vitamin D3 receptor-interacting protein (ESX/MED23) | Q9ULK4|P78545 | PROTEIN-PROTEIN INTERACTION | Homo sapiens | 8 | 14 | 9606 | FALSE |
| CHEMBL3038500 | MAST1/MAST2 | Q9Y2H9|Q6P0Q8 | PROTEIN FAMILY | Homo sapiens | 6 | 6 | 9606 | FALSE |
| CHEMBL3309107 | Taste receptor type 2 member 4 | Q9NYW5 | SINGLE PROTEIN | Homo sapiens | 11 | 14 | 9606 | FALSE |
| CHEMBL3309109 | Taste receptor type 2 member 60 | P59551 | SINGLE PROTEIN | Homo sapiens | 7 | 8 | 9606 | FALSE |
| CHEMBL3832633 | Ribosomal protein S6 kinase | P51812|Q9UBS0|O75676|Q15349|O75582|P23443|Q9UK32|Q15418 | PROTEIN FAMILY | Homo sapiens | 83 | 128 | 9606 | FALSE |
| CHEMBL1942 | Alpha-2b adrenergic receptor | P18089 | SINGLE PROTEIN | Homo sapiens | 2744 | 4708 | 9606 | FALSE |
| CHEMBL5483085 | COP1-AGTL | Q96AD5|Q8NHY2 | PROTEIN-PROTEIN INTERACTION | Homo sapiens | 38 | 38 | 9606 | FALSE |
| CHEMBL5465229 | VHL/T-cell protein-tyrosine phosphatase | P17706|P40337 | PROTEIN-PROTEIN INTERACTION | Homo sapiens | 13 | 52 | 9606 | FALSE |
| CHEMBL5002 | Nuclear receptor subfamily 4 group A member 2 | P43354 | SINGLE PROTEIN | Homo sapiens | 279 | 712 | 9606 | FALSE |
| CHEMBL4106123 | MDM2-MDMX | Q00987|O15151 | PROTEIN-PROTEIN INTERACTION | Homo sapiens | 21 | 24 | 9606 | FALSE |
| CHEMBL5482971 | ICOS-ICOSL | O75144|Q9Y6W8 | PROTEIN-PROTEIN INTERACTION | Homo sapiens | 17 | 20 | 9606 | FALSE |
| CHEMBL3509582 | POU domain, class 2, transcription factor 2 | P09086 | SINGLE PROTEIN | Homo sapiens | 25 | 42 | 9606 | FALSE |
| CHEMBL4106136 | DNA-PK/Ku70/Ku85 | P78527|P12956|P13010 | PROTEIN COMPLEX | Homo sapiens | 23 | 23 | 9606 | FALSE |
| CHEMBL3885599 | LIM domain kinase 1/2 | P53671|P53667 | PROTEIN FAMILY | Homo sapiens | 19 | 20 | 9606 | FALSE |
| CHEMBL4296045 | Gamma-aminobutyric acid receptor subunit beta-1/beta-2 | P47870|P18505 | PROTEIN COMPLEX | Homo sapiens | 14 | 14 | 9606 | FALSE |
| CHEMBL1795166 | Sterol regulatory element-binding protein 2 | Q12772 | SINGLE PROTEIN | Homo sapiens | 31 | 50 | 9606 | FALSE |
| CHEMBL5847 | Aldo-keto reductase family 1 member C2 | P52895 | SINGLE PROTEIN | Homo sapiens | 669 | 778 | 9606 | FALSE |
| CHEMBL4069 | Corticotropin releasing factor receptor 2 | Q13324 | SINGLE PROTEIN | Homo sapiens | 380 | 399 | 9606 | FALSE |
| CHEMBL4630834 | Alpha N-terminal protein methyltransferase 1B | Q5VVY1 | SINGLE PROTEIN | Homo sapiens | 8 | 8 | 9606 | FALSE |
| CHEMBL4523640 | Gamma-aminobutyric acid receptor subunit alpha-4/beta-2/gamma-2 | P47870|P18507|P48169 | PROTEIN COMPLEX | Homo sapiens | 16 | 58 | 9606 | FALSE |
| CHEMBL4523196 | Tryptase beta-2 | P20231 | SINGLE PROTEIN | Homo sapiens | 17 | 24 | 9606 | FALSE |
| CHEMBL5874 | Solute carrier family 2, facilitated glucose transporter member 4 | P14672 | SINGLE PROTEIN | Homo sapiens | 381 | 396 | 9606 | FALSE |
| CHEMBL4106158 | AMP-activated protein kinase alpha-2/beta-2/gamma-2 | P54646|O43741|Q9UGJ0 | PROTEIN COMPLEX | Homo sapiens | 13 | 14 | 9606 | FALSE |
| CHEMBL5462 | Baculoviral IAP repeat-containing protein 2 | Q13490 | SINGLE PROTEIN | Homo sapiens | 776 | 1037 | 9606 | FALSE |
| CHEMBL4524132 | Calcium-activated potassium channel | Q92952|Q9UGI6|Q12791|O15554|Q9H2S1|A8MYU2 | PROTEIN FAMILY | Homo sapiens | 7 | 7 | 9606 | FALSE |
| CHEMBL4295830 | Myeloid leukemia factor 2 | Q15773 | SINGLE PROTEIN | Homo sapiens | 1 | 1 | 9606 | FALSE |
| CHEMBL1904 | Glutamate [NMDA] receptor subunit epsilon 2 | Q13224 | SINGLE PROTEIN | Homo sapiens | 334 | 507 | 9606 | FALSE |
| CHEMBL4296017 | GMP reductase 2 | Q9P2T1 | SINGLE PROTEIN | Homo sapiens | 49 | 49 | 9606 | FALSE |
| CHEMBL3301400 | Von Hippel-Lindau disease tumor suppressor/Elongin B/Elongin C | P40337|Q15370|Q15369 | PROTEIN COMPLEX | Homo sapiens | 90 | 230 | 9606 | FALSE |
| CHEMBL1075163 | Serine/threonine-protein kinase haspin | Q8TF76 | SINGLE PROTEIN | Homo sapiens | 682 | 960 | 9606 | FALSE |
| CHEMBL5465370 | Hyaluronan-binding protein 2 | Q14520 | SINGLE PROTEIN | Homo sapiens | 1 | 1 | 9606 | FALSE |
| CHEMBL4524021 | Mitotic spindle assembly checkpoint protein MAD2B | Q9UI95 | SINGLE PROTEIN | Homo sapiens | 1 | 1 | 9606 | FALSE |
| CHEMBL4112 | Retina-specific amine oxidase, copper containing | O75106 | SINGLE PROTEIN | Homo sapiens | 18 | 42 | 9606 | FALSE |
| CHEMBL4105828 | Tudor domain-containing protein 7 | Q8NHU6 | SINGLE PROTEIN | Homo sapiens | 2 | 4 | 9606 | FALSE |
| CHEMBL1255131 | Eukaryotic translation initiation factor 2 subunit 1 | P05198 | SINGLE PROTEIN | Homo sapiens | 82 | 143 | 9606 | FALSE |
| CHEMBL2777 | myosin light chain kinase 2 | Q9H1R3 | SINGLE PROTEIN | Homo sapiens | 361 | 456 | 9606 | FALSE |
| CHEMBL1971 | Sulfonylurea receptor 2 | O60706 | SINGLE PROTEIN | Homo sapiens | 56 | 109 | 9606 | FALSE |
| CHEMBL5912 | Serine/threonine-protein kinase 32B | Q9NY57 | SINGLE PROTEIN | Homo sapiens | 306 | 360 | 9606 | FALSE |
| CHEMBL4295903 | Lysophosphatidylcholine acyltransferase 1 | Q8NF37 | SINGLE PROTEIN | Homo sapiens | 1 | 1 | 9606 | FALSE |
| CHEMBL4105767 | Anoctamin-2 | Q9NQ90 | SINGLE PROTEIN | Homo sapiens | 39 | 52 | 9606 | FALSE |
| CHEMBL4523868 | Probable G-protein coupled receptor 85 | P60893 | SINGLE PROTEIN | Homo sapiens | 1 | 1 | 9606 | FALSE |
| CHEMBL4296500 | THLE-2 |  | CELL-LINE | Homo sapiens | 228 | 262 | 9606 | FALSE |
| CHEMBL2111346 | Serotonin and norepinephrine transporters (SERT/NET) | P31645|P23975 | SELECTIVITY GROUP | Homo sapiens | 83 | 90 | 9606 | FALSE |
| CHEMBL4888447 | E3 ubiquitin-protein ligase XIAP/Receptor-interacting serine/threonine-protein kinase 2 | P98170|O43353 | PROTEIN-PROTEIN INTERACTION | Homo sapiens | 21 | 23 | 9606 | FALSE |
| CHEMBL1743297 | Sulfotransferase family cytosolic 2B member 1 | O00204 | SINGLE PROTEIN | Homo sapiens | 3 | 4 | 9606 | FALSE |
| CHEMBL5483008 | VHL/LSD1/HDAC3/RCOR1 | O15379|O60341|P40337|Q9UKL0 | PROTEIN-PROTEIN INTERACTION | Homo sapiens | 1 | 1 | 9606 | FALSE |
| CHEMBL5483090 | cIAP1-alpha-Synuclein | Q13490|P37840 | PROTEIN-PROTEIN INTERACTION | Homo sapiens | 2 | 2 | 9606 | FALSE |
| CHEMBL4938 | Adenylate kinase 2 | P54819 | SINGLE PROTEIN | Homo sapiens | 252 | 264 | 9606 | FALSE |
| CHEMBL1628470 | Transient receptor potential cation channel subfamily M member 6 | Q9BX84 | SINGLE PROTEIN | Homo sapiens | 222 | 229 | 9606 | FALSE |
| CHEMBL5196 | Fatty acid transport protein 5 | Q9Y2P5 | SINGLE PROTEIN | Homo sapiens | 42 | 42 | 9606 | FALSE |
| CHEMBL5938 | Serine/threonine-protein kinase PLK2 | Q9NYY3 | SINGLE PROTEIN | Homo sapiens | 814 | 1066 | 9606 | FALSE |
| CHEMBL4229 | Hydroxyacid oxidase 1 | Q9UJM8 | SINGLE PROTEIN | Homo sapiens | 70 | 99 | 9606 | FALSE |
| CHEMBL4326 | Fatty acid transport protein 2 | O14975 | SINGLE PROTEIN | Homo sapiens | 44 | 44 | 9606 | FALSE |
| CHEMBL1075468 | HSC-2 |  | CELL-LINE | Homo sapiens | 516 | 772 | 9606 | FALSE |
| CHEMBL4296065 | Cyclin-dependent kinase 1/ G1/S-specific cyclin-D3 | P06493|P30281 | PROTEIN COMPLEX | Homo sapiens | 24 | 24 | 9606 | FALSE |
| CHEMBL4105775 | Meprin A subunit alpha | Q16819 | SINGLE PROTEIN | Homo sapiens | 9 | 11 | 9606 | FALSE |
| CHEMBL4105879 | Meprin A subunit beta | Q16820 | SINGLE PROTEIN | Homo sapiens | 90 | 94 | 9606 | FALSE |
| CHEMBL4105756 | STE20-related kinase adapter protein beta | Q9C0K7 | SINGLE PROTEIN | Homo sapiens | 12 | 20 | 9606 | FALSE |
| CHEMBL4739862 | Interferon-induced helicase C domain-containing protein 1 | Q9BYX4 | SINGLE PROTEIN | Homo sapiens | 1 | 1 | 9606 | FALSE |
| CHEMBL5465363 | N-acetyllactosaminide beta-1,3-N-acetylglucosaminyltransferase 2 | Q9NY97 | SINGLE PROTEIN | Homo sapiens | 42 | 42 | 9606 | FALSE |
| CHEMBL4748224 | VHL/Apoptosis regulator Bcl-2 | P10415|P40337 | PROTEIN-PROTEIN INTERACTION | Homo sapiens | 32 | 39 | 9606 | FALSE |
| CHEMBL4296117 | von Hippel-Lindau disease tumor suppressor/Elongin-C/Elongin-B/HIF1A | Q16665|P40337|Q15370|Q15369 | PROTEIN COMPLEX | Homo sapiens | 12 | 14 | 9606 | FALSE |
| CHEMBL2111336 | VEGF-receptor 2 and tyrosine-protein kinase SRC | P12931|P35968 | SELECTIVITY GROUP | Homo sapiens | 25 | 25 | 9606 | FALSE |
| CHEMBL4888448 | Syntenin-1/Syndecan-2 | O00560|P34741 | PROTEIN COMPLEX | Homo sapiens | 78 | 86 | 9606 | FALSE |
| CHEMBL2094254 | Cysteinyl leukotriene receptor | Q9Y271|Q9NS75 | PROTEIN FAMILY | Homo sapiens | 826 | 1147 | 9606 | FALSE |
| CHEMBL4523135 | Inositol hexakisphosphate and diphosphoinositol-pentakisphosphate kinase 2 | O43314 | SINGLE PROTEIN | Homo sapiens | 2 | 4 | 9606 | FALSE |
| CHEMBL4296116 | CRY2/PER2 | O15055|Q49AN0 | PROTEIN COMPLEX | Homo sapiens | 55 | 55 | 9606 | FALSE |
| CHEMBL4802030 | Baculoviral IAP repeat-containing protein 2/Prostaglandin E synthase | Q13490|O14684 | PROTEIN-PROTEIN INTERACTION | Homo sapiens | 1 | 1 | 9606 | FALSE |
| CHEMBL4106141 | Aurora-C/INCENP | Q9UQB9|Q9NQS7 | PROTEIN COMPLEX | Homo sapiens | 5 | 6 | 9606 | FALSE |
| CHEMBL4524011 | P2Y receptor | Q9H244|P51582|P47900|P41231|Q15391|Q15077|Q96G91|Q9BPV8 | PROTEIN FAMILY | Homo sapiens | 4 | 4 | 9606 | FALSE |
| CHEMBL2239 | Carbohydrate (N-acetylglucosamine 6-O) sulfotransferase 4 | Q8NCG5 | SINGLE PROTEIN | Homo sapiens | 2 | 4 | 9606 | FALSE |
| CHEMBL2109233 | Neuronal acetylcholine receptor; alpha3/alpha6/beta2/beta3 | P17787|P32297|Q15825|Q05901 | PROTEIN COMPLEX | Homo sapiens | 38 | 91 | 9606 | FALSE |
| CHEMBL1944495 | Proteasome subunit beta type-9 | P28065 | SINGLE PROTEIN | Homo sapiens | 196 | 310 | 9606 | FALSE |
| CHEMBL5291553 | Ceramide synthase 2 | Q96G23 | SINGLE PROTEIN | Homo sapiens | 2 | 3 | 9606 | FALSE |
| CHEMBL5169130 | Heat shock 70 kDa protein 4 | P34932 | SINGLE PROTEIN | Homo sapiens | 1 | 1 | 9606 | FALSE |
| CHEMBL6002 | Cell division cycle 2-like protein kinase 6 | Q9BWU1 | SINGLE PROTEIN | Homo sapiens | 484 | 563 | 9606 | FALSE |
| CHEMBL4376 | Dual-specificity tyrosine-phosphorylation regulated kinase 2 | Q92630 | SINGLE PROTEIN | Homo sapiens | 1730 | 2460 | 9606 | FALSE |
| CHEMBL4804 | Dual specificity phosphatase Cdc25B | P30305 | SINGLE PROTEIN | Homo sapiens | 863 | 1111 | 9606 | FALSE |
| CHEMBL4829 | Acetyl-CoA carboxylase 2 | O00763 | SINGLE PROTEIN | Homo sapiens | 3245 | 3474 | 9606 | FALSE |
| CHEMBL3492 | Proteasome Macropain subunit | P49721 | SINGLE PROTEIN | Homo sapiens | 700 | 1027 | 9606 | FALSE |
| CHEMBL2094130 | GABA-A receptor; alpha-2/beta-3/gamma-2 | P18507|P28472|P47869 | PROTEIN COMPLEX | Homo sapiens | 624 | 949 | 9606 | FALSE |
| CHEMBL2111429 | Histone deacetylase (HDAC1 and HDAC2) | Q13547|Q92769 | PROTEIN FAMILY | Homo sapiens | 558 | 628 | 9606 | FALSE |
| CHEMBL4370 | UDP-glucuronosyltransferase 2B7 | P16662 | SINGLE PROTEIN | Homo sapiens | 283 | 793 | 9606 | FALSE |
| CHEMBL3530 | Dual specificity mitogen-activated protein kinase kinase 7 | O14733 | SINGLE PROTEIN | Homo sapiens | 911 | 1180 | 9606 | FALSE |
| CHEMBL3060 | Glycine transporter 2 | Q9Y345 | SINGLE PROTEIN | Homo sapiens | 627 | 697 | 9606 | FALSE |
| CHEMBL5777 | Bcl-2-like protein 11 | O43521 | SINGLE PROTEIN | Homo sapiens | 8 | 8 | 9606 | FALSE |
| CHEMBL5315 | Peroxiredoxin-1 | Q06830 | SINGLE PROTEIN | Homo sapiens | 10 | 29 | 9606 | FALSE |
| CHEMBL5144 | Vasoactive intestinal polypeptide receptor 1 | P32241 | SINGLE PROTEIN | Homo sapiens | 1004 | 1906 | 9606 | FALSE |
| CHEMBL5427 | Homeodomain-interacting protein kinase 1 | Q86Z02 | SINGLE PROTEIN | Homo sapiens | 1009 | 1605 | 9606 | FALSE |
| CHEMBL1615383 | Solute carrier family 12 member 2 | P55011 | SINGLE PROTEIN | Homo sapiens | 42 | 75 | 9606 | FALSE |
| CHEMBL1615384 | Solute carrier family 12 member 5 | Q9H2X9 | SINGLE PROTEIN | Homo sapiens | 16 | 37 | 9606 | FALSE |
| CHEMBL1628473 | Relaxin-3 receptor 2 | Q8TDU9 | SINGLE PROTEIN | Homo sapiens | 51 | 124 | 9606 | FALSE |
| CHEMBL2111366 | GABA A receptor alpha-4/beta-3/gamma-2 | P18507|P28472|P48169 | PROTEIN COMPLEX | Homo sapiens | 90 | 109 | 9606 | FALSE |
| CHEMBL3885601 | Lysophosphatidic acid receptor 1/3 | Q9UBY5|Q92633 | PROTEIN FAMILY | Homo sapiens | 1 | 1 | 9606 | FALSE |
| CHEMBL2111468 | Dopamine D2 receptor and serotonin 2a receptor | P14416|P28223 | SELECTIVITY GROUP | Homo sapiens | 93 | 120 | 9606 | FALSE |
| CHEMBL5169122 | B-cell differentiation antigen CD72 | P21854 | SINGLE PROTEIN | Homo sapiens | 1 | 1 | 9606 | FALSE |
| CHEMBL3430887 | Induced myeloid leukemia cell differentiation protein Mcl-1/Bcl-2-like protein 11 | Q07820|O43521 | PROTEIN-PROTEIN INTERACTION | Homo sapiens | 94 | 104 | 9606 | FALSE |
| CHEMBL4630753 | Cyclin-dependent kinase 12/13 | Q14004|Q9NYV4 | PROTEIN FAMILY | Homo sapiens | 40 | 66 | 9606 | FALSE |
| CHEMBL5281 | Cytochrome P450 2E1 | P05181 | SINGLE PROTEIN | Homo sapiens | 1156 | 2177 | 9606 | FALSE |
| CHEMBL3886062 | Cytosolic carboxypeptidase 2 | Q5U5Z8 | SINGLE PROTEIN | Homo sapiens | 4 | 9 | 9606 | FALSE |
| CHEMBL3885536 | Canalicular multispecific organic anion transporter 1/Solute carrier organic anion transporter family member 1B1 | Q92887|Q9Y6L6 | PROTEIN FAMILY | Homo sapiens | 7 | 10 | 9606 | FALSE |
| CHEMBL5780 | Sodium/nucleoside cotransporter 2 | O43868 | SINGLE PROTEIN | Homo sapiens | 69 | 91 | 9606 | FALSE |
| CHEMBL4630760 | Sodium channel protein type 1 subunit alpha/beta-1/beta-2 | P35498|O60939|Q07699 | PROTEIN COMPLEX | Homo sapiens | 41 | 42 | 9606 | FALSE |
| CHEMBL3879824 | Redox-regulatory protein FAM213A | Q9BRX8 | SINGLE PROTEIN | Homo sapiens | 2 | 3 | 9606 | FALSE |
| CHEMBL3988638 | Voltage-dependent L-type calcium channel alpha1C/alpha2delta/beta1b | P54289|Q13936|Q02641 | PROTEIN COMPLEX | Homo sapiens | 52 | 64 | 9606 | FALSE |
| CHEMBL5519 | Serine/threonine-protein kinase DCLK2 | Q8N568 | SINGLE PROTEIN | Homo sapiens | 867 | 1477 | 9606 | FALSE |
| CHEMBL2321630 | Alpha-1,6-mannosyl-glycoprotein 2-beta-N-acetylglucosaminyltransferase | Q10469 | SINGLE PROTEIN | Homo sapiens | 527 | 722 | 9606 | FALSE |
| CHEMBL2382 | Calpain 2 | P17655 | SINGLE PROTEIN | Homo sapiens | 171 | 185 | 9606 | FALSE |
| CHEMBL5698 | NUAK family SNF1-like kinase 2 | Q9H093 | SINGLE PROTEIN | Homo sapiens | 530 | 666 | 9606 | FALSE |
| CHEMBL2109232 | Adrenomedullin receptor AM1; CALCRL/RAMP2 | O60895|Q16602 | PROTEIN COMPLEX | Homo sapiens | 8 | 8 | 9606 | FALSE |
| CHEMBL4742261 | Cereblon/Procollagen-lysine,2-oxoglutarate 5-dioxygenase 2 | Q96SW2|O00469 | PROTEIN-PROTEIN INTERACTION | Homo sapiens | 1 | 1 | 9606 | FALSE |
| CHEMBL5660 | Kinetochore protein NDC80 homolog | O14777 | SINGLE PROTEIN | Homo sapiens | 5 | 14 | 9606 | FALSE |
| CHEMBL3885643 | Toll-like receptor 1/2 | O60603|Q15399 | PROTEIN COMPLEX | Homo sapiens | 221 | 410 | 9606 | FALSE |
| CHEMBL4105907 | Sigma intracellular receptor 2 | Q5BJF2 | SINGLE PROTEIN | Homo sapiens | 977 | 1345 | 9606 | FALSE |
| CHEMBL4295923 | Pyrroline-5-carboxylate reductase 2 | Q96C36 | SINGLE PROTEIN | Homo sapiens | 1 | 2 | 9606 | FALSE |
| CHEMBL5465207 | Cereblon/T-cell protein-tyrosine phosphatase | P17706|Q96SW2 | PROTEIN-PROTEIN INTERACTION | Homo sapiens | 5 | 5 | 9606 | FALSE |
| CHEMBL4234 | Estradiol 17-beta-dehydrogenase 3 | P37058 | SINGLE PROTEIN | Homo sapiens | 377 | 821 | 9606 | FALSE |
| CHEMBL5014 | Serine/threonine-protein kinase RIPK2 | O43353 | SINGLE PROTEIN | Homo sapiens | 1114 | 1617 | 9606 | FALSE |
| CHEMBL1870 | Retinoid X receptor beta | P28702 | SINGLE PROTEIN | Homo sapiens | 449 | 803 | 9606 | FALSE |
| CHEMBL1743121 | Solute carrier organic anion transporter family member 1B3 | Q9NPD5 | SINGLE PROTEIN | Homo sapiens | 2121 | 2517 | 9606 | FALSE |
| CHEMBL5856 | Serine/threonine-protein kinase PFTAIRE-2 | Q96Q40 | SINGLE PROTEIN | Homo sapiens | 234 | 247 | 9606 | FALSE |
| CHEMBL3430901 | Voltage-dependent N-type calcium channel subunit alpha-1B/Voltage-dependent calcium channel subunit alpha-2/delta-1/Voltage-dependent L-type calcium channel subunit beta-3 | P54289|Q00975|P54284 | PROTEIN COMPLEX | Homo sapiens | 1 | 2 | 9606 | FALSE |
| CHEMBL5465235 | VHL/Probable global transcription activator SNF2L2 | P51531|P40337 | PROTEIN-PROTEIN INTERACTION | Homo sapiens | 34 | 96 | 9606 | FALSE |
| CHEMBL2366260 | LC-2-ad |  | CELL-LINE | Homo sapiens | 173 | 184 | 9606 | FALSE |
| CHEMBL2331067 | Alkaline ceramidase 2 | Q5QJU3 | SINGLE PROTEIN | Homo sapiens | 1 | 1 | 9606 | FALSE |
| CHEMBL4106163 | AMP-activated protein kinase alpha-1/beta-2/gamma-2 | O43741|Q9UGJ0|Q13131 | PROTEIN COMPLEX | Homo sapiens | 2 | 3 | 9606 | FALSE |
| CHEMBL3430896 | Histone deacetylase 1/2/3/6 | Q13547|O15379|Q9UBN7|Q92769 | PROTEIN FAMILY | Homo sapiens | 29 | 89 | 9606 | FALSE |
| CHEMBL4523650 | Proto-oncogene DBL/Transforming protein RhoA | P61586|P10911 | PROTEIN COMPLEX | Homo sapiens | 1 | 1 | 9606 | FALSE |
| CHEMBL3217393 | Transforming growth factor beta-2 | P61812 | SINGLE PROTEIN | Homo sapiens | 5 | 5 | 9606 | FALSE |
| CHEMBL3988637 | Transforming growth factor beta | P01137|P61812|P10600 | PROTEIN FAMILY | Homo sapiens | 1 | 1 | 9606 | FALSE |
| CHEMBL3123 | Death-associated protein kinase 2 | Q9UIK4 | SINGLE PROTEIN | Homo sapiens | 510 | 769 | 9606 | FALSE |
| CHEMBL2095200 | Serotonin 2 (5-HT2) receptor | P41595|P28223|P28335 | PROTEIN FAMILY | Homo sapiens | 241 | 283 | 9606 | FALSE |
| CHEMBL3038469 | CDK2/Cyclin A2 | P24941|P20248 | PROTEIN COMPLEX | Homo sapiens | 2000 | 2318 | 9606 | FALSE |
| CHEMBL3309105 | Taste receptor type 2 member 14 | Q9NYV8 | SINGLE PROTEIN | Homo sapiens | 166 | 240 | 9606 | FALSE |
| CHEMBL1932895 | Natural resistance-associated macrophage protein 2 | P49281 | SINGLE PROTEIN | Homo sapiens | 53 | 70 | 9606 | FALSE |
| CHEMBL6114 | Phospholipase A-2-activating protein | Q9Y263 | SINGLE PROTEIN | Homo sapiens | 41 | 44 | 9606 | FALSE |
| CHEMBL5618 | Aryl hydrocarbon receptor nuclear translocator | P27540 | SINGLE PROTEIN | Homo sapiens | 15 | 25 | 9606 | FALSE |
| CHEMBL5871 | Glycine receptor subunit alpha-2 | P23416 | SINGLE PROTEIN | Homo sapiens | 38 | 49 | 9606 | FALSE |
| CHEMBL2111414 | Tyrosine-protein kinase ABL | P00519|P42684 | PROTEIN FAMILY | Homo sapiens | 160 | 162 | 9606 | FALSE |
| CHEMBL4742303 | Cereblon/E3 ubiquitin-protein ligase DTX3L | Q96SW2|Q8TDB6 | PROTEIN-PROTEIN INTERACTION | Homo sapiens | 1 | 1 | 9606 | FALSE |
| CHEMBL4105887 | Guanine nucleotide-binding protein G(i) subunit alpha-2 | P04899 | SINGLE PROTEIN | Homo sapiens | 179 | 180 | 9606 | FALSE |
| CHEMBL1743127 | Multidrug and toxin extrusion protein 2 | Q86VL8 | SINGLE PROTEIN | Homo sapiens | 47 | 58 | 9606 | FALSE |
| CHEMBL4225 | Dual specificity protein kinase CLK2 | P49760 | SINGLE PROTEIN | Homo sapiens | 3263 | 4285 | 9606 | FALSE |
| CHEMBL2176856 | C-type lectin domain family 6 member A | Q6EIG7 | SINGLE PROTEIN | Homo sapiens | 6 | 6 | 9606 | FALSE |
| CHEMBL4159 | Endoplasmic reticulum-associated amyloid beta-peptide-binding protein | Q99714 | SINGLE PROTEIN | Homo sapiens | 15343 | 20754 | 9606 | FALSE |
| CHEMBL2046259 | Kynurenine/alpha-aminoadipate aminotransferase, mitochondrial | Q8N5Z0 | SINGLE PROTEIN | Homo sapiens | 76 | 211 | 9606 | FALSE |
| CHEMBL3562162 | Neutral amino acid transporter B(0) | Q15758 | SINGLE PROTEIN | Homo sapiens | 54 | 65 | 9606 | FALSE |
| CHEMBL2221345 | Hypoxia inducible factors; HIF-1-alpha, HIF-2-alpha | Q16665|Q99814 | PROTEIN FAMILY | Homo sapiens | 440 | 800 | 9606 | FALSE |
| CHEMBL4742321 | Cereblon/Transmembrane protease serine 4 | Q9NRS4|Q96SW2 | PROTEIN-PROTEIN INTERACTION | Homo sapiens | 1 | 1 | 9606 | FALSE |
| CHEMBL4296143 | von Hippel-Lindau disease tumor suppressor/Bromodomain-containing protein 2 | P25440|P40337 | PROTEIN-PROTEIN INTERACTION | Homo sapiens | 23 | 53 | 9606 | FALSE |
| CHEMBL4295730 | Receptor-type tyrosine-protein phosphatase zeta | P23471 | SINGLE PROTEIN | Homo sapiens | 17 | 43 | 9606 | FALSE |
| CHEMBL5482973 | SKP1-SKP2-CKS1 | P61024|Q13309|P63208 | PROTEIN-PROTEIN INTERACTION | Homo sapiens | 89 | 89 | 9606 | FALSE |
| CHEMBL4523964 | COX-1/COX-2 | P35354|P23219 | SELECTIVITY GROUP | Homo sapiens | 283 | 292 | 9606 | FALSE |
| CHEMBL2734 | Phospholipase D2 | O14939 | SINGLE PROTEIN | Homo sapiens | 313 | 437 | 9606 | FALSE |
| CHEMBL2150837 | ATPase family AAA domain-containing protein 2 | Q6PL18 | SINGLE PROTEIN | Homo sapiens | 384 | 605 | 9606 | FALSE |
| CHEMBL2095172 | GABA-A receptor; alpha-1/beta-2/gamma-2 | P14867|P47870|P18507 | PROTEIN COMPLEX | Homo sapiens | 675 | 1171 | 9606 | FALSE |
| CHEMBL3774295 | Lysine-specific demethylase 5B | Q9UGL1 | SINGLE PROTEIN | Homo sapiens | 657 | 938 | 9606 | FALSE |
| CHEMBL1075189 | Pyruvate kinase isozymes M1/M2 | P14618 | SINGLE PROTEIN | Homo sapiens | 7145 | 14931 | 9606 | FALSE |
| CHEMBL3683 | Glutamate receptor ionotropic kainate 2 | Q13002 | SINGLE PROTEIN | Homo sapiens | 209 | 370 | 9606 | FALSE |
| CHEMBL3003 | Nuclear factor NF-kappa-B p100/p49 subunits | Q00653 | SINGLE PROTEIN | Homo sapiens | 6 | 8 | 9606 | FALSE |
| CHEMBL2052034 | Solute carrier family 2, facilitated glucose transporter member 9 | Q9NRM0 | SINGLE PROTEIN | Homo sapiens | 13 | 16 | 9606 | FALSE |
| CHEMBL615011 | TE-2 |  | CELL-LINE | Homo sapiens | 6 | 11 | 9606 | FALSE |
| CHEMBL4015 | C-C chemokine receptor type 2 | P41597 | SINGLE PROTEIN | Homo sapiens | 3113 | 5687 | 9606 | FALSE |
| CHEMBL3116 | Cyclin-dependent kinase 9 | P50750 | SINGLE PROTEIN | Homo sapiens | 1995 | 2698 | 9606 | FALSE |
| CHEMBL3430911 | Serine/threonine-protein kinase Aurora | Q96GD4|Q9UQB9|O14965 | PROTEIN FAMILY | Homo sapiens | 2 | 2 | 9606 | FALSE |
| CHEMBL3298 | Alpha enolase | P06733 | SINGLE PROTEIN | Homo sapiens | 15 | 29 | 9606 | FALSE |
| CHEMBL4523330 | Disabled homolog 2-interacting protein | Q5VWQ8 | SINGLE PROTEIN | Homo sapiens | 8 | 17 | 9606 | FALSE |
| CHEMBL3559704 | Taste receptor type 2 member 10 | Q9NYW0 | SINGLE PROTEIN | Homo sapiens | 13 | 51 | 9606 | FALSE |
| CHEMBL4523253 | Taste receptor type 2 member 46 | P59540 | SINGLE PROTEIN | Homo sapiens | 24 | 155 | 9606 | FALSE |
| CHEMBL4524041 | Son of sevenless homolog 2 | Q07890 | SINGLE PROTEIN | Homo sapiens | 10 | 10 | 9606 | FALSE |
| CHEMBL3885631 | Retinoic acid receptor RXR-alpha/Retinoic acid receptor beta | P10826|P19793 | PROTEIN COMPLEX | Homo sapiens | 11 | 11 | 9606 | FALSE |
| CHEMBL2971 | Tyrosine-protein kinase JAK2 | O60674 | SINGLE PROTEIN | Homo sapiens | 10345 | 13487 | 9606 | FALSE |
| CHEMBL4296043 | Calmodulin-2 | P0DP24 | SINGLE PROTEIN | Homo sapiens | 1 | 1 | 9606 | FALSE |
| CHEMBL3588731 | Bromodomain-containing protein 8 | Q9H0E9 | SINGLE PROTEIN | Homo sapiens | 26 | 42 | 9606 | FALSE |
| CHEMBL4179 | c-Jun N-terminal kinase 2 | P45984 | SINGLE PROTEIN | Homo sapiens | 3716 | 4887 | 9606 | FALSE |
| CHEMBL2363071 | Retinoid receptor | P28702|P13631|P48443|P10826|P10276|P19793 | PROTEIN FAMILY | Homo sapiens | 1 | 1 | 9606 | FALSE |
| CHEMBL2453 | AMP-activated protein kinase, gamma-2 subunit | Q9UGJ0 | SINGLE PROTEIN | Homo sapiens | 243 | 243 | 9606 | FALSE |
| CHEMBL5169082 | CRBN/AKT2 | P31751|Q96SW2 | PROTEIN-PROTEIN INTERACTION | Homo sapiens | 2 | 2 | 9606 | FALSE |
| CHEMBL5169089 | CRBN/PARP2 | Q9UGN5|Q96SW2 | PROTEIN-PROTEIN INTERACTION | Homo sapiens | 1 | 1 | 9606 | FALSE |
| CHEMBL4295744 | Peroxiredoxin-2 | P32119 | SINGLE PROTEIN | Homo sapiens | 4 | 16 | 9606 | FALSE |
| CHEMBL4296247 | Cry1/Cry2 | Q49AN0|Q16526 | PROTEIN FAMILY | Homo sapiens | 244 | 247 | 9606 | FALSE |
| CHEMBL2021754 | Neutral ceramidase | Q9NR71 | SINGLE PROTEIN | Homo sapiens | 51 | 95 | 9606 | FALSE |
| CHEMBL2176858 | C-type lectin domain family 4 member M | Q9H2X3 | SINGLE PROTEIN | Homo sapiens | 82 | 115 | 9606 | FALSE |
| CHEMBL4447 | Kallikrein 5 | Q9Y337 | SINGLE PROTEIN | Homo sapiens | 208 | 307 | 9606 | FALSE |
| CHEMBL4374 | Photoreceptor-specific nuclear receptor | Q9Y5X4 | SINGLE PROTEIN | Homo sapiens | 483 | 502 | 9606 | FALSE |
| CHEMBL5119 | Zinc finger protein GLI2 | P10070 | SINGLE PROTEIN | Homo sapiens | 13 | 13 | 9606 | FALSE |
| CHEMBL4202 | Serine/threonine-protein kinase 2 | Q9H2G2 | SINGLE PROTEIN | Homo sapiens | 1430 | 1693 | 9606 | FALSE |
| CHEMBL1649052 | C-C motif chemokine 2 | P13500 | SINGLE PROTEIN | Homo sapiens | 73 | 105 | 9606 | FALSE |
| CHEMBL4523249 | Taste receptor type 2 member 39 | P59534 | SINGLE PROTEIN | Homo sapiens | 46 | 48 | 9606 | FALSE |
| CHEMBL3430883 | Retinoic acid receptor alpha/Retinoid X receptor alpha | P10276|P19793 | PROTEIN COMPLEX | Homo sapiens | 21 | 28 | 9606 | FALSE |
| CHEMBL2169716 | N-lysine methyltransferase SMYD2 | Q9NRG4 | SINGLE PROTEIN | Homo sapiens | 188 | 410 | 9606 | FALSE |
| CHEMBL4901 | Ephrin type-B receptor 3 | P54753 | SINGLE PROTEIN | Homo sapiens | 1198 | 1925 | 9606 | FALSE |
| CHEMBL4523301 | Transcriptional enhancer factor TEF-4 | Q15562 | SINGLE PROTEIN | Homo sapiens | 46 | 96 | 9606 | FALSE |
| CHEMBL5051 | Transient receptor potential cation channel subfamily V member 2 | Q9Y5S1 | SINGLE PROTEIN | Homo sapiens | 13 | 25 | 9606 | FALSE |
| CHEMBL4523641 | Gamma-aminobutyric acid receptor subunit alpha-5/beta-2/gamma-2 | P47870|P18507|P31644 | PROTEIN COMPLEX | Homo sapiens | 44 | 82 | 9606 | FALSE |
| CHEMBL6032 | Histone-lysine N-methyltransferase, H3 lysine-9 specific 3 | Q96KQ7 | SINGLE PROTEIN | Homo sapiens | 91188 | 93144 | 9606 | FALSE |
| CHEMBL3760 | Adenylate cyclase type II | Q08462 | SINGLE PROTEIN | Homo sapiens | 125 | 177 | 9606 | FALSE |
| CHEMBL3627583 | Kinase suppressor of Ras 2 | Q6VAB6 | SINGLE PROTEIN | Homo sapiens | 24 | 36 | 9606 | FALSE |
| CHEMBL1741187 | Importin subunit alpha-2 | P52292 | SINGLE PROTEIN | Homo sapiens | 14 | 14 | 9606 | FALSE |
| CHEMBL212 | Integrin alpha-IIb | P08514 | SINGLE PROTEIN | Homo sapiens | 32 | 36 | 9606 | FALSE |
| CHEMBL3883286 | Bcl-xL/BAX | Q07817|Q07812 | PROTEIN-PROTEIN INTERACTION | Homo sapiens | 4 | 4 | 9606 | FALSE |
| CHEMBL1255167 | Axin-2 | Q9Y2T1 | SINGLE PROTEIN | Homo sapiens | 12 | 22 | 9606 | FALSE |
| CHEMBL1613741 | Taste receptor type 1 member 2 | Q8TE23 | SINGLE PROTEIN | Homo sapiens | 3 | 15 | 9606 | FALSE |
| CHEMBL3714292 | Ecto-NOX disulfide-thiol exchanger 2 | Q16206 | SINGLE PROTEIN | Homo sapiens | 1 | 2 | 9606 | FALSE |
| CHEMBL1075490 | LK-2 |  | CELL-LINE | Homo sapiens | 111 | 114 | 9606 | FALSE |
| CHEMBL3038498 | Keap1/Nrf2 | Q16236|Q14145 | PROTEIN-PROTEIN INTERACTION | Homo sapiens | 989 | 1824 | 9606 | FALSE |
| CHEMBL614356 | ML-2 |  | CELL-LINE | Homo sapiens | 190 | 244 | 9606 | FALSE |
| CHEMBL4802035 | JAK2/JAK3 | P52333|O60674 | PROTEIN FAMILY | Homo sapiens | 1 | 4 | 9606 | FALSE |
| CHEMBL2366232 | SK-MM-2 |  | CELL-LINE | Homo sapiens | 79 | 80 | 9606 | FALSE |
| CHEMBL4523699 | Protein cereblon/STAT2 | Q96SW2|P52630 | PROTEIN-PROTEIN INTERACTION | Homo sapiens | 4 | 10 | 9606 | FALSE |
| CHEMBL2098 | TAR RNA binding protein 2 | Q15633 | SINGLE PROTEIN | Homo sapiens | 14 | 21 | 9606 | FALSE |
| CHEMBL4296098 | Mitogen-activated protein kinase 8/9 | P45983|P45984 | PROTEIN FAMILY | Homo sapiens | 7 | 11 | 9606 | FALSE |
| CHEMBL5600 | Serine/threonine-protein kinase c-TAK1 | P27448 | SINGLE PROTEIN | Homo sapiens | 1915 | 2592 | 9606 | FALSE |
| CHEMBL1932896 | Ezrin | P15311 | SINGLE PROTEIN | Homo sapiens | 16 | 16 | 9606 | FALSE |
| CHEMBL4523702 | Protein cereblon/E3 ubiquitin-protein ligase Mdm2 | Q00987|Q96SW2 | PROTEIN-PROTEIN INTERACTION | Homo sapiens | 53 | 233 | 9606 | FALSE |
| CHEMBL4106184 | CDK4/CDK9 | P11802|P50750 | PROTEIN FAMILY | Homo sapiens | 1 | 6 | 9606 | FALSE |
| CHEMBL2396512 | Mothers against decapentaplegic homolog 2 | Q15796 | SINGLE PROTEIN | Homo sapiens | 9 | 9 | 9606 | FALSE |
| CHEMBL4295930 | Calponin-2 | Q99439 | SINGLE PROTEIN | Homo sapiens | 1 | 2 | 9606 | FALSE |
| CHEMBL2111384 | Nicotinic acetylcholine receptor alpha3/betaX | P11230|P17787|P30926|P32297|Q05901 | PROTEIN COMPLEX GROUP | Homo sapiens | 42 | 45 | 9606 | FALSE |
| CHEMBL3337327 | Tau-tubulin kinase 2 | Q6IQ55 | SINGLE PROTEIN | Homo sapiens | 111 | 113 | 9606 | FALSE |
| CHEMBL2111370 | GABA A receptor alpha-6/beta-2/gamma-2 | P47870|P18507|Q16445 | PROTEIN COMPLEX | Homo sapiens | 23 | 27 | 9606 | FALSE |
| CHEMBL4100 | Phospholipase C-gamma-2 | P16885 | SINGLE PROTEIN | Homo sapiens | 43 | 80 | 9606 | FALSE |
| CHEMBL3180 | Carboxylesterase 2 | O00748 | SINGLE PROTEIN | Homo sapiens | 371 | 584 | 9606 | FALSE |
| CHEMBL4105852 | Eukaryotic translation initiation factor 5B | O60841 | SINGLE PROTEIN | Homo sapiens | 170 | 170 | 9606 | FALSE |
| CHEMBL3885522 | Baculoviral IAP repeat-containing protein 3/Mucosa-associated lymphoid tissue lymphoma translocation protein 1 | Q13489|Q9UDY8 | CHIMERIC PROTEIN | Homo sapiens | 7 | 7 | 9606 | FALSE |
| CHEMBL4330 | Cysteinyl leukotriene receptor 2 | Q9NS75 | SINGLE PROTEIN | Homo sapiens | 119 | 135 | 9606 | FALSE |
| CHEMBL5291974 | BRD2/BRD3 | P25440|Q15059 | PROTEIN FAMILY | Homo sapiens | 2 | 2 | 9606 | FALSE |
| CHEMBL4295838 | Leucine-rich repeat flightless-interacting protein 1 | Q32MZ4 | SINGLE PROTEIN | Homo sapiens | 1 | 1 | 9606 | FALSE |
| CHEMBL4295957 | Acyl-coenzyme A thioesterase 13 | Q9NPJ3 | SINGLE PROTEIN | Homo sapiens | 1 | 1 | 9606 | FALSE |
| CHEMBL4105835 | Ubiquitin-conjugating enzyme E2 K | P61086 | SINGLE PROTEIN | Homo sapiens | 10 | 15 | 9606 | FALSE |
| CHEMBL4295866 | DCN1-like protein 2 | Q6PH85 | SINGLE PROTEIN | Homo sapiens | 5 | 6 | 9606 | FALSE |
| CHEMBL3038489 | Kir3.1/Kir3.2 | P48549|P48051 | PROTEIN COMPLEX | Homo sapiens | 222 | 445 | 9606 | FALSE |
| CHEMBL2095189 | Platelet-derived growth factor receptor | P09619|P16234 | PROTEIN COMPLEX | Homo sapiens | 476 | 507 | 9606 | FALSE |
| CHEMBL3430864 | Dimethylaniline monooxygenase [N-oxide-forming] 3 | P31513 | SINGLE PROTEIN | Homo sapiens | 4 | 9 | 9606 | FALSE |
| CHEMBL2111470 | Phosphodiesterase 2 and 5 (PDE2 and PDE5) | O76074|O00408 | SELECTIVITY GROUP | Homo sapiens | 30 | 34 | 9606 | FALSE |
| CHEMBL4879410 | [3-methyl-2-oxobutanoate dehydrogenase [lipoamide]] kinase, mitochondrial | O14874 | SINGLE PROTEIN | Homo sapiens | 5 | 8 | 9606 | FALSE |
| CHEMBL2111435 | Annexin A2/S100-A10 | P07355|P60903 | PROTEIN-PROTEIN INTERACTION | Homo sapiens | 92 | 201 | 9606 | FALSE |
| CHEMBL4523672 | SCN2A/SCN1B | Q99250|Q07699 | PROTEIN COMPLEX | Homo sapiens | 5 | 5 | 9606 | FALSE |
| CHEMBL4296134 | Cereblon/Cullin-4A/Bromodomain-containing protein 2 | P25440|Q96SW2|Q13619 | PROTEIN-PROTEIN INTERACTION | Homo sapiens | 19 | 31 | 9606 | FALSE |
| CHEMBL2095204 | Trypsin | P07477|P07478|P35030 | PROTEIN FAMILY | Homo sapiens | 2015 | 2222 | 9606 | FALSE |
| CHEMBL2109244 | GABA-A receptor; agonist GABA site | P14867|P47870|P28472|P48169|P34903|P18505|P47869|P31644|Q16445 | PROTEIN COMPLEX GROUP | Homo sapiens | 97 | 140 | 9606 | FALSE |
| CHEMBL3864 | Protein-tyrosine phosphatase 2C | Q06124 | SINGLE PROTEIN | Homo sapiens | 1692 | 2516 | 9606 | FALSE |
| CHEMBL3784904 | Inhibitor of growth protein 2 | Q9H160 | SINGLE PROTEIN | Homo sapiens | 7 | 8 | 9606 | FALSE |
| CHEMBL4523689 | Protein cereblon/Cyclin-dependent kinase 2 | P24941|Q96SW2 | PROTEIN-PROTEIN INTERACTION | Homo sapiens | 18 | 27 | 9606 | FALSE |
| CHEMBL3430897 | Histone deacetylase 1/2/3 | Q13547|O15379|Q92769 | PROTEIN FAMILY | Homo sapiens | 75 | 170 | 9606 | FALSE |
| CHEMBL3706558 | eIF-2-alpha kinase activator GCN1 | Q92616 | SINGLE PROTEIN | Homo sapiens | 4 | 9 | 9606 | FALSE |
| CHEMBL2111390 | Beta-secretase (BACE) | Q9Y5Z0|P56817 | PROTEIN FAMILY | Homo sapiens | 56 | 58 | 9606 | FALSE |
| CHEMBL5048 | Neutral cholesterol ester hydrolase 1 | Q6PIU2 | SINGLE PROTEIN | Homo sapiens | 128 | 158 | 9606 | FALSE |
| CHEMBL5873 | Solute carrier family 2, facilitated glucose transporter member 2 | P11168 | SINGLE PROTEIN | Homo sapiens | 422 | 428 | 9606 | FALSE |
| CHEMBL4739683 | RNA cytosine C(5)-methyltransferase NSUN2 | Q08J23 | SINGLE PROTEIN | Homo sapiens | 11 | 11 | 9606 | FALSE |
| CHEMBL1649059 | Serine/threonine-protein kinase VRK2 | Q86Y07 | SINGLE PROTEIN | Homo sapiens | 526 | 773 | 9606 | FALSE |
| CHEMBL5483007 | VHL/LSD1/HDAC2/RCOR1 | Q92769|O60341|P40337|Q9UKL0 | PROTEIN-PROTEIN INTERACTION | Homo sapiens | 1 | 1 | 9606 | FALSE |
| CHEMBL5465210 | Cereblon/Hexokinase type II | P52789|Q96SW2 | PROTEIN-PROTEIN INTERACTION | Homo sapiens | 3 | 5 | 9606 | FALSE |
| CHEMBL4879483 | Fatty acid 2-hydroxylase | Q7L5A8 | SINGLE PROTEIN | Homo sapiens | 3 | 3 | 9606 | FALSE |
| CHEMBL5267 | ADM2 | Q7Z4H4 | SINGLE PROTEIN | Homo sapiens | 28 | 28 | 9606 | FALSE |
| CHEMBL3713541 | Calcitonin gene-related peptide 2 | P10092 | SINGLE PROTEIN | Homo sapiens | 24 | 24 | 9606 | FALSE |
| CHEMBL3751646 | Adenosylhomocysteinase 2 | O43865 | SINGLE PROTEIN | Homo sapiens | 5 | 5 | 9606 | FALSE |
| CHEMBL4973 | Excitatory amino acid transporter 2 | P43004 | SINGLE PROTEIN | Homo sapiens | 296 | 562 | 9606 | FALSE |
| CHEMBL4296148 | von Hippel-Lindau disease tumor suppressor/Elongin-B/Elongin-C/Bromodomain-containing protein 4 | O60885|P40337|Q15370|Q15369 | PROTEIN-PROTEIN INTERACTION | Homo sapiens | 11 | 54 | 9606 | FALSE |
| CHEMBL3580523 | Prosaposin | P07602 | SINGLE PROTEIN | Homo sapiens | 3 | 12 | 9606 | FALSE |
| CHEMBL3885523 | MCL1-BAK1 complex | Q07820|Q16611 | PROTEIN COMPLEX | Homo sapiens | 31 | 39 | 9606 | FALSE |
| CHEMBL3038451 | AMPK alpha1/beta1/gamma2 | Q9UGJ0|Q9Y478|Q13131 | PROTEIN COMPLEX | Homo sapiens | 16 | 17 | 9606 | FALSE |
| CHEMBL5626 | SHC-transforming protein 1 | P29353 | SINGLE PROTEIN | Homo sapiens | 65 | 70 | 9606 | FALSE |
| CHEMBL4523724 | VHL/Steroid hormone receptor ERR2 | O95718|P40337 | PROTEIN-PROTEIN INTERACTION | Homo sapiens | 1 | 1 | 9606 | FALSE |
| CHEMBL4607 | Angiotensin II type 2 (AT-2) receptor | P50052 | SINGLE PROTEIN | Homo sapiens | 1520 | 2563 | 9606 | FALSE |
| CHEMBL1907597 | GABA-A receptor; GABA-A site (alpha1/beta2 interface) | P14867|P47870 | PROTEIN COMPLEX | Homo sapiens | 47 | 116 | 9606 | FALSE |
| CHEMBL4523129 | Synaptojanin-2 | O15056 | SINGLE PROTEIN | Homo sapiens | 32 | 76 | 9606 | FALSE |
| CHEMBL2034804 | Taste receptor type 2 member 31 | P59538 | SINGLE PROTEIN | Homo sapiens | 22 | 86 | 9606 | FALSE |
| CHEMBL3309106 | Taste receptor type 2 member 16 | Q9NYV7 | SINGLE PROTEIN | Homo sapiens | 27 | 102 | 9606 | FALSE |
| CHEMBL4245 | Estrogen-related receptor gamma | P62508 | SINGLE PROTEIN | Homo sapiens | 308 | 593 | 9606 | FALSE |
| CHEMBL2675 | Glutamate receptor ionotropic kainate 5 | Q16478 | SINGLE PROTEIN | Homo sapiens | 40 | 45 | 9606 | FALSE |
| CHEMBL5639 | Serine/threonine-protein kinase WNK2 | Q9Y3S1 | SINGLE PROTEIN | Homo sapiens | 392 | 596 | 9606 | FALSE |
| CHEMBL4523239 | Signal transducer and activator of transcription 2 | P52630 | SINGLE PROTEIN | Homo sapiens | 5 | 18 | 9606 | FALSE |
| CHEMBL5335 | Baculoviral IAP repeat-containing protein 3 | Q13489 | SINGLE PROTEIN | Homo sapiens | 211 | 337 | 9606 | FALSE |
| CHEMBL1955711 | Solute carrier family 22 member 7 | Q9Y694 | SINGLE PROTEIN | Homo sapiens | 31 | 39 | 9606 | FALSE |
| CHEMBL1743124 | Solute carrier organic anion transporter family member 2B1 | O94956 | SINGLE PROTEIN | Homo sapiens | 302 | 580 | 9606 | FALSE |
| CHEMBL5699 | Serine/threonine-protein kinase SIK2 | Q9H0K1 | SINGLE PROTEIN | Homo sapiens | 1063 | 1652 | 9606 | FALSE |
| CHEMBL2189137 | Lysosomal thioesterase PPT2 | Q9UMR5 | SINGLE PROTEIN | Homo sapiens | 1 | 1 | 9606 | FALSE |
| CHEMBL5483188 | CDK11B/Cyclin L2 | P21127|Q96S94 | PROTEIN COMPLEX | Homo sapiens | 4 | 4 | 9606 | FALSE |
| CHEMBL5483187 | CDK11A/Cyclin L2 | Q9UQ88|Q96S94 | PROTEIN COMPLEX | Homo sapiens | 4 | 4 | 9606 | FALSE |
| CHEMBL3411 | Prenyl protein specific protease | Q9Y256 | SINGLE PROTEIN | Homo sapiens | 299 | 365 | 9606 | FALSE |
| CHEMBL2730 | Protein-glutamine gamma-glutamyltransferase | P21980 | SINGLE PROTEIN | Homo sapiens | 673 | 1270 | 9606 | FALSE |
| CHEMBL2096665 | Pyruvate dehydrogenase kinase | Q15119|Q15120|Q16654|Q15118 | PROTEIN FAMILY | Homo sapiens | 452 | 522 | 9606 | FALSE |
| CHEMBL1907602 | Cyclin-dependent kinase 1/cyclin B1 | P06493|P14635 | PROTEIN COMPLEX | Homo sapiens | 1489 | 1898 | 9606 | FALSE |
| CHEMBL2096661 | Intercellular adhesion molecule (ICAM-1), Integrin alpha-L/beta-2 | P20701|P05362|P05107 | PROTEIN COMPLEX | Homo sapiens | 588 | 760 | 9606 | FALSE |
| CHEMBL1961791 | Nuclear receptor subfamily 2 group F member 6 | P10588 | SINGLE PROTEIN | Homo sapiens | 2 | 2 | 9606 | FALSE |
| CHEMBL2896 | cGMP-dependent protein kinase 2 | Q13237 | SINGLE PROTEIN | Homo sapiens | 1186 | 1651 | 9606 | FALSE |
| CHEMBL4295884 | Hermansky-Pudlak syndrome 6 protein | Q86YV9 | SINGLE PROTEIN | Homo sapiens | 1 | 1 | 9606 | FALSE |
| CHEMBL2221346 | Neuronal acetylcholine receptors; alpha-3/beta-4, alpha-5, beta-2 | P17787|P30926|P32297|P30532 | PROTEIN COMPLEX GROUP | Homo sapiens | 15 | 45 | 9606 | FALSE |
| CHEMBL2321628 | C-C chemokine receptor type 10 | P46092 | SINGLE PROTEIN | Homo sapiens | 141 | 178 | 9606 | FALSE |
| CHEMBL2366181 | DOHH-2 |  | CELL-LINE | Homo sapiens | 282 | 361 | 9606 | FALSE |
| CHEMBL1293237 | Bloom syndrome protein | P54132 | SINGLE PROTEIN | Homo sapiens | 3038 | 4307 | 9606 | FALSE |
| CHEMBL4411 | Prostaglandin E synthase 2 | Q9H7Z7 | SINGLE PROTEIN | Homo sapiens | 305 | 329 | 9606 | FALSE |
| CHEMBL1795194 | Phosphatidylinositol-5-phosphate 4-kinase type-2 alpha | P48426 | SINGLE PROTEIN | Homo sapiens | 1315 | 1636 | 9606 | FALSE |
| CHEMBL1287627 | Cytochrome b-245 heavy chain | P04839 | SINGLE PROTEIN | Homo sapiens | 141 | 238 | 9606 | FALSE |
| CHEMBL4630730 | Protein cereblon/Tyrosine-protein kinase receptor FLT3 | P36888|Q96SW2 | PROTEIN-PROTEIN INTERACTION | Homo sapiens | 7 | 32 | 9606 | FALSE |
| CHEMBL5072 | Ephrin type-B receptor 1 | P54762 | SINGLE PROTEIN | Homo sapiens | 584 | 884 | 9606 | FALSE |
| CHEMBL5465457 | LX-2 |  | CELL-LINE | Homo sapiens | 90 | 286 | 9606 | FALSE |
| CHEMBL3885518 | Aryl hydrocarbon receptor nuclear translocator/Endothelial PAS domain-containing protein 1 | P27540|Q99814 | PROTEIN-PROTEIN INTERACTION | Homo sapiens | 117 | 117 | 9606 | FALSE |
| CHEMBL4296046 | Gamma-aminobutyric acid receptor subunit alpha-4/beta-2/delta | P47870|P48169|O14764 | PROTEIN COMPLEX | Homo sapiens | 1 | 1 | 9606 | FALSE |
| CHEMBL3096 | Phosphoenolpyruvate carboxykinase mitochondrial | Q16822 | SINGLE PROTEIN | Homo sapiens | 1 | 1 | 9606 | FALSE |
| CHEMBL2111472 | Cytochrome P450 3A4/3A5 | P08684|P20815 | PROTEIN FAMILY | Homo sapiens | 78 | 100 | 9606 | FALSE |
| CHEMBL4295672 | Lysine-specific demethylase PHF2 | O75151 | SINGLE PROTEIN | Homo sapiens | 16 | 16 | 9606 | FALSE |
| CHEMBL2111431 | Epidermal growth factor receptor and ErbB2 (HER1 and HER2) | P04626|P00533 | PROTEIN FAMILY | Homo sapiens | 168 | 186 | 9606 | FALSE |
| CHEMBL5465394 | Integrin alpha1/beta2 complex | P05107|P56199 | PROTEIN COMPLEX | Homo sapiens | 10 | 18 | 9606 | FALSE |
| CHEMBL614058 | Caco-2 |  | CELL-LINE | Homo sapiens | 7494 | 12425 | 9606 | FALSE |
| CHEMBL2111341 | Dopamine D1 and D2 receptor | P14416|P21728 | SELECTIVITY GROUP | Homo sapiens | 183 | 197 | 9606 | FALSE |
| CHEMBL2189110 | Histone-lysine N-methyltransferase EZH2 | Q15910 | SINGLE PROTEIN | Homo sapiens | 1071 | 2179 | 9606 | FALSE |
| CHEMBL2008 | Retinoic acid receptor beta | P10826 | SINGLE PROTEIN | Homo sapiens | 642 | 1239 | 9606 | FALSE |
| CHEMBL2362996 | Voltage-gated potassium channel | P51787|Q9UK17|Q12809|P16389|Q09470|O43526|O43525|Q96RP8|Q9NR82|P56696|O95259|P22459|P22460|P22001|P17658|P48547|Q9NZV8|Q92953|Q03721|Q14003|Q14721|Q16322|Q6PIU1|Q8NCM2|Q8TAE7|Q8TDN1|Q8TDN2|Q96KK3|Q96L42|Q96PR1|Q9BQ31|Q9H252|Q9H3M0|Q9NS40|Q9NSA2|Q9UIX4|Q9UJ96|Q9ULD8|Q9ULS6|Q9UQ05 | PROTEIN FAMILY | Homo sapiens | 25 | 25 | 9606 | FALSE |
| CHEMBL3133 | Sodium/hydrogen exchanger 2 | Q9UBY0 | SINGLE PROTEIN | Homo sapiens | 17 | 37 | 9606 | FALSE |
| CHEMBL3885515 | Apoptosis regulator BAX/Induced myeloid leukemia cell differentiation protein Mcl-1 | Q07820|Q07812 | PROTEIN-PROTEIN INTERACTION | Homo sapiens | 4 | 7 | 9606 | FALSE |
| CHEMBL5813 | Tumor necrosis factor ligand superfamily member 10 | P50591 | SINGLE PROTEIN | Homo sapiens | 10 | 21 | 9606 | FALSE |
| CHEMBL3746 | 11-beta-hydroxysteroid dehydrogenase 2 | P80365 | SINGLE PROTEIN | Homo sapiens | 1032 | 1168 | 9606 | FALSE |
| CHEMBL4295690 | Alpha-2-macroglobulin | P01023 | SINGLE PROTEIN | Homo sapiens | 2 | 2 | 9606 | FALSE |
| CHEMBL4142 | Fibroblast growth factor receptor 2 | P21802 | SINGLE PROTEIN | Homo sapiens | 2517 | 3526 | 9606 | FALSE |
| CHEMBL2094253 | Cyclooxygenase | P35354|P23219 | PROTEIN FAMILY | Homo sapiens | 930 | 1259 | 9606 | FALSE |
| CHEMBL2111353 | Serine/threonine-protein kinase AKT | P31751|Q9Y243|P31749 | PROTEIN FAMILY | Homo sapiens | 172 | 294 | 9606 | FALSE |
| CHEMBL279 | Vascular endothelial growth factor receptor 2 | P35968 | SINGLE PROTEIN | Homo sapiens | 15111 | 21232 | 9606 | FALSE |
| CHEMBL3137267 | PKC alpha and beta-2 | P17252|P05771 | PROTEIN FAMILY | Homo sapiens | 3 | 4 | 9606 | FALSE |
| CHEMBL3712901 | Connective tissue growth factor | P29279 | SINGLE PROTEIN | Homo sapiens | 13 | 36 | 9606 | FALSE |
| CHEMBL2021748 | von Willebrand factor | P04275 | SINGLE PROTEIN | Homo sapiens | 11 | 34 | 9606 | FALSE |
| CHEMBL2044 | Gamma-amino-N-butyrate transaminase | P80404 | SINGLE PROTEIN | Homo sapiens | 101 | 155 | 9606 | FALSE |
| CHEMBL2007 | Platelet-derived growth factor receptor alpha | P16234 | SINGLE PROTEIN | Homo sapiens | 2416 | 5771 | 9606 | FALSE |
| CHEMBL614541 | CCRF-HSB-2 |  | CELL-LINE | Homo sapiens | 180 | 188 | 9606 | FALSE |
| CHEMBL5988 | Bcl-2-like protein 10 | Q9HD36 | SINGLE PROTEIN | Homo sapiens | 7 | 7 | 9606 | FALSE |
| CHEMBL2046267 | Anoctamin-1 | Q5XXA6 | SINGLE PROTEIN | Homo sapiens | 187 | 237 | 9606 | FALSE |
| CHEMBL2061 | Retinoid X receptor alpha | P19793 | SINGLE PROTEIN | Homo sapiens | 1704 | 3765 | 9606 | FALSE |
| CHEMBL2095169 | Dopamine receptors; D2 & D3 | P14416|P35462 | SELECTIVITY GROUP | Homo sapiens | 532 | 635 | 9606 | FALSE |
| CHEMBL3559703 | PI3-kinase class I | P27986|P42338|P48736|P42336|O00459|O00329|Q8WYR1|Q92569 | PROTEIN COMPLEX GROUP | Homo sapiens | 430 | 432 | 9606 | FALSE |
| CHEMBL4295646 | Mannan-binding lectin serine protease 2 | O00187 | SINGLE PROTEIN | Homo sapiens | 6 | 9 | 9606 | FALSE |
| CHEMBL2363062 | Janus Kinase (JAK) | P52333|P23458|O60674|P29597 | PROTEIN FAMILY | Homo sapiens | 151 | 190 | 9606 | FALSE |
| CHEMBL2094124 | Glutamate [NMDA] receptor | Q05586|Q12879|Q13224|O15399|O60391|Q14957|Q8TCU5 | PROTEIN COMPLEX GROUP | Homo sapiens | 671 | 939 | 9606 | FALSE |
| CHEMBL3883306 | MDM2/MDMX-p53 | P04637|Q00987|O15151 | PROTEIN-PROTEIN INTERACTION | Homo sapiens | 3 | 27 | 9606 | FALSE |
| CHEMBL3885505 | Matrix metalloproteinase 2/9 | P08253|P14780 | PROTEIN FAMILY | Homo sapiens | 2 | 2 | 9606 | FALSE |
| CHEMBL4106188 | Melanin-concentrating hormone receptor 2/HERG | Q12809|Q969V1 | SELECTIVITY GROUP | Homo sapiens | 14 | 14 | 9606 | FALSE |
| CHEMBL4630763 | Sodium channel protein type 5 subunit alpha/beta-1/beta-2 | Q14524|O60939|Q07699 | PROTEIN COMPLEX | Homo sapiens | 41 | 42 | 9606 | FALSE |
| CHEMBL3883310 | Axin-1/Low-density lipoprotein receptor-related protein 2 | O15169|P98164 | PROTEIN-PROTEIN INTERACTION | Homo sapiens | 1 | 1 | 9606 | FALSE |
| CHEMBL2004 | Retinoid X receptor gamma | P48443 | SINGLE PROTEIN | Homo sapiens | 422 | 718 | 9606 | FALSE |
| CHEMBL2095165 | Heat shock protein HSP90 | P07900|P08238 | PROTEIN FAMILY | Homo sapiens | 1817 | 3704 | 9606 | FALSE |
| CHEMBL2111392 | GABA A receptor alpha-1/beta-1/gamma-2 | P14867|P18507|P18505 | PROTEIN COMPLEX | Homo sapiens | 38 | 83 | 9606 | FALSE |
| CHEMBL3935 | Serine/threonine-protein kinase Aurora-C | Q9UQB9 | SINGLE PROTEIN | Homo sapiens | 1088 | 1822 | 9606 | FALSE |
| CHEMBL5465278 | Pachytene checkpoint protein 2 homolog | Q15645 | SINGLE PROTEIN | Homo sapiens | 6 | 6 | 9606 | FALSE |
| CHEMBL2095152 | Sulfonylurea receptors; K-ATP channels | Q14654|O60706|Q09428|Q15842 | PROTEIN COMPLEX GROUP | Homo sapiens | 325 | 596 | 9606 | FALSE |
| CHEMBL2095217 | Fibroblast growth factor receptor | P22607|P11362|P22455|P21802 | PROTEIN FAMILY | Homo sapiens | 276 | 356 | 9606 | FALSE |
| CHEMBL4295685 | Sodium-dependent phosphate transport protein 2B | O95436 | SINGLE PROTEIN | Homo sapiens | 100 | 105 | 9606 | FALSE |
| CHEMBL2111480 | Vascular endothelial growth factor receptor 1 and 2 (Flt-1 and KDR) | P17948|P35968 | SELECTIVITY GROUP | Homo sapiens | 27 | 27 | 9606 | FALSE |
| CHEMBL2189121 | GTPase KRas | P01116 | SINGLE PROTEIN | Homo sapiens | 888 | 2145 | 9606 | FALSE |
| CHEMBL2096619 | Phosphatidylinositol 4-kinase, PI4K | Q9BTU6|Q8TCG2|Q9UBF8|P42356 | PROTEIN FAMILY | Homo sapiens | 93 | 160 | 9606 | FALSE |
| CHEMBL1778 | Interleukin-2 receptor alpha chain | P01589 | SINGLE PROTEIN | Homo sapiens | 1 | 2 | 9606 | FALSE |
| CHEMBL614263 | ACH-2 cell line |  | CELL-LINE | Homo sapiens | 3 | 10 | 9606 | FALSE |
| CHEMBL5465459 | CRMM-2 |  | CELL-LINE | Homo sapiens | 45 | 59 | 9606 | FALSE |
| CHEMBL3886061 | Nucleosome assembly protein 1-like 4 | Q99733 | SINGLE PROTEIN | Homo sapiens | 5 | 5 | 9606 | FALSE |
| CHEMBL3638346 | Methyl-CpG-binding protein 2 | P51608 | SINGLE PROTEIN | Homo sapiens | 12 | 12 | 9606 | FALSE |
| CHEMBL5465357 | mRNA (2'-O-methyladenosine-N(6)-)-methyltransferase | Q9H4Z3 | SINGLE PROTEIN | Homo sapiens | 4 | 4 | 9606 | FALSE |
| CHEMBL5483141 | Cell cycle and apoptosis regulator protein 2 | Q8N163 | SINGLE PROTEIN | Homo sapiens | 1 | 1 | 9606 | FALSE |
| CHEMBL1795 | Atrial natriuretic peptide receptor B | P20594 | SINGLE PROTEIN | Homo sapiens | 11 | 11 | 9606 | FALSE |
| CHEMBL1917 | Somatostatin receptor 1 | P30872 | SINGLE PROTEIN | Homo sapiens | 752 | 861 | 9606 | FALSE |
| CHEMBL5517 | Secreted frizzled-related protein 1 | Q8N474 | SINGLE PROTEIN | Homo sapiens | 111 | 243 | 9606 | FALSE |
| CHEMBL2424504 | Lysine-specific demethylase 5A | P29375 | SINGLE PROTEIN | Homo sapiens | 658 | 903 | 9606 | FALSE |
| CHEMBL4523719 | BRD4/E3 ubiquitin-protein ligase Mdm2 | Q00987|O60885 | PROTEIN-PROTEIN INTERACTION | Homo sapiens | 3 | 9 | 9606 | FALSE |
| CHEMBL3883323 | Cyclin-C/Cyclin-dependent kinase 19 | Q9BWU1|P24863 | PROTEIN COMPLEX | Homo sapiens | 309 | 334 | 9606 | FALSE |
| CHEMBL5291949 | Gamma-aminobutyric acid receptor subunit alpha-2/beta-1/gamma-2 | P18507|P18505|P47869 | PROTEIN COMPLEX | Homo sapiens | 2 | 2 | 9606 | FALSE |
| CHEMBL3259470 | G-protein coupled receptor 183 | P32249 | SINGLE PROTEIN | Homo sapiens | 207 | 257 | 9606 | FALSE |
| CHEMBL3559692 | Cyclin-dependent kinase 12 | Q9NYV4 | SINGLE PROTEIN | Homo sapiens | 356 | 465 | 9606 | FALSE |
| CHEMBL2109 | Dual specificity mitogen-activated protein kinase kinase 3 | P46734 | SINGLE PROTEIN | Homo sapiens | 531 | 682 | 9606 | FALSE |
| CHEMBL4105769 | CCR4-NOT transcription complex subunit 3 | O75175 | SINGLE PROTEIN | Homo sapiens | 3 | 3 | 9606 | FALSE |
| CHEMBL5465253 | HAT1/Rbap46 | Q16576|O14929 | PROTEIN COMPLEX | Homo sapiens | 49 | 59 | 9606 | FALSE |
| CHEMBL5465213 | Cereblon/Tyrosine-protein kinase ITK/TSK | Q08881|Q96SW2 | PROTEIN-PROTEIN INTERACTION | Homo sapiens | 20 | 52 | 9606 | FALSE |
| CHEMBL5548 | Prokineticin receptor 2 | Q8NFJ6 | SINGLE PROTEIN | Homo sapiens | 4 | 9 | 9606 | FALSE |
| CHEMBL1961787 | Nuclear receptor subfamily 2 group C member 1 | P13056 | SINGLE PROTEIN | Homo sapiens | 3 | 5 | 9606 | FALSE |
| CHEMBL4524043 | Mucolipin-1 | Q9GZU1 | SINGLE PROTEIN | Homo sapiens | 19 | 56 | 9606 | FALSE |
| CHEMBL3980 | Serine/threonine-protein kinase 17B | O94768 | SINGLE PROTEIN | Homo sapiens | 562 | 807 | 9606 | FALSE |
| CHEMBL1938223 | TP53-regulating kinase | Q96S44 | SINGLE PROTEIN | Homo sapiens | 273 | 284 | 9606 | FALSE |
| CHEMBL2311243 | D-3-phosphoglycerate dehydrogenase | O43175 | SINGLE PROTEIN | Homo sapiens | 551 | 967 | 9606 | FALSE |
| CHEMBL1961784 | Nuclear receptor subfamily 1 group D member 2 | Q14995 | SINGLE PROTEIN | Homo sapiens | 8 | 17 | 9606 | FALSE |
| CHEMBL4483229 | BE(2)-C |  | CELL-LINE | Homo sapiens | 155 | 265 | 9606 | FALSE |
| CHEMBL5465214 | Cereblon/Histone acetyltransferase p300 | Q09472|Q96SW2 | PROTEIN-PROTEIN INTERACTION | Homo sapiens | 9 | 10 | 9606 | FALSE |
| CHEMBL1075417 | Capan-2 |  | CELL-LINE | Homo sapiens | 261 | 287 | 9606 | FALSE |
| CHEMBL3430903 | DNA polymerase subunit gamma-1/gamma-2 | P54098|Q9UHN1 | PROTEIN FAMILY | Homo sapiens | 16 | 16 | 9606 | FALSE |
| CHEMBL5914 | Mitogen-activated protein kinase kinase kinase 2 | Q9Y2U5 | SINGLE PROTEIN | Homo sapiens | 521 | 639 | 9606 | FALSE |
| CHEMBL2519 | Neutral alpha-glucosidase AB | Q14697 | SINGLE PROTEIN | Homo sapiens | 28 | 90 | 9606 | FALSE |
| CHEMBL3885521 | Baculoviral IAP repeat-containing protein 2/Estrogen receptor | P03372|Q13490 | PROTEIN COMPLEX | Homo sapiens | 4 | 7 | 9606 | FALSE |
| CHEMBL2189135 | Acyl-coenzyme A thioesterase 2, mitochondrial | P49753 | SINGLE PROTEIN | Homo sapiens | 1 | 1 | 9606 | FALSE |
| CHEMBL1909487 | Protein-arginine deiminase type-2 | Q9Y2J8 | SINGLE PROTEIN | Homo sapiens | 80 | 139 | 9606 | FALSE |
| CHEMBL3091268 | Nuclear receptor ROR-beta | Q92753 | SINGLE PROTEIN | Homo sapiens | 459 | 607 | 9606 | FALSE |
| CHEMBL2789 | Estradiol 17-beta-dehydrogenase 2 | P37059 | SINGLE PROTEIN | Homo sapiens | 1317 | 1680 | 9606 | FALSE |
| CHEMBL3883300 | CREB-binding protein/Histone acetyltransferase p300 | Q09472|Q92793 | PROTEIN FAMILY | Homo sapiens | 286 | 392 | 9606 | FALSE |
| CHEMBL3038502 | JNK2/JNK3 | P53779|P45984 | PROTEIN FAMILY | Homo sapiens | 1 | 1 | 9606 | FALSE |
| CHEMBL2096989 | Angiotensin-converting enzyme | P12821|Q9BYF1 | PROTEIN FAMILY | Homo sapiens | 61 | 76 | 9606 | FALSE |
| CHEMBL3176 | Galanin receptor 2 | O43603 | SINGLE PROTEIN | Homo sapiens | 202 | 361 | 9606 | FALSE |
| CHEMBL5465266 | Fermitin family homolog 2 | Q96AC1 | SINGLE PROTEIN | Homo sapiens | 26 | 48 | 9606 | FALSE |
| CHEMBL3108639 | Cat eye syndrome critical region protein 2 | Q9BXF3 | SINGLE PROTEIN | Homo sapiens | 249 | 356 | 9606 | FALSE |
| CHEMBL3761 | Beta-glucosidase | Q9HCG7 | SINGLE PROTEIN | Homo sapiens | 202 | 258 | 9606 | FALSE |
| CHEMBL1932891 | Acyl-protein thioesterase 2 | O95372 | SINGLE PROTEIN | Homo sapiens | 12 | 25 | 9606 | FALSE |
| CHEMBL4469 | Small conductance calcium-activated potassium channel protein 2 | Q9H2S1 | SINGLE PROTEIN | Homo sapiens | 28 | 31 | 9606 | FALSE |
| CHEMBL1075104 | Leucine-rich repeat serine/threonine-protein kinase 2 | Q5S007 | SINGLE PROTEIN | Homo sapiens | 3215 | 6728 | 9606 | FALSE |
| CHEMBL3038483 | Histone deacetylase 1/3/5/8 | Q13547|O15379|Q9UQL6|Q9BY41 | PROTEIN FAMILY | Homo sapiens | 46 | 49 | 9606 | FALSE |
| CHEMBL3724 | Lysophosphatidic acid receptor Edg-4 | Q9HBW0 | SINGLE PROTEIN | Homo sapiens | 287 | 482 | 9606 | FALSE |
| CHEMBL1795192 | Cyclin-dependent kinase 13 | Q14004 | SINGLE PROTEIN | Homo sapiens | 491 | 610 | 9606 | FALSE |
| CHEMBL3959 | Quinone reductase 2 | P16083 | SINGLE PROTEIN | Homo sapiens | 655 | 885 | 9606 | FALSE |
| CHEMBL5169265 | BCL2/BID | P10415|P55957 | PROTEIN-PROTEIN INTERACTION | Homo sapiens | 18 | 18 | 9606 | FALSE |
| CHEMBL5095 | Wee1-like protein kinase 2 | P0C1S8 | SINGLE PROTEIN | Homo sapiens | 256 | 283 | 9606 | FALSE |
| CHEMBL4105949 | Peptidyl-prolyl cis-trans isomerase FKBP2 | P26885 | SINGLE PROTEIN | Homo sapiens | 2 | 2 | 9606 | FALSE |
| CHEMBL5465328 | Ribosomal oxygenase 2 | Q8IUF8 | SINGLE PROTEIN | Homo sapiens | 10 | 10 | 9606 | FALSE |
| CHEMBL3137287 | EZH1/SUZ12/EED/AEBP2/RBBP4 complex | Q92800|O75530|Q15022|Q6ZN18|Q09028 | PROTEIN COMPLEX | Homo sapiens | 29 | 29 | 9606 | FALSE |
| CHEMBL3885513 | Apoptosis regulator BAX/Bcl-2 | P10415|Q07812 | PROTEIN-PROTEIN INTERACTION | Homo sapiens | 1 | 1 | 9606 | FALSE |
| CHEMBL3632458 | S-phase kinase-associated protein 2 | Q13309 | SINGLE PROTEIN | Homo sapiens | 2 | 9 | 9606 | FALSE |
| CHEMBL4296075 | Glycine receptor subunit alpha-2/beta | P23416|P48167 | PROTEIN COMPLEX | Homo sapiens | 1 | 1 | 9606 | FALSE |
| CHEMBL2093862 | Runt-related transcription factor 1/Core-binding factor subunit beta | Q01196|Q13951 | PROTEIN-PROTEIN INTERACTION | Homo sapiens | 7454 | 7867 | 9606 | FALSE |
| CHEMBL2525 | Beta secretase 2 | Q9Y5Z0 | SINGLE PROTEIN | Homo sapiens | 1566 | 1716 | 9606 | FALSE |
| CHEMBL2189126 | Calcium-independent phospholipase A2-gamma | Q9NP80 | SINGLE PROTEIN | Homo sapiens | 1 | 1 | 9606 | FALSE |
| CHEMBL3308911 | Early activation antigen CD69 | Q07108 | SINGLE PROTEIN | Homo sapiens | 61 | 107 | 9606 | FALSE |
| CHEMBL3883291 | Glutamate receptor AMPA 2/3 | P42263|P42262 | PROTEIN COMPLEX | Homo sapiens | 6 | 11 | 9606 | FALSE |
| CHEMBL4523664 | HSPA2/STIP1 | P54652|P31948 | PROTEIN-PROTEIN INTERACTION | Homo sapiens | 15 | 35 | 9606 | FALSE |
| CHEMBL3885623 | Prostaglandin E synthase/G/H synthase 2 | P35354|O14684 | PROTEIN FAMILY | Homo sapiens | 4 | 7 | 9606 | FALSE |
| CHEMBL2029198 | Rap guanine nucleotide exchange factor 4 | Q8WZA2 | SINGLE PROTEIN | Homo sapiens | 10782 | 11476 | 9606 | FALSE |
| CHEMBL1075166 | Beta-adrenergic receptor kinase 2 | P35626 | SINGLE PROTEIN | Homo sapiens | 240 | 271 | 9606 | FALSE |
| CHEMBL5305043 | Estradiol 17-beta-dehydrogenase 11 | Q8NBQ5 | SINGLE PROTEIN | Homo sapiens | 3 | 4 | 9606 | FALSE |
| CHEMBL3039 | NADH-ubiquinone oxidoreductase 49 kDa subunit | O75306 | SINGLE PROTEIN | Homo sapiens | 2 | 6 | 9606 | FALSE |
| CHEMBL4523193 | General transcription and DNA repair factor IIH helicase subunit XPB | P19447 | SINGLE PROTEIN | Homo sapiens | 2 | 2 | 9606 | FALSE |
| CHEMBL4523605 | C5a anaphylatoxin chemotactic receptor 1/2 | P21730|Q9P296 | PROTEIN COMPLEX | Homo sapiens | 2 | 2 | 9606 | FALSE |
| CHEMBL1741184 | ETS domain-containing protein Elk-3 | P41970 | SINGLE PROTEIN | Homo sapiens | 1 | 1 | 9606 | FALSE |
| CHEMBL3713687 | Cytochrome P450 26B1 | Q9NR63 | SINGLE PROTEIN | Homo sapiens | 24 | 25 | 9606 | FALSE |
| CHEMBL3038511 | TCF4/beta-catenin | P35222|Q9NQB0 | PROTEIN COMPLEX | Homo sapiens | 334 | 616 | 9606 | FALSE |
| CHEMBL1994 | Mineralocorticoid receptor | P08235 | SINGLE PROTEIN | Homo sapiens | 1434 | 2137 | 9606 | FALSE |
| CHEMBL3474 | Phospholipase A2 group IIA | P14555 | SINGLE PROTEIN | Homo sapiens | 624 | 1079 | 9606 | FALSE |
| CHEMBL5469 | Protein tyrosine kinase 2 beta | Q14289 | SINGLE PROTEIN | Homo sapiens | 2104 | 2895 | 9606 | FALSE |
| CHEMBL4899 | Mitogen-activated protein kinase kinase kinase 8 | P41279 | SINGLE PROTEIN | Homo sapiens | 403 | 469 | 9606 | FALSE |
| CHEMBL2362979 | Probable global transcription activator SNF2L2 | P51531 | SINGLE PROTEIN | Homo sapiens | 412 | 503 | 9606 | FALSE |
| CHEMBL2094115 | MAP kinase p38 | O15264|Q15759|Q16539|P53778 | PROTEIN FAMILY | Homo sapiens | 1047 | 1594 | 9606 | FALSE |
| CHEMBL5465270 | Sphingosine-1-phosphate transporter SPNS2 | Q8IVW8 | SINGLE PROTEIN | Homo sapiens | 17 | 19 | 9606 | FALSE |
| CHEMBL2096988 | Thrombin & trypsin | P00734|P07477|P07478|P35030 | SELECTIVITY GROUP | Homo sapiens | 257 | 271 | 9606 | FALSE |
| CHEMBL4739693 | Potassium channel subfamily T member 2 | Q6UVM3 | SINGLE PROTEIN | Homo sapiens | 17 | 32 | 9606 | FALSE |
| CHEMBL5291561 | Interleukin-36 gamma | Q9NZH8 | SINGLE PROTEIN | Homo sapiens | 1 | 1 | 9606 | FALSE |
| CHEMBL3769300 | Sodium-dependent phosphate transport protein 3 | O00624 | SINGLE PROTEIN | Homo sapiens | 1 | 1 | 9606 | FALSE |
| CHEMBL4680047 | Baculoviral IAP repeat-containing protein 2/Hematopoietic prostaglandin D synthase | Q13490|O60760 | PROTEIN-PROTEIN INTERACTION | Homo sapiens | 1 | 1 | 9606 | FALSE |
| CHEMBL5465301 | Paired box protein Pax-2 | Q02962 | SINGLE PROTEIN | Homo sapiens | 1 | 1 | 9606 | FALSE |
| CHEMBL1744524 | Sodium/myo-inositol cotransporter 2 | Q8WWX8 | SINGLE PROTEIN | Homo sapiens | 4 | 4 | 9606 | FALSE |
| CHEMBL5291951 | Dual specificity protein kinase CLK1/CLK2/CLK4 | Q9HAZ1|P49759|P49760 | PROTEIN FAMILY | Homo sapiens | 1 | 1 | 9606 | FALSE |
| CHEMBL4739852 | Forkhead box protein M1 | Q08050 | SINGLE PROTEIN | Homo sapiens | 25 | 77 | 9606 | FALSE |
| CHEMBL2899 | Brain adenylate cyclase 1 | Q08828 | SINGLE PROTEIN | Homo sapiens | 212 | 399 | 9606 | FALSE |
| CHEMBL2094122 | GABA-A receptor; alpha-5/beta-3/gamma-2 | P18507|P28472|P31644 | PROTEIN COMPLEX | Homo sapiens | 799 | 1334 | 9606 | FALSE |
| CHEMBL3883298 | Inhibitor of NF-kappa-B kinase alpha/beta | O14920|O15111 | PROTEIN COMPLEX | Homo sapiens | 17 | 19 | 9606 | FALSE |
| CHEMBL4742293 | Cereblon/Apoptosis-stimulating of p53 protein 2 | Q96SW2|Q13625 | PROTEIN-PROTEIN INTERACTION | Homo sapiens | 1 | 1 | 9606 | FALSE |
| CHEMBL3817720 | 1,2-dihydroxy-3-keto-5-methylthiopentene dioxygenase | Q9BV57 | SINGLE PROTEIN | Homo sapiens | 4 | 4 | 9606 | FALSE |
| CHEMBL4105932 | Serine/threonine-protein kinase/endoribonuclease IRE2 | Q76MJ5 | SINGLE PROTEIN | Homo sapiens | 255 | 257 | 9606 | FALSE |
| CHEMBL5465381 | MUS81-ECE2 | P0DPD6|Q96NY9 | PROTEIN COMPLEX | Homo sapiens | 2 | 10 | 9606 | FALSE |
| CHEMBL3751654 | 5'(3')-deoxyribonucleotidase, mitochondrial | Q9NPB1 | SINGLE PROTEIN | Homo sapiens | 2 | 2 | 9606 | FALSE |
| CHEMBL4105858 | Putative heat shock protein HSP 90-beta 2 | Q58FF8 | SINGLE PROTEIN | Homo sapiens | 233 | 233 | 9606 | FALSE |
| CHEMBL5465215 | Cereblon/Baculoviral IAP repeat-containing protein 3 | Q13489|Q96SW2 | PROTEIN-PROTEIN INTERACTION | Homo sapiens | 21 | 45 | 9606 | FALSE |
| CHEMBL3885613 | Nuclear receptor subfamily 4 group A member 2/Retinoic acid receptor RXR-alpha | P19793|P43354 | PROTEIN COMPLEX | Homo sapiens | 8 | 14 | 9606 | FALSE |
| CHEMBL4680036 | Terminal nucleotidyltransferase 4B | Q8NDF8 | SINGLE PROTEIN | Homo sapiens | 3 | 3 | 9606 | FALSE |
| CHEMBL4523916 | Probable G-protein coupled receptor 63 | Q9BZJ6 | SINGLE PROTEIN | Homo sapiens | 1 | 1 | 9606 | FALSE |
| CHEMBL2366286 | MHH-CALL-2 |  | CELL-LINE | Homo sapiens | 77 | 78 | 9606 | FALSE |
| CHEMBL4523754 | VHL/Cullin-2/Estrogen receptor alpha | P03372|P40337|Q13617 | PROTEIN-PROTEIN INTERACTION | Homo sapiens | 37 | 138 | 9606 | FALSE |
| CHEMBL1250344 | Serine palmitoyltransferase 2 | O15270 | SINGLE PROTEIN | Homo sapiens | 14 | 14 | 9606 | FALSE |
| CHEMBL4295811 | Interleukin enhancer-binding factor 2 | Q12905 | SINGLE PROTEIN | Homo sapiens | 1 | 1 | 9606 | FALSE |
| CHEMBL5291557 | Cyclic AMP-dependent transcription factor ATF-2 | P15336 | SINGLE PROTEIN | Homo sapiens | 1 | 4 | 9606 | FALSE |
| CHEMBL2612 | Valyl-tRNA synthetase 2 | P26640 | SINGLE PROTEIN | Homo sapiens | 11 | 11 | 9606 | FALSE |
| CHEMBL3271 | Sodium-dependent vitamin C transporter 2 | Q9UGH3 | SINGLE PROTEIN | Homo sapiens | 6 | 28 | 9606 | FALSE |
| CHEMBL4879445 | Gamma-aminobutyric acid receptor-associated protein-like 2 | P60520 | SINGLE PROTEIN | Homo sapiens | 1 | 1 | 9606 | FALSE |
| CHEMBL5291687 | Cereblon/RAC-gamma serine/threonine-protein kinase | Q9Y243|Q96SW2 | PROTEIN-PROTEIN INTERACTION | Homo sapiens | 1 | 1 | 9606 | FALSE |
| CHEMBL3038470 | CDK2/Cyclin A1 | P24941|P78396 | PROTEIN COMPLEX | Homo sapiens | 184 | 201 | 9606 | FALSE |
| CHEMBL3508692 | Docosahexaenoic acid omega-hydroxylase CYP4F3 | Q08477 | SINGLE PROTEIN | Homo sapiens | 35 | 58 | 9606 | FALSE |
| CHEMBL5404 | Serine/threonine-protein kinase tousled-like 2 | Q86UE8 | SINGLE PROTEIN | Homo sapiens | 499 | 752 | 9606 | FALSE |
| CHEMBL1250342 | Deoxyribonuclease-2-alpha | O00115 | SINGLE PROTEIN | Homo sapiens | 1 | 1 | 9606 | FALSE |
| CHEMBL3038480 | Ionotropic glutamate receptor kainate 2/5 | Q16478|Q13002 | PROTEIN COMPLEX | Homo sapiens | 2 | 2 | 9606 | FALSE |
| CHEMBL5477 | Proteinase-activated receptor 3 | O00254 | SINGLE PROTEIN | Homo sapiens | 4 | 4 | 9606 | FALSE |
| CHEMBL614826 | HMV-2 cell line |  | CELL-LINE | Homo sapiens | 97 | 109 | 9606 | FALSE |
| CHEMBL5483189 | CDK1/Cyclin E | P06493|P24864|O96020 | PROTEIN COMPLEX GROUP | Homo sapiens | 1 | 1 | 9606 | FALSE |
| CHEMBL3038466 | Calpain1/2 | P17655|P07384 | PROTEIN FAMILY | Homo sapiens | 9 | 15 | 9606 | FALSE |
| CHEMBL3831281 | P2X2/P2X3 heterotrimeric receptor | Q9UBL9|P56373 | PROTEIN COMPLEX | Homo sapiens | 510 | 633 | 9606 | FALSE |
| CHEMBL3032 | Protein kinase N2 | Q16513 | SINGLE PROTEIN | Homo sapiens | 1568 | 2037 | 9606 | FALSE |
| CHEMBL3924 | Dual specificity phosphatase 22 | Q9NRW4 | SINGLE PROTEIN | Homo sapiens | 79 | 89 | 9606 | FALSE |
| CHEMBL1293258 | Mothers against decapentaplegic homolog 3 | P84022 | SINGLE PROTEIN | Homo sapiens | 64081 | 68039 | 9606 | FALSE |
| CHEMBL2375201 | Tyrosine-protein kinase transmembrane receptor ROR2 | Q01974 | SINGLE PROTEIN | Homo sapiens | 5 | 5 | 9606 | FALSE |
| CHEMBL2111434 | VEGF-receptor 2 and Fibroblast growth factor receptor 2 | P35968|P21802 | SELECTIVITY GROUP | Homo sapiens | 15 | 15 | 9606 | FALSE |
| CHEMBL2401 | Sarcoplasmic/endoplasmic reticulum calcium ATPase 3 | Q93084 | SINGLE PROTEIN | Homo sapiens | 5 | 6 | 9606 | FALSE |
| CHEMBL3709670 | ADP/ATP translocase 2 | P05141 | SINGLE PROTEIN | Homo sapiens | 246 | 249 | 9606 | FALSE |
| CHEMBL2096906 | Carbonic anhydrases; II & IX | P00918|Q16790 | SELECTIVITY GROUP | Homo sapiens | 207 | 262 | 9606 | FALSE |
| CHEMBL3313835 | S-adenosylmethionine synthase isoform type-2 | P31153 | SINGLE PROTEIN | Homo sapiens | 667 | 805 | 9606 | FALSE |
| CHEMBL3137286 | EZH2/SUZ12/EED complex | Q15910|O75530|Q15022 | PROTEIN COMPLEX | Homo sapiens | 12 | 20 | 9606 | FALSE |
| CHEMBL2189127 | Monoacylglycerol lipase ABHD6 | Q9BV23 | SINGLE PROTEIN | Homo sapiens | 249 | 331 | 9606 | FALSE |
| CHEMBL5516 | Monoacylglycerol lipase ABHD12 | Q8N2K0 | SINGLE PROTEIN | Homo sapiens | 151 | 199 | 9606 | FALSE |
| CHEMBL3708265 | 7,8-dihydro-8-oxoguanine triphosphatase | P36639 | SINGLE PROTEIN | Homo sapiens | 164 | 282 | 9606 | FALSE |
| CHEMBL2364708 | Calcium-activated chloride channel | Q9Y6N3|A8K7I4|Q9UQC9|Q14CN2 | PROTEIN FAMILY | Homo sapiens | 1 | 1 | 9606 | FALSE |
| CHEMBL4426 | Phospholipase A2 group 1B | P04054 | SINGLE PROTEIN | Homo sapiens | 615 | 720 | 9606 | FALSE |
| CHEMBL4742263 | Cereblon/Tyrosine-protein kinase JAK2 | O60674|Q96SW2 | PROTEIN-PROTEIN INTERACTION | Homo sapiens | 22 | 30 | 9606 | FALSE |
| CHEMBL3430884 | Sphingosine 1-phosphate receptor Edg-5/Sphingosine 1-phosphate receptor Edg-3 | O95136|Q99500 | PROTEIN COMPLEX | Homo sapiens | 2 | 2 | 9606 | FALSE |
| CHEMBL2073704 | Monocarboxylate transporter 2 | O60669 | SINGLE PROTEIN | Homo sapiens | 10 | 11 | 9606 | FALSE |
| CHEMBL1961785 | Receptor-type tyrosine-protein phosphatase U | Q92729 | SINGLE PROTEIN | Homo sapiens | 2 | 2 | 9606 | FALSE |
| CHEMBL3038490 | Kir3.2/Kir3.3 | P48051|Q92806 | PROTEIN COMPLEX | Homo sapiens | 14 | 15 | 9606 | FALSE |
| CHEMBL3038468 | CDK1/Cyclin E | P06493|P24864 | PROTEIN COMPLEX | Homo sapiens | 39 | 49 | 9606 | FALSE |
| CHEMBL3351218 | 2'-deoxynucleoside 5'-phosphate N-hydrolase 1 | O43598 | SINGLE PROTEIN | Homo sapiens | 25 | 25 | 9606 | FALSE |
| CHEMBL2955 | Sphingosine 1-phosphate receptor Edg-5 | O95136 | SINGLE PROTEIN | Homo sapiens | 978 | 1597 | 9606 | FALSE |
| CHEMBL4105960 | Serine/threonine-protein phosphatase 6 regulatory subunit 2 | O75170 | SINGLE PROTEIN | Homo sapiens | 2 | 2 | 9606 | FALSE |
| CHEMBL2169736 | Tyrosyl-DNA phosphodiesterase 2 | O95551 | SINGLE PROTEIN | Homo sapiens | 624 | 864 | 9606 | FALSE |
| CHEMBL3430878 | Retinoic acid receptor RXR-alpha/oxysterols receptor LXR-beta | P19793|P55055 | PROTEIN COMPLEX | Homo sapiens | 68 | 97 | 9606 | FALSE |
| CHEMBL5169267 | BCL2L1/BAD | Q92934|Q07817 | PROTEIN-PROTEIN INTERACTION | Homo sapiens | 18 | 18 | 9606 | FALSE |
| CHEMBL613852 | CAKI-2 |  | CELL-LINE | Homo sapiens | 28 | 29 | 9606 | FALSE |
| CHEMBL4105990 | Ubiquitin-conjugating enzyme E2 D2 | P62837 | SINGLE PROTEIN | Homo sapiens | 6 | 8 | 9606 | FALSE |
| CHEMBL3879825 | tRNA-dihydrouridine(20) synthase [NAD(P)+]-like | Q9NX74 | SINGLE PROTEIN | Homo sapiens | 1 | 1 | 9606 | FALSE |
| CHEMBL4295780 | 39S ribosomal protein L12, mitochondrial | P52815 | SINGLE PROTEIN | Homo sapiens | 1 | 1 | 9606 | FALSE |
| CHEMBL1893 | Synaptic vesicular amine transporter | Q05940 | SINGLE PROTEIN | Homo sapiens | 38 | 118 | 9606 | FALSE |
| CHEMBL4296080 | Heat shock protein HSP 90-alpha/Hsp90 co-chaperone Cdc37 | P07900|Q16543 | PROTEIN COMPLEX | Homo sapiens | 1 | 1 | 9606 | FALSE |
| CHEMBL4487 | Serine/threonine-protein kinase PAK 2 | Q13177 | SINGLE PROTEIN | Homo sapiens | 1262 | 1983 | 9606 | FALSE |
| CHEMBL5120 | Non-secretory ribonuclease | P10153 | SINGLE PROTEIN | Homo sapiens | 2 | 2 | 9606 | FALSE |
| CHEMBL4524009 | Sigma receptor | Q99720|Q5BJF2 | PROTEIN FAMILY | Homo sapiens | 7 | 8 | 9606 | FALSE |
| CHEMBL4523963 | CDK4/CDK1 | P06493|P11802 | SELECTIVITY GROUP | Homo sapiens | 29 | 29 | 9606 | FALSE |
| CHEMBL4295815 | Cytoplasmic dynein 1 intermediate chain 2 | Q13409 | SINGLE PROTEIN | Homo sapiens | 1 | 1 | 9606 | FALSE |
| CHEMBL1075481 | KOSC-2 |  | CELL-LINE | Homo sapiens | 98 | 99 | 9606 | FALSE |
| CHEMBL4523275 | Antigen peptide transporter 1 | Q03518 | SINGLE PROTEIN | Homo sapiens | 1 | 1 | 9606 | FALSE |
| CHEMBL4296326 | Anthrax toxin receptor 2 | P58335 | SINGLE PROTEIN | Homo sapiens | 34 | 35 | 9606 | FALSE |
| CHEMBL4523456 | 5-azacytidine-induced protein 2 | Q9H6S1 | SINGLE PROTEIN | Homo sapiens | 1 | 1 | 9606 | FALSE |
| CHEMBL4105714 | Serine/threonine-protein phosphatase 4 regulatory subunit 3B | Q5MIZ7 | SINGLE PROTEIN | Homo sapiens | 1 | 1 | 9606 | FALSE |
| CHEMBL4523399 | CDGSH iron-sulfur domain-containing protein 2 | Q8N5K1 | SINGLE PROTEIN | Homo sapiens | 3 | 10 | 9606 | FALSE |
| CHEMBL3885556 | Cyclin-dependent kinase 8/19 | P49336|Q9BWU1 | PROTEIN FAMILY | Homo sapiens | 71 | 99 | 9606 | FALSE |
| CHEMBL2403 | Thioredoxin reductase 2 | Q9NNW7 | SINGLE PROTEIN | Homo sapiens | 2 | 5 | 9606 | FALSE |
| CHEMBL4295727 | Inter-alpha-trypsin inhibitor heavy chain H2 | P19823 | SINGLE PROTEIN | Homo sapiens | 1 | 2 | 9606 | FALSE |
| CHEMBL4105833 | Signal peptide peptidase-like 2A | Q8TCT8 | SINGLE PROTEIN | Homo sapiens | 34 | 36 | 9606 | FALSE |
| CHEMBL2345 | Ribosomal protein S6 kinase alpha 3 | P51812 | SINGLE PROTEIN | Homo sapiens | 2871 | 4397 | 9606 | FALSE |
| CHEMBL2111460 | Dopamine D2 receptor and serotonin 1a receptor | P14416|P08908 | SELECTIVITY GROUP | Homo sapiens | 10 | 10 | 9606 | FALSE |
| CHEMBL3525 | Integrin alpha-3 | P26006 | SINGLE PROTEIN | Homo sapiens | 8 | 12 | 9606 | FALSE |
| CHEMBL4106180 | p38 MAP kinase alpha/beta | Q15759|Q16539 | PROTEIN FAMILY | Homo sapiens | 1 | 2 | 9606 | FALSE |
| CHEMBL3043 | Tyrosine aminotransferase | P17735 | SINGLE PROTEIN | Homo sapiens | 13 | 14 | 9606 | FALSE |
| CHEMBL2111401 | Thrombin and coagulation factor X | P00734|P00742 | SELECTIVITY GROUP | Homo sapiens | 140 | 145 | 9606 | FALSE |
| CHEMBL4523988 | Class 1 histone deacetylase | Q13547|O15379|Q92769|Q9BY41 | PROTEIN FAMILY | Homo sapiens | 184 | 515 | 9606 | FALSE |
| CHEMBL3885624 | PAC1-PAC2 complex | O95456|Q969U7 | PROTEIN COMPLEX | Homo sapiens | 17 | 49 | 9606 | FALSE |
| CHEMBL4523251 | Taste receptor type 2 member 43 | P59537 | SINGLE PROTEIN | Homo sapiens | 11 | 15 | 9606 | FALSE |
| CHEMBL6097 | Fatty acid desaturase 2 | O95864 | SINGLE PROTEIN | Homo sapiens | 100 | 102 | 9606 | FALSE |
| CHEMBL4523632 | CDK9/Cyclin-H | P51946|P50750 | PROTEIN COMPLEX | Homo sapiens | 1 | 1 | 9606 | FALSE |
| CHEMBL5358 | Eukaryotic translation initiation factor 2-alpha kinase 4 | Q9P2K8 | SINGLE PROTEIN | Homo sapiens | 399 | 515 | 9606 | FALSE |
| CHEMBL3430908 | Aurora kinase B/Receptor-type tyrosine-protein kinase | P36888|Q96GD4 | SELECTIVITY GROUP | Homo sapiens | 2 | 2 | 9606 | FALSE |
| CHEMBL4523364 | Hepatoma-derived growth factor-related protein 2 | Q7Z4V5 | SINGLE PROTEIN | Homo sapiens | 1 | 1 | 9606 | FALSE |
| CHEMBL5482995 | NRP2-VEGA | P15692|O60462 | PROTEIN COMPLEX | Homo sapiens | 2 | 4 | 9606 | FALSE |
| CHEMBL3826864 | Myelin P2 protein | P02689 | SINGLE PROTEIN | Homo sapiens | 1 | 1 | 9606 | FALSE |
| CHEMBL3421525 | 6-phosphofructo-2-kinase/fructose-2,6-bisphosphatase 2 | O60825 | SINGLE PROTEIN | Homo sapiens | 38 | 38 | 9606 | FALSE |
| CHEMBL3885598 | Kv channel-interacting protein 2/Potassium voltage-gated channel subfamily D member 3 | Q9UK17|Q9NS61 | PROTEIN COMPLEX | Homo sapiens | 9 | 9 | 9606 | FALSE |
| CHEMBL3885531 | C-C motif chemokine 5/Neutrophil defensin 1 (Defensin, alpha 1) | P13501|P59665 | PROTEIN-PROTEIN INTERACTION | Homo sapiens | 6 | 7 | 9606 | FALSE |
| CHEMBL4707 | Matrix metalloproteinase 26 | Q9NRE1 | SINGLE PROTEIN | Homo sapiens | 22 | 24 | 9606 | FALSE |
| CHEMBL1848 | Tubulin beta-2 chain | P68371 | SINGLE PROTEIN | Homo sapiens | 19 | 26 | 9606 | FALSE |
| CHEMBL5122 | Discoidin domain-containing receptor 2 | Q16832 | SINGLE PROTEIN | Homo sapiens | 1353 | 2246 | 9606 | FALSE |
| CHEMBL5950 | Non-specific lipid-transfer protein | P22307 | SINGLE PROTEIN | Homo sapiens | 2 | 5 | 9606 | FALSE |
| CHEMBL1974 | Tyrosine-protein kinase receptor FLT3 | P36888 | SINGLE PROTEIN | Homo sapiens | 7214 | 14422 | 9606 | FALSE |
| CHEMBL1892 | Glutamate carboxypeptidase II | Q04609 | SINGLE PROTEIN | Homo sapiens | 467 | 734 | 9606 | FALSE |
| CHEMBL5169271 | BCL2L2/BID | Q92843|P55957 | PROTEIN-PROTEIN INTERACTION | Homo sapiens | 5 | 5 | 9606 | FALSE |
| CHEMBL4106169 | CDK12/Cyclin K | O75909|Q9NYV4 | PROTEIN COMPLEX | Homo sapiens | 764 | 920 | 9606 | FALSE |
| CHEMBL4748231 | VHL/Uridine-cytidine kinase 2 | Q9BZX2|P40337 | PROTEIN-PROTEIN INTERACTION | Homo sapiens | 1 | 1 | 9606 | FALSE |
| CHEMBL1795140 | Transmembrane protease serine 2 | O15393 | SINGLE PROTEIN | Homo sapiens | 25 | 29 | 9606 | FALSE |
| CHEMBL4580 | Thymidine kinase, mitochondrial | O00142 | SINGLE PROTEIN | Homo sapiens | 78 | 96 | 9606 | FALSE |
| CHEMBL5465216 | Cereblon/Baculoviral IAP repeat-containing protein 2 | Q13490|Q96SW2 | PROTEIN-PROTEIN INTERACTION | Homo sapiens | 9 | 17 | 9606 | FALSE |
| CHEMBL1764937 | Membrane-associated phosphatidylinositol transfer protein 1 | O00562 | SINGLE PROTEIN | Homo sapiens | 2 | 2 | 9606 | FALSE |
| CHEMBL4296409 | CNE-2 |  | CELL-LINE | Homo sapiens | 245 | 395 | 9606 | FALSE |
| CHEMBL4748229 | VHL/Bcl-2-like protein 1 | Q07817|P40337 | PROTEIN-PROTEIN INTERACTION | Homo sapiens | 44 | 79 | 9606 | FALSE |
| CHEMBL2469 | Uridine-cytidine kinase 2 | Q9BZX2 | SINGLE PROTEIN | Homo sapiens | 20 | 58 | 9606 | FALSE |
| CHEMBL1741162 | Phosphomannomutase 2 | O15305 | SINGLE PROTEIN | Homo sapiens | 182 | 229 | 9606 | FALSE |
| CHEMBL1163123 | Mitogen-activated protein kinase kinase kinase 6 | O95382 | SINGLE PROTEIN | Homo sapiens | 447 | 537 | 9606 | FALSE |
| CHEMBL5839 | Delta(14)-sterol reductase | O76062 | SINGLE PROTEIN | Homo sapiens | 2 | 2 | 9606 | FALSE |
| CHEMBL3038479 | fMet-Leu-Phe receptor 1/2 | P21462|P25090 | PROTEIN FAMILY | Homo sapiens | 26 | 66 | 9606 | FALSE |
| CHEMBL2960 | Adenylate cyclase type VIII | P40145 | SINGLE PROTEIN | Homo sapiens | 109 | 190 | 9606 | FALSE |
| CHEMBL5465558 | TEAD2-YAP1 | P46937|Q15562 | PROTEIN-PROTEIN INTERACTION | Homo sapiens | 9 | 15 | 9606 | FALSE |
| CHEMBL5482985 | PD1-PDL2 | Q15116|Q9BQ51 | PROTEIN COMPLEX | Homo sapiens | 1 | 2 | 9606 | FALSE |
| CHEMBL4523905 | Mas-related G-protein coupled receptor member X3 | Q96LB0 | SINGLE PROTEIN | Homo sapiens | 2 | 2 | 9606 | FALSE |
| CHEMBL5446 | Sodium channel beta-2 subunit | O60939 | SINGLE PROTEIN | Homo sapiens | 1 | 1 | 9606 | FALSE |
| CHEMBL5291975 | Ephrin type-A receptor 2/Ephrin-A1 | P29317|P20827 | PROTEIN-PROTEIN INTERACTION | Homo sapiens | 2 | 2 | 9606 | FALSE |
| CHEMBL3038484 | Histone deacetylase 3/NCoR1 | O15379|O75376 | PROTEIN COMPLEX | Homo sapiens | 84 | 106 | 9606 | FALSE |
| CHEMBL1275209 | PH domain leucine-rich repeat-containing protein phosphatase 2 | Q6ZVD8 | SINGLE PROTEIN | Homo sapiens | 31 | 32 | 9606 | FALSE |
| CHEMBL5069373 | Zinc finger and BTB domain-containing protein 2 | Q8N680 | SINGLE PROTEIN | Homo sapiens | 1 | 9 | 9606 | FALSE |
| CHEMBL6008 | Carbonyl reductase [NADPH] 3 | O75828 | SINGLE PROTEIN | Homo sapiens | 14 | 42 | 9606 | FALSE |
| CHEMBL2073722 | Solute carrier family 22 member 16 | Q86VW1 | SINGLE PROTEIN | Homo sapiens | 4 | 4 | 9606 | FALSE |
| CHEMBL5465382 | MDM2-HDAC1 | Q00987|Q13547 | PROTEIN-PROTEIN INTERACTION | Homo sapiens | 4 | 20 | 9606 | FALSE |
| CHEMBL4523478 | C5a anaphylatoxin chemotactic receptor 2 | Q9P296 | SINGLE PROTEIN | Homo sapiens | 15 | 35 | 9606 | FALSE |
| CHEMBL3663 | Growth factor receptor-bound protein 2 | P62993 | SINGLE PROTEIN | Homo sapiens | 456 | 663 | 9606 | FALSE |
| CHEMBL4523298 | Pre-mRNA-splicing regulator WTAP | Q15007 | SINGLE PROTEIN | Homo sapiens | 1 | 1 | 9606 | FALSE |
| CHEMBL2111428 | VEGF-receptor 2 and stem cell growth factor receptor (KDR and KIT) | P10721|P35968 | SELECTIVITY GROUP | Homo sapiens | 10 | 10 | 9606 | FALSE |
| CHEMBL4523609 | Epoxide hydrolase 1/Bifunctional epoxide hydrolase 2 | P07099|P34913 | PROTEIN FAMILY | Homo sapiens | 4 | 8 | 9606 | FALSE |
| CHEMBL5209635 | Sodium/potassium/calcium exchanger 2 | Q9UI40 | SINGLE PROTEIN | Homo sapiens | 1 | 1 | 9606 | FALSE |
| CHEMBL4523738 | VHL/Estrogen-related receptor gamma | P62508|P40337 | PROTEIN-PROTEIN INTERACTION | Homo sapiens | 1 | 1 | 9606 | FALSE |
| CHEMBL4523705 | Protein cereblon/Bcl-2-like protein 1 | Q07817|Q96SW2 | PROTEIN-PROTEIN INTERACTION | Homo sapiens | 4 | 8 | 9606 | FALSE |
| CHEMBL3885576 | Gamma-aminobutyric acid receptor subunit alpha-5/beta-2 | P47870|P31644 | PROTEIN COMPLEX | Homo sapiens | 3 | 6 | 9606 | FALSE |
| CHEMBL4523735 | VHL/FLT3 | P36888|P40337 | PROTEIN-PROTEIN INTERACTION | Homo sapiens | 5 | 15 | 9606 | FALSE |
| CHEMBL3885588 | Histone acetyltransferase p300/Hypoxia-inducible factor 1-alpha | Q09472|Q16665 | PROTEIN COMPLEX | Homo sapiens | 17 | 21 | 9606 | FALSE |
| CHEMBL3885632 | Retinoic acid receptor RXR-alpha/Vitamin D3 receptor | P11473|P19793 | PROTEIN COMPLEX | Homo sapiens | 15 | 15 | 9606 | FALSE |
| CHEMBL3708 | Presenilin 2 | P49810 | SINGLE PROTEIN | Homo sapiens | 9 | 10 | 9606 | FALSE |
| CHEMBL4523926 | G-protein coupled receptor family C group 5 member B | Q9NZH0 | SINGLE PROTEIN | Homo sapiens | 1 | 1 | 9606 | FALSE |
| CHEMBL2095216 | Collagenase | P08253|P14780|P22894|P45452|P03956 | PROTEIN FAMILY | Homo sapiens | 150 | 153 | 9606 | FALSE |
| CHEMBL3632454 | Kinesin-like protein KIF15 | Q9NS87 | SINGLE PROTEIN | Homo sapiens | 32 | 55 | 9606 | FALSE |
| CHEMBL4598 | Serine/threonine-protein kinase MST1 | Q13043 | SINGLE PROTEIN | Homo sapiens | 1626 | 2689 | 9606 | FALSE |
| CHEMBL4523467 | Acetyl-coenzyme A synthetase, cytoplasmic | Q9NR19 | SINGLE PROTEIN | Homo sapiens | 424 | 548 | 9606 | FALSE |
| CHEMBL2111436 | Somatostatin receptor | P35346|P30874|P31391|P30872|P32745 | PROTEIN FAMILY | Homo sapiens | 39 | 50 | 9606 | FALSE |
| CHEMBL5043 | Endoplasmic reticulum aminopeptidase 2 | Q6P179 | SINGLE PROTEIN | Homo sapiens | 353 | 388 | 9606 | FALSE |
| CHEMBL4722 | Serine/threonine-protein kinase Aurora-A | O14965 | SINGLE PROTEIN | Homo sapiens | 7916 | 10395 | 9606 | FALSE |
| CHEMBL2363032 | Voltage-gated calcium channel | O95180|P54289|Q13936|Q8IZS8|Q13698|Q9NY47|Q01668|O00555|Q00975|O43497|Q7Z3S7|Q9P0X4|O60840|Q15878|O00305|Q08289|Q02641|P54284|P62955|Q9UF02|Q9Y698|Q9UBN1|Q8WXS5|O60359|Q9BXT2|Q06432 | PROTEIN COMPLEX GROUP | Homo sapiens | 129 | 146 | 9606 | FALSE |
| CHEMBL3885566 | Dual specificity mitogen-activated protein kinase kinase 1/Mitogen-activated protein kinase 1/RAF proto-oncogene serine/threonine-protein kinase | P28482|P04049|Q02750 | PROTEIN FAMILY | Homo sapiens | 2 | 2 | 9606 | FALSE |
| CHEMBL2095215 | Ribonucleoside-diphosphate reductase RR1 | P23921|Q7LG56|P31350 | PROTEIN COMPLEX GROUP | Homo sapiens | 205 | 391 | 9606 | FALSE |
| CHEMBL2111388 | Beta-2 adrenergic receptor and beta-3 adrenergic receptor | P07550|P13945 | SELECTIVITY GROUP | Homo sapiens | 153 | 173 | 9606 | FALSE |
| CHEMBL308 | Cyclin-dependent kinase 1 | P06493 | SINGLE PROTEIN | Homo sapiens | 3196 | 3984 | 9606 | FALSE |
| CHEMBL2169735 | 7-dehydrocholesterol reductase | Q9UBM7 | SINGLE PROTEIN | Homo sapiens | 22 | 35 | 9606 | FALSE |
| CHEMBL3407320 | Neurogenic locus notch homolog protein 2 | Q04721 | SINGLE PROTEIN | Homo sapiens | 1 | 1 | 9606 | FALSE |
| CHEMBL2221342 | Complex of retinoic acid binding (CRABPII) and inhibitor of apoptosis (cIAP1) proteins | P29373|Q13490 | PROTEIN COMPLEX | Homo sapiens | 4 | 18 | 9606 | FALSE |
| CHEMBL2095190 | GABA-A receptor; alpha-6/beta-3/gamma-2 | P18507|P28472|Q16445 | PROTEIN COMPLEX | Homo sapiens | 243 | 367 | 9606 | FALSE |
| CHEMBL5716 | Nuclear receptor subfamily 2 group C member 2 | P49116 | SINGLE PROTEIN | Homo sapiens | 40 | 46 | 9606 | FALSE |
| CHEMBL5903 | Activin receptor type-1 | Q04771 | SINGLE PROTEIN | Homo sapiens | 1156 | 1561 | 9606 | FALSE |
| CHEMBL4625 | Apoptosis regulator Bcl-X | Q07817 | SINGLE PROTEIN | Homo sapiens | 1840 | 2616 | 9606 | FALSE |
| CHEMBL4296070 | Somatostatin receptor type 2/type 5 | P35346|P30874 | PROTEIN FAMILY | Homo sapiens | 2 | 4 | 9606 | FALSE |
| CHEMBL3544 | Orphan nuclear receptor LRH-1 | O00482 | SINGLE PROTEIN | Homo sapiens | 380 | 740 | 9606 | FALSE |
| CHEMBL4523214 | Transcription factor HIVEP2 | P31629 | SINGLE PROTEIN | Homo sapiens | 1 | 1 | 9606 | FALSE |
| CHEMBL2095162 | Peroxisome proliferator-activated receptor gamma/Nuclear receptor coactivator 1 | P37231|Q15788 | PROTEIN-PROTEIN INTERACTION | Homo sapiens | 749 | 749 | 9606 | FALSE |
| CHEMBL1293227 | Ubiquitin carboxyl-terminal hydrolase 2 | O75604 | SINGLE PROTEIN | Homo sapiens | 8148 | 8821 | 9606 | FALSE |
| CHEMBL3137290 | SUMO E1/E2 | Q9UBE0|Q9UBT2|P63279 | PROTEIN COMPLEX | Homo sapiens | 112 | 116 | 9606 | FALSE |
| CHEMBL3136 | Sarcoplasmic/endoplasmic reticulum calcium ATPase 1 | O14983 | SINGLE PROTEIN | Homo sapiens | 45 | 55 | 9606 | FALSE |
| CHEMBL5482987 | cIAP1-BTK | Q06187|Q13490 | PROTEIN-PROTEIN INTERACTION | Homo sapiens | 1 | 1 | 9606 | FALSE |
| CHEMBL3217376 | Putative N-acetylglucosamine-6-phosphate deacetylase | Q9Y303 | SINGLE PROTEIN | Homo sapiens | 1 | 1 | 9606 | FALSE |
| CHEMBL5872 | G-protein coupled estrogen receptor 1 | Q99527 | SINGLE PROTEIN | Homo sapiens | 33 | 111 | 9606 | FALSE |
| CHEMBL5465335 | E3 ubiquitin-protein ligase COP1 | Q8NHY2 | SINGLE PROTEIN | Homo sapiens | 1 | 1 | 9606 | FALSE |
| CHEMBL3627587 | Indoleamine 2,3-dioxygenase 2 | Q6ZQW0 | SINGLE PROTEIN | Homo sapiens | 140 | 323 | 9606 | FALSE |
| CHEMBL2111438 | Neurotensin receptor | O95665|P30989 | PROTEIN FAMILY | Homo sapiens | 14 | 16 | 9606 | FALSE |
| CHEMBL1250402 | Transient receptor potential cation channel subfamily M member 2 | O94759 | SINGLE PROTEIN | Homo sapiens | 209 | 348 | 9606 | FALSE |
| CHEMBL5465341 | SWI/SNF-related matrix-associated actin-dependent regulator of chromatin subfamily D member 2 | Q92925 | SINGLE PROTEIN | Homo sapiens | 1 | 1 | 9606 | FALSE |
| CHEMBL4106172 | Glucocerebrosidase GBA1/GBA2 | P04062|Q9HCG7 | PROTEIN FAMILY | Homo sapiens | 2 | 3 | 9606 | FALSE |
| CHEMBL5465285 | Peroxisome biogenesis factor 2 | P28328 | SINGLE PROTEIN | Homo sapiens | 1 | 1 | 9606 | FALSE |
| CHEMBL3038492 | JAK2/JAK1 | P23458|O60674 | PROTEIN COMPLEX | Homo sapiens | 70 | 101 | 9606 | FALSE |
| CHEMBL4535 | GABA transporter 2 | Q9NSD5 | SINGLE PROTEIN | Homo sapiens | 94 | 120 | 9606 | FALSE |
| CHEMBL3038453 | AMPK alpha1/beta2/gamma1 | O43741|P54619|Q13131 | PROTEIN COMPLEX | Homo sapiens | 43 | 55 | 9606 | FALSE |
| CHEMBL2111437 | Melanin-concentrating hormone receptor | Q969V1|Q99705 | PROTEIN FAMILY | Homo sapiens | 51 | 55 | 9606 | FALSE |
| CHEMBL614894 | SAOS-2 |  | CELL-LINE | Homo sapiens | 474 | 702 | 9606 | FALSE |
| CHEMBL1907603 | Glutamate NMDA receptor; GRIN1/GRIN2B | Q05586|Q13224 | PROTEIN COMPLEX | Homo sapiens | 520 | 775 | 9606 | FALSE |
| CHEMBL3596085 | Angiotensinogen | P01019 | SINGLE PROTEIN | Homo sapiens | 5 | 5 | 9606 | FALSE |
| CHEMBL2095160 | Leukotriene B4 receptor | Q9NPC1|Q15722 | PROTEIN FAMILY | Homo sapiens | 375 | 823 | 9606 | FALSE |
| CHEMBL4860 | Apoptosis regulator Bcl-2 | P10415 | SINGLE PROTEIN | Homo sapiens | 2683 | 3801 | 9606 | FALSE |
| CHEMBL4296012 | Telomeric repeat-binding factor 2 | Q15554 | SINGLE PROTEIN | Homo sapiens | 59 | 63 | 9606 | FALSE |
| CHEMBL5137 | Metabotropic glutamate receptor 2 | Q14416 | SINGLE PROTEIN | Homo sapiens | 1871 | 3266 | 9606 | FALSE |
| CHEMBL3038515 | Tankyrase 1/2 | Q9H2K2|O95271 | PROTEIN FAMILY | Homo sapiens | 236 | 384 | 9606 | FALSE |
| CHEMBL5483091 | VHL-EP300 | Q09472|P40337 | PROTEIN-PROTEIN INTERACTION | Homo sapiens | 3 | 3 | 9606 | FALSE |
| CHEMBL3276 | Interleukin-2 receptor beta chain | P14784 | SINGLE PROTEIN | Homo sapiens | 1 | 1 | 9606 | FALSE |
| CHEMBL3038465 | cIAP1/cIAP2 | Q13489|Q13490 | PROTEIN FAMILY | Homo sapiens | 2 | 2 | 9606 | FALSE |
| CHEMBL4522 | BMP-2-inducible protein kinase | Q9NSY1 | SINGLE PROTEIN | Homo sapiens | 575 | 759 | 9606 | FALSE |
| CHEMBL2111321 | Matrix metalloproteinase (2 and 3) | P08254|P08253 | SELECTIVITY GROUP | Homo sapiens | 53 | 56 | 9606 | FALSE |
| CHEMBL4523965 | Cholecystokinin(CCK) A/B | P32239|P32238 | SELECTIVITY GROUP | Homo sapiens | 3 | 3 | 9606 | FALSE |
| CHEMBL5393 | ATP-binding cassette sub-family G member 2 | Q9UNQ0 | SINGLE PROTEIN | Homo sapiens | 2139 | 5069 | 9606 | FALSE |
| CHEMBL3751647 | Telomeric repeat-binding factor 2-interacting protein 1 | Q9NYB0 | SINGLE PROTEIN | Homo sapiens | 30 | 53 | 9606 | FALSE |
| CHEMBL4523718 | E3 ubiquitin-protein ligase Mdm2/CDK6 | Q00987|Q00534 | PROTEIN-PROTEIN INTERACTION | Homo sapiens | 3 | 6 | 9606 | FALSE |
| CHEMBL2226 | Caspase-4 | P49662 | SINGLE PROTEIN | Homo sapiens | 65 | 81 | 9606 | FALSE |
| CHEMBL4875 | Adenylosuccinate synthetase 2 | P30520 | SINGLE PROTEIN | Homo sapiens | 3 | 3 | 9606 | FALSE |
| CHEMBL3835 | Serine/threonine-protein kinase NEK2 | P51955 | SINGLE PROTEIN | Homo sapiens | 2551 | 3570 | 9606 | FALSE |
| CHEMBL5169204 | Palmitoyltransferase ZDHHC2 | Q9UIJ5 | SINGLE PROTEIN | Homo sapiens | 11 | 13 | 9606 | FALSE |
| CHEMBL2366188 | OS-RC-2 |  | CELL-LINE | Homo sapiens | 423 | 488 | 9606 | FALSE |
| CHEMBL1907605 | Cyclin-dependent kinase 2/cyclin E1 | P24864|P24941 | PROTEIN COMPLEX | Homo sapiens | 1794 | 1968 | 9606 | FALSE |
| CHEMBL1795129 | Interleukin-6 | P05231 | SINGLE PROTEIN | Homo sapiens | 41 | 43 | 9606 | FALSE |
| CHEMBL2109237 | Neuronal acetylcholine receptor; alpha6/beta2 | P17787|Q15825 | PROTEIN COMPLEX | Homo sapiens | 12 | 12 | 9606 | FALSE |
| CHEMBL3885585 | Heat shock 70 kDa protein 1A/1B | P0DMV8|P0DMV9 | PROTEIN FAMILY | Homo sapiens | 2 | 2 | 9606 | FALSE |
| CHEMBL2185 | Serine/threonine-protein kinase Aurora-B | Q96GD4 | SINGLE PROTEIN | Homo sapiens | 5095 | 6989 | 9606 | FALSE |
| CHEMBL4523286 | GRB2-associated-binding protein 1 | Q13480 | SINGLE PROTEIN | Homo sapiens | 22 | 44 | 9606 | FALSE |
| CHEMBL3885564 | Dipeptidyl peptidase 8/9 | Q6V1X1|Q86TI2 | PROTEIN FAMILY | Homo sapiens | 22 | 22 | 9606 | FALSE |
| CHEMBL5483182 | CDK1/Cyclin A | P06493|P20248|P78396 | PROTEIN COMPLEX GROUP | Homo sapiens | 1 | 1 | 9606 | FALSE |
| CHEMBL4802036 | Fibroblast growth factor 1/Fibroblast growth factor receptor 2 | P05230|P21802 | PROTEIN COMPLEX | Homo sapiens | 2 | 3 | 9606 | FALSE |
| CHEMBL4295945 | 5'-nucleotidase domain-containing protein 2 | Q9H857 | SINGLE PROTEIN | Homo sapiens | 1 | 1 | 9606 | FALSE |
| CHEMBL3112379 | Phosphatidylcholine:ceramide cholinephosphotransferase 2 | Q8NHU3 | SINGLE PROTEIN | Homo sapiens | 148 | 194 | 9606 | FALSE |
| CHEMBL5318 | Apoptosis regulator BAX | Q07812 | SINGLE PROTEIN | Homo sapiens | 50 | 78 | 9606 | FALSE |
| CHEMBL3038513 | Toll-like receptor 4/MD-2/CD14 | O00206|Q9Y6Y9|P08571 | PROTEIN COMPLEX | Homo sapiens | 10 | 27 | 9606 | FALSE |
| CHEMBL1250350 | Prolyl 4-hydroxylase subunit alpha-1 | P13674 | SINGLE PROTEIN | Homo sapiens | 8 | 16 | 9606 | FALSE |
| CHEMBL4630762 | Sodium channel protein type 4 subunit alpha/beta-1/beta-2 | P35499|O60939|Q07699 | PROTEIN COMPLEX | Homo sapiens | 1 | 2 | 9606 | FALSE |
| CHEMBL4105790 | Bifunctional 3'-phosphoadenosine 5'-phosphosulfate synthase 2 | O95340 | SINGLE PROTEIN | Homo sapiens | 4 | 4 | 9606 | FALSE |
| CHEMBL2640 | Hexokinase type II | P52789 | SINGLE PROTEIN | Homo sapiens | 56 | 89 | 9606 | FALSE |
| CHEMBL5291978 | Catenin beta-1/BCL9L | P35222|Q86UU0 | PROTEIN COMPLEX | Homo sapiens | 1 | 1 | 9606 | FALSE |
| CHEMBL4923 | Peptidyl-prolyl cis-trans isomerase NIMA-interacting 2 | Q9Y237 | SINGLE PROTEIN | Homo sapiens | 6 | 7 | 9606 | FALSE |
| CHEMBL5949 | Nuclear receptor corepressor 2 | Q9Y618 | SINGLE PROTEIN | Homo sapiens | 3 | 3 | 9606 | FALSE |
| CHEMBL2321627 | C-C chemokine receptor-like 2 | O00421 | SINGLE PROTEIN | Homo sapiens | 156 | 177 | 9606 | FALSE |
| CHEMBL3301392 | JAK2/TYK2 | O60674|P29597 | PROTEIN COMPLEX | Homo sapiens | 56 | 76 | 9606 | FALSE |
| CHEMBL3751648 | Period circadian protein homolog 2 | O15055 | SINGLE PROTEIN | Homo sapiens | 41 | 80 | 9606 | FALSE |
| CHEMBL5465555 | VHL-Estrogen receptor beta | Q92731|P40337 | PROTEIN-PROTEIN INTERACTION | Homo sapiens | 2 | 2 | 9606 | FALSE |
| CHEMBL4296243 | Protein-cysteine N-palmitoyltransferase HHAT | Q5VTY9 | SINGLE PROTEIN | Homo sapiens | 59 | 159 | 9606 | FALSE |
| CHEMBL4630733 | Protein cereblon/Platelet-derived growth factor receptor alpha | P16234|Q96SW2 | PROTEIN-PROTEIN INTERACTION | Homo sapiens | 1 | 1 | 9606 | FALSE |
| CHEMBL5667 | Phosphatidylinositol-5-phosphate 4-kinase type-2 beta | P78356 | SINGLE PROTEIN | Homo sapiens | 372 | 457 | 9606 | FALSE |
| CHEMBL3976 | Dipeptidyl peptidase II | Q9UHL4 | SINGLE PROTEIN | Homo sapiens | 1801 | 2059 | 9606 | FALSE |
| CHEMBL5465243 | VHL/Baculoviral IAP repeat-containing protein 2 | Q13490|P40337 | PROTEIN-PROTEIN INTERACTION | Homo sapiens | 17 | 30 | 9606 | FALSE |
| CHEMBL3325308 | SPRY domain-containing SOCS box protein 2 | Q99619 | SINGLE PROTEIN | Homo sapiens | 21 | 22 | 9606 | FALSE |
| CHEMBL5291691 | VHL/Vascular endothelial growth factor receptor 2 | P35968|P40337 | PROTEIN-PROTEIN INTERACTION | Homo sapiens | 19 | 56 | 9606 | FALSE |
| CHEMBL3038493 | Casein kinase I/MDM2 | Q00987|P48729 | PROTEIN COMPLEX | Homo sapiens | 5 | 24 | 9606 | FALSE |
| CHEMBL2094256 | Angiotensin II receptor | P30556|P50052 | PROTEIN FAMILY | Homo sapiens | 934 | 1039 | 9606 | FALSE |
| CHEMBL2363017 | Potassium voltage-gated channel subfamily H member 3 | Q9ULD8 | SINGLE PROTEIN | Homo sapiens | 111 | 112 | 9606 | FALSE |
| CHEMBL2194 | Arylamine N-acetyltransferase 2 | P11245 | SINGLE PROTEIN | Homo sapiens | 3 | 3 | 9606 | FALSE |
| CHEMBL4523252 | Taste receptor type 2 member 45 | P59539 | SINGLE PROTEIN | Homo sapiens | 7 | 8 | 9606 | FALSE |
| CHEMBL3707462 | Methyl-CpG-binding domain protein 2 | Q9UBB5 | SINGLE PROTEIN | Homo sapiens | 8 | 8 | 9606 | FALSE |
| CHEMBL3293 | Thyroid oxidase 2 | Q9NRD8 | SINGLE PROTEIN | Homo sapiens | 1 | 1 | 9606 | FALSE |
| CHEMBL3885557 | Cyclin-dependent kinase inhibitor 1B/S-phase kinase-associated protein 2 | Q13309|P46527 | PROTEIN-PROTEIN INTERACTION | Homo sapiens | 1 | 1 | 9606 | FALSE |
| CHEMBL3721310 | Putative short transient receptor potential channel 2-like protein | Q6ZNB5 | SINGLE PROTEIN | Homo sapiens | 1 | 1 | 9606 | FALSE |
| CHEMBL2097 | Phospholipase A2 group IIC | Q5R387 | SINGLE PROTEIN | Homo sapiens | 24 | 24 | 9606 | FALSE |
| CHEMBL3763001 | Sodium/potassium/calcium exchanger 6, mitochondrial | Q6J4K2 | SINGLE PROTEIN | Homo sapiens | 4 | 8 | 9606 | FALSE |
| CHEMBL2111461 | Integrin alpha-2/beta-3 | P05106|P17301 | PROTEIN COMPLEX | Homo sapiens | 20 | 21 | 9606 | FALSE |
| CHEMBL2366168 | RL95-2 |  | CELL-LINE | Homo sapiens | 64 | 68 | 9606 | FALSE |
| CHEMBL2682 | Epididymis-specific alpha-mannosidase | Q9Y2E5 | SINGLE PROTEIN | Homo sapiens | 14 | 14 | 9606 | FALSE |
| CHEMBL5911 | Elongation of very long chain fatty acids protein 2 | Q9NXB9 | SINGLE PROTEIN | Homo sapiens | 4 | 4 | 9606 | FALSE |
| CHEMBL5640 | Prolyl 4-hydroxylase subunit alpha-2 | O15460 | SINGLE PROTEIN | Homo sapiens | 12 | 12 | 9606 | FALSE |
| CHEMBL2374 | Gamma-secretase subunit PEN-2 | Q9NZ42 | SINGLE PROTEIN | Homo sapiens | 26 | 38 | 9606 | FALSE |
| CHEMBL2375 | GABA receptor rho-2 subunit | P28476 | SINGLE PROTEIN | Homo sapiens | 9 | 11 | 9606 | FALSE |
| CHEMBL3396 | DNA topoisomerase II beta | Q02880 | SINGLE PROTEIN | Homo sapiens | 549 | 967 | 9606 | FALSE |
| CHEMBL4708 | Serine/threonine-protein kinase MST2 | Q13188 | SINGLE PROTEIN | Homo sapiens | 2217 | 3144 | 9606 | FALSE |
| CHEMBL2366353 | OPM-2 |  | CELL-LINE | Homo sapiens | 179 | 221 | 9606 | FALSE |
| CHEMBL3885546 | Corticotropin-releasing factor receptor 2/Corticotropin-releasing factor-binding protein | Q13324|P24387 | PROTEIN COMPLEX | Homo sapiens | 2420 | 4148 | 9606 | FALSE |
| CHEMBL4589 | Glutathione S-transferase Mu 2 | P28161 | SINGLE PROTEIN | Homo sapiens | 84 | 85 | 9606 | FALSE |
| CHEMBL5247 | Serine/threonine-protein kinase ILK-1 | Q13418 | SINGLE PROTEIN | Homo sapiens | 436 | 618 | 9606 | FALSE |
| CHEMBL4630742 | VHL/Protein-tyrosine phosphatase 2C | Q06124|P40337 | PROTEIN-PROTEIN INTERACTION | Homo sapiens | 23 | 71 | 9606 | FALSE |
| CHEMBL2111443 | Integrin alpha-V/beta-3 and alpha-IIb/beta 3 | P05106|P06756|P08514 | SELECTIVITY GROUP | Homo sapiens | 49 | 49 | 9606 | FALSE |
| CHEMBL2095396 | Dopamine receptors; D2 & D4 | P14416|P21917 | SELECTIVITY GROUP | Homo sapiens | 284 | 375 | 9606 | FALSE |
| CHEMBL5465245 | VHL/Cyclin-dependent kinase 12 | P40337|Q9NYV4 | PROTEIN-PROTEIN INTERACTION | Homo sapiens | 2 | 4 | 9606 | FALSE |
| CHEMBL4295967 | Diphosphoinositol polyphosphate phosphohydrolase 2 | Q9NZJ9 | SINGLE PROTEIN | Homo sapiens | 5 | 12 | 9606 | FALSE |
| CHEMBL4739855 | 2-hydroxyacylsphingosine 1-beta-galactosyltransferase | Q16880 | SINGLE PROTEIN | Homo sapiens | 21 | 38 | 9606 | FALSE |
| CHEMBL3093 | Dihydroorotase | P27708 | SINGLE PROTEIN | Homo sapiens | 26 | 27 | 9606 | FALSE |
| CHEMBL3712851 | Solute carrier family 13 member 2 | Q13183 | SINGLE PROTEIN | Homo sapiens | 2 | 3 | 9606 | FALSE |
| CHEMBL2685 | Collagen alpha 2 chain | P08123 | SINGLE PROTEIN | Homo sapiens | 1 | 4 | 9606 | FALSE |
| CHEMBL1743292 | Sulfotransferase 1A2 | P50226 | SINGLE PROTEIN | Homo sapiens | 3 | 3 | 9606 | FALSE |
| CHEMBL5819 | Serine/threonine-protein kinase Nek4 | P51957 | SINGLE PROTEIN | Homo sapiens | 947 | 1011 | 9606 | FALSE |
| CHEMBL5169190 | Dehydrogenase/reductase SDR family member 6 | Q9BUT1 | SINGLE PROTEIN | Homo sapiens | 1 | 1 | 9606 | FALSE |
| CHEMBL4295686 | Mitochondrial pyruvate carrier 2 | O95563 | SINGLE PROTEIN | Homo sapiens | 2 | 2 | 9606 | FALSE |
| CHEMBL4421 | HM74 nicotinic acid GPCR | P49019 | SINGLE PROTEIN | Homo sapiens | 285 | 353 | 9606 | FALSE |
| CHEMBL4523636 | CDK17/Cyclin-A2 | P20248|Q00537 | PROTEIN COMPLEX | Homo sapiens | 1 | 1 | 9606 | FALSE |
| CHEMBL2527 | Serine/threonine-protein kinase Chk2 | O96017 | SINGLE PROTEIN | Homo sapiens | 2796 | 4104 | 9606 | FALSE |
| CHEMBL3885514 | Apoptosis regulator BAX/Bcl-2-related protein A1 | Q07812|Q16548 | PROTEIN-PROTEIN INTERACTION | Homo sapiens | 1 | 1 | 9606 | FALSE |
| CHEMBL3884 | Sodium/glucose cotransporter 2 | P31639 | SINGLE PROTEIN | Homo sapiens | 1604 | 2023 | 9606 | FALSE |
| CHEMBL3885641 | Solute carrier family 12 member 2/member 5 | P55011|Q9H2X9 | PROTEIN COMPLEX | Homo sapiens | 22 | 22 | 9606 | FALSE |
| CHEMBL2531 | P2X purinoceptor 2 | Q9UBL9 | SINGLE PROTEIN | Homo sapiens | 137 | 190 | 9606 | FALSE |
| CHEMBL5261 | Serine/threonine-protein kinase TAO1 | Q7L7X3 | SINGLE PROTEIN | Homo sapiens | 1612 | 2058 | 9606 | FALSE |
| CHEMBL3885595 | Integrin alpha-5/Neuronal acetylcholine receptor subunit alpha-3/Neuronal acetylcholine receptor subunit beta-2/Neuronal acetylcholine receptor subunit beta-4 | P17787|P30926|P32297|P08648 | PROTEIN COMPLEX | Homo sapiens | 13 | 25 | 9606 | FALSE |
| CHEMBL5435 | Serine/threonine-protein kinase ULK2 | Q8IYT8 | SINGLE PROTEIN | Homo sapiens | 481 | 680 | 9606 | FALSE |
| CHEMBL2189116 | Histone-lysine N-methyltransferase EZH1 | Q92800 | SINGLE PROTEIN | Homo sapiens | 103 | 123 | 9606 | FALSE |
| CHEMBL3299 | Src homology 2 domain containing transforming protein C2 | P98077 | SINGLE PROTEIN | Homo sapiens | 1 | 1 | 9606 | FALSE |
| CHEMBL4523690 | Protein cereblon/MAP2K2 | P36507|Q96SW2 | PROTEIN-PROTEIN INTERACTION | Homo sapiens | 9 | 15 | 9606 | FALSE |
| CHEMBL4296126 | Cereblon/Cyclin-dependent kinase 19 | Q9BWU1|Q96SW2 | PROTEIN-PROTEIN INTERACTION | Homo sapiens | 1 | 1 | 9606 | FALSE |
| CHEMBL3175 | Topoisomerase (DNA) II binding protein 1 | Q92547 | SINGLE PROTEIN | Homo sapiens | 18 | 18 | 9606 | FALSE |
| CHEMBL4076 | Sodium/calcium exchanger 1 | P32418 | SINGLE PROTEIN | Homo sapiens | 386 | 416 | 9606 | FALSE |
| CHEMBL4793 | Dipeptidyl peptidase IX | Q86TI2 | SINGLE PROTEIN | Homo sapiens | 1544 | 1702 | 9606 | FALSE |
| CHEMBL4523999 | mTORC2 | P42345|Q6R327|Q9BVC4|Q9BPZ7 | PROTEIN COMPLEX | Homo sapiens | 187 | 194 | 9606 | FALSE |
| CHEMBL4204 | MAP kinase signal-integrating kinase 2 | Q9HBH9 | SINGLE PROTEIN | Homo sapiens | 2622 | 3634 | 9606 | FALSE |
| CHEMBL3038475 | CDK9/Cyclin K | P50750|O75909 | PROTEIN COMPLEX | Homo sapiens | 144 | 151 | 9606 | FALSE |
| CHEMBL4523 | Serine/threonine-protein kinase PIM2 | Q9P1W9 | SINGLE PROTEIN | Homo sapiens | 4631 | 5954 | 9606 | FALSE |
| CHEMBL5291586 | Fibroblast growth factor receptor substrate 2 | Q8WU20 | SINGLE PROTEIN | Homo sapiens | 1 | 3 | 9606 | FALSE |
| CHEMBL5291961 | CDK9/Cyclin T | O60563|P50750|O60583 | PROTEIN COMPLEX GROUP | Homo sapiens | 17 | 17 | 9606 | FALSE |
| CHEMBL3619 | UDP-glucuronosyltransferase 1A4 | P22310 | SINGLE PROTEIN | Homo sapiens | 203 | 294 | 9606 | FALSE |
| CHEMBL6190 | Voltage-dependent anion-selective channel protein 2 | P45880 | SINGLE PROTEIN | Homo sapiens | 4 | 6 | 9606 | FALSE |
| CHEMBL2150840 | 2-oxoglutarate receptor 1 | Q96P68 | SINGLE PROTEIN | Homo sapiens | 9 | 9 | 9606 | FALSE |
| CHEMBL3883303 | Aurora kinase A/B | Q96GD4|O14965 | PROTEIN FAMILY | Homo sapiens | 31 | 33 | 9606 | FALSE |
| CHEMBL4610 | Xaa-Pro aminopeptidase 2 | O43895 | SINGLE PROTEIN | Homo sapiens | 21 | 41 | 9606 | FALSE |
| CHEMBL4712 | Neutral sphingomyelinase | O60906 | SINGLE PROTEIN | Homo sapiens | 28 | 40 | 9606 | FALSE |
| CHEMBL2094127 | Cyclin-dependent kinase 1/cyclin B | P06493|P14635|Q8WWL7|O95067 | PROTEIN COMPLEX | Homo sapiens | 758 | 924 | 9606 | FALSE |
| CHEMBL4523470 | Sphingomyelin phosphodiesterase 3 | Q9NY59 | SINGLE PROTEIN | Homo sapiens | 112 | 116 | 9606 | FALSE |
| CHEMBL1961788 | Nuclear receptor subfamily 2 group E member 1 | Q9Y466 | SINGLE PROTEIN | Homo sapiens | 42 | 119 | 9606 | FALSE |
| CHEMBL2366231 | HDLM-2 |  | CELL-LINE | Homo sapiens | 130 | 132 | 9606 | FALSE |
| CHEMBL4729 | Cytochrome P450 2B6 | P20813 | SINGLE PROTEIN | Homo sapiens | 957 | 1373 | 9606 | FALSE |
| CHEMBL3885600 | Lymphocyte function-associated antigen 3/T-cell surface antigen CD2 | P06729|P19256 | PROTEIN-PROTEIN INTERACTION | Homo sapiens | 7 | 9 | 9606 | FALSE |
| CHEMBL3191 | Leukotriene B4 receptor 2 | Q9NPC1 | SINGLE PROTEIN | Homo sapiens | 87 | 146 | 9606 | FALSE |
| CHEMBL4523685 | Protein cereblon/Apoptosis regulator Bcl-2 | P10415|Q96SW2 | PROTEIN-PROTEIN INTERACTION | Homo sapiens | 15 | 74 | 9606 | FALSE |
| CHEMBL3883321 | Mu opioid receptor/Alpha-2A adrenergic receptor | P08913|P35372 | PROTEIN COMPLEX | Homo sapiens | 2 | 11 | 9606 | FALSE |
| CHEMBL2176776 | Sentrin-specific protease 2 | Q9HC62 | SINGLE PROTEIN | Homo sapiens | 74 | 79 | 9606 | FALSE |
| CHEMBL3638356 | Membrane metallo-endopeptidase-like 1 | Q495T6 | SINGLE PROTEIN | Homo sapiens | 44 | 65 | 9606 | FALSE |
| CHEMBL4523157 | Potassium channel subfamily K member 5 | O95279 | SINGLE PROTEIN | Homo sapiens | 2 | 4 | 9606 | FALSE |
| CHEMBL2366359 | JVM-2 |  | CELL-LINE | Homo sapiens | 147 | 152 | 9606 | FALSE |
| CHEMBL4802032 | Baculoviral IAP repeat-containing protein 2/Aldo-keto reductase family 1 member B1 | P15121|Q13490 | PROTEIN-PROTEIN INTERACTION | Homo sapiens | 1 | 1 | 9606 | FALSE |
| CHEMBL4105988 | Atypical chemokine receptor 2 | O00590 | SINGLE PROTEIN | Homo sapiens | 1 | 2 | 9606 | FALSE |
| CHEMBL4295746 | Long-chain-fatty-acid--CoA ligase 1 | P33121 | SINGLE PROTEIN | Homo sapiens | 39 | 40 | 9606 | FALSE |
| CHEMBL3301390 | JAK1/JAK2/TYK2 | P23458|O60674|P29597 | PROTEIN COMPLEX | Homo sapiens | 38 | 47 | 9606 | FALSE |
| CHEMBL1163126 | Serine/threonine-protein kinase ICK | Q9UPZ9 | SINGLE PROTEIN | Homo sapiens | 443 | 513 | 9606 | FALSE |
| CHEMBL5291683 | Cereblon/Serine/threonine-protein kinase PAK 2 | Q13177|Q96SW2 | PROTEIN-PROTEIN INTERACTION | Homo sapiens | 1 | 2 | 9606 | FALSE |
| CHEMBL1741216 | NADPH oxidase 3 | Q9HBY0 | SINGLE PROTEIN | Homo sapiens | 8 | 9 | 9606 | FALSE |
| CHEMBL4523992 | Serine/threonine-protein kinase LATS | Q9NRM7|O95835 | PROTEIN FAMILY | Homo sapiens | 289 | 902 | 9606 | FALSE |
| CHEMBL4930 | Granzyme K | P49863 | SINGLE PROTEIN | Homo sapiens | 15 | 19 | 9606 | FALSE |
| CHEMBL2111394 | RXR alpha/PPAR gamma | P37231|P19793 | PROTEIN COMPLEX | Homo sapiens | 53 | 112 | 9606 | FALSE |
| CHEMBL2546 | Heme oxygenase 2 | P30519 | SINGLE PROTEIN | Homo sapiens | 247 | 250 | 9606 | FALSE |
| CHEMBL4523440 | Spindlin-2B | Q9BPZ2 | SINGLE PROTEIN | Homo sapiens | 3 | 4 | 9606 | FALSE |
| CHEMBL3883296 | Serine/threonine-protein kinase Chk1/2 | O96017|O14757 | PROTEIN FAMILY | Homo sapiens | 4 | 5 | 9606 | FALSE |
| CHEMBL3301388 | EZH2/SUZ12/EED/RBBP7/RBBP4 | Q15910|O75530|Q15022|Q09028|Q16576 | PROTEIN COMPLEX | Homo sapiens | 7 | 11 | 9606 | FALSE |
| CHEMBL5808 | PITSLRE serine/threonine-protein kinase CDC2L1 | P21127 | SINGLE PROTEIN | Homo sapiens | 278 | 349 | 9606 | FALSE |
| CHEMBL2109242 | GABA-C receptor | P28476|A8MPY1|P24046 | PROTEIN COMPLEX GROUP | Homo sapiens | 14 | 14 | 9606 | FALSE |
| CHEMBL1908381 | Peripheral plasma membrane protein CASK | O14936 | SINGLE PROTEIN | Homo sapiens | 293 | 357 | 9606 | FALSE |
| CHEMBL614917 | SK-MEL-2 |  | CELL-LINE | Homo sapiens | 43643 | 46541 | 9606 | FALSE |
| CHEMBL4106126 | TLR4-MD2 | O00206|Q9Y6Y9 | PROTEIN-PROTEIN INTERACTION | Homo sapiens | 4 | 7 | 9606 | FALSE |
| CHEMBL4681 | Aldo-keto-reductase family 1 member C3 | P42330 | SINGLE PROTEIN | Homo sapiens | 965 | 1567 | 9606 | FALSE |
| CHEMBL5785 | Interferon-induced, double-stranded RNA-activated protein kinase | P19525 | SINGLE PROTEIN | Homo sapiens | 494 | 587 | 9606 | FALSE |
| CHEMBL4523734 | VHL/MAP2K2 | P36507|P40337 | PROTEIN-PROTEIN INTERACTION | Homo sapiens | 12 | 53 | 9606 | FALSE |
| CHEMBL4296111 | GRIA1/CACNG2 | P42261|Q9Y698 | PROTEIN COMPLEX | Homo sapiens | 32 | 35 | 9606 | FALSE |
| CHEMBL4879503 | Calcium and integrin-binding protein 1 | Q99828 | SINGLE PROTEIN | Homo sapiens | 35 | 49 | 9606 | FALSE |
| CHEMBL4105941 | 2-amino-3-carboxymuconate-6-semialdehyde decarboxylase | Q8TDX5 | SINGLE PROTEIN | Homo sapiens | 79 | 133 | 9606 | FALSE |
| CHEMBL4295946 | Jupiter microtubule associated homolog 2 | Q9H910 | SINGLE PROTEIN | Homo sapiens | 1 | 1 | 9606 | FALSE |
| CHEMBL2417355 | Microtubule-associated serine/threonine-protein kinase 2 | Q6P0Q8 | SINGLE PROTEIN | Homo sapiens | 5 | 12 | 9606 | FALSE |
| CHEMBL4523508 | Ubiquitin carboxyl-terminal hydrolase 15 | Q9Y4E8 | SINGLE PROTEIN | Homo sapiens | 7 | 7 | 9606 | FALSE |
| CHEMBL4524005 | Secretory phospholipase A2 | Q5R387|Q9NZK7|P14555|Q9BZM2|Q9UNK4|P39877|O15496|P04054|Q9NZ20|Q9BZM1|Q9BX93 | PROTEIN FAMILY | Homo sapiens | 14 | 27 | 9606 | FALSE |
| CHEMBL2002 | Inosine-5'-monophosphate dehydrogenase 2 | P12268 | SINGLE PROTEIN | Homo sapiens | 1176 | 1326 | 9606 | FALSE |
| CHEMBL6000 | Serine/threonine-protein kinase RIO2 | Q9BVS4 | SINGLE PROTEIN | Homo sapiens | 500 | 661 | 9606 | FALSE |
| CHEMBL3885572 | Gamma-aminobutyric acid receptor subunit alpha-3/ beta-2 | P47870|P34903 | PROTEIN COMPLEX | Homo sapiens | 3 | 6 | 9606 | FALSE |
| CHEMBL1075195 | Serine/threonine-protein kinase TAO2 | Q9UL54 | SINGLE PROTEIN | Homo sapiens | 725 | 1007 | 9606 | FALSE |
| CHEMBL1795139 | Transmembrane protease serine 6 | Q8IU80 | SINGLE PROTEIN | Homo sapiens | 193 | 211 | 9606 | FALSE |
| CHEMBL1961789 | COUP transcription factor 1 | P10589 | SINGLE PROTEIN | Homo sapiens | 7 | 7 | 9606 | FALSE |
| CHEMBL4523971 | MMP-2/MMP-14 | P50281|P08253 | SELECTIVITY GROUP | Homo sapiens | 16 | 16 | 9606 | FALSE |
| CHEMBL3088 | Insulin-like growth factor binding protein 2 | P18065 | SINGLE PROTEIN | Homo sapiens | 2 | 2 | 9606 | FALSE |
| CHEMBL4523986 | Cytochrome P450 | P08684|P04798|P33260|P20815|P05177|P78329|P11712|P51589|P33261|P10632|P10635|Q02928|P20813|Q16678|P11509|Q9HB55|P05181|P24462|Q8TAV3|Q08477|Q16696|P98187|P20853|P24903|Q6VVX0|Q96SQ9|Q7Z449|Q5TCH4|P13584 | PROTEIN FAMILY | Homo sapiens | 291 | 439 | 9606 | FALSE |
| CHEMBL2331055 | PERQ amino acid-rich with GYF domain-containing protein 2 | Q6Y7W6 | SINGLE PROTEIN | Homo sapiens | 2 | 8 | 9606 | FALSE |
| CHEMBL2111363 | Histone deacetylase 3/Nuclear receptor corepressor 2 (HDAC3/NCoR2) | O15379|Q9Y618 | PROTEIN COMPLEX | Homo sapiens | 570 | 765 | 9606 | FALSE |
| CHEMBL3832941 | Tubulin alpha | P0DPH7|P68366|Q71U36|P68363|Q9BQE3|Q6PEY2|A6NHL2|Q9NY65 | PROTEIN FAMILY | Homo sapiens | 35 | 84 | 9606 | FALSE |
| CHEMBL1743125 | Solute carrier family 15 member 2 | Q16348 | SINGLE PROTEIN | Homo sapiens | 91 | 186 | 9606 | FALSE |
| CHEMBL5932 | LIM domain kinase 2 | P53671 | SINGLE PROTEIN | Homo sapiens | 744 | 980 | 9606 | FALSE |
| CHEMBL4524007 | Notch receptor | P46531|Q9UM47|Q04721|Q99466 | PROTEIN FAMILY | Homo sapiens | 2 | 2 | 9606 | FALSE |
| CHEMBL2363009 | Potassium voltage-gated channel subfamily C member 2 | Q96PR1 | SINGLE PROTEIN | Homo sapiens | 12 | 22 | 9606 | FALSE |
| CHEMBL4523243 | Glycogen [starch] synthase, liver | P54840 | SINGLE PROTEIN | Homo sapiens | 3 | 4 | 9606 | FALSE |
| CHEMBL4105974 | Eukaryotic peptide chain release factor GTP-binding subunit ERF3B | Q8IYD1 | SINGLE PROTEIN | Homo sapiens | 54 | 54 | 9606 | FALSE |
| CHEMBL4296128 | Cereblon/Histone deacetylase 2 | Q92769|Q96SW2 | PROTEIN-PROTEIN INTERACTION | Homo sapiens | 7 | 8 | 9606 | FALSE |
| CHEMBL2331053 | 6-phosphofructo-2-kinase/fructose-2,6-bisphosphatase 3 | Q16875 | SINGLE PROTEIN | Homo sapiens | 921 | 1469 | 9606 | FALSE |
| CHEMBL3038476 | ORAI 1/2/3 | Q96D31|Q96SN7|Q9BRQ5 | PROTEIN FAMILY | Homo sapiens | 41 | 82 | 9606 | FALSE |
| CHEMBL5169086 | CRBN/EZH2 | Q15910|Q96SW2 | PROTEIN-PROTEIN INTERACTION | Homo sapiens | 4 | 7 | 9606 | FALSE |
| CHEMBL6030 | Eukaryotic translation initiation factor 2-alpha kinase 3 | Q9NZJ5 | SINGLE PROTEIN | Homo sapiens | 374 | 651 | 9606 | FALSE |
| CHEMBL2366347 | KMOE-2 |  | CELL-LINE | Homo sapiens | 107 | 108 | 9606 | FALSE |
| CHEMBL6014 | Testis-specific serine/threonine-protein kinase 2 | Q96PF2 | SINGLE PROTEIN | Homo sapiens | 1165 | 1729 | 9606 | FALSE |
| CHEMBL1795108 | Elongation factor 2 | P13639 | SINGLE PROTEIN | Homo sapiens | 8 | 22 | 9606 | FALSE |
| CHEMBL5880 | Interleukin-2 | P60568 | SINGLE PROTEIN | Homo sapiens | 127 | 147 | 9606 | FALSE |
| CHEMBL5853 | Diacylglycerol O-acyltransferase 2 | Q96PD7 | SINGLE PROTEIN | Homo sapiens | 321 | 364 | 9606 | FALSE |
| CHEMBL4523755 | VHL-MAP2K1/MAP2K2 | P36507|Q02750|P40337 | PROTEIN-PROTEIN INTERACTION | Homo sapiens | 22 | 64 | 9606 | FALSE |
| CHEMBL2439944 | 2-acylglycerol O-acyltransferase 2 | Q3SYC2 | SINGLE PROTEIN | Homo sapiens | 225 | 249 | 9606 | FALSE |
| CHEMBL1961786 | Hepatocyte nuclear factor 4-gamma | Q14541 | SINGLE PROTEIN | Homo sapiens | 2 | 2 | 9606 | FALSE |
| CHEMBL2514 | Neurotensin receptor 2 | O95665 | SINGLE PROTEIN | Homo sapiens | 268 | 303 | 9606 | FALSE |
| CHEMBL5465325 | 2-acylglycerol O-acyltransferase 3 | Q86VF5 | SINGLE PROTEIN | Homo sapiens | 1 | 1 | 9606 | FALSE |
| CHEMBL1920 | GABA receptor beta-2 subunit | P47870 | SINGLE PROTEIN | Homo sapiens | 2 | 2 | 9606 | FALSE |
| CHEMBL1938208 | Lysine-specific histone demethylase 1B | Q8NB78 | SINGLE PROTEIN | Homo sapiens | 133 | 151 | 9606 | FALSE |
| CHEMBL2433 | Prohormone convertase 2 | P16519 | SINGLE PROTEIN | Homo sapiens | 8 | 8 | 9606 | FALSE |
| CHEMBL2117 | AMP-activated protein kinase, beta-2 subunit | O43741 | SINGLE PROTEIN | Homo sapiens | 4 | 4 | 9606 | FALSE |
| CHEMBL4574 | BR serine/threonine-protein kinase 2 | Q8IWQ3 | SINGLE PROTEIN | Homo sapiens | 947 | 1598 | 9606 | FALSE |
| CHEMBL5465273 | Procollagen-lysine,2-oxoglutarate 5-dioxygenase 2 | O00469 | SINGLE PROTEIN | Homo sapiens | 18 | 18 | 9606 | FALSE |
| CHEMBL5465300 | Procollagen-lysine,2-oxoglutarate 5-dioxygenase 1 | Q02809 | SINGLE PROTEIN | Homo sapiens | 6 | 6 | 9606 | FALSE |
| CHEMBL3822353 | Patatin-like phospholipase domain-containing protein 2 | Q96AD5 | SINGLE PROTEIN | Homo sapiens | 3 | 4 | 9606 | FALSE |
| CHEMBL3779760 | Lysine-specific demethylase 2B | Q8NHM5 | SINGLE PROTEIN | Homo sapiens | 339 | 347 | 9606 | FALSE |
| CHEMBL4532 | Vasoactive intestinal polypeptide receptor 2 | P41587 | SINGLE PROTEIN | Homo sapiens | 212 | 744 | 9606 | FALSE |
| CHEMBL4295810 | Aminoacyl tRNA synthase complex-interacting multifunctional protein 1 | Q12904 | SINGLE PROTEIN | Homo sapiens | 1 | 1 | 9606 | FALSE |
| CHEMBL5303741 | Gamma-aminobutyric acid receptor subunit alpha-1/alpha-2/beta-2/gamma-2 | P14867|P47870|P18507|P47869 | PROTEIN COMPLEX | Homo sapiens | 725 | 1034 | 9606 | FALSE |
| CHEMBL5249 | Geranylgeranyl transferase type II alpha subunit | Q92696 | SINGLE PROTEIN | Homo sapiens | 2 | 2 | 9606 | FALSE |
| CHEMBL4523969 | IKK2/IKK1 | O14920|O15111 | SELECTIVITY GROUP | Homo sapiens | 2 | 2 | 9606 | FALSE |
| CHEMBL4523487 | DNA dC->dU-editing enzyme APOBEC-3B | Q9UH17 | SINGLE PROTEIN | Homo sapiens | 8 | 8 | 9606 | FALSE |
| CHEMBL5291678 | Cereblon/FGFR2 | P21802|Q96SW2 | PROTEIN-PROTEIN INTERACTION | Homo sapiens | 10 | 24 | 9606 | FALSE |
| CHEMBL4630847 | Ubiquitin thioesterase OTUB2 | Q96DC9 | SINGLE PROTEIN | Homo sapiens | 3 | 3 | 9606 | FALSE |
| CHEMBL3885593 | Histone-lysine N-methyltransferase EHMT1/EHMT2 | Q9H9B1|Q96KQ7 | PROTEIN FAMILY | Homo sapiens | 7 | 8 | 9606 | FALSE |
| CHEMBL4105759 | Interleukin-1 receptor-associated kinase-like 2 | O43187 | SINGLE PROTEIN | Homo sapiens | 2 | 2 | 9606 | FALSE |
| CHEMBL2321618 | Potassium voltage-gated channel subfamily B member 2 | Q92953 | SINGLE PROTEIN | Homo sapiens | 2 | 2 | 9606 | FALSE |
| CHEMBL5169064 | VHL/NSD2 | O96028|P40337 | PROTEIN-PROTEIN INTERACTION | Homo sapiens | 4 | 7 | 9606 | FALSE |
| CHEMBL3832943 | Casein kinase 2 | P67870|P68400|P19784|Q8NEV1 | PROTEIN FAMILY | Homo sapiens | 329 | 420 | 9606 | FALSE |
| CHEMBL4523623 | PDE6D/KRAS | O43924|P01116 | PROTEIN-PROTEIN INTERACTION | Homo sapiens | 7 | 21 | 9606 | FALSE |
| CHEMBL3774299 | Histone acetyltransferase KAT7 | O95251 | SINGLE PROTEIN | Homo sapiens | 14 | 17 | 9606 | FALSE |
| CHEMBL3713355 | Polypeptide N-acetylgalactosaminyltransferase 2 | Q10471 | SINGLE PROTEIN | Homo sapiens | 8 | 34 | 9606 | FALSE |
| CHEMBL1926496 | Bone morphogenetic protein 2 | P12643 | SINGLE PROTEIN | Homo sapiens | 8 | 45 | 9606 | FALSE |
| CHEMBL6154 | Tankyrase-2 | Q9H2K2 | SINGLE PROTEIN | Homo sapiens | 989 | 1575 | 9606 | FALSE |
| CHEMBL5483010 | GRASP55-JAMB | Q9H8Y8|P57087 | PROTEIN COMPLEX | Homo sapiens | 2 | 2 | 9606 | FALSE |
| CHEMBL2161 | Glutathione transferase omega 2 | Q9H4Y5 | SINGLE PROTEIN | Homo sapiens | 2 | 2 | 9606 | FALSE |
| CHEMBL2111451 | Inositol 1,4,5-trisphosphate receptor | Q14571|Q14573|Q14643 | PROTEIN FAMILY | Homo sapiens | 6 | 13 | 9606 | FALSE |
| CHEMBL5646 | N-formyl peptide receptor 3 | P25089 | SINGLE PROTEIN | Homo sapiens | 548 | 561 | 9606 | FALSE |
| CHEMBL4105827 | Nucleotide triphosphate diphosphatase NUDT15 | Q9NV35 | SINGLE PROTEIN | Homo sapiens | 1 | 1 | 9606 | FALSE |
| CHEMBL5553 | ATP-dependent RNA helicase DDX3X | O00571 | SINGLE PROTEIN | Homo sapiens | 405 | 438 | 9606 | FALSE |
| CHEMBL3885541 | TCF4-CTNNB1 complex | P35222|P15884 | PROTEIN COMPLEX | Homo sapiens | 39 | 55 | 9606 | FALSE |
| CHEMBL3559705 | Taste receptor type 2 member 20 | P59543 | SINGLE PROTEIN | Homo sapiens | 8 | 10 | 9606 | FALSE |
| CHEMBL2062348 | Heat shock-related 70 kDa protein 2 | P54652 | SINGLE PROTEIN | Homo sapiens | 47 | 94 | 9606 | FALSE |
| CHEMBL2321629 | Protein O-linked-mannose beta-1,2-N-acetylglucosaminyltransferase 1 | Q8WZA1 | SINGLE PROTEIN | Homo sapiens | 3 | 3 | 9606 | FALSE |
| CHEMBL5586 | Carbonyl reductase [NADPH] 1 | P16152 | SINGLE PROTEIN | Homo sapiens | 45 | 138 | 9606 | FALSE |
| CHEMBL4879529 | Cereblon/Serine/threonine-protein kinase RIPK2 | O43353|Q96SW2 | PROTEIN-PROTEIN INTERACTION | Homo sapiens | 3 | 5 | 9606 | FALSE |
| CHEMBL4523560 | SU-DHL-2 |  | CELL-LINE | Homo sapiens | 94 | 129 | 9606 | FALSE |
| CHEMBL4523566 | SET-2 |  | CELL-LINE | Homo sapiens | 21 | 53 | 9606 | FALSE |
| CHEMBL2146312 | Werner syndrome ATP-dependent helicase | Q14191 | SINGLE PROTEIN | Homo sapiens | 8356 | 8824 | 9606 | FALSE |
| CHEMBL4296494 | SK-N-BE(2)-M17 |  | CELL-LINE | Homo sapiens | 35 | 195 | 9606 | FALSE |
| CHEMBL3559708 | Transient receptor potential cation channel subfamily M member 3 | Q9HCF6 | SINGLE PROTEIN | Homo sapiens | 2 | 2 | 9606 | FALSE |
| CHEMBL4295657 | Actin-related protein 2/3 complex subunit 2 | O15144 | SINGLE PROTEIN | Homo sapiens | 1 | 2 | 9606 | FALSE |
| CHEMBL3038455 | AMPK alpha2/beta1/gamma1 | P54646|P54619|Q9Y478 | PROTEIN COMPLEX | Homo sapiens | 145 | 216 | 9606 | FALSE |
| CHEMBL6029 | Eukaryotic translation initiation factor 2-alpha kinase 1 | Q9BQI3 | SINGLE PROTEIN | Homo sapiens | 537 | 712 | 9606 | FALSE |
| CHEMBL2111440 | VEGF-receptor 2 and PDGF-receptor beta (KDR and PDGFR beta) | P09619|P35968 | SELECTIVITY GROUP | Homo sapiens | 28 | 29 | 9606 | FALSE |
| CHEMBL4296108 | HIF1A/p300/CREB-binding protein | Q09472|Q16665|Q92793 | PROTEIN-PROTEIN INTERACTION | Homo sapiens | 8 | 14 | 9606 | FALSE |
| CHEMBL1615386 | Core-binding factor subunit beta | Q13951 | SINGLE PROTEIN | Homo sapiens | 34 | 36 | 9606 | FALSE |
| CHEMBL4295980 | Myosin-2 | Q9UKX2 | SINGLE PROTEIN | Homo sapiens | 5 | 5 | 9606 | FALSE |
| CHEMBL2111339 | GABA A receptor alpha-3/beta-2/gamma-2 | P47870|P18507|P34903 | PROTEIN COMPLEX | Homo sapiens | 71 | 101 | 9606 | FALSE |
| CHEMBL1770034 | Phosphatidylinositol-5-phosphate 4-kinase type-2 gamma | Q8TBX8 | SINGLE PROTEIN | Homo sapiens | 641 | 791 | 9606 | FALSE |
| CHEMBL4888444 | Cyclin-dependent kinase 2/4/6 | P11802|P24941|Q00534 | PROTEIN FAMILY | Homo sapiens | 2 | 3 | 9606 | FALSE |
| CHEMBL2778 | Ileal bile acid transporter | Q12908 | SINGLE PROTEIN | Homo sapiens | 344 | 415 | 9606 | FALSE |
| CHEMBL5952 | Neuropeptide FF receptor 2 | Q9Y5X5 | SINGLE PROTEIN | Homo sapiens | 229 | 549 | 9606 | FALSE |
| CHEMBL4523511 | Mitochondrial carrier homolog 2 | Q9Y6C9 | SINGLE PROTEIN | Homo sapiens | 2 | 4 | 9606 | FALSE |
| CHEMBL2363065 | Mitochondrial complex I (NADH dehydrogenase) | P03923|O95299|O00217|P03901|P56556|P03886|O95139|O00483|Q86Y39|P17568|P03891|O75306|P03915|P51970|O43674|P03905|P03897|O14561|O15239|O43181|O43676|O43677|O43678|O43920|O75251|O75380|O75438|O75489|O95167|O95168|O95169|O95178|O95182|O95298|O96000|P19404|P28331|P49821|P56181|Q16718|Q16795|Q8N183|Q9BU61|Q9NRX3|Q9NX14|Q9P032|Q9P0J0|Q9UI09|Q9Y375|Q9Y6M9 | PROTEIN COMPLEX | Homo sapiens | 13 | 28 | 9606 | FALSE |
| CHEMBL4523707 | Protein cereblon/Histone acetyltransferase KAT2A | Q92830|Q96SW2 | PROTEIN-PROTEIN INTERACTION | Homo sapiens | 1 | 4 | 9606 | FALSE |
| CHEMBL5169270 | BCL2A11/BBC3 | Q16548|Q9BXH1 | PROTEIN-PROTEIN INTERACTION | Homo sapiens | 1 | 2 | 9606 | FALSE |
| CHEMBL5169073 | VHL/EZH2 | Q15910|P40337 | PROTEIN-PROTEIN INTERACTION | Homo sapiens | 1 | 3 | 9606 | FALSE |
| CHEMBL3650 | Fibroblast growth factor receptor 1 | P11362 | SINGLE PROTEIN | Homo sapiens | 6364 | 9272 | 9606 | FALSE |
| CHEMBL5169077 | SOS1/VHL/ELOC/ELOB | Q07889|P40337|Q15370|Q15369 | PROTEIN-PROTEIN INTERACTION | Homo sapiens | 7 | 7 | 9606 | FALSE |
| CHEMBL5169136 | RNA-binding protein FXR2 | P51116 | SINGLE PROTEIN | Homo sapiens | 9 | 24 | 9606 | FALSE |
| CHEMBL3885587 | Histone acetyltransferase KAT2A/KAT2B | Q92831|Q92830 | PROTEIN FAMILY | Homo sapiens | 4 | 4 | 9606 | FALSE |
| CHEMBL4523679 | Protein cereblon/Histone deacetylase 3 | O15379|Q96SW2 | PROTEIN-PROTEIN INTERACTION | Homo sapiens | 4 | 4 | 9606 | FALSE |
| CHEMBL5465263 | Insulin-like growth factor 2 mRNA-binding protein 1 | Q9NZI8 | SINGLE PROTEIN | Homo sapiens | 2 | 7 | 9606 | FALSE |
| CHEMBL2366174 | CW-2 |  | CELL-LINE | Homo sapiens | 140 | 142 | 9606 | FALSE |
| CHEMBL4295652 | Myosin regulatory light chain 12B | O14950 | SINGLE PROTEIN | Homo sapiens | 1 | 1 | 9606 | FALSE |
| CHEMBL3883319 | Guanine nucleotide-binding protein G(I)/G(S)/G(T) subunit beta-1/gamma-2 | P62873|P59768 | PROTEIN COMPLEX | Homo sapiens | 39 | 71 | 9606 | FALSE |
| CHEMBL4295992 | YTH domain-containing family protein 2 | Q9Y5A9 | SINGLE PROTEIN | Homo sapiens | 19 | 27 | 9606 | FALSE |
| CHEMBL1764938 | Annexin A2 | P07355 | SINGLE PROTEIN | Homo sapiens | 7 | 15 | 9606 | FALSE |
| CHEMBL4295952 | X antigen family member 1 | Q9HD64 | SINGLE PROTEIN | Homo sapiens | 1 | 1 | 9606 | FALSE |
| CHEMBL4296096 | D(2) dopamine receptor/Metabotropic glutamate receptor 5 | P14416|P41594 | PROTEIN COMPLEX | Homo sapiens | 13 | 16 | 9606 | FALSE |
| CHEMBL5609 | Bcl-2 homologous antagonist/killer | Q16611 | SINGLE PROTEIN | Homo sapiens | 1 | 1 | 9606 | FALSE |
| CHEMBL3232682 | Ubiquitin carboxyl-terminal hydrolase 20 | Q9Y2K6 | SINGLE PROTEIN | Homo sapiens | 12 | 16 | 9606 | FALSE |
| CHEMBL3301399 | TLR2/TLR6 | O60603|Q9Y2C9 | PROTEIN COMPLEX | Homo sapiens | 30 | 54 | 9606 | FALSE |
| CHEMBL4523994 | Geranylgeranyl transferase type-2 | P53611|Q92696 | PROTEIN COMPLEX | Homo sapiens | 21 | 41 | 9606 | FALSE |
| CHEMBL5064 | Folate receptor beta | P14207 | SINGLE PROTEIN | Homo sapiens | 64 | 148 | 9606 | FALSE |
| CHEMBL3883304 | Aurora kinase A/Targeting protein for Xklp2 | O14965|Q9ULW0 | PROTEIN COMPLEX | Homo sapiens | 23 | 24 | 9606 | FALSE |
| CHEMBL3157 | Bradykinin B2 receptor | P30411 | SINGLE PROTEIN | Homo sapiens | 2357 | 3980 | 9606 | FALSE |
| CHEMBL2160 | Nucleoside diphosphate kinase 2 | P22392 | SINGLE PROTEIN | Homo sapiens | 78 | 168 | 9606 | FALSE |
| CHEMBL4523421 | Transcription factor IIIB 90 kDa subunit | Q92994 | SINGLE PROTEIN | Homo sapiens | 1 | 1 | 9606 | FALSE |
| CHEMBL4524008 | Galanin receptor | O60755|O43603|P47211 | PROTEIN FAMILY | Homo sapiens | 1 | 1 | 9606 | FALSE |
| CHEMBL4296044 | Gamma-aminobutyric acid receptor subunit alpha-1/beta-2/delta | P14867|P47870|O14764 | PROTEIN COMPLEX | Homo sapiens | 12 | 28 | 9606 | FALSE |
| CHEMBL3308910 | DNA replication licensing factor MCM2 | P49736 | SINGLE PROTEIN | Homo sapiens | 3 | 21 | 9606 | FALSE |
| CHEMBL2111463 | GABA-B receptor | Q9UBS5|O75899 | PROTEIN COMPLEX | Homo sapiens | 157 | 281 | 9606 | FALSE |
| CHEMBL1937 | Histone deacetylase 2 | Q92769 | SINGLE PROTEIN | Homo sapiens | 3765 | 5068 | 9606 | FALSE |
| CHEMBL3713026 | Killer cell immunoglobulin-like receptor 2DL3 | P43628 | SINGLE PROTEIN | Homo sapiens |  |  | 9606 | FALSE |
| CHEMBL4630877 | Sialic acid-binding Ig-like lectin 8 | Q9NYZ4 | SINGLE PROTEIN | Homo sapiens |  |  | 9606 | FALSE |
| CHEMBL4630724 | p90RSK | P51812|Q15349|Q9UK32|Q15418 | PROTEIN FAMILY | Homo sapiens |  |  | 9606 | FALSE |
| CHEMBL2363043 | Ephrin receptor | P29317|P29323|P54756|P54764|P29322|Q9UF33|Q15375|P54753|P29320|P54762|P54760|P21709|O15197|Q5JZY3 | PROTEIN FAMILY | Homo sapiens |  |  | 9606 | FALSE |
| CHEMBL4665590 | RSPO3-LGR4 | Q9BXY4|Q9BXB1 | PROTEIN-PROTEIN INTERACTION | Homo sapiens |  |  | 9606 | FALSE |
| CHEMBL2364170 | Interferon alpha/beta receptor | P17181|P48551 | PROTEIN COMPLEX | Homo sapiens |  |  | 9606 | FALSE |
| CHEMBL2364156 | T-lymphocyte activation antigen CD86 | P42081 | SINGLE PROTEIN | Homo sapiens |  |  | 9606 | FALSE |
| CHEMBL3542430 | 11-beta-hydroxysteroid dehydrogenase | P80365|P28845 | PROTEIN FAMILY | Homo sapiens |  |  | 9606 | FALSE |
| CHEMBL3988361 | Transferrin receptor | P02786|Q9UP52 | PROTEIN FAMILY | Homo sapiens |  |  | 9606 | FALSE |
| CHEMBL2364160 | Vesicle-associated membrane protein 2 | P63027 | SINGLE PROTEIN | Homo sapiens |  |  | 9606 | FALSE |
| CHEMBL3391661 | Betaine-homocysteine S-methyltransferase | Q93088|Q9H2M3 | PROTEIN FAMILY | Homo sapiens |  |  | 9606 | FALSE |
| CHEMBL4665591 | IL36 receptor | Q9HB29|Q9NPH3 | PROTEIN COMPLEX | Homo sapiens |  |  | 9606 | FALSE |
| CHEMBL3856161 | Interferon alpha | P05013|P32881|P01569|P01566|P01563|P01562|P01567|P01568|P05015|P01570|P01571|P05014 | PROTEIN FAMILY | Homo sapiens |  |  | 9606 | FALSE |
| CHEMBL3580489 | Angiopoietin-2 | O15123 | SINGLE PROTEIN | Homo sapiens |  |  | 9606 | FALSE |
| CHEMBL3713941 | Interleukin-13 receptor subunit alpha-2 | Q14627 | SINGLE PROTEIN | Homo sapiens |  |  | 9606 | FALSE |
| CHEMBL4106174 | Topoisomerase I/II | P11387|P11388 | PROTEIN FAMILY | Homo sapiens | 3 | 8 | 9606 | FALSE |
| CHEMBL4523475 | Taste receptor type 2 member 1 | Q9NYW7 | SINGLE PROTEIN | Homo sapiens | 13 | 37 | 9606 | FALSE |
| CHEMBL4295995 | Tolloid-like protein 2 | Q9Y6L7 | SINGLE PROTEIN | Homo sapiens | 393 | 396 | 9606 | FALSE |
| CHEMBL4523434 | Bifunctional polynucleotide phosphatase/kinase | Q96T60 | SINGLE PROTEIN | Homo sapiens | 5 | 17 | 9606 | FALSE |
| CHEMBL4523490 | Phosphatidylcholine transfer protein | Q9UKL6 | SINGLE PROTEIN | Homo sapiens | 31 | 43 | 9606 | FALSE |
| CHEMBL4105732 | Brefeldin A-inhibited guanine nucleotide-exchange protein 2 | Q9Y6D5 | SINGLE PROTEIN | Homo sapiens | 2 | 2 | 9606 | FALSE |
| CHEMBL4146 | Vitamin D3 receptor-interacting protein | Q9ULK4 | SINGLE PROTEIN | Homo sapiens | 2 | 2 | 9606 | FALSE |
| CHEMBL2431 | Serine/threonine-protein kinase AKT2 | P31751 | SINGLE PROTEIN | Homo sapiens | 3280 | 4365 | 9606 | FALSE |
| CHEMBL210 | Beta-2 adrenergic receptor | P07550 | SINGLE PROTEIN | Homo sapiens | 6648 | 12193 | 9606 | FALSE |
| CHEMBL3023 | Sphingosine kinase 2 | Q9NRA0 | SINGLE PROTEIN | Homo sapiens | 1023 | 1621 | 9606 | FALSE |
| CHEMBL1806 | DNA topoisomerase II alpha | P11388 | SINGLE PROTEIN | Homo sapiens | 3482 | 6638 | 9606 | FALSE |
| CHEMBL217 | Dopamine D2 receptor | P14416 | SINGLE PROTEIN | Homo sapiens | 13103 | 24629 | 9606 | FALSE |
| CHEMBL3831286 | Cardiac myosin | P12883|P13533|A7E2Y1|P08590|P10916|Q01449|P12829 | PROTEIN COMPLEX | Homo sapiens |  |  | 9606 | FALSE |
| CHEMBL4296642 | TEC family kinase | Q08881|P51813|P42680|P42681|Q06187 | PROTEIN FAMILY | Homo sapiens |  |  | 9606 | FALSE |
| CHEMBL4665589 | Cap binding complex | P52298|Q09161 | PROTEIN COMPLEX | Homo sapiens |  |  | 9606 | FALSE |
| CHEMBL4622828 | Bcl-2 mRNA | ENSG00000171791 | NUCLEIC-ACID | Homo sapiens |  |  | 9606 | FALSE |
| CHEMBL4296614 | Bromodomain and extra-terminal motif (BET) | P25440|O60885|Q58F21|Q15059 | PROTEIN FAMILY | Homo sapiens |  |  | 9606 | FALSE |
| CHEMBL2364177 | 2'-deoxyadenosine |  | SMALL MOLECULE | Homo sapiens |  |  | 9606 | FALSE |
| CHEMBL3831284 | Interferon lambda receptor | Q08334|Q8IU57 | PROTEIN COMPLEX | Homo sapiens |  |  | 9606 | FALSE |
| CHEMBL4665592 | Interleukin-15 receptor | P31785|P14784|Q13261 | PROTEIN COMPLEX | Homo sapiens |  |  | 9606 | FALSE |
| CHEMBL4303061 | Sialic acid-binding Ig-like lectin 10 | Q96LC7 | SINGLE PROTEIN | Homo sapiens |  |  | 9606 | FALSE |
| CHEMBL4523586 | E3 ubiquitin ligase TRAF3IP2 | O43734 | SINGLE PROTEIN | Homo sapiens |  |  | 9606 | FALSE |
| CHEMBL3856163 | Tumor-associated calcium signal transducer 2 | P09758 | SINGLE PROTEIN | Homo sapiens |  |  | 9606 | FALSE |
| CHEMBL4630723 | ErbB-2/ErbB-3 heterodimer | P04626|P21860 | PROTEIN COMPLEX | Homo sapiens | 2 | 7 | 9606 | FALSE |
| CHEMBL2095198 | Sulfonylurea receptor 2, Kir6.2 | Q14654|O60706 | PROTEIN COMPLEX | Homo sapiens | 119 | 426 | 9606 | FALSE |
| CHEMBL3107 | Basic fibroblast growth factor | P09038 | SINGLE PROTEIN | Homo sapiens | 150 | 185 | 9606 | FALSE |
| CHEMBL4128 | Tyrosine-protein kinase TIE-2 | Q02763 | SINGLE PROTEIN | Homo sapiens | 2332 | 3409 | 9606 | FALSE |
| CHEMBL3922 | Methionine aminopeptidase 2 | P50579 | SINGLE PROTEIN | Homo sapiens | 1090 | 1524 | 9606 | FALSE |
| CHEMBL2093872 | GABA-A receptor; anion channel | P14867|P47870|P18507|P28472|P48169|Q99928|Q9UN88|P34903|O00591|O14764|Q8N1C3|P18505|P78334|P47869|P31644|Q16445 | PROTEIN COMPLEX GROUP | Homo sapiens | 755 | 991 | 9606 | FALSE |
| CHEMBL2916 | Telomerase reverse transcriptase | O14746 | SINGLE PROTEIN | Homo sapiens | 1212 | 2469 | 9606 | FALSE |
| CHEMBL2364675 | Cytochrome P450 3A | P08684|P20815|Q9HB55|P24462 | PROTEIN FAMILY | Homo sapiens | 95 | 140 | 9606 | FALSE |
| CHEMBL2434 | Interleukin-8 receptor B | P25025 | SINGLE PROTEIN | Homo sapiens | 2306 | 3598 | 9606 | FALSE |
| CHEMBL4523229 | G-protein coupled receptor 1 | P46091 | SINGLE PROTEIN | Homo sapiens | 5 | 5 | 9606 | FALSE |
| CHEMBL5465393 | SOS1-KRAS | Q07889|P01116 | PROTEIN COMPLEX | Homo sapiens | 85 | 121 | 9606 | FALSE |
| CHEMBL3885636 | Serine/threonine-protein kinase PAK 1/PAK 2 | Q13177|Q13153 | PROTEIN FAMILY | Homo sapiens | 18 | 18 | 9606 | FALSE |
| CHEMBL3108647 | Histone-lysine N-methyltransferase SETD2 | Q9BYW2 | SINGLE PROTEIN | Homo sapiens | 96 | 132 | 9606 | FALSE |
| CHEMBL4739686 | Na(+)/H(+) exchange regulatory cofactor NHE-RF2 | Q15599 | SINGLE PROTEIN | Homo sapiens | 4 | 8 | 9606 | FALSE |
| CHEMBL3879857 | Chromodomain Y-like protein 2 | Q8N8U2 | SINGLE PROTEIN | Homo sapiens | 104 | 106 | 9606 | FALSE |
| CHEMBL3885590 | Histone deacetylase 1/Nuclear receptor corepressor 2 | Q13547|Q9Y618 | PROTEIN COMPLEX | Homo sapiens | 6 | 6 | 9606 | FALSE |
| CHEMBL5581 | Ras-related C3 botulinum toxin substrate 2 | P15153 | SINGLE PROTEIN | Homo sapiens | 2 | 2 | 9606 | FALSE |
| CHEMBL4105742 | Chromodomain-helicase-DNA-binding protein 4 | Q14839 | SINGLE PROTEIN | Homo sapiens | 221 | 221 | 9606 | FALSE |
| CHEMBL4105867 | Bifunctional coenzyme A synthase | Q13057 | SINGLE PROTEIN | Homo sapiens | 7 | 7 | 9606 | FALSE |
| CHEMBL4296077 | Transforming growth factor beta-1 proprotein/Mothers against decapentaplegic homolog 3 | P84022|P01137 | PROTEIN-PROTEIN INTERACTION | Homo sapiens | 2 | 6 | 9606 | FALSE |
| CHEMBL3421524 | 6-phosphofructo-2-kinase/fructose-2,6-bisphosphatase 1 | P16118 | SINGLE PROTEIN | Homo sapiens | 38 | 38 | 9606 | FALSE |
| CHEMBL4680052 | Cereblon/Focal adhesion kinase 1 | Q05397|Q96SW2 | PROTEIN-PROTEIN INTERACTION | Homo sapiens | 47 | 131 | 9606 | FALSE |
| CHEMBL4295694 | Alpha-2-HS-glycoprotein | P02765 | SINGLE PROTEIN | Homo sapiens | 1 | 2 | 9606 | FALSE |
| CHEMBL4295671 | DnaJ homolog subfamily A member 2 | O60884 | SINGLE PROTEIN | Homo sapiens | 1 | 1 | 9606 | FALSE |
| CHEMBL4748216 | VHL/Aurora kinase A | O14965|P40337 | PROTEIN-PROTEIN INTERACTION | Homo sapiens | 1 | 1 | 9606 | FALSE |
| CHEMBL4105810 | Dedicator of cytokinesis protein 2 | Q92608 | SINGLE PROTEIN | Homo sapiens | 7 | 8 | 9606 | FALSE |
| CHEMBL4295667 | Neuropilin-2 | O60462 | SINGLE PROTEIN | Homo sapiens | 2 | 2 | 9606 | FALSE |
| CHEMBL4296020 | Eukaryotic translation initiation factor 6 | P56537 | SINGLE PROTEIN | Homo sapiens | 1 | 2 | 9606 | FALSE |
| CHEMBL4296149 | von Hippel-Lindau disease tumor suppressor/Elongin-B/Elongin-C/Bromodomain-containing protein 2 | P25440|P40337|Q15370|Q15369 | PROTEIN-PROTEIN INTERACTION | Homo sapiens | 9 | 24 | 9606 | FALSE |
| CHEMBL4296073 | Lysine-specific demethylase 5A/5B | P29375|Q9UGL1 | PROTEIN FAMILY | Homo sapiens | 88 | 101 | 9606 | FALSE |
| CHEMBL4888446 | DNA damage-binding protein 1/Protein cereblon/E3 ubiquitin-protein ligase Mdm2 | Q00987|Q96SW2|Q16531 | PROTEIN COMPLEX | Homo sapiens | 3 | 4 | 9606 | FALSE |
| CHEMBL5465219 | Cereblon/Leucine-rich repeat serine/threonine-protein kinase 2 | Q5S007|Q96SW2 | PROTEIN-PROTEIN INTERACTION | Homo sapiens | 5 | 12 | 9606 | FALSE |
| CHEMBL4281 | Group IID secretory phospholipase A2 | Q9UNK4 | SINGLE PROTEIN | Homo sapiens | 24 | 24 | 9606 | FALSE |
| CHEMBL4523972 | MMP-2/MMP-9 | P08253|P14780 | SELECTIVITY GROUP | Homo sapiens | 135 | 137 | 9606 | FALSE |
| CHEMBL298 | Cholecystokinin B receptor | P32239 | SINGLE PROTEIN | Homo sapiens | 2737 | 3553 | 9606 | FALSE |
| CHEMBL2096662 | Serotonin 2 receptors; 5-HT2a & 5-HT2c | P28223|P28335 | SELECTIVITY GROUP | Homo sapiens | 710 | 792 | 9606 | FALSE |
| CHEMBL4342 | Group X secretory phospholipase A2 | O15496 | SINGLE PROTEIN | Homo sapiens | 166 | 219 | 9606 | FALSE |
| CHEMBL3514 | LDL-associated phospholipase A2 | Q13093 | SINGLE PROTEIN | Homo sapiens | 554 | 1339 | 9606 | FALSE |
| CHEMBL3691 | Autotaxin | Q13822 | SINGLE PROTEIN | Homo sapiens | 1744 | 2726 | 9606 | FALSE |
| CHEMBL1628482 | Relaxin receptor 2 | Q8WXD0 | SINGLE PROTEIN | Homo sapiens | 369 | 377 | 9606 | FALSE |
| CHEMBL5465401 | B2M-MR1 | P61769|Q95460 | PROTEIN COMPLEX | Homo sapiens | 10 | 13 | 9606 | FALSE |
| CHEMBL4295718 | Plastin-2 | P13796 | SINGLE PROTEIN | Homo sapiens | 2 | 6 | 9606 | FALSE |
| CHEMBL4680043 | Protein argonaute-2 | Q9UKV8 | SINGLE PROTEIN | Homo sapiens | 9 | 21 | 9606 | FALSE |
| CHEMBL5482984 | LC3-B-PDEdelta | O43924|Q9GZQ8 | PROTEIN-PROTEIN INTERACTION | Homo sapiens | 13 | 25 | 9606 | FALSE |
| CHEMBL2029194 | Kinesin heavy chain isoform 5C | O60282 | SINGLE PROTEIN | Homo sapiens | 10 | 11 | 9606 | FALSE |
| CHEMBL2176777 | Poly [ADP-ribose] polymerase 14 | Q460N5 | SINGLE PROTEIN | Homo sapiens | 284 | 395 | 9606 | FALSE |
| CHEMBL5995 | Cytohesin-2 | Q99418 | SINGLE PROTEIN | Homo sapiens | 19 | 52 | 9606 | FALSE |
| CHEMBL4227 | Lipoxin A4 receptor | P25090 | SINGLE PROTEIN | Homo sapiens | 1471 | 3499 | 9606 | FALSE |
| CHEMBL1293193 | Atrial natriuretic factor | P01160 | SINGLE PROTEIN | Homo sapiens | 17 | 17 | 9606 | FALSE |
| CHEMBL3817 | Bcl2-antagonist of cell death (BAD) | Q92934 | SINGLE PROTEIN | Homo sapiens | 140 | 147 | 9606 | FALSE |
| CHEMBL2111326 | Cyclin-dependent kinase 2 and 4 (CDK2 and CDK4) | P11802|P24941 | SELECTIVITY GROUP | Homo sapiens | 16 | 21 | 9606 | FALSE |
| CHEMBL4802068 | Monocarboxylate transporter 2/3 | O60669|O95907 | SELECTIVITY GROUP | Homo sapiens | 2 | 2 | 9606 | FALSE |
| CHEMBL5482993 | RAC1/RAC2/RAC3 | P15153|P60763|P63000 | PROTEIN FAMILY | Homo sapiens | 1 | 1 | 9606 | FALSE |
| CHEMBL4680048 | Acyl-protein thioesterase 1/2 | O75608|O95372 | PROTEIN FAMILY | Homo sapiens | 2 | 2 | 9606 | FALSE |
| CHEMBL5465345 | NACHT, LRR and PYD domains-containing protein 4 | Q96MN2 | SINGLE PROTEIN | Homo sapiens | 1 | 1 | 9606 | FALSE |
| CHEMBL1075144 | Neuromedin-U receptor 2 | Q9GZQ4 | SINGLE PROTEIN | Homo sapiens | 230 | 383 | 9606 | FALSE |
| CHEMBL1628477 | Amiloride-sensitive cation channel 2, neuronal | P78348 | SINGLE PROTEIN | Homo sapiens | 21 | 57 | 9606 | FALSE |
| CHEMBL4295917 | Acidic leucine-rich nuclear phosphoprotein 32 family member B | Q92688 | SINGLE PROTEIN | Homo sapiens | 1 | 2 | 9606 | FALSE |
| CHEMBL4295687 | AP-2 complex subunit alpha-1 | O95782 | SINGLE PROTEIN | Homo sapiens | 1 | 1 | 9606 | FALSE |
| CHEMBL4879 | Fatty acid binding protein intestinal | P12104 | SINGLE PROTEIN | Homo sapiens | 20 | 28 | 9606 | FALSE |
| CHEMBL6010 | Cyclin-dependent kinase 2-associated protein 2 | O75956 | SINGLE PROTEIN | Homo sapiens | 2 | 2 | 9606 | FALSE |
| CHEMBL4627241 | Growth factor receptor-bound protein 2 mRNA | ENSG00000177885 | NUCLEIC-ACID | Homo sapiens |  |  | 9606 | FALSE |
| CHEMBL3580493 | Epithelial cell adhesion molecule | P16422 | SINGLE PROTEIN | Homo sapiens |  |  | 9606 | FALSE |
| CHEMBL5806 | Glycophorin-A | P02724 | SINGLE PROTEIN | Homo sapiens | 5 | 6 | 9606 | FALSE |
| CHEMBL4739700 | Methionine--tRNA ligase, mitochondrial | Q96GW9 | SINGLE PROTEIN | Homo sapiens | 12 | 12 | 9606 | FALSE |
| CHEMBL2241 | Glutathione S-transferase A2 | P09210 | SINGLE PROTEIN | Homo sapiens | 39 | 52 | 9606 | FALSE |
| CHEMBL3631 | Integrin beta-2 | P05107 | SINGLE PROTEIN | Homo sapiens |  |  | 9606 | FALSE |
| CHEMBL5169071 | VHL/SOS2 | P40337|Q07890 | PROTEIN-PROTEIN INTERACTION | Homo sapiens | 1 | 4 | 9606 | FALSE |
| CHEMBL4802020 | AFG3-like protein 2 | Q9Y4W6 | SINGLE PROTEIN | Homo sapiens | 2 | 2 | 9606 | FALSE |
| CHEMBL2189164 | Kv channel-interacting protein 2 | Q9NS61 | SINGLE PROTEIN | Homo sapiens | 5 | 5 | 9606 | FALSE |
| CHEMBL4295879 | Zinc finger CCCH-type antiviral protein 1 | Q7Z2W4 | SINGLE PROTEIN | Homo sapiens | 5 | 5 | 9606 | FALSE |
| CHEMBL3831288 | Selectin | P14151|P16581|P16109 | PROTEIN FAMILY | Homo sapiens |  |  | 9606 | FALSE |
| CHEMBL4105996 | Sjoegren syndrome/scleroderma autoantigen 1 | O60232 | SINGLE PROTEIN | Homo sapiens | 2 | 2 | 9606 | FALSE |
| CHEMBL5578 | Cyclin-dependent kinase 2-associated protein 1 | O14519 | SINGLE PROTEIN | Homo sapiens | 33 | 33 | 9606 | FALSE |
| CHEMBL4523649 | Rho-associated protein kinase 2/Transforming protein RhoA | O75116|P61586 | PROTEIN COMPLEX | Homo sapiens | 1 | 1 | 9606 | FALSE |
| CHEMBL5465227 | VHL/Receptor protein-tyrosine kinase erbB-2 | P04626|P40337 | PROTEIN-PROTEIN INTERACTION | Homo sapiens | 1 | 1 | 9606 | FALSE |
| CHEMBL4296118 | Androgen receptor/Baculoviral IAP repeat-containing protein 2 | P10275|Q13490 | PROTEIN-PROTEIN INTERACTION | Homo sapiens | 20 | 32 | 9606 | FALSE |
| CHEMBL4296150 | von Hippel-Lindau disease tumor suppressor/Elongin-B/Elongin-C/Bromodomain-containing protein 3 | Q15059|P40337|Q15370|Q15369 | PROTEIN-PROTEIN INTERACTION | Homo sapiens | 9 | 22 | 9606 | FALSE |
| CHEMBL4296131 | Cereblon/NAD-dependent protein deacetylase sirtuin-2 | Q8IXJ6|Q96SW2 | PROTEIN-PROTEIN INTERACTION | Homo sapiens | 8 | 35 | 9606 | FALSE |
| CHEMBL4523473 | Taste receptor type 2 member 5 | Q9NYW4 | SINGLE PROTEIN | Homo sapiens | 9 | 11 | 9606 | FALSE |
| CHEMBL2363064 | Monoamine transporter | P31645|Q01959|P23975 | PROTEIN FAMILY | Homo sapiens |  |  | 9606 | FALSE |
| CHEMBL2116 | AMP-activated protein kinase, alpha-2 subunit | P54646 | SINGLE PROTEIN | Homo sapiens | 887 | 1363 | 9606 | FALSE |
| CHEMBL2189122 | DnaJ homolog subfamily A member 1 | P31689 | SINGLE PROTEIN | Homo sapiens | 246 | 246 | 9606 | FALSE |
| CHEMBL4296139 | von Hippel-Lindau disease tumor suppressor/Receptor-interacting serine/threonine-protein kinase 2 | O43353|P40337 | PROTEIN-PROTEIN INTERACTION | Homo sapiens | 5 | 13 | 9606 | FALSE |
| CHEMBL4576 | Homeodomain-interacting protein kinase 2 | Q9H2X6 | SINGLE PROTEIN | Homo sapiens | 1374 | 1698 | 9606 | FALSE |
| CHEMBL3301389 | Focal adhesion kinase 1/vascular endothelial growth factor receptor 3 | P35916|Q05397 | PROTEIN-PROTEIN INTERACTION | Homo sapiens | 2 | 10 | 9606 | FALSE |
| CHEMBL5907 | Serine/threonine-protein kinase LATS2 | Q9NRM7 | SINGLE PROTEIN | Homo sapiens | 534 | 601 | 9606 | FALSE |
| CHEMBL3038457 | AMPK alpha2/beta2/gamma3 | P54646|O43741|Q9UGI9 | PROTEIN COMPLEX | Homo sapiens | 14 | 25 | 9606 | FALSE |
| CHEMBL4295741 | Peroxiredoxin-6 | P30041 | SINGLE PROTEIN | Homo sapiens | 2 | 7 | 9606 | FALSE |
| CHEMBL4295658 | Actin-related protein 2/3 complex subunit 5 | O15511 | SINGLE PROTEIN | Homo sapiens | 2 | 3 | 9606 | FALSE |
| CHEMBL4295965 | TGF-beta-activated kinase 1 and MAP3K7-binding protein 2 | Q9NYJ8 | SINGLE PROTEIN | Homo sapiens | 1 | 1 | 9606 | FALSE |
| CHEMBL2096677 | Phosphoglycerate kinase | P00558|P07205 | PROTEIN FAMILY | Homo sapiens | 252 | 287 | 9606 | FALSE |
| CHEMBL4523124 | tRNA (cytosine(38)-C(5))-methyltransferase | O14717 | SINGLE PROTEIN | Homo sapiens | 70 | 277 | 9606 | FALSE |
| CHEMBL4105820 | Thyroid hormone receptor-associated protein 3 | Q9Y2W1 | SINGLE PROTEIN | Homo sapiens | 177 | 177 | 9606 | FALSE |
| CHEMBL4523970 | MMP-2/MMP-13 | P08253|P45452 | SELECTIVITY GROUP | Homo sapiens | 124 | 128 | 9606 | FALSE |
| CHEMBL4523460 | E3 ubiquitin-protein ligase SMURF2 | Q9HAU4 | SINGLE PROTEIN | Homo sapiens | 35 | 35 | 9606 | FALSE |
| CHEMBL4523752 | Protein cereblon/Bromodomain-containing protein 2 | P25440|Q96SW2 | PROTEIN-PROTEIN INTERACTION | Homo sapiens | 18 | 73 | 9606 | FALSE |
| CHEMBL5389 | Targeting protein for Xklp2 | Q9ULW0 | SINGLE PROTEIN | Homo sapiens | 6 | 6 | 9606 | FALSE |
| CHEMBL4523673 | BCL-6/NCOR2 | Q9Y618|P41182 | PROTEIN-PROTEIN INTERACTION | Homo sapiens | 37 | 49 | 9606 | FALSE |
| CHEMBL5465257 | BAG family molecular chaperone regulator 3 | O95817 | SINGLE PROTEIN | Homo sapiens | 44 | 56 | 9606 | FALSE |
| CHEMBL3714503 | Tubulin alpha-8 chain | Q9NY65 | SINGLE PROTEIN | Homo sapiens | 1 | 1 | 9606 | FALSE |
| CHEMBL4105777 | Galactoside 2-alpha-L-fucosyltransferase 2 | Q10981 | SINGLE PROTEIN | Homo sapiens | 2 | 2 | 9606 | FALSE |
| CHEMBL5169264 | BCL2/BCL2L11 | P10415|O43521 | PROTEIN-PROTEIN INTERACTION | Homo sapiens | 33 | 33 | 9606 | FALSE |
| CHEMBL4523638 | Gamma-aminobutyric acid receptor subunit alpha-1/gamma-2 | P14867|P18507 | PROTEIN COMPLEX | Homo sapiens | 1 | 1 | 9606 | FALSE |
| CHEMBL4296114 | Lysine-specific histone demethylase 1A/REST corepressor | O60341|Q9P2K3 | PROTEIN COMPLEX | Homo sapiens | 5 | 5 | 9606 | FALSE |
| CHEMBL1874 | Sodium-(potassium)-chloride cotransporter 2 | Q13621 | SINGLE PROTEIN | Homo sapiens |  |  | 9606 | FALSE |
| CHEMBL4804257 | Cancer/testis antigen 1 | P78358 | SINGLE PROTEIN | Homo sapiens |  |  | 9606 | FALSE |
| CHEMBL4295976 | Prefoldin subunit 2 | Q9UHV9 | SINGLE PROTEIN | Homo sapiens | 1 | 1 | 9606 | FALSE |
| CHEMBL1628474 | Transient receptor potential cation channel subfamily V member 5 | Q9NQA5 | SINGLE PROTEIN | Homo sapiens | 4 | 4 | 9606 | FALSE |
| CHEMBL2142 | Glutathione S-transferase theta 2 | P0CG29 | SINGLE PROTEIN | Homo sapiens | 2 | 2 | 9606 | FALSE |
| CHEMBL4879535 | Cereblon/Cyclin-dependent kinase 12 | Q9NYV4|Q96SW2 | PROTEIN-PROTEIN INTERACTION | Homo sapiens | 50 | 75 | 9606 | FALSE |
| CHEMBL2109236 | Neuronal acetylcholine receptor; alpha2/beta2 | P17787|Q15822 | PROTEIN COMPLEX | Homo sapiens | 22 | 25 | 9606 | FALSE |
| CHEMBL2111379 | Thrombin and urokinase-type plasminogen activator | P00734|P00749 | SELECTIVITY GROUP | Homo sapiens | 29 | 31 | 9606 | FALSE |
| CHEMBL4105854 | ADP/ATP translocase 3 | P12236 | SINGLE PROTEIN | Homo sapiens | 241 | 241 | 9606 | FALSE |
| CHEMBL5214862 | PR domain zinc finger protein 2 | Q13029 | SINGLE PROTEIN | Homo sapiens | 1 | 1 | 9606 | FALSE |
| CHEMBL4523741 | VHL/Focal adhesion kinase 1 | Q05397|P40337 | PROTEIN-PROTEIN INTERACTION | Homo sapiens | 10 | 53 | 9606 | FALSE |
| CHEMBL4295630 | ADP-ribose glycohydrolase MACROD2 | A1Z1Q3 | SINGLE PROTEIN | Homo sapiens | 6 | 6 | 9606 | FALSE |
| CHEMBL4295832 | Zyxin | Q15942 | SINGLE PROTEIN | Homo sapiens | 1 | 1 | 9606 | FALSE |
| CHEMBL5465287 | 2-oxoisovalerate dehydrogenase subunit beta, mitochondrial | P21953 | SINGLE PROTEIN | Homo sapiens | 1 | 1 | 9606 | FALSE |
| CHEMBL4742315 | Cereblon/E3 SUMO-protein ligase NSE2 | Q96SW2|Q96MF7 | PROTEIN-PROTEIN INTERACTION | Homo sapiens | 1 | 1 | 9606 | FALSE |
| CHEMBL4524133 | Nicotinic Acetylcholine Receptor | P02708|P07510|P11230|P17787|P30926|P32297|P43681|Q07001|Q15822|Q9UGM1|Q15825|P30532|Q04844|P36544|Q9GZZ6|Q05901 | PROTEIN COMPLEX GROUP | Homo sapiens | 2 | 2 | 9606 | FALSE |
| CHEMBL3885524 | Bcl-2-like protein 11/Bcl-2-related protein A1 | O43521|Q16548 | PROTEIN COMPLEX | Homo sapiens | 8 | 12 | 9606 | FALSE |
| CHEMBL2096976 | Peroxisome proliferator-activated receptor gamma/Nuclear receptor corepressor 2 | P37231|Q9Y618 | PROTEIN-PROTEIN INTERACTION | Homo sapiens | 239 | 239 | 9606 | FALSE |
| CHEMBL3542431 | Deiodinase | P49895|Q92813|P55073 | PROTEIN FAMILY | Homo sapiens |  |  | 9606 | FALSE |
| CHEMBL3542434 | Aldehyde dehydrogenase | P51649|P05091|P49189|P48448|P00352|P30838|P47895|P43353|P30837|O94788|P30038 | PROTEIN FAMILY | Homo sapiens |  |  | 9606 | FALSE |
| CHEMBL1907589 | Neuronal acetylcholine receptor; alpha4/beta2 | P17787|P43681 | PROTEIN COMPLEX | Homo sapiens | 1875 | 4043 | 9606 | FALSE |
| CHEMBL3832942 | Tubulin beta | P68371|Q9H4B7|Q13509|P04350|Q3ZCM7|P07437|Q13885|Q9BVA1|Q9BUF5|A6NNZ2 | PROTEIN FAMILY | Homo sapiens | 103 | 181 | 9606 | FALSE |
| CHEMBL4465 | Acyl coenzyme A:cholesterol acyltransferase 2 | O75908 | SINGLE PROTEIN | Homo sapiens | 246 | 288 | 9606 | FALSE |
| CHEMBL1907606 | Mitogen-activated protein kinase; ERK1/ERK2 | P27361|P28482 | PROTEIN FAMILY | Homo sapiens | 508 | 707 | 9606 | FALSE |
| CHEMBL1824 | Receptor protein-tyrosine kinase erbB-2 | P04626 | SINGLE PROTEIN | Homo sapiens | 5577 | 7963 | 9606 | FALSE |
| CHEMBL3831223 | Aminopeptidase | P15144|P55786|Q9H4A4|P53582|Q9UIQ6|Q9ULA0|Q07075|Q9NQW7|P28838|P50579|O43895|Q6P179|Q9NZ08|Q6Q4G3|Q8N6M6|Q6UB28|Q8NDH3|Q9HAU8|Q9NQH7 | PROTEIN FAMILY | Homo sapiens | 1 | 1 | 9606 | FALSE |
| CHEMBL4523974 | Melanocortin receptor 3/5 | P33032|P41968 | SELECTIVITY GROUP | Homo sapiens | 1 | 1 | 9606 | FALSE |
| CHEMBL3885507 | Acetyl-CoA carboxylase | Q13085|O00763 | PROTEIN FAMILY | Homo sapiens | 102 | 106 | 9606 | FALSE |
| CHEMBL4956 | GABA receptor alpha-2 subunit | P47869 | SINGLE PROTEIN | Homo sapiens | 188 | 274 | 9606 | FALSE |
| CHEMBL4630726 | E2F | Q01094|Q14209|O00716|Q16254|Q15329|O75461|Q96AV8|A0AVK6 | PROTEIN FAMILY | Homo sapiens |  |  | 9606 | FALSE |
| CHEMBL3831287 | Tryptophan 5-hydroxylase | Q8IWU9|P17752 | PROTEIN FAMILY | Homo sapiens |  |  | 9606 | FALSE |
| CHEMBL3307226 | Orexin receptor | O43614|O43613 | PROTEIN FAMILY | Homo sapiens |  |  | 9606 | FALSE |
| CHEMBL2363063 | KCNQ (Kv7) potassium channel | P51787|O43526|O43525|Q9NR82|P56696 | PROTEIN FAMILY | Homo sapiens |  |  | 9606 | FALSE |
| CHEMBL3559684 | Neurotrophic tyrosine kinase receptor | P04629|Q16620|Q16288 | PROTEIN FAMILY | Homo sapiens |  |  | 9606 | FALSE |
| CHEMBL3712996 | Blood group Rh(D) polypeptide | Q02161 | SINGLE PROTEIN | Homo sapiens |  |  | 9606 | FALSE |
| CHEMBL2363041 | Sphingosine 1-phosphate receptor | Q9H228|O95136|O95977|Q99500|P21453 | PROTEIN FAMILY | Homo sapiens |  |  | 9606 | FALSE |
| CHEMBL2364154 | Interleukin-23 | P29460|Q9NPF7 | PROTEIN COMPLEX | Homo sapiens |  |  | 9606 | FALSE |
| CHEMBL2364188 | Collagen | P08123|P02452|P02458|P02461|P02462|P20908|P12109|P12107|P39059|P39060|Q17RW2|Q8IZC6|Q2UY09|P08572|P05997|P12110|P13942|Q01955|P25940|P12111|P53420|P29400|A8TX70|Q14031|A6NMZ7 | PROTEIN COMPLEX GROUP | Homo sapiens |  |  | 9606 | FALSE |
| CHEMBL4622826 | Transforming growth factor beta-2 mRNA | ENSG00000092969 | NUCLEIC-ACID | Homo sapiens |  |  | 9606 | FALSE |
| CHEMBL2364187 | Laminin | P25391|P24043|Q16787|Q16363|O15230|P07942|P55268|Q13751|A4D0S4|P11047|Q13753|Q9Y6N6 | PROTEIN COMPLEX GROUP | Homo sapiens |  |  | 9606 | FALSE |
| CHEMBL4630889 | Glypican-3 | P51654 | SINGLE PROTEIN | Homo sapiens |  |  | 9606 | FALSE |
| CHEMBL3712949 | ICOS ligand | O75144 | SINGLE PROTEIN | Homo sapiens |  |  | 9606 | FALSE |
| CHEMBL4187 | Sodium channel protein type II alpha subunit | Q99250 | SINGLE PROTEIN | Homo sapiens | 285 | 511 | 9606 | FALSE |
| CHEMBL1916 | Alpha-2c adrenergic receptor | P18825 | SINGLE PROTEIN | Homo sapiens | 2933 | 5120 | 9606 | FALSE |
| CHEMBL5467 | Bone morphogenetic protein receptor type-2 | Q13873 | SINGLE PROTEIN | Homo sapiens | 554 | 703 | 9606 | FALSE |
| CHEMBL301 | Cyclin-dependent kinase 2 | P24941 | SINGLE PROTEIN | Homo sapiens | 6545 | 9307 | 9606 | FALSE |
| CHEMBL1829 | Histone deacetylase 3 | O15379 | SINGLE PROTEIN | Homo sapiens | 2828 | 3888 | 9606 | FALSE |
| CHEMBL2285 | ADAMTS5 | Q9UNA0 | SINGLE PROTEIN | Homo sapiens | 628 | 711 | 9606 | FALSE |
| CHEMBL2111462 | Thyroid hormone receptor | P10827|P10828 | PROTEIN COMPLEX | Homo sapiens | 127 | 146 | 9606 | FALSE |
| CHEMBL3301398 | Ribonucleotide reductase | P23921|Q7LG56 | PROTEIN COMPLEX | Homo sapiens | 4 | 12 | 9606 | FALSE |
| CHEMBL2096904 | Serotonin (5-HT) receptor | P30939|P41595|Q13639|P28222|P46098|P08908|P28221|P28223|P28335|P28566|P34969|P50406|P47898|A5X5Y0|O95264|Q70Z44|Q8WXA8 | PROTEIN FAMILY | Homo sapiens | 46 | 68 | 9606 | FALSE |
| CHEMBL2094118 | Adrenergic receptor beta | P08588|P07550|P13945 | PROTEIN FAMILY | Homo sapiens | 741 | 1214 | 9606 | FALSE |
| CHEMBL6136 | Lysine-specific histone demethylase 1 | O60341 | SINGLE PROTEIN | Homo sapiens | 2450 | 4338 | 9606 | FALSE |
| CHEMBL4680031 | RNA-binding protein 39 | Q14498 | SINGLE PROTEIN | Homo sapiens | 1 | 9 | 9606 | FALSE |
| CHEMBL2997 | AMP deaminase 2 | Q01433 | SINGLE PROTEIN | Homo sapiens | 68 | 110 | 9606 | FALSE |
| CHEMBL2375204 | Acyl-CoA wax alcohol acyltransferase 2 | Q6E213 | SINGLE PROTEIN | Homo sapiens | 15 | 15 | 9606 | FALSE |
| CHEMBL3883285 | Bcl-xL/BH3-interacting domain death agonist | Q07817|P55957 | PROTEIN-PROTEIN INTERACTION | Homo sapiens | 15 | 15 | 9606 | FALSE |
| CHEMBL2375205 | Acyl-CoA wax alcohol acyltransferase 1 | Q58HT5 | SINGLE PROTEIN | Homo sapiens | 12 | 12 | 9606 | FALSE |
| CHEMBL5291692 | VHL/E3 ubiquitin-protein ligase Mdm2 | Q00987|P40337 | PROTEIN-PROTEIN INTERACTION | Homo sapiens | 1 | 20 | 9606 | FALSE |
| CHEMBL5169181 | Cysteine protease ATG4A | Q8WYN0 | SINGLE PROTEIN | Homo sapiens | 1 | 1 | 9606 | FALSE |
| CHEMBL4295633 | Putative annexin A2-like protein | A6NMY6 | SINGLE PROTEIN | Homo sapiens | 1 | 1 | 9606 | FALSE |
| CHEMBL5465383 | MDM2-HDAC2 | Q00987|Q92769 | PROTEIN-PROTEIN INTERACTION | Homo sapiens | 4 | 20 | 9606 | FALSE |
| CHEMBL3751 | Estrogen-related receptor beta | O95718 | SINGLE PROTEIN | Homo sapiens | 150 | 173 | 9606 | FALSE |
| CHEMBL2375202 | Lymphocyte antigen 96 | Q9Y6Y9 | SINGLE PROTEIN | Homo sapiens | 37 | 117 | 9606 | FALSE |
| CHEMBL5465542 | Lysosome-associated membrane glycoprotein 2 | P13473 | SINGLE PROTEIN | Homo sapiens | 1 | 1 | 9606 | FALSE |
| CHEMBL4524130 | NEK kinase | P51955|Q9HC98|Q8TDX7|Q6P3R8|Q8TD19|Q8NG66|P51956|P51957|Q96PY6|Q86SG6|Q6ZWH5 | PROTEIN FAMILY | Homo sapiens | 1 | 1 | 9606 | FALSE |
| CHEMBL4888459 | STE20-like serine/threonine-protein kinase/Serine/threonine-protein kinase 10 | O94804|Q9H2G2 | PROTEIN FAMILY | Homo sapiens | 1 | 2 | 9606 | FALSE |
| CHEMBL4524128 | Vasoactive intestinal peptide receptor | P41587|P32241|P41586 | PROTEIN FAMILY | Homo sapiens | 1 | 1 | 9606 | FALSE |
| CHEMBL4523927 | Trace amine-associated receptor 2 | Q9P1P5 | SINGLE PROTEIN | Homo sapiens | 1 | 1 | 9606 | FALSE |
| CHEMBL3137268 | Integrin alpha2/beta1 | P05556|P17301 | PROTEIN COMPLEX | Homo sapiens | 63 | 69 | 9606 | FALSE |
| CHEMBL5465255 | Zinc finger protein Helios | Q9UKS7 | SINGLE PROTEIN | Homo sapiens | 31 | 59 | 9606 | FALSE |
| CHEMBL4630750 | E3 ubiquitin-protein ligase XIAP/JAK2 | O60674|P98170 | PROTEIN-PROTEIN INTERACTION | Homo sapiens | 8 | 14 | 9606 | FALSE |
| CHEMBL5169272 | BCL2L10/BID | Q9HD36|P55957 | PROTEIN-PROTEIN INTERACTION | Homo sapiens | 3 | 3 | 9606 | FALSE |
| CHEMBL4523634 | CDK2/Cyclin-Y | P24941|Q8ND76 | PROTEIN COMPLEX | Homo sapiens | 1 | 1 | 9606 | FALSE |
| CHEMBL4630833 | Ubiquitin thioesterase OTU1 | Q5VVQ6 | SINGLE PROTEIN | Homo sapiens | 3 | 3 | 9606 | FALSE |
| CHEMBL3137272 | Nicotinic acetylcholine receptor alpha3/beta2/alpha5 | P17787|P32297|P30532 | PROTEIN COMPLEX | Homo sapiens | 2 | 2 | 9606 | FALSE |
| CHEMBL3885512 | Adenosine receptor A1/Alpha-2A adrenergic receptor | P30542|P08913 | PROTEIN COMPLEX | Homo sapiens | 1 | 1 | 9606 | FALSE |
| CHEMBL4523224 | Aquaporin-2 | P41181 | SINGLE PROTEIN | Homo sapiens | 4 | 7 | 9606 | FALSE |
| CHEMBL2095227 | Vascular endothelial growth factor receptor | P17948|P35916|P35968 | PROTEIN FAMILY | Homo sapiens | 275 | 287 | 9606 | FALSE |
| CHEMBL1250360 | Tumor necrosis factor receptor superfamily member 3 | P36941 | SINGLE PROTEIN | Homo sapiens | 1 | 1 | 9606 | FALSE |
| CHEMBL1804 | Somatostatin receptor 2 | P30874 | SINGLE PROTEIN | Homo sapiens | 1182 | 1527 | 9606 | FALSE |
| CHEMBL2959 | Tyrosine-protein kinase ITK/TSK | Q08881 | SINGLE PROTEIN | Homo sapiens | 2548 | 3758 | 9606 | FALSE |
| CHEMBL2363049 | Epidermal growth factor receptor | P04626|P00533|Q15303|P21860 | PROTEIN FAMILY | Homo sapiens | 157 | 225 | 9606 | FALSE |
| CHEMBL3979 | Peroxisome proliferator-activated receptor delta | Q03181 | SINGLE PROTEIN | Homo sapiens | 3777 | 6483 | 9606 | FALSE |
| CHEMBL2111289 | Dual specificity mitogen-activated protein kinase kinase; MEK1/2 | P36507|Q02750 | PROTEIN FAMILY | Homo sapiens | 263 | 426 | 9606 | FALSE |
| CHEMBL3714029 | Lysyl oxidase homolog 2 | Q9Y4K0 | SINGLE PROTEIN | Homo sapiens | 630 | 834 | 9606 | FALSE |
| CHEMBL6044 | Bcl-2-related protein A1 | Q16548 | SINGLE PROTEIN | Homo sapiens | 174 | 316 | 9606 | FALSE |
| CHEMBL3038461 | Nicotinic acetylcholine receptor alpha4/beta2/alpha5 | P17787|P43681|P30532 | PROTEIN COMPLEX | Homo sapiens | 52 | 114 | 9606 | FALSE |
| CHEMBL2093869 | Integrin alpha-IIb/beta-3 | P05106|P08514 | PROTEIN COMPLEX | Homo sapiens | 2362 | 3528 | 9606 | FALSE |
| CHEMBL2873 | Mitogen-activated protein kinase kinase kinase 10 | Q02779 | SINGLE PROTEIN | Homo sapiens | 347 | 434 | 9606 | FALSE |
| CHEMBL2208 | MAP kinase-activated protein kinase 2 | P49137 | SINGLE PROTEIN | Homo sapiens | 3588 | 4888 | 9606 | FALSE |
| CHEMBL5844 | Glucagon-like peptide 2 receptor | O95838 | SINGLE PROTEIN | Homo sapiens | 192 | 294 | 9606 | FALSE |
| CHEMBL4361 | Induced myeloid leukemia cell differentiation protein Mcl-1 | Q07820 | SINGLE PROTEIN | Homo sapiens | 2899 | 3853 | 9606 | FALSE |
| CHEMBL2094258 | Nuclear factor NF-kappa-B complex | Q00653|P19838|Q04206 | PROTEIN COMPLEX GROUP | Homo sapiens | 1908 | 2351 | 9606 | FALSE |
| CHEMBL2095185 | Cholecystokinin receptor | P32239|P32238 | PROTEIN FAMILY | Homo sapiens | 406 | 506 | 9606 | FALSE |
| CHEMBL3706564 | Liver X receptor | Q13133|P55055 | PROTEIN FAMILY | Homo sapiens | 124 | 311 | 9606 | FALSE |
| CHEMBL4531 | Galectin-3 | P17931 | SINGLE PROTEIN | Homo sapiens | 455 | 729 | 9606 | FALSE |
| CHEMBL1744522 | Endothelial PAS domain-containing protein 1 | Q99814 | SINGLE PROTEIN | Homo sapiens | 262 | 642 | 9606 | FALSE |
| CHEMBL4106159 | AMP-activated protein kinase alpha-2/beta-1/gamma-3 | P54646|Q9Y478|Q9UGI9 | PROTEIN COMPLEX | Homo sapiens | 14 | 15 | 9606 | FALSE |
| CHEMBL2096981 | Cannabinoid receptor | P34972|P21554 | PROTEIN FAMILY | Homo sapiens | 230 | 239 | 9606 | FALSE |
| CHEMBL4295806 | Sodium-dependent phosphate transporter 2 | Q08357 | SINGLE PROTEIN | Homo sapiens | 12 | 12 | 9606 | FALSE |
| CHEMBL2021753 | Kinesin-like protein KIF20A | O95235 | SINGLE PROTEIN | Homo sapiens | 40 | 148 | 9606 | FALSE |
| CHEMBL3559706 | Taste receptor type 2 member 50 | P59544 | SINGLE PROTEIN | Homo sapiens | 10 | 12 | 9606 | FALSE |
| CHEMBL3988599 | Taste receptor type 2 member 8 | Q9NYW2 | SINGLE PROTEIN | Homo sapiens | 329 | 387 | 9606 | FALSE |
| CHEMBL4523254 | Taste receptor type 2 member 30 | P59541 | SINGLE PROTEIN | Homo sapiens | 10 | 12 | 9606 | FALSE |
| CHEMBL5875 | Solute carrier family 2, facilitated glucose transporter member 5 | P22732 | SINGLE PROTEIN | Homo sapiens | 24 | 25 | 9606 | FALSE |
| CHEMBL4523471 | Taste receptor type 2 member 13 | Q9NYV9 | SINGLE PROTEIN | Homo sapiens | 10 | 13 | 9606 | FALSE |
| CHEMBL5885 | Potassium voltage-gated channel subfamily D member 2 | Q9NZV8 | SINGLE PROTEIN | Homo sapiens | 12 | 12 | 9606 | FALSE |
| CHEMBL1628475 | Fatty-acid amide hydrolase 2 | Q6GMR7 | SINGLE PROTEIN | Homo sapiens | 6 | 6 | 9606 | FALSE |
| CHEMBL3112384 | Retinal dehydrogenase 2 | O94788 | SINGLE PROTEIN | Homo sapiens | 190 | 226 | 9606 | FALSE |
| CHEMBL4189 | Homeobox protein Brn-2 | P20265 | SINGLE PROTEIN | Homo sapiens | 4 | 4 | 9606 | FALSE |
| CHEMBL2609 | NAALADase II | Q9Y3Q0 | SINGLE PROTEIN | Homo sapiens | 20 | 20 | 9606 | FALSE |
| CHEMBL5465522 | VHL/MAP kinase p38 alpha | Q16539|P40337 | PROTEIN-PROTEIN INTERACTION | Homo sapiens | 1 | 2 | 9606 | FALSE |
| CHEMBL4106164 | L-type calcium channel alpha-1c/beta-2/alpha2delta-1 | P54289|Q13936|Q08289 | PROTEIN COMPLEX | Homo sapiens | 6 | 14 | 9606 | FALSE |
| CHEMBL4105920 | CCR4-NOT transcription complex subunit 2 | Q9NZN8 | SINGLE PROTEIN | Homo sapiens | 3 | 3 | 9606 | FALSE |
| CHEMBL2646 | Phosphoribosyl pyrophosphate synthetase-associated protein 2 | O60256 | SINGLE PROTEIN | Homo sapiens | 3 | 9 | 9606 | FALSE |
| CHEMBL2816 | Alpha-ketoglutarate dehydrogenase | Q02218 | SINGLE PROTEIN | Homo sapiens | 1 | 4 | 9606 | FALSE |
| CHEMBL4138 | Voltage-gated L-type calcium channel alpha-1D subunit | Q01668 | SINGLE PROTEIN | Homo sapiens | 32 | 39 | 9606 | FALSE |
| CHEMBL3286076 | C-X-C motif chemokine 2 | P19875 | SINGLE PROTEIN | Homo sapiens | 3 | 3 | 9606 | FALSE |
| CHEMBL1741213 | Sentrin-specific protease 7 | Q9BQF6 | SINGLE PROTEIN | Homo sapiens | 989 | 1073 | 9606 | FALSE |
| CHEMBL2096973 | Peptide N-myristoyltransferase | P30419|O60551 | PROTEIN FAMILY | Homo sapiens | 101 | 241 | 9606 | FALSE |
| CHEMBL3861 | Pyruvate dehydrogenase kinase isoform 2 | Q15119 | SINGLE PROTEIN | Homo sapiens | 848 | 925 | 9606 | FALSE |
| CHEMBL3826866 | Chromobox protein homolog 3 | Q13185 | SINGLE PROTEIN | Homo sapiens | 2 | 2 | 9606 | FALSE |
| CHEMBL4523683 | Protein cereblon/Cyclin-dependent kinase 1 | P06493|Q96SW2 | PROTEIN-PROTEIN INTERACTION | Homo sapiens | 5 | 7 | 9606 | FALSE |
| CHEMBL4630845 | Josephin-2 | Q8TAC2 | SINGLE PROTEIN | Homo sapiens | 3 | 3 | 9606 | FALSE |
| CHEMBL4295931 | Prohibitin-2 | Q99623 | SINGLE PROTEIN | Homo sapiens | 54 | 142 | 9606 | FALSE |
| CHEMBL4105890 | Structural maintenance of chromosomes protein 2 | O95347 | SINGLE PROTEIN | Homo sapiens | 241 | 241 | 9606 | FALSE |
| CHEMBL2111477 | Thrombin and coagulation factor VII | P00734|P08709 | SELECTIVITY GROUP | Homo sapiens | 162 | 175 | 9606 | FALSE |
| CHEMBL4523360 | Taste receptor type 2 member 42 | Q7RTR8 | SINGLE PROTEIN | Homo sapiens | 6 | 6 | 9606 | FALSE |
| CHEMBL3240 | Insulin-like growth factor II receptor | P11717 | SINGLE PROTEIN | Homo sapiens | 12 | 20 | 9606 | FALSE |
| CHEMBL2111409 | Vascular endothelial growth factor receptor 2 and 3 (KDR and Flt-4) | P35916|P35968 | SELECTIVITY GROUP | Homo sapiens | 23 | 23 | 9606 | FALSE |
| CHEMBL1293249 | Kruppel-like factor 5 | Q13887 | SINGLE PROTEIN | Homo sapiens | 175 | 178 | 9606 | FALSE |
| CHEMBL4295906 | Solute carrier family 2, facilitated glucose transporter member 14 | Q8TDB8 | SINGLE PROTEIN | Homo sapiens | 1 | 1 | 9606 | FALSE |
| CHEMBL2111397 | Melanocortin receptor (M4 and M5) | P32245|P33032 | SELECTIVITY GROUP | Homo sapiens | 49 | 50 | 9606 | FALSE |
| CHEMBL2420 | Carbonic anhydrase-related protein 2 | O75493 | SINGLE PROTEIN | Homo sapiens | 20 | 23 | 9606 | FALSE |
| CHEMBL4523248 | Taste receptor type 2 member 38 | P59533 | SINGLE PROTEIN | Homo sapiens | 22 | 37 | 9606 | FALSE |
| CHEMBL4523472 | Taste receptor type 2 member 7 | Q9NYW3 | SINGLE PROTEIN | Homo sapiens | 11 | 31 | 9606 | FALSE |
| CHEMBL4630807 | Mitofusin-2 | O95140 | SINGLE PROTEIN | Homo sapiens | 5 | 7 | 9606 | FALSE |
| CHEMBL4523250 | Taste receptor type 2 member 40 | P59535 | SINGLE PROTEIN | Homo sapiens | 8 | 10 | 9606 | FALSE |
| CHEMBL3038456 | AMPK alpha2/beta2/gamma1 | P54646|O43741|P54619 | PROTEIN COMPLEX | Homo sapiens | 60 | 81 | 9606 | FALSE |
| CHEMBL4523255 | Taste receptor type 2 member 19 | P59542 | SINGLE PROTEIN | Homo sapiens | 8 | 20 | 9606 | FALSE |
| CHEMBL3885511 | Adenomatous polyposis coli protein/Transcription factor 7-like 2 | P25054|Q9NQB0 | PROTEIN-PROTEIN INTERACTION | Homo sapiens | 8 | 14 | 9606 | FALSE |
| CHEMBL3159 | Trypsin II | P07478 | SINGLE PROTEIN | Homo sapiens | 14 | 14 | 9606 | FALSE |
| CHEMBL3885633 | Retinoic acid receptor RXR-alpha/gamma | P13631|P19793 | PROTEIN COMPLEX | Homo sapiens | 11 | 11 | 9606 | FALSE |
| CHEMBL2311230 | Spermatogenesis-associated protein 5 | Q8NB90 | SINGLE PROTEIN | Homo sapiens | 3 | 3 | 9606 | FALSE |
| CHEMBL6156 | Tripeptidyl-peptidase 2 | P29144 | SINGLE PROTEIN | Homo sapiens | 1 | 1 | 9606 | FALSE |
| CHEMBL2046260 | Kynurenine--oxoglutarate transaminase 3 | Q6YP21 | SINGLE PROTEIN | Homo sapiens | 8 | 9 | 9606 | FALSE |
| CHEMBL4296066 | Cyclin-dependent kinase 1/Cyclin A1 | P06493|P78396 | PROTEIN COMPLEX | Homo sapiens | 8 | 8 | 9606 | FALSE |
| CHEMBL1250356 | Tumor necrosis factor receptor superfamily member 1B | P20333 | SINGLE PROTEIN | Homo sapiens | 3 | 5 | 9606 | FALSE |
| CHEMBL4680045 | SLC34A2-ROS1 | P08922|O95436 | CHIMERIC PROTEIN | Homo sapiens | 2 | 3 | 9606 | FALSE |
| CHEMBL4739672 | Erlin-2 | O94905 | SINGLE PROTEIN | Homo sapiens | 1 | 1 | 9606 | FALSE |
| CHEMBL4106121 | MAC1-CD40L | P05107|P11215|P29965 | PROTEIN-PROTEIN INTERACTION | Homo sapiens | 6 | 6 | 9606 | FALSE |
| CHEMBL1938216 | Connector enhancer of kinase suppressor of ras 2 | Q8WXI2 | SINGLE PROTEIN | Homo sapiens | 3 | 3 | 9606 | FALSE |
| CHEMBL4802031 | Baculoviral IAP repeat-containing protein 2/Epidermal growth factor receptor | P00533|Q13490 | PROTEIN-PROTEIN INTERACTION | Homo sapiens | 1 | 2 | 9606 | FALSE |
| CHEMBL2692 | Cellular retinoic acid-binding protein II | P29373 | SINGLE PROTEIN | Homo sapiens | 10 | 10 | 9606 | FALSE |
| CHEMBL4523486 | Hydroxyproline dehydrogenase | Q9UF12 | SINGLE PROTEIN | Homo sapiens | 15 | 30 | 9606 | FALSE |
| CHEMBL3347256 | Proteasome subunit beta type-7 | Q99436 | SINGLE PROTEIN | Homo sapiens | 13 | 13 | 9606 | FALSE |
| CHEMBL4524033 | Aspartate aminotransferase, mitochondrial | P00505 | SINGLE PROTEIN | Homo sapiens | 2 | 3 | 9606 | FALSE |
| CHEMBL4630817 | Ubiquitin carboxyl-terminal hydrolase 6 | P35125 | SINGLE PROTEIN | Homo sapiens | 5 | 5 | 9606 | FALSE |
| CHEMBL4523342 | 2',5'-phosphodiesterase 12 | Q6L8Q7 | SINGLE PROTEIN | Homo sapiens | 2 | 2 | 9606 | FALSE |
| CHEMBL4888450 | Protein orai-2/Stromal interaction molecule 1 | Q96SN7|Q13586 | PROTEIN COMPLEX | Homo sapiens | 1 | 1 | 9606 | FALSE |
| CHEMBL1613743 | Neutrophil cytosol factor 1 | P14598 | SINGLE PROTEIN | Homo sapiens | 149 | 348 | 9606 | FALSE |
| CHEMBL4295834 | Dihydropyrimidinase-related protein 2 | Q16555 | SINGLE PROTEIN | Homo sapiens | 2 | 2 | 9606 | FALSE |
| CHEMBL5465554 | Cereblon-IKZF2 | Q96SW2|Q9UKS7 | PROTEIN-PROTEIN INTERACTION | Homo sapiens | 60 | 90 | 9606 | FALSE |
| CHEMBL4295924 | Nuclear receptor-binding factor 2 | Q96F24 | SINGLE PROTEIN | Homo sapiens | 1 | 1 | 9606 | FALSE |
| CHEMBL1795085 | Ataxin-2 | Q99700 | SINGLE PROTEIN | Homo sapiens | 53924 | 54410 | 9606 | FALSE |
| CHEMBL4295826 | Poly(rC)-binding protein 2 | Q15366 | SINGLE PROTEIN | Homo sapiens | 2 | 2 | 9606 | FALSE |
| CHEMBL2349 | Phosphorylase kinase gamma subunit 2 | P15735 | SINGLE PROTEIN | Homo sapiens | 1556 | 2317 | 9606 | FALSE |
| CHEMBL4523864 | Neuropeptides B/W receptor type 2 | P48146 | SINGLE PROTEIN | Homo sapiens | 1 | 1 | 9606 | FALSE |
| CHEMBL4106179 | Ribosomal protein S6 kinase alpha-1/alpha-3 | P51812|Q15418 | PROTEIN FAMILY | Homo sapiens | 1 | 1 | 9606 | FALSE |
| CHEMBL4301 | L-type amino acid transporter 2 | Q9UHI5 | SINGLE PROTEIN | Homo sapiens | 1 | 3 | 9606 | FALSE |
| CHEMBL4296067 | Cyclin-dependent kinase 13/Cyclin-K | Q14004|O75909 | PROTEIN COMPLEX | Homo sapiens | 66 | 70 | 9606 | FALSE |
| CHEMBL4630749 | Baculoviral IAP repeat-containing protein 2/Cyclin-dependent kinase 6 | Q00534|Q13490 | PROTEIN-PROTEIN INTERACTION | Homo sapiens | 3 | 3 | 9606 | FALSE |
| CHEMBL3779761 | Chromobox protein homolog 2 | Q14781 | SINGLE PROTEIN | Homo sapiens | 14 | 14 | 9606 | FALSE |
| CHEMBL1883 | Neuronal acetylcholine receptor protein beta-2 subunit | P17787 | SINGLE PROTEIN | Homo sapiens | 2 | 2 | 9606 | FALSE |
| CHEMBL2331048 | Transmembrane protease serine 4 | Q9NRS4 | SINGLE PROTEIN | Homo sapiens | 81 | 563 | 9606 | FALSE |
| CHEMBL1795105 | Far upstream element-binding protein 2 | Q92945 | SINGLE PROTEIN | Homo sapiens | 4 | 16 | 9606 | FALSE |
| CHEMBL4524012 | P2X receptor | P51575|Q99571|Q9UBL9|O15547|P56373|Q99572|Q93086 | PROTEIN FAMILY | Homo sapiens | 1 | 1 | 9606 | FALSE |
| CHEMBL5465277 | Multifunctional procollagen lysine hydroxylase and glycosyltransferase LH3 | O60568 | SINGLE PROTEIN | Homo sapiens | 6 | 6 | 9606 | FALSE |
| CHEMBL4523678 | Protein cereblon/MAP2K7 | O14733|Q96SW2 | PROTEIN-PROTEIN INTERACTION | Homo sapiens | 1 | 2 | 9606 | FALSE |
| CHEMBL4295738 | Gap junction beta-2 protein | P29033 | SINGLE PROTEIN | Homo sapiens | 35 | 74 | 9606 | FALSE |
| CHEMBL5465322 | Diacylglycerol O-acyltransferase 2-like protein 6 | Q6ZPD8 | SINGLE PROTEIN | Homo sapiens | 1 | 1 | 9606 | FALSE |
| CHEMBL2321632 | Beta-1,3-galactosyl-O-glycosyl-glycoprotein beta-1,6-N-acetylglucosaminyltransferase | Q02742 | SINGLE PROTEIN | Homo sapiens | 3 | 3 | 9606 | FALSE |
| CHEMBL5892 | Endothelin B receptor-like protein 2 | O60883 | SINGLE PROTEIN | Homo sapiens | 2 | 2 | 9606 | FALSE |
| CHEMBL2363011 | Potassium voltage-gated channel subfamily H member 6 | Q9H252 | SINGLE PROTEIN | Homo sapiens | 1 | 1 | 9606 | FALSE |
| CHEMBL5851 | Baculoviral IAP repeat-containing protein 8 | Q96P09 | SINGLE PROTEIN | Homo sapiens | 6 | 6 | 9606 | FALSE |
| CHEMBL3885551 | Cyclin-dependent kinase 1/G1/S-specific cyclin-D1 | P06493|P24385 | PROTEIN COMPLEX | Homo sapiens | 4 | 4 | 9606 | FALSE |
| CHEMBL4524042 | Tribbles homolog 1 | Q96RU8 | SINGLE PROTEIN | Homo sapiens | 1 | 1 | 9606 | FALSE |
| CHEMBL2189112 | Histone-lysine N-methyltransferase MLL4 | Q9UMN6 | SINGLE PROTEIN | Homo sapiens | 9 | 11 | 9606 | FALSE |
| CHEMBL4630842 | OTU domain-containing protein 6A | Q7L8S5 | SINGLE PROTEIN | Homo sapiens | 3 | 3 | 9606 | FALSE |
| CHEMBL2069163 | Dual specificity testis-specific protein kinase 2 | Q96S53 | SINGLE PROTEIN | Homo sapiens | 173 | 173 | 9606 | FALSE |
| CHEMBL2331064 | Phosphatidylinositol 3,4,5-trisphosphate 5-phosphatase 2 | O15357 | SINGLE PROTEIN | Homo sapiens | 83 | 180 | 9606 | FALSE |
| CHEMBL5977 | Galectin-2 | P05162 | SINGLE PROTEIN | Homo sapiens | 44 | 56 | 9606 | FALSE |
| CHEMBL6090 | Actin-related protein 2 | P61160 | SINGLE PROTEIN | Homo sapiens | 250 | 252 | 9606 | FALSE |
| CHEMBL3706565 | Liver X receptor (LXR alpha AND LXR beta) | Q13133|P55055 | SELECTIVITY GROUP | Homo sapiens | 107 | 115 | 9606 | FALSE |
| CHEMBL1615387 | Nuclear receptor coactivator 1 | Q15788 | SINGLE PROTEIN | Homo sapiens | 241 | 241 | 9606 | FALSE |
| CHEMBL3712870 | OX-2 membrane glycoprotein | P41217 | SINGLE PROTEIN | Homo sapiens |  |  | 9606 | FALSE |
| CHEMBL4630578 | Beta-2 adrenergic receptor mRNA | ENSG00000169252 | NUCLEIC-ACID | Homo sapiens |  |  | 9606 | FALSE |
| CHEMBL4804251 | IL22 Receptor | Q08334|Q8N6P7 | PROTEIN COMPLEX | Homo sapiens |  |  | 9606 | FALSE |
| CHEMBL3831290 | Sarcoplasmic/endoplasmic reticulum calcium ATPase | Q93084|O14983|P16615 | PROTEIN FAMILY | Homo sapiens |  |  | 9606 | FALSE |
| CHEMBL4295833 | Cysteine and glycine-rich protein 2 | Q16527 | SINGLE PROTEIN | Homo sapiens | 2 | 2 | 9606 | FALSE |
| CHEMBL4105863 | Bis(5'-nucleosyl)-tetraphosphatase [asymmetrical] | P50583 | SINGLE PROTEIN | Homo sapiens | 1 | 1 | 9606 | FALSE |
| CHEMBL4106175 | Serine/threonine-protein kinase Rac alpha/beta | P31751|P31749 | PROTEIN FAMILY | Homo sapiens | 1 | 1 | 9606 | FALSE |
| CHEMBL3509601 | POU domain, class 2, transcription factor 1 | P14859 | SINGLE PROTEIN | Homo sapiens | 43 | 93 | 9606 | FALSE |
| CHEMBL4523637 | CDK17/Cyclin-Y | Q00537|Q8ND76 | PROTEIN COMPLEX | Homo sapiens | 28 | 30 | 9606 | FALSE |
| CHEMBL5483086 | Glutamate NMDA receptor; GRIN1/GRIN2A/GRIN2B | Q05586|Q12879|Q13224 | PROTEIN COMPLEX | Homo sapiens | 1 | 1 | 9606 | FALSE |
| CHEMBL4079 | G-protein coupled receptor kinase 2 | P25098 | SINGLE PROTEIN | Homo sapiens | 938 | 1062 | 9606 | FALSE |
| CHEMBL4630765 | Sodium channel protein type 9 subunit alpha/beta-1/beta-2 | Q15858|O60939|Q07699 | PROTEIN COMPLEX | Homo sapiens | 46 | 47 | 9606 | FALSE |
| CHEMBL4523612 | Relaxin-3 receptor 1/2 | Q9NSD7|Q8TDU9 | PROTEIN FAMILY | Homo sapiens | 1 | 2 | 9606 | FALSE |
| CHEMBL5996 | Dihydrodipicolinate synthase-like, mitochondrial | Q86XE5 | SINGLE PROTEIN | Homo sapiens | 11 | 14 | 9606 | FALSE |
| CHEMBL4630759 | TEL/KDR | P35968|P41212 | CHIMERIC PROTEIN | Homo sapiens | 9 | 9 | 9606 | FALSE |
| CHEMBL5169075 | VHL/GPER1 | Q99527|P40337 | PROTEIN-PROTEIN INTERACTION | Homo sapiens | 2 | 2 | 9606 | FALSE |
| CHEMBL2094120 | GABA-A receptor; alpha-3/beta-3/gamma-2 | P18507|P28472|P34903 | PROTEIN COMPLEX | Homo sapiens | 740 | 1250 | 9606 | FALSE |
| CHEMBL4296121 | Baculoviral IAP repeat-containing protein 3/Huntingtin | Q13489|P42858 | PROTEIN-PROTEIN INTERACTION | Homo sapiens | 3 | 23 | 9606 | FALSE |
| CHEMBL4742316 | Cereblon/SOSS complex subunit B1 | Q96SW2|Q9BQ15 | PROTEIN-PROTEIN INTERACTION | Homo sapiens | 1 | 1 | 9606 | FALSE |
| CHEMBL3178 | Protein-tyrosine sulfotransferase 2 | O60704 | SINGLE PROTEIN | Homo sapiens | 20 | 23 | 9606 | FALSE |
| CHEMBL3883287 | Bcl-xL/Bcl-2-like protein 11 | Q07817|O43521 | PROTEIN-PROTEIN INTERACTION | Homo sapiens | 50 | 53 | 9606 | FALSE |
| CHEMBL4105889 | Exosome RNA helicase MTR4 | P42285 | SINGLE PROTEIN | Homo sapiens | 243 | 243 | 9606 | FALSE |
| CHEMBL4295982 | Proteasome activator complex subunit 2 | Q9UL46 | SINGLE PROTEIN | Homo sapiens | 1 | 2 | 9606 | FALSE |
| CHEMBL4888452 | MAP2K3/Myc proto-oncogene protein | P46734|P01106 | PROTEIN-PROTEIN INTERACTION | Homo sapiens | 5 | 25 | 9606 | FALSE |
| CHEMBL2375207 | Alpha-1,3-mannosyl-glycoprotein 2-beta-N-acetylglucosaminyltransferase | P26572 | SINGLE PROTEIN | Homo sapiens | 12 | 13 | 9606 | FALSE |
| CHEMBL4296064 | Gamma-aminobutyric acid receptor subunit alpha-2/beta-3 | P28472|P47869 | PROTEIN COMPLEX | Homo sapiens | 1 | 1 | 9606 | FALSE |
| CHEMBL1628478 | Chloride channel protein 2 | P51788 | SINGLE PROTEIN | Homo sapiens |  |  | 9606 | FALSE |
| CHEMBL4879528 | Cereblon/Protein-tyrosine phosphatase 2C | Q06124|Q96SW2 | PROTEIN-PROTEIN INTERACTION | Homo sapiens | 15 | 33 | 9606 | FALSE |
| CHEMBL3721311 | 6-phosphofructo-2-kinase/fructose-2,6-bisphosphatase 4 | Q16877 | SINGLE PROTEIN | Homo sapiens | 19 | 37 | 9606 | FALSE |
| CHEMBL3885558 | Cyclin-dependent kinases regulatory subunit 1/S-phase kinase-associated protein 2 | P61024|Q13309 | PROTEIN-PROTEIN INTERACTION | Homo sapiens | 39 | 95 | 9606 | FALSE |
| CHEMBL5958 | Alpha-1-acid glycoprotein 2 | P19652 | SINGLE PROTEIN | Homo sapiens | 1 | 1 | 9606 | FALSE |
| CHEMBL2442 | Kallikrein 2 | P20151 | SINGLE PROTEIN | Homo sapiens | 21 | 35 | 9606 | FALSE |
| CHEMBL4742286 | Cereblon/MAP2K1/MAP2K2 | P36507|Q02750|Q96SW2 | PROTEIN-PROTEIN INTERACTION | Homo sapiens | 13 | 13 | 9606 | FALSE |
| CHEMBL4295645 | AH receptor-interacting protein | O00170 | SINGLE PROTEIN | Homo sapiens | 1 | 2 | 9606 | FALSE |
| CHEMBL4296019 | DNA mismatch repair protein Msh2 | P43246 | SINGLE PROTEIN | Homo sapiens | 1 | 2 | 9606 | FALSE |
| CHEMBL4295819 | Ubiquitin-associated protein 2-like | Q14157 | SINGLE PROTEIN | Homo sapiens | 1 | 1 | 9606 | FALSE |
| CHEMBL4105952 | Eukaryotic initiation factor 4A-II | Q14240 | SINGLE PROTEIN | Homo sapiens | 9 | 9 | 9606 | FALSE |
| CHEMBL4295751 | ADP-ribosylation factor-like protein 2 | P36404 | SINGLE PROTEIN | Homo sapiens | 1 | 1 | 9606 | FALSE |
| CHEMBL4295656 | Actin-related protein 2/3 complex subunit 1B | O15143 | SINGLE PROTEIN | Homo sapiens | 1 | 1 | 9606 | FALSE |
| CHEMBL4523633 | CDK2/Cyclin-E2 | P24941|O96020 | PROTEIN COMPLEX | Homo sapiens | 5 | 5 | 9606 | FALSE |
| CHEMBL4523504 | Bifunctional UDP-N-acetylglucosamine 2-epimerase/N-acetylmannosamine kinase | Q9Y223 | SINGLE PROTEIN | Homo sapiens | 2 | 2 | 9606 | FALSE |
| CHEMBL4105994 | Programmed cell death protein 6 | O75340 | SINGLE PROTEIN | Homo sapiens | 3 | 3 | 9606 | FALSE |
| CHEMBL4295734 | High mobility group protein B2 | P26583 | SINGLE PROTEIN | Homo sapiens | 1 | 1 | 9606 | FALSE |
| CHEMBL5465343 | Oxysterol-binding protein 2 | Q969R2 | SINGLE PROTEIN | Homo sapiens | 30 | 30 | 9606 | FALSE |
| CHEMBL3885591 | Histone deacetylase 2/Nuclear receptor corepressor 2 | Q92769|Q9Y618 | PROTEIN COMPLEX | Homo sapiens | 6 | 6 | 9606 | FALSE |
| CHEMBL5291693 | VHL/NTMT2 | P40337|Q5VVY1 | PROTEIN-PROTEIN INTERACTION | Homo sapiens | 1 | 2 | 9606 | FALSE |
| CHEMBL1795088 | Beta-arrestin-1 | P49407 | SINGLE PROTEIN | Homo sapiens | 29 | 39 | 9606 | FALSE |
| CHEMBL3430886 | Induced myeloid leukemia cell differentiation protein Mcl-1/BH3-interacting domain death agonist | Q07820|P55957 | PROTEIN-PROTEIN INTERACTION | Homo sapiens | 59 | 64 | 9606 | FALSE |
| CHEMBL3392947 | Adiponectin receptor protein 2 | Q86V24 | SINGLE PROTEIN | Homo sapiens | 1 | 1 | 9606 | FALSE |
| CHEMBL4296119 | Baculoviral IAP repeat-containing protein 2/BCR/ABL | P00519|P11274|Q13490 | PROTEIN-PROTEIN INTERACTION | Homo sapiens | 7 | 34 | 9606 | FALSE |
| CHEMBL2146301 | Small ubiquitin-related modifier 2 | P61956 | SINGLE PROTEIN | Homo sapiens | 46 | 88 | 9606 | FALSE |
| CHEMBL1795148 | Arginase-2, mitochondrial | P78540 | SINGLE PROTEIN | Homo sapiens | 9138 | 9290 | 9606 | FALSE |
| CHEMBL2189153 | Thioredoxin, mitochondrial | Q99757 | SINGLE PROTEIN | Homo sapiens | 8 | 16 | 9606 | FALSE |
| CHEMBL4526 | Ephrin type-A receptor 6 | Q9UF33 | SINGLE PROTEIN | Homo sapiens | 311 | 394 | 9606 | FALSE |
| CHEMBL4630743 | VHL/Histone deacetylase 2 | Q92769|P40337 | PROTEIN-PROTEIN INTERACTION | Homo sapiens | 32 | 83 | 9606 | FALSE |
| CHEMBL1255125 | Segment polarity protein dishevelled homolog DVL-2 | O14641 | SINGLE PROTEIN | Homo sapiens | 5 | 5 | 9606 | FALSE |
| CHEMBL4523584 | Lymphocyte antigen 6E | Q16553 | SINGLE PROTEIN | Homo sapiens |  |  | 9606 | FALSE |
| CHEMBL4879538 | Cereblon/Tumour suppressor p53/oncoprotein Mdm2 | P04637|Q00987|Q96SW2 | PROTEIN-PROTEIN INTERACTION | Homo sapiens | 29 | 52 | 9606 | FALSE |
| CHEMBL5169266 | BCL2/BAD | Q92934|P10415 | PROTEIN-PROTEIN INTERACTION | Homo sapiens | 1 | 1 | 9606 | FALSE |
| CHEMBL2095163 | Peroxisome proliferator-activated receptor gamma/Nuclear receptor coactivator 2 | P37231|Q15596 | PROTEIN-PROTEIN INTERACTION | Homo sapiens | 749 | 749 | 9606 | FALSE |
| CHEMBL5433 | Tryptophan 5-hydroxylase 2 | Q8IWU9 | SINGLE PROTEIN | Homo sapiens | 114 | 116 | 9606 | FALSE |
| CHEMBL1743296 | Sulfotransferase 1C4 | O75897 | SINGLE PROTEIN | Homo sapiens | 8 | 8 | 9606 | FALSE |
| CHEMBL5465206 | Cereblon/Carbonic anhydrase II | P00918|Q96SW2 | PROTEIN-PROTEIN INTERACTION | Homo sapiens | 3 | 19 | 9606 | FALSE |
| CHEMBL5169078 | VHL/KDM1A/HDAC1/RCOR1 | Q13547|O60341|P40337|Q9UKL0 | PROTEIN-PROTEIN INTERACTION | Homo sapiens | 1 | 1 | 9606 | FALSE |
| CHEMBL3407327 | Pantothenate kinase 2, mitochondrial | Q9BZ23 | SINGLE PROTEIN | Homo sapiens | 1 | 1 | 9606 | FALSE |
| CHEMBL3217402 | Rhombotin-2 | P25791 | SINGLE PROTEIN | Homo sapiens | 1 | 1 | 9606 | FALSE |
| CHEMBL4667 | Group III secretory phopholipase A2 | Q9NZ20 | SINGLE PROTEIN | Homo sapiens | 22 | 31 | 9606 | FALSE |
| CHEMBL3713436 | Tumor necrosis factor ligand superfamily member 13 | O75888 | SINGLE PROTEIN | Homo sapiens |  |  | 9606 | FALSE |
| CHEMBL4680051 | Sortilin/Progranulin | Q99523|P28799 | PROTEIN-PROTEIN INTERACTION | Homo sapiens | 34 | 37 | 9606 | FALSE |
| CHEMBL3137262 | LSD1/CoREST complex | O60341|Q9UKL0 | PROTEIN COMPLEX | Homo sapiens | 345 | 444 | 9606 | FALSE |
| CHEMBL3885533 | Cadherin-1/Transcription factor 7-like 2 | Q9NQB0|P12830 | PROTEIN-PROTEIN INTERACTION | Homo sapiens | 19 | 26 | 9606 | FALSE |
| CHEMBL4680050 | Sphingosine kinase 1/2 | Q9NRA0|Q9NYA1 | PROTEIN FAMILY | Homo sapiens | 11 | 28 | 9606 | FALSE |
| CHEMBL3232685 | E3 SUMO-protein ligase CBX4 | O00257 | SINGLE PROTEIN | Homo sapiens | 67 | 69 | 9606 | FALSE |
| CHEMBL4296016 | Nucleolar RNA helicase 2 | Q9NR30 | SINGLE PROTEIN | Homo sapiens | 2 | 2 | 9606 | FALSE |
| CHEMBL4295674 | DnaJ homolog subfamily B member 6 | O75190 | SINGLE PROTEIN | Homo sapiens | 1 | 2 | 9606 | FALSE |
| CHEMBL2169732 | Hydroxyacid oxidase 2 | Q9NYQ3 | SINGLE PROTEIN | Homo sapiens | 54 | 107 | 9606 | FALSE |
| CHEMBL3031 | Kallikrein 11 | Q9UBX7 | SINGLE PROTEIN | Homo sapiens | 7 | 7 | 9606 | FALSE |
| CHEMBL3308 | Caspase-6 | P55212 | SINGLE PROTEIN | Homo sapiens | 1094 | 1290 | 9606 | FALSE |
| CHEMBL4742282 | Cereblon/Small EDRK-rich factor 2 | Q96SW2|P84101 | PROTEIN-PROTEIN INTERACTION | Homo sapiens | 1 | 1 | 9606 | FALSE |
| CHEMBL2095174 | SUMO-activating enzyme | Q9UBE0|Q9UBT2 | PROTEIN COMPLEX | Homo sapiens | 474 | 861 | 9606 | FALSE |
| CHEMBL4748233 | VHL/PARP2 | Q9UGN5|P40337 | PROTEIN-PROTEIN INTERACTION | Homo sapiens | 3 | 6 | 9606 | FALSE |
| CHEMBL4106153 | CDK2/Bovine cyclin A | P24941|P30274 | PROTEIN COMPLEX | Homo sapiens | 36 | 36 | 9606 | FALSE |
| CHEMBL1949490 | Midkine | P21741 | SINGLE PROTEIN | Homo sapiens | 16 | 16 | 9606 | FALSE |
| CHEMBL3509606 | Equilibrative nucleoside transporter 2 | Q14542 | SINGLE PROTEIN | Homo sapiens | 6 | 12 | 9606 | FALSE |
| CHEMBL5482983 | JAK2-STAT3 | O60674|P40763 | PROTEIN COMPLEX | Homo sapiens | 1 | 4 | 9606 | FALSE |
| CHEMBL5034 | GABA-B receptor 2 | O75899 | SINGLE PROTEIN | Homo sapiens |  |  | 9606 | FALSE |
| CHEMBL2316 | Granzyme B | P10144 | SINGLE PROTEIN | Homo sapiens | 45 | 52 | 9606 | FALSE |
| CHEMBL4630884 | Prolow-density lipoprotein receptor-related protein 1 | Q07954 | SINGLE PROTEIN | Homo sapiens |  |  | 9606 | FALSE |
| CHEMBL4296072 | Histone deacetylase 6/3 | O15379|Q9UBN7 | PROTEIN FAMILY | Homo sapiens | 2 | 6 | 9606 | FALSE |
| CHEMBL2154 | Group IIE secretory phospholipase A2 | Q9NZK7 | SINGLE PROTEIN | Homo sapiens | 19 | 20 | 9606 | FALSE |
| CHEMBL4278 | Group IIF secretory phospholipase A2 | Q9BZM2 | SINGLE PROTEIN | Homo sapiens | 15 | 15 | 9606 | FALSE |
| CHEMBL5291602 | NAD-dependent malic enzyme, mitochondrial | P23368 | SINGLE PROTEIN | Homo sapiens | 32 | 32 | 9606 | FALSE |
| CHEMBL2111469 | Dipeptidyl peptidase II and dipeptidyl peptidase IV (DPP2 and DPP4) | P27487|Q9UHL4 | SELECTIVITY GROUP | Homo sapiens | 91 | 94 | 9606 | FALSE |
| CHEMBL3885637 | Serine/threonine-protein kinase PAK 1/PAK 2/PAK 3 | O75914|Q13177|Q13153 | PROTEIN FAMILY | Homo sapiens | 13 | 13 | 9606 | FALSE |
| CHEMBL4802034 | HEC1/NEK2 | P51955|O14777 | PROTEIN-PROTEIN INTERACTION | Homo sapiens | 1 | 2 | 9606 | FALSE |
| CHEMBL2271 | Alpha-L-fucosidase 2 | Q9BTY2 | SINGLE PROTEIN | Homo sapiens | 3 | 3 | 9606 | FALSE |
| CHEMBL3885604 | Mothers against decapentaplegic homolog 2/homolog 3 | P84022|Q15796 | PROTEIN FAMILY | Homo sapiens | 15 | 19 | 9606 | FALSE |
| CHEMBL4523350 | NAD(+) hydrolase SARM1 | Q6SZW1 | SINGLE PROTEIN | Homo sapiens | 47 | 87 | 9606 | FALSE |
| CHEMBL5169164 | DNA oxidative demethylase ALKBH2 | Q6NS38 | SINGLE PROTEIN | Homo sapiens | 18 | 21 | 9606 | FALSE |
| CHEMBL1275210 | Stearoyl-CoA desaturase 5 | Q86SK9 | SINGLE PROTEIN | Homo sapiens | 22 | 22 | 9606 | FALSE |
| CHEMBL3988561 | Human leukocyte antigen DR beta chain | P79483|Q30154|P01911|P13762 | PROTEIN FAMILY | Homo sapiens |  |  | 9606 | FALSE |
| CHEMBL5291677 | Cereblon/FGFR1 | P11362|Q96SW2 | PROTEIN-PROTEIN INTERACTION | Homo sapiens | 6 | 8 | 9606 | FALSE |
| CHEMBL4296084 | Stromal interaction molecule 1 & 2/Calcium release-activated calcium channel protein 1 | Q96D31|Q13586|Q9P246 | PROTEIN COMPLEX | Homo sapiens | 2 | 2 | 9606 | FALSE |
| CHEMBL4879514 | Microtubule-associated proteins 1A/1B light chain 3B | Q9GZQ8 | SINGLE PROTEIN | Homo sapiens | 71 | 126 | 9606 | FALSE |
| CHEMBL3856162 | Low affinity immunoglobulin gamma Fc region receptor III-A | P08637 | SINGLE PROTEIN | Homo sapiens |  |  | 9606 | FALSE |
| CHEMBL2109243 | GABA-A receptor; benzodiazepine site | P14867|P18507|P48169|Q99928|P34903|Q8N1C3|P47869|P31644|Q16445 | PROTEIN COMPLEX GROUP | Homo sapiens | 1 | 3 | 9606 | FALSE |
| CHEMBL2406895 | G protein-activated inward rectifier potassium channel 2 | P48051 | SINGLE PROTEIN | Homo sapiens | 13 | 13 | 9606 | FALSE |
| CHEMBL4056 | Alpha-mannosidase 2A1 | Q16706 | SINGLE PROTEIN | Homo sapiens | 30 | 30 | 9606 | FALSE |
| CHEMBL2331075 | D2-like dopamine receptor | P14416|P35462|P21917 | PROTEIN FAMILY | Homo sapiens |  |  | 9606 | FALSE |
| CHEMBL2849 | Peptide N-myristoyltransferase 2 | O60551 | SINGLE PROTEIN | Homo sapiens | 44 | 44 | 9606 | FALSE |
| CHEMBL4630802 | Interferon-inducible protein AIM2 | O14862 | SINGLE PROTEIN | Homo sapiens | 5 | 13 | 9606 | FALSE |
| CHEMBL3832947 | Calcitonin gene-related peptide | P06881|P10092 | PROTEIN FAMILY | Homo sapiens |  |  | 9606 | FALSE |
| CHEMBL4295823 | Serum paraoxonase/arylesterase 2 | Q15165 | SINGLE PROTEIN | Homo sapiens | 3 | 3 | 9606 | FALSE |
| CHEMBL4106162 | AMP-activated protein kinase alpha-1/beta-2/gamma-3 | O43741|Q13131|Q9UGI9 | PROTEIN COMPLEX | Homo sapiens | 14 | 15 | 9606 | FALSE |
| CHEMBL5291524 | CTP synthase 2 | Q9NRF8 | SINGLE PROTEIN | Homo sapiens | 4 | 4 | 9606 | FALSE |
| CHEMBL4523474 | Taste receptor type 2 member 3 | Q9NYW6 | SINGLE PROTEIN | Homo sapiens | 10 | 12 | 9606 | FALSE |
| CHEMBL2111458 | Casein kinase I | Q9HCP0|P78368|P48730|P49674|P48729 | PROTEIN FAMILY | Homo sapiens | 80 | 83 | 9606 | FALSE |
| CHEMBL5465557 | CCN2-EGFR | P00533|P29279 | PROTEIN-PROTEIN INTERACTION | Homo sapiens | 34 | 68 | 9606 | FALSE |
| CHEMBL5483087 | NRF2-MAFG | Q16236|O15525 | PROTEIN COMPLEX | Homo sapiens | 1 | 1 | 9606 | FALSE |
| CHEMBL5890 | Endothelin-converting enzyme 2 | P0DPD6 | SINGLE PROTEIN | Homo sapiens | 5 | 7 | 9606 | FALSE |
| CHEMBL2364171 | Interferon gamma receptor | P38484|P15260 | PROTEIN COMPLEX | Homo sapiens |  |  | 9606 | FALSE |
| CHEMBL5291946 | BRD8/BRD9 | Q9H8M2|Q9H0E9 | SELECTIVITY GROUP | Homo sapiens | 4 | 8 | 9606 | FALSE |
| CHEMBL4665588 | IL-2R beta/gamma | P31785|P14784 | PROTEIN COMPLEX | Homo sapiens |  |  | 9606 | FALSE |
| CHEMBL2364153 | Interleukin-12 | P29460|P29459 | PROTEIN COMPLEX | Homo sapiens |  |  | 9606 | FALSE |
| CHEMBL2111439 | VEGF-receptor 2 and Fibroblast growth factor receptor 1 | P35968|P11362 | PROTEIN FAMILY | Homo sapiens | 50 | 50 | 9606 | FALSE |
| CHEMBL4295988 | Deoxyribose-phosphate aldolase | Q9Y315 | SINGLE PROTEIN | Homo sapiens | 1 | 2 | 9606 | FALSE |
| CHEMBL4295850 | Histone H2B type 2-F | Q5QNW6 | SINGLE PROTEIN | Homo sapiens | 1 | 1 | 9606 | FALSE |
| CHEMBL4295964 | Solute carrier family 2, facilitated glucose transporter member 8 | Q9NY64 | SINGLE PROTEIN | Homo sapiens | 4 | 5 | 9606 | FALSE |
| CHEMBL2321622 | Ubiquitin-like modifier-activating enzyme 6 | A0AVT1 | SINGLE PROTEIN | Homo sapiens | 4 | 5 | 9606 | FALSE |
| CHEMBL4105743 | General transcription and DNA repair factor IIH helicase subunit XPD | P18074 | SINGLE PROTEIN | Homo sapiens | 243 | 244 | 9606 | FALSE |
| CHEMBL2019 | Type I iodothyronine deiodinase (Type-I 5'-deiodinase) (DIOI) (Type 1 DI) (5DI) | P49895 | SINGLE PROTEIN | Homo sapiens |  |  | 9606 | FALSE |
| CHEMBL2094126 | Cyclin-dependent kinase 2/cyclin E | P24864|P24941|O96020 | PROTEIN COMPLEX | Homo sapiens | 1159 | 1421 | 9606 | FALSE |
| CHEMBL2096978 | Thioredoxin reductase | Q16881|Q9NNW7|Q86VQ6 | PROTEIN FAMILY | Homo sapiens | 124 | 275 | 9606 | FALSE |
| CHEMBL3819 | Lysophosphatidic acid receptor Edg-2 | Q92633 | SINGLE PROTEIN | Homo sapiens | 532 | 843 | 9606 | FALSE |
| CHEMBL248 | Leukocyte elastase | P08246 | SINGLE PROTEIN | Homo sapiens | 5185 | 8215 | 9606 | FALSE |
| CHEMBL5493 | Free fatty acid receptor 2 | O15552 | SINGLE PROTEIN | Homo sapiens | 354 | 602 | 9606 | FALSE |
| CHEMBL2476 | Voltage-gated potassium channel subunit Kv7.2 | O43526 | SINGLE PROTEIN | Homo sapiens | 199 | 339 | 9606 | FALSE |
| CHEMBL2364167 | Interleukin-2 receptor | P01589|P31785|P14784 | PROTEIN COMPLEX | Homo sapiens |  |  | 9606 | FALSE |
| CHEMBL2363042 | DNA polymerase (alpha/delta/epsilon) | P09884|Q9HCU8|Q15054|P28340|P49005|Q07864|Q14181|P49642|P49643|P56282|Q9NRF9 | PROTEIN FAMILY | Homo sapiens |  |  | 9606 | FALSE |
| CHEMBL4680040 | Mitochondrial 2-oxodicarboxylate carrier | Q9BQT8 | SINGLE PROTEIN | Homo sapiens | 2 | 2 | 9606 | FALSE |
| CHEMBL2390810 | Microtubule-associated protein 2 | P11137 | SINGLE PROTEIN | Homo sapiens | 50 | 50 | 9606 | FALSE |
| CHEMBL5465242 | VHL-Baculoviral IAP repeat-containing protein 3 | Q13489|P40337 | PROTEIN-PROTEIN INTERACTION | Homo sapiens | 17 | 26 | 9606 | FALSE |
| CHEMBL3430881 | PI3-kinase subunit gamma/Phosphoinositide 3-kinase regulatory subunit 5 | P48736|Q8WYR1 | PROTEIN COMPLEX | Homo sapiens | 10 | 11 | 9606 | FALSE |
| CHEMBL4296285 | Gamma-crystallin C | P07315 | SINGLE PROTEIN | Homo sapiens | 8 | 41 | 9606 | FALSE |
| CHEMBL4804254 | IL-10 receptor | Q08334|Q13651 | PROTEIN COMPLEX | Homo sapiens |  |  | 9606 | FALSE |
| CHEMBL1991 | Inhibitor of nuclear factor kappa B kinase beta subunit | O14920 | SINGLE PROTEIN | Homo sapiens | 3522 | 5618 | 9606 | FALSE |
| CHEMBL2221348 | Voltage-gated potassium channel, KQT; KCNQ2(Kv7.2)/KCNQ3(Kv7.3) | O43526|O43525 | PROTEIN COMPLEX | Homo sapiens | 246 | 375 | 9606 | FALSE |
| CHEMBL230 | Cyclooxygenase-2 | P35354 | SINGLE PROTEIN | Homo sapiens | 8694 | 14150 | 9606 | FALSE |
| CHEMBL1825 | TNF-alpha | P01375 | SINGLE PROTEIN | Homo sapiens | 1175 | 1907 | 9606 | FALSE |
| CHEMBL2886 | Phosphoglycerate kinase 1 | P00558 | SINGLE PROTEIN | Homo sapiens | 94 | 94 | 9606 | FALSE |
| CHEMBL4879493 | Mucolipin-2 | Q8IZK6 | SINGLE PROTEIN | Homo sapiens | 38 | 60 | 9606 | FALSE |
| CHEMBL5169268 | BCL2A1/BID | Q16548|P55957 | PROTEIN-PROTEIN INTERACTION | Homo sapiens | 6 | 6 | 9606 | FALSE |
| CHEMBL2111324 | Phosphorylase kinase | P15735|P46020|P46019|Q93100|Q16816 | PROTEIN COMPLEX GROUP | Homo sapiens | 49 | 51 | 9606 | FALSE |
| CHEMBL1075145 | Transitional endoplasmic reticulum ATPase | P55072 | SINGLE PROTEIN | Homo sapiens | 699 | 1008 | 9606 | FALSE |
| CHEMBL3611 | Type III iodothyronine deiodinase | P55073 | SINGLE PROTEIN | Homo sapiens | 9 | 17 | 9606 | FALSE |
| CHEMBL4106160 | N-type calcium channel alpha-1b/alpha2delta-1/beta-1b | P54289|Q00975|Q02641 | PROTEIN COMPLEX | Homo sapiens | 50 | 50 | 9606 | FALSE |
| CHEMBL2109234 | Neuronal acetylcholine receptor; alpha3/beta2 | P17787|P32297 | PROTEIN COMPLEX | Homo sapiens | 123 | 156 | 9606 | FALSE |
| CHEMBL4523422 | NEDD8-conjugating enzyme UBE2F | Q969M7 | SINGLE PROTEIN | Homo sapiens | 1 | 2 | 9606 | FALSE |
| CHEMBL3111 | Ribosomal protein S6 kinase 2 | Q9UBS0 | SINGLE PROTEIN | Homo sapiens | 89 | 111 | 9606 | FALSE |
| CHEMBL3901 | Sarcoplasmic/endoplasmic reticulum calcium ATPase 2 | P16615 | SINGLE PROTEIN | Homo sapiens | 55 | 63 | 9606 | FALSE |
| CHEMBL5026 | Serine/threonine-protein kinase EEF2K | O00418 | SINGLE PROTEIN | Homo sapiens | 988 | 1281 | 9606 | FALSE |
| CHEMBL4677 | Apoptosis regulator Bcl-W | Q92843 | SINGLE PROTEIN | Homo sapiens | 108 | 122 | 9606 | FALSE |
| CHEMBL4898 | Neurotrophic tyrosine kinase receptor type 2 | Q16620 | SINGLE PROTEIN | Homo sapiens | 2522 | 3363 | 9606 | FALSE |
| CHEMBL3038467 | CDK1/Cyclin A | P06493|P20248 | PROTEIN COMPLEX | Homo sapiens | 141 | 152 | 9606 | FALSE |
| CHEMBL1795172 | Potassium/sodium hyperpolarization-activated cyclic nucleotide-gated channel 2 | Q9UL51 | SINGLE PROTEIN | Homo sapiens | 7 | 7 | 9606 | FALSE |
| CHEMBL3414409 | Macrophage-expressed gene 1 protein | Q2M385 | SINGLE PROTEIN | Homo sapiens | 50 | 83 | 9606 | FALSE |
| CHEMBL4523736 | VHL/Tyrosine-protein kinase ABL2 | P42684|P40337 | PROTEIN-PROTEIN INTERACTION | Homo sapiens | 1 | 1 | 9606 | FALSE |
| CHEMBL3351198 | Protein phosphatase Slingshot homolog 2 | Q76I76 | SINGLE PROTEIN | Homo sapiens | 4 | 4 | 9606 | FALSE |
| CHEMBL5465239 | VHL/Leucine-rich repeat serine/threonine-protein kinase 2 | Q5S007|P40337 | PROTEIN-PROTEIN INTERACTION | Homo sapiens | 4 | 12 | 9606 | FALSE |
| CHEMBL2952 | Glutamate decarboxylase 65 kDa isoform | Q05329 | SINGLE PROTEIN | Homo sapiens | 2 | 2 | 9606 | FALSE |
| CHEMBL1961790 | COUP transcription factor 2 | P24468 | SINGLE PROTEIN | Homo sapiens | 234 | 234 | 9606 | FALSE |
| CHEMBL5465201 | Cereblon/Aurora kinase A | O14965|Q96SW2 | PROTEIN-PROTEIN INTERACTION | Homo sapiens | 13 | 32 | 9606 | FALSE |
| CHEMBL5728 | Cyclin-dependent kinase-like 2 | Q92772 | SINGLE PROTEIN | Homo sapiens | 260 | 280 | 9606 | FALSE |
| CHEMBL3038478 | Dopamine receptor D2L/neurotensin receptor NTS1 | P14416|P30989 | PROTEIN COMPLEX | Homo sapiens | 1 | 1 | 9606 | FALSE |
| CHEMBL4296261 | YEATS domain-containing protein 2 | Q9ULM3 | SINGLE PROTEIN | Homo sapiens | 16 | 26 | 9606 | FALSE |
| CHEMBL1697668 | Solute carrier organic anion transporter family member 1B1 | Q9Y6L6 | SINGLE PROTEIN | Homo sapiens | 2210 | 2675 | 9606 | FALSE |
| CHEMBL5284 | CaM-kinase kinase beta | Q96RR4 | SINGLE PROTEIN | Homo sapiens | 947 | 1378 | 9606 | FALSE |
| CHEMBL1628465 | Transient receptor potential cation channel subfamily V member 6 | Q9H1D0 | SINGLE PROTEIN | Homo sapiens | 75 | 136 | 9606 | FALSE |
| CHEMBL1293275 | Eyes absent homolog 2 | O00167 | SINGLE PROTEIN | Homo sapiens | 5756 | 5884 | 9606 | FALSE |
| CHEMBL240 | HERG | Q12809 | SINGLE PROTEIN | Homo sapiens | 23427 | 30567 | 9606 | FALSE |
| CHEMBL2096682 | Sodium channel alpha subunits; brain (Types I, II, III) | P35498|Q99250|Q9NY46 | PROTEIN FAMILY | Homo sapiens | 212 | 374 | 9606 | FALSE |
| CHEMBL2543 | Casein kinase I gamma 2 | P78368 | SINGLE PROTEIN | Homo sapiens | 1750 | 2578 | 9606 | FALSE |
| CHEMBL3218 | CD22 | P20273 | SINGLE PROTEIN | Homo sapiens | 90 | 125 | 9606 | FALSE |
| CHEMBL5814 | Peroxisomal multifunctional enzyme type 2 | P51659 | SINGLE PROTEIN | Homo sapiens | 117 | 123 | 9606 | FALSE |
| CHEMBL5603 | Nuclear receptor subfamily 0 group B member 2 | Q15466 | SINGLE PROTEIN | Homo sapiens | 18 | 24 | 9606 | FALSE |
| CHEMBL2331065 | Alpha-ketoglutarate-dependent dioxygenase FTO | Q9C0B1 | SINGLE PROTEIN | Homo sapiens | 391 | 563 | 9606 | FALSE |
| CHEMBL2096670 | Glutamate receptor ionotropic AMPA | P48058|P42263|P42262|P42261 | PROTEIN COMPLEX GROUP | Homo sapiens | 193 | 284 | 9606 | FALSE |
| CHEMBL3309110 | Taste receptor type 2 member 9 | Q9NYW1 | SINGLE PROTEIN | Homo sapiens | 11 | 14 | 9606 | FALSE |
| CHEMBL4296428 | HK-2 |  | CELL-LINE | Homo sapiens | 207 | 297 | 9606 | FALSE |
| CHEMBL5049 | Ectonucleoside triphosphate diphosphohydrolase 2 | Q9Y5L3 | SINGLE PROTEIN | Homo sapiens | 86 | 117 | 9606 | FALSE |
| CHEMBL4523697 | Protein cereblon/CDK9 | P50750|Q96SW2 | PROTEIN-PROTEIN INTERACTION | Homo sapiens | 73 | 170 | 9606 | FALSE |
| CHEMBL4630860 | Phospholipase A and acyltransferase 2 | Q9NWW9 | SINGLE PROTEIN | Homo sapiens | 56 | 67 | 9606 | FALSE |
| CHEMBL2086 | Voltage-gated potassium channel subunit Kv1.2 | P16389 | SINGLE PROTEIN | Homo sapiens | 27 | 28 | 9606 | FALSE |
| CHEMBL5167 | Sodium channel protein type XI alpha subunit | Q9UI33 | SINGLE PROTEIN | Homo sapiens | 13 | 15 | 9606 | FALSE |
| CHEMBL4816 | Serine/threonine-protein kinase AKT3 | Q9Y243 | SINGLE PROTEIN | Homo sapiens | 2297 | 3209 | 9606 | FALSE |
| CHEMBL4523488 | Inositol hexakisphosphate kinase 2 | Q9UHH9 | SINGLE PROTEIN | Homo sapiens | 66 | 89 | 9606 | FALSE |
| CHEMBL5704 | Elongation of very long chain fatty acids protein 6 | Q9H5J4 | SINGLE PROTEIN | Homo sapiens | 131 | 140 | 9606 | FALSE |
| CHEMBL5069364 | Transcription regulator protein BACH2 | Q9BYV9 | SINGLE PROTEIN | Homo sapiens | 1 | 9 | 9606 | FALSE |
| CHEMBL4523206 | Endothelial transcription factor GATA-2 | P23769 | SINGLE PROTEIN | Homo sapiens | 1 | 1 | 9606 | FALSE |
| CHEMBL5058623 | SK-N-BE(2) |  | CELL-LINE | Homo sapiens | 35 | 49 | 9606 | FALSE |
| CHEMBL4884 | Caspase-2 | P42575 | SINGLE PROTEIN | Homo sapiens | 112 | 176 | 9606 | FALSE |
| CHEMBL4630850 | Phospholipase A and acyltransferase 5 | Q96KN8 | SINGLE PROTEIN | Homo sapiens | 56 | 67 | 9606 | FALSE |
| CHEMBL5942 | Phosphatidylcholine-sterol acyltransferase | P04180 | SINGLE PROTEIN | Homo sapiens | 148 | 155 | 9606 | FALSE |
| CHEMBL614929 | SL-2 |  | CELL-LINE | Homo sapiens | 15 | 16 | 9606 | FALSE |
| CHEMBL3883294 | Glutamate receptor AMPA 1/2 | P42262|P42261 | PROTEIN COMPLEX | Homo sapiens | 12 | 26 | 9606 | FALSE |
| CHEMBL2321644 | Histone-lysine N-methyltransferase SUV420H2 | Q86Y97 | SINGLE PROTEIN | Homo sapiens | 49 | 60 | 9606 | FALSE |
| CHEMBL2062349 | RuvB-like 2 | Q9Y230 | SINGLE PROTEIN | Homo sapiens | 43 | 47 | 9606 | FALSE |
| CHEMBL5750 | Histone acetyltransferase KAT5 | Q92993 | SINGLE PROTEIN | Homo sapiens | 45 | 90 | 9606 | FALSE |
| CHEMBL4523712 | MCL1/Protein cereblon | Q07820|Q96SW2 | PROTEIN-PROTEIN INTERACTION | Homo sapiens | 22 | 136 | 9606 | FALSE |
| CHEMBL5023 | p53-binding protein Mdm-2 | Q00987 | SINGLE PROTEIN | Homo sapiens | 3160 | 4678 | 9606 | FALSE |
| CHEMBL5169273 | VHL/KRAS | P01116|P40337 | PROTEIN-PROTEIN INTERACTION | Homo sapiens | 16 | 69 | 9606 | FALSE |
| CHEMBL2094121 | GABA-A receptor; alpha-1/beta-3/gamma-2 | P14867|P18507|P28472 | PROTEIN COMPLEX | Homo sapiens | 904 | 1569 | 9606 | FALSE |
| CHEMBL5483184 | CDK15/Cyclin Y | Q96Q40|Q8ND76 | PROTEIN COMPLEX | Homo sapiens | 4 | 4 | 9606 | FALSE |
| CHEMBL2111328 | Inhibitor of NF-kappa-B kinase (IKK) | O14920|O15111|Q9Y6K9 | PROTEIN COMPLEX | Homo sapiens | 35 | 78 | 9606 | FALSE |
| CHEMBL1867 | Alpha-2a adrenergic receptor | P08913 | SINGLE PROTEIN | Homo sapiens | 4190 | 9766 | 9606 | FALSE |
| CHEMBL2073693 | Solute carrier family 22 member 5 | O76082 | SINGLE PROTEIN | Homo sapiens | 77 | 138 | 9606 | FALSE |
| CHEMBL2094128 | Cyclin-dependent kinase 2/cyclin A | P24941|P20248|P78396 | PROTEIN COMPLEX | Homo sapiens | 1906 | 2251 | 9606 | FALSE |
| CHEMBL614206 | NTERA-2-cl-D1 |  | CELL-LINE | Homo sapiens | 162 | 234 | 9606 | FALSE |
| CHEMBL1293289 | Bromodomain-containing protein 2 | P25440 | SINGLE PROTEIN | Homo sapiens | 741 | 1422 | 9606 | FALSE |
| CHEMBL3774290 | Sodium bicarbonate cotransporter 3 | Q9Y6M7 | SINGLE PROTEIN | Homo sapiens | 3 | 3 | 9606 | FALSE |
| CHEMBL4523616 | RPS6KA1/RPS6KA2/RPS6KA3 | P51812|Q15349|Q15418 | PROTEIN FAMILY | Homo sapiens | 2 | 8 | 9606 | FALSE |
| CHEMBL4630748 | Baculoviral IAP repeat-containing protein 2/Cyclin-dependent kinase 4 | P11802|Q13490 | PROTEIN-PROTEIN INTERACTION | Homo sapiens | 3 | 5 | 9606 | FALSE |
| CHEMBL2311041 | 3-phosphoinositide dependent protein kinase-2, PDK2 |  | UNKNOWN | Homo sapiens | 53 | 54 | 9606 | FALSE |
| CHEMBL3739248 | Ubiquitin carboxyl-terminal hydrolase 17 | Q6R6M4 | SINGLE PROTEIN | Homo sapiens | 5 | 5 | 9606 | FALSE |
| CHEMBL2073703 | Solute carrier organic anion transporter family member 2A1 | Q92959 | SINGLE PROTEIN | Homo sapiens | 15 | 17 | 9606 | FALSE |
| CHEMBL1697669 | Cadherin-2 | P19022 | SINGLE PROTEIN | Homo sapiens | 3 | 7 | 9606 | FALSE |
| CHEMBL4296308 | Rhodopsin | P08100 | SINGLE PROTEIN | Homo sapiens | 2 | 2 | 9606 | FALSE |
| CHEMBL4772 | 1-acylglycerol-3-phosphate O-acyltransferase beta | O15120 | SINGLE PROTEIN | Homo sapiens | 125 | 135 | 9606 | FALSE |
| CHEMBL2157858 | Dual specificity protein phosphatase 2 | Q05923 | SINGLE PROTEIN | Homo sapiens | 1 | 1 | 9606 | FALSE |
| CHEMBL3509592 | Diamine acetyltransferase 2 | Q96F10 | SINGLE PROTEIN | Homo sapiens | 6 | 15 | 9606 | FALSE |
| CHEMBL4523720 | GSPT1/E3 ubiquitin-protein ligase Mdm2 | Q00987|P15170 | PROTEIN-PROTEIN INTERACTION | Homo sapiens | 1 | 1 | 9606 | FALSE |
| CHEMBL3079 | Thiamine transporter 1 | O60779 | SINGLE PROTEIN | Homo sapiens | 1 | 2 | 9606 | FALSE |
| CHEMBL4523433 | Potassium channel subfamily K member 17 | Q96T54 | SINGLE PROTEIN | Homo sapiens | 2 | 2 | 9606 | FALSE |
| CHEMBL3885592 | Histone deacetylase complex subunit SAP130/Splicing factor 3B subunit 1/Splicing factor 3B subunit 2 | Q13435|Q9H0E3|O75533 | PROTEIN COMPLEX | Homo sapiens | 3 | 4 | 9606 | FALSE |
| CHEMBL3317336 | Voltage-dependent L-type calcium channel subunit beta-2 | Q08289 | SINGLE PROTEIN | Homo sapiens | 1 | 1 | 9606 | FALSE |
| CHEMBL3885547 | Cullin-1/S-phase kinase-associated protein 1 | Q13616|P63208 | PROTEIN-PROTEIN INTERACTION | Homo sapiens | 1 | 1 | 9606 | FALSE |
| CHEMBL3831 | MAP/microtubule affinity-regulating kinase 2 | Q7KZI7 | SINGLE PROTEIN | Homo sapiens | 1790 | 2588 | 9606 | FALSE |
| CHEMBL614735 | HEp-2 |  | CELL-LINE | Homo sapiens | 2705 | 3911 | 9606 | FALSE |
| CHEMBL3290 | Ephrin type-B receptor 2 | P29323 | SINGLE PROTEIN | Homo sapiens | 1197 | 1939 | 9606 | FALSE |
| CHEMBL1935 | Aldehyde dehydrogenase | P05091 | SINGLE PROTEIN | Homo sapiens | 373 | 520 | 9606 | FALSE |
| CHEMBL3764 | Urotensin II receptor | Q9UKP6 | SINGLE PROTEIN | Homo sapiens | 740 | 1389 | 9606 | FALSE |
| CHEMBL2096667 | c-Jun N-terminal kinase, JNK | P45983|P53779|P45984 | PROTEIN FAMILY | Homo sapiens | 555 | 691 | 9606 | FALSE |
| CHEMBL1919 | Voltage-gated calcium channel alpha2/delta subunit 1 | P54289 | SINGLE PROTEIN | Homo sapiens | 260 | 266 | 9606 | FALSE |
| CHEMBL253 | Cannabinoid CB2 receptor | P34972 | SINGLE PROTEIN | Homo sapiens | 9744 | 17251 | 9606 | FALSE |
| CHEMBL2096909 | Interleukin-8 receptors, CXCR1/CXCR2 | P25025|P25024 | PROTEIN FAMILY | Homo sapiens | 241 | 285 | 9606 | FALSE |
| CHEMBL2096990 | Monoamine transporters; Norepinephrine & dopamine | Q01959|P23975 | SELECTIVITY GROUP | Homo sapiens | 174 | 216 | 9606 | FALSE |
| CHEMBL3831261 | Hypoxia-inducible factor prolyl hydroxylase | Q96KS0|Q9NXG6|Q9GZT9|Q9H6Z9 | PROTEIN FAMILY | Homo sapiens | 23 | 45 | 9606 | FALSE |
| CHEMBL4018 | Neuropeptide Y receptor type 2 | P49146 | SINGLE PROTEIN | Homo sapiens | 2233 | 3775 | 9606 | FALSE |
| CHEMBL2695 | Focal adhesion kinase 1 | Q05397 | SINGLE PROTEIN | Homo sapiens | 3773 | 4850 | 9606 | FALSE |
| CHEMBL4599 | Tyrosine kinase non-receptor protein 2 | Q07912 | SINGLE PROTEIN | Homo sapiens | 1992 | 2932 | 9606 | FALSE |
| CHEMBL5748 | Canalicular multispecific organic anion transporter 1 | Q92887 | SINGLE PROTEIN | Homo sapiens | 877 | 1191 | 9606 | FALSE |
| CHEMBL3906 | Ribosomal protein S6 kinase alpha 2 | Q15349 | SINGLE PROTEIN | Homo sapiens | 1272 | 2261 | 9606 | FALSE |
| CHEMBL3401 | Pregnane X receptor | O75469 | SINGLE PROTEIN | Homo sapiens | 3548 | 6901 | 9606 | FALSE |
| CHEMBL5209636 | Sodium/potassium/calcium exchanger 4 | Q8NFF2 | SINGLE PROTEIN | Homo sapiens | 1 | 1 | 9606 | FALSE |
| CHEMBL3885634 | S-phase kinase-associated protein 1/2 | Q13309|P63208 | PROTEIN-PROTEIN INTERACTION | Homo sapiens | 1 | 1 | 9606 | FALSE |
| CHEMBL3712947 | Solute carrier family 13 member 3 | Q8WWT9 | SINGLE PROTEIN | Homo sapiens | 6 | 7 | 9606 | FALSE |
| CHEMBL1229011 | Splicing factor 3B subunit 2 | Q13435 | SINGLE PROTEIN | Homo sapiens | 4 | 4 | 9606 | FALSE |
| CHEMBL3807 | T-cell protein-tyrosine phosphatase | P17706 | SINGLE PROTEIN | Homo sapiens | 1204 | 1344 | 9606 | FALSE |
| CHEMBL3988640 | Voltage-dependent P/Q-type calcium channel alpha1A/alpha2delta/beta1b | P54289|O00555|Q02641 | PROTEIN COMPLEX | Homo sapiens | 6 | 6 | 9606 | FALSE |
| CHEMBL614725 | MIA PaCa-2 |  | CELL-LINE | Homo sapiens | 4005 | 6432 | 9606 | FALSE |
| CHEMBL4523285 | Aminoacyl tRNA synthase complex-interacting multifunctional protein 2 | Q13155 | SINGLE PROTEIN | Homo sapiens | 72 | 109 | 9606 | FALSE |
| CHEMBL2275 | Sorbitol dehydrogenase | Q00796 | SINGLE PROTEIN | Homo sapiens | 118 | 133 | 9606 | FALSE |
| CHEMBL4608 | Melanocortin receptor 5 | P33032 | SINGLE PROTEIN | Homo sapiens | 2373 | 4352 | 9606 | FALSE |
| CHEMBL222 | Norepinephrine transporter | P23975 | SINGLE PROTEIN | Homo sapiens | 6861 | 10394 | 9606 | FALSE |
| CHEMBL2095182 | Tubulin | P68371|Q9H4B7|P0DPH7|P68366|Q13509|Q71U36|P04350|Q3ZCM7|P07437|P68363|Q9BQE3|Q6PEY2|Q13885|Q9BVA1|Q9BUF5 | PROTEIN COMPLEX GROUP | Homo sapiens | 2654 | 5762 | 9606 | FALSE |
| CHEMBL3797016 | C-terminal-binding protein 2 | P56545 | SINGLE PROTEIN | Homo sapiens | 25 | 36 | 9606 | FALSE |
| CHEMBL3883307 | Axin-1/DVL-2 | O14641|O15169 | PROTEIN-PROTEIN INTERACTION | Homo sapiens | 1 | 1 | 9606 | FALSE |
| CHEMBL2364701 | 26S proteasome | Q99460|P49721|P20618|P28074|P28062|P28065|P28072|O00487|P25786|P25787|P25788|P25789|P28066|P60900|O14818|Q8TAA3|P40306|A5LHX3|P49720|P28070|Q99436|Q16186|P35998|P62191|P43686|P62333|P17980|P62195|Q13200|O43242|O00232|O00231|Q15008|P51665|Q9UNM6|P55036|P48556|P60896 | PROTEIN COMPLEX | Homo sapiens | 67 | 146 | 9606 | FALSE |
| CHEMBL4523654 | Antigen peptide transporter 1/2 | Q03518|Q03519 | PROTEIN COMPLEX | Homo sapiens | 5 | 5 | 9606 | FALSE |
| CHEMBL3038458 | Nicotinic acetylcholine receptor alpha2/beta2 | P02708|P17787 | PROTEIN COMPLEX | Homo sapiens | 15 | 15 | 9606 | FALSE |
| CHEMBL5963 | Proteinase-activated receptor 2 | P55085 | SINGLE PROTEIN | Homo sapiens | 341 | 707 | 9606 | FALSE |
| CHEMBL5918 | Canalicular multispecific organic anion transporter 2 | O15438 | SINGLE PROTEIN | Homo sapiens | 660 | 718 | 9606 | FALSE |
| CHEMBL2364163 | Complement C5 | P01031 | SINGLE PROTEIN | Homo sapiens | 11 | 49 | 9606 | FALSE |
| CHEMBL4792 | Orexin receptor 2 | O43614 | SINGLE PROTEIN | Homo sapiens | 4496 | 6005 | 9606 | FALSE |
| CHEMBL3885571 | Gamma-aminobutyric acid receptor subunit alpha-2/beta-2 | P47870|P47869 | PROTEIN COMPLEX | Homo sapiens | 3 | 6 | 9606 | FALSE |
| CHEMBL3831201 | 20S proteasome | P49721|P20618|P28074|P28062|P28065|P28072|P25786|P25787|P25788|P25789|P28066|P60900|O14818|Q8TAA3|P40306|A5LHX3|P49720|P28070|Q99436 | PROTEIN COMPLEX GROUP | Homo sapiens | 392 | 646 | 9606 | FALSE |
| CHEMBL3559682 | Serine/threonine-protein kinase PIM | P11309|Q9P1W9|Q86V86 | PROTEIN FAMILY | Homo sapiens | 103 | 117 | 9606 | FALSE |
| CHEMBL3880 | Heat shock protein HSP 90-alpha | P07900 | SINGLE PROTEIN | Homo sapiens | 3019 | 4197 | 9606 | FALSE |
| CHEMBL5790 | Serine/threonine-protein kinase PCTAIRE-2 | Q00537 | SINGLE PROTEIN | Homo sapiens | 429 | 548 | 9606 | FALSE |
| CHEMBL614339 | Me665/2/21 |  | CELL-LINE | Homo sapiens | 2 | 2 | 9606 | FALSE |
| CHEMBL2940 | Immunoglobulin epsilon Fc receptor | P06734 | SINGLE PROTEIN | Homo sapiens | 88 | 92 | 9606 | FALSE |
| CHEMBL5209631 | Sodium/hydrogen exchanger 9B2 | Q86UD5 | SINGLE PROTEIN | Homo sapiens | 1 | 1 | 9606 | FALSE |
| CHEMBL3832646 | fructose-2,6-bisphosphatase 3/4 | Q16875|Q16877 | PROTEIN COMPLEX | Homo sapiens | 28 | 29 | 9606 | FALSE |
| CHEMBL1743122 | Solute carrier family 22 member 2 | O15244 | SINGLE PROTEIN | Homo sapiens | 147 | 261 | 9606 | FALSE |
| CHEMBL2111389 | CDK9/cyclin T1 | O60563|P50750 | PROTEIN COMPLEX | Homo sapiens | 2147 | 2783 | 9606 | FALSE |
| CHEMBL4462 | NAD-dependent deacetylase sirtuin 2 | Q8IXJ6 | SINGLE PROTEIN | Homo sapiens | 2468 | 4045 | 9606 | FALSE |
| CHEMBL4106134 | FASN/HER2 | P04626|P49327 | PROTEIN COMPLEX | Homo sapiens | 1 | 2 | 9606 | FALSE |
| CHEMBL5736 | Glucagon | P01275 | SINGLE PROTEIN | Homo sapiens | 122 | 136 | 9606 | FALSE |
| CHEMBL1914276 | Inward rectifier potassium channel 2 | P63252 | SINGLE PROTEIN | Homo sapiens | 32 | 37 | 9606 | FALSE |
| CHEMBL1075094 | Nuclear factor erythroid 2-related factor 2 | Q16236 | SINGLE PROTEIN | Homo sapiens | 86443 | 95554 | 9606 | FALSE |
| CHEMBL4917 | Complement C3 | P01024 | SINGLE PROTEIN | Homo sapiens | 70 | 152 | 9606 | FALSE |
| CHEMBL2363070 | Retinoid X receptor | P28702|P48443|P19793 | PROTEIN FAMILY | Homo sapiens | 51 | 115 | 9606 | FALSE |
| CHEMBL2111348 | Soluble guanylate cyclase | Q02153|O75343|P33402|Q02108 | PROTEIN COMPLEX GROUP | Homo sapiens | 620 | 680 | 9606 | FALSE |
| CHEMBL4163 | Toll-like receptor 2 | O60603 | SINGLE PROTEIN | Homo sapiens | 715 | 1015 | 9606 | FALSE |
| CHEMBL2111369 | Inosine-5'-monophosphate dehydrogenase (IMPDH) | P20839|P12268 | PROTEIN FAMILY | Homo sapiens | 52 | 61 | 9606 | FALSE |
| CHEMBL5697 | Egl nine homolog 1 | Q9GZT9 | SINGLE PROTEIN | Homo sapiens | 1277 | 1744 | 9606 | FALSE |
| CHEMBL3119 | Transient receptor potential cation channel subfamily V member 4 | Q9HBA0 | SINGLE PROTEIN | Homo sapiens | 646 | 825 | 9606 | FALSE |
| CHEMBL284 | Dipeptidyl peptidase IV | P27487 | SINGLE PROTEIN | Homo sapiens | 5497 | 7291 | 9606 | FALSE |
| CHEMBL2364173 | Amylin receptor AMY2; CALCR/RAMP2 | P30988|O60895 | PROTEIN COMPLEX | Homo sapiens | 1 | 1 | 9606 | FALSE |
| CHEMBL3559683 | Peroxisome proliferator-activated receptor | P37231|Q07869|Q03181 | PROTEIN FAMILY | Homo sapiens | 85 | 137 | 9606 | FALSE |
| CHEMBL2040 | T-cell surface antigen CD2 | P06729 | SINGLE PROTEIN | Homo sapiens | 6 | 9 | 9606 | FALSE |
| CHEMBL1293266 | Nucleotide-binding oligomerization domain-containing protein 2 | Q9HC29 | SINGLE PROTEIN | Homo sapiens | 1032 | 1620 | 9606 | FALSE |
| CHEMBL2964 | Dual specificity mitogen-activated protein kinase kinase 2 | P36507 | SINGLE PROTEIN | Homo sapiens | 1041 | 1332 | 9606 | FALSE |
| CHEMBL3670 | 3-beta-hydroxysteroid dehydrogenase/delta 5-->4-isomerase type II | P26439 | SINGLE PROTEIN | Homo sapiens | 4 | 4 | 9606 | FALSE |
| CHEMBL4630879 | Hepatitis A virus cellular receptor 2 | Q8TDQ0 | SINGLE PROTEIN | Homo sapiens | 64 | 74 | 9606 | FALSE |
| CHEMBL2093866 | Estrogen receptor | Q92731|P03372 | PROTEIN FAMILY | Homo sapiens | 1789 | 3070 | 9606 | FALSE |
| CHEMBL260 | MAP kinase p38 alpha | Q16539 | SINGLE PROTEIN | Homo sapiens | 8452 | 13014 | 9606 | FALSE |
| CHEMBL2331041 | Potassium channel subfamily K member 10 | P57789 | SINGLE PROTEIN | Homo sapiens | 45 | 76 | 9606 | FALSE |
| CHEMBL3883293 | MAP kinase-interacting serine/threonine-protein kinase 1/2 | Q9HBH9|Q9BUB5 | PROTEIN FAMILY | Homo sapiens | 129 | 155 | 9606 | FALSE |
| CHEMBL1907611 | Tumour suppressor p53/oncoprotein Mdm2 | P04637|Q00987 | PROTEIN-PROTEIN INTERACTION | Homo sapiens | 1601 | 2107 | 9606 | FALSE |
| CHEMBL4822 | Beta-secretase 1 | P56817 | SINGLE PROTEIN | Homo sapiens | 10006 | 15784 | 9606 | FALSE |
| CHEMBL4093 | LXR-beta | P55055 | SINGLE PROTEIN | Homo sapiens | 2134 | 3883 | 9606 | FALSE |
| CHEMBL2111337 | Atrial natriuretic peptide receptor | P20594|P16066|P17342 | PROTEIN FAMILY | Homo sapiens | 38 | 49 | 9606 | FALSE |
| CHEMBL2096668 | Alcohol dehydrogenase | P07327|P08319|P00325|P00326|P40394|P11766|P28332 | PROTEIN FAMILY | Homo sapiens | 24 | 35 | 9606 | FALSE |
| CHEMBL5398 | Hepatocyte nuclear factor 4-alpha | P41235 | SINGLE PROTEIN | Homo sapiens | 283 | 305 | 9606 | FALSE |
| CHEMBL3785 | Hydroxycarboxylic acid receptor 2 | Q8TDS4 | SINGLE PROTEIN | Homo sapiens | 1013 | 1909 | 9606 | FALSE |
| CHEMBL4040 | MAP kinase ERK2 | P28482 | SINGLE PROTEIN | Homo sapiens | 21475 | 25131 | 9606 | FALSE |
| CHEMBL2095186 | Sodium/potassium-transporting ATPase | P05023|P14415|P05026|P50993|P54709|P13637|Q13733|P54710 | PROTEIN COMPLEX GROUP | Homo sapiens | 359 | 388 | 9606 | FALSE |
| CHEMBL2363069 | Retinoic acid receptor | P13631|P10826|P10276 | PROTEIN FAMILY | Homo sapiens | 29 | 39 | 9606 | FALSE |
| CHEMBL2409 | Epoxide hydratase | P34913 | SINGLE PROTEIN | Homo sapiens | 2801 | 3944 | 9606 | FALSE |
| CHEMBL2095180 | Carbonic anhydrase | P00918|P43166|P00915|P07451|P23280|O43570|Q9ULX7|Q16790|P22748|Q8N1Q1|Q9Y2D0|P35218 | PROTEIN FAMILY | Homo sapiens | 124 | 131 | 9606 | FALSE |
| CHEMBL1795122 | Elongation factor 1-alpha 2 | Q05639 | SINGLE PROTEIN | Homo sapiens | 3 | 9 | 9606 | FALSE |
| CHEMBL3816 | Cytosolic phospholipase A2 | P47712 | SINGLE PROTEIN | Homo sapiens | 588 | 787 | 9606 | FALSE |
| CHEMBL3559691 | Cyclin-dependent kinase | P06493|P11802|P24941|Q00535|Q00534|P50613|Q00526|Q00536|Q07002|P49336|Q00537|P21127|Q96Q40|Q9BWU1|O94921|P50750|Q15131|Q14004|Q8IZL9|Q9NYV4 | PROTEIN FAMILY | Homo sapiens | 38 | 77 | 9606 | FALSE |
| CHEMBL2094135 | Gamma-secretase | Q96BI3|Q9NZ42|Q8WW43|P49768|Q92542|P49810 | PROTEIN COMPLEX | Homo sapiens | 2862 | 4921 | 9606 | FALSE |
| CHEMBL4323 | Phospholipase A2 group V | P39877 | SINGLE PROTEIN | Homo sapiens | 222 | 238 | 9606 | FALSE |
| CHEMBL2176855 | C-type lectin domain family 4 member C | Q8WTT0 | SINGLE PROTEIN | Homo sapiens | 6 | 7 | 9606 | FALSE |
| CHEMBL3713006 | Programmed cell death 1 ligand 2 | Q9BQ51 | SINGLE PROTEIN | Homo sapiens | 3 | 3 | 9606 | FALSE |
| CHEMBL3385 | MAP kinase ERK1 | P27361 | SINGLE PROTEIN | Homo sapiens | 2944 | 4793 | 9606 | FALSE |
| CHEMBL4998 | Integrin alpha-2 | P17301 | SINGLE PROTEIN | Homo sapiens | 10 | 11 | 9606 | FALSE |
| CHEMBL4398 | Purinergic receptor P2Y2 | P41231 | SINGLE PROTEIN | Homo sapiens | 635 | 1110 | 9606 | FALSE |
| CHEMBL1887 | Interferon-alpha/beta receptor alpha chain | P17181 | SINGLE PROTEIN | Homo sapiens | 38 | 50 | 9606 | FALSE |
| CHEMBL204 | Thrombin | P00734 | SINGLE PROTEIN | Homo sapiens | 10010 | 11834 | 9606 | FALSE |
| CHEMBL2111362 | Integrin alpha-M/beta-2 | P05107|P11215 | PROTEIN COMPLEX | Homo sapiens | 39 | 50 | 9606 | FALSE |
| CHEMBL2096907 | AMP-activated protein kinase, AMPK | P54646|O43741|P54619|Q9UGJ0|Q9Y478|Q13131|Q9UGI9 | PROTEIN COMPLEX GROUP | Homo sapiens | 11688 | 12274 | 9606 | FALSE |
| CHEMBL242 | Estrogen receptor beta | Q92731 | SINGLE PROTEIN | Homo sapiens | 5110 | 9511 | 9606 | FALSE |
| CHEMBL340 | Cytochrome P450 3A4 | P08684 | SINGLE PROTEIN | Homo sapiens | 34417 | 54600 | 9606 | FALSE |
| CHEMBL2093865 | Histone deacetylase | Q13547|O15379|Q9UBN7|Q92769|Q9UQL6|Q8WUI4|Q9BY41|Q96DB2|P56524|Q9UKV0|Q969S8 | PROTEIN FAMILY | Homo sapiens | 4183 | 7025 | 9606 | FALSE |
| CHEMBL4016 | Glutamate receptor ionotropic, AMPA 2 | P42262 | SINGLE PROTEIN | Homo sapiens | 455 | 865 | 9606 | FALSE |
| CHEMBL2973 | Rho-associated protein kinase 2 | O75116 | SINGLE PROTEIN | Homo sapiens | 4813 | 6441 | 9606 | FALSE |
| CHEMBL2821 | Coagulation factor XII | P00748 | SINGLE PROTEIN | Homo sapiens | 1283 | 1462 | 9606 | FALSE |
| CHEMBL3831181 | Growth/differentiation factor 2 | Q9UK05 | SINGLE PROTEIN | Homo sapiens | 2 | 2 | 9606 | FALSE |
| CHEMBL2095203 | Adrenergic receptor alpha | P35348|P08913|P18825|P25100|P35368|P18089 | PROTEIN FAMILY | Homo sapiens | 103 | 139 | 9606 | FALSE |
| CHEMBL4660 | Lymphocyte differentiation antigen CD38 | P28907 | SINGLE PROTEIN | Homo sapiens | 358 | 414 | 9606 | FALSE |
| CHEMBL2095158 | Adrenergic receptor alpha-2 | P08913|P18825|P18089 | PROTEIN FAMILY | Homo sapiens | 491 | 812 | 9606 | FALSE |
| CHEMBL1947 | Thyroid hormone receptor beta-1 | P10828 | SINGLE PROTEIN | Homo sapiens | 6356 | 7937 | 9606 | FALSE |
| CHEMBL3390820 | PARP 1, 2 and 3 | P09874|Q9Y6F1|Q9UGN5 | PROTEIN FAMILY | Homo sapiens | 96 | 151 | 9606 | FALSE |
| CHEMBL5071 | G protein-coupled receptor 44 | Q9Y5Y4 | SINGLE PROTEIN | Homo sapiens | 2943 | 4688 | 9606 | FALSE |
| CHEMBL5038 | Melanin-concentrating hormone receptor 2 | Q969V1 | SINGLE PROTEIN | Homo sapiens | 295 | 304 | 9606 | FALSE |
| CHEMBL5668 | Serine/threonine-protein kinase SRPK2 | P78362 | SINGLE PROTEIN | Homo sapiens | 529 | 817 | 9606 | FALSE |
| CHEMBL4267 | TGF-beta receptor type II | P37173 | SINGLE PROTEIN | Homo sapiens | 671 | 850 | 9606 | FALSE |
| CHEMBL3220 | PI4-kinase type II beta | Q8TCG2 | SINGLE PROTEIN | Homo sapiens | 7 | 12 | 9606 | FALSE |
| CHEMBL3988631 | Heat shock factor protein | Q00613|Q03933|Q9ULV5 | PROTEIN FAMILY | Homo sapiens | 28 | 28 | 9606 | FALSE |
| CHEMBL2366281 | EM-2 |  | CELL-LINE | Homo sapiens | 129 | 130 | 9606 | FALSE |
| CHEMBL4524006 | RAS | P01112|P01111|P01116 | PROTEIN FAMILY | Homo sapiens | 2 | 4 | 9606 | FALSE |
| CHEMBL2068 | Ephrin type-A receptor 2 | P29317 | SINGLE PROTEIN | Homo sapiens | 2632 | 3552 | 9606 | FALSE |
| CHEMBL3038512 | Toll-like receptor 4/MD-2 | O00206|Q9Y6Y9 | PROTEIN COMPLEX | Homo sapiens | 19 | 56 | 9606 | FALSE |
| CHEMBL3238 | Carnitine palmitoyltransferase 2 | P23786 | SINGLE PROTEIN | Homo sapiens | 496 | 510 | 9606 | FALSE |
| CHEMBL3712886 | 5,6-dihydroxyindole-2-carboxylic acid oxidase | P17643 | SINGLE PROTEIN | Homo sapiens | 5 | 8 | 9606 | FALSE |
| CHEMBL2363075 | Steroid 5-alpha-reductase | P18405|P31213|Q9H8P0 | PROTEIN FAMILY | Homo sapiens | 1 | 1 | 9606 | FALSE |
| CHEMBL2331043 | Sodium channel alpha subunit | P35498|Q14524|P35499|Q01118|Q99250|Q15858|Q9NY46|Q9UI33|Q9UQD0|Q9Y5Y9 | PROTEIN FAMILY | Homo sapiens | 25 | 30 | 9606 | FALSE |
| CHEMBL2096905 | Dopamine receptor | P14416|P35462|P21918|P21917|P21728 | PROTEIN FAMILY | Homo sapiens | 99 | 117 | 9606 | FALSE |
| CHEMBL2364172 | Integrin alpha-L/beta-2 (LFA-1) | P20701|P05107 | PROTEIN COMPLEX | Homo sapiens | 33 | 51 | 9606 | FALSE |
| CHEMBL3559686 | Frizzled-2 | Q14332 | SINGLE PROTEIN | Homo sapiens | 25 | 110 | 9606 | FALSE |
| CHEMBL2327 | Neurokinin 2 receptor | P21452 | SINGLE PROTEIN | Homo sapiens | 2193 | 3352 | 9606 | FALSE |
| CHEMBL1856 | Steroid 5-alpha-reductase 2 | P31213 | SINGLE PROTEIN | Homo sapiens | 757 | 937 | 9606 | FALSE |
| CHEMBL2111330 | Ribosomal protein S6 kinase (P70S6K) | Q9UBS0|P23443 | PROTEIN COMPLEX | Homo sapiens | 88 | 90 | 9606 | FALSE |
| CHEMBL1965 | Melanocortin receptor 2 | Q01718 | SINGLE PROTEIN | Homo sapiens | 64 | 112 | 9606 | FALSE |
| CHEMBL3896 | Voltage-gated calcium channel alpha2/delta subunit 2 | Q9NY47 | SINGLE PROTEIN | Homo sapiens | 36 | 37 | 9606 | FALSE |
| CHEMBL3961 | MAP kinase p38 beta | Q15759 | SINGLE PROTEIN | Homo sapiens | 1793 | 2841 | 9606 | FALSE |
| CHEMBL2331074 | Adrenergic receptor | P35348|P08913|P08588|P07550|P18825|P25100|P35368|P18089|P13945 | PROTEIN FAMILY | Homo sapiens | 4 | 7 | 9606 | FALSE |
| CHEMBL224 | Serotonin 2a (5-HT2a) receptor | P28223 | SINGLE PROTEIN | Homo sapiens | 9084 | 15395 | 9606 | FALSE |
| CHEMBL3831289 | Caspase | P49662|P55211|P42574|P51878|P55212|P55210|Q14790|P29466|P42575|Q92851|P31944|Q6UXS9 | PROTEIN FAMILY | Homo sapiens | 2 | 2 | 9606 | FALSE |
| CHEMBL2109241 | Glutamate receptor ionotropic kainate | P39086|Q16478|Q16099|Q13002|Q13003 | PROTEIN COMPLEX GROUP | Homo sapiens | 61 | 68 | 9606 | FALSE |
| CHEMBL3833502 | Alpha glucosidase | O43451|P10253|P14410|Q2M2H8 | PROTEIN FAMILY | Homo sapiens | 701 | 873 | 9606 | FALSE |
| CHEMBL2094255 | DNA topoisomerase II | P11388|Q02880 | PROTEIN FAMILY | Homo sapiens | 854 | 1344 | 9606 | FALSE |
| CHEMBL2111459 | Rho-associated protein kinase | O75116|Q13464 | PROTEIN FAMILY | Homo sapiens | 126 | 137 | 9606 | FALSE |
| CHEMBL1075153 | Tumor necrosis factor receptor superfamily member 10B | O14763 | SINGLE PROTEIN | Homo sapiens | 13 | 15 | 9606 | FALSE |
| CHEMBL205 | Carbonic anhydrase II | P00918 | SINGLE PROTEIN | Homo sapiens | 10971 | 18047 | 9606 | FALSE |
| CHEMBL3108633 | Platelet-derived growth factor subunit B | P01127 | SINGLE PROTEIN | Homo sapiens | 2 | 2 | 9606 | FALSE |
| CHEMBL5366 | Poly [ADP-ribose] polymerase 2 | Q9UGN5 | SINGLE PROTEIN | Homo sapiens | 983 | 1287 | 9606 | FALSE |
| CHEMBL3890 | Selectin E | P16581 | SINGLE PROTEIN | Homo sapiens | 501 | 659 | 9606 | FALSE |
| CHEMBL333 | Matrix metalloproteinase-2 | P08253 | SINGLE PROTEIN | Homo sapiens | 5651 | 6713 | 9606 | FALSE |
| CHEMBL2095229 | Voltage-gated L-type calcium channel | Q13936|Q13698|Q01668|O60840 | PROTEIN FAMILY | Homo sapiens | 478 | 710 | 9606 | FALSE |
| CHEMBL2321615 | Potassium channel subfamily K member 2 | O95069 | SINGLE PROTEIN | Homo sapiens | 201 | 490 | 9606 | FALSE |
| CHEMBL3028 | Hypoxia-inducible factor prolyl hydroxylase 1 | Q96KS0 | SINGLE PROTEIN | Homo sapiens | 394 | 404 | 9606 | FALSE |

**Table S3. DEHP targets from CTD database.**

| **Gene Symbol** | **Gene ID** | **Interaction Count** | **Organism Count** |
| --- | --- | --- | --- |
| PPARA | 5465 | 553 | 4 |
| PPARG | 5468 | 89 | 6 |
| CASP3 | 836 | 84 | 6 |
| BAX | 581 | 79 | 7 |
| ESR1 | 2099 | 78 | 7 |
| BCL2 | 596 | 76 | 7 |
| AKT1 | 207 | 72 | 4 |
| INS1 | 16333 | 63 | 3 |
| STAR | 6770 | 61 | 3 |
| CAT | 847 | 56 | 10 |
| HMOX1 | 3162 | 55 | 6 |
| NFE2L2 | 4780 | 55 | 6 |
| CYP11A1 | 1583 | 50 | 3 |
| TNF | 7124 | 45 | 4 |
| CYP19A1 | 1588 | 43 | 3 |
| PTGS2 | 5743 | 42 | 3 |
| HSD3B1 | 3283 | 41 | 3 |
| IL1B | 3553 | 38 | 4 |
| AHR | 196 | 37 | 4 |
| NR1I2 | 8856 | 37 | 5 |
| SOD1 | 6647 | 37 | 7 |
| CGA | 1081 | 36 | 3 |
| SOD2 | 6648 | 36 | 6 |
| CYP17A1 | 1586 | 35 | 3 |
| MAPK1 | 5594 | 33 | 4 |
| MAPK3 | 5595 | 33 | 4 |
| GPX4 | 2879 | 32 | 2 |
| IL6 | 3569 | 31 | 5 |
| GPX1 | 2876 | 30 | 4 |
| MMP9 | 4318 | 30 | 3 |
| CYP1A1 | 1543 | 29 | 4 |
| CYP3A4 | 1576 | 29 | 4 |
| NLRP3 | 114548 | 29 | 4 |
| AR | 367 | 28 | 5 |
| INS | 3630 | 28 | 4 |
| MTOR | 2475 | 28 | 3 |
| MMP2 | 4313 | 27 | 3 |
| NRG1 | 3084 | 26 | 2 |
| ESR2 | 2100 | 25 | 3 |
| IFNG | 3458 | 25 | 1 |
| IL33 | 90865 | 25 | 2 |
| PIK3R1 | 5295 | 25 | 4 |
| PINK1 | 65018 | 25 | 4 |
| VIM | 7431 | 25 | 3 |
| NR1I3 | 9970 | 24 | 4 |
| SLC2A4 | 6517 | 24 | 3 |
| TP53 | 7157 | 24 | 4 |
| IGF1 | 3479 | 23 | 3 |
| IRS1 | 3667 | 23 | 3 |
| NQO1 | 1728 | 23 | 5 |
| SCARB1 | 949 | 23 | 3 |
| CDKN1A | 1026 | 22 | 3 |
| CYP1B1 | 1545 | 22 | 4 |
| GJA1 | 2697 | 22 | 4 |
| INSL3 | 3640 | 22 | 2 |
| OVAL | 396058 | 22 | 1 |
| PCNA | 5111 | 22 | 4 |
| PRKN | 5071 | 22 | 3 |
| TJP1 | 7082 | 22 | 3 |
| FABP4 | 2167 | 21 | 4 |
| MYC | 4609 | 21 | 3 |
| CASP8 | 841 | 20 | 3 |
| CTNNB1 | 1499 | 20 | 3 |
| INSR | 3643 | 20 | 4 |
| PTEN | 5728 | 20 | 4 |
| RELA | 5970 | 20 | 3 |
| ACOX1 | 51 | 19 | 2 |
| FSHB | 2488 | 19 | 4 |
| IL4 | 3565 | 19 | 2 |
| MAP1LC3B | 81631 | 19 | 4 |
| SIRT1 | 23411 | 19 | 6 |
| SLC7A11 | 23657 | 19 | 2 |
| TGFB1 | 7040 | 19 | 3 |
| CASP1 | 834 | 18 | 3 |
| CDH2 | 1000 | 18 | 3 |
| FAS | 355 | 18 | 3 |
| GPT | 2875 | 18 | 5 |
| TIMP2 | 7077 | 18 | 3 |
| CASP9 | 842 | 17 | 6 |
| CD36 | 948 | 17 | 2 |
| FASN | 2194 | 17 | 3 |
| LEP | 3952 | 17 | 3 |
| OCLN | 100506658 | 17 | 2 |
| PPARGC1A | 10891 | 17 | 5 |
| SREBF1 | 6720 | 17 | 3 |
| ACSL4 | 2182 | 16 | 1 |
| CPT1A | 1374 | 16 | 2 |
| FGF21 | 26291 | 16 | 1 |
| HDAC6 | 10013 | 16 | 3 |
| PDK4 | 5166 | 16 | 2 |
| RAP1A | 5906 | 16 | 2 |
| SQSTM1 | 8878 | 16 | 4 |
| CYP4A14 | 13119 | 15 | 1 |
| GSK3B | 2932 | 15 | 3 |
| LHB | 3972 | 15 | 2 |
| TSHR | 7253 | 15 | 2 |
| ADIPOQ | 9370 | 14 | 3 |
| AMH | 268 | 14 | 3 |
| CREB1 | 1385 | 14 | 2 |
| CSNK2B | 1460 | 14 | 1 |
| H2AX | 3014 | 14 | 3 |
| HIF1A | 3091 | 14 | 1 |
| MFN2 | 9927 | 14 | 3 |
| MKI67 | 4288 | 14 | 3 |
| NOTCH1 | 4851 | 14 | 4 |
| PARP1 | 142 | 14 | 3 |
| PAX8 | 7849 | 14 | 3 |
| SLC39A5 | 283375 | 14 | 3 |
| TFEB | 7942 | 14 | 2 |
| APOE | 348 | 13 | 3 |
| CEBPB | 1051 | 13 | 3 |
| CSF2 | 1437 | 13 | 1 |
| CYCS | 54205 | 13 | 3 |
| FTH1 | 2495 | 13 | 1 |
| IL10 | 3586 | 13 | 2 |
| LHCGR | 3973 | 13 | 3 |
| SLC2A2 | 6514 | 13 | 2 |
| CCND1 | 595 | 12 | 3 |
| EIF2AK3 | 9451 | 12 | 3 |
| EPHX1 | 2052 | 12 | 2 |
| FOXO1 | 2308 | 12 | 3 |
| IL12B | 3593 | 12 | 1 |
| MIR93 | 407050 | 12 | 2 |
| NR3C1 | 2908 | 12 | 3 |
| NR5A1 | 2516 | 12 | 2 |
| PDE3A | 5139 | 12 | 1 |
| SIRT3 | 23410 | 12 | 3 |
| SP3 | 6670 | 12 | 2 |
| ACADM | 34 | 11 | 2 |
| ANGPTL4 | 51129 | 11 | 2 |
| CCND2 | 894 | 11 | 1 |
| CDH1 | 999 | 11 | 3 |
| FOS | 2353 | 11 | 4 |
| FOXO3 | 2309 | 11 | 2 |
| FSHR | 2492 | 11 | 3 |
| GATA4 | 2626 | 11 | 1 |
| GCLM | 2730 | 11 | 3 |
| GSDMD | 79792 | 11 | 3 |
| HMGCR | 3156 | 11 | 3 |
| HSD17B3 | 3293 | 11 | 2 |
| HSPA5 | 3309 | 11 | 3 |
| HSPD1 | 3329 | 11 | 4 |
| METTL3 | 56339 | 11 | 1 |
| MFN1 | 55669 | 11 | 3 |
| PIK3CG | 5294 | 11 | 3 |
| RBP4 | 5950 | 11 | 3 |
| SLC27A1 | 376497 | 11 | 2 |
| SOD3 | 6649 | 11 | 4 |
| THRB | 7068 | 11 | 3 |
| VEGFA | 7422 | 11 | 3 |
| WNT4 | 54361 | 11 | 2 |
| XBP1 | 7494 | 11 | 2 |
| CDK4 | 1019 | 10 | 2 |
| CYP51 | 13121 | 10 | 2 |
| DNMT1 | 1786 | 10 | 3 |
| DNMT3A | 1788 | 10 | 3 |
| EGR1 | 1958 | 10 | 4 |
| FABP3 | 2170 | 10 | 2 |
| HMGCS1 | 3157 | 10 | 3 |
| HSD17B1 | 3292 | 10 | 4 |
| HSP90AA1 | 3320 | 10 | 3 |
| LPCAT3 | 10162 | 10 | 1 |
| PARK | 40336 | 10 | 2 |
| SOHLH2 | 54937 | 10 | 2 |
| STAT5A | 6776 | 10 | 1 |
| STAT5B | 6777 | 10 | 3 |
| TH | 7054 | 10 | 3 |
| THRA | 7067 | 10 | 3 |
| TIMP1 | 7076 | 10 | 3 |
| TXNIP | 10628 | 10 | 3 |
| VDAC1 | 7416 | 10 | 4 |
| ACTA2 | 59 | 9 | 3 |
| ALDH1A1 | 216 | 9 | 3 |
| BCL2L1 | 598 | 9 | 2 |
| BDNF | 627 | 9 | 2 |
| CCL2 | 6347 | 9 | 2 |
| CCNE1 | 898 | 9 | 3 |
| CDKN1C | 1028 | 9 | 2 |
| CYP2C9 | 1559 | 9 | 2 |
| CYP4A10 | 13117 | 9 | 2 |
| CYP7A1 | 1581 | 9 | 2 |
| DIO3 | 1735 | 9 | 3 |
| DLL4 | 54567 | 9 | 3 |
| DNM1L | 10059 | 9 | 2 |
| DNMT3B | 1789 | 9 | 4 |
| EPHX2 | 2053 | 9 | 2 |
| GCLC | 2729 | 9 | 3 |
| GDNF | 2668 | 9 | 3 |
| GNRH1 | 2796 | 9 | 2 |
| IFNL2 | 282616 | 9 | 2 |
| JUNB | 3726 | 9 | 2 |
| KEAP1 | 9817 | 9 | 3 |
| KISS1 | 3814 | 9 | 2 |
| KLF7 | 8609 | 9 | 2 |
| LIPE | 3991 | 9 | 1 |
| LPL | 4023 | 9 | 3 |
| MT2 | 17750 | 9 | 1 |
| NOTCH2 | 4853 | 9 | 2 |
| NR0B1 | 190 | 9 | 2 |
| PCK1 | 5105 | 9 | 3 |
| PLIN2 | 123 | 9 | 2 |
| POU5F1 | 5460 | 9 | 4 |
| PPARD | 5467 | 9 | 3 |
| PYCARD | 29108 | 9 | 2 |
| SCD1 | 20249 | 9 | 1 |
| SLC2A1 | 6513 | 9 | 2 |
| SOX9 | 6662 | 9 | 2 |
| STRA8 | 346673 | 9 | 3 |
| THBS1 | 7057 | 9 | 4 |
| TOMM20 | 9804 | 9 | 4 |
| TSHB | 7252 | 9 | 3 |
| ARNT | 405 | 8 | 1 |
| ATF3 | 467 | 8 | 2 |
| BECN1 | 8678 | 8 | 2 |
| CCNB1 | 891 | 8 | 2 |
| CEBPA | 1050 | 8 | 3 |
| CTSD | 1509 | 8 | 2 |
| CYP1A2 | 1544 | 8 | 2 |
| DDIT3 | 1649 | 8 | 3 |
| ERBB2 | 2064 | 8 | 2 |
| ERBB4 | 2066 | 8 | 2 |
| ERN1 | 2081 | 8 | 3 |
| FOSB | 2354 | 8 | 2 |
| GADD45A | 1647 | 8 | 1 |
| GOT1 | 2805 | 8 | 4 |
| GPER1 | 2852 | 8 | 3 |
| GSTM1 | 2944 | 8 | 1 |
| HMGCS2 | 3158 | 8 | 2 |
| IGF1R | 3480 | 8 | 2 |
| LDLR | 3949 | 8 | 3 |
| NOS2 | 4843 | 8 | 3 |
| NOTCH4 | 4855 | 8 | 3 |
| NR4A1 | 3164 | 8 | 3 |
| NRF1 | 4899 | 8 | 4 |
| PGR | 5241 | 8 | 3 |
| PLTP | 5360 | 8 | 2 |
| PRDX5 | 25824 | 8 | 1 |
| PRKACA | 5566 | 8 | 2 |
| TFAM | 7019 | 8 | 4 |
| TFRC | 7037 | 8 | 3 |
| TSPO | 706 | 8 | 2 |
| ABCA1 | 19 | 7 | 2 |
| ACAA1A | 113868 | 7 | 1 |
| ACACA | 31 | 7 | 2 |
| ACLY | 47 | 7 | 1 |
| ACSL1 | 2180 | 7 | 3 |
| ALPL | 249 | 7 | 5 |
| BCL6 | 604 | 7 | 2 |
| BHMT | 635 | 7 | 2 |
| CASP7 | 840 | 7 | 3 |
| CCNA1 | 8900 | 7 | 3 |
| CCNA2 | 890 | 7 | 3 |
| CYP2B10 | 13088 | 7 | 1 |
| CYP2C6 | 293989 | 7 | 1 |
| CYP3A11 | 13112 | 7 | 1 |
| CYP3A2 | 266682 | 7 | 1 |
| DIABLO | 56616 | 7 | 2 |
| DIO2 | 1734 | 7 | 3 |
| EBP | 10682 | 7 | 2 |
| EGFR | 1956 | 7 | 3 |
| FABP5 | 2171 | 7 | 3 |
| FDPS | 2224 | 7 | 2 |
| GADD45G | 10912 | 7 | 3 |
| GSR | 2936 | 7 | 3 |
| GSTA3 | 2940 | 7 | 4 |
| HAS2 | 3037 | 7 | 3 |
| HEY2 | 23493 | 7 | 1 |
| HSD3B2 | 3284 | 7 | 3 |
| INSIG2 | 51141 | 7 | 2 |
| JAG1 | 182 | 7 | 3 |
| JAG2 | 3714 | 7 | 2 |
| JAK3 | 3718 | 7 | 1 |
| JUN | 3725 | 7 | 2 |
| KIT | 3815 | 7 | 2 |
| LAMC2 | 3918 | 7 | 2 |
| LDHA | 3939 | 7 | 3 |
| MAPK8 | 5599 | 7 | 3 |
| MGLL | 11343 | 7 | 2 |
| MSMO1 | 6307 | 7 | 3 |
| MT1 | 17748 | 7 | 2 |
| MVD | 4597 | 7 | 2 |
| NOTCH3 | 4854 | 7 | 2 |
| NR1H3 | 10062 | 7 | 3 |
| NSDHL | 50814 | 7 | 2 |
| NSG1 | 27065 | 7 | 3 |
| PTGER2 | 5732 | 7 | 3 |
| PTGER4 | 5734 | 7 | 2 |
| PTGES | 9536 | 7 | 3 |
| RUNX2 | 860 | 7 | 4 |
| SFXN3 | 81855 | 7 | 3 |
| SLAMF1 | 6504 | 7 | 1 |
| SLC25A20 | 788 | 7 | 2 |
| SPRY4 | 81848 | 7 | 3 |
| SULT1E1 | 6783 | 7 | 1 |
| TG | 7038 | 7 | 2 |
| TLR4 | 7099 | 7 | 3 |
| TUBB3 | 10381 | 7 | 3 |
| UCP2 | 7351 | 7 | 2 |
| ABCB1B | 18669 | 6 | 2 |
| ABCC3 | 8714 | 6 | 2 |
| ABCG2 | 9429 | 6 | 3 |
| ACOT1 | 641371 | 6 | 1 |
| ACOT2 | 10965 | 6 | 1 |
| ACTB | 60 | 6 | 3 |
| ADAMTS1 | 9510 | 6 | 2 |
| AIFM1 | 9131 | 6 | 1 |
| ALDH3A2 | 224 | 6 | 1 |
| APOA4 | 337 | 6 | 2 |
| ATF6 | 22926 | 6 | 2 |
| ATM | 472 | 6 | 3 |
| BAD | 572 | 6 | 2 |
| BCL2L10 | 10017 | 6 | 1 |
| CAV1 | 857 | 6 | 4 |
| CCL5 | 6352 | 6 | 1 |
| CDKN2A | 1029 | 6 | 2 |
| COX4I1 | 1327 | 6 | 2 |
| CTSL | 1514 | 6 | 3 |
| CXCL1 | 2919 | 6 | 3 |
| CXCL10 | 3627 | 6 | 3 |
| CXCL8 | 3576 | 6 | 2 |
| CYP26B1 | 56603 | 6 | 2 |
| DDIT4 | 54541 | 6 | 2 |
| DHCR7 | 1717 | 6 | 2 |
| DLL1 | 28514 | 6 | 3 |
| DUSP1 | 1843 | 6 | 3 |
| ECH1 | 1891 | 6 | 2 |
| FABP1 | 2168 | 6 | 4 |
| FGF9 | 2254 | 6 | 2 |
| FIGLA | 344018 | 6 | 1 |
| FOXL2 | 668 | 6 | 3 |
| G6PC1 | 2538 | 6 | 3 |
| GCK | 2645 | 6 | 3 |
| GSTA2 | 2939 | 6 | 2 |
| GSTK1 | 373156 | 6 | 3 |
| GSTM2 | 2946 | 6 | 2 |
| HADHA | 3030 | 6 | 2 |
| HES1 | 3280 | 6 | 3 |
| HP | 3240 | 6 | 1 |
| HPGD | 3248 | 6 | 3 |
| HSD11B2 | 3291 | 6 | 3 |
| HSD17B4 | 3295 | 6 | 2 |
| HSP90AB1 | 3326 | 6 | 2 |
| ID1 | 3397 | 6 | 3 |
| IGF2R | 3482 | 6 | 3 |
| IL18 | 3606 | 6 | 2 |
| INHA | 3623 | 6 | 3 |
| INS2 | 16334 | 6 | 2 |
| ISYNA1 | 51477 | 6 | 2 |
| ITGA6 | 3655 | 6 | 3 |
| JAGN1 | 84522 | 6 | 2 |
| KIF5C | 3800 | 6 | 3 |
| KNDC1 | 85442 | 6 | 3 |
| LCN2 | 3934 | 6 | 2 |
| LPIN1 | 23175 | 6 | 1 |
| MAFA | 389692 | 6 | 3 |
| MAP2K6 | 5608 | 6 | 2 |
| MEF2C | 4208 | 6 | 1 |
| MIR106B | 406900 | 6 | 3 |
| MYD88 | 4615 | 6 | 2 |
| NR4A3 | 8013 | 6 | 3 |
| OPA1 | 4976 | 6 | 2 |
| OXTR | 5021 | 6 | 2 |
| PAX6 | 5080 | 6 | 3 |
| PIK3CA | 5290 | 6 | 2 |
| PLIN1 | 5346 | 6 | 1 |
| PTCH1 | 5727 | 6 | 3 |
| S100A10 | 6281 | 6 | 2 |
| SCD | 6319 | 6 | 3 |
| SH2D1A | 4068 | 6 | 1 |
| SNAI1 | 6615 | 6 | 3 |
| SRC | 6714 | 6 | 3 |
| TBC1D4 | 9882 | 6 | 2 |
| TLX1 | 3195 | 6 | 2 |
| TPO | 7173 | 6 | 2 |
| TUBB4A | 10382 | 6 | 3 |
| TXNRD1 | 7296 | 6 | 3 |

**Table S4. DEHP targets from pharmmapper database.**

| **Pharma Model** | **Num Feature** | **Fit** | **Norm Fit** | **zscore** | **Num Hydrophobic** | **Num HB Acceptor** | **Num HB Donor** | **Num Positive** | **Num Negative** | **Num Aromatic** | **Name** | **Class** | **Uniplot** | **Function** | **Disease** | **Idication** |  |
| --- | --- | --- | --- | --- | --- | --- | --- | --- | --- | --- | --- | --- | --- | --- | --- | --- | --- |
| 1f86_v | 3 | 2.985 | 0.995 | 0.559468 | 3 | 0 | 0 | 0 | 0 | 0 | Transthyretin | TRANSPORT PROTEIN | TTHY_HUMAN | Thyroid hormone-binding protein. Probably transports thyroxine from the bloodstream to the brain. | Defects in TTR are the cause of amyloidosis type 1 (AMYL1) [MIM:176300]. AMYL1 is a hereditary generalized amyloidosis due to transthyretin amyloid deposition. Protein fibrils can form in different tissues leading to amyloid polyneuropathies, amyloidotic cardiomyopathy, carpal tunnel syndrome, systemic senile amyloidosis.;Defects in TTR are the cause of amyloidosis type 7 (AMYL7) [MIM:105210]; also known as leptomeningeal amyloidosis or meningocerebrovascular amyloidosis. AMYL7 is a form of hereditary transthyretin amyloidosis characterized by primary involvement of the central nervous system. Neuropathologic examination shows amyloid in the walls of leptomeningeal vessels, in pia arachnoid, and subpial deposits. Some patients also develop vitreous amyloid deposition that leads to visual impairment (oculoleptomeningeal amyloidosis). Clinical features include seizures, stroke-like episodes, dementia, psychomotor deterioration, variable amyloid deposition in the vitreous humor. Mild systemic amyloidosis may occurr.;Defects in TTR are a cause of hyperthyroxinemia [MIM:176300]. | NONE |  |
| 1j96_v | 3 | 2.975 | 0.9918 | 0.574504 | 3 | 0 | 0 | 0 | 0 | 0 | Aldo-keto reductase family 1 member C2 | OXIDOREDUCTASE | AK1C2_HUMAN | Works in concert with the 5-alpha/5-beta-steroid reductases to convert steroid hormones into the 3-alpha/5-alpha and 3-alpha/5-beta-tetrahydrosteroids. Catalyzes the inactivation of the most potent androgen 5-alpha-dihydrotestosterone (5-alpha- DHT) to 5- | NONE | NONE |  |
| 1no9_v | 3 | 2.972 | 0.9907 | 0.639987 | 2 | 1 | 0 | 0 | 0 | 0 | Prothrombin | HYDROLASE | THRB_HUMAN | Thrombin, which cleaves bonds after Arg and Lys, converts fibrinogen to fibrin and activates factors V, VII, VIII, XIII, and, in complex with thrombomodulin, protein C. Functions in blood homeostasis, inflammation and wound healing. | Defects in F2 are the cause of various forms of dysprothrombinemia [MIM:176930].;Genetic variations in F2 may be a cause of susceptibility to ischemic stroke [MIM:601367]; also known as cerebrovascular accident or cerebral infarction. A stroke is an acute neurologic event leading to death of neural tissue of the brain and resulting in loss of motor, sensory and/or cognitive function. Ischemic strokes, resulting from vascular occlusion, is considered to be a highly complex disease consisting of a group of heterogeneous disorders with multiple genetic and environmental risk factors. | Deep vein thrombosis;Thrombocytopenia;Cerebrovascular disease;Wound healing;Ebola virus infection;Bone injury;Cerebrovascular ischemia;Restenosis;Thrombosis;Diabetic foot ulcer;Ischemic heart disease;Marburg virus infection;Myocardial disease;Embolism and thrombosis;Myocardial infarction;Cartilage disease;Thromboembolism;Coronary artery disease;Hemophilia;Blood clotting disorder;Bleeding;Atrial fibrillation;Skin burns;Disseminated intravascular coagulation;Connective tissue disease;Lung embolism;Cardiovascular disease;Sepsis;Angina;Infarction |  |
| 1w8m_v | 3 | 2.947 | 0.9825 | 0.611485 | 1 | 2 | 0 | 0 | 0 | 0 | Peptidyl-prolyl cis-trans isomerase A | NONE | P62937 | Posttranslational modification, protein turnover, chaperones | NONE | For treatment of transplant rejection, rheumatoid arthritis, severe psoriasis |  |
| 1pmv_v | 3 | 2.946 | 0.982 | 0.308116 | 2 | 1 | 0 | 0 | 0 | 0 | Mitogen-activated protein kinase 10 | TRANSFERASE | MK10_HUMAN | Responds to activation by environmental stress and pro- inflammatory cytokines by phosphorylating a number of transcription factors, primarily components of AP-1 such as c-Jun and ATF2 and thus regulates AP-1 transcriptional activity. Required for stress- | A chromosomal rearrangement involving MAPK10 is a cause of epileptic encephalopathy Lennox-Gastaut type [MIM:606369]. Translocation t(Y;4)(q11.2;q21) which causes MAPK10 truncation. Epileptic encephalopathies of the Lennox-Gastaut group are childhood epileptic disorders characterized by severe psychomotor delay and seizures. | Central nervous system disease;Inflammation;Pulmonary fibrosis;Ischemia;Cerebrovascular ischemia;Neurodegenerative disease |  |
| 2o65_v | 3 | 2.94 | 0.9802 | 0.522159 | 3 | 0 | 0 | 0 | 0 | 0 | Proto-oncogene serine/threonine-protein kinase Pim-1 | TRANSFERASE | PIM1_HUMAN | Plays a role in signal transduction in blood cells. Contributes to both cell proliferation and survival and thus provide a selective advantage in tumorigenesis. May affect the structure or silencing of chromatin by phosphorylating HP1 gamma/CBX3. | NONE | Cancer |  |
| 1p0i_v | 3 | 2.936 | 0.9785 | 0.577093 | 1 | 2 | 0 | 0 | 0 | 0 | Cholinesterase | HYDROLASE | CHLE_HUMAN | NONE | Defects in BCHE are the cause of butyrylcholinesterase deficiency (BChE deficiency) [MIM:177400]. BChE deficiency is a metabolic disorder characterized by prolonged apnoea after the use of certain anesthetic drugs, including the muscle relaxants succinylcholine or mivacurium and other ester local anesthetics. The duration of the prolonged apnoea varies significantly depending on the extent of the enzyme deficiency. BChE deficiency is a multifactorial disorder. The hereditary condition is transmitted as an autosomal recessive trait. | Neurotoxicity, drug-induced;Toxicity;Alzheimers disease |  |
| 2jbp_v | 3 | 2.925 | 0.9749 | 0.425278 | 3 | 0 | 0 | 0 | 0 | 0 | MAP kinase-activated protein kinase 2 | NONE | P49137 | Involved in protein kinase activity | NONE | NONE |  |
| 1reu_v | 3 | 2.925 | 0.9748 | 0.0820348 | 3 | 0 | 0 | 0 | 0 | 0 | Bone morphogenetic protein 2 | HORMONE/GROWTH FACTOR | BMP2_HUMAN | Induces cartilage and bone formation. | NONE | Bone disease;Musculoskeletal disease;Periodontal disease;Bone injury;Osteoporosis |  |
| 1eub_v | 3 | 2.924 | 0.9746 | 0.392593 | 3 | 0 | 0 | 0 | 0 | 0 | Collagenase 3 | HYDROLASE/HYDROLASE INHIBITOR | MMP13_HUMAN | Degrades collagen type I. Does not act on gelatin or casein. Could have a role in tumoral process. | Defects in MMP13 are the cause of spondyloepimetaphyseal dysplasia type 2 (SEMD2) [MIM:602111]; also known as spondyloepimetaphyseal dysplasia type Missouri. SEMDs are a heterogeneous group of skeletal disorders characterized by defective growth and modeling of the spine and long bones. The SEMDs are distinguished from the spondylometaphyseal dysplasias and the spondyloepiphyseal dysplasias by the combined involvement of the epiphyses and metaphyses. The 3 disorders have malformations of the vertebrae in common. | NONE |  |
| 1bm6_v | 3 | 2.924 | 0.9745 | 0.439109 | 2 | 1 | 0 | 0 | 0 | 0 | Stromelysin-1 | METALLOPROTEASE | MMP3_HUMAN | Can degrade fibronectin, laminin, gelatins of type I, III, IV, and V; collagens III, IV, X, and IX, and cartilage proteoglycans. Activates procollagenase. | NONE | NONE |  |
| 2brg_v | 3 | 2.918 | 0.9725 | 0.643065 | 2 | 1 | 0 | 0 | 0 | 0 | Serine/threonine-protein kinase Chk1 | TRANSFERASE | CHK1_HUMAN | Required for checkpoint mediated cell cycle arrest in response to DNA damage or the presence of unreplicated DNA. May also negatively regulate cell cycle progression during unperturbed cell cycles. Recognizes the substrate consensus sequence [R-X-X- S/T]. | NONE | Cancer;Solid tumor |  |
| 1pq9_v | 3 | 2.917 | 0.9722 | 0.563935 | 1 | 2 | 0 | 0 | 0 | 0 | Oxysterols receptor LXR-beta | TRANSCRIPTION REGULATION | NR1H2_HUMAN | Orphan receptor. Binds preferentially to double-stranded oligonucleotide direct repeats having the consensus half-site sequence 5-AGGTCA-3 and 4-nt spacing (DR-4). | NONE | Lipid metabolism disorder;Atherosclerosis |  |
| 1if4_v | 3 | 2.909 | 0.9697 | 0.443114 | 2 | 1 | 0 | 0 | 0 | 0 | Carbonic anhydrase 2 | LYASE | CAH2_HUMAN | Essential for bone resorption and osteoclast differentiation (By similarity). Reversible hydration of carbon dioxide. | Defects in CA2 are the cause of autosomal recessive osteopetrosis type 3 (OPTB3) [MIM:259730]; also known as osteopetrosis with renal tubular acidosis, carbonic anhydrase II deficiency syndrome, Guibaud-Vainsel syndrome or marble brain disease. Osteopetrosis is a rare genetic disease characterized by abnormally dense bone, due to defective resorption of immature bone. The disorder occurs in two forms: a severe autosomal recessive form occurring in utero, infancy, or childhood, and a benign autosomal dominant form occurring in adolescence or adulthood. Autosomal recessive osteopetrosis is usually associated with normal or elevated amount of non-functional osteoclasts. OPTB3 is associated with renal tubular acidosis, cerebral calcification (marble brain disease) and in some cases with mental retardation. | NONE |  |
| 1uki_v | 3 | 2.908 | 0.9693 | 0.39523 | 2 | 1 | 0 | 0 | 0 | 0 | Mitogen-activated protein kinase 8 | TRANSFERASE | MK08_HUMAN | Responds to activation by environmental stress and pro- inflammatory cytokines by phosphorylating a number of transcription factors, primarily components of AP-1 such as JUN, JDP2 and ATF2 and thus regulates AP-1 transcriptional activity. In T-cells, JNK1;JNK1 isoforms display different binding patterns: beta-1 preferentially binds to c-Jun, whereas alpha-1, alpha-2, and beta- 2 have a similar low level of binding to both c-Jun or ATF2. However, there is no correlation between binding and phosphorylation, | NONE | Psoriasis;Asthma;Cancer;Neurodegenerative disease;Inflammation;Liver disease;Acute myelogenous leukemia;Diabetes mellitus;Pulmonary fibrosis;Ischemia |  |
| 1shj_v | 3 | 2.902 | 0.9673 | 0.335214 | 3 | 0 | 0 | 0 | 0 | 0 | Caspase-7 | HYDROLASE | CASP7_HUMAN | Involved in the activation cascade of caspases responsible for apoptosis execution. Cleaves and activates sterol regulatory element binding proteins (SREBPs). Proteolytically cleaves poly(ADP-ribose) polymerase (PARP) at a 216-Asp- | -Gly- 217 bond. Overe | NONE | NONE |
| 1rs0_v | 3 | 2.862 | 0.954 | 0.25056 | 2 | 1 | 0 | 0 | 0 | 0 | Complement factor B | HYDROLASE | CFAB_HUMAN | Factor B which is part of the alternate pathway of the complement system is cleaved by factor D into 2 fragments: Ba and Bb. Bb, a serine protease, then combines with complement factor 3b to generate the C3 or C5 convertase. It has also been implicated in | NONE | NONE |  |
| 3eqm_v | 3 | 2.862 | 0.954 | 0.448557 | 2 | 1 | 0 | 0 | 0 | 0 | Cytochrome P450 19A1 | NONE | P11511 | Catalyzes the formation of aromatic C18 estrogens from C19 androgens. | NONE | NONE |  |
| 1lbk_v | 3 | 2.822 | 0.9407 | 0.195159 | 1 | 2 | 0 | 0 | 0 | 0 | Glutathione S-transferase P | TRANSFERASE | GSTP1_HUMAN | Conjugation of reduced glutathione to a wide number of exogenous and endogenous hydrophobic electrophiles. | NONE | NONE |  |
| 1klt_v | 3 | 2.805 | 0.9349 | 0.401306 | 1 | 2 | 0 | 0 | 0 | 0 | Chymase | SERINE PROTEASE | CMA1_HUMAN | Major secreted protease of mast cells with suspected roles in vasoactive peptide generation, extracellular matrix degradation, and regulation of gland secretion. | NONE | Asthma;Atopic dermatitis;Atherosclerosis;Thrombosis;Bacterial infection;Fungal infection;Inflammation;Congestive heart failure;Chronic obstructive pulmonary disease;Viral infection;HIV infection;Cardiovascular disease |  |
| 2wmt_v | 3 | 2.795 | 0.9318 | 0.290494 | 2 | 1 | 0 | 0 | 0 | 0 | NONE | NONE | NONE | NONE | NONE | NONE |  |
| 2ovm_v | 3 | 2.779 | 0.9262 | 0.360639 | 2 | 1 | 0 | 0 | 0 | 0 | Progesterone receptor | TRANSCRIPTION | PRGR_HUMAN | The steroid hormones and their receptors are involved in the regulation of eukaryotic gene expression and affect cellular proliferation and differentiation in target tissues. | NONE | Uterine fibroids;Menstruation disorder;Infertility;Acne;Breast tumor;Female contraception;Neoplasm;Planned abortion;Dysmenorrhea;Endometriosis;Hormone deficiency;Unidentified indication;Gynecological disorder;Musculoskeletal disease;Estrogen deficiency;Cancer;Contraception;Amenorrhea;Female infertility;Carcinoma;Menopause;Premenstrual syndrome;Anesthesia;Age related macular degeneration;Osteoporosis |  |
| 3gam_v | 3 | 2.772 | 0.924 | 0.460476 | 2 | 1 | 0 | 0 | 0 | 0 | Ribosyldihydronicotinamide dehydrogenase [quinone] | NONE | P16083 | Involved in oxidoreductase activity | NONE | Some evidence suggests that NADH might be useful in treating Parkinsons disease, chronic fatigue syndrome, Alzheimers disease and cardiovascular disease. |  |
| 3dej_v | 3 | 2.733 | 0.9112 | 0.14801 | 2 | 1 | 0 | 0 | 0 | 0 | Caspase-3 | HYDROLASE, APOPTOSIS | CASP3_HUMAN | Involved in the activation cascade of caspases responsible for apoptosis execution. At the onset of apoptosis it proteolytically cleaves poly(ADP-ribose) polymerase (PARP) at a 216-Asp- | -Gly-217 bond. Cleaves and activates sterol regulatory element bind | NONE | Cancer;Liver disease;Cognitive disorder;Neurodegenerative disease;Cerebrovascular ischemia |
| 1xf0_v | 3 | 2.719 | 0.9063 | 0.199732 | 2 | 1 | 0 | 0 | 0 | 0 | Aldo-keto reductase family 1 member C3 | OXIDOREDUCTASE | AK1C3_HUMAN | Catalyzes the conversion of aldehydes and ketones to alcohols. Catalyzes the reduction of prostaglandin (PG) D2, PGH2 and phenanthrenequinone (PQ) and the oxidation of 9-alpha,11-beta- PGF2 to PGD2. Functions as a bi-directional 3-alpha-, 17-beta- and 20- | NONE | NONE |  |
| 1p49_v | 3 | 2.7 | 0.9001 | -0.231525 | 3 | 0 | 0 | 0 | 0 | 0 | Steryl-sulfatase | HYDROLASE | STS_HUMAN | Conversion of sulfated steroid precursors to estrogens during pregnancy. | Defects in STS are the cause of ichthyosis X-linked (IXL) [MIM:308100]. Ichthyosis X-linked is a keratinization disorder manifesting with mild erythroderma and generalized exfoliation of the skin within a few weeks after birth. Affected boys later develop large, polygonal, dark brown scales, especially on the neck, extremities, trunk, and buttocks. | Cognitive disorder;Cancer;Breast tumor |  |
| 1e7a_v | 3 | 2.699 | 0.8996 | 0.0426612 | 3 | 0 | 0 | 0 | 0 | 0 | Serum albumin | CARRIER PROTEIN | ALBU_HUMAN | Serum albumin, the main protein of plasma, has a good binding capacity for water, Ca(2+), Na(+), K(+), fatty acids, hormones, bilirubin and drugs. Its main function is the regulation of the colloidal osmotic pressure of blood. | Defects in ALB are a cause of familial dysalbuminemic hyperthyroxinemia (FDH) [MIM:103600]. FDH is a form of euthyroid hyperthyroxinemia that is due to increased affinity of ALB for T(4). It is the most common cause of inherited euthyroid hyperthyroxinemia in Caucasian population.;A variant structure of albumin could lead to increased binding of zinc resulting in an asymptomatic augmentation of zinc concentration in the blood [MIM:194470]. | NONE |  |
| 2fky_v | 4 | 3.553 | 0.8882 | 1.72799 | 4 | 0 | 0 | 0 | 0 | 0 | Kinesin-like protein KIF11 | CELL CYCLE | KIF11_HUMAN | Motor protein required for establishing a bipolar spindle. Blocking of KIF11 prevents centrosome migration and arrest cells in mitosis with monoastral microtubule arrays. | NONE | Melanoma;Hodgkins disease;Acute leukemia;Breast tumor;Head and neck tumor;Hepatocellular carcinoma;Ovary tumor;Prostate tumor;Solid tumor;Non-Hodgkin lymphoma;Cancer;Non-small-cell lung cancer;Renal cell carcinoma;Colorectal tumor |  |
| 2agt_v | 3 | 2.663 | 0.8877 | 0.134904 | 2 | 1 | 0 | 0 | 0 | 0 | Aldose reductase | OXIDOREDUCTASE | ALDR_HUMAN | Catalyzes the NADPH-dependent reduction of a wide variety of carbonyl-containing compounds to their corresponding alcohols with a broad range of catalytic efficiencies. | In diabetes and galactosemia, increased AR activity leads to high levels of sorbitol and galactitol, respectively, in the cells of many tissues. Accumulation of sugar alcohols has been shown to cause osmotic cataracts in the lens. AR is also thought to play a key role in diabetic complications of three other target tissues, namely, nerve, kidney and retina. | Neuropathy;Diabetic neuropathy;Diabetic foot ulcer;Diabetic complication;Diabetic nephropathy;Retinopathy;Ocular disease;Central nervous system disease;Diabetic retinopathy;Diabetes mellitus;Diabetic cataract;Cataract |  |
| 2oji_v | 3 | 2.646 | 0.882 | 0.0149691 | 2 | 1 | 0 | 0 | 0 | 0 | Mitogen-activated protein kinase 1 | TRANSFERASE | MK01_HUMAN | Involved in both the initiation and regulation of meiosis, mitosis, and postmitotic functions in differentiated cells by phosphorylating a number of transcription factors such as ELK1. Phosphorylates EIF4EBP1; required for initiation of translation. Phosp | NONE | NONE |  |
| 1l6l_v | 3 | 2.624 | 0.8746 | -0.371473 | 3 | 0 | 0 | 0 | 0 | 0 | Apolipoprotein A-II | LIPID TRANSPORT | APOA2_HUMAN | May stabilize HDL (high density lipoprotein) structure by its association with lipids, and affect the HDL metabolism. | NONE | NONE |  |
| 2ama_v | 3 | 2.62 | 0.8735 | -0.0303766 | 2 | 1 | 0 | 0 | 0 | 0 | Androgen receptor | HORMONE/GROWTH FACTOR RECEPTOR | ANDR_HUMAN | Steroid hormone receptors are ligand-activated transcription factors that regulate eukaryotic gene expression and affect cellular proliferation and differentiation in target tissues. Transcription factor activity is modulated by bound coactivator and core | Genetic variation in AR can be responsible of androgenetic alopecia (AGA) [MIM:109200].;Defects in AR are the cause of androgen insensibility syndrome (AIS) [MIM:300068]; previously known as testicular feminization syndrome (TFM). It can be complete (CAIS) when external genitalia are phenotypically female; or partial (PAIS) when external genitalia are substantively ambiguous or mild (MAIS) when external genitalia are normal male or nearly so.;Defects in AR are the cause of X-linked spinal and bulbar muscular atrophy (SBMA) [MIM:313200]; also known as Kennedy disease. In SBMA patients the number of Gln ranges from 40 to 52. Longer expansions result in earlier onset and more severe clinical manifestations of the disease.;Defects in AR may play a role in metastatic prostate cancer. The mutated receptor stimulates prostate growth and metastases development despite of androgen ablation. This treatment can reduce primary and metastatic lesions probably by inducing apoptosis of tumor cells when they express the wild-type receptor.;Defects in AR may be the cause of infertility male syndrome [MIM:308370]; also called androgen insensitivity. It is characterized by azoospermia, elevated testosterone and luteinizing hormone plasma levels and an abnormal androgen receptor.;Defects in AR are the cause of Reifenstein syndrome [MIM:312300]; also known as partial androgen insensitivity. The features of this form of male pseudohermaphroditism are hypospadias, hypogonadism, gynecomastia, normal XY karyotype, and a pedigree pattern consistent with X-linked recessive inheritance. | Hypoactive sexual desire disorder;Hepatitis B virus infection;Cardiac failure;Andrology;Hirsutism;Female sexual dysfunction;Psoriasis;Reperfusion injury;Plasmodium infection;Atherosclerosis;Prostate hyperplasia;Fatigue;Male sexual dysfunction;Autoimmune disease;Testosterone deficiency;Uterine fibroids;Metabolic disorder;Myalgia;Obesity;Gynecological disorder;Contraception;Cancer;Hypercholesterolemia;Skin burns;Seborrhea;Growth disorder;Bone tumor;Muscular dystrophy;Erectile dysfunction;Systemic lupus erythematosus;Asthma;Wound healing;Cerebrovascular ischemia;Acne;Hypogonadism;HIV infection;Neoplasm;Dermatological disease;Prostate disease;Multiple sclerosis;Lacrimal gland disease;Alopecia;Mycobacterium tuberculosis infection;Ovary cyst;Carcinoma;Cardiovascular disease;Cachexia;Muscle wasting disease;Cervical dystonia;Anemia;Endocrine disease;Non-insulin dependent diabetes;Breast tumor;Female contraception;Fungal infection;Cystic fibrosis;Prostate tumor;Estrogen deficiency;Endometriosis;Hormone deficiency;Turners syndrome;Keratosis;Premenstrual syndrome;Hepatitis C virus infection;Benign tumor;Osteoporosis;Male contraception |  |
| 1unh_v | 3 | 2.587 | 0.8624 | -0.0432327 | 2 | 1 | 0 | 0 | 0 | 0 | Cyclin-dependent kinase 5 activator 1 | COMPLEX(KINASE/ACTIVATOR) | CD5R1_HUMAN | p35 is a neuron specific activator of CDK5. The complex p35/CDK5 is required for neurite outgrowth and cortical lamination. Activator of TPKII. | Cleavage of p35 to p25 may be involved in the pathogenesis of Alzheimer disease. The p25 form accumulates in neurons in the brain of patients with Alzheimer disease, but not in normal brain. This accumulation correlates with an increase in CDK5 kinase activity. Application of amyloid beta peptide A- beta(1-42) induced the conversion of p35 to p25 in primary cortical neurons. Expression of the p25/Cdk5 complex in cultured primary neurons induces cytoskeletal disruption, morphological degeneration and apoptosis. | Neurodegenerative disease |  |
| 2vwu_v | 3 | 2.436 | 0.8119 | -0.493362 | 2 | 1 | 0 | 0 | 0 | 0 | Ephrin type-B receptor 4 | TRANSFERASE | EPHB4_HUMAN | Receptor for members of the ephrin-B family. Binds to ephrin-B2. May have a role in events mediating differentiation and development. | NONE | Ocular disease;Rheumatoid arthritis;Cancer |  |
| 2cji_v | 5 | 3.952 | 0.7904 | 3.13777 | 4 | 1 | 0 | 0 | 0 | 0 | Coagulation factor X | HYDROLASE | FA10_HUMAN | Factor Xa is a vitamin K-dependent glycoprotein that converts prothrombin to thrombin in the presence of factor Va, calcium and phospholipid during blood clotting. | NONE | Thromboembolism |  |
| 1oj9_v | 3 | 2.335 | 0.7785 | -0.900072 | 3 | 0 | 0 | 0 | 0 | 0 | Amine oxidase [flavin-containing] B | OXIDOREDUCTASE | AOFB_HUMAN | Catalyzes the oxidative deamination of biogenic and xenobiotic amines and has important functions in the metabolism of neuroactive and vasoactive amines in the central nervous system and peripheral tissues. MAOB preferentially degrades benzylamine and phe | NONE | Alzheimers disease;Cocaine addiction;Major depressive disorder;Epilepsy;Neurodegenerative disease;Attention deficit hyperactivity disorder;Head injury;Parkinsons disease;Dementia;Nicotine dependence;Cerebrovascular ischemia |  |
| 1csb_v | 4 | 3.112 | 0.778 | 1.07261 | 1 | 3 | 0 | 0 | 0 | 0 | Cathepsin B | HYDROLASE (THIOL PROTEASE) | CATB_HUMAN | Thiol protease which is believed to participate in intracellular degradation and turnover of proteins. Has also been implicated in tumor invasion and metastasis. | NONE | Myocardial infarction;Arthritis;Inflammation;Cancer |  |
| 1hak_v | 4 | 2.997 | 0.7493 | 0.149911 | 3 | 1 | 0 | 0 | 0 | 0 | Annexin A5 | CALCIUM/PHOSPHOLIPID-BINDING | ANXA5_HUMAN | This protein is an anticoagulant protein that acts as an indirect inhibitor of the thromboplastin-specific complex, which is involved in the blood coagulation cascade. | NONE | NONE |  |
| 1j78_v | 4 | 2.997 | 0.7493 | -0.273372 | 4 | 0 | 0 | 0 | 0 | 0 | Vitamin D-binding protein | TRANSPORT, LIGAND BINDING PROTEIN | VTDB_HUMAN | Multifunctional protein found in plasma, ascitic fluid, cerebrospinal fluid, and urine and on the surface of many cell types. In plasma, it carries the vitamin D sterols and prevents polymerization of actin by binding its monomers. DBP associates with mem | NONE | NONE |  |
| 1zyj_v | 4 | 2.995 | 0.7487 | 0.32554 | 3 | 1 | 0 | 0 | 0 | 0 | Mitogen-activated protein kinase 14 | NONE | Q16539 | Involved in MAP kinase activity | NONE | NONE |  |
| 1tow_v | 4 | 2.989 | 0.7473 | 0.205669 | 2 | 1 | 0 | 0 | 1 | 0 | Fatty acid-binding protein, adipocyte | LIPID TRANSPORT | FABP4_HUMAN | Lipid transport protein in adipocytes. Binds both long chain fatty acids and retinoic acid. Delivers long-chain fatty acids and retinoic acid to their cognate receptors in the nucleus (By similarity). | NONE | Non-insulin dependent diabetes |  |
| 3dy6_v | 4 | 2.984 | 0.7459 | 0.524998 | 3 | 1 | 0 | 0 | 0 | 0 | Peroxisome proliferator-activated receptor delta | NONE | Q03181 | Involved in DNA binding | NONE | EPA can be used for lowering elevated triglycerides in those who are hyperglyceridemic. In addition, EPA may play a therapeutic role in patients with cystic fibrosis by reducing disease severity and may play a similar role in type 2 diabetics in slowing the progression of diabetic nephropathy. |  |
| 2b53_v | 4 | 2.979 | 0.7447 | 0.395295 | 4 | 0 | 0 | 0 | 0 | 0 | Cell division protein kinase 2 | NONE | P24941 | Involved in protein kinase activity | NONE | NONE |  |
| 1o4f_v | 4 | 2.973 | 0.7432 | 0.136241 | 1 | 2 | 0 | 0 | 1 | 0 | Proto-oncogene tyrosine-protein kinase Src | SIGNALING PROTEIN | SRC_HUMAN | NONE | NONE | Hypercalcemia;Cancer;Osteoporosis;Cerebrovascular ischemia;Bone metastases;Metastasis;Solid tumor |  |
| 1i7i_v | 4 | 2.958 | 0.7395 | 0.526617 | 3 | 0 | 0 | 0 | 1 | 0 | Peroxisome proliferator-activated receptor gamma | TRANSCRIPTION | PPARG_HUMAN | Receptor that binds peroxisome proliferators such as hypolipidemic drugs and fatty acids. Once activated by a ligand, the receptor binds to a promoter element in the gene for acyl-CoA oxidase and activates its transcription. It therefore controls the pero | Defects in PPARG can lead to type 2 insulin-resistant diabetes and hyptertension.;Defects in PPARG may be associated with susceptibility to obesity [MIM:601665].;Defects in PPARG may be associated with colon cancer.;Defects in PPARG are the cause of familial partial lipodystrophy type 3 (FPLD3) [MIM:604367]. Familial partial lipodystrophies (FPLD) are a heterogeneous group of genetic disorders characterized by marked loss of subcutaneous (sc) fat from the extremities. Affected individuals show an increased preponderance of insulin resistance, diabetes mellitus and dyslipidemia.;Variation in PPARG is associated with carotid intimal medial thickness 1 (CIMT1) [MIM:609338]. CIMT is a measure of atherosclerosis that is independently associated with traditional atherosclerotic cardiovascular disease risk factors and coronary atherosclerotic burden. 35 to 45% of the variability in multivariable-adjusted CIMT is explained by genetic factors. | Asthma;Cerebrovascular ischemia;Conjunctivitis;Inflammation;Multiple sclerosis;Fibrosis;Unidentified indication;Psoriasis;Heart arrhythmia;Rheumatoid arthritis;Atherosclerosis;Corneal disease;Cardiovascular disease;Ulcerative colitis;Alzheimers disease;Infertility;Syndrome X;Lipid metabolism disorder;Non-insulin dependent diabetes;Metabolic disorder;Diabetic complication;Hyperlipidemia;Lacrimal gland disease;Myocardial infarction;Obesity;Coronary artery disease;Insulin dependent diabetes;Vascular disease;Cancer;Cognitive disorder;Hypertension;Diabetes mellitus;Osteoporosis;Dermatological disease |  |
| 2uzd_v | 3 | 2.206 | 0.7354 | -0.848831 | 2 | 1 | 0 | 0 | 0 | 0 | Cyclin-A2 | TRANSFERASE | CCNA2_HUMAN | Essential for the control of the cell cycle at the G1/S (start) and the G2/M (mitosis) transitions. | NONE | NONE |  |
| 1zs0_v | 4 | 2.937 | 0.7342 | 0.566286 | 2 | 1 | 0 | 0 | 0 | 1 | Neutrophil collagenase | HYDROLASE | MMP8_HUMAN | Can degrade fibrillar type I, II, and III collagens. | NONE | NONE |  |
| 2j5f_v | 4 | 2.936 | 0.7339 | 0.43268 | 3 | 1 | 0 | 0 | 0 | 0 | Epidermal growth factor receptor | TRANSFERASE | EGFR_HUMAN | Receptor for EGF, but also for other members of the EGF family, as TGF-alpha, amphiregulin, betacellulin, heparin-binding EGF-like growth factor, GP30 and vaccinia virus growth factor. Is involved in the control of cell growth and differentiation. Phospho;Isoform 2/truncated isoform may act as an antagonist. | Defects in EGFR are associated with lung cancer [MIM:211980]. | Squamous cell carcinoma;Arteriosclerosis;Melanoma;Central nervous system tumor;Diabetes mellitus;Esophagus tumor;Ischemic heart disease;Brain tumor;Nasopharyngeal carcinoma;Liver tumor;Pancreas tumor;Lung tumor;Psoriasis;Bladder tumor;Esophageal disease;Mesothelioma;Atherosclerosis;Multiple myeloma;Bacterial infection;Uterine cervix tumor;Nasopharynx tumor;Carcinoma;Restenosis;Stomach tumor;Thyroid tumor;Small-cell lung cancer;Renal disease;Nervous system tumor;Hepatobiliary system tumor;Breast tumor;Head and neck tumor;Ovary tumor;Prostate tumor;Renal tumor;Solid tumor;Colon tumor;Glioma;Cancer;Non-small-cell lung cancer;Metastasis;Renal cell carcinoma;Glioblastoma;Colorectal tumor;Neoplasm;Sarcoma;Hypercholesterolemia |  |
| 1d3g_v | 4 | 2.93 | 0.7324 | 0.3269 | 3 | 0 | 1 | 0 | 0 | 0 | Dihydroorotate dehydrogenase, mitochondrial | OXIDOREDUCTASE | PYRD_HUMAN | NONE | NONE | NONE |  |
| 1a8j_v | 4 | 2.923 | 0.7309 | 0.660862 | 1 | 2 | 0 | 1 | 0 | 0 | Ig lambda chain V-II region MGC | NONE | P01709 | V region of the variable domain of immunoglobulin light chains that participates in the antigen recognition (PubMed:24600447). Immunoglobulins, also known as antibodies, are membrane-bound or secreted glycoproteins produced by B lymphocytes. In the recognition phase of humoral immunity, the membrane-bound immunoglobulins serve as receptors which, upon binding of a specific antigen, trigger the clonal expansion and differentiation of B lymphocytes into immunoglobulins-secreting plasma cells. Secreted immunoglobulins mediate the effector phase of humoral immunity, which results in the elimination of bound antigens (PubMed:20176268, PubMed:22158414). The antigen binding site is formed by the variable domain of one heavy chain, together with that of its associated light chain. Thus, each immunoglobulin has two antigen binding sites with remarkable affinity for a particular antigen. The variable domains are assembled by a process called V-(D)-J rearrangement and can then be subjected to somatic hypermutations which, after exposure to antigen and selection, allow affinity maturation for a particular antigen (PubMed:17576170, PubMed:20176268). | NONE | NONE |  |
| 1dic_v | 4 | 2.92 | 0.7301 | 0.481835 | 2 | 2 | 0 | 0 | 0 | 0 | Complement factor D | SERINE PROTEASE | CFAD_HUMAN | Factor D cleaves factor B when the latter is complexed with factor C3b, activating the C3bbb complex, which then becomes the C3 convertase of the alternate pathway. Its function is homologous to that of C1s in the classical pathway. | Defects in CFD are the cause of complement factor D deficiency [MIM:134350]. This deficiency predisposes to invasive meningococcal disease. | Autoimmune disease;Reperfusion injury;Age related macular degeneration |  |
| 1wok_v | 5 | 3.642 | 0.7285 | 2.13074 | 3 | 1 | 1 | 0 | 0 | 0 | Poly [ADP-ribose] polymerase 1 | NONE | P09874 | Involved in DNA binding | NONE | NONE |  |
| 1dig_v | 4 | 2.909 | 0.7273 | 0.664762 | 1 | 2 | 0 | 0 | 0 | 1 | C-1-tetrahydrofolate synthase, cytoplasmic | NONE | P11586 | Nucleotide transport and metabolism | NONE | For nutritional supplementation, also for treating dietary shortage or imbalance. |  |
| 1gcz_v | 4 | 2.9 | 0.725 | 0.463949 | 2 | 2 | 0 | 0 | 0 | 0 | Macrophage migration inhibitory factor | IMMUNE SYSTEM | MIF_HUMAN | The expression of MIF at sites of inflammation suggest a role for the mediator in regulating the function of macrophage in host defense. Also acts as a phenylpyruvate tautomerase. | Genetic variations in MIF are associated with susceptibility to systemic juvenile rheumatoid arthritis [MIM:604302]. Systemic juvenile rheumatoid arthritis is juvenile chronic arthritis associated with severe, debilitating, extraarticular features, and occasionally fatal complications. Despite medical treatment, many children still experience early joint destruction, necessitating surgical replacement. | Insulin dependent diabetes;Glomerulonephritis;Cancer;Rheumatoid arthritis;Sepsis;Autoimmune disease;Atherosclerosis;Infection;Inflammation;Myocardial infarction;Immune disorder |  |
| 3bmy_v | 4 | 2.899 | 0.7247 | 0.447776 | 2 | 1 | 1 | 0 | 0 | 0 | Heat shock protein HSP 90-alpha | CHAPERONE | HS90A_HUMAN | Molecular chaperone. Has ATPase activity (By similarity). | NONE | NONE |  |
| 1mkp_v | 4 | 2.889 | 0.7223 | 0.323909 | 3 | 0 | 1 | 0 | 0 | 0 | Dual specificity protein phosphatase 6 | HYDROLASE | DUS6_HUMAN | Inactivates MAP kinases. Has a specificity for the ERK family. | NONE | NONE |  |
| 1vjy_v | 4 | 2.886 | 0.7216 | 0.0331732 | 3 | 1 | 0 | 0 | 0 | 0 | TGF-beta receptor type-1 | TRANSFERASE | TGFR1_HUMAN | On ligand binding, forms a receptor complex consisting of two type II and two type I transmembrane serine/threonine kinases. Type II receptors phosphorylate and activate type I receptors which autophosphorylate, then bind and activate SMAD transcriptional | Defects in TGFBR1 are the cause of Loeys-Dietz syndrome type 1A (LDS1A) [MIM:609192]; also known as Furlong syndrome or Loeys-Dietz aortic aneurysm syndrome (LDAS). LDS1 is an aortic aneurysm syndrome with widespread systemic involvement. The disorder is characterized by arterial tortuosity and aneurysms, craniosynostosis, hypertelorism, and bifid uvula or cleft palate. Other findings include exotropy, micrognathia and retrognathia, structural brain abnormalities, intellectual deficit, congenital heart disease, translucent skin, joint hyperlaxity and aneurysm with dissection throughout the arterial tree.;Defects in TGFBR1 are the cause of Loeys-Dietz syndrome type 2A (LDS2A) [MIM:608967]. LDS2 is an aortic aneurysm syndrome with widespread systemic involvement. Physical findings include prominent joint laxity, easy bruising, wide and atrophic scars, velvety and translucent skin with easily visible veins, spontaneous rupture of the spleen or bowel, diffuse arterial aneurysms and dissections, and catastrophic complications of pregnancy, including rupture of the gravid uterus and the arteries, either during pregnancy or in the immediate postpartum period. LDS2 is characterized by the absence of craniofacial abnormalities with the exception of bifid uvula that can be present in some patients.;Defects in TGFBR1 are the cause of aortic aneurysm familial thoracic type 5 (AAT5) [MIM:608967]. Aneurysms and dissections of the aorta usually result from degenerative changes in the aortic wall. Thoracic aortic aneurysms and dissections are primarily associated with a characteristic histologic appearance known as medial necrosis in which there is degeneration and fragmentation of elastic fibers, loss of smooth muscle cells, and an accumulation of basophilic ground substance. | Skin burns;Glomerulonephritis;Wound healing;Renal disease;Scleroderma;Glaucoma;Diabetic complication;Scar tissue;Inflammation;Urinary tract disease;Pulmonary fibrosis;Diabetic nephropathy;Fibrosis;Solid tumor;Angiogenesis disorder;Glioma;Cancer;Atherosclerosis;Glomerular disease;Lung tumor;Nephritis;Immune disorder;Bone marrow transplantation |  |
| 2rl5_v | 5 | 3.607 | 0.7213 | 2.04842 | 4 | 1 | 0 | 0 | 0 | 0 | Vascular endothelial growth factor receptor 2 | NONE | P35968 | Tyrosine-protein kinase that acts as a cell-surface receptor for VEGFA, VEGFC and VEGFD. Plays an essential role in the regulation of angiogenesis, vascular development, vascular permeability, and embryonic hematopoiesis. Promotes proliferation, survival, migration and differentiation of endothelial cells. Promotes reorganization of the actin cytoskeleton. Isoforms lacking a transmembrane domain, such as isoform 2 and isoform 3, may function as decoy receptors for VEGFA, VEGFC and/or VEGFD. Isoform 2 plays an important role as negative regulator of VEGFA- and VEGFC-mediated lymphangiogenesis by limiting the amount of free VEGFA and/or VEGFC and preventing their binding to FLT4. Modulates FLT1 and FLT4 signaling by forming heterodimers. Binding of vascular growth factors to isoform 1 leads to the activation of several signaling cascades. Activation of PLCG1 leads to the production of the cellular signaling molecules diacylglycerol and inositol 1,4,5-trisphosphate and the activation of protein kinase C. Mediates activation of MAPK1/ERK2, MAPK3/ERK1 and the MAP kinase signaling pathway, as well as of the AKT1 signaling pathway. Mediates phosphorylation of PIK3R1, the regulatory subunit of phosphatidylinositol 3-kinase, reorganization of the actin cytoskeleton and activation of PTK2/FAK1. Required for VEGFA-mediated induction of NOS2 and NOS3, leading to the production of the signaling molecule nitric oxide (NO) by endothelial cells. Phosphorylates PLCG1. Promotes phosphorylation of FYN, NCK1, NOS3, PIK3R1, PTK2/FAK1 and SRC. | NONE | NONE |  |
| 1gyk_v | 4 | 2.885 | 0.7213 | -0.324732 | 0 | 3 | 0 | 0 | 1 | 0 | Serum amyloid P-component | NONE | P02743 | Involved in unfolded protein binding | NONE | NONE |  |
| 1s95_v | 4 | 2.883 | 0.7207 | 0.25002 | 3 | 0 | 1 | 0 | 0 | 0 | Serine/threonine-protein phosphatase 5 | NONE | P53041 | Involved in hydrolase activity | NONE | NONE |  |
| 2fb8_v | 4 | 2.871 | 0.7178 | 0.199622 | 3 | 1 | 0 | 0 | 0 | 0 | B-Raf proto-oncogene serine/threonine-protein kinase | TRANSFERASE | BRAF1_HUMAN | Involved in the transduction of mitogenic signals from the cell membrane to the nucleus. May play a role in the postsynaptic responses of hippocampal neuron. | Defects in BRAF are a cause of cardiofaciocutaneous syndrome (CFC syndrome) [MIM:115150]; also known as cardio-facio- cutaneous syndrome. CFC syndrome is characterized by a distinctive facial appearance, heart defects and mental retardation. Heart defects include pulmonic stenosis, atrial septal defects and hypertrophic cardiomyopathy. Some affected individuals present with ectodermal abnormalities such as sparse, friable hair, hyperkeratotic skin lesions and a generalized ichthyosis-like condition. Typical facial features are similar to Noonan syndrome. They include high forehead with bitemporal constriction, hypoplastic supraorbital ridges, downslanting palpebral fissures, a depressed nasal bridge, and posteriorly angulated ears with prominent helices. The inheritance of CFC syndrome is autosomal dominant.;Defects in BRAF are involved in a wide range of cancers.;Defects in BRAF are involved in lung cancer [MIM:211980].;Defects in BRAF are involved in non-Hodgkin lymphoma (NHL) [MIM:605027]. NHL is a cancer that starts in cells of the lymph system, which is part of the bodys immune system. NHLs can occur at any age and are often marked by enlarged lymph nodes, fever and weight loss.;Defects in BRAF may be a cause of colorectal cancer (CRC) [MIM:114500]. | Melanoma;Cancer |  |
| 2g1y_v | 4 | 2.87 | 0.7175 | 0.514085 | 2 | 1 | 1 | 0 | 0 | 0 | Renin | HYDROLASE | RENI_HUMAN | Renin is a highly specific endopeptidase, whose only known function is to generate angiotensin I from angiotensinogen in the plasma, initiating a cascade of reactions that produce an elevation of blood pressure and increased sodium retention by the kidney | Defects in REN are a cause of renal tubular dysgenesis (RTD) [MIM:267430]. RTD is an autosomal recessive severe disorder of renal tubular development characterized by persistent fetal anuria and perinatal death, probably due to pulmonary hypoplasia from early-onset oligohydramnios (the Potter phenotype). | Glaucoma;Renal disease;Cardiac failure;Atherosclerosis;HIV infection;Hypertension;Cardiovascular disease;Ocular hypertension;Renal failure |  |
| 5gal_v | 4 | 2.87 | 0.7175 | 0.401058 | 0 | 4 | 0 | 0 | 0 | 0 | Galectin-7 | LECTIN | LEG7_HUMAN | Could be involved in cell-cell and/or cell-matrix interactions necessary for normal growth control. Pro-apoptotic protein that functions intracellularly upstream of JNK activation and cytochrome c release. | NONE | NONE |  |
| 1k59_v | 4 | 2.861 | 0.7153 | -0.0190767 | 0 | 4 | 0 | 0 | 0 | 0 | Angiogenin | HYDROLASE | ANGI_HUMAN | May function as a tRNA-specific ribonuclease that binds to actin on the surface of endothelial cells; once bound, angiogenin is endocytosed and translocated to the nucleus, thereby promoting the endothelial invasiveness necessary for blood vessel formatio | Defects in ANG are the cause of susceptibility to amyotrophic lateral sclerosis type 9 (ALS9) [MIM:611895]. ALS is a degenerative disorder of motor neurons in the cortex, brain stem and spinal cord. ALS is characterized by muscular weakness and atrophy. | NONE |  |
| 1ya4_v | 4 | 2.86 | 0.715 | -0.0042763 | 4 | 0 | 0 | 0 | 0 | 0 | Liver carboxylesterase 1 | HYDROLASE | EST1_HUMAN | Involved in the detoxification of xenobiotics and in the activation of ester and amide prodrugs. Hydrolyzes aromatic and aliphatic esters, but has no catalytic activity toward amides or a fatty acyl CoA ester. | NONE | Arteriosclerosis;Cerebrovascular disease;Cancer;Melanoma;Multiple myeloma;Hypercholesterolemia;Atherosclerosis;Breast tumor;Hyperlipidemia;Solid tumor;Cardiovascular disease;Prostate tumor;Alzheimers disease;Dementia;Osteoporosis |  |
| 1fdu_v | 4 | 2.859 | 0.7146 | 0.15258 | 3 | 0 | 1 | 0 | 0 | 0 | Estradiol 17-beta-dehydrogenase 1 | NONE | P14061 | Lipid transport and metabolism | NONE | Some evidence suggests that NADH might be useful in treating Parkinsons disease, chronic fatigue syndrome, Alzheimers disease and cardiovascular disease. |  |
| 1kbo_v | 4 | 2.858 | 0.7144 | 0.260635 | 3 | 0 | 1 | 0 | 0 | 0 | NAD(P)H dehydrogenase [quinone] 1 | OXIDOREDUCTASE | NQO1_HUMAN | The enzyme apparently serves as a quinone reductase in connection with conjugation reactions of hydroquinons involved in detoxification pathways as well as in biosynthetic processes such as the vitamin K-dependent gamma-carboxylation of glutamate residues | NONE | NONE |  |
| 1qkt_v | 4 | 2.853 | 0.7133 | -0.232563 | 3 | 1 | 0 | 0 | 0 | 0 | Estrogen receptor | NUCLEAR RECEPTOR | ESR1_HUMAN | Nuclear hormone receptor. The steroid hormones and their receptors are involved in the regulation of eukaryotic gene expression and affect cellular proliferation and differentiation in target tissues. | NONE | Melanoma;Brain tumor;Inflammation;Myocardial infarction;Fibrosis;Brain ischemia;Psoriasis;Rheumatoid arthritis;Retinitis pigmentosa;Atherosclerosis;Genitourinary disease;Breast disease;Atrophy;Crohns disease;Testosterone deficiency;Uterine fibroids;Fibrocystic breast disease;Arthralgia;Prostatitis;Prostate hyperplasia;Inflammatory bowel disease;Solid tumor;Gynecological disorder;Vascular disease;Contraception;Cancer;Sepsis;Hypercholesterolemia;Neurological disease;Ataxia;Cognitive disorder;Osteoporosis;Gynecomastia;Major depressive disorder;Acne;Female contraception;Dysmenorrhea;Neoplasm;Central nervous system disease;Multiple sclerosis;Alcoholism;Lacrimal gland disease;Carcinoma;Cardiovascular disease;Menopause;Unidentified indication;Alzheimers disease;Breast tumor;Scar tissue;Hyperlipidemia;Postmenopausal osteoporosis;Papillomavirus infection;Ovary tumor;Prostate tumor;Adrenal disease;Urinary dysfunction;Estrogen deficiency;Endometriosis;Hormone deficiency;Musculoskeletal disease;Glioma;Vagina disease;Non-small-cell lung cancer;Parkinsons disease;Uterus tumor;Psychiatric disorder |  |
| 1lhw_v | 4 | 2.847 | 0.7117 | 0.102287 | 2 | 2 | 0 | 0 | 0 | 0 | Sex hormone-binding globulin | TRANSPORT PROTEIN | SHBG_HUMAN | Functions as an androgen transport protein, but may also be involved in receptor mediated processes. Each dimer binds one molecule of steroid. Specific for 5-alpha-dihydrotestosterone, testosterone, and 17-beta-estradiol. Regulates the plasma metabolic cl | NONE | NONE |  |
| 2f6y_v | 4 | 2.828 | 0.7071 | 0.235997 | 2 | 1 | 0 | 0 | 1 | 0 | Tyrosine-protein phosphatase non-receptor type 1 | HYDROLASE | PTN1_HUMAN | May play an important role in CKII- and p60c-src-induced signal transduction cascades (By similarity). | NONE | NONE |  |
| 2acl_v | 4 | 2.825 | 0.7062 | 0.176154 | 3 | 1 | 0 | 0 | 0 | 0 | Retinoic acid receptor RXR-alpha | TRANSCRIPTION | RXRA_HUMAN | Nuclear hormone receptor. Involved in the retinoic acid response pathway. Binds 9-cis retinoic acid (9C-RA). ARF6 acts as a key regulator of the tissue-specific adipocyte P2 (aP2) enhancer (By similarity). | NONE | Acute promyelocytic leukemia |  |
| 1xm4_v | 4 | 2.823 | 0.7058 | 0.500021 | 2 | 1 | 0 | 0 | 0 | 1 | cAMP-specific 3,5-cyclic phosphodiesterase 4B | HYDROLASE | PDE4B_HUMAN | May be involved in mediating central nervous system effects of therapeutic agents ranging from antidepressants to antiasthmatic and anti-inflammatory agents. | NONE | Chronic obstructive pulmonary disease;Asthma |  |
| 1rkp_v | 4 | 2.822 | 0.7054 | 0.375594 | 1 | 2 | 0 | 0 | 0 | 1 | cGMP-specific 3,5-cyclic phosphodiesterase | HYDROLASE | PDE5A_HUMAN | Plays a role in signal transduction by regulating the intracellular concentration of cyclic nucleotides. This phosphodiesterase catalyzes the specific hydrolysis of cGMP to 5- GMP. | NONE | Anal fissure;Raynauds disease;Cardiac failure;Thrombosis;Diabetic complication;Prostate hyperplasia;Erectile dysfunction;Urinary tract disease;Female sexual dysfunction;Vascular disease;Viral infection;Cancer;Atherosclerosis;Pulmonary disease;Metabolic disorder;Hypertension;Cardiovascular disease;Pulmonary hypertension;Angina;Male sexual dysfunction |  |
| 1kta_v | 4 | 2.808 | 0.702 | -0.368999 | 1 | 2 | 0 | 0 | 1 | 0 | Branched-chain-amino-acid aminotransferase, mitochondrial | NONE | O15382 | Amino acid transport and metabolism | NONE | For nutritional supplementation and for treating dietary shortage or imbalance. |  |
| 2jdt_v | 4 | 2.8 | 0.7 | 0.281661 | 3 | 0 | 0 | 1 | 0 | 0 | cAMP-dependent protein kinase catalytic subunit alpha | NONE | P00517 | Involved in protein serine/threonine kinase activity | NONE | NONE |  |
| 1ih0_v | 4 | 2.794 | 0.6986 | 0.0554021 | 3 | 0 | 1 | 0 | 0 | 0 | Troponin C, slow skeletal and cardiac muscles | NONE | P63316 | Involved in calcium ion binding | NONE | For short term treatment of acutely decompensated severe chronic heart failure (CHF). Also being investigated for use/treatment in heart disease. |  |
| 1ov4_v | 5 | 3.48 | 0.6959 | 0.863803 | 4 | 1 | 0 | 0 | 0 | 0 | Bile salt sulfotransferase | NONE | Q06520 | Involved in sulfotransferase activity | NONE | NONE |  |
| 1yw9_v | 4 | 2.784 | 0.6959 | 0.0817941 | 3 | 0 | 0 | 0 | 1 | 0 | Methionine aminopeptidase 2 | HYDROLASE | AMPM2_HUMAN | Removes the amino-terminal methionine from nascent proteins. | NONE | NONE |  |
| 2zas_v | 4 | 2.783 | 0.6957 | -0.11929 | 3 | 1 | 0 | 0 | 0 | 0 | Estrogen-related receptor gamma | NONE | P62508 | Orphan receptor that acts as transcription activator in the absence of bound ligand. Binds specifically to an estrogen response element and activates reporter genes controlled by estrogen response elements (By similarity). Induces the expression of PERM1 in the skeletal muscle. | NONE | NONE |  |
| 1m9j_v | 4 | 2.779 | 0.6946 | -0.0609978 | 2 | 1 | 1 | 0 | 0 | 0 | Nitric oxide synthase, endothelial | OXIDOREDUCTASE | NOS3_HUMAN | Produces nitric oxide (NO) which is implicated in vascular smooth muscle relaxation through a cGMP-mediated signal transduction pathway. NO mediates vascular endothelial growth factor (VEGF)-induced angiogenesis in coronary vessels and promotes blood clot | NONE | Coronary artery disease;Hypertension;Congestive heart failure;Angina;Myocardial infarction;Peripheral vascular disease;Atherosclerosis;Ischemic heart disease |  |
| 2of0_v | 4 | 2.75 | 0.6874 | -0.0203525 | 3 | 0 | 0 | 1 | 0 | 0 | Beta-secretase 1 | HYDROLASE | BACE1_HUMAN | Responsible for the proteolytic processing of the amyloid precursor protein (APP). Cleaves at the N-terminus of the A-beta peptide sequence, between residues 671 and 672 of APP, leads to the generation and extracellular release of beta-cleaved soluble APP | NONE | Alzheimers disease |  |
| 1d7i_v | 3 | 2.058 | 0.686 | -1.15913 | 1 | 2 | 0 | 0 | 0 | 0 | Peptidyl-prolyl cis-trans isomerase FKBP1A | ISOMERASE | FKB1A_HUMAN | May play a role in modulation of ryanodine receptor isoform-1 (RYR-1), a component of the calcium release channel of skeletal muscle sarcoplasmic reticulum. There are four molecules of FKBP12 per skeletal muscle RYR. PPIases accelerate the folding of prot | NONE | NONE |  |
| 3f5p_v | 4 | 2.727 | 0.6817 | -0.0724841 | 3 | 1 | 0 | 0 | 0 | 0 | Insulin-like growth factor 1 receptor | NONE | P08069 | Involved in transmembrane receptor protein tyrosine kinase activity | NONE | For treatment of diabetes (type I and II) |  |
| 2pe0_v | 4 | 2.716 | 0.679 | -0.142297 | 3 | 1 | 0 | 0 | 0 | 0 | 3-phosphoinositide-dependent protein kinase 1 | TRANSFERASE | PDPK1_HUMAN | Phosphorylates and activates not only PKB/AKT, but also PKA, PKC-zeta, RPS6KA1 and RPS6KB1. May play a general role in signaling processes and in development (By similarity). Isoform 3 is catalytically inactive. | NONE | NONE |  |
| 1ya3_v | 4 | 2.711 | 0.6778 | -0.152217 | 3 | 1 | 0 | 0 | 0 | 0 | Mineralocorticoid receptor | TRANSCRIPTION | MCR_HUMAN | Receptor for both mineralocorticoids (MC) such as aldosterone and glucocorticoids (GC) such as corticosterone or cortisol. Binds to mineralocorticoid response elements (MRE) and transactivates target genes. The effect of MC is to increase ion and water tr | Defects in NR3C2 are a cause of autosomal dominant pseudohypoaldosteronism type I (PHA1) [MIM:177735]. PHA1 is characterized by urinary salt wasting, resulting from target organ unresponsiveness to mineralocorticoids. There are 2 forms of PHA1: the autosomal dominant form that is mild, and the recessive form which is more severe and due to defects in any of the epithelial sodium channel subunits. In autosomal dominant PHA1 the target organ defect is confined to kidney. Clinical expression can vary from asymptomatic to moderate. It may be severe at birth, but symptoms remit with age. Familial and sporadic cases have been reported.;Defects in NR3C2 are a cause of early onset hypertension with severe exacerbation in pregnancy [MIM:605115]. Inheritance is autosomal dominant. The disease is characterized by the onset of severe hypertension before the age of 20, and by suppression of aldosterone secretion. | Metabolic disorder;Hypertension;Cardiovascular disease;Urinary tract disease |  |
| 1zxc_v | 4 | 2.699 | 0.6747 | -0.403702 | 3 | 1 | 0 | 0 | 0 | 0 | ADAM 17 | HYDROLASE | ADA17_HUMAN | Cleaves the membrane-bound precursor of TNF-alpha to its mature soluble form. Responsible for the proteolytic release of several other cell-surface proteins, including p75 TNF-receptor, interleukin 1 receptor type II, p55 TNF-receptor, transforming growth | NONE | Psoriasis;Inflammation;Rheumatoid arthritis;Cardiac failure;Multiple sclerosis;Metastasis;Immune disorder |  |
| 1y2c_v | 5 | 3.368 | 0.6736 | 1.35078 | 4 | 1 | 0 | 0 | 0 | 0 | cAMP-specific 3,5-cyclic phosphodiesterase 4D | HYDROLASE | PDE4D_HUMAN | Regulates the levels of cAMP in the cell. | Genetic variations in PDE4D might be associated with susceptibility to stroke type 1 (STRK1) [MIM:606799]. A stroke is an acute neurologic event leading to death of neural tissue of the brain and resulting in loss of motor, sensory and/or cognitive function. PubMed:17006457 states that association with stroke has to be considered with caution. | NONE |  |
| 2q6c_v | 5 | 3.356 | 0.6713 | 1.72748 | 2 | 2 | 0 | 0 | 1 | 0 | 3-hydroxy-3-methylglutaryl-coenzyme A reductase | OXIDOREDUCTASE | HMDH_HUMAN | This transmembrane glycoprotein is involved in the control of cholesterol biosynthesis. It is the rate-limiting enzyme of sterol biosynthesis. | NONE | Alzheimers disease;Renal disease;Lipid metabolism disorder;Cardiac failure;Hyperlipidemia;Intermittent claudication;Myocardial infarction;Coronary artery disease;Glioma;Hypercholesterolemia;Atherosclerosis;Peripheral arterial occlusive disease;Cardiovascular disease;Congestive heart failure;Angina;Hypertension;Heart disease;Cerebrovascular ischemia;Osteoporosis |  |
| 1n83_v | 4 | 2.669 | 0.6672 | -0.707812 | 4 | 0 | 0 | 0 | 0 | 0 | Nuclear receptor ROR-alpha | LIPID BINDING PROTEIN | RORA_HUMAN | Orphan nuclear receptor. Binds DNA as a monomer to hormone response elements (HRE) containing a single core motif half-site preceded by a short A-T-rich sequence. This isomer binds to the consensus sequence 5-[AT][TA]A[AT][CGT]TAGGTCA-3. | NONE | NONE |  |
| 2w1f_v | 4 | 2.667 | 0.6668 | 0.0509406 | 2 | 1 | 1 | 0 | 0 | 0 | Serine/threonine-protein kinase 6 | NONE | O14965 | Involved in protein kinase activity | NONE | NONE |  |
| 1dmw_v | 5 | 3.297 | 0.6594 | 1.25382 | 1 | 3 | 1 | 0 | 0 | 0 | Phenylalanine-4-hydroxylase | OXIDOREDUCTASE | PH4H_HUMAN | NONE | Defects in PAH are the cause of phenylketonuria (PKU) [MIM:261600]. PKU is an autosomal recessive inborn error of phenylalanine metabolism, due to severe phenylalanine hydroxylase deficiency. It is characterized by blood concentrations of phenylalanine persistently above 1200 mumol (normal concentration 100 mumol) which usually causes mental retardation (unless low phenylalanine diet is introduced early in life). They tend to have light pigmentation, rashes similar to eczema, epilepsy, extreme hyperactivity, psychotic states and an unpleasant mousy odor.;Defects in PAH are the cause of non-phenylketonuria hyperphenylalaninemia (Non-PKU HPA) [MIM:261600]. Non-PKU HPA is a mild form of phenylalanine hydroxylase deficiency characterized by phenylalanine levels persistently below 600 mumol, which allows normal intellectual and behavioral development without treatment. Non-PKU HPA is usually caused by the combined effect of a mild hyperphenylalaninemia mutation and a severe one.;Defects in PAH are the cause of hyperphenylalaninemia (HPA) [MIM:261600]. HPA is the mildest form of phenylalanine hydroxylase deficiency. | Phenylketonuria;Sickle cell anemia;Vascular disease;Genetic disorder;Diabetic nephropathy;Hypertension;Ataxia;Peripheral arterial occlusive disease;Coronary artery disease |  |
| 3fzk_v | 4 | 2.635 | 0.6588 | -0.229989 | 2 | 2 | 0 | 0 | 0 | 0 | Heat shock cognate 71 kDa protein | NONE | P11142 | Molecular chaperone implicated in a wide variety of cellular processes, including protection of the proteome from stress, folding and transport of newly synthesized polypeptides, activation of proteolysis of misfolded proteins and the formation and dissociation of protein complexes. Plays a pivotal role in the protein quality control system, ensuring the correct folding of proteins, the re-folding of misfolded proteins and controlling the targeting of proteins for subsequent degradation (PubMed:21150129, PubMed:21148293, PubMed:24732912, PubMed:27916661, PubMed:23018488). This is achieved through cycles of ATP binding, ATP hydrolysis and ADP release, mediated by co-chaperones (PubMed:21150129, PubMed:21148293, PubMed:24732912, PubMed:27916661, PubMed:23018488). The co-chaperones have been shown to not only regulate different steps of the ATPase cycle of HSP70, but they also have an individual specificity such that one co-chaperone may promote folding of a substrate while another may promote degradation (PubMed:21150129, PubMed:21148293, PubMed:24732912, PubMed:27916661, PubMed:23018488). The affinity of HSP70 for polypeptides is regulated by its nucleotide bound state. In the ATP-bound form, it has a low affinity for substrate proteins. However, upon hydrolysis of the ATP to ADP, it undergoes a conformational change that increases its affinity for substrate proteins. HSP70 goes through repeated cycles of ATP hydrolysis and nucleotide exchange, which permits cycles of substrate binding and release. The HSP70-associated co-chaperones are of three types: J-domain co-chaperones HSP40s (stimulate ATPase hydrolysis by HSP70), the nucleotide exchange factors (NEF) such as BAG1/2/3 (facilitate conversion of HSP70 from the ADP-bound to the ATP-bound state thereby promoting substrate release), and the TPR domain chaperones such as HOPX and STUB1 (PubMed:24318877, PubMed:27474739, PubMed:24121476, PubMed:26865365). Acts as a repressor of transcriptional activation. Inhibits the transcriptional coactivator activity of CITED1 on Smad-mediated transcription. Component of the PRP19-CDC5L complex that forms an integral part of the spliceosome and is required for activating pre-mRNA splicing. May have a scaffolding role in the spliceosome assembly as it contacts all other components of the core complex. Binds bacterial lipopolysaccharide (LPS) and mediates LPS-induced inflammatory response, including TNF secretion by monocytes (PubMed:10722728, PubMed:11276205). Participates in the ER-associated degradation (ERAD) quality control pathway in conjunction with J domain-containing co-chaperones and the E3 ligase STUB1 (PubMed:23990462). | NONE | NONE |  |
| 1q6k_v | 4 | 2.632 | 0.6581 | -0.101559 | 1 | 2 | 1 | 0 | 0 | 0 | Cathepsin K | HYDROLASE | CATK_HUMAN | Closely involved in osteoclastic bone resorption and may participate partially in the disorder of bone remodeling. Displays potent endoprotease activity against fibrinogen at acid pH. May play an important role in extracellular matrix degradation. | Defects in CTSK are the cause of pycnodysostosis (PKND) [MIM:265800]. PKND is an autosomal recessive osteochondrodysplasia characterized by osteosclerosis and short stature. | Multiple sclerosis;Asthma;Cancer;Rheumatoid arthritis;Autoimmune disease;Arthritis;Bacterial infection;Bone disease;Osteoarthritis;Bone metastases;Immune disorder;Osteoporosis |  |
| 2of2_v | 4 | 2.595 | 0.6488 | -0.378562 | 3 | 0 | 0 | 1 | 0 | 0 | Proto-oncogene tyrosine-protein kinase LCK | TRANSFERASE | LCK_HUMAN | Tyrosine kinase that plays an essential role for the selection and maturation of developing T-cell in the thymus and in mature T-cell function. Is constitutively associated with the cytoplasmic portions of the CD4 and CD8 surface receptors and plays a key | A chromosomal aberration involving LCK is found in leukemias. Translocation t(1;7)(p34;q34) with TCRB. | Psoriasis;Cancer;Rheumatoid arthritis;Cardiac failure;Atherosclerosis;Delayed hypersensitivity;Transplant rejection;Inflammation;Multiple sclerosis;Autoimmune disease |  |
| 3efj_v | 5 | 3.236 | 0.6473 | 1.39402 | 3 | 1 | 0 | 0 | 0 | 1 | Hepatocyte growth factor receptor | NONE | P08581 | Involved in hepatocyte growth factor activity | NONE | NONE |  |
| 2rku_v | 4 | 2.563 | 0.6407 | -0.372036 | 2 | 1 | 1 | 0 | 0 | 0 | Serine/threonine-protein kinase PLK1 | NONE | P53350 | Serine/threonine-protein kinase that performs several important functions throughout M phase of the cell cycle, including the regulation of centrosome maturation and spindle assembly, the removal of cohesins from chromosome arms, the inactivation of anaphase-promoting complex/cyclosome (APC/C) inhibitors, and the regulation of mitotic exit and cytokinesis. Polo-like kinase proteins acts by binding and phosphorylating proteins are that already phosphorylated on a specific motif recognized by the POLO box domains. Phosphorylates BORA, BUB1B/BUBR1, CCNB1, CDC25C, CEP55, ECT2, ERCC6L, FBXO5/EMI1, FOXM1, KIF20A/MKLP2, CENPU, NEDD1, NINL, NPM1, NUDC, PKMYT1/MYT1, KIZ, PPP1R12A/MYPT1, PRC1, RACGAP1/CYK4, SGO1, STAG2/SA2, TEX14, TOPORS, p73/TP73, TPT1 and WEE1. Plays a key role in centrosome functions and the assembly of bipolar spindles by phosphorylating KIZ, NEDD1 and NINL. NEDD1 phosphorylation promotes subsequent targeting of the gamma-tubulin ring complex (gTuRC) to the centrosome, an important step for spindle formation. Phosphorylation of NINL component of the centrosome leads to NINL dissociation from other centrosomal proteins. Involved in mitosis exit and cytokinesis by phosphorylating CEP55, ECT2, KIF20A/MKLP2, CENPU, PRC1 and RACGAP1. Recruited at the central spindle by phosphorylating and docking PRC1 and KIF20A/MKLP2; creates its own docking sites on PRC1 and KIF20A/MKLP2 by mediating phosphorylation of sites subsequently recognized by the POLO box domains. Phosphorylates RACGAP1, thereby creating a docking site for the Rho GTP exchange factor ECT2 that is essential for the cleavage furrow formation. Promotes the central spindle recruitment of ECT2. Plays a central role in G2/M transition of mitotic cell cycle by phosphorylating CCNB1, CDC25C, FOXM1, CENPU, PKMYT1/MYT1, PPP1R12A/MYPT1 and WEE1. Part of a regulatory circuit that promotes the activation of CDK1 by phosphorylating the positive regulator CDC25C and inhibiting the negative regulators WEE1 and PKMYT1/MYT1. Also acts by mediating phosphorylation of cyclin-B1 (CCNB1) on centrosomes in prophase. Phosphorylates FOXM1, a key mitotic transcription regulator, leading to enhance FOXM1 transcriptional activity. Involved in kinetochore functions and sister chromatid cohesion by phosphorylating BUB1B/BUBR1, FBXO5/EMI1 and STAG2/SA2. PLK1 is high on non-attached kinetochores suggesting a role of PLK1 in kinetochore attachment or in spindle assembly checkpoint (SAC) regulation. Required for kinetochore localization of BUB1B. Regulates the dissociation of cohesin from chromosomes by phosphorylating cohesin subunits such as STAG2/SA2. Phosphorylates SGO1: required for spindle pole localization of isoform 3 of SGO1 and plays a role in regulating its centriole cohesion function. Mediates phosphorylation of FBXO5/EMI1, a negative regulator of the APC/C complex during prophase, leading to FBXO5/EMI1 ubiquitination and degradation by the proteasome. Acts as a negative regulator of p53 family members: phosphorylates TOPORS, leading to inhibit the sumoylation of p53/TP53 and simultaneously enhance the ubiquitination and subsequent degradation of p53/TP53. Phosphorylates the transactivation domain of the transcription factor p73/TP73, leading to inhibit p73/TP73-mediated transcriptional activation and pro-apoptotic functions. Phosphorylates BORA, and thereby promotes the degradation of BORA. Contributes to the regulation of AURKA function. Also required for recovery after DNA damage checkpoint and entry into mitosis. Phosphorylates MISP, leading to stabilization of cortical and astral microtubule attachments required for proper spindle positioning (PubMed:8991084, PubMed:11202906, PubMed:12207013, PubMed:12447691, PubMed:12524548, PubMed:12738781, PubMed:12852856, PubMed:12939256, PubMed:14532005, PubMed:14734534, PubMed:15070733, PubMed:15148369, PubMed:15469984, PubMed:16198290, PubMed:16247472, PubMed:16980960, PubMed:17081991, PubMed:17351640, PubMed:17376779, PubMed:17617734, PubMed:18174154, PubMed:18331714, PubMed:18418051, PubMed:18477460, PubMed:18521620, PubMed:18615013, PubMed:19160488, PubMed:19351716, PubMed:19468300, PubMed:19468302, PubMed:19473992, PubMed:19509060, PubMed:19597481, PubMed:23455478, PubMed:23509069). Together with MEIKIN, acts as a regulator of kinetochore function during meiosis I: required both for mono-orientation of kinetochores on sister chromosomes and protection of centromeric cohesin from separase-mediated cleavage (By similarity). Phosphorylates CEP68 and is required for its degradation (PubMed:25503564). Regulates nuclear envelope breakdown during prophase by phosphorylating DCTN1 resulting in its localization in the nuclear envelope (PubMed:20679239). Phosphorylates the heat shock transcription factor HSF1, promoting HSF1 nuclear translocation upon heat shock (PubMed:15661742). Phosphorylates HSF1 also in the early mitotic period; this phosphorylation regulates HSF1 localization to the spindle pole, the recruitment of the SCF(BTRC) ubiquitin ligase complex induicing HSF1 degradation, and hence mitotic progression (PubMed:18794143). | NONE | NONE |  |
| 1t67_v | 6 | 3.816 | 0.636 | 2.25095 | 3 | 1 | 2 | 0 | 0 | 0 | Histone deacetylase 8 | HYDROLASE | HDAC8_HUMAN | Responsible for the deacetylation of lysine residues on the N-terminal part of the core histones (H2A, H2B, H3 and H4). Histone deacetylation gives a tag for epigenetic repression and plays an important role in transcriptional regulation, cell cycle progr | NONE | NONE |  |
| 3f7z_v | 5 | 3.178 | 0.6356 | 0.933183 | 3 | 2 | 0 | 0 | 0 | 0 | Glycogen synthase kinase-3 beta | NONE | P49841 | Involved in protein kinase activity | NONE | NONE |  |
| 1juj_v | 4 | 2.529 | 0.6323 | -1.34219 | 2 | 1 | 0 | 0 | 1 | 0 | Thymidylate synthase | TRANSFERASE | TYSY_HUMAN | NONE | NONE | Small-cell lung cancer;Solid tumor;Breast tumor;Head and neck tumor;Brain tumor;Neoplasm;Hepatocellular carcinoma;Ovary tumor;Prostate tumor;Pancreas tumor;Urinary tract tumor;Stomach tumor;Retinopathy;Bladder tumor;Colon tumor;Cancer;Mesothelioma;Non-small-cell lung cancer;Cholangiocarcinoma;Gastrointestinal tumor;Liver tumor;Lung tumor;Colorectal tumor;Uterine cervix tumor;Carcinoma;Biliary tumor;Gallbladder disease;Keratosis |  |
| 1vj5_v | 5 | 3.16 | 0.632 | 0.704096 | 3 | 1 | 1 | 0 | 0 | 0 | Epoxide hydrolase 2 | HYDROLASE | HYES_HUMAN | Acts on epoxides (alkene oxides, oxiranes) and arene oxides. Plays a role in xenobiotic metabolism by degrading potentially toxic epoxides. Also determines steady-state levels of physiological mediators. Has low phosphatase activity. | NONE | NONE |  |
| 1ctr_v | 5 | 3.121 | 0.6242 | 0.276611 | 5 | 0 | 0 | 0 | 0 | 0 | Calmodulin | CALCIUM-BINDING PROTEIN | CALM_HUMAN | Calmodulin mediates the control of a large number of enzymes and other proteins by Ca(2+). Among the enzymes to be stimulated by the calmodulin-Ca(2+) complex are a number of protein kinases and phosphatases. Together with CEP110 and centrin, is involved | NONE | NONE |  |
| 1he2_v | 6 | 3.726 | 0.621 | 2.60061 | 4 | 1 | 0 | 0 | 1 | 0 | Flavin reductase | NONE | P30043 | Cell wall/membrane/envelope biogenesis | NONE | For the treatment of ariboflavinosis (vitamin B2 deficiency). |  |
| 1m51_v | 6 | 3.687 | 0.6145 | 0.860745 | 3 | 3 | 0 | 0 | 0 | 0 | Phosphoenolpyruvate carboxykinase, cytosolic [GTP] | NONE | P35558 | Energy production and conversion | NONE | NONE |  |
| 1agw_v | 4 | 2.457 | 0.6143 | -0.738066 | 2 | 1 | 1 | 0 | 0 | 0 | Basic fibroblast growth factor receptor 1 | PROTEIN KINASE | FGFR1_HUMAN | Receptor for basic fibroblast growth factor. A shorter form of the receptor could be a receptor for FGF1 (aFGF). | Defects in FGFR1 are a cause of Pfeiffer syndrome (PS) [MIM:101600]; also known as acrocephalosyndactyly type V (ACS5). PS is characterized by craniosynostosis (premature fusion of the skull sutures) with deviation and enlargement of the thumbs and great toes, brachymesophalangy, with phalangeal ankylosis and a varying degree of soft tissue syndactyly.;Defects in FGFR1 are a cause of isolated hypogonadotropic hypogonadism (IHH) [MIM:146110]. Hypogonadism is a condition characterized by abnormally decreased gonadal function, with retardation of growth and sexual development. Hypogonadotropic hypogonadism is due to inadequate secretion of gonadotropins. It results from failure to release sufficient gonadotropin-releasing hormone.;Defects in FGFR1 are the cause of Kallmann syndrome type 2 (KAL2) [MIM:147950]; also known as hypogonadotropic hypogonadism and anosmia. Anosmia or hyposmia is related to the absence or hypoplasia of the olfactory bulbs and tracts. Hypogonadism is due to deficiency in gonadotropin-releasing hormone and probably results from a failure of embryonic migration of gonadotropin- releasing hormone-synthesizing neurons. In some cases, midline cranial anomalies (cleft lip/palate and imperfect fusion) are present and anosmia may be absent or inconspicuous.;Defects in FGFR1 are the cause of osteoglophonic dysplasia (OGD) [MIM:166250]; also known as osteoglophonic dwarfism. OGD is characterized by craniosynostosis, prominent supraorbital ridge, and depressed nasal bridge, as well as by rhizomelic dwarfism and nonossifying bone lesions. Inheritance is autosomal dominant.;Defects in FGFR1 are the cause of non-syndromic trigonocephaly [MIM:190440]; also known as metopic craniosynostosis. The term trigonocephaly describes the typical keel-shaped deformation of the forehead resulting from premature fusion of the frontal suture. Trigonocephaly may occur also as a part of a syndrome.;A chromosomal aberration involving FGFR1 may be a cause of stem cell leukemia lymphoma syndrome (SCLL). Translocation t(8;13)(p11;q12) with ZMYM2. SCLL usually presents as lymphoblastic lymphoma in association with a myeloproliferative disorder, often accompanied by pronounced peripheral eosinophilia and/or prominent eosinophilic infiltrates in the affected bone marrow.;A chromosomal aberration involving FGFR1 may be a cause of stem cell myeloproliferative disorder (MPD). Translocation t(6;8)(q27;p11) with FGFR1OP. Insertion ins(12;8)(p11;p11p22) with FGFR1OP2. MPD is characterized by myeloid hyperplasia, eosinophilia and T-cell or B-cell lymphoblastic lymphoma. In general it progresses to acute myeloid leukemia. The fusion proteins FGFR1OP2-FGFR1, FGFR1OP-FGFR1 or FGFR1-FGFR1OP may exhibit constitutive kinase activity and be responsible for the transforming activity.;A chromosomal aberration involving FGFR1 may be a cause of stem cell myeloproliferative disorder (MPD). Translocation t(8;9)(p12;q33) with CEP110. MPD is characterized by myeloid hyperplasia, eosinophilia and T-cell or B-cell lymphoblastic lymphoma. In general it progresses to acute myeloid leukemia. The fusion protein CEP110-FGFR1 is found in the cytoplasm, exhibits constitutive kinase activity and may be responsible for the transforming activity. | NONE |  |
| 1uhl_v | 6 | 3.682 | 0.6136 | 1.94967 | 5 | 1 | 0 | 0 | 0 | 0 | Oxysterols receptor LXR-alpha | DNA BINDING PROTEIN | NR1H3_HUMAN | Orphan receptor. Interaction with RXR shifts RXR from its role as a silent DNA-binding partner to an active ligand- binding subunit in mediating retinoid responses through target genes defined by LXRES. LXRES are DR4-type response elements characterized b | NONE | NONE |  |
| 2iit_v | 6 | 3.674 | 0.6123 | 1.70559 | 5 | 0 | 0 | 1 | 0 | 0 | Dipeptidyl peptidase 4 | HYDROLASE | DPP4_HUMAN | Removes N-terminal dipeptides sequentially from polypeptides having unsubstituted N-termini provided that the penultimate residue is proline. Plays a role in T-cell activation. | NONE | Rheumatoid arthritis;Diabetes mellitus;Non-insulin dependent diabetes;Autoimmune disease;Immune disorder |  |
| 1h1b_v | 6 | 3.67 | 0.6117 | 2.1414 | 3 | 3 | 0 | 0 | 0 | 0 | Leukocyte elastase | HYDROLASE(SERINE PROTEASE) | ELNE_HUMAN | Modifies the functions of natural killer cells, monocytes and granulocytes. Inhibits C5a-dependent neutrophil enzyme release and chemotaxis. | Defects in ELA2 are a cause of cyclic haematopoiesis (CH) [MIM:162800]; also known as cyclic neutropenia. CH is an autosomal dominant disease in which blood-cell production from the bone marrow oscillates with 21-day periodicity. Circulating neutrophils vary between almost normal numbers and zero. During intervals of neutropenia, affected individuals are at risk for opportunistic infection. Monocytes, platelets, lymphocytes and reticulocytes also cycle with the same frequency.;Defects in ELA2 are the cause of autosomal dominant severe congenital neutropenia type 1 (SCN1) [MIM:202700]. Severe congenital neutropenia is a heterogeneous disorder of hematopoiesis characterized by a maturation arrest of granulopoiesis at the level of promyelocytes with peripheral blood absolute neutrophil counts below 0.5 x 10(9)/l and early onset of severe bacterial infections. | Blood clotting disorder;Chronic bronchitis;Lung injury;Cerebrovascular ischemia;Emphysema;Respiratory disease;Cystic fibrosis;Chronic obstructive pulmonary disease;Myocardial infarction;Lung inflammation;Pulmonary fibrosis;Psoriasis;Pancreatitis;Bronchitis;Rheumatoid arthritis;Arthritis;Pulmonary disease;Lung tumor;Respiratory distress syndrome;Transplant rejection;Pulmonary hypertension;Pneumonia;Inflammation |  |
| 1e8z_v | 5 | 3 | 0.6 | 0.334623 | 3 | 1 | 0 | 1 | 0 | 0 | Phosphatidylinositol-4,5-bisphosphate 3-kinase catalytic subunit gamma isoform | PHOSPHOINOSITIDE 3-KINASE GAMMA | PK3CG_HUMAN | 3-phosphorylates the cellular phosphoinositide PtdIns- 4,5-biphosphate (PtdIns(4,5)P2) to produce PtdIns-3, 4,5- triiphosphate (PtdIns(3,4,5)P3). Links G-protein coupled receptor activation to the secondary messenger PtdIns(3,4,5)P3 production. | NONE | NONE |  |
| 1oec_v | 5 | 2.998 | 0.5996 | 0.199688 | 3 | 1 | 0 | 1 | 0 | 0 | Fibroblast growth factor receptor 2 | NONE | P21802 | Involved in protein kinase activity | NONE | For treatment of mucositis (mouth sores) |  |
| 1o6u_v | 5 | 2.996 | 0.5992 | -0.170598 | 5 | 0 | 0 | 0 | 0 | 0 | SEC14-like protein 2 | NONE | O76054 | Involved in transporter activity | NONE | Vitamin E, known for its antioxidant activities, is protective against cardiovascular disease and some forms of cancer and has also demonstrated immune-enhancing effects. It may be of limited benefit in some with asthma and rheumatoid arthritis. It may be helpful in some neurological diseases including Alzheimers, some eye disorders including cataracts, and diabetes and premenstrual syndrome. It may also help protect skin from ultraviolet irradiation although claims that it reverses skin aging, enhances male fertility and exercise performance are poorly supported. It may help relieve some muscle cramps. |  |
| 1osh_v | 5 | 2.995 | 0.599 | 0.129588 | 5 | 0 | 0 | 0 | 0 | 0 | Bile acid receptor | TRANSCRIPTION | NR1H4_HUMAN | Receptor for bile acids such as chenodeoxycholic acid, lithocholic acid and deoxycholic acid. Represses the transcription of the cholesterol 7-alpha-hydroxylase gene (CYP7A1) and activates the intestinal bile acid-binding protein (IBABP). Activates the tr | NONE | Liver fibrosis;Non-insulin dependent diabetes;Atherosclerosis;Hypertriglyceridemia;Hyperlipidemia;Liver disease;Primary biliary cirrhosis;Non-alcoholic steatohepatitis |  |
| 1og5_v | 6 | 3.592 | 0.5987 | 1.80513 | 3 | 3 | 0 | 0 | 0 | 0 | Cytochrome P450 2C9 | ELECTRON TRANSPORT | CP2C9_HUMAN | Cytochromes P450 are a group of heme-thiolate monooxygenases. In liver microsomes, this enzyme is involved in an NADPH-dependent electron transport pathway. It oxidizes a variety of structurally unrelated compounds, including steroids, fatty acids, and xe | NONE | NONE |  |
| 1oiz_v | 5 | 2.989 | 0.5979 | 0.109771 | 4 | 1 | 0 | 0 | 0 | 0 | Alpha-tocopherol transfer protein | NONE | P49638 | Involved in vitamin E transporter activity | NONE | Vitamin E, known for its antioxidant activities, is protective against cardiovascular disease and some forms of cancer and has also demonstrated immune-enhancing effects. It may be of limited benefit in some with asthma and rheumatoid arthritis. It may be helpful in some neurological diseases including Alzheimers, some eye disorders including cataracts, and diabetes and premenstrual syndrome. It may also help protect skin from ultraviolet irradiation although claims that it reverses skin aging, enhances male fertility and exercise performance are poorly supported. It may help relieve some muscle cramps. |  |
| 1u3w_v | 5 | 2.982 | 0.5965 | 0.285692 | 3 | 1 | 0 | 1 | 0 | 0 | Alcohol dehydrogenase 1C | NONE | P00326 | Energy production and conversion | NONE | Some evidence suggests that NADH might be useful in treating Parkinsons disease, chronic fatigue syndrome, Alzheimers disease and cardiovascular disease. |  |
| 1t84_v | 5 | 2.982 | 0.5963 | -0.209428 | 5 | 0 | 0 | 0 | 0 | 0 | Wiskott-Aldrich syndrome protein | SIGNALING PROTEIN | WASP_HUMAN | Effector protein for Rho-type GTPases, providing a link with the Arp2/3 complex that regulates the structure and dynamics of the actin cytoskeleton. Important for efficient actin polymerization. Possible regulator of lymphocyte and platelet function. | Defects in WAS are the cause of Wiskott-Aldrich syndrome (WAS) [MIM:301000]; also known as eczema-thrombocytopenia- immunodeficiency syndrome. WAS is an X-linked recessive immunodeficiency characterized by eczema, thrombocytopenia, recurrent infections, and bloody diarrhea. Death usually occurs before age 10.;Defects in WAS are the cause of thrombocytopenia type 1 (THC1) [MIM:313900]. Thrombocytopenia is defined by a decrease in the number of platelets in circulating blood, resulting in the potential for increased bleeding and decreased ability for clotting.;Defects in WAS are a cause of X-linked severe congenital neutropenia (XLN) [MIM:300299]. XLN is an X-linked immunodeficiency syndrome characterized by recurrent major bacterial infections, severe congenital neutropenia, and monocytopenia. | Wiskott-Aldrich syndrome |  |
| 1xbc_v | 5 | 2.97 | 0.5939 | 0.25938 | 3 | 1 | 1 | 0 | 0 | 0 | Tyrosine-protein kinase SYK | TRANSFERASE | KSYK_HUMAN | Positive effector of BCR-stimulated responses. Couples the B-cell antigen receptor (BCR) to the mobilization of calcium ion either through a phosphoinositide 3-kinase-dependent pathway, when not phosphorylated on tyrosines of the linker region, or through | NONE | Systemic lupus erythematosus;Asthma;Rheumatoid arthritis;Allergy;Allergic rhinitis;Inflammation;Thrombocytopenic purpura;Acute myelogenous leukemia;Lymphoma |  |
| 1soj_v | 4 | 2.374 | 0.5935 | -0.590542 | 1 | 2 | 0 | 0 | 0 | 1 | cGMP-inhibited 3,5-cyclic phosphodiesterase B | HYDROLASE | PDE3B_HUMAN | May play a role in fat metabolism. | NONE | NONE |  |
| 1wma_v | 5 | 2.964 | 0.5928 | 0.432933 | 2 | 2 | 1 | 0 | 0 | 0 | Carbonyl reductase [NADPH] 1 | OXIDOREDUCTASE | CBR1_HUMAN | Catalyzes the reduction of a wide variety of carbonyl compounds including the antitumor anthracycline antibiotics. Can convert prostaglandin E2 to prostaglandin F2-alpha. | NONE | NONE |  |
| 1pl6_v | 5 | 2.962 | 0.5925 | -0.114221 | 2 | 3 | 0 | 0 | 0 | 0 | Sorbitol dehydrogenase | OXIDOREDUCTASE | DHSO_HUMAN | NONE | NONE | Diabetic complication |  |
| 1gj8_v | 5 | 2.959 | 0.5917 | 0.717055 | 2 | 1 | 1 | 1 | 0 | 0 | Urokinase-type plasminogen activator | BLOOD CLOTTING, HYDROLASE | UROK_HUMAN | Specifically cleave the zymogen plasminogen to form the active enzyme plasmin. | NONE | Angiogenesis disorder;Ulcer;Cancer;Lung embolism;Restenosis;Metastasis;Skin ulcer;Cardiovascular disease |  |
| 3bzu_v | 5 | 2.958 | 0.5916 | 0.38718 | 3 | 1 | 1 | 0 | 0 | 0 | Corticosteroid 11-beta-dehydrogenase isozyme 1 | OXIDOREDUCTASE | DHI1_HUMAN | Catalyzes reversibly the conversion of cortisol to the inactive metabolite cortisone. Catalyzes reversibly the conversion of 7-ketocholesterol to 7-beta-hydroxycholesterol. In intact cells, the reaction runs only in one direction, from 7- ketocholesterol | Defects in HSD11B1 are a cause of cortisone reductase deficiency (CRD) [MIM:604931]. In CRD, activation of cortisone to cortisol does not occur, resulting in adrenocorticotropin-mediated androgen excess and a phenotype resembling polycystic ovary syndrome (PCOS). | Non-insulin dependent diabetes;Rheumatoid arthritis;Diabetes mellitus;Obesity;Atherosclerosis;Metabolic disorder;Periodontitis |  |
| 1rd4_v | 6 | 3.545 | 0.5909 | 1.42334 | 5 | 0 | 0 | 1 | 0 | 0 | Integrin alpha-L | IMMUNE SYSTEM | ITAL_HUMAN | Integrin alpha-L/beta-2 is a receptor for ICAM1, ICAM2, ICAM3 and ICAM4. It is involved in a variety of immune phenomena including leukocyte-endothelial cell interaction, cytotoxic T-cell mediated killing, and antibody dependent killing by granulocytes an | NONE | NONE |  |
| 1b56_v | 5 | 2.944 | 0.5889 | 0.182495 | 3 | 1 | 0 | 0 | 1 | 0 | Fatty acid-binding protein, epidermal | LIPID-BINDING | FABP5_HUMAN | High specificity for fatty acids. Highest affinity for C18 chain length. Decreasing the chain length or introducing double bonds reduces the affinity. May be involved in keratinocyte differentiation. | NONE | NONE |  |
| 2shp_v | 5 | 2.937 | 0.5875 | -0.312351 | 4 | 0 | 0 | 1 | 0 | 0 | Tyrosine-protein phosphatase non-receptor type 11 | TYROSINE PHOSPHATASE | PTN11_HUMAN | Acts downstream of various receptor and cytoplasmic protein tyrosine kinases to participate in the signal transduction from the cell surface to the nucleus. | Defects in PTPN11 are the cause of LEOPARD syndrome [MIM:151100]. It is an autosomal dominant disorder allelic with Noonan syndrome. The acronym LEOPARD stands for lentigines, electrocardiographic conduction abnormalities, ocular hypertelorism, pulmonic stenosis, abnormalities of genitalia, retardation of growth, and deafness.;Defects in PTPN11 are the cause of Noonan syndrome 1 (NS1) [MIM:163950]. Noonan syndrome (NS) is a disorder characterized by dysmorphic facial features, short stature, hypertelorism, cardiac anomalies, deafness, motor delay, and a bleeding diathesis. It is a genetically heterogeneous and relatively common syndrome, with an estimated incidence of 1 in 1000-2500 live births. Mutations in PTPN11 account for more than 50% of the cases. Rarely, NS is associated with juvenile myelomonocytic leukemia (JMML). NS1 inheritance is autosomal dominant.;Defects in PTPN11 are a cause of Noonan-like syndrome [MIM:163955]; also known as Noonan-like/multiple giant cell lesion syndrome. It is an autosomal dominant disorder characterized by Noonan features associates with giant cell lesions of bone and soft tissue.;Defects in PTPN11 are a cause of juvenile myelomonocytic leukemia (JMML) [MIM:607785]. JMML is a pediatric myelodysplastic syndrome that constitutes approximately 30% of childhood cases of myelodysplastic syndrome (MDS) and 2% of leukemia. It is characterized by leukocytosis with tissue infiltration and in vitro hypersensitivity of myeloid progenitors to granulocyte- macrophage colony stimulating factor. | NONE |  |
| 1q11_v | 5 | 2.935 | 0.587 | 0.2904 | 2 | 1 | 1 | 1 | 0 | 0 | Tyrosyl-tRNA synthetase, cytoplasmic | NONE | P54577 | Translation, ribosomal structure and biogenesis | NONE | Tyrosine is claimed to act as an effective antidepressant, however results are mixed. Tyrosine has also been claimed to reduce stress and combat narcolepsy and chronic fatigue, however these claims have been refuted by some studies. |  |
| 1sm2_v | 6 | 3.519 | 0.5865 | 1.27918 | 4 | 2 | 0 | 0 | 0 | 0 | Tyrosine-protein kinase ITK/TSK | NONE | Q08881 | Involved in protein kinase activity | NONE | NONE |  |
| 1cgh_v | 5 | 2.932 | 0.5865 | 0.522169 | 1 | 2 | 0 | 2 | 0 | 0 | Cathepsin G | COMPLEX (SERINE PROTEASE/INHIBITOR) | CATG_HUMAN | Serine protease with trypsin- and chymotrypsin-like specificity. | NONE | Connective tissue disease;Viral infection;Inflammation;HIV infection |  |
| 1rv1_v | 5 | 2.932 | 0.5864 | 0.0923583 | 4 | 0 | 1 | 0 | 0 | 0 | E3 ubiquitin-protein ligase Mdm2 | LIGASE | MDM2_HUMAN | Inhibits TP53/p53- and TP73/p73-mediated cell cycle arrest and apoptosis by binding its transcriptional activation domain. Functions as a ubiquitin ligase E3, in the presence of E1 and E2, toward p53 and itself. Permits the nuclear export of p53 and targe | Seems to be amplified in certain tumors (including soft tissue sarcomas, osteosarcomas and gliomas). A higher frequency of splice variants lacking p53 binding domain sequences was found in late-stage and high-grade ovarian and bladder carcinomas. Four of the splice variants show loss of p53 binding. | Leukemia;Cancer;Solid tumor |  |
| 1mq0_v | 5 | 2.925 | 0.585 | 0.348129 | 1 | 3 | 1 | 0 | 0 | 0 | Cytidine deaminase | HYDROLASE | CDD_HUMAN | This enzyme scavenge exogenous and endogenous cytidine and 2-deoxycytidine for UMP synthesis. | NONE | NONE |  |
| 2pjl_v | 5 | 2.91 | 0.5821 | 0.152668 | 4 | 0 | 0 | 1 | 0 | 0 | Steroid hormone receptor ERR1 | NONE | P11474 | Binds to an ERR-alpha response element (ERRE) containing a single consensus half-site, 5'-TNAAGGTCA-3'. Can bind to the medium-chain acyl coenzyme A dehydrogenase (MCAD) response element NRRE-1 and may act as an important regulator of MCAD promoter. Binds to the C1 region of the lactoferrin gene promoter. Requires dimerization and the coactivator, PGC-1A, for full activity. The ERRalpha/PGC1alpha complex is a regulator of energy metabolism. Induces the expression of PERM1 in the skeletal muscle. | NONE | NONE |  |
| 1fzv_v | 5 | 2.904 | 0.5808 | 0.16445 | 3 | 0 | 2 | 0 | 0 | 0 | Placenta growth factor | HORMONE/GROWTH FACTOR | PLGF_HUMAN | Growth factor active in angiogenesis, and endothelial cell growth, stimulating their proliferation and migration. It binds to receptor VEGFR-1/FLT1. PLGF-2 binds neuropilin-1 and 2 in a heparin-dependent manner. | NONE | Retinopathy;Vascular disease;Diabetic macular edema;Inflammation;Cancer;Gastrointestinal disease;Age related macular degeneration |  |
| 1gz4_v | 5 | 2.895 | 0.5791 | -0.422424 | 0 | 3 | 0 | 0 | 2 | 0 | NAD-dependent malic enzyme, mitochondrial | OXIDOREDUCTASE | MAOM_HUMAN | NONE | NONE | NONE |  |
| 2f57_v | 5 | 2.893 | 0.5787 | 0.31433 | 3 | 1 | 1 | 0 | 0 | 0 | Serine/threonine-protein kinase PAK 7 | TRANSFERASE | PAK7_HUMAN | The activated kinase acts on a variety of targets (By similarity). | NONE | NONE |  |
| 1utt_v | 5 | 2.89 | 0.578 | 0.00709335 | 2 | 3 | 0 | 0 | 0 | 0 | Macrophage metalloelastase | HYDROLASE | MMP12_HUMAN | May be involved in tissue injury and remodeling. Has significant elastolytic activity. Can accept large and small amino acids at the P1 site, but has a preference for leucine. Aromatic or hydrophobic residues are preferred at the P1 site, with small hydr | NONE | Chronic obstructive pulmonary disease |  |
| 2flr_v | 5 | 2.889 | 0.5779 | 0.184644 | 3 | 0 | 2 | 0 | 0 | 0 | Coagulation factor VII | HYDROLASE/BLOOD CLOTTING | FA7_HUMAN | Initiates the extrinsic pathway of blood coagulation. Serine protease that circulates in the blood in a zymogen form. Factor VII is converted to factor VIIa by factor Xa, factor XIIa, factor IXa, or thrombin by minor proteolysis. In the presence of tissue | Defects in F7 are the cause of factor VII deficiency [MIM:227500]. Factor VII deficiency is a rare hereditary hemorrhagic disease. The clinical picture can be very severe, with the early occurrence of intracerebral hemorrhages or hemarthroses, or, in contrast, moderate with cutaneous-mucosal hemorrhages (epistaxis, menorrhagia) or hemorrhages provoked by a surgical intervention. Numerous subjects are completely asymptomatic despite a very low F7 level. | Deep vein thrombosis;Blood clotting disorder;Cancer;Ebola virus infection;Thrombosis;Lung embolism;Unstable angina;Angina;Myocardial infarction;Thromboembolism;Cardiovascular disease |  |
| 2bu5_v | 6 | 3.463 | 0.5772 | 0.972474 | 5 | 1 | 0 | 0 | 0 | 0 | [Pyruvate dehydrogenase [lipoamide]] kinase isozyme 2, mitochondrial | TRANSFERASE | PDK2_HUMAN | Inhibits the mitochondrial pyruvate dehydrogenase complex by phosphorylation of the E1 alpha subunit, thus contributing to the regulation of glucose metabolism. | NONE | NONE |  |
| 1h6g_v | 5 | 2.878 | 0.5756 | 0.0823223 | 3 | 2 | 0 | 0 | 0 | 0 | Catenin alpha-1 | CYTOSKELETON | CTNA1_HUMAN | Associates with the cytoplasmic domain of a variety of cadherins. The association of catenins to cadherins produces a complex which is linked to the actin filament network, and which seems to be of primary importance for cadherins cell-adhesion properties | Abnormalities of alpha-catenin are involved in the process of cancer invasion and metastasis. | NONE |  |
| 1m6w_v | 5 | 2.868 | 0.5736 | -0.159579 | 3 | 0 | 1 | 0 | 1 | 0 | Alcohol dehydrogenase class-3 | OXIDOREDUCTASE | ADHX_HUMAN | Class-III ADH is remarkably ineffective in oxidizing ethanol, but it readily catalyzes the oxidation of long-chain primary alcohols and the oxidation of S-(hydroxymethyl) glutathione. | NONE | NONE |  |
| 1wlj_v | 5 | 2.862 | 0.5723 | -0.332394 | 0 | 4 | 1 | 0 | 0 | 0 | Interferon-stimulated gene 20 kDa protein | HYDROLASE | ISG20_HUMAN | Exonuclease with specificity for single-stranded RNA and, to a lesser extent for DNA. Degrades RNA at a rate that is approximately 35-fold higher than its rate for single-stranded DNA. Involved in the antiviral function of IFN against RNA viruses. | NONE | NONE |  |
| 1ld8_v | 4 | 2.286 | 0.5715 | -0.983929 | 2 | 1 | 0 | 1 | 0 | 0 | Protein farnesyltransferase/geranylgeranyltransferase type-1 subunit alpha | TRANSFERASE | FNTA_HUMAN | Catalyzes the transfer of a farnesyl or geranyl-geranyl moiety from farnesyl or geranyl-geranyl pyrophosphate to a cysteine at the fourth position from the C-terminus of several proteins having the C-terminal sequence Cys-aliphatic-aliphatic-X. The alpha | NONE | NONE |  |
| 1ewf_v | 5 | 2.845 | 0.569 | 0.442289 | 3 | 1 | 0 | 0 | 1 | 0 | Bactericidal permeability-increasing protein | NONE | P17213 | Involved in lipid binding | NONE | NONE |  |
| 1a5h_v | 5 | 2.842 | 0.5685 | 0.192315 | 2 | 1 | 0 | 2 | 0 | 0 | Tissue-type plasminogen activator | HYDROLASE | TPA_HUMAN | Converts the abundant, but inactive, zymogen plasminogen to plasmin by hydrolyzing a single Arg-Val bond in plasminogen. By controlling plasmin-mediated proteolysis, it plays an important role in tissue remodeling and degradation, in cell migration and ma | Increased activity of TPA is the cause of hyperfibrinolysis [MIM:173370]. Hyperfibrinolysis leads to excessive bleeding. Defective release of TPA causes hypofibrinolysis, leading to thrombosis or embolism. | Inflammation;Cancer;Myocardial infarction;Non-insulin dependent diabetes;Thrombosis |  |
| 1mrq_v | 5 | 2.838 | 0.5677 | -0.166067 | 4 | 1 | 0 | 0 | 0 | 0 | Aldo-keto reductase family 1 member C1 | NONE | Q04828 | Involved in oxidoreductase activity | NONE | Some evidence suggests that NADH might be useful in treating Parkinsons disease, chronic fatigue syndrome, Alzheimers disease and cardiovascular disease. |  |
| 2fq9_v | 5 | 2.818 | 0.5635 | 0.334757 | 2 | 1 | 1 | 1 | 0 | 0 | Cathepsin S | HYDROLASE | CATS_HUMAN | Thiol protease. Key protease responsible for the removal of the invariant chain from MHC class II molecules. The bond- specificity of this proteinase is in part similar to the specificities of cathepsin L and cathepsin N. | NONE | Psoriasis;Arteriosclerosis;Pain;Rheumatoid arthritis;Autoimmune disease;Allergy;Inflammation;Asthma;Multiple sclerosis;Immune disorder |  |
| 1bsx_v | 5 | 2.814 | 0.5628 | -0.036686 | 4 | 0 | 0 | 0 | 1 | 0 | Thyroid hormone receptor beta | NONE | P10828 | Nuclear hormone receptor that can act as a repressor or activator of transcription. High affinity receptor for thyroid hormones, including triiodothyronine and thyroxine. | NONE | NONE |  |
| 3bbt_v | 6 | 3.346 | 0.5577 | 1.05461 | 5 | 1 | 0 | 0 | 0 | 0 | Receptor tyrosine-protein kinase erbB-4 | TRANSFERASE | ERBB4_HUMAN | Specifically binds and is activated by neuregulins, NRG- 2, NRG-3, heparin-binding EGF-like growth factor, betacellulin and NTAK. Interaction with these factors induces cell differentiation. Not activated by EGF, TGF-A, and amphiregulin. | NONE | NONE |  |
| 2i6a_v | 5 | 2.788 | 0.5576 | 0.341355 | 2 | 1 | 1 | 0 | 0 | 1 | Adenosine kinase | TRANSFERASE | ADK_HUMAN | ATP dependent phosphorylation of adenosine and other related nucleoside analogs to monophosphate derivatives. Serves as a potential regulator of concentrations of extracellular adenosine and intracellular adenine nucleotides. | NONE | Septic shock;Pain;Inflammation;Seizure disorder;Seizure, epilepsy & convulsion;Epilepsy;Anesthesia |  |
| 1w6j_v | 5 | 2.75 | 0.5501 | -0.363878 | 2 | 2 | 1 | 0 | 0 | 0 | Lanosterol synthase | ISOMERASE | ERG7_HUMAN | Catalyzes the cyclization of (S)-2,3 oxidosqualene to lanosterol, a reaction that forms the sterol nucleus. | NONE | Hyperlipidemia;Hypercholesterolemia;Atherosclerosis |  |
| 1yvj_v | 6 | 3.297 | 0.5495 | 0.761701 | 4 | 1 | 1 | 0 | 0 | 0 | Tyrosine-protein kinase JAK3 | TRANSFERASE | JAK3_HUMAN | Tyrosine kinase of the non-receptor type, involved in the interleukin-2 and interleukin-4 signaling pathway. Phosphorylates STAT6, IRS1, IRS2 and PI3K. | Defects in JAK3 are a cause of severe combined immunodeficiency autosomal recessive T-cell-negative/B-cell- positive/NK-cell-negative (T(-)B(+)NK(-)SCID) [MIM:600802]. SCID refers to a genetically and clinically heterogeneous group of rare congenital disorders characterized by impairment of both humoral and cell-mediated immunity, leukopenia, and low or absent antibody levels. Patients with SCID present in infancy with recurrent, persistent infections by opportunistic organisms. The common characteristic of all types of SCID is absence of T-cell-mediated cellular immunity due to a defect in T-cell development. | Psoriasis;Transplant rejection;Rheumatoid arthritis;Respiratory tract inflammation;Autoimmune disease |  |
| 1xil_v | 5 | 2.743 | 0.5486 | -0.257693 | 2 | 2 | 1 | 0 | 0 | 0 | Superoxide dismutase [Mn], mitochondrial | NONE | P04179 | Destroys superoxide anion radicals which are normally produced within the cells and which are toxic to biological systems. | NONE | NONE |  |
| 1ayp_v | 5 | 2.741 | 0.5482 | -0.123487 | 3 | 1 | 0 | 0 | 1 | 0 | Phospholipase A2, membrane associated | NONE | P14555 | Involved in phospholipase A2 activity | NONE | NONE |  |
| 1o5e_v | 5 | 2.74 | 0.5481 | 0.262795 | 2 | 1 | 1 | 1 | 0 | 0 | Serine protease hepsin | NONE | P05981 | Plays an essential role in cell growth and maintenance of cell morphology | NONE | For treatment of hemorrhagic complications in hemophilia A and B |  |
| 3cm7_v | 7 | 3.774 | 0.5392 | 2.55483 | 3 | 2 | 0 | 1 | 0 | 1 | Baculoviral IAP repeat-containing protein 4 | NONE | P98170 | Involved in caspase inhibition and apoptosis suppression | NONE | NONE |  |
| 2o9i_v | 7 | 3.679 | 0.5255 | 1.32568 | 5 | 2 | 0 | 0 | 0 | 0 | Nuclear receptor subfamily 1 group I member 2 | TRANSCRIPTION | NR1I2_HUMAN | Orphan receptor; its natural ligand is probably pregnane. Binds to a response element in the CYP3A4 and ABCB1/MDR1 genes promoter. Activates its expression in response to a wide variety of endobiotics and xenobiotics. | NONE | NONE |  |
| 1g3m_v | 7 | 3.663 | 0.5233 | 1.12782 | 6 | 1 | 0 | 0 | 0 | 0 | Estrogen sulfotransferase | TRANSFERASE | ST1E1_HUMAN | May control the level of the estrogen receptor by sulfurylating free estradiol. Maximally sulfates beta-estradiol and estrone at concentrations of 20 nM. Also sulfates dehydroepiandrosterone, pregnenolone, ethinylestradiol, equalenin, diethylstilbesterol | NONE | NONE |  |
| 1xan_v | 4 | 2.073 | 0.5182 | -2.01996 | 2 | 1 | 1 | 0 | 0 | 0 | Glutathione reductase, mitochondrial | NONE | P00390 | RNA processing and modification | NONE | For nutritional supplementation, also for treating dietary shortage or imbalance |  |
| 3blr_v | 5 | 2.569 | 0.5139 | -0.329692 | 2 | 2 | 0 | 1 | 0 | 0 | Cyclin-T1 | TRANSCRIPTION | CCNT1_HUMAN | Regulatory subunit of the cyclin-dependent kinase pair (CDK9/cyclin-T1) complex, also called positive transcription elongation factor B (P-TEFb), which is proposed to facilitate the transition from abortive to productive elongation by phosphorylating the | NONE | NONE |  |
| 1x89_v | 7 | 3.59 | 0.5129 | 1.6646 | 5 | 1 | 0 | 0 | 1 | 0 | Neutrophil gelatinase-associated lipocalin | ANTIMICROBIAL PROTEIN | NGAL_HUMAN | Transport of small lipophilic substances (Potential). | NONE | NONE |  |
| 1qab_v | 6 | 3.077 | 0.5129 | 0.167327 | 5 | 0 | 1 | 0 | 0 | 0 | Retinol-binding protein 4 | TRANSPORT PROTEIN | RET4_HUMAN | Delivers retinol from the liver stores to the peripheral tissues. In plasma, the RBP-retinol complex interacts with transthyretin, this prevents its loss by filtration through the kidney glomeruli. | Defects in RBP4 are a cause of retinol-binding protein deficiency [MIM:180250]. This condition causes night vision problems. It produces a typical fundus xerophthalmicus, featuring a progressed atrophy of the retinal pigment epithelium.;A deficiency of vitamin A blocks secretion of the binding protein post-translationally and results in defective delivery and supply of vitamin to the epidermal cells (a condition associated with a dermatosis). | NONE |  |
| 1t2f_v | 5 | 2.53 | 0.5061 | -1.40417 | 0 | 3 | 1 | 0 | 1 | 0 | L-lactate dehydrogenase B chain | NONE | P07195 | Energy production and conversion | NONE | Some evidence suggests that NADH might be useful in treating Parkinsons disease, chronic fatigue syndrome, Alzheimers disease and cardiovascular disease. |  |
| 1mmq_v | 5 | 2.515 | 0.503 | -0.786702 | 1 | 2 | 2 | 0 | 0 | 0 | Matrilysin | METALLOPROTEASE | MMP7_HUMAN | Degrades casein, gelatins of types I, III, IV, and V, and fibronectin. Activates procollagenase. | NONE | NONE |  |
| 2vd1_v | 6 | 3.013 | 0.5022 | -0.128063 | 5 | 1 | 0 | 0 | 0 | 0 | Glutathione-requiring prostaglandin D synthase | ISOMERASE | PTGD2_HUMAN | Catalyzes the conversion of PGH2 to PGD2, a prostaglandin involved in smooth muscle contraction/relaxation and a potent inhibitor of platelet aggregation. | NONE | NONE |  |
| 1cg6_v | 5 | 2.51 | 0.5021 | -1.13819 | 2 | 1 | 2 | 0 | 0 | 0 | S-methyl-5-thioadenosine phosphorylase | NONE | Q13126 | Nucleotide transport and metabolism | NONE | For nutritional supplementation, also for treating dietary shortage or imbalance |  |
| 1wda_v | 6 | 3.006 | 0.501 | 0.110613 | 2 | 2 | 1 | 1 | 0 | 0 | Protein-arginine deiminase type-4 | HYDROLASE | PADI4_HUMAN | Catalyzes the citrullination/deimination of arginine residues of proteins. Citrullinates histone H3 at Arg-8 and/or Arg-17 and histone H4 at Arg-3, which prevents their methylation by CARM1 and HRMT1L2/PRMT1 and represses transcription. Citrullinate | PADI4 is thought to be a rheumatoid arthritis susceptibility locus [MIM:180300]. Could have an important role in the pathogenesis of rheumatoid arthritis by increasing citrullination of proteins in rheumatoid arthritis synovial tissues, leading, in a cytokine-rich milieu, to a break in tolerance to citrullinated peptides processed and presented in the appropriate HLA context. | NONE |  |
| 1i7g_v | 6 | 2.993 | 0.4988 | -0.026619 | 4 | 2 | 0 | 0 | 0 | 0 | Peroxisome proliferator-activated receptor alpha | TRANSCRIPTION | PPARA_HUMAN | Receptor that binds peroxisome proliferators such as hypolipidemic drugs and fatty acids. Once activated by a ligand, the receptor binds to a promoter element in the gene for acyl-CoA oxidase and activates its transcription. It therefore controls the pero | NONE | Syndrome X;Lipid metabolism disorder;Non-insulin dependent diabetes;Atherosclerosis;Hypertriglyceridemia;Hyperlipidemia;Cardiovascular disease;Congestive heart failure;Diabetes mellitus;Obesity;Inflammation |  |
| 1xvp_v | 6 | 2.991 | 0.4984 | -0.113433 | 6 | 0 | 0 | 0 | 0 | 0 | Nuclear receptor subfamily 1 group I member 3 | DNA BINDING PROTEIN | NR1I3_HUMAN | Binds and transactivates the retinoic acid response elements that control expression of the retinoic acid receptor beta 2 and alcohol dehydrogenase 3 genes. Transactivates both the phenobarbital responsive element module of the human CYP2B6 gene and the C | NONE | NONE |  |
| 2obf_v | 5 | 2.48 | 0.496 | -0.717216 | 3 | 0 | 1 | 1 | 0 | 0 | Phenylethanolamine N-methyltransferase | TRANSFERASE | PNMT_HUMAN | Converts noradrenaline to adrenaline. | NONE | NONE |  |
| 1iz2_v | 6 | 2.975 | 0.4958 | 0.193104 | 3 | 1 | 2 | 0 | 0 | 0 | Alpha-1-antitrypsin | PROTEIN BINDING | A1AT_HUMAN | Inhibitor of serine proteases. Its primary target is elastase, but it also has a moderate affinity for plasmin and thrombin. Inhibits trypsin, chymotrypsin and plasminogen activator. The aberrant form inhibits insulin-induced NO synthesis in platelets, de | The major physiological function of AAT is the protection of the lower respiratory tract against proteolytic destruction by human leukocyte elastase (HLE). A hereditary deficiency of AAT, is associated with a 20-30 fold increased risk of developing chronic obstructive pulmonary disease.;Deficiency of the normal inhibitor in individuals homozygous for allele Z or M-Malton can result in the development of chronic emphysema or infantile liver cirrhosis.;Variant Pittsburgh is the cause of bleeding diathesis. | Psoriasis;Genital tract inflammation;Otitis media;Wound healing;Atopic dermatitis;Dermatitis;Cystitis;Inflammatory bowel disease;Cystic fibrosis;Alpha-1 antitrypsin deficiency;Dermatological disease |  |
| 1lv2_v | 7 | 3.463 | 0.4947 | 0.700971 | 5 | 1 | 0 | 0 | 1 | 0 | Hepatocyte nuclear factor 4-gamma | NONE | Q14541 | Involved in transcription factor activity | NONE | NONE |  |
| 1lzj_v | 6 | 2.968 | 0.4946 | 0.580536 | 2 | 2 | 2 | 0 | 0 | 0 | B transferase | NONE | Q9NY01 | Involved in transferase activity, transferring hexosyl groups | NONE | NONE |  |
| 1y0x_v | 6 | 2.953 | 0.4921 | 0.290601 | 4 | 0 | 1 | 0 | 1 | 0 | Thyroid hormone receptor beta-2 | NONE | P37243 | Involved in transcription factor activity | NONE | Used as replacement or supplemental therapy in patients with hypothyroidism of any etiology, except transient hypothyrodism during the recovery phase of subacute thyroiditis. |  |
| 1gzr_v | 6 | 2.952 | 0.492 | 0.0409742 | 3 | 2 | 0 | 1 | 0 | 0 | Insulin-like growth factor IA | NONE | P01343 | NONE | NONE | NONE |  |
| 1r7t_v | 6 | 2.949 | 0.4914 | 0.0663199 | 3 | 1 | 2 | 0 | 0 | 0 | Histo-blood group ABO system transferase | NONE | P16442 | Involved in transferase activity, transferring hexosyl groups | NONE | NONE |  |
| 3edz_v | 5 | 2.455 | 0.491 | -0.21398 | 2 | 2 | 0 | 0 | 0 | 1 | Disintegrin and metalloproteinase domain-containing protein 17 | NONE | P78536 | Cleaves the membrane-bound precursor of TNF-alpha to its mature soluble form. Responsible for the proteolytical release of soluble JAM3 from endothelial cells surface. Responsible for the proteolytic release of several other cell-surface proteins, including p75 TNF-receptor, interleukin 1 receptor type II, p55 TNF-receptor, transforming growth factor-alpha, L-selectin, growth hormone receptor, MUC1 and the amyloid precursor protein. Acts as an activator of Notch pathway by mediating cleavage of Notch, generating the membrane-associated intermediate fragment called Notch extracellular truncation (NEXT). Plays a role in the proteolytic processing of ACE2. | NONE | NONE |  |
| 1sz7_v | 6 | 2.944 | 0.4907 | -0.288156 | 5 | 1 | 0 | 0 | 0 | 0 | Trafficking protein particle complex subunit 3 | NONE | O43617 | May play a role in vesicular transport from endoplasmic reticulum to Golgi | NONE | NONE |  |
| 1nd5_v | 6 | 2.928 | 0.488 | -0.394427 | 2 | 2 | 0 | 1 | 1 | 0 | Prostatic acid phosphatase | HYDROLASE | PPAP_HUMAN | NONE | NONE | Prostate tumor |  |
| 1w7n_v | 6 | 2.926 | 0.4877 | -0.177911 | 1 | 2 | 1 | 1 | 1 | 0 | Kynurenine--oxoglutarate transaminase 1 | TRANSFERASE | KAT1_HUMAN | Catalyzes the irreversible transamination of the L- tryptophan metabolite L-kynurenine to form kynurenic acid (KA). Metabolizes the cysteine conjugates of certain halogenated alkenes and alkanes to form reactive metabolites. Catalyzes the beta- eliminatio | NONE | NONE |  |
| 1tjj_v | 6 | 2.918 | 0.4864 | 0.0846717 | 4 | 1 | 0 | 1 | 0 | 0 | Ganglioside GM2 activator | NONE | P17900 | Involved in sphingolipid activator protein activity | NONE | NONE |  |
| 2qtu_v | 5 | 2.429 | 0.4857 | -0.975647 | 3 | 1 | 1 | 0 | 0 | 0 | Estrogen receptor beta | NONE | Q92731 | Involved in transcription factor activity | NONE | For the prevention of osteoporosis in post-menopausal women |  |
| 1gse_v | 6 | 2.906 | 0.4844 | -0.44357 | 5 | 1 | 0 | 0 | 0 | 0 | Glutathione S-transferase A1 | NONE | P08263 | Involved in glutathione transferase activity | NONE | For nutritional supplementation, also for treating dietary shortage or imbalance |  |
| 1hms_v | 8 | 3.867 | 0.4834 | 1.00216 | 6 | 1 | 0 | 0 | 1 | 0 | Fatty acid-binding protein, heart | LIPID-BINDING PROTEIN | FABPH_HUMAN | FABP are thought to play a role in the intracellular transport of long-chain fatty acids and their acyl-CoA esters. | NONE | NONE |  |
| 3d7t_v | 6 | 2.885 | 0.4809 | -0.011164 | 3 | 1 | 1 | 1 | 0 | 0 | Tyrosine-protein kinase CSK | TRANSFERASE | CSK_HUMAN | Specifically phosphorylates Tyr-504 on LCK, which acts as a negative regulatory site. Can also act on the LYN and FYN kinases. | NONE | NONE |  |
| 3f7h_v | 6 | 2.884 | 0.4806 | 0.132585 | 2 | 2 | 1 | 1 | 0 | 0 | Baculoviral IAP repeat-containing protein 7 | NONE | Q96CA5 | Apoptotic regulator capable of exerting proapoptotic and anti-apoptotic activities and plays crucial roles in apoptosis, cell proliferation, and cell cycle control. Its anti-apoptotic activity is mediated through the inhibition of CASP3, CASP7 and CASP9, as well as by its E3 ubiquitin-protein ligase activity. As it is a weak caspase inhibitor, its anti-apoptotic activity is thought to be due to its ability to ubiquitinate DIABLO/SMAC targeting it for degradation thereby promoting cell survival. May contribute to caspase inhibition, by blocking the ability of DIABLO/SMAC to disrupt XIAP/BIRC4-caspase interactions. Protects against apoptosis induced by TNF or by chemical agents such as adriamycin, etoposide or staurosporine. Suppression of apoptosis is mediated by activation of MAPK8/JNK1, and possibly also of MAPK9/JNK2. This activation depends on TAB1 and NR2C2/TAK1. In vitro, inhibits CASP3 and proteolytic activation of pro-CASP9. Isoform 1 blocks staurosporine-induced apoptosis. Isoform 2 blocks etoposide-induced apoptosis. Isoform 2 protects against natural killer (NK) cell killing whereas isoform 1 augments killing. | NONE | NONE |  |
| 1lf7_v | 5 | 2.39 | 0.478 | -1.68081 | 0 | 3 | 0 | 0 | 2 | 0 | Complement component C8 gamma chain | NONE | P07360 | Involved in transporter activity | NONE | NONE |  |
| 1qcf_v | 7 | 3.335 | 0.4765 | 0.469647 | 3 | 3 | 1 | 0 | 0 | 0 | Tyrosine-protein kinase HCK | TYROSINE KINASE | HCK_HUMAN | May serve as part of a signaling pathway coupling the Fc receptor to the activation of the respiratory burst. May also contribute to neutrophil migration and may regulate the degranulation process of neutrophils. | NONE | NONE |  |
| 1u4l_v | 6 | 2.854 | 0.4757 | -0.0170576 | 0 | 5 | 1 | 0 | 0 | 0 | C-C motif chemokine 5 | ATTRACTANT | CCL5_HUMAN | Chemoattractant for blood monocytes, memory T-helper cells and eosinophils. Causes the release of histamine from basophils and activates eosinophils. Binds to CCR1, CCR3, CCR4 and CCR5. One of the major HIV-suppressive factors produced by CD8+ T- cells. R | NONE | NONE |  |
| 1qdd_v | 5 | 2.379 | 0.4757 | -1.18836 | 0 | 4 | 1 | 0 | 0 | 0 | Lithostathine-1-alpha | NONE | P05451 | Involved in sugar binding | NONE | NONE |  |
| 1bj4_v | 6 | 2.831 | 0.4719 | -0.0264642 | 2 | 4 | 0 | 0 | 0 | 0 | Serine hydroxymethyltransferase, cytosolic | TRANSFERASE | GLYC_HUMAN | Interconversion of serine and glycine. | NONE | NONE |  |
| 1uej_v | 6 | 2.83 | 0.4716 | -0.195836 | 1 | 3 | 2 | 0 | 0 | 0 | Uridine-cytidine kinase 2 | NONE | Q9BZX2 | Nucleotide transport and metabolism | NONE | NONE |  |
| 4ald_v | 6 | 2.829 | 0.4716 | -0.360508 | 0 | 4 | 1 | 0 | 1 | 0 | Fructose-bisphosphate aldolase A | LYASE | ALDOA_HUMAN | NONE | Defects in ALDOA are the cause of aldolase A deficiency [MIM:611881]; also known as aldoA deficiency or red cell aldolase deficiency. Aldolase A deficiency is an autosomal recessive disorder associated with hereditary hemolytic anemia. | NONE |  |
| 1fe3_v | 7 | 3.293 | 0.4704 | 0.0627421 | 6 | 0 | 0 | 0 | 1 | 0 | Fatty acid-binding protein, brain | LIPID BINDING PROTEIN | FABP7_HUMAN | B-FABP could be involved in the transport of a so far unknown hydrophobic ligand with potential morphogenic activity during CNS development. It is required for the establishment of the radial glial fiber system in developing brain, a system that is necess | NONE | NONE |  |
| 1isg_v | 6 | 2.819 | 0.4699 | -0.459538 | 0 | 5 | 1 | 0 | 0 | 0 | ADP-ribosyl cyclase 2 | HYDROLASE | BST1_HUMAN | Synthesizes cyclic ADP-ribose, a second messenger that elicits calcium release from intracellular stores. May be involved in pre-B-cell growth. | Rheumatoid arthritis (RA) patients show enhanced expression of BST-1 transcripts in bone marrow stromal cell lines. This suggests that BST-1 overexpression may play a role in B-cell abnormalities in RA. | Hematological neoplasm;Multiple myeloma |  |
| 1fd0_v | 8 | 3.757 | 0.4696 | 1.2748 | 6 | 1 | 0 | 0 | 1 | 0 | Retinoic acid receptor gamma | GENE REGULATION | RARG_HUMAN | This is a receptor for retinoic acid. This metabolite has profound effects on vertebrate development. Retinoic acid is a morphogen and is a powerful teratogen. This receptor controls cell function by directly regulating gene expression. | NONE | Emphysema |  |
| 1o1v_v | 7 | 3.284 | 0.4691 | 0.234546 | 5 | 1 | 1 | 0 | 0 | 0 | Gastrotropin | LIPID BINDING PROTEIN | FABP6_HUMAN | Ileal protein which stimulates gastric acid and pepsinogen secretion. Seems to be able to bind to bile salts and bilirubins. | NONE | NONE |  |
| 2hzi_v | 8 | 3.741 | 0.4676 | 1.63703 | 6 | 1 | 1 | 0 | 0 | 0 | Proto-oncogene tyrosine-protein kinase ABL1 | NONE | P00519 | Involved in protein kinase activity | NONE | For nutritional supplementation, also for treating dietary shortage or imbalance |  |
| 2b7a_v | 7 | 3.241 | 0.4631 | 0.360786 | 3 | 2 | 2 | 0 | 0 | 0 | Tyrosine-protein kinase JAK2 | TRANSFERASE | JAK2_HUMAN | Plays a role in leptin signaling and control of body weight (By similarity). Tyrosine kinase of the non-receptor type, involved in interleukin-3 and probably interleukin-23 signal transduction. | Chromosomal aberrations involving JAK2 are found in both chronic and acute forms of eosinophilic, lymphoblastic and myeloid leukemia. Translocation t(8;9)(p22;p24) with PCM1 links the protein kinase domain of JAK2 to the major portion of PCM1. Translocation t(9;12)(p24;p13) with ETV6.;Defects in JAK2 are a cause of susceptibility to Budd- Chiari syndrome [MIM:600880]. Budd-Chiari syndrome is a spectrum of disease states, including anatomic abnormalities and hypercoagulable disorders, resulting in hepatic venous outflow occlusion. Clinical manifestations observed in the majority of patients include hepatomegaly, right upper quadrant pain, and abdominal ascites.;Defects in JAK2 are associated with polycythemia vera (PV) [MIM:263300]. PV, the most common form of primary polycythemia, is caused by somatic mutation in a single hematopoietic stem cell leading to clonal hematopoiesis. PV is a myeloproliferative disorder characterized predominantly by erythroid hyperplasia, but also by myeloid leukocytosis, thrombocytosis, and splenomegaly. Familial cases of PV are very rare and usually manifest in elderly patients.;Defects in JAK2 gene may be a cause of essential thrombocythemia (ET) [MIM:187950]. ET is characterized by elevated platelet levels due to sustained proliferation of megakaryocytes, and frequently lead to thrombotic and haemorrhagic complications.;Defects in JAK2 are associated with familial myelofibrosis [MIM:254450]. Myelofibrosis with myeloid metaplasia is a myeloproliferative disease with annual incidence of 0.5-1.5 cases per 100,000 individuals and age at diagnosis around 60 (an increased prevalence is noted in Ashkenazi Jews). Clinical manifestations depend on the type of blood cell affected and may include anemia, pallor, splenomegaly, hypermetabolic state, petechiae, ecchymosis, bleeding, lymphadenopathy, hepatomegaly, portal hypertension.;Defects in JAK2 are a cause of acute myelogenous leukemia (AML) [MIM:601626]. AML is a malignant disease in which hematopoietic precursors are arrested in an early stage of development. | Psoriasis;Cancer;Rheumatoid arthritis;Pulmonary hypertension;Myeloid leukemia;Inflammation;Prostate tumor;Pancreas tumor;Myeloproliferative disorder;Solid tumor;Myelofibrosis;Hematological neoplasm;Cardiovascular disease |  |
| 1dkf_v | 8 | 3.683 | 0.4604 | 1.21589 | 5 | 3 | 0 | 0 | 0 | 0 | Retinoic acid receptor alpha | HORMONE/GROWTH FACTOR RECEPTOR | RARA_HUMAN | This is a receptor for retinoic acid. This metabolite has profound effects on vertebrate development. Retinoic acid is a morphogen and is a powerful teratogen. This receptor controls cell function by directly regulating gene expression. | Chromosomal aberrations involving RARA may be a cause of acute promyelocytic leukemia (APL). Translocation t(11;17)(q32;q21) with ZBTB16/PLZF; translocation t(15;17)(q21;q21) with PML; translocation t(5;17)(q32;q11) with NPM. | Squamous cell carcinoma;Psoriasis;Colon tumor;Cancer;Metabolic disorder;Leukemia;Breast tumor;Skin infection;Neutropenia;Carcinoma;Dermatological disease;Ocular disease;Genital system disease;Lymphoma;Immune disorder |  |
| 1r1i_v | 8 | 3.673 | 0.4592 | 1.5485 | 3 | 2 | 1 | 1 | 1 | 0 | Neprilysin | HYDROLASE | NEP_HUMAN | Thermolysin-like specificity, but is almost confined on acting on polypeptides of up to 30 amino acids. Biologically important in the destruction of opioid peptides such as Met- and Leu-enkephalins by cleavage of a Gly-Phe bond. Involved in the degradatio | Important cell surface marker in the diagnostic of human acute lymphocytic leukemia. | Hypertension;Neuropathic pain;Renovascular hypertension;Congestive heart failure |  |
| 1jqe_v | 8 | 3.672 | 0.459 | 0.807655 | 7 | 0 | 0 | 1 | 0 | 0 | Histamine N-methyltransferase | NONE | P50135 | Inactivates histamine by N-methylation. Plays an important role in degrading histamine and in regulating the airway response to histamine | NONE | For treatment of acute malarial attacks in non-immune subjects. |  |
| 1xoi_v | 7 | 3.207 | 0.4582 | 0.246574 | 2 | 4 | 0 | 0 | 1 | 0 | Glycogen phosphorylase, liver form | NONE | P06737 | Carbohydrate transport and metabolism | NONE | For nutritional supplementation and for treating dietary shortage or imbalance. |  |
| 1s9j_v | 9 | 4.063 | 0.4514 | 1.11052 | 7 | 2 | 0 | 0 | 0 | 0 | Dual specificity mitogen-activated protein kinase kinase 1 | TRANSFERASE | MP2K1_HUMAN | Catalyzes the concomitant phosphorylation of a threonine and a tyrosine residue in a Thr-Glu-Tyr sequence located in MAP kinases. Activates ERK1 and ERK2 MAP kinases. | Defects in MAP2K1 are a cause of cardiofaciocutaneous syndrome (CFC syndrome) [MIM:115150]; also known as cardio-facio- cutaneous syndrome. CFC syndrome is characterized by a distinctive facial appearance, heart defects and mental retardation. Heart defects include pulmonic stenosis, atrial septal defects and hypertrophic cardiomyopathy. Some affected individuals present with ectodermal abnormalities such as sparse, friable hair, hyperkeratotic skin lesions and a generalized ichthyosis-like condition. Typical facial features are similar to Noonan syndrome. They include high forehead with bitemporal constriction, hypoplastic supraorbital ridges, downslanting palpebral fissures, a depressed nasal bridge, and posteriorly angulated ears with prominent helices. The inheritance of CFC syndrome is autosomal dominant. | Colon tumor;Melanoma;Cancer;Rheumatoid arthritis;Non-small-cell lung cancer;Neurodegenerative disease;Breast tumor;Colorectal tumor;Transplant rejection;Inflammation;Pancreas tumor;Solid tumor |  |
| 1xjd_v | 7 | 3.155 | 0.4507 | 0.191412 | 4 | 1 | 1 | 1 | 0 | 0 | Protein kinase C theta type | TRANSFERASE | KPCT_HUMAN | This is a calcium-independent, phospholipid-dependent, serine- and threonine-specific enzyme. Essential for T-cell receptor (TCR)-mediated T-cell activation, but is dispensable during TCR-dependent thymocyte development. Links the TCR signaling complex to;PKC is activated by diacylglycerol which in turn phosphorylates a range of cellular proteins. PKC also serves as the receptor for phorbol esters, a class of tumor promoters. | NONE | Inflammation |  |
| 1u59_v | 7 | 3.149 | 0.4499 | 0.235762 | 4 | 1 | 2 | 0 | 0 | 0 | Tyrosine-protein kinase ZAP-70 | TRANSFERASE | ZAP70_HUMAN | Plays a role in T-cell development and lymphocyte activation. Essential for TCR-mediated IL-2 production. Isoform 1 induces TCR-mediated signal transduction, isoform 2 does not. | Defects in ZAP70 are the cause of selective T-cell defect (STD) [MIM:176947]. STD is an autosomal recessive form of severe combined immunodeficiency characterized by a selective absence of CD8-type T-cells. | Transplant rejection;Immune disorder |  |
| 1n69_v | 7 | 3.133 | 0.4475 | 0.251169 | 6 | 0 | 0 | 0 | 1 | 0 | Proactivator polypeptide | NONE | P07602 | Involved in enzyme activator activity | NONE | NONE |  |
| 2oo8_v | 8 | 3.504 | 0.438 | 0.797438 | 5 | 1 | 1 | 1 | 0 | 0 | Angiopoietin-1 receptor | TRANSFERASE | TIE2_HUMAN | This protein is a protein tyrosine-kinase transmembrane receptor for angiopoietin 1. It may constitute the earliest mammalian endothelial cell lineage marker. Probably regulates endothelial cell proliferation, differentiation and guides the proper pattern | Defects in TEK are a cause of dominantly inherited venous malformations (VMCM) [MIM:600195]; an error of vascular morphogenesis characterized by dilated, serpiginous channels. | Retinopathy;Ovary tumor;Cancer;Renal cell carcinoma;Breast tumor;Solid tumor |  |
| 1dyt_v | 6 | 2.623 | 0.4371 | -1.1977 | 0 | 5 | 1 | 0 | 0 | 0 | Eosinophil cationic protein | NONE | P12724 | Involved in nucleic acid binding | NONE | Used as an adjunct to the standard therapy of inhaled steroids with inhaled long- and/or short-acting beta-agonists. |  |
| 3cbs_v | 9 | 3.913 | 0.4347 | 0.780066 | 6 | 2 | 0 | 0 | 1 | 0 | Cellular retinoic acid-binding protein 2 | NONE | P29373 | Involved in retinoid binding | NONE | For topical treatment of cutaneous lesions in patients with AIDS-related Kaposis sarcoma. |  |
| 1s8c_v | 8 | 3.462 | 0.4327 | 1.72326 | 5 | 2 | 1 | 0 | 0 | 0 | Heme oxygenase 1 | OXIDOREDUCTASE | HMOX1_HUMAN | Heme oxygenase cleaves the heme ring at the alpha methene bridge to form biliverdin. Biliverdin is subsequently converted to bilirubin by biliverdin reductase. Under physiological conditions, the activity of heme oxygenase is highest in the spleen, where | NONE | Sepsis;Cancer |  |
| 3cld_v | 8 | 3.459 | 0.4324 | 1.2385 | 7 | 0 | 1 | 0 | 0 | 0 | Glucocorticoid receptor | TRANSCRIPTION | GCR_HUMAN | Receptor for glucocorticoids (GC). Has a dual mode of action: as a transcription factor that binds to glucocorticoid response elements (GRE) and as a modulator of other transcription factors. Affects inflammatory responses, cellular proliferation and diff | Defects in NR3C1 are a cause of glucocorticoid resistance [MIM:138040]; also known as cortisol resistance. It is a hypertensive, hyperandrogenic disorder characterized by increased serum cortisol concentrations. Inheritance is autosomal dominant. | Alzheimers disease;Immune deficiency;Breast tumor;Major depressive disorder;Glaucoma;Metabolic disorder;Psychotic disorder;HIV infection;Cocaine addiction;Inflammation;Planned abortion;Diabetes mellitus;Weight gain;Endometriosis;Obesity;Insulin dependent diabetes;Cushings disease;Glioma;Endometroid carcinoma;Neurodegenerative disease;Hypertension;Hepatitis C virus infection;Carcinoma;Non-insulin dependent diabetes;Psychiatric disorder |  |
| 1hov_v | 7 | 3.015 | 0.4307 | 0.16853 | 4 | 1 | 2 | 0 | 0 | 0 | 72 kDa type IV collagenase | HYDROLASE/HYDROLASE INHIBITOR | MMP2_HUMAN | In addition to gelatin and collagens, it cleaves KiSS1 at a Gly- | -Leu bond. | Defects in MMP2 are the cause of multicentric osteolysis nodulosis and arthropathy (MONA) [MIM:605156]. Inherited osteolyses or vanishing bone syndromes are rare disorders of unknown etiology characterized by destruction and resorption of affected bones. MONA is an autosomal recessive osteolysis with multicentric involvement characterized by carpal and tarsal resorption, crippling arthritic changes, marked osteoporosis, palmar and plantar subcutaneous nodules and distinctive facies.;Defects in MMP2 are the cause of Winchester syndrome [MIM:277950]. Winchester syndrome is an autosomal recessive osteolysis syndrome. Winchester syndrome is severe with generalized osteolysis and osteopenia. Subcutaneous nodules are usually absent. Winchester syndrome has been associated with a number of additional features including coarse face, corneal opacities, patches of thickened, hyperpigmented skin, hypertrichosis and gum hypertrophy. However, these features are not always present and have occasionally been observed in other osteolysis syndromes. The clinical and molecular findings suggest that Winchester syndrome and MONA are allelic disorders that form a continuous clinical spectrum. | NONE |
| 1m48_v | 7 | 3.005 | 0.4292 | 0.0114932 | 4 | 1 | 2 | 0 | 0 | 0 | Interleukin-2 | CYTOKINE | IL2_HUMAN | Produced by T-cells in response to antigenic or mitogenic stimulation, this protein is required for T-cell proliferation and other activities crucial to regulation of the immune response. Can stimulate B-cells, monocytes, lymphokine- activated killer cell | A chromosomal aberration involving IL2 is found in a form of T-cell acute lymphoblastic leukemia (T-ALL). Translocation t(4;16)(q26;p13) with involves TNFRSF17. | Cancer |  |
| 1p62_v | 7 | 2.998 | 0.4283 | 0.075204 | 3 | 3 | 1 | 0 | 0 | 0 | Deoxycytidine kinase | TRANSFERASE | DCK_HUMAN | Required for the phosphorylation of the deoxyribonucleosides deoxycytidine (dC), deoxyguanosine (dG) and deoxyadenosine (dA). It is also an essential enzyme for the phosphorylation of numerous nucleoside analogs widely employed as antiviral and chemothera | NONE | NONE |  |
| 1irj_v | 7 | 2.986 | 0.4266 | -0.0627585 | 4 | 0 | 3 | 0 | 0 | 0 | Protein S100-A9 | METAL BINDING PROTEIN | S10A9_HUMAN | Expressed by macrophages in acutely inflammated tissues and in chronic inflammations. Seem to be an inhibitor of protein kinases. Also expressed in epithelial cells constitutively or induced during dermatoses. May interact with components of the intermedi | NONE | NONE |  |
| 1nav_v | 7 | 2.979 | 0.4255 | -0.452857 | 4 | 2 | 1 | 0 | 0 | 0 | Thyroid hormone receptor alpha | NONE | P10827 | Involved in transcription factor activity | NONE | Used as replacement or supplemental therapy in patients with hypothyroidism of any etiology, except transient hypothyrodism during the recovery phase of subacute thyroiditis. |  |
| 1t46_v | 9 | 3.821 | 0.4245 | 1.50333 | 5 | 1 | 1 | 2 | 0 | 0 | Mast/stem cell growth factor receptor | TRANSFERASE ACTIVATOR | KIT_HUMAN | This is the receptor for stem cell factor (mast cell growth factor). It has a tyrosine-protein kinase activity. Binding of the ligands leads to the autophosphorylation of KIT and its association with substrates such as phosphatidylinositol 3-kinase (Pi3K) | Defects in KIT are a cause of piebaldism [MIM:172800]. Piebaldism is an autosomal dominant genetic developmental abnormality of pigmentation characterized by congenital patches of white skin and hair that lack melanocytes.;Defects in KIT are a cause of gastrointestinal stromal tumor (GIST) [MIM:606764].;Defects in KIT have been associated with testicular tumors [MIM:273300]. It includes germ cell tumor (GCT) or testicular germ cell tumor (TGCT). | Pancreas tumor;Ovary tumor;Cancer;Mastocytosis;Gastrointestinal tumor |  |
| 1itu_v | 8 | 3.396 | 0.4244 | 0.214057 | 4 | 2 | 1 | 0 | 1 | 0 | Dipeptidase 1 | HYDROLASE | DPEP1_HUMAN | Hydrolyzes a wide range of dipeptides. Implicated in the renal metabolism of glutathione and its conjugates. Converts leukotriene D4 to leukotriene E4; it may play an important role in the regulation of leukotriene activity. | NONE | NONE |  |
| 1tfg_v | 9 | 3.793 | 0.4214 | 1.36976 | 4 | 2 | 2 | 1 | 0 | 0 | Transforming growth factor beta-2 | GROWTH FACTOR | TGFB2_HUMAN | TGF-beta 2 has suppressive effects on interleukin-2 dependent T-cell growth. | A chromosomal aberration involving TGFB2 is found in a family with Peters anomaly [MIM:604229]. Translocation t(1;7)(q41;p21) with HDAC9. Peters anomaly consists of a central corneal leukoma, absence of the posterior corneal stroma and Descemet membrane, and a variable degree of iris and lenticular attachments to the central aspect of the posterior cornea. | NONE |  |
| 1pq2_v | 7 | 2.947 | 0.421 | -0.349112 | 5 | 1 | 0 | 0 | 1 | 0 | Cytochrome P450 2C8 | OXIDOREDUCTASE | CP2C8_HUMAN | Cytochromes P450 are a group of heme-thiolate monooxygenases. In liver microsomes, this enzyme is involved in an NADPH-dependent electron transport pathway. It oxidizes a variety of structurally unrelated compounds, including steroids, fatty acids, and xe | NONE | NONE |  |
| 1q22_v | 7 | 2.941 | 0.4201 | -0.444533 | 6 | 1 | 0 | 0 | 0 | 0 | Sulfotransferase family cytosolic 2B member 1 | NONE | O00204 | Involved in sulfotransferase activity | NONE | NONE |  |
| 1m6d_v | 7 | 2.928 | 0.4183 | 0.0942902 | 3 | 2 | 2 | 0 | 0 | 0 | Cathepsin F | NONE | Q9UBX1 | Involved in cysteine-type endopeptidase activity | NONE | NONE |  |
| 1p5j_v | 7 | 2.914 | 0.4163 | -0.474732 | 2 | 4 | 0 | 0 | 1 | 0 | L-serine dehydratase | NONE | P20132 | Amino acid transport and metabolism | NONE | For nutritional supplementation and for treating dietary shortage or imbalance. |  |
| 1iri_v | 7 | 2.913 | 0.4162 | -0.537718 | 0 | 6 | 1 | 0 | 0 | 0 | Glucose-6-phosphate isomerase | ISOMERASE | G6PI_HUMAN | Neurotrophic factor for spinal and sensory neurons. | Defects in GPI are a cause of hereditary nonspherocytic hemolytic anemia (HA) [MIM:172400]. Severe GPI deficiency can be associated with hydrops fetalis, immediate neonatal death and neurological impairment. | NONE |  |
| 2aeb_v | 7 | 2.908 | 0.4155 | -0.217109 | 2 | 2 | 1 | 1 | 1 | 0 | Arginase-1 | HYDROLASE | ARGI1_HUMAN | NONE | Defects in ARG1 are the cause of argininemia [MIM:207800]; also known as hyperargininemia. Argininemia is a rare autosomal recessive disorder of the urea cycle. Arginine is elevated in the blood and cerebrospinal fluid, and periodic hyperammonemia occurs. Clinical manifestations include developmental delay, seizures, mental retardation, hypotonia, ataxia, progressive spastic quadriplegia. | NONE |  |
| 3d2e_v | 6 | 2.492 | 0.4153 | -1.11033 | 0 | 4 | 2 | 0 | 0 | 0 | Heat shock protein homolog SSE1 | NONE | P32589 | Has a calcium-dependent calmodulin-binding activity. Required for normal growth at various temperatures. | NONE | NONE |  |
| 1h0c_v | 7 | 2.905 | 0.415 | -0.425318 | 2 | 2 | 1 | 0 | 1 | 1 | Serine--pyruvate aminotransferase | TRANSFERASE | SPYA_HUMAN | NONE | Defects in AGXT are the cause of primary hyperoxaluria type I (PH1) [MIM:259900]; also known as oxalosis I. PH1 is a rare autosomal recessive inborn error of glyoxylate metabolism characterized by increased excretion of oxalate and glycolate, and the progressive accumulation of insoluble calcium oxalate in the kidney and urinary tract. | Hyperoxaluria |  |
| 1l9n_v | 8 | 3.319 | 0.4148 | 0.0493151 | 3 | 4 | 1 | 0 | 0 | 0 | Protein-glutamine gamma-glutamyltransferase E | TRANSFERASE | TGM3_HUMAN | Catalyzes the cross-linking of proteins and the conjugation of polyamines to proteins. It is responsible for the later stages of cell envelope formation in the epidermis and the hair follicle. | NONE | NONE |  |
| 1kms_v | 7 | 2.903 | 0.4147 | -0.190576 | 2 | 2 | 3 | 0 | 0 | 0 | Dihydrofolate reductase | OXIDOREDUCTASE | DYR_HUMAN | NONE | NONE | NONE |  |
| 1svh_v | 7 | 2.902 | 0.4146 | -0.0148989 | 4 | 1 | 1 | 1 | 0 | 0 | cAMP-dependent protein kinase, alpha-catalytic subunit | NONE | P00517 | Involved in protein serine/threonine kinase activity | NONE | NONE |  |
| 2gv7_v | 6 | 2.475 | 0.4124 | -0.529468 | 1 | 2 | 1 | 2 | 0 | 0 | Suppressor of tumorigenicity protein 14 | HYDROLASE | ST14_HUMAN | Degrades extracellular matrix. Proposed to play a role in breast cancer invasion and metastasis. Exhibits trypsin-like activity as defined by cleavage of synthetic substrates with Arg or Lys as the P1 site. | Defects in ST14 are a cause of ichthyosis autosomal recessive with hypotrichosis (ARIH) [MIM:610765]. ARIH is a skin disorder characterized by congenital ichthyosis associated with the presence of less than the normal amount of hair. | Cancer;Solid tumor |  |
| 1s0z_v | 10 | 4.103 | 0.4103 | 1.57279 | 7 | 1 | 2 | 0 | 0 | 0 | Vitamin D3 receptor | GENE REGULATION | VDR_HUMAN | Nuclear hormone receptor. Transcription factor that mediates the action of vitamin D3 by controlling the expression of hormone sensitive genes. Plays a central role in calcium homeostasis. | Defects in VDR are the cause of type IIA rickets [MIM:277440]; also known as hypocalcemic vitamin D-resistant rickets (HVDRR). HVDRR is most frequently an autosomal recessive disorder characterized by severe rickets, hypocalcemia and secondary hyperparathyroidism. | Pagets bone disease;Melanoma;Hyperthyroidism;Aging;Skin tumor;Acne;Breast tumor;Neurodegenerative disease;Acute myelogenous leukemia;Hepatocellular carcinoma;Prostate tumor;Pancreas tumor;Alzheimers disease;Multiple sclerosis;Psoriasis;Colon tumor;Cancer;Renal disease;Leukemia;Hyperparathyroidism;Sarcoidosis;Carcinoma;Insulin dependent diabetes;Transplant rejection;Osteoporosis |  |
| 1gzu_v | 7 | 2.869 | 0.4099 | -0.414407 | 1 | 4 | 1 | 0 | 1 | 0 | Nicotinamide mononucleotide adenylyltransferase 1 | NONE | Q9HAN9 | Coenzyme transport and metabolism | NONE | NONE |  |
| 1x6v_v | 7 | 2.852 | 0.4075 | -0.686347 | 0 | 5 | 1 | 0 | 1 | 0 | Bifunctional 3-phosphoadenosine 5-phosphosulfate synthetase 1 | NONE | O43252 | Involved in ATP binding | NONE | NONE |  |
| 1hs6_v | 9 | 3.649 | 0.4054 | 1.37317 | 3 | 2 | 1 | 2 | 1 | 0 | Leukotriene A-4 hydrolase | HYDROLASE | LKHA4_HUMAN | Hydrolyzes an epoxide moiety of leukotriene A4 (LTA-4) to form leukotriene B4 (LTB-4). The enzyme also has some peptidase activity. | NONE | Inflammation;Cancer;Acute myelogenous leukemia;Myocardial infarction;Non-small-cell lung cancer;Solid tumor |  |
| 1onq_v | 6 | 2.422 | 0.4036 | -0.679974 | 3 | 2 | 0 | 0 | 1 | 0 | T-cell surface glycoprotein CD1a | IMMUNE SYSTEM | CD1A_HUMAN | Antigen-presenting protein that binds self and non-self lipid and glycolipid antigens and presents them to T-cell receptors on natural killer T-cells. | NONE | NONE |  |
| 1zpb_v | 8 | 3.211 | 0.4014 | 0.693002 | 3 | 3 | 1 | 1 | 0 | 0 | Coagulation factor XI | HYDROLASE | FA11_HUMAN | Factor XI triggers the middle phase of the intrinsic pathway of blood coagulation by activating factor IX. | Defects in F11 are the cause of F11 deficiency [MIM:264900]; also called plasma thromboplastin antecedent deficiency or Rosenthal syndrome. It is a blood coagulation abnormality occurring in high frequency in Ashkenazi jews. F11- deficient patients are prone to excessive bleeding after haemostatic challenge. | Thrombosis |  |
| 1kjr_v | 7 | 2.799 | 0.3998 | 0.269632 | 2 | 2 | 2 | 0 | 0 | 1 | Galectin-3 | SUGAR BINDING PROTEIN | LEG3_HUMAN | Galactose-specific lectin which binds IgE. May mediate with the alpha-3, beta-1 integrin the stimulation by CSPG4 of endothelial cells migration. Together with DMBT1, required for terminal differentiation of columnar epithelial cells during early embryoge | NONE | Colorectal tumor;Chronic lymphocytic leukemia;Cancer;Prostate tumor;Pancreas tumor;Metastasis;Multiple myeloma |  |
| 1jk7_v | 9 | 3.596 | 0.3996 | 1.16106 | 6 | 1 | 1 | 0 | 1 | 0 | Serine/threonine-protein phosphatase PP1-gamma catalytic subunit | NONE | P36873 | Signal transduction mechanisms | NONE | NONE |  |
| 1gkd_v | 7 | 2.791 | 0.3988 | -0.616491 | 1 | 4 | 2 | 0 | 0 | 0 | Matrix metalloproteinase-9 | HYDROLASE | MMP9_HUMAN | May play an essential role in local proteolysis of the extracellular matrix and in leukocyte migration. Could play a role in bone osteoclastic resorption. Cleaves KiSS1 at a Gly- | -Leu bond. | Defects in MMP9 may be a cause of susceptibility to lumbar disk herniation (LDH) [MIM:603932]. LDH is the predominant cause of low-back pain and unilateral leg pain. | NONE |
| 1egc_v | 7 | 2.776 | 0.3966 | -0.199212 | 3 | 2 | 2 | 0 | 0 | 0 | Medium-chain specific acyl-CoA dehydrogenase, mitochondrial | ELECTRON TRANSFER | ACADM_HUMAN | This enzyme is specific for acyl chain lengths of 4 to 16. | Defects in ACADM are the cause of medium-chain acyl-CoA dehydrogenase deficiency (MCAD deficiency) [MIM:201450]. It is an autosomal recessive disease which causes fasting hypoglycemia, hepatic dysfunction, and encephalopathy, often resulting in death in infancy. The disease frequency is one in 13000. | NONE |  |
| 1mt6_v | 7 | 2.775 | 0.3964 | -0.43558 | 2 | 2 | 2 | 0 | 1 | 0 | Histone-lysine N-methyltransferase SETD7 | TRANSFERASE | SETD7_HUMAN | Histone methyltransferase that specifically monomethylates Lys-4 of histone H3. H3 Lys-4 methylation represents a specific tag for epigenetic transcriptional activation. Plays a central role in the transcriptional activation of genes such as collagena | NONE | NONE |  |
| 1c9h_v | 9 | 3.543 | 0.3937 | 2.14309 | 3 | 3 | 3 | 0 | 0 | 0 | Peptidyl-prolyl cis-trans isomerase FKBP1B | IMMUNE SYSTEM | FKB1B_HUMAN | Associates with the ryanodine receptor (RYR-2) in cardiac muscle sarcoplasmic reticulum and may play a unique physiological role in excitation-contraction coupling in cardiac muscle. There are four molecules of FKBP12.6 per heart muscle RYR. Has the poten | NONE | NONE |  |
| 1ice_v | 10 | 3.907 | 0.3907 | 1.66141 | 4 | 3 | 1 | 1 | 1 | 0 | Caspase-1 | CYTOKINE | CASP1_HUMAN | Thiol protease that cleaves IL-1 beta between an Asp and an Ala, releasing the mature cytokine which is involved in a variety of inflammatory processes. Important for defense against pathogens. Cleaves and activates sterol regulatory element binding prote | NONE | Psoriasis;Septic shock;Alzheimers disease;Rheumatoid arthritis;Sepsis;Neurological disease;Osteoarthritis;Inflammation;Diabetes mellitus;Metastasis;Cardiovascular disease |  |
| 1fo3_v | 7 | 2.717 | 0.3881 | -0.685716 | 0 | 4 | 3 | 0 | 0 | 0 | Endoplasmic reticulum mannosyl-oligosaccharide 1,2-alpha-mannosidase | NONE | Q9UKM7 | Involved in mannosyl-oligosaccharide 1,2-alpha-mannosidase activity | NONE | NONE |  |
| 1mlw_v | 7 | 2.699 | 0.3855 | -0.957341 | 1 | 4 | 2 | 0 | 0 | 0 | Tryptophan 5-hydroxylase 1 | OXIDOREDUCTASE | TPH1_HUMAN | NONE | NONE | NONE |  |
| 1xap_v | 9 | 3.467 | 0.3852 | 0.351054 | 7 | 1 | 0 | 0 | 1 | 0 | Retinoic acid receptor beta | TRANSCRIPTION | RARB_HUMAN | This is a receptor for retinoic acid. This metabolite has profound effects on vertebrate development. Retinoic acid is a morphogen and is a powerful teratogen. This receptor controls cell function by directly regulating gene expression. | NONE | Psoriasis;Acne;Cancer |  |
| 1liu_v | 9 | 3.452 | 0.3836 | 0.546622 | 0 | 6 | 1 | 0 | 2 | 0 | Pyruvate kinase isozymes R/L | TRANSFERASE | KPYR_HUMAN | NONE | Defects in PKLR are the cause of pyruvate kinase hyperactivity [MIM:102900]; also known as high red cell ATP syndrome. This autosomal dominant phenotype is characterized by increase of red blood cell ATP.;Defects in PKLR are a cause of chronic nonspherocytic hemolytic anemia (CNSHA) [MIM:266200]; also called hereditary nonspherocytic hemolytic anemia (HNSHA). | NONE |  |
| 1yq7_v | 8 | 3.036 | 0.3795 | -0.517571 | 1 | 4 | 1 | 0 | 2 | 0 | Farnesyl pyrophosphate synthetase | TRANSFERASE | FPPS_HUMAN | Key enzyme in isoprenoid biosynthesis which catalyzes the formation of farnesyl diphosphate (FPP), a precursor for several classes of essential metabolites including sterols, dolichols, carotenoids, and ubiquinones. FPP also serves as substrate for protei | NONE | NONE |  |
| 1ljr_v | 7 | 2.636 | 0.3765 | -1.02712 | 1 | 3 | 1 | 1 | 1 | 0 | Glutathione S-transferase theta-2 | TRANSFERASE | GSTT2_HUMAN | Conjugation of reduced glutathione to a wide number of exogenous and endogenous hydrophobic electrophiles. Has a sulfatase activity. | NONE | NONE |  |
| 1uzf_v | 7 | 2.631 | 0.3758 | -1.05297 | 2 | 3 | 0 | 0 | 2 | 0 | Angiotensin-converting enzyme | METALLOPROTEASE | ACE_HUMAN | Converts angiotensin I to angiotensin II by release of the terminal His-Leu, this results in an increase of the vasoconstrictor activity of angiotensin. Also able to inactivate bradykinin, a potent vasodilator. Has also a glycosidase activity which releas | Genetic variations in ACE may be a cause of susceptibility to ischemic stroke [MIM:601367]; also known as cerebrovascular accident or cerebral infarction. A stroke is an acute neurologic event leading to death of neural tissue of the brain and resulting in loss of motor, sensory and/or cognitive function. Ischemic strokes, resulting from vascular occlusion, is considered to be a highly complex disease consisting of a group of heterogeneous disorders with multiple genetic and environmental risk factors.;Defects in ACE are a cause of renal tubular dysgenesis (RTD) [MIM:267430]. RTD is an autosomal recessive severe disorder of renal tubular development characterized by persistent fetal anuria and perinatal death, probably due to pulmonary hypoplasia from early-onset oligohydramnios (the Potter phenotype).;Genetic variations in ACE can influence susceptibility to diabetic nephropathy [MIM:603933]. Diabetic nephropathy is a kidney disease and resultant kidney function impairment due to the long standing effects of diabetes on the microvasculature (glomerulus) of the kidney. Features include increased urine protein and declining kidney function. | NONE |  |
| 1tx4_v | 6 | 2.24 | 0.3733 | -1.44783 | 0 | 4 | 2 | 0 | 0 | 0 | Rho GTPase-activating protein 1 | NONE | Q07960 | GTPase activator for the Rho, Rac and Cdc42 proteins, converting them to the putatively inactive GDP-bound state. Cdc42 seems to be the preferred substrate. | NONE | NONE |  |
| 1hrk_v | 8 | 2.985 | 0.3731 | -0.267269 | 5 | 1 | 2 | 0 | 0 | 0 | Ferrochelatase, mitochondrial | NONE | P22830 | Coenzyme transport and metabolism | NONE | NONE |  |
| 2rjp_v | 6 | 2.223 | 0.3705 | -1.0365 | 3 | 1 | 0 | 0 | 0 | 2 | A disintegrin and metalloproteinase with thrombospondin motifs 4 | HYDROLASE | ATS4_HUMAN | Cleaves aggrecan, a cartilage proteoglycan, and may be involved in its turnover. May play an important role in the destruction of aggrecan in arthritic diseases. Could also be a critical factor in the exacerbation of neurodegeneration in Alzheimer disease | NONE | NONE |  |
| 1v4s_v | 8 | 2.955 | 0.3694 | -0.326868 | 3 | 3 | 2 | 0 | 0 | 0 | Glucokinase | TRANSFERASE | HXK4_HUMAN | Catalyzes the initial step in utilization of glucose by the beta-cell and liver at physiological glucose concentration. Glucokinase has a high Km for glucose, and so it is effective only when glucose is abundant. The role of GCK is to provide G6P for the | Defects in GCK are the cause of maturity onset diabetes of the young type 2 (MODY2) [MIM:125851]; also shortened MODY-2. MODY [MIM:606391] is a form of diabetes mellitus characterized by autosomal dominant transmission and early age of onset. Mutations in GCK result in mild chronic hyperglycemia due to reduced pancreatic beta cell responsiveness to glucose, decreased net accumulation of hepatic glycogen and increased hepatic gluconeogenesis following meals.;Defects in GCK are the cause of familial hyperinsulinemic hypoglycemia type 3 (HHF3) [MIM:602485]. HHF is the most common cause of persistent hypoglycemia in infancy. Unless early and aggressive intervention is undertaken, brain damage from recurrent episodes of hypoglycemia may occur. | Diabetes mellitus;Non-insulin dependent diabetes |  |
| 1gbn_v | 8 | 2.948 | 0.3684 | -0.333025 | 2 | 4 | 0 | 0 | 2 | 0 | Ornithine aminotransferase, mitochondrial | TRANSFERASE | OAT_HUMAN | NONE | Defects in OAT are the cause of hyperornithinemia with gyrate atrophy of choroid and retina (HOGA) [MIM:258870]. HOGA is a slowly progressive blinding autosomal recessive disorder. | Cancer |  |
| 1uym_v | 8 | 2.943 | 0.3678 | -0.0477913 | 5 | 1 | 1 | 0 | 0 | 1 | Heat shock protein HSP 90-beta | NONE | P08238 | Posttranslational modification, protein turnover, chaperones | NONE | NONE |  |
| 2gpq_v | 8 | 2.928 | 0.366 | 0.248937 | 3 | 2 | 2 | 1 | 0 | 0 | Eukaryotic translation initiation factor 4E | NONE | P06730 | Involved in RNA binding | NONE | NONE |  |
| 1nn0_v | 8 | 2.927 | 0.3659 | -0.576676 | 1 | 5 | 1 | 0 | 1 | 0 | Thymidylate kinase | TRANSFERASE | KTHY_HUMAN | Catalyzes the conversion of dTMP to dTDP. | NONE | NONE |  |
| 1lt8_v | 8 | 2.926 | 0.3658 | -0.246278 | 2 | 5 | 0 | 1 | 0 | 0 | Betaine--homocysteine S-methyltransferase 1 | NONE | Q93088 | Amino acid transport and metabolism | NONE | Used for protein synthesis including the formation of SAMe, L-homocysteine, L-cysteine, taurine, and sulfate. |  |
| 2fs9_v | 8 | 2.926 | 0.3657 | 0.243942 | 2 | 3 | 2 | 1 | 0 | 0 | Tryptase beta-2 | HYDROLASE | TRYB2_HUMAN | Tryptase is the major neutral protease present in mast cells and is secreted upon the coupled activation-degranulation response of this cell type. Has an immunoprotective role during bacterial infection. Required to efficiently combat K.pneumoniae infecti | NONE | NONE |  |
| 1i7b_v | 8 | 2.926 | 0.3657 | -0.258023 | 2 | 3 | 3 | 0 | 0 | 0 | S-adenosylmethionine decarboxylase proenzyme | LYASE | DCAM_HUMAN | NONE | NONE | Colorectal tumor;Non-Hodgkin lymphoma;Neoplasm;Cancer;Trypanosomiasis;Pneumocystis carinii infection;Bacterial infection |  |
| 1yvl_v | 8 | 2.923 | 0.3653 | -0.20781 | 1 | 4 | 2 | 1 | 0 | 0 | Signal transducer and activator of transcription 1-alpha/beta | SIGNALING PROTEIN | STAT1_HUMAN | Signal transducer and activator of transcription that mediates signaling by interferons (IFNs). Following type I IFN (IFN-alpha and IFN-beta) binding to cell surface receptors, Jak kinases (TYK2 and JAK1) are activated, leading to tyrosine phosphorylation | Defects in STAT1 are the cause of STAT1 deficiency [MIM:600555]. Patients generally suffer from mycobacterial or viral diseases. In the case of complete deficiency, patients can die of viral disease.;Defects in STAT1 are a cause of mendelian susceptibility to mycobacterial disease (MSMD) [MIM:209950]; also known as familial disseminated atypical mycobacterial infection. This rare condition confers predisposition to illness caused by moderately virulent mycobacterial species, such as Bacillus Calmette-Guerin (BCG) vaccine and environmental non-tuberculous mycobacteria, and by the more virulent Mycobacterium tuberculosis. Other microorganisms rarely cause severe clinical disease in individuals with susceptibility to mycobacterial infections, with the exception of Salmonella which infects less than 50% of these individuals. The pathogenic mechanism underlying MSMD is the impairment of interferon-gamma mediated immunity whose severity determines the clinical outcome. Some patients die of overwhelming mycobacterial disease with lepromatous-like lesions in early childhood, whereas others develop, later in life, disseminated but curable infections with tuberculoid granulomas. MSMD is a genetically heterogeneous disease with autosomal recessive, autosomal dominant or X-linked inheritance. | Respiratory disease;Asthma |  |
| 1wb0_v | 8 | 2.903 | 0.3629 | -0.196782 | 2 | 3 | 2 | 1 | 0 | 0 | Chitotriosidase-1 | NONE | Q13231 | Involved in chitinase activity | NONE | NONE |  |
| 1zdz_v | 7 | 2.532 | 0.3617 | -1.17355 | 0 | 3 | 2 | 2 | 0 | 0 | Spermidine synthase | NONE | P19623 | Amino acid transport and metabolism | NONE | S-Adenosylmethionine (SAMe) is used as a drug in Europe for the treatment of depression, liver disorders, fibromyalgia, and osteoarthritis. It has also been introduced into the United States market as a dietary supplement for the support of bone and joint health, as well as mood and emotional well being. |  |
| 1h9o_v | 8 | 2.884 | 0.3606 | 0.166652 | 3 | 2 | 2 | 0 | 1 | 0 | Phosphatidylinositol 3-kinase regulatory subunit alpha | COMPLEX (PHOSPHOTRANSFERASE/RECEPTOR) | P85A_HUMAN | Binds to activated (phosphorylated) protein-Tyr kinases, through its SH2 domain, and acts as an adapter, mediating the association of the p110 catalytic unit to the plasma membrane. Necessary for the insulin-stimulated increase in glucose uptake and glyco | Defects in PIK3R1 are a cause of severe insulin resistance. | NONE |  |
| 1ln3_v | 11 | 3.936 | 0.3578 | 1.23531 | 9 | 0 | 0 | 1 | 1 | 0 | Phosphatidylcholine transfer protein | NONE | Q9UKL6 | Involved in phosphatidylcholine transmembrane transporter activity | NONE | NONE |  |
| 1h9u_v | 10 | 3.569 | 0.3569 | 0.256438 | 8 | 1 | 0 | 0 | 1 | 0 | Retinoic acid receptor RXR-beta | NUCLEAR RECEPTOR | RXRB_HUMAN | Nuclear hormone receptor. Involved in the retinoic acid response pathway. Binds 9-cis retinoic acid (9C-RA). | NONE | NONE |  |
| 1r55_v | 10 | 3.535 | 0.3535 | 0.582978 | 2 | 4 | 4 | 0 | 0 | 0 | ADAM 33 | HYDROLASE | ADA33_HUMAN | NONE | Defects in ADAM33 may be a cause of susceptibility to asthma. | NONE |  |
| 1jbq_v | 7 | 2.449 | 0.3498 | -1.22837 | 2 | 4 | 0 | 0 | 1 | 0 | Cystathionine beta-synthase | NONE | P35520 | Amino acid transport and metabolism | NONE | For nutritional supplementation and for treating dietary shortage or imbalance. |  |
| 1q5h_v | 10 | 3.441 | 0.3441 | 0.283031 | 1 | 6 | 2 | 0 | 1 | 0 | Deoxyuridine 5-triphosphate nucleotidohydrolase, mitochondrial | HYDROLASE | DUT_HUMAN | This enzyme is involved in nucleotide metabolism: it produces dUMP, the immediate precursor of thymidine nucleotides and it decreases the intracellular concentration of dUTP so that uracil cannot be incorporated into DNA. | NONE | NONE |  |
| 1oth_v | 8 | 2.742 | 0.3428 | -0.980063 | 1 | 4 | 1 | 1 | 1 | 0 | Ornithine carbamoyltransferase, mitochondrial | NONE | P00480 | Amino acid transport and metabolism | NONE | Used for nutritional supplementation, also for treating dietary shortage or imbalance. It has been claimed that ornithine improves athletic performance, has anabolic effects, has wound-healing effects, and is immuno-enhancing. |  |
| 1nfb_v | 9 | 3.036 | 0.3373 | -0.429758 | 0 | 7 | 1 | 0 | 0 | 1 | Inosine-5-monophosphate dehydrogenase 2 | OXIDOREDUCTASE | IMDH2_HUMAN | Rate limiting enzyme in the de novo synthesis of guanine nucleotides and therefore is involved in the regulation of cell growth. It may also have a role in the development of malignancy and the growth progression of some tumors. | NONE | Autoimmune disease;Transplant rejection |  |
| 2auh_v | 11 | 3.686 | 0.3351 | 0.753963 | 3 | 4 | 1 | 1 | 2 | 0 | Insulin receptor | TRANSFERASE/SIGNALING PROTEIN | INSR_HUMAN | This receptor binds insulin and has a tyrosine-protein kinase activity. Isoform Short has a higher affinity for insulin. Mediates the metabolic functions of insulin. Binding to insulin stimulates association of the receptor with downstream mediators inclu | Defects in INSR are the cause of insulin resistance (Ins resistance) [MIM:125853].;Defects in INSR are the cause of Rabson-Mendenhall syndrome [MIM:262190]; also known as Mendenhall syndrome. It is a severe insulin resistance syndrome characterized by insulin- resistant diabetes mellitus with pineal hyperplasia and somatic abnormalities. Typical features include coarse, senile-appearing facies, dental and skin abnormalities, abdominal distension, and phallic enlargement. Inheritance is autosomal recessive.;Defects in INSR are the cause of leprechaunism [MIM:246200]; also known as Donohue syndrome. Leprechaunism represents the most severe form of insulin resistance syndrome, characterized by intrauterine and postnatal growth retardation and death in early infancy. Inheritance is autosomal recessive.;Defects in INSR may be associated with noninsulin- dependent diabetes mellitus (NIDDM) [MIM:125853]; also known as diabetes mellitus type 2.;Defects in INSR are the cause of familial hyperinsulinemic hypoglycemia 5 (HHF5) [MIM:609968]. Familial hyperinsulinemic hypoglycemia [MIM:256450], also referred to as congenital hyperinsulinism, nesidioblastosis, or persistent hyperinsulinemic hypoglycemia of infancy (PPHI), is the most common cause of persistent hypoglycemia in infancy and is due to defective negative feedback regulation of insulin secretion by low glucose levels.;Defects in INSR are the cause of insulin-resistant diabetes mellitus with acanthosis nigricans type A (IRAN type A) [MIM:610549]. This syndrome is characterized by the association of severe insulin resistance (manifested by marked hyperinsulinemia and a failure to respond to exogenous insulin) with the skin lesion acanthosis nigricans and ovarian hyperandrogenism in adolescent female subjects. Women frequently present with hirsutism, acne, amenorrhea or oligomenorrhea, and virilization. This syndrome is different from the type B that has been demonstrated to be secondary to the presence of circulating autoantibodies against the insulin receptor. | Insulin dependent diabetes;Cancer;Non-insulin dependent diabetes;Infection;Parkinsons disease;Hypertension;Diabetes mellitus;Hyperinsulinemia |  |
| 2yxj_v | 9 | 2.996 | 0.3328 | 0.0724418 | 6 | 1 | 1 | 1 | 0 | 0 | Bcl-2-like protein 1 | NONE | Q07817 | Potent inhibitor of cell death. Inhibits activation of caspases. Appears to regulate cell death by blocking the voltage-dependent anion channel (VDAC) by binding to it and preventing the release of the caspase activator, CYC1, from the mitochondrial membrane. Also acts as a regulator of G2 checkpoint and progression to cytokinesis during mitosis. | NONE | NONE |  |
| 1x0n_v | 9 | 2.972 | 0.3303 | -0.505896 | 5 | 1 | 2 | 0 | 1 | 0 | Growth factor receptor-bound protein 2 | PEPTIDE BINDING PROTEIN | GRB2_HUMAN | Adapter protein that provides a critical link between cell surface growth factor receptors and the Ras signaling pathway.;Isoform GRB3-3 does not bind to phosphorylated epidermal growth factor receptor (EGFR) but inhibits EGF-induced transactivation of a RAS-responsive element. Isoform GRB3-3 acts as a dominant negative protein over GRB2 and by suppressing proliferative sign | NONE | Leukemia;Breast tumor;Cancer |  |
| 1fro_v | 9 | 2.956 | 0.3284 | -0.286396 | 3 | 3 | 1 | 1 | 1 | 0 | Lactoylglutathione lyase | LACTOYLGLUTATHIONE LYASE | LGUL_HUMAN | Catalyzes the conversion of hemimercaptal, formed from methylglyoxal and glutathione, to S-lactoylglutathione. | NONE | Cancer |  |
| 1q91_v | 9 | 2.937 | 0.3263 | -0.740748 | 1 | 6 | 1 | 0 | 1 | 0 | 5(3)-deoxyribonucleotidase, mitochondrial | NONE | Q9NPB1 | Involved in nucleotidase activity | NONE | NONE |  |
| 1a4r_v | 8 | 2.607 | 0.3259 | -1.36964 | 0 | 5 | 2 | 0 | 1 | 0 | Cell division control protein 42 homolog | HYDROLASE | CDC42_HUMAN | Plasma membrane-associated small GTPase which cycles between an active GTP-bound and an inactive GDP-bound state. In active state binds to a variety of effector proteins to regulate cellular responses. Involved in epithelial cell polarization processes. C | NONE | NONE |  |
| 1tdi_v | 9 | 2.93 | 0.3255 | -0.605928 | 1 | 5 | 1 | 1 | 1 | 0 | Glutathione S-transferase A3 | NONE | Q16772 | Involved in glutathione transferase activity | NONE | For nutritional supplementation, also for treating dietary shortage or imbalance |  |
| 1nb9_v | 11 | 3.562 | 0.3238 | 0.612348 | 0 | 7 | 2 | 0 | 2 | 0 | Riboflavin kinase | NONE | Q969G6 | Coenzyme transport and metabolism | NONE | For the treatment of ariboflavinosis (vitamin B2 deficiency). |  |
| 1r6u_v | 9 | 2.898 | 0.322 | -0.520484 | 1 | 4 | 2 | 1 | 1 | 0 | Tryptophanyl-tRNA synthetase, cytoplasmic | NONE | P23381 | Translation, ribosomal structure and biogenesis | NONE | Tryptophan may be useful in increasing serotonin production, promoting healthy sleep, managing depression by enhancing mental and emotional well-being, managing pain tolerance, and managing weight. |  |
| 1hfc_v | 8 | 2.563 | 0.3204 | -1.1662 | 1 | 3 | 4 | 0 | 0 | 0 | Interstitial collagenase | NONE | P03956 | Cleaves collagens of types I, II, and III at one site in the helical domain. Also cleaves collagens of types VII and X (PubMed:2557822, PubMed:2153297, PubMed:1645757). In case of HIV infection, interacts and cleaves the secreted viral Tat protein, leading to a decrease in neuronal Tat's mediated neurotoxicity (PubMed:16807369). | NONE | NONE |  |
| 1xcx_v | 9 | 2.874 | 0.3193 | -0.146455 | 1 | 3 | 4 | 1 | 0 | 0 | Pancreatic alpha-amylase | HYDROLASE | AMYP_HUMAN | NONE | NONE | Bacillus anthracis infection |  |
| 1cm0_v | 9 | 2.872 | 0.3191 | -0.594157 | 1 | 6 | 2 | 0 | 0 | 0 | Histone acetyltransferase PCAF | NONE | Q92831 | Involved in N-acetyltransferase activity | NONE | NONE |  |
| 1pbk_v | 11 | 3.506 | 0.3188 | 1.62472 | 3 | 5 | 3 | 0 | 0 | 0 | FK506-binding protein 3 | ISOMERASE | FKBP3_HUMAN | FK506- and rapamycin-binding proteins (FKBPs) constitute a family of receptors for the two immunosuppressants which inhibit T-cell proliferation by arresting two distinct cytoplasmic signal transmission pathways. PPIases accelerate the folding of proteins | NONE | NONE |  |
| 1yj6_v | 9 | 2.828 | 0.3143 | -0.646844 | 1 | 3 | 2 | 1 | 2 | 0 | Glutathione S-transferase Mu 1 | TRANSFERASE | GSTM1_HUMAN | Conjugation of reduced glutathione to a wide number of exogenous and endogenous hydrophobic electrophiles. | NONE | NONE |  |
| 1xw5_v | 9 | 2.823 | 0.3136 | -0.725358 | 1 | 5 | 1 | 1 | 1 | 0 | Glutathione S-transferase Mu 2 | TRANSFERASE | GSTM2_HUMAN | Conjugation of reduced glutathione to a wide number of exogenous and endogenous hydrophobic electrophiles. | NONE | NONE |  |
| 1p4r_v | 9 | 2.819 | 0.3132 | -0.859544 | 0 | 7 | 1 | 0 | 1 | 0 | Bifunctional purine biosynthesis protein PURH | TRANSFERASE, HYDROLASE | PUR9_HUMAN | NONE | Defects in ATIC are the cause of AICA-ribosuria [MIM:608688]; also known as AICA-ribosiduria. AICA-ribosuria is a neurologically devastating inborn error of purine biosynthesis. AICA-ribosuria patients excrete massive amounts of AICA-riboside in the urine and accumulate AICA-ribotide and its derivatives in erythrocytes and fibroblasts. AICA-ribosuria causes profound mental retardation, epilepsy, dysmorphic features and congenital blindness. | NONE |  |
| 1tu4_v | 10 | 3.126 | 0.3126 | -0.211467 | 0 | 8 | 2 | 0 | 0 | 0 | Ras-related protein Rab-5A | PROTEIN TRANSPORT | RAB5A_HUMAN | Required for the fusion of plasma membranes and early endosomes. | NONE | NONE |  |
| 1kpe_v | 8 | 2.49 | 0.3112 | -1.4805 | 0 | 3 | 5 | 0 | 0 | 0 | Histidine triad nucleotide-binding protein 1 | PROTEIN KINASE INHIBITOR | HINT1_HUMAN | Hydrolyzes adenosine 5-monophosphoramidate substrates such as AMP-morpholidate, AMP-N-alanine methyl ester, AMP-alpha- acetyl lysine methyl ester and AMP-NH2 (By similarity). | NONE | NONE |  |
| 1o7a_v | 8 | 2.48 | 0.3099 | -1.30662 | 1 | 4 | 3 | 0 | 0 | 0 | Beta-hexosaminidase beta chain | NONE | P07686 | Carbohydrate transport and metabolism | NONE | NONE |  |
| 1ua2_v | 8 | 2.425 | 0.3031 | -1.86712 | 1 | 3 | 1 | 0 | 3 | 0 | Cell division protein kinase 7 | NONE | P50613 | Involved in protein kinase activity | NONE | NONE |  |
| 1g1t_v | 9 | 2.671 | 0.2968 | -1.23369 | 0 | 7 | 1 | 0 | 1 | 0 | E-selectin | IMMUNE SYSTEM, MEMBRANE PROTEIN | LYAM2_HUMAN | Cell-surface glycoprotein having a role in immunoadhesion. Mediates in the adhesion of blood neutrophils in cytokine-activated endothelium through interaction with PSGL1/SELPLG. May have a role in capillary morphogenesis. | NONE | Asthma;Lung injury;Atopic dermatitis;Cerebrovascular ischemia;Shock;Ischemic heart disease;Dermatitis;Respiratory disease;Inflammatory bowel disease;Inflammation;Chronic obstructive pulmonary disease;Myocardial infarction;Injury;Psoriasis;Reperfusion injury;Sepsis;Skin burns;Respiratory distress syndrome;Transplant rejection;Hypertension |  |
| 1g55_v | 10 | 2.953 | 0.2953 | -0.450369 | 1 | 6 | 2 | 1 | 0 | 0 | tRNA (cytosine-5-)-methyltransferase | NONE | O14717 | Specifically methylates cytosine 38 in the anticodon loop of tRNA(Asp). | NONE | NONE |  |
| 1f12_v | 10 | 2.943 | 0.2943 | -0.223512 | 1 | 7 | 1 | 0 | 1 | 0 | Hydroxyacyl-coenzyme A dehydrogenase, mitochondrial | NONE | Q16836 | Lipid transport and metabolism | NONE | Some evidence suggests that NADH might be useful in treating Parkinsons disease, chronic fatigue syndrome, Alzheimers disease and cardiovascular disease. |  |
| 1z57_v | 7 | 2.051 | 0.293 | -3.14356 | 0 | 5 | 1 | 1 | 0 | 0 | Dual specificity protein kinase CLK1 | TRANSFERASE | CLK1_HUMAN | Phosphorylates serine- and arginine-rich (SR) proteins of the spliceosomal complex may be a constituent of a network of regulatory mechanisms that enable SR proteins to control RNA splicing. Phosphorylates serines, threonines and tyrosines (By similarity) | NONE | Toxicity;Metabolic disorder;Neurodegenerative disease;Cancer;Cardiovascular disease |  |
| 1ivh_v | 12 | 3.508 | 0.2923 | 1.28121 | 2 | 3 | 5 | 0 | 2 | 0 | Isovaleryl-CoA dehydrogenase, mitochondrial | NONE | P26440 | Lipid transport and metabolism | NONE | NONE |  |
| 1hmp_v | 9 | 2.588 | 0.2876 | -1.51141 | 0 | 6 | 3 | 0 | 0 | 0 | Hypoxanthine-guanine phosphoribosyltransferase | NONE | P00492 | Nucleotide transport and metabolism | NONE | For remission induction and remission consolidation treatment of acute nonlymphocytic leukemias. |  |
| 1z6y_v | 9 | 2.491 | 0.2768 | -1.70508 | 0 | 5 | 3 | 0 | 1 | 0 | ADP-ribosylation factor-like protein 5A | NONE | Q9Y689 | Involved in GTP binding | NONE | NONE |  |
| 1xmm_v | 11 | 2.956 | 0.2687 | -0.590124 | 1 | 5 | 3 | 1 | 1 | 0 | Scavenger mRNA-decapping enzyme DcpS | NONE | Q96C86 | Involved in mRNA degradation | NONE | NONE |  |

**Table S5. DEHP targets from SwissTargetPrediction.**

| **Target** | **Common name** | **Uniprot ID** | **ChEMBL ID** | **Target Class** | **Probability*** | **Known actives (3D/2D)** |
| --- | --- | --- | --- | --- | --- | --- |
| Protein kinase C delta | PRKCD | Q05655 | CHEMBL2996 | Kinase | 0.115736675 | 33 / 8 |
| Protein-tyrosine phosphatase 1B | PTPN1 | P18031 | CHEMBL335 | Phosphatase | 0.115736675 | 32 / 10 |
| Protein kinase C alpha | PRKCA | P17252 | CHEMBL299 | Kinase | 0.115736675 | 37 / 9 |
| T-cell protein-tyrosine phosphatase | PTPN2 | P17706 | CHEMBL3807 | Phosphatase | 0.115736675 | 0 / 7 |
| Androgen Receptor | AR | P10275 | CHEMBL1871 | Nuclear receptor | 0.115736675 | 104 / 1 |
| Cathepsin K | CTSK | P43235 | CHEMBL268 | Protease | 0.115736675 | 410 / 0 |
| Cathepsin S | CTSS | P25774 | CHEMBL2954 | Protease | 0.115736675 | 305 / 0 |
| Cathepsin L | CTSL | P07711 | CHEMBL3837 | Protease | 0.115736675 | 247 / 0 |
| Cathepsin (B and K) | CTSB | P07858 | CHEMBL4072 | Protease | 0.115736675 | 215 / 0 |
| FK506-binding protein 1A | FKBP1A | P62942 | CHEMBL1902 | Isomerase | 0.115736675 | 148 / 0 |
| Phosphodiesterase 10A (by homology) | PDE10A | Q9Y233 | CHEMBL4409 | Phosphodiesterase | 0.115736675 | 1572 / 0 |
| Metabotropic glutamate receptor 2 | GRM2 | Q14416 | CHEMBL5137 | Family C G protein-coupled receptor | 0.115736675 | 98 / 0 |
| Cyclin-dependent kinase 4/cyclin D1 | CCND1 CDK4 | P24385 P11802 | CHEMBL1907601 | Kinase | 0.115736675 | 67 / 0 |
| Cyclin-dependent kinase 1/cyclin B1 | CDK1 CCNB1 | P06493 P14635 | CHEMBL1907602 | Other cytosolic protein | 0.115736675 | 23 / 0 |
| Cyclin-dependent kinase 2/cyclin E1 | CCNE1 CDK2 | P24864 P24941 | CHEMBL1907605 | Kinase | 0.115736675 | 91 / 0 |
| MAP kinase p38 alpha | MAPK14 | Q16539 | CHEMBL260 | Kinase | 0.115736675 | 938 / 0 |
| Translocator protein (by homology) | TSPO | P30536 | CHEMBL5742 | Membrane receptor | 0.115736675 | 640 / 0 |
| Prostanoid EP1 receptor | PTGER1 | P34995 | CHEMBL1811 | Family A G protein-coupled receptor | 0.115736675 | 70 / 0 |
| GABA-A receptor; alpha-3/beta-3/gamma-2 | GABRB3 GABRA3 GABRG2 | P28472 P34903 P18507 | CHEMBL2094120 | Ligand-gated ion channel | 0.115736675 | 324 / 0 |
| GABA-A receptor; alpha-1/beta-3/gamma-2 | GABRB3 GABRG2 GABRA1 | P28472 P18507 P14867 | CHEMBL2094121 | Ligand-gated ion channel | 0.115736675 | 271 / 0 |
| GABA-A receptor; alpha-5/beta-3/gamma-2 | GABRB3 GABRG2 GABRA5 | P28472 P18507 P31644 | CHEMBL2094122 | Ligand-gated ion channel | 0.115736675 | 300 / 0 |
| GABA-A receptor; alpha-2/beta-3/gamma-2 | GABRA2 GABRB3 GABRG2 | P47869 P28472 P18507 | CHEMBL2094130 | Ligand-gated ion channel | 0.115736675 | 274 / 0 |
| Phosphodiesterase 5A | PDE5A | O76074 | CHEMBL1827 | Phosphodiesterase | 0.115736675 | 287 / 0 |
| Apoptosis regulator Bcl-2 | BCL2 | P10415 | CHEMBL4860 | Other ion channel | 0.115736675 | 27 / 0 |
| G-protein coupled bile acid receptor 1 | GPBAR1 | Q8TDU6 | CHEMBL5409 | Family A G protein-coupled receptor | 0.115736675 | 65 / 0 |
| Protein farnesyltransferase | FNTA FNTB | P49354 P49356 | CHEMBL2094108 | Enzyme | 0.115736675 | 461 / 0 |
| Prolyl endopeptidase | PREP | P48147 | CHEMBL3202 | Protease | 0.115736675 | 140 / 0 |
| MAP kinase p38 beta | MAPK11 | Q15759 | CHEMBL3961 | Kinase | 0.115736675 | 64 / 0 |
| Angiotensin-converting enzyme (by homology) | ACE | P12821 | CHEMBL1808 | Protease | 0.115736675 | 5 / 0 |
| P2X purinoceptor 7 | P2RX7 | Q99572 | CHEMBL4805 | Ligand-gated ion channel | 0.115736675 | 480 / 0 |
| GABA-A receptor; alpha-6/beta-3/gamma-2 | GABRG2 GABRB3 GABRA6 | P18507 P28472 Q16445 | CHEMBL2095190 | Ligand-gated ion channel | 0.115736675 | 33 / 0 |
| Elongation of very long chain fatty acids protein 6 | ELOVL6 | Q9H5J4 | CHEMBL5704 | Enzyme | 0.115736675 | 28 / 0 |
| Voltage-gated potassium channel subunit Kv1.5 | KCNA5 | P22460 | CHEMBL4306 | Voltage-gated ion channel | 0.115736675 | 182 / 0 |
| Geranylgeranyl transferase type I | PGGT1B FNTA | P53609 P49354 | CHEMBL2095164 | Enzyme | 0.115736675 | 125 / 0 |
| Tumor necrosis factor receptor R1 | TNFRSF1A | P19438 | CHEMBL3378 | Membrane receptor | 0.115736675 | 17 / 0 |
| Sodium channel protein type X alpha subunit | SCN10A | Q9Y5Y9 | CHEMBL5451 | Voltage-gated ion channel | 0.115736675 | 46 / 0 |
| Carboxypeptidase B | CPB1 | P15086 | CHEMBL2552 | Protease | 0.115736675 | 3 / 0 |
| Menin | MEN1 | O00255 | CHEMBL1615381 | Unclassified protein | 0.115736675 | 7 / 0 |
| Short transient receptor potential channel 6 | TRPC6 | Q9Y210 | CHEMBL2417347 | Voltage-gated ion channel | 0.115736675 | 22 / 0 |
| Short transient receptor potential channel 3 | TRPC3 | Q13507 | CHEMBL2417348 | Voltage-gated ion channel | 0.115736675 | 20 / 0 |
| Leukocyte elastase | ELANE | P08246 | CHEMBL248 | Protease | 0.115736675 | 198 / 0 |
| Transient receptor potential cation channel subfamily V member 4 | TRPV4 | Q9HBA0 | CHEMBL3119 | Voltage-gated ion channel | 0.115736675 | 8 / 0 |
| Orexin receptor 2 | HCRTR2 | O43614 | CHEMBL4792 | Family A G protein-coupled receptor | 0.115736675 | 944 / 0 |
| Vanilloid receptor | TRPV1 | Q8NER1 | CHEMBL4794 | Voltage-gated ion channel | 0.115736675 | 381 / 0 |
| Mitogen-activated protein kinase kinase kinase 8 | MAP3K8 | P41279 | CHEMBL4899 | Kinase | 0.115736675 | 51 / 0 |
| Orexin receptor 1 | HCRTR1 | O43613 | CHEMBL5113 | Family A G protein-coupled receptor | 0.115736675 | 792 / 0 |
| Transient receptor potential cation channel subfamily A member 1 | TRPA1 | O75762 | CHEMBL6007 | Voltage-gated ion channel | 0.115736675 | 36 / 0 |
| Glucose transporter | SLC2A1 | P11166 | CHEMBL2535 | Electrochemical transporter | 0.115736675 | 24 / 0 |
| Metabotropic glutamate receptor 5 | GRM5 | P41594 | CHEMBL3227 | Family C G protein-coupled receptor | 0.115736675 | 1156 / 0 |
| ADAM17 | ADAM17 | P78536 | CHEMBL3706 | Protease | 0.115736675 | 22 / 0 |
| Chymase | CMA1 | P23946 | CHEMBL4068 | Protease | 0.115736675 | 76 / 0 |
| G protein-coupled receptor 44 | PTGDR2 | Q9Y5Y4 | CHEMBL5071 | Family A G protein-coupled receptor | 0.115736675 | 28 / 0 |
| Solute carrier family 2, facilitated glucose transporter member 3 | SLC2A3 | P11169 | CHEMBL5215 | Electrochemical transporter | 0.115736675 | 21 / 0 |
| Solute carrier family 2, facilitated glucose transporter member 2 | SLC2A2 | P11168 | CHEMBL5873 | Electrochemical transporter | 0.115736675 | 13 / 0 |
| Adenosine A1 receptor | ADORA1 | P30542 | CHEMBL226 | Family A G protein-coupled receptor | 0.115736675 | 792 / 0 |
| Adenosine A2a receptor | ADORA2A | P29274 | CHEMBL251 | Family A G protein-coupled receptor | 0.115736675 | 584 / 0 |
| Adenosine A2b receptor | ADORA2B | P29275 | CHEMBL255 | Family A G protein-coupled receptor | 0.115736675 | 124 / 0 |
| Matrix metalloproteinase 9 | MMP9 | P14780 | CHEMBL321 | Protease | 0.115736675 | 75 / 0 |
| Matrix metalloproteinase 2 | MMP2 | P08253 | CHEMBL333 | Protease | 0.115736675 | 87 / 0 |
| Matrix metalloproteinase 7 | MMP7 | P09237 | CHEMBL4073 | Protease | 0.115736675 | 4 / 0 |
| Nuclear receptor ROR-gamma | RORC | P51449 | CHEMBL1741186 | Nuclear receptor | 0.115736675 | 128 / 0 |
| Polyadenylate-binding protein 1 | PABPC1 | P11940 | CHEMBL1293286 | Unclassified protein | 0.115736675 | 16 / 0 |
| Tyrosine-protein kinase ABL | ABL1 | P00519 | CHEMBL1862 | Kinase | 0.115736675 | 209 / 0 |
| Cyclin-dependent kinase 4/cyclin D | CCND3 CCND1 CDK4 CCND2 | P30281 P24385 P11802 P30279 | CHEMBL2095942 | Other cytosolic protein | 0.115736675 | 33 / 0 |
| Calpain 1 | CAPN1 | P07384 | CHEMBL3891 | Protease | 0.115736675 | 141 / 0 |
| Sodium channel protein type II alpha subunit | SCN2A | Q99250 | CHEMBL4187 | Voltage-gated ion channel | 0.115736675 | 39 / 0 |
| Prostaglandin E synthase | PTGES | O14684 | CHEMBL5658 | Enzyme | 0.115736675 | 55 / 0 |
| Prostanoid FP receptor | PTGFR | P43088 | CHEMBL1987 | Family A G protein-coupled receptor | 0.115736675 | 1 / 0 |
| Type-1 angiotensin II receptor (by homology) | AGTR1 | P30556 | CHEMBL227 | Family A G protein-coupled receptor | 0.115736675 | 47 / 0 |
| Telomerase reverse transcriptase | TERT | O14746 | CHEMBL2916 | Enzyme | 0.115736675 | 32 / 0 |
| Cathepsin (V and K) | CTSV | O60911 | CHEMBL3272 | Protease | 0.115736675 | 45 / 0 |
| Intermediate conductance calcium-activated potassium channel protein 4 | KCNN4 | O15554 | CHEMBL4305 | Voltage-gated ion channel | 0.115736675 | 17 / 0 |
| Transitional endoplasmic reticulum ATPase | VCP | P55072 | CHEMBL1075145 | Primary active transporter | 0.115736675 | 62 / 0 |
| Melatonin receptor 1A | MTNR1A | P48039 | CHEMBL1945 | Family A G protein-coupled receptor | 0.115736675 | 421 / 0 |
| Melatonin receptor 1B | MTNR1B | P49286 | CHEMBL1946 | Family A G protein-coupled receptor | 0.115736675 | 373 / 0 |
| Caspase-3 | CASP3 | P42574 | CHEMBL2334 | Protease | 0.115736675 | 126 / 0 |
| Glycine transporter 1 | SLC6A9 | P48067 | CHEMBL2337 | Electrochemical transporter | 0.115736675 | 141 / 0 |
| Phosphodiesterase 4A | PDE4A | P27815 | CHEMBL254 | Phosphodiesterase | 0.115736675 | 124 / 0 |
| Phosphodiesterase 4B | PDE4B | Q07343 | CHEMBL275 | Phosphodiesterase | 0.115736675 | 193 / 0 |
| Arachidonate 15-lipoxygenase | ALOX15 | P16050 | CHEMBL2903 | Enzyme | 0.115736675 | 37 / 0 |
| Phosphodiesterase 4C | PDE4C | Q08493 | CHEMBL291 | Phosphodiesterase | 0.115736675 | 35 / 0 |
| Bcl2-antagonist of cell death (BAD) | BAD | Q92934 | CHEMBL3817 | Other cytosolic protein | 0.115736675 | 16 / 0 |
| PI3-kinase p110-alpha subunit | PIK3CA | P42336 | CHEMBL4005 | Enzyme | 0.115736675 | 280 / 0 |
| Induced myeloid leukemia cell differentiation protein Mcl-1 | MCL1 | Q07820 | CHEMBL4361 | Other cytosolic protein | 0.115736675 | 21 / 0 |
| Apoptosis regulator Bcl-X | BCL2L1 | Q07817 | CHEMBL4625 | Other ion channel | 0.115736675 | 10 / 0 |
| Apoptosis regulator Bcl-W | BCL2L2 | Q92843 | CHEMBL4677 | Other cytosolic protein | 0.115736675 | 2 / 0 |
| Bcl-2-like protein 10 | BCL2L10 | Q9HD36 | CHEMBL5988 | Unclassified protein | 0.115736675 | 1 / 0 |
| Bcl-2-related protein A1 | BCL2A1 | Q16548 | CHEMBL6044 | Unclassified protein | 0.115736675 | 14 / 0 |
| Corticotropin releasing factor receptor 1 | CRHR1 | P34998 | CHEMBL1800 | Family B G protein-coupled receptor | 0.115736675 | 438 / 0 |
| Cyclin-dependent kinase 5/CDK5 activator 1 | CDK5R1 CDK5 | Q15078 Q00535 | CHEMBL1907600 | Kinase | 0.115736675 | 156 / 0 |
| c-Jun N-terminal kinase 1 | MAPK8 | P45983 | CHEMBL2276 | Kinase | 0.115736675 | 277 / 0 |
| C-C chemokine receptor type 1 | CCR1 | P32246 | CHEMBL2413 | Family A G protein-coupled receptor | 0.115736675 | 90 / 0 |
| Beta amyloid A4 protein | APP | P05067 | CHEMBL2487 | Membrane receptor | 0.115736675 | 48 / 0 |
| Platelet activating factor receptor | PTAFR | P25105 | CHEMBL250 | Family A G protein-coupled receptor | 0.115736675 | 48 / 0 |
| Scavenger receptor class B member 1 | SCARB1 | Q8WTV0 | CHEMBL1914272 | Unclassified protein | 0.115736675 | 24 / 0 |
| Tryptophan 2,3-dioxygenase | TDO2 | P48775 | CHEMBL2140 | Enzyme | 0.115736675 | 12 / 0 |
| Metabotropic glutamate receptor 4 | GRM4 | Q14833 | CHEMBL2736 | Family C G protein-coupled receptor | 0.115736675 | 109 / 0 |
| Neuromedin B receptor | NMBR | P28336 | CHEMBL3636 | Family A G protein-coupled receptor | 0.115736675 | 44 / 0 |
| Neurokinin 3 receptor | TACR3 | P29371 | CHEMBL4429 | Family A G protein-coupled receptor | 0.115736675 | 172 / 0 |
| Indoleamine 2,3-dioxygenase | IDO1 | P14902 | CHEMBL4685 | Enzyme | 0.115736675 | 49 / 0 |
